# Azetidinylideneketenimines as a Multimodal Synthetic Platform: Coordination Chemistry and Cycloaddition‐Triggered Transformations

**DOI:** 10.1002/anie.1569919

**Published:** 2026-03-30

**Authors:** Taichi Koike, Keisuke Ito, Shintaro Ishida, Takeaki Iwamoto

**Affiliations:** ^1^ Department of Chemistry Graduate School of Science Tohoku University Sendai Japan; ^2^ Department of Chemistry and Biotechnology Graduate School of Engineering The University of Tokyo Tokyo Japan

**Keywords:** coordination chemistry, cumulene, cycloaddition, reactive organic species, ring opening

## Abstract

Methyleneketenimines, due to their highly unsaturated and CN‐cumulenic nature, represent a rare class of heterocumulenes with potential as building blocks for pharmaceuticals and molecular electronics. However, their systematic chemistry has been limited. Herein, we report a concise C═C═N homologation reaction, driven by the strain release of the three‐membered‐ring carbene, bis(diisopropylamino)cyclopropenylidene (**BAC**), which unlocks rapid and scalable access to a series of isolable methyleneketenimines, including the first example of a bis(methyleneketenimine), featuring terminal azetidine moieties (azetidinylideneketenimines). Mechanistic studies, supported by control experiments and computational analysis, elucidate the important role of a methyleneketeniminyl carbene intermediate in forming the target heterocumulene. Spectroscopic and electrochemical investigations show that the visible‐light absorption and redox potentials correlate with the degree of *N*‐substituent π‐conjugation. Most importantly, the highly polarized nature of the C═C═C═N framework enables a multimodal reactivity platform, spanning from unprecedented coordination to transition metals (Au(I), Rh(I)), CO_2_ splitting, alkyne C≡C bond insertion, to divergent skeletal rearrangements via (2 + 2) or (3 + 2) cycloaddition. This work establishes methyleneketenimines as a versatile toolkit for accessing complex heterocycles and novel organometallic complexes.

## Graphical abstract

Cross‐coupling/homologation sequence between a stable three‐membered‐ring carbene and isocyanides enables facile and rapid access to novel methyleneketenimines and the first bis(methyleneketenimine). Reactivity studies gave rise to the first methyleneketenimine‐transition metal complexes, unique multimodal reactivity ranging from CO_2_, isocyanate splitting, to formal alkyne C≡C bond insertion triggered via dipolar cycloaddition.

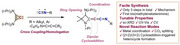

## 1 Introduction

The strategic incorporation of p‐block elements into π‐electron systems has revolutionized the design of functional materials, providing a versatile platform for developing next‐generation optoelectronics, stimuli‐responsive materials [1, 2, 3, 4, 5], and green catalysis [6]. Among such π‐electron species, heteroallenes and heterocumulenes, compounds featuring two or more consecutive double bonds, are reactive synthetic intermediates for the construction of complex heterocyclic architectures via cycloaddition reactions [7] or as candidates for next‐generation molecular electronics [8, 9, 10]. Recently, heteroallenes and heterocumulenes containing consecutive C═C and C═N bonds in the neutral or anionic state have gained great attention through the recent groundbreaking synthetic studies led independently by the groups of Gessner [11, 12, 13], Hansmann [14, 15, 16, 17], Liu [18, 19], Severin [20], and Xu [21].

**SCHEME 1:**
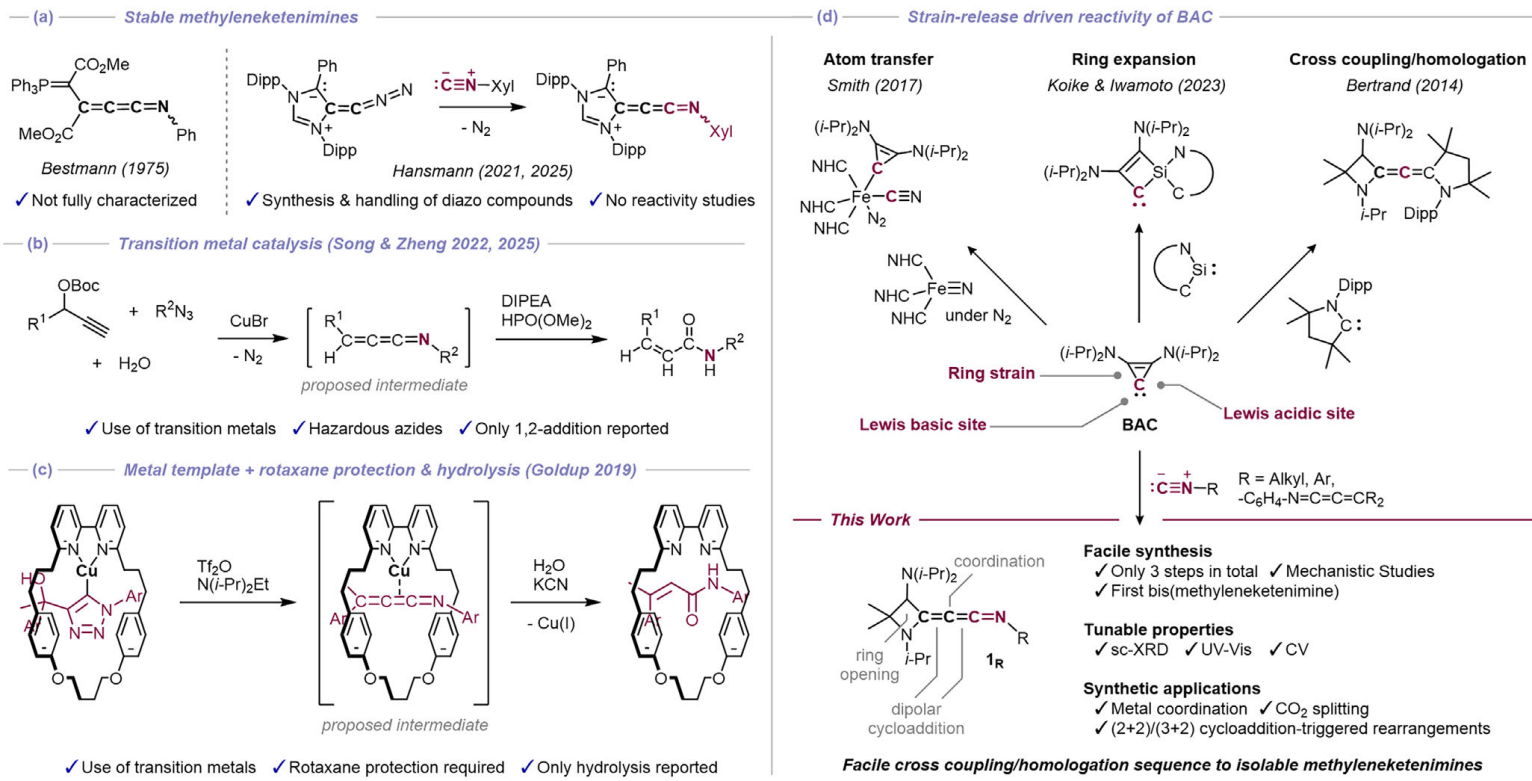
(a) Previous reports on persistent and stable methyleneketenimines. (b) Transition‐metal catalysis and (c) metal‐template/rotaxane‐protection strategy for the generation of transient methyleneketenimines and their application. (d) The diverse reactivity of bis(diisopropylamino)cyclopropenylidene (**BAC**) induced by ring opening and (this work) its application towards facile azetidinylideneketenimine synthesis (Tf = trifluoromethanesulfonyl, DIPEA = *N*,*N*‐diisopropylethylamine, Dipp =2,6‐diisopropylphenyl, Xyl = 2,6‐dimethylphenyl).

Among these, methyleneketenimines (R_2_C═C═C═NR) represent a rare and challenging class of aza‐cumulenes. Although the first persistent methyleneketenimine was reported by Bestmann and coworkers in 1975 (Scheme 1a), [22, 23] Goldup and coworkers reported in 2019 that a metal‐template strategy enables the capture of these species as transient intermediates within a rotaxane scaffold (Scheme 1c) [24]. Subsequently, Song, Zheng and coworkers developed copper‐catalyzed approaches using azides to generate transient methyleneketenimines, which could be intercepted via simple 1,2‐addition reactions such as hydrolysis or amination (Scheme 1b) [25, 26]. Simultaneously, Hansmann and coworkers achieved the first synthesis and crystallographic characterization of a stable, isolable methyleneketenimine, revealing its bent‐cumulenic structure (Scheme 1a) [14]. However, this latter study relies on the preparation of air‐, moisture‐, and light‐sensitive diazoalkenes (R_2_C═C═N_2_) [14, 15, 16, 17, 20], a step‐intensive process requiring the handling of high‐energy diazo compounds and nitrous oxide. Consequently, the systematic exploration of methyleneketenimine physical properties, and a reactivity landscape extending beyond simple 1,2‐additions has remained unexplored. This prompted us to establish a more rapid and modular method to access isolable methyleneketenimines to further probe their potential as both functional materials and synthetic reagents.

As a suitable reagent, we directed our attention to the highly strained yet isolable two‐coordinate carbon species, bis(diisopropylamino)cyclopropenylidene (**BAC**) [27, 28, 29]. **BAC** can be prepared just in two steps from commercially available chemicals and shares the electronic properties typical of *N*‐heterocyclic carbenes (NHCs) [30, 31, 32, 33], making it suitable for Lewis‐base catalysis [34, 35, 36], as a ligand for transition metal complexes [37, 38, 39, 40], and for stabilizing electron‐deficient reactive intermediates [41, 42, 43, 44, 45, 46]. However, the most characteristic property of **BAC** is its potential to undergo transformations induced by the strain‐release of the three‐membered ring [47, 48, 49, 50], in some cases acting even as a carbon atom surrogate (Scheme 1d) [51].

Strategically exploiting this strain release and ambiphilicity in synthetic chemistry is rarely pursued, as the robustness and stability of NHCs are typically prioritized. Inspired by the previous work of Bertrand and coworkers regarding homologative allene synthesis [52], as well as our recent studies on the skeletal editing of **BAC** [53], we report herein the facile and rapid synthesis, physical properties, and multimodal reactivity of a novel library of methyleneketenimines, featuring terminal azetidine moieties (azetidinylideneketenimines, Scheme 1d bottom).

## 2 Results and Discussion

### 2.1 Synthesis, Characterization, and Formation Mechanism

**FIGURE 1:**
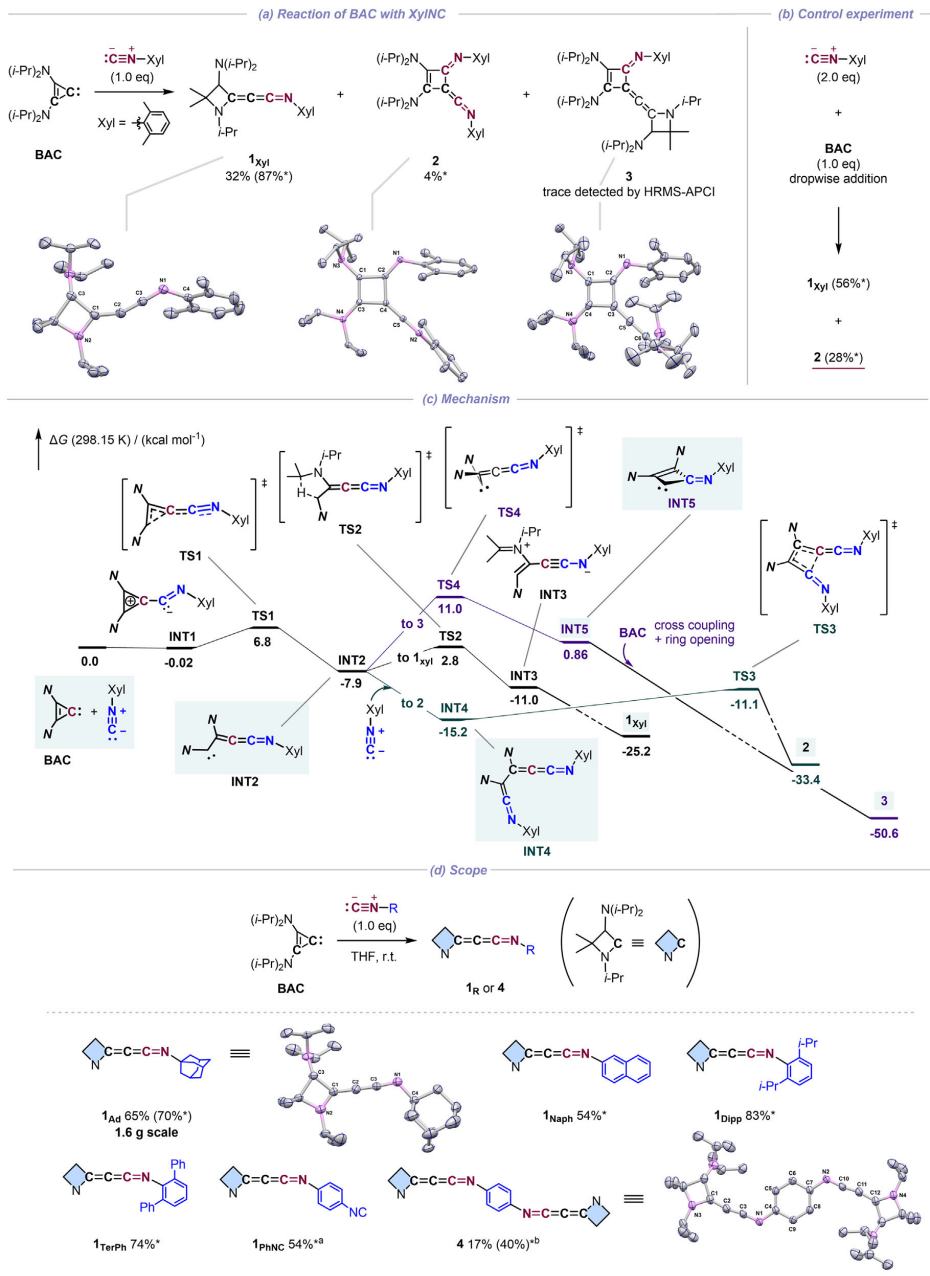
(a) Reaction of **BAC** with 1.0 equiv. of XylNC to give **1_Xyl_**, **2**, and **3** (top). (b) Control experiment aimed to increase the yield of **2**. (c) Proposed Gibbs energy profile for the reaction of **BAC** with XylNC (r^2^SCAN‐3c (SMD = THF); Δ*G* in kcal mol^−1^; ***N* =** N(*i*‐Pr)_2_). (d) Reaction of **BAC** with various isocyanides and their isolated yields. *Yields with asterisk are determined by ^1^H NMR integrals using 1,3,5‐tri‐*tert*‐butylbenzene as an internal standard. ^a^1.0 equiv. and ^b^0.5 equiv. of 1,4‐bis(isocyano)benzene used. Crystal structures of **1_Xyl_**, **2**, **3**, **1_Ad_**, and **4** are shown with ellipsoids at the 50% probability.

We initiated our investigation by reacting **BAC** with 1.0 equiv. of XylNC (Xyl = 2,6‐dimethylphenyl) in THF at ambient temperature. This straightforward procedure triggered a cross‐coupling/homologation sequence, affording the target azetidinylideneketenimine **1_Xyl_** as orange crystals in 32% yield (Figure 1a; 87% NMR yield based on ^1^H NMR integrals using an internal standard). Compound **1_Xyl_** readily undergoes hydrolysis, yielding the corresponding acrylamide (for details, see supporting information), which is consistent with the mechanism of the copper‐catalysis reported by Song, Zheng, and coworkers (Figure 1b) [25]. Nevertheless, **1_Xyl_** exhibits thermal stability up to 70°C in solution and could be unambiguously characterized by multinuclear NMR spectroscopy, high resolution mass spectrometry (HRMS‐APCI), and elemental analysis.

Single‐crystal X‐ray diffraction (sc‐XRD) analysis confirmed the formation of a slightly bent heterocumulenic C═C═C═N core. The asymmetric unit contains two independent molecules, which exhibit C1═C2═C3 bond angles of 156.37(16)° and 168.35(15)°, respectively. To the best of our knowledge, **1_Xyl_** represents the first structurally characterized alkyl‐substituted methyleneketenimine and the first derivative featuring an azetidine moiety. The observed bond lengths (C1═C2 1.343(2) Å/1.340(2) Å; C2═C3 1.259(2) Å/1.249(2) Å; C3═N1 1.244(2) Å/1.254(2) Å) are comparable to those of the triazole‐incorporated methyleneketenimines synthesized by Hansmann and coworkers [14, 15, 16, 17]. Notably, this comparison suggests that the electron‐donating ability of the terminal *N*‐heterocycle exerts minimum influence on the structural parameters of the cumulenic core [54, 55].

Prompted by the unexpectedly low isolation yield of **1_Xyl_** caused by minor crystalline by‐products, the by‐product distribution and reaction mechanism were investigated. This analysis revealed two rare conjugated cyclobutenes: keteniminylidene cyclobutene **2** (4% NMR yield) and allenylidene cyclobutene **3** (detected from HRMS‐APCI spectrum), alongside the major product **1_Xyl_** from the aforementioned reaction mixture (Figure 1a). The structures of cyclobutenes **2** and **3** were further confirmed by sc‐XRD analysis (Figure 1a). To further probe the formation pathway of **2**, the reaction conditions were modified. The dropwise addition of a **BAC** (1.0 equiv.) solution to a vigorously stirred THF solution of XylNC (2.0 equiv.) significantly increased the yield of **2%** to 28% yield while decreasing **1_Xyl_** to 56% (Figure 1b). In contrast, the formation of **3** could not be promoted by reversing the addition sequence, suggesting that the formation of **3** is kinetically disfavored.

DFT calculations (r^2^SCAN‐3c level (SMD = THF); Δ*G* (298.15 K) in kcal mol^−1^) conducted for the reaction between **BAC** and XylNC further gave insights into the selectivity between **1_Xyl_**, **2**, and **3** (Figure 1c). The barrierless cross coupling of **BAC** with XylNC (**INT1**; −0.02 kcal mol^−1^), followed by a ring opening of the three‐membered ring forms the reaction's key intermediate, methyleneketeniminyl carbene **INT2** (−7.9 kcal mol^−1^), which branches into three different pathways. The most favored pathway starts with the barrierless cross coupling of **INT2** with XylNC to give **INT4** (−15.2 kcal mol^−1^), which undergoes (2 + 2) cycloaddition (**TS3**: −11.1 kcal mol^−1^) to eventually form **2** (−33.4 kcal mol^−1^). Given that XylNC is consumed to generate **INT1**, the predominant pathway for **INT2** involves a proton‐transfer/barrierless‐bond‐formation sequence (**TS2**: −2.8 kcal mol^−1^) to afford the desired azetidinylideneketenimine **1_Xyl_** (−25.2 kcal mol^−1^), which resembles the case seen in some literatures [47, 52]. The computational results are consistent with experimental observations, where increasing the equivalent of XylNC shifts the product distribution toward **2**.

Conversely, the ring‐closure of **INT2** to cyclobutenylidene **INT5** (0.86 kcal mol^−1^) is kinetically disfavored (**TS4**: 11.0 kcal mol^−1^). The generated **INT5** is subsequently trapped by additional XylNC or **BAC** to furnish **2** or **3** (−50.6 kcal mol^−1^). The low conversion to **INT5** is consistent with the lack of driving force for the ring closure of **INT2**. This stands in sharp contrast with our previously reported cyclic (alkyl)silylene systems [53, 56, 57], where the electropositive silicon atom facilitates this cyclization.

The versatility of this homologation strategy was demonstrated by extending the scope to *N*‐alkyl (Ad = adamantyl) and other *N*‐aryl (2‐naphthyl, 2,6‐diisopropylphenyl (Dipp), 2,6‐diphenylphenyl (TerPh)) isocyanides, which afforded the corresponding azetidinylideneketenimines in good yield (Figure 1d). The *N*–Ad derivative **1_Ad_** was isolable on a gram scale (1.6 g, 4.1 mmol, 65%) as colorless crystals. Furthermore, the reaction of **BAC** with 1,4‐bis(isocyano)benzene (1.0 equiv.) yielded the isocyano‐functionalized azetidinylideneketenimine **1_PhNC_** alongside the first bis(methyleneketenimine) **4**. Azetidinylideneketenimines **1_Naph_**, **1_Dipp_**, **1_TerPh_**, and **1_PhCN_** were not isolated due to their oily nature, but were characterized by multinuclear NMR, HRMS‐APCI spectra analysis, and could also be directly derivatized to *N*‐heterocycles by one‐pot trapping reactions (vide infra). Notably, the inversion barrier of the nitrogen lone pair electrons in ketenimines (*E*/*Z* isomerization) are known to be typically low at room temperature, [58] which is consistent with the observation of a single set of signals in the NMR spectra for the methyleneketenimines synthesized herein and by Hansmann and coworkers [14, 15, 16, 17]. Importantly, compound **4** was successfully isolated as orange crystals in 17% yield (40% NMR yield) by treating **BAC** with 0.5 equiv. of the bis(isocyanide). Although the structural parameters of the C═C═C═N core in **4** [C1═C2 1.345(3) Å, C2═C3 1.245(3) Å, C3═N1 1.256(3) Å; C1═C2═C3 168.1(2)°] are comparable to those of **1_Xyl_**, the coplanar conformation of the entire azetidine─CCCN─Ph─NCCC–azetidine backbone suggests significant π‐conjugation between the two azetidine moieties through the phenylene bridge.

### 2.2 Photophysical and Electronic Properties

**FIGURE 2:**
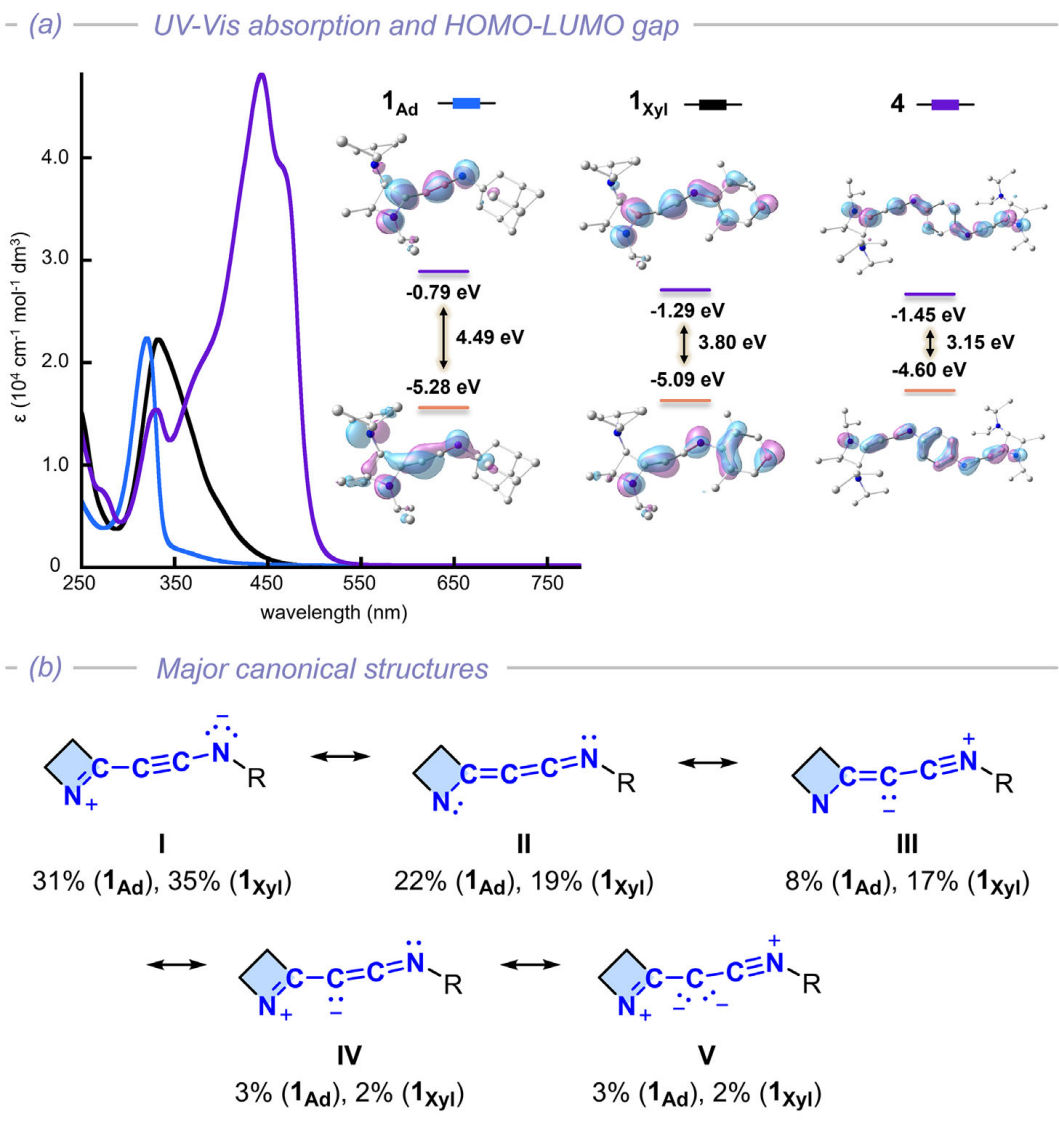
(a) UV–vis absorption spectra overlay of **1_Ad_**, **1_Xyl_**, and **4** in THF at room temperature (left) and their calculated HOMO‐LUMO energy gap (right). (b) Selected major canonical structures of **1_Ad_** and **1_Xyl_** obtained by NRT analysis. (Xyl = 2,6‐dimethylphenyl, Ad = 1‐adamantyl).

Compounds **1_Xyl_**, **1_Ad_**, and **4** served as ideal candidates to systematically probe the electronic influence of the *N*‐substituent on the C═C═C═N framework. Accordingly, their electronic profiles were investigated using UV–vis spectroscopy (Figure 2a), cyclic voltammetry (CV; Figures S225–S230), and DFT calculations (Figure 2b). UV‐Vis absorption spectra in THF exhibit intense bands assigned to the π(C═C═C═N) → π*(C═C═C═N) transition. A substantial bathochromic shift is observed in the order of **1_Ad_** (365 nm), **1_Xyl_** (400 nm), and **4** (467 nm), which is consistent with the cathodic shift observed for the irreversible oxidation events (oxidation potentials (V vs. Fc/Fc^+^ in acetonitrile); **1_Ad_**: 0.25; **1_Xyl_**: 0.17, **4**: 0.03) and the anodic shift of the irreversible reduction events (**1_Ad_**: −3.00 V; **1_Xyl_**: −2.75 V; **4**: −2.70 V) from CV measurements. TD‐DFT calculations conducted at the B3LYP‐D3(BJ)/6‐311++G(d,p)/r^2^SCAN‐3c level (SMD = THF) corroborate the experimental data, predicting a narrowing of the HOMO‐LUMO gap (**1_Ad_**: 4.49 eV, **1_Xyl_**: 3.80 eV, **4**: 3.15 eV) that mirrors the red shift of the absorption (Figure 2a). Overall, these results demonstrate that the electronic properties of the methyleneketenimine core can be drastically tuned via the π‐nature of the *N*‐substituent.

While the Mayer bond order analysis supports a hetero [3] cumulene with three consecutive double bonds, natural population analysis (NPA) charge analysis reveals higher electron density on the central C2 and terminal N1 atoms (C1 0.24, C2 −0.36, C3 0.35, N1 −0.49). This polarization is consistent with the natural resonance theory (NRT) analysis (Figures 2c and S238) which describes the electronic structure as a hybrid of a push‐pull acetylene **I** (**1_Xyl_** 35%; **1_Ad_** 31%) and a [3] cumulene **II** (**1_Xyl_** 19%; **1_Ad_** 22%). Notably, NRT analysis also indicates non‐negligible contributions from zwitterionic C2‐vinyl anion **III** (**1_Xyl_** 17%; **1_Ad_** 8%), zwitterionic ketenimine **IV** (**1_Xyl_** 2%; **1_Ad_** 3%), and carbone **V** (**1_Xyl_** 2%; **1_Ad_** 3%) resonance forms. The relatively low weight of resonance structures featuring a nucleophilic C2 atom is consistent with the observed high linearity of the C1═C2═C3═N1 core as well as previous theoretical studies on similar heterocumulene systems (R_2_C═C═N═N, R_2_C═C═C═O) [59, 60]. Notably, the weight of these resonance contributors is likely to be more influenced by the linearity of the heterocumulene framework than by the electronic nature of the *N*‐substituent (C1═C2═C3 angle, **1_Xyl_** 165.1°; **1_Ad_** 169.2°).

### 2.3 Coordination Chemistry

**FIGURE 3:**
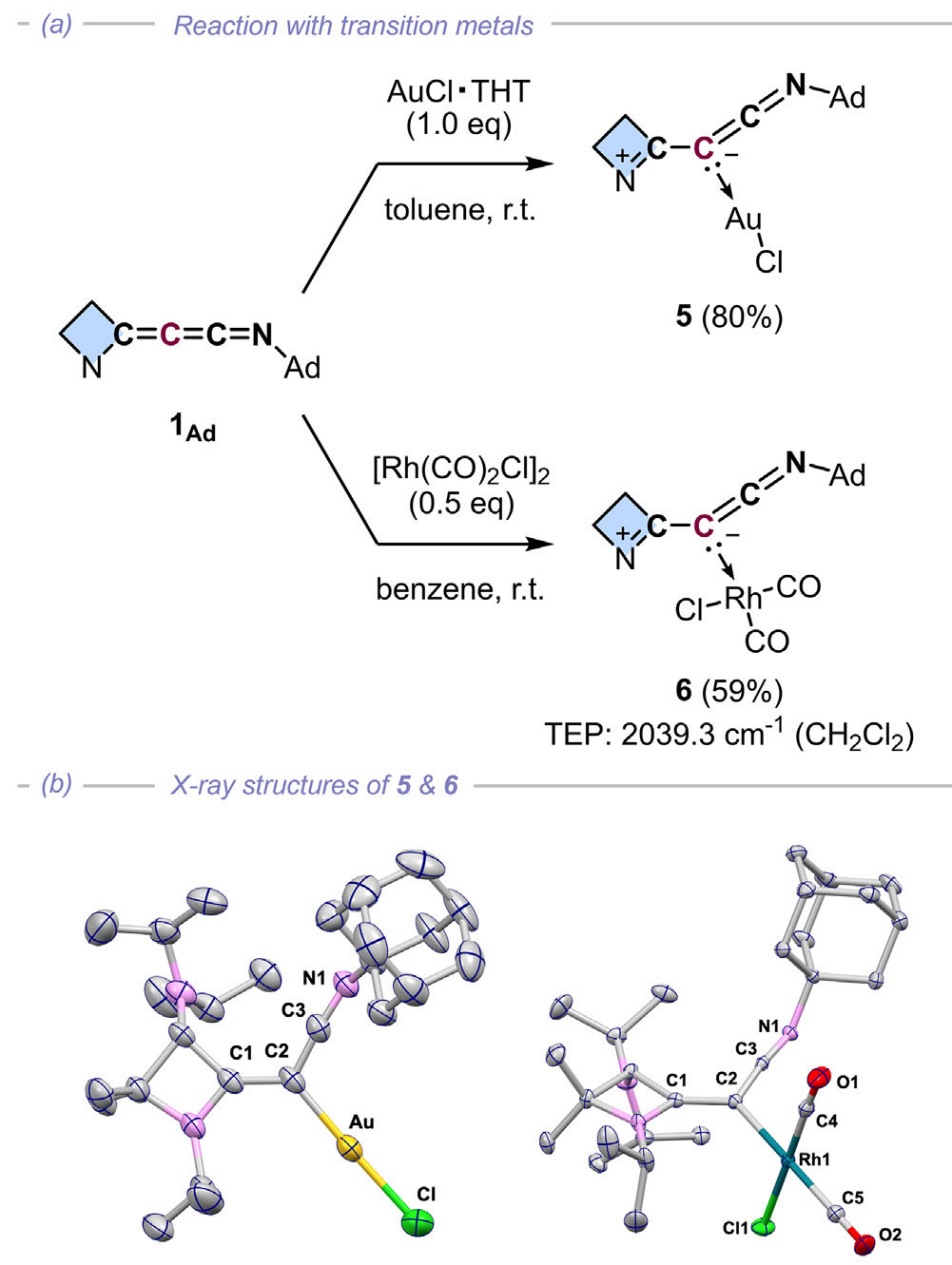
(a) Metal coordination of azetidinylideneketenimine **1_Ad_** and (b) the x‐ray structures of Au(I) complex **5** (left) and Rh(I) complex **6** (right). (Ad = 1‐adamantyl).

The coordination ability of **1_Ad_** was demonstrated by its reaction with Rh(I) and Au(I) sources, yielding the first methyleneketenimine transition metal complexes **5** (80% yield) and **6** (59% yield) via *η* ^1^‐coordination of the C2 atom (Figure 3). This C2‐selectivity aligns with the relatively high electron density on the C2 atom (vide infra) as well as with the trends observed for diazoalkene complexes [14, 61, 62], although competitive *N*‐coordination cannot be entirely ruled out depending on reaction conditions. The X‐ray structures of **5** and **6** reveal a substantial decrease in the central C1─C2─C3 bond angle (**5**, 118.0(3)°; **6**, 117.3(1)°) as well as the elongation of the C1─C2 bond (**5**, 1.393(2) Å; **6**, 1.404(2) Å). These structural features point toward a predominant contribution of a zwitterionic ketenimine resonance structure similar to that of **IV** (Figure 2c), which also agrees well with the NPA charge analysis of **1_Ad_** discussed above.

The Tolman electron parameter (TEP) of **6** (2039.34 cm^−1^, Figure 3), calculated using the Rh‐to‐Ni linear regression equation [54, 55], is exceptionally low compared to cyclic aminocarbenes and mesoionic carbenes (TEP: 2042–2069 cm^−1^) [54, 55] but falls into the lower end of carbones and bent allenes (TEP: 2023–2036 cm^−1^) [54, 55, 63, 64, 65]. This result implies that azetidinylideneketenimines could be utilized as carbon‐centered ligands which fills the gap of electron‐donating ability between classical NHCs and stronger donor carbones.

### 2.4 Cycloaddition‐Triggered Transformations

**FIGURE 4:**
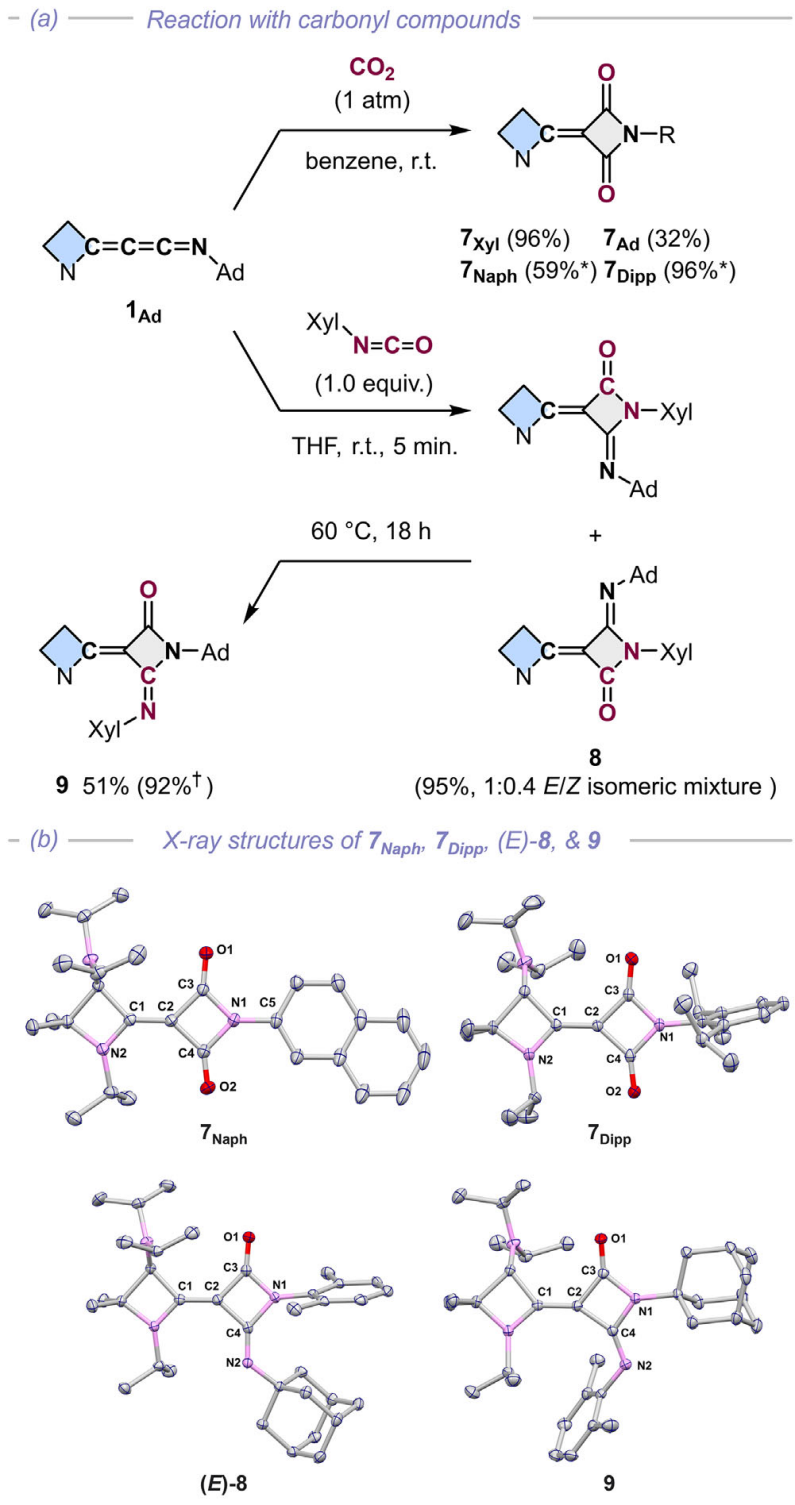
(a) Reactions of azetidinylideneketenimines with CO_2_, XylNCO, and (b) the X‐ray structures of **7_Naph_** (top left), **7_Dipp_** (top right), (*E)*‐**8** (bottom left), and **9** (bottom right). (b) The X‐ray structures of azetidine‐2,4‐dione. *The reaction yield is based on in situ generated **1_R_**. †The reaction yield is based on ^1^H NMR integrals. (Xyl = 2,6‐dimethylphenyl, Ad = 1‐adamantyl).

**FIGURE 5:**
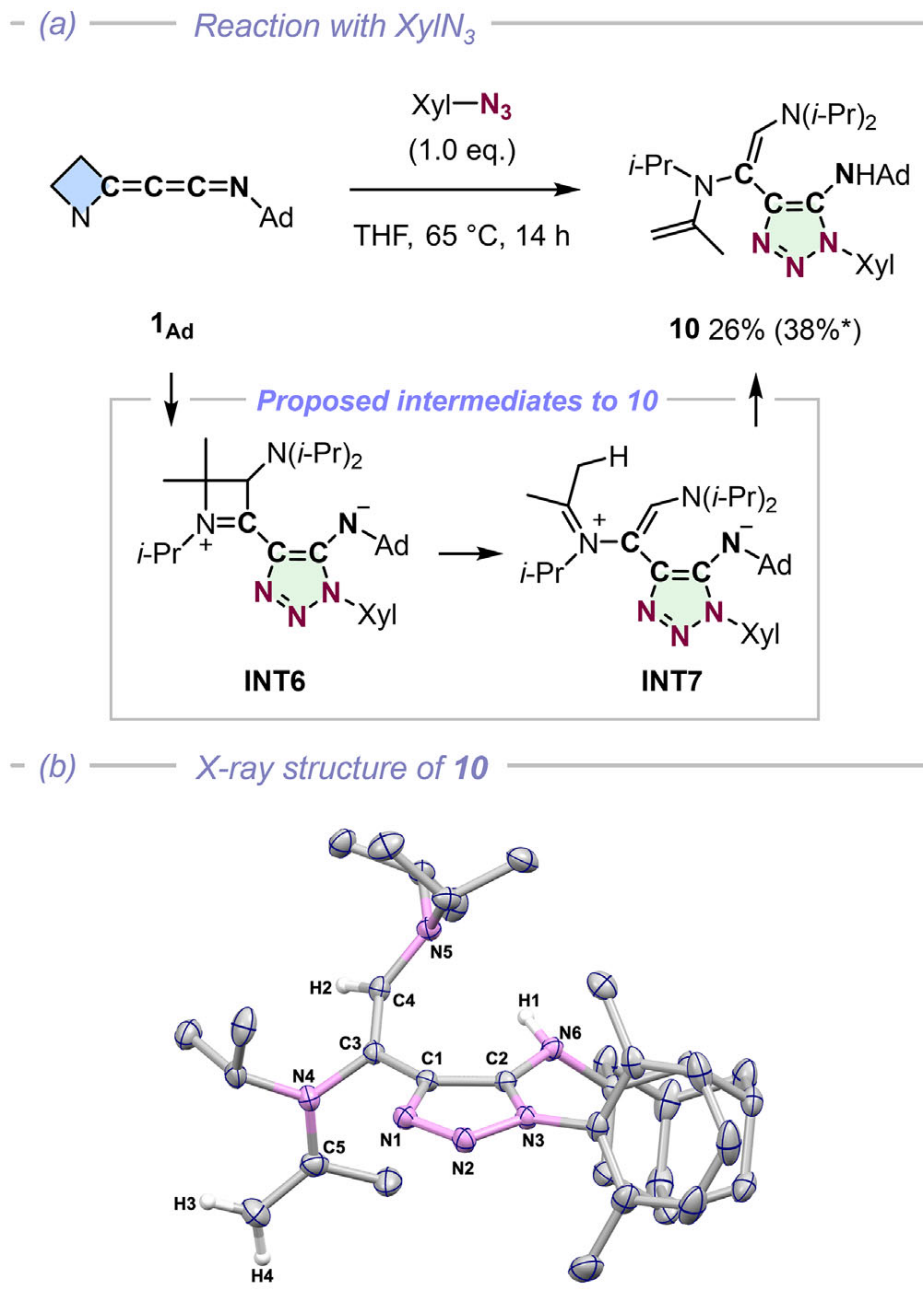
(a) Reaction of azetidinylideneketenimine **1_Ad_** with XylN_3_ and (b) the X‐ray structure of **10**. *The reaction yield is based on ^1^H NMR integrals. (Xyl = 2,6‐dimethylphenyl, Ad = 1‐adamantyl).

**FIGURE 6:**
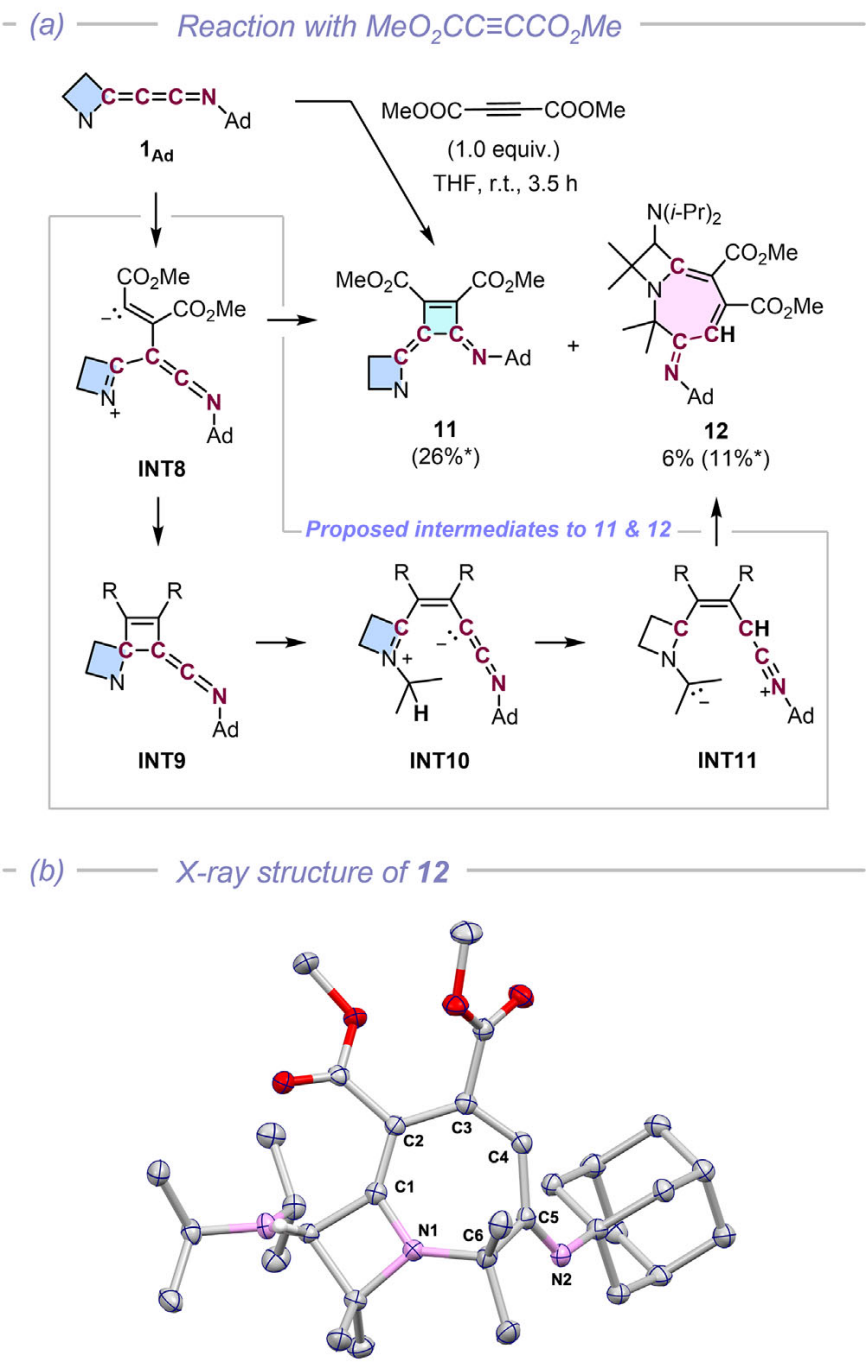
(a) Reaction of azetidinylideneketenimine **1_Ad_** with dimethyl acetylenedicarboxylate and (b) the X‐ray structure of **12**. *The reaction yield is based on ^1^H NMR integrals. (Ad = 1‐adamantyl).

Reactivity studies toward CO_2_ as well as typical organic dipoles (Figures 4, 5, 6) proved that the azetidinylideneketenimine scaffold serves as a potent reagent for constructing strained or multi‐functionalized N‐heterocycles. Remarkably, these species cleave the strong C–O bond of CO_2_ under ambient conditions to afford rare azetidine‐2,4‐diones **7** (R = Xyl, Ad, 2‐Naph, Dipp) in good yields (Figure 4). Mechanistically, this likely proceeds via a (2+2) cycloaddition followed by a C–O cleavage/N–C bond formation sequence (for details, see Scheme S1), analogous to phosphoranylidene ketenimines but unprecedented for this class of heterocumulenes [66].

The treatment of **1_Ad_** with 1.0 equiv. of xylyl isocyanate at ambient temperature initially formed azetidine‐2‐imine‐4‐one **8** as a *E*/*Z* isomeric mixture ((*E*)‐**8**: 67%; (*Z*)‐**8**: 28%; Figure 4). Upon heating at 60°C for 18 h, this mixture was converted exclusively to isomer **9** (92% yield). This isomerization implies a ring‐opening/ring‐closing sequence, mechanistically reminiscent of the CO_2_ activation pathway (for details, see Scheme S2).

More profound skeletal reorganization was observed with xylyl azide. Although sluggish at room temperature, the reaction of **1_Ad_** with xylyl azide at 65°C for 14 h promoted a cascade reaction yielding triazole **10** in 38% yield (Figure 5). This transformation is proposed to proceed via (3 + 2) dipolar cycloaddition (**INT6**), followed by azetidine ring opening (**INT7**) and proton transfer (for details, see Scheme S3). This represents a double skeletal deconstruction, wherein both the original three‐membered ring of **BAC** and the transient four‐membered ring of the azetidine intermediate (**INT6**) are cleaved to assemble complex peripheral functionality on the resulting *N*‐heterocycle. Although the low yield and complex rearrangement sequence hints toward other byproducts, characterization of the other minor byproducts was unsuccessful.

While **1_Ad_** proved inert toward electron‐rich alkenes and alkynes (e.g. cycloheptene, phenylacetylene, bis(trimethylsilyl)acetylene; for details of the unreactive substrates, see Scheme S4), **1_Ad_** readily reacted with the electron‐deficient dimethyl acetylenedicarboxylate. This reaction proved less selective, diverging to yield cyclobutene **11** and a formal C≡C triple bond insertion product, azacycloheptadiene **12** (Figure 6). Compound **13** was characterized by multinuclear NMR spectrometry and GIAO calculation (Figure S239) and the structure of **12** was unambiguously determined by sc‐XRD analysis (Figure 6).

This result stands in contrast with the dipolar cycloaddition reactions discussed before, as the formation of **11** and **12** implies that both the terminal C1═C2 and central C2═C3 double bond could participate in dipolar cycloaddition reactions with the same substrate. This presumably occurs via common intermediate **INT8**, which likely accounts for the low selectivity. The carbanion of **INT8** could attack either the C3 or C1 atom to afford cyclobutene **11** or **INT9**. The ring strain in the spirocycle of **INT9** likely facilitates a retro‐(2+2) cycloaddition to give the reactive methyleneketenimine **INT10**, followed by a proton migration (or abstraction; **INT11**), and ring closure to eventually form the seven‐membered ring (Figure 6; for details, see Scheme S4). The aforementioned reactivity of **1_Ad_** towards organic dipoles highlights the diverse molecular scaffolds accessible with azetidinylideneketenimines.

## 3 Conclusion

By utilizing an isolable three‐membered‐ring carbene, we have established a facile strain‐release strategy that enables the access to rare nitrogen‐doped heterocumulenes, specifically isolable azetidine‐incorporated methyleneketenimines and the first bis(methyleneketenimine). The redox potentials and optical properties of these species can be modulated simply by varying the π‐conjugation of the starting isocyanide. The facile and rapid synthesis unlocked the multimodal synthetic potential of methyleneketenimines: they function as *η* ^1^‐ligands and serve as useful synthons for accessing diverse heterocycles, including azetidine‐2,4‐diones, azetidine‐2‐imine‐4‐ones, triazoles, azacycloheptadienes, and various functionalized cyclobutenes via cycloaddition‐triggered transformations. We anticipate that this strategy for constructing nitrogen‐doped cumulenes will serve as a versatile platform for the development of next‐generation organic materials and complex heterocyclic architectures.

## Conflicts of Interest

The authors declare no conflicts of interest.

## Supporting information




**Supporting File 1**: The authors have cited additional references within the Supporting Information^[68–86]^. Crystallographic data for the structures reported in this article have been deposited at the Cambridge Crystallographic Data Centre [67].

**Supporting File 2**: anie72019‐sup‐0002‐Data.zip.

## Data Availability

The data that support the findings of this study are available in the Supporting Information of this article.
