# Supplementary material for: Azetidinylideneketenimines as a Multimodal Synthetic Platform: Coordination Chemistry and Cycloaddition‐Triggered Transformations
Source: Angew Chem Int Ed Engl. 2026 Mar 30;65(20):e1569919. doi: 10.1002/anie.1569919 (PMC13159403; doi:10.1002/anie.1569919)
Supplement: Supplementary file 1 — Supporting File 1: The authors have cited additional references within the Supporting Information[68–86]. Crystallographic data for the structures reported in this article have been deposited at the Cambridge Crystallographic Data Centre [67]. [file ANIE-65-e1569919-s002.pdf]

Supporting Information

Azetidinylideneketenimines as a Multimodal Synthetic Platform: Coordination Chemistry and Cycloaddition-Triggered Transformations

Taichi Koike,[*1,2] Keisuke Ito,[1] Shintaro Ishida,[1] Takeaki Iwamoto[*1]

[1]*Department of Chemistry, Graduate School of Science, Tohoku University, Aoba-ku, Sendai 980-8578, Japan*
[2]*Department of Chemistry and Biotechnology, Graduate School of Engineering, The University of Tokyo, Bunkyo-ku, Tokyo 113-8656, Japan*

Table of Contents

1. Experimental Details S2
2. NMR Spectra S15
3. HRMS Spectra S104
4. X-ray Analysis S110
5. UV-Vis Absorption Spectra S118
6. FT-IR Spectra S121
7. Cyclic Voltammetry S133
8. Computational Studies S136
9. References S158

Experimental Details

General procedures

All reactions treating air-sensitive compounds were carried out under Ar atmosphere using a high-vacuum line, standard Schlenk techniques, or a glovebox, as well as dry and oxygen-free solvents. ^{1}H, ^{13}C nuclear magnetic resonance (NMR) spectra were recorded on a Bruker Biospin Avance 500 FT NMR spectrometer at room temperature (r.t.) unless otherwise noted. ^{1}H NMR chemical shifts were referenced to the peaks of residual protons of $CDCl_3$ (δ 7.26), C_6D_6 (δ 7.16), THF-d_8 (δ 1.72).[68] ^{13}C NMR chemical shifts in $CDCl_3$, C_6D_6 or THF-d_8 were relative to $SiMe_4$ in ppm. Data are reported as follows: chemical shift, multiplicity (s = singlet, d = doublet, t = triplet, sep = septet, brs = broad singlet, brd = broad doublet, m = multiplet), coupling constant in hertz (Hz), and an integration value. Melting points (m.p.) were measured by an OptiMelt. High-resolution mass spectra were performed on a Bruker Daltonics solariX 9.4T FT-ICR spectrometer using the APCI method or a JOEL JMS-T100GCV spectrometer using a FT+(eiFi) method. Elemental analysis was performed on a J-SCIENCE LAB JM-11 for CHN elements at the Research and Analytical Center for Giant Molecules (Graduate School of Science, Tohoku University). Cyclic voltammetry (CV) was performed on an ALS/chi-600A electrochemical analyzer. The CV cell consisted of a glassy carbon electrode, a Pt wire counter electrode, and an Ag/AgNO reference electrode. The reactions were performed under dry argon atmosphere unless otherwise noted. Sampling of air-sensitive compounds was carried out using a VAC NEXUS 100027 type glovebox. UV-vis spectra were recorded on a JASCO V-770 spectrometer. IR spectra were recorded on a HORIBA FT-720 spectrometer.

Materials

Dry and degassed benzene, hexane, toluene, and THF were prepared using a VAC 103991 solvent purifier. Et_2O was distilled over lithium aluminum hydride and degassed prior to use. C_6D_6 was degassed and dried by MS 4Å prior to use. THF-d_8 (C_4D_8O) was degassed and preserved in the presence of a potassium mirror prior to use. Bis(diisopropylamino)cyclopropenylidene (**BAC**),[27] 2,6-diisopropylphenyl isocyanide (DippNC),[69] 2,6-diphenylphenyl isocyanide (terPhNC),[70,71] 1,4-bis(isocyano)benzene,[72] and AuCl(THT)[73] were prepared according to the published procedures. All other chemicals were of reagent grade and used without further purification.

Preparation of Bis(diisopropylamino)cyclopropenylidene (BAC)

BAC was prepared with modification to the literature procedure[29] as follows. A 1:1 mixture of the cyclopropenium salt ([DAC–H]BPh_4, 4.50 g, 8.08 mmol) and potassium bis(trimethylsilyl)amide (1.63 g, 8.13 mmol) was cooled to -78 °C, and Et_2O (80 mL) was slowly added. The suspension was stirred for 10 minutes and then warmed up to room temperature and stirred for 30 minutes. After evaporation of the solvent under vacuum, hexane (120 mL) was added to the mixture, and the insoluble material was removed by filtration, affording a yellow solution. After the yellow solution was evaporated, a yellow solid was obtained. The solid was recrystallized from hexane twice (6.0 mL, 4.0 mL) to give **BAC** as pale-yellow crystals in 74% yield (1.41 g, 5.97 mmol).

pale yellow crystals; mp 111-113 °C; ^{1}H NMR (500 MHz, C_6D_6, 296 K, δ) 3.64 (sep, ^3J(H,H) = 6.7 Hz, 4H, CH(*i*-Pr)), (brs, 24H, CH_3(*i*-Pr)); ^{13}C{^{1}H} NMR (126 MHz, C_6D_6, 298 K, δ) 21.8 (CH_3), 51.1 (CH), 158.8 (C), 185.3($C_{carbene}$).

Reaction of BAC with XylNC (Xyl = 2,6-dimethylphenyl)

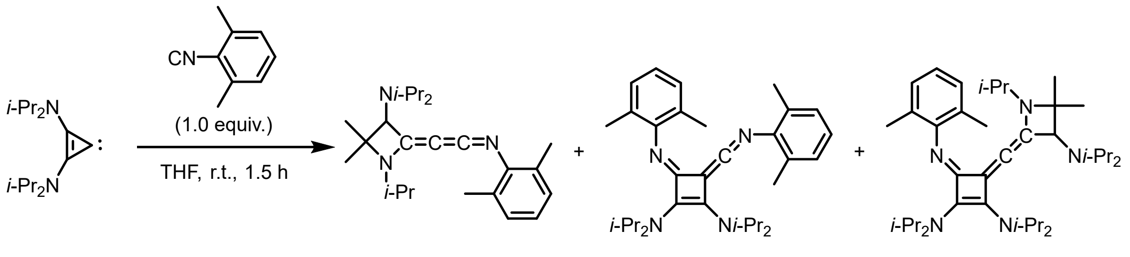

BAC | **1$_{Xyl}$** 87%* | **2** 4%* | **3** detected by HRMS and XRD

In a sample tube (5 mL) equipped with a magnetic stir bar, THF (1.0 mL) was added to the mixture of **BAC** (20 mg, 0.085 mmol) and xylyl isocyanide (11 mg, 0.084 mmol). The mixture was stirred at room temperature for 1.5 hours. The formation of **1$_{Xyl}$** (87% yield) and **2** (4% yield) was confirmed by NMR spectra analysis. The yields were determined by ^{1}H NMR integrals using 1,3,5-tri-*tert*-butylbenzene as an internal standard. The formation of **3** was confirmed by HRMS spectra and X-ray analysis (Figure S200).

3: HRMS (FD+eiFi) (*m*/*z*): Calcd for $C_{39}H_{65}N_5$ $[M]^+$ 603.52400, Found. 603.52241.

Isolation of Methyleneketenimine 1_{Xyl} (Xyl = 2,6-dimethylphenyl)

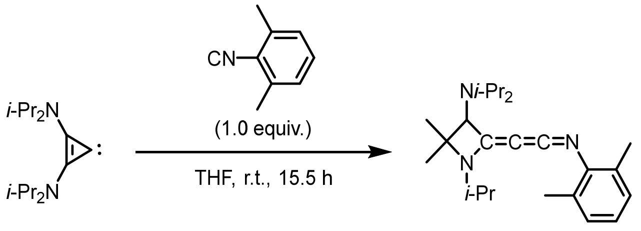

BAC $\mathbf{1_{Xyl}}$

In a sample tube (10 mL), THF (1.0 mL) was added to the mixture of **BAC** (101 mg, 0.43 mmol) and xylyl isocyanide (56 mg, 0.42 mmol). The mixture was stirred at room temperature for 15.5 hours to give a reddish black solution. After removing the volatiles in vacuo, the resulting brown oil was recrystallized from hexane:hexamethyldisiloxane (1:1) solution (0.8 mL) at –35 °C to give pale yellow crystals and a brown solution. The brown solution was evaporated to 0.4 mL and cooled to –35 °C to give orange crystals of $\mathbf{1_{Xyl}}$ in 32% yield (51 mg, 0.14 mmol).

$\mathbf{1_{Xyl}}$: orange crystals; mp 69-70 °C; ^{1}H NMR (500 MHz, C_6D_6, 296 K, δ) 0.83 (brd, ^3J(H,H) = 6.5 Hz, 6H, CH_3(*i*-Pr)), 0.89 (s, 3H, CH_3), 1.00-1.02 (s+d+d, 3H+3H+3H, CH_3+CH_3(*i*-Pr)+CH_3(*i*-Pr)), 1.12 (brd, ^3J(H,H) = 5.5 Hz, 6H, CH_3(*i*-Pr)), 2.65 (s, 6H, CH_3(Ar)), 3.10 (brs, 2H, CH(*i*-Pr)), 3.27 (sep, ^3J(H,H) = 7.0 Hz, 1H, CH(*i*-Pr)), 3.72 (s, 1H, CH), 6.92 (t, ^3J(H,H) = 7.5 Hz, 1H, CH(Ar)), 7.05 (d, ^3J(H,H) = 7.5 Hz, 2H, CH(Ar)); ^{13}C{^{1}H} NMR (126 MHz, C_6D_6, 297 K, δ) 19.8 (CH_3(Ar)), 20.8 (CH_3), 21.3 (CH_3(*i*-Pr)), 22.6 (CH_3(*i*-Pr)), 24.3 (CH_3(*i*-Pr)), 25.3 (CH_3), 47.4 (CH), 70.8 (CH), 73.1 (C), 78.5 (C*C*CN), 123.0 (CH(Ar)), 128.7 (CH(Ar)), 131.2 (C(Ar)), 145.4 (C(Ar)), 150.7 (CC*C*N), 151.7 (*C*CCN). One *C*H(*i*-Pr) signal and one *C*H_3(*i*-Pr) signal could not be determined due to signal overlapping; UV-vis (THF, 293 K) λ_{max}/nm (ε) 244 (1.6× 10^4), 332 (2.2 × 10^4), 360 (sh, 1.6 × 10^4), 400 (sh, 5.8 × 10^3), 445 (sh, 1.1 × 10^4); HRMS (FD+eiFi) (*m*/*z*): Calcd for $C_{24}H_{37}N_3$ $[M]^+$ 367.29875, Found. 367.29865; Elem. Anal. Calcd for $C_{24}H_{37}N_3$: C, 78.42%; H, 10.15%; N, 11.43%. Found: C, 78.55%; H, 10.12%; N, 11.60%.

Isolation of Cyclobutene 2

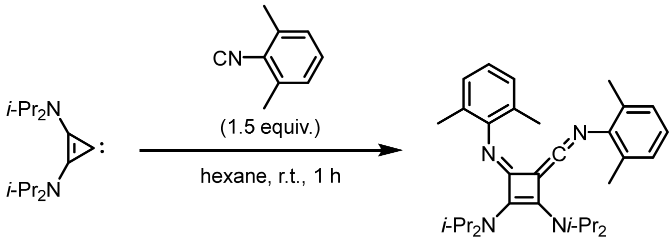

BAC **2**

In a Schlenk flask (50 mL) equipped with a magnetic stir bar, a hexane solution (2.0 mL) of xylyl isocyanide (65 mg, 0.50 mmol) was placed at room temperature and vigorously stirred. To the solution, **BAC** (59 mg, 0.25 mmol) in hexane (8.0 mL) was added dropwise (1 drop in 5 seconds) and stirred for 1 hour to give a dense orange solution. After removing in vacuo, the resulting red brown oil was recrystallized from Et_2O (0.5 mL) at -23 °C to give red orange crystals. The red orange crystals were washed with -23 °C hexane (0.5 mL) two times to give **2** as an orange solid in 23% yield (28 mg, 0.056 mmol).

2: orange solid; m.p. 152-154 °C; ^{1}H NMR (500 MHz, C_6D_6, 295 K, δ) 0.97 (d, ^3J(H,H) = 6.5 Hz, 12H, CH_3), 1.36 (d, ^3J(H,H) = 6.5 Hz, 12H, CH_3), 2.16 (s, 6H, CH_3), 2.52 (s, 6H, CH_3), 3.61 (sep, ^3J(H,H) = 6.5 Hz, 2H, CH), 4.35 (brs, 2H, CH), 6.71 (t, ^3J(H,H) = 7.5 Hz.1H, CH), 6.78 (s, 3H, CH), 6.94 (d, ^3J(H,H) = 7.5 Hz, 2H, CH); ^{13}C{^{1}H} NMR (126 MHz, C_6D_6, 298 K, δ) 19.1 (CH_3), 19.4 (CH_3), 22.0 (CH_3), 22.6 (CH_3), 48.0 (CH), 50.2 (CH), 73.0 (C), 106.1 (C), 122.7 (CH), 126.8 (CH), 128.0 (CH), 129.0 (CH), 129.7 (C), 133.8 (C), 138.0 (C), 149.8 (C), 157.6 (C), 158.3 (C), 180.9 (C). One *C*H signal overlaps with the C_6D_6 signal at 128.0 ppm in the ^{13}C{^{1}H} NMR spectrum; UV-vis (hexane, 293 K) λ_{max}/nm (ε) 244 (4.0 × 10^4), 277 (sh, 2.0 × 10^4), 312 (sh, 1.2 × 10^4), 428 (7.2 × 10^2); HRMS (FD+eiFi) (*m*/*z*): Calcd for $C_{33}H_{46}N_4$ $[M]^+$ Calc. 498.37225, Found. 498.37210; Elem. Anal. Calcd for [$C_{33}H_{46}N_4$]: C, 79.47%; H, 9.30%; N, 11.23%. Found: C, 79.44%; H, 9.43%; N, 11.24%.

Reaction of BAC with 1-Adamantyl Isocyanide

In a sample tube (5 mL) equipped with a magnetic stir bar, THF (1.0 mL) was added to the mixture of **BAC** (21 mg, 0.087 mmol) and 1-adamantyl isocyanide (14 mg, 0.088 mmol). The mixture was stirred at room temperature for 20.5 hours to give an orange solution. The formation of methyleneketenimine $\mathbf{1_{Ad}}$ (70% yield) was confirmed by NMR spectra analysis. The yield was determined by ^{1}H NMR integrals using 1,3,5-tri-*tert*-butylbenzene as an internal standard.

Isolation of Methyleneketenimine 1_{Ad}

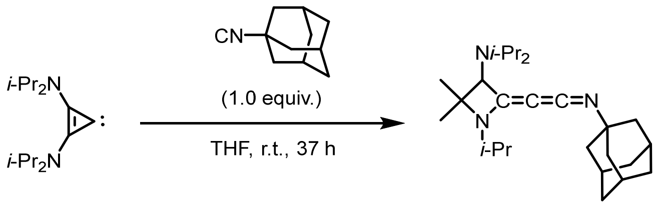

BAC 1_{Ad}

In a J. Young tube (10 mL), **BAC** (205 mg, 0.867 mmol) and 1-adamantyl isocyanide (140 mg, 0.868 mmol) were dissolved in THF (3.0 mL) at room temperature. The pale-yellow solution was stirred at room temperature for 37 hours to give a red solution. After evaporating the volatiles to 0.5 mL, $(SiMe_3)_2O$ (1.0 mL) was slowly added to the solution and placed at -35 °C to give a pale-yellow solid of $\mathbf{1_{Ad}}$ in 65% yield (218 mg, 0.548 mmol).

$\mathbf{1_{Ad}}$: pale yellow solid; mp 97-99 °C; ^{1}H NMR (500 MHz, C_6D_6, 296 K, δ) 0.88 (brd, ^3J(H,H) = 6.5 Hz, 6H, CH_3(*i*-Pr)), 0.96 (s, 3H, CH_3), 1.09 (s, 3H, CH_3), 1.13 (d, ^3J(H,H) = 7.0 Hz, 3H, CH_3(*i*-Pr)), 1.15 (d, ^3J(H,H) = 7.0 Hz, 3H, CH_3(*i*-Pr)), 1.21 (brd, ^3J(H,H) = 6.0 Hz, 6H, CH_3(*i*-Pr)), 1.59-1.67 (m, 6H, Ad), 2.07 (brs, 3H, Ad), 2.14-2.20 (m, 6H, Ad), 3.17 (brs, 2H, CH(*i*-Pr)), 3.31 (sep, ^3J(H,H) = 7.0 Hz, 1H, CH(*i*-Pr)), 3.71 (s, 1H, CH); ^{13}C{^{1}H} NMR (126 MHz, C_6D_6, 298 K, δ) 21.0 (CH_3(*i*-Pr)), 21.6 (CH_3(*i*-Pr)), 22.7 (CH_3), 22.8 (CH_3(*i*-Pr)), 24.4 (CH_3(*i*-Pr)), 25.7 (CH_3), 30.8 (CH(Ad)), 37.1 (CH_2(Ad)), 45.4 (CH_2(Ad)), 47.0 (CH(*i*-Pr)), 57.3 (C(Ad)), 70.6 (CH), 72.0 (C), 87.8 (*C*CCN), 146.0 (*C*CCN), 152.7 (CC*C*N). One *C*H(*i*-Pr) signal could not be determined due to signal overlapping; IR [cm^{-1}]: ῦ (CH_2Cl_2) = 2966, 2912, 2850, 2086 (CCCN), 1655, 1601, 1493, 1419, 1403, 1389, 1365, 1257, 1241, 1203, 1184, 1119, 764, 420, 405, ῦ (toluene) = 3032, 2962, 2927, 2908, 2850, 2090 (CCCN), 1415, 1392, 1365, 1330, 1303, 1242, 1207, 1184, 1157, 1115, 1099, 737, 467, 420; UV-vis (hexane, 293 K) λ_{max}/nm (ε) 214 (2.3 × 10^4), 250 (sh, 5.8 × 10^3), 319 (2.4 × 10^4), 365 (sh, 1.2 × 10^4), (THF, 293 K) λ_{max}/nm (ε) 250 (sh, 6.5 × 10^3), 320 (2.2 × 10^4), 366 (sh, 1.4 × 10^3); HRMS (FD+eiFi) (*m*/*z*): Calcd for $C_{26}H_{43}N_3$ [M]$^+$ 397.34570, Found. 397.34555; Elem. Anal. Calcd for $C_{26}H_{43}N_3$: C, 78.53%; H, 10.90%; N, 10.57%. Found: C, 78.58%; H, 11.04%; N, 10.44%.

Gram Scale Synthesis of Methyleneketenimine 1_{Ad}

In a J. Young tube (50 mL), a THF (14 mL) solution of 1-adamantyl isocyanide (1.1 g, 6.6 mmol) was added dropwise to a THF (11 mL) solution of **DAC** (1.6 g, 6.6 mmol) at room temperature. The red solution was stirred at room temperature for 24 hours to give a red solution. After evaporating the volatiles, the obtained reddish-brown oil was recrystallized from THF:hexane (1:9 solution (10 mL) to afford $\mathbf{1_{Ad}}$ (1.4 g) as a pale-yellow solid. The mother liquid was evaporated and recrystallized from Et_2O two times to a pale-yellow solid (197 mg) of $\mathbf{1_{Ad}}$. Finally, $\mathbf{1_{Ad}}$ was obtained in 65% yield (1.6 g, 4.1 mmol) in total.

Reaction of BAC and 2-Naphthyl Isocyanide (Naph = 2-naphthyl)

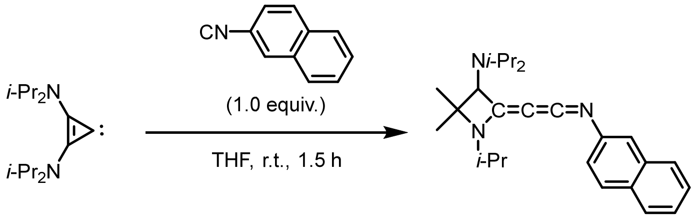

BAC 1_{Naph}

In a sample tube (5 mL) equipped with a magnetic stir bar, THF (1.0 mL) was added to the mixture of **BAC** (21 mg, 0.087 mmol) and 2-naphthyl isocyanide (14 mg, 0.088 mmol). The mixture was stirred at room temperature for 1.5 hours to give a brown solution. The formation of methyleneketenimine $\mathbf{1_{Naph}}$ (54% yield) was confirmed by NMR and HRMS spectra analysis. The yield was determined by ^{1}H NMR integrals using 1,3,5-tri-*tert*-butylbenzene as an internal standard. Compound $\mathbf{1_{Naph}}$ could not be isolated in a pure form due to its oily nature.

$\mathbf{1_{Naph}}$: ^{1}H NMR (500 MHz, C_6D_6, 297 K, δ) 0.84 (brd, ^3J(H,H) = 6.5 Hz, 6H, CH_3(*i*-Pr)), 0.90 (s, 3H, CH_3), 1.02 (s, 3H, CH_3), 1.08 (d, ^3J(H,H) = 7.0 Hz, 3H, CH_3(*i*-Pr)), 1.10 (d, ^3J(H,H) = 6.5 Hz, 3H, CH_3(*i*-Pr)), 1.20 (brd, ^3J(H,H) = 6.5 Hz, 6H, CH_3(*i*-Pr)), 3.10 (brs, 2H, CH(*i*-Pr)), 3.29 (sep, ^3J(H,H) = 6.5 Hz, 1H, CH(*i*-Pr)), 3.73 (s, 1H, CH), 7.18-7.19 (m, 1H, CH(Ar)), 7.25-7.27 (m, 1H, CH(Ar)), 7.62-7.64 (m, 1H, CH(Ar)), 7.67-7.70 (m, 2H, CH(Ar)), 7.88-7.90 (m, 1H, CH(Ar)), 8.06-8.07 (m, 1H, CH(Ar)); ^{13}C{^{1}H} NMR (126 MHz, C_6D_6, 297 K, δ) 20.7 (CH_3), 21.3 (CH_3), 22.3 (CH_3), 22.8 (CH_3), 24.2 (CH_3), 25.1 (CH_3), 47.8 (CH), 70.8 (CH), 74.1 (CMe_2), 80.5 (*C*CCN), 119.4 (CH), 123.8 (CH), 124.2 (CH), 126.0 (CH), 126.4 (CH), 127.6 (CH), 129.2 (CH), 131.5 (C), 135.6 (C), 147.3 (C), 150.1 (C), 152.5 (*C*CCN). One CH(*i*-Pr) signal could not be determined due to signal broadening but it is likely to be at 46.7 ppm. One *C*H(Ar) signal overlaps with the signals of C_6D_6 in the ^{13}C NMR spectrum; HRMS (FD+eiFi) (*m*/*z*): Calcd for $C_{26}H_{35}N_3$ [M]$^+$ 389.28310, Found. 389.28295.

Reaction of BAC and DippNC (Dipp = 2,6-diisopropylphenyl)

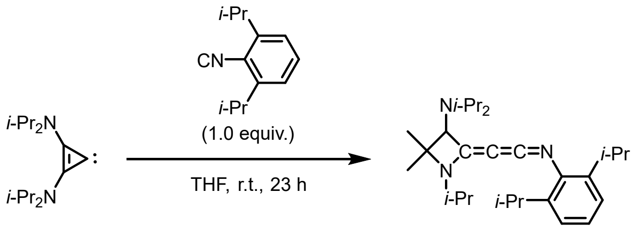

BAC $\mathbf{1_{Dipp}}$

In a sample tube (10 mL) equipped with a magnetic stir bar, a THF solution (3.0 mL) of DippNC (32 mg, 0.17 mmol) was added to **BAC** (41 mg, 0.17 mmol) at room temperature. The mixture was stirred at room temperature for 6 hours to give a dense orange solution. The formation of $\mathbf{1_{Dipp}}$ (83% yield) was confirmed by NMR and HRMS spectra analysis. The yield was determined by ^{1}H NMR integrals using 1,3,5-tri-*tert*-butylbenzene as an internal standard. Crude $\mathbf{1_{Dipp}}$ was obtained as an oil, but could not be purified by silica column chromatography due to its sensitivity to the column and by distillation due to decomposition at 100 °C.

$\mathbf{1_{Dipp}}$: ^{1}H NMR (500 MHz, C_6D_6, 295 K, δ) 0.85 (brd, ^3J(H,H) = 6.5 Hz, 6H, CH_3(*i*-Pr)), 0.90 (s, 3H, CH_3), 1.015 (d, ^3J(H,H) = 7.0 Hz, 3H, CH_3(*i*-Pr)), 1.022 (d, ^3J(H,H) = 6.5 Hz, 3H, CH_3(*i*-Pr)), 1.03 (s, 3H, CH_3) 1.17 (brd, ^3J(H,H) = 3.0 Hz, 6H, CH_3(*i*-Pr)), 1.39 (d, ^3J(H,H) = 6.5 Hz, 6H, CH_3(Ar)), 1.40 (d, ^3J(H,H) = 6.5 Hz, 6H, CH_3(Ar)), 3.13 (brs, 1H, CH(*i*-Pr)), 3.31 (sep, ^3J(H,H) = 7.0 Hz, 1H, CH(*i*-Pr)), 3.73 (s, 1H, CH), 3.99 (sep, ^3J(H,H) = 6.5 Hz, 2H, CH(Ar)), 7.10 (t, 1H, ^3J(H,H) = 7.5 Hz, CH(Ar)), 7.21 (d, 2H, ^3J(H,H) = 7.5 Hz, CH(Ar)); ^{13}C{^{1}H} NMR (126 MHz, C_6D_6, 297 K, δ) 20.8 (CH_3), 21.4 (CH_3), 22.6 (CH_3), 22.7 (CH_3), 24.01 (CH_3), 24.02 (CH_3), 24.5 (CH_3), 25.4 (CH_3), 28.9 (CH), 47.4 (CH), 70.9 (CH), 73.1 (CMe_2), 77.3 (C*C*CN), 123.5 (CH(Ar)), 123.7 (CH(Ar)), 140.9 (C(Ar)), 143.5 (C(Ar)), 149.9 (CC*C*N), 151.7 (*C*CCN). One CH(i-Pr) signal could not be determined due to signal broadening; HRMS (FD+eiFi) (*m*/*z*): Calcd for $C_{28}H_{45}N_3$ $[M]^+$ 423.36135, Found. 423.36125.

Reaction of BAC and TerPhNC (TerPh = 2,6-diphenylphenyl)

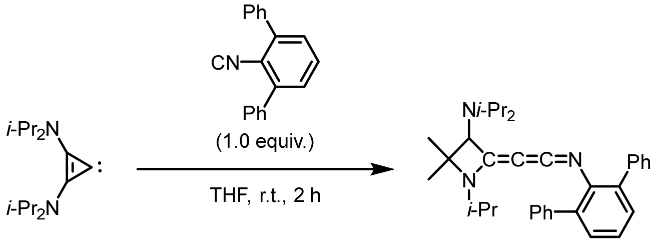

BAC $\mathbf{1_{TerPh}}$

In a sample tube (10 mL) equipped with a magnetic stir bar, THF (0.6 mL) was added to the mixture of terPh-NC (47 mg, 0.19 mmol) and **BAC** (45 mg, 0.19 mmol) at room temperature. The mixture was stirred at room temperature for 2 hours to provide a yellow solution. The formation of $\mathbf{1_{TerPh}}$ (74% yield) was confirmed by NMR and HRMS spectra analysis. The yield was determined by ^{1}H NMR integrals using 1,3,5-tri-*tert*-butylbenzene as an internal standard. Compound $\mathbf{1_{TerPh}}$ could not be isolated in a pure form due to its oily nature.

$\mathbf{1_{TerPh}}$: ^{1}H NMR (500 MHz, C_6D_6, 297 K, δ) 0.80 (s, 3H+6H, CH_3+CH_3(*i*-Pr)), 0.82 (d, ^3J(H,H) = 7.0 Hz, 3H, CH_3(*i*-Pr)), 0.83 (d, ^3J(H,H) = 7.0 Hz, 3H, CH_3(*i*-Pr)), 0.93 (s, 3H, CH_3), 0.98 (brs, 3H, CH_3(*i*-Pr)), 2.93 (brs, 2H, CH(*i*-Pr)), 3.05 (sep, ^3J(H,H) = 7.0 Hz, 1H, CH(*i*-Pr)), 3.42 (s, 1H, CH), 7.01 (t, 1H, ^3J(H,H) = 7.0 Hz, CH(Ar)), 7.11 (t, 2H, ^3J(H,H) = 7.5 Hz, CH(Ar)), 7.25 (t, ^3J(H,H) = 8.0 Hz, 4H(Ar)), 7.35 (d, ^3J(H,H) = 7.5 Hz, 2H(Ar)), 7.77-7.89 (m, 4H, CH(Ar)); ^{13}C{^{1}H} NMR (126 MHz, C_6D_6, 297 K, δ) 20.7 (CH_3), 21.5 (CH_3), 22.67 (CH_3), 22.74 (CH_3), 24.3 (CH_3), 25.3 (CH_3), 47.4 (CH), 70.5 (CH), 73.2 (CMe_2), 77.9 (C*C*CN), 123.2 (CH(Ar)), 126.4 (CH(Ar)), 128.1(CH(Ar)), 130.6 (CH(Ar)), 130.7 (CH(Ar)), 136.3 (C(Ar)), 142.2 (C(Ar)), 144.1 (C(Ar)), 150.5 (CC*C*N), 151.8 (*C*CCN). One CH(i-Pr) signal could not be determined due to signal broadening; HRMS (FD+eiFi) (*m*/*z*): Calcd for $C_{34}H_{41}N_3$ $[M]^+$ 491.33005, Found. 491.33004.

Reaction of BAC with 1,4-Bis(isocyano)benzene (0.5 eq)

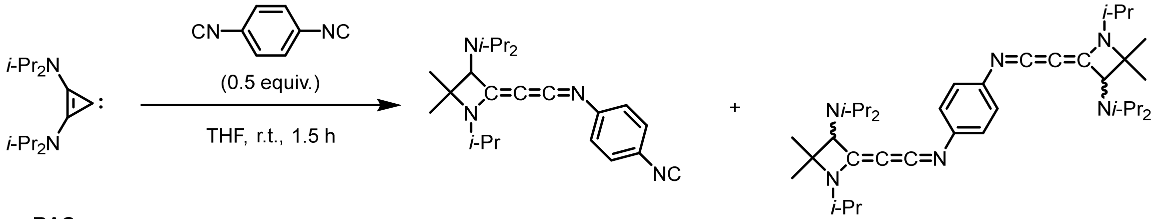

BAC **1_{PhNC}** 9%* **4** 40%*

In a sample tube (5 mL) with a magnetic stir bar, THF (1.0 mL) was added to the mixture of **BAC** (41 mg, 0.17 mmol) and 1,4-bis(isocyano)benzene (11 mg, 0.088 mmol). The mixture was stirred at room temperature for 1.5 hours to give a brown solution. The formation of **1_{PhNC}** (9% yield) and **4** (40% yield) was confirmed by NMR spectra analysis. The yields were determined by ^{1}H NMR integrals using 1,3,5-tri-*tert*-butylbenzene as an internal standard.

Reaction of BAC with 1,4-Bis(isocyano)benzene (1.0 eq)

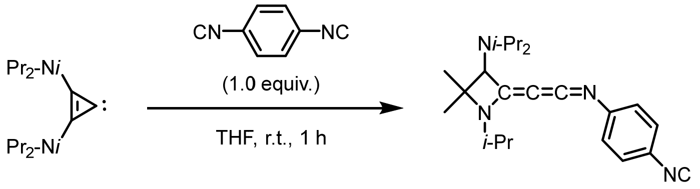

BAC **1_{PhNC}**

In a sample tube (10 mL) equipped with a magnetic stir bar, a THF (1.0 mL) solution of **BAC** (38 mg, 0.16 mmol) was added dropwise to a THF (1.0 mL) solution of 1,4-bis(isocyano)benzene (21 mg, 0.16 mmol). The mixture was stirred at room temperature for an hour to give a red solution. The formation of **1_{PhNC}** (54% yield) was confirmed by NMR and HRMS spectra analysis. The yield was determined by ^{1}H NMR integrals using 1,3,5-tri-*tert*-butylbenzene as an internal standard. Compound **1_{PhNC}** could not be isolated in a pure form due to the presence of other byproducts and **1_{PhNC}**'s oily nature.

1_{PhNC}: ^{1}H NMR (500 MHz, C_6D_6, 293 K, δ) 0.81 (brd, 6H, ^3J(H,H) = 6.5 Hz, CH_3(*i*-Pr)), 0.85 (s, 3H, CH_3), 0.99 (s, 3H, CH_3), 1.01 (d, 3H, ^3J(H,H) = 7.5 Hz, CH_3(*i*-Pr)), 1.03 (d, 3H, ^3J(H,H) = 7.5 Hz, CH_3(*i*-Pr)), 1.10 (d, 6H, ^3J(H,H) = 7.5 Hz, CH_3(*i*-Pr)), 2.98-3.06 (m, 2H, CH(*i*-Pr)), 3.23 (sep, ^3J(H,H) = 6.5 Hz, 1H, CH(*i*-Pr)), 3.66 (s, 1H, CH), 6.89 (d, 2H, ^3J(H,H) = 8.5 Hz, CH(Ar)), 7.28 (d, 2H, ^3J(H,H) = 8.5 Hz, CH(Ar)); ^{13}C{^{1}H} NMR (126 MHz, C_6D_6, 297 K, δ) 20.6 (CH_3), 21.2 (CH_3), 22.0 (CH_3), 22.7 (CH_3), 24.3 (CH_3), 24.9 (CH_3), 48.0 (CH), 70.8 (CMe_2), 74.9 (CH), 78.0 (C*C*CN), 121.3 (C), 123.2 (CH(Ar)), 127.5 (CH(Ar)), 148.0 (NC), 150.6 (C(Ar)), 154.6 (*C*CCN). One CH(*i*-Pr) signal could not be determined due to signal broadening. The CC*C*N signal could not be determined; HRMS (FD+eiFi) (*m*/*z*): Calcd for $C_{23}H_{32}N_4$ $[M]^+$ 364.26270, Found. 364.26206.

Isolation of Bis(methyleneketenimine) 4

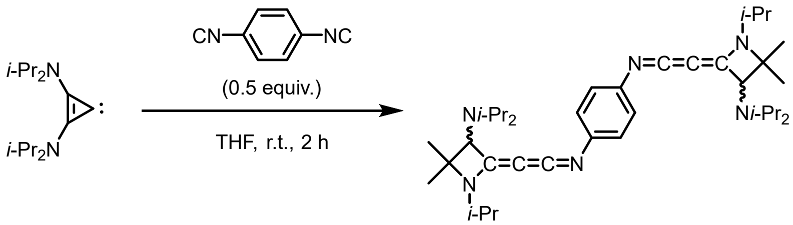

BAC **4**

In a sample tube (10 mL) equipped with a magnetic stir bar, THF (0.8 mL) was added to a mixture of **BAC** (62 mg, 0.26 mmol) and 1,4-bis(isocyano)benzene (15 mg, 0.12 mmol), and the solution was stirred for 2 hours. After removing the volatiles in vacuo, the brown oil was obtained. Recrystallization from $(SiMe_3)_2O$ (0.5 mL) at –35 °C afforded a yellow solid of **4** in 17% yield (13 mg, 0.021 mmol).

4: yellow solid; mp 80°C (decomp.); ^{1}H NMR (500 MHz, C_6D_6, 299 K, δ) 0.84 (brd, ^3J(H,H) = 6.5 Hz, 12H, CH_3(*i*-Pr)), 0.90 (s, 6H, CH_3), 1.03 (s, 6H, CH_3), 1.05 (d, ^3J(H,H) = 7.0 Hz, 6H, CH_3(*i*-Pr)), 1.07 (d, ^3J(H,H) = 7.0 Hz, 6H, CH_3(*i*-Pr)), 1.18 (brd, ^3J(H,H) = 5.5 Hz, 12H, CH_3(*i*-Pr)), 3.11 (brs, 4H, CH(*i*-Pr)), 3.29 (sep, ^3J(H,H) = 6.8 Hz, 2H, CH(*i*-Pr)), 3.71 (s, 2H, CH), 7.75 (s, 4H, CH(Ar)); ^{13}C{^{1}H} NMR (126 MHz, C_6D_6, 300 K, δ) 20.7 (CH_3), 21.3 (CH_3), 22.5 (CH_3), 22.8 (CH_3), 24.4 (CH_3), 25.3 (CH_3), 47.5 (CH), 70.7 (CH), 73.5 (CMe_2), 82.4 (CCCN), 123.8 (CH(Ar)), 144.8 (C(Ar)), 150.0 (CC*C*N), 150.4 (*C*CCN). One *C*H(*i*-Pr) signal could not be observed in the ^{13}C NMR spectrum, possibly due to signal broadening; UV-vis (THF, 293 K) λ_{max}/nm (*ε*) 240 (sh, 1.8 × 10^4), 276 (sh, 7.1 × 10^3), 330 (1.5× 10^4), 373 (sh, 1.9 × 10^4), 440

(4.8× 10^4), 467 (sh, 3.9 × 10^4), 500 (sh, 4.1 × 10^3);HRMS (FD+eiFi) (*m/z*): Calcd for $C_{38}H_{60}N_6$ $[M]^+$ 600.48794 Found. 600.48782; Elem. Anal. Calcd for $C_{38}H_{60}N_6$: C, 75.95%; H, 10.06%; N, 13.99%. Found: C, 74.56%; H, 9.83%; N, 13.99%. Numerous attempts of CHN analysis failed due to **4**`s thermal instability.

Reaction of Methyleneketenimine $\mathbf{1_{Ad}}$ with AuCl(THT)

In a sample tube (10 mL) equipped with a magnetic stir bar, toluene (1.0 mL) was added to a mixture of methyleneketenimine $\mathbf{1_{Ad}}$ (51 mg, 0.13 mmol) and AuCl(THT) (42 mg, 0.13 mmol). After stirring for 2 min, the formation of **5** (92% yield) was confirmed by NMR spectra analysis. The yield was determined by ^{1}H NMR integrals using 1,3,5-tri-*tert*-butylbenzene as an internal standard.

Isolation of Methyleneketenimine AuCl complex 5

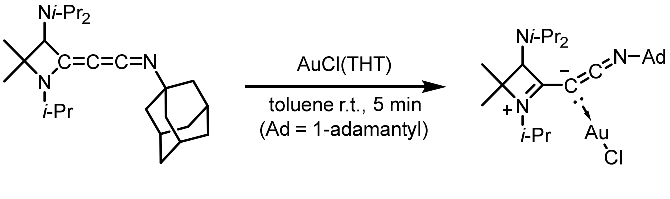

$\mathbf{1_{Ad}}$ **5**

In a sample tube (10 mL) equipped with a magnetic stir bar, toluene (1.2 mL) was added to the mixture of methyleneketenimine $\mathbf{1_{Ad}}$ (52 mg, 0.130 mmol) and AuCl(THT) (41 mg, 0.127 mmol). After stirring for 5 min, the solution was filtered. The volatiles were removed from the filtrate in vacuo, and the resulting solid was washed with hexane (0.8 mL) twice, which afforded pale yellow crystals of **5** in 80% yield (64 mg, 0.102 mmol).

5: pale yellow crystals; mp 72°C (decomp.); ^{1}H NMR (500 MHz, THF-*d₈*, 292 K, δ) 0.98 (brd, 3H, ^3J(H,H) = 5.0 Hz, CH_3(*i*-Pr)), 1.16 (brd, 3H, ^3J(H,H) = 5.5 Hz, CH_3(*i*-Pr)), 1.20 (brs, 6H, CH_3(*i*-Pr)), 1.34 (d, ^3J(H,H) = 7.0 Hz, 3H, CH_3(*i*-Pr)), 1.36 (d, ^3J(H,H) = 7.0 Hz, 3H, CH_3(*i*-Pr)), 1.48 (s, 3H+3H, CH_3+CH_3), 1.70-1.71 (m, 6H, Ad), 1.95-2.05 (m, 6H, Ad), 2.08-2.14 (m, 3H, Ad), 3.15-3.25 (m, 1H, CH(*i*-Pr)), 3.26-3.36 (m, 1H, CH(*i*-Pr)), 4.12 (s, 1H, CH), 5.45 (sep, ^3J(H,H) = 7.0 Hz, 1H, CH(*i*-Pr)); ^{1}H NMR (500 MHz, THF-d_8, 273 K, δ) 0.98 (d, 3H, ^3J(H,H) = 6.5 Hz, CH_3(*i*-Pr)), 1.15 (d, 3H, ^3J(H,H) = 6.5 Hz, CH_3(*i*-Pr)), 1.18 (d, 3H, ^3J(H,H) = 6.5 Hz, CH_3(*i*-Pr)), 1.20 (d, 3H, ^3J(H,H) = 6.0 Hz, CH_3(*i*-Pr)), 1.33 (d, ^3J(H,H) = 7.0 Hz, 3H, CH_3(*i*-Pr)), 1.36 (d, ^3J(H,H) = 7.0 Hz, 3H, CH_3(*i*-Pr)), 1.476 (s, 3H, CH_3), 1.481 (s, 3H, CH_3), 1.70-1.71 (m, 6H, Ad), 1.97-1.99 (m, 6H, Ad), 2.08-2.13 (m, 3H, Ad), 3.19 (sep, ^3J(H,H) = 7.0 Hz, 1H, CH(*i*-Pr)), 3.31 (sep, ^3J(H,H) = 7.0 Hz, 1H, CH(*i*-Pr)), 4.15 (s, 1H, CH), 5.47 (sep, ^3J(H,H) = 7.0 Hz, 1H, CH(*i*-Pr)); ^{1}H NMR (500 MHz, THF-d_8, 253 K, δ) 0.97 (d, 3H, ^3J(H,H) = 6.5 Hz, CH_3(*i*-Pr)), 1.15 (d, 3H, ^3J(H,H) = 6.5 Hz, CH_3(*i*-Pr)), 1.18 (d, 3H, ^3J(H,H) = 7.0 Hz, CH_3(*i*-Pr)), 1.19 (d, 3H, ^3J(H,H) = 6.5 Hz, CH_3(*i*-Pr)), 1.32 (d, ^3J(H,H) = 7.0 Hz, 3H, CH_3(*i*-Pr)), 1.36 (d, ^3J(H,H) = 7.0 Hz, 3H, CH_3(*i*-Pr)), 1.48 (s, 3H, CH_3), 1.49 (s, 3H, CH_3), 1.69-1.70 (m, 6H, Ad), 1.97-2.01 (m, 6H, Ad), 2.08-2.13 (m, 3H, Ad), 3.18 (sep, ^3J(H,H) = 6.5 Hz, 1H, CH(*i*-Pr)), 3.31 (sep, ^3J(H,H) = 6.5 Hz, 1H, CH(*i*-Pr)), 4.18 (s, 1H, CH), 5.48 (sep, ^3J(H,H) = 7.0 Hz, 1H, CH(*i*-Pr)); $^{13}C\{^1H\}$ NMR (126 MHz, THF-d_8, 253 K, δ) 20.2 (CH_3), 21.9 (CH_3), 22.96 (CH_3), 23.04 (CH_3), 23.9 (CH_3), 25.7 (CH_3), 25.8 (CH_3), 30.7 (CH(Ad)), 36.3 (CH_2(Ad)), 43.6 (CH), 44.0 (CH_2(Ad)), 50.0 (CH), 50.7 (CH), 53.0 (CAu), 61.9 (C(Ad)), 69.0 (CH), 79.6 (CMe_2), 151.5 (C*C*CN), 183.7 (*C*CCN). One *C*H_3(*i*-Pr) signal is overlapped with signals either at 22.96 and 23.04 ppm based on ^{1}H-^{13}C HSQC spectra (Figure S76). NMR data were collected at low temperatures because the Au complex **5** gradually decomposes at room temperature; HRMS (FD+eiFi): Calcd for $C_{26}H_{43}AuClN_3$ $[M]^+$ 629.28110, Found. 629.28101; Elem. Anal. Calcd for $C_{26}H_{43}AuClN_3$: C, 49.56%; H, 6.88%; N, 6.67%. Found: C, 48.56%; H, 6.72%; N, 6.39%. Numerous attempts of CHN analysis failed due to **5**`s thermal instability.

Reaction of Methyleneketenimine $\mathbf{1_{Ad}}$ with $[Rh(CO)_2Cl]_2$

In a sample tube (10 mL) equipped with a magnetic stir bar, $[Rh(CO)_2Cl]_2$ (29 mg, 0.075 mmol) was added to a benzene (2.0 mL) solution of methyleneketenimine $\mathbf{1_{Ad}}$ (60 mg, 0.15 mmol) for 10 min in the dark to give an orange solution. The formation of **6** (96% yield) was confirmed by NMR spectra. The yield was determined by ^{1}H NMR integrals using 1,3,5-tri-*tert*-butylbenzene as an internal standard.

Isolation of Methyleneketenimine $Rh(CO)_2Cl$ Complex 6

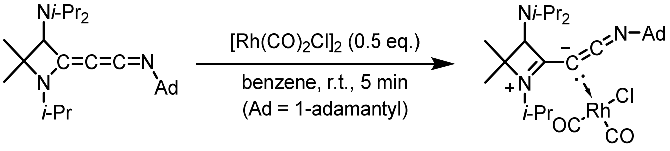

$\mathbf{1_{Ad}}$ **6**

In a sample tube (10 mL) equipped with a magnetic stir bar, $[Rh(CO)_2Cl]_2$ (14 mg, 0.037 mmol) was added to a benzene (1.0 mL) solution

of methyleneketenimine $\mathbf{1_{Ad}}$ (30 mg, 0.074 mmol) in the dark and stirred for 5 min. to give an orange solution. The volatiles were removed from the filtrate in vacuo, and the resulting orange solid was washed with hexane (0.5 mL) two times, which afforded orange crystals of **6** in 59% yield (26 mg, 0.043 mmol).

6: orange crystals; mp 80 °C (decomp.); ^{1}H NMR (500 MHz, THF-*d*$_8$, 293 K, δ) 0.97 (brd, 3H, ^{3}J(H,H) = 5.0 Hz, CH_3(*i*-Pr)), 1.13 (brs, 3H, CH_3(*i*-Pr)), 1.19 (d, ^{3}J(H,H) = 6.0 Hz, 3H+3H, CH_3(*i*-Pr)+CH_3(*i*-Pr)), 1.31 (d, ^{3}J(H,H) = 7.0 Hz, 3H, CH_3(*i*-Pr)), 1.34 (d, ^{3}J(H,H) = 7.0 Hz, 3H, CH_3(*i*-Pr)), 1.469 (s, 3H, CH_3), 1.472 (s, 3H, CH_3), 1.65-1.70 (m, 6H, Ad), 2.00-2.09 (m, 9H, Ad), 3.31 (brs, 1H, CH(*i*-Pr)), 3.43 (brs, 1H, CH(*i*-Pr)), 4.08 (s, 1H, CH), 5.80 (sep, ^{3}J(H,H) = 7.0 Hz, 1H, CH(*i*-Pr)); ^{1}H NMR (500 MHz, THF-*d*$_8$, 273 K, δ) 0.97 (d, 3H, ^{3}J(H,H) = 7.0 Hz, CH_3(*i*-Pr)), 1.13 (d, 3H, ^{3}J(H,H) = 6.0 Hz, CH_3(*i*-Pr)), 1.18 (d, 3H, ^{3}J(H,H) = 5.5 Hz, CH_3(*i*-Pr)), 1.19 (d, 3H, ^{3}J(H,H) = 5.0 Hz, CH_3(*i*-Pr)), 1.31 (d, ^{3}J(H,H) = 6.5 Hz, 3H, CH_3(*i*-Pr)), 1.34 (d, ^{3}J(H,H) = 7.0 Hz, 3H, CH_3(*i*-Pr)), 1.47 (s, 3H+3H, CH_3+CH_3), 1.66-1.71 (m, 6H, Ad), 2.00-2.09 (m, 9H, Ad), 3.30 (sep, ^{3}J(H,H) = 6.5 Hz, 1H, CH(*i*-Pr)), 3.43 (brs, 1H, CH(*i*-Pr)), 4.10 (s, 1H, CH), 5.81 (sep, ^{3}J(H,H) = 7.0 Hz, 1H, CH(*i*-Pr)); ^{1}H NMR (500 MHz, THF-*d*$_8$, 254 K, δ) 0.97 (d, 3H, ^{3}J(H,H) = 6.5 Hz, CH_3(*i*-Pr)), 1.12 (d, 3H, ^{3}J(H,H) = 6.5 Hz, CH_3(*i*-Pr)), 1.17-1.19 (m, 3H+3H, CH_3(*i*-Pr)+CH_3(*i*-Pr)), 1.31 (d, ^{3}J(H,H) = 7.0 Hz, 3H, CH_3(*i*-Pr)), 1.34 (d, ^{3}J(H,H) = 7.0 Hz, 3H, CH_3(*i*-Pr)), 1.47 (s, 3H+3H, CH_3+CH_3), 1.65-1.71 (m, 6H, Ad), 1.99-2.08 (m, 9H, Ad), 3.30 (sep, ^{3}J(H,H) = 6.5 Hz, 1H, CH(*i*-Pr)), 3.43 (brs, 1H, CH(*i*-Pr)), 4.13 (s, 1H, CH), 5.81 (sep, ^{3}J(H,H) = 7.0 Hz, 1H, CH(*i*-Pr)); ^{13}C{^{1}H} NMR (126 MHz, THF-*d*$_8$, 253 K, δ) 20.0 (CH_3), 22.0 (CH_3), 22.8 (CH_3), 23.0 (CH_3), 23.9 (CH_3), 25.7 (CH_3), 25.8 (CH_3), 30.7 (CH(Ad)), 36.4 (CH_2(Ad)), 43.7 (CH), 44.1 (CH_2(Ad)), 50.7 (CH), 50.9 (CH), 51.9 (CRh, d, ^{1}J(Rh, C) = 25 Hz), 68.5 (CH), 79.0 (C), 186.2 (CO, d, ^{1}J(Rh, C) = 60 Hz), 187 (*C*CCN)*. NMR data were collected at low temperatures because the Rh complex **6** gradually decomposes at room temperature. One *C*H_3(*i*-Pr) signal is overlapped with signals either at 22.8 and 23.0 ppm based on ^{1}H-^{13}C HSQC spectra (Figure S88). One *C*O, *C*CCN, and CC*C*N could not be observed possibly due to signal broadening and coupling to ^{103}Rh. *The *C*CCN signal could not directly be observed from ^{13}C{^{1}H} NMR spectrum but was observed in the ^{1}H-^{13}C HMBC NMR spectrum; IR [cm^{-1}]: ῦ (CH_2Cl_2) = 2966, 2935, 2918, 2856, 2063 (CO), 2031(CCN), 2021, 1984 (CO), 1574, 1369, 1365, 1304, 1244, 1186, 1103, 1055, 594, 422, 413, 407, ṽ (toluene) = 2966, 2931, 2912, 2854, 2060 (CO), 2033 (CCN), 1979 (CO), 1574, 1462, 1408, 1392, 1365, 1304, 1242, 1184, 1115, 1103, 1057, 594, 463; HRMS (FD+eiFi): Calcd for $C_{28}H_{43}ClN_3O_2Rh$ $[M]^+$ 591.20988, Found. 591.20969; Elem. Anal. Calcd for $C_{28}H_{43}ClN_3O_2Rh$: C, 56.81%; H, 7.32%; N, 7.10%. Found: C, 56.42%; H, 7.33%; N, 7.06%.

Reaction of Methyleneketenimine $\mathbf{1_{Xyl}}$ with $\mathbf{CO_2}$

In a J. Young NMR tube, C_6D_6 (0.55 mL) was added to a mixture of **BAC** (38 mg, 0.16 mmol) and xylyl isocyanide (21 mg, 0.16 mmol), and the tube was placed at room temperature for 5 hours. The formation of $\mathbf{1_{Xyl}}$ (79% yield) was confirmed by NMR spectra. After freeze-pump-thaw, CO_2 gas (1 atm) was introduced to the tube and the resulting red solution was placed at room temperature for 17 hours, and the formation of $\mathbf{7_{Xyl}}$ (77% yield) was confirmed by NMR spectra analysis. The yield was determined by ^{1}H NMR integrals using 1,3,5-tri-*tert*-butylbenzene as an internal standard.

Isolation of Azetidine-2,4-dione $\mathbf{7_{Xyl}}$

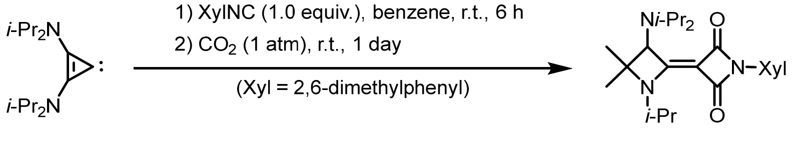

BAC $\mathbf{7_{Xyl}}$

In a J. Young tube (50 mL) equipped with a magnetic stir bar, benzene (1.5 mL) was added to a mixture of **BAC** (81 mg, 0.35 mmol) and xylyl isocyanide (45 mg, 0.34 mmol) and stirred at room temperature for 6 hours. After three freeze-pump-thaw cycles, CO_2 gas (1 atm) was introduced to the tube and stirred for 5 minutes. After sealing the tube, the solution was stirred for 1 day. The volatiles were removed in vacuo to afford a brown oil. Recrystallization from hexane (0.5 mL×2 times) at -23 °C gave a pale-yellow solid. The solid was purified by subjecting the crude mixture to silica gel column chromatography (eluent: CH_2Cl_2). The silica gel was deactivated with CH_2Cl_2 containing 1% NEt_3. After the volatiles were removed in vacuo, colorless crystals of $\mathbf{7_{Xyl}}$ were obtained in 59% yield (83 mg, 0.20 mmol).

$\mathbf{7_{Xyl}}$: colorless crystals; mp 122 °C (decomp.); ^{1}H NMR (500 MHz, C_6D_6, 295 K, δ) 0.74 (brs, 3H, CH_3(*i*-Pr)), 0.88 (s, 3H, CH_3), 1.00 (d+brs, ^{3}J(H,H) = 7.0 Hz, 3H+3H, CH_3(*i*-Pr)+CH_3(*i*-Pr)), 1.05 (s, 3H, CH_3), 1.15 (d, ^{3}J(H,H) = 6.5 Hz, 3H, CH_3(*i*-Pr)), 1.32 (brs, 3H, CH_3(*i*-Pr)), 1.42 (brs, 3H, CH_3(*i*-Pr)), 2.418 (s, 3H, CH_3(Ar)), 2.424 (s, 3H, CH_3(Ar)), 3.07 (brs, 1H, CH(*i*-Pr)), 3.20 (brs, 1H, CH(*i*-Pr)), 3.90 (s, 1H, CH), 4.39 (sep, ^{3}J(H,H) = 7.0 Hz, 1H, CH(*i*-Pr)), 6.87-6.89 (m, 2H, (CH)Ar), 6.92-6.95 (m, 1H, (CH)Ar); ^{13}C{^{1}H} NMR (126 MHz, C_6D_6, 296 K, δ) 19.2 (CH_3(Ar)), 20.5 (CH_3(*i*-Pr)), 21.4 (CH_3(*i*-Pr)), 22.1 (CH_3(*i*-Pr)), 22.2 (CH_3(*i*-Pr)), 23.3 (CH_3), 24.0 (CH_3 (*i*-Pr)), 26.2 (CH_3), 26.4 (CH_3(*i*-Pr)), 43.4 (CH(*i*-Pr)), 50.2 (CH(*i*-Pr)+CH(*i*-Pr)*), 65.1 (CH), 76.8 (CMe_2), 94.2 (C=*C*), 128.1 (CH), 128.56 (CH), 128.58 (CH), 136.8 (C), 136.9 (C), 156.1 (*C*=C), 168.2 (CO), 168.3 (CO). *Two CH(*i*-Pr) signals are overlapped; HRMS (FD+eiFi): Calcd for $C_{25}H_{37}N_3O_2$ $[M]^+$ 411.28858, Found. 411.28834; Elem. Anal. Calcd for $C_{25}H_{37}N_3O_2$: C, 72.95%; H, 9.06%; N, 10.21%. Found: C, 72.80%; H, 9.01%; N, 10.11%.

Reaction of Isolated Methyleneketenimine 1_{Xyl} with CO_2

In a J. Young NMR tube, C_6D_6 (0.50 mL) was added to methyleneketenimine $\mathbf{1_{Xyl}}$. After three freeze-pump-thaw cycles, CO_2 gas (1 atm) was introduced to the tube and the resulting yellow solution was placed at room temperature for 22.5 hours, and the formation of $\mathbf{7_{Xyl}}$ (96% yield) was confirmed by NMR spectra analysis. The yield was determined by ^{1}H NMR integrals using 1,3,5-tri-*tert*-butylbenzene as an internal standard.

Reaction of Methyleneketenimine 1_{Ad} with CO_2

In a J. Young NMR tube, C_6D_6 (0.55 mL) was added to methyleneketenimine $\mathbf{1_{Ad}}$ (56 mg, 0.15 mmol). After three freeze-pump-thaw cycles, CO_2 gas (1 atm) was introduced to the tube and the resulting red solution was stirred at room temperature for 22 hours, and the formation of $\mathbf{7_{Ad}}$ (32% yield) was confirmed by NMR spectra analysis. The yield was determined by ^{1}H NMR integrals using 1,3,5-tri-*tert*-butylbenzene as an internal standard.

Isolation of Azetidine-2,4-dione 7_{Ad}

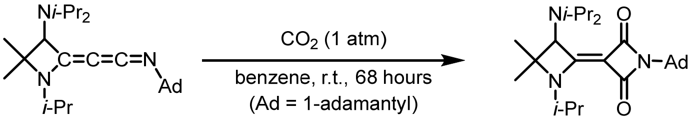

$\mathbf{1_{Ad}}$ $\mathbf{7_{Ad}}$

In a J. Young tube (50 mL), methyleneketenimine $\mathbf{1_{Ad}}$ (105.0 mg, 0.264 mmol) was dissolved in benzene (1.8 mL) at room temperature. After two freeze-pump-thaw cycles, CO_2 gas (1 atm) was introduced to the tube and stirred for 10 minutes. After sealing the tube, the yellowish orange solution was stirred for 68 hours, and the color of the solution changed to dark red. The volatiles were removed in vacuo to afford a brown solid (0.148 mg). The solid was purified by subjecting the crude mixture to silica gel column chromatography (eluent: CH_2Cl_2). The silica gel was deactivated with CH_2Cl_2 containing 2% NEt_3. After the volatiles were removed in vacuo, a pale orange solid (31.9 mg) was obtained. Recrystallization of the solid from hexane (1.0 mL) at –30 °C gave colorless crystals of $\mathbf{7_{Ad}}$ in 16% yield (19 mg, 0.043 mmol). The yield of $\mathbf{7_{Ad}}$ was low due to the competing hydrolysis of $\mathbf{1_{Ad}}$ during the reaction.

$\mathbf{7_{Ad}}$: colorless crystals; mp 140 °C (decomp.); ^{1}H NMR (500 MHz, C_6D_6, 295 K, δ) 0.75 (brs, 3H, CH_3(*i*-Pr)), 0.88 (s, 3H, CH_3), 1.00 (d+brs, ^3J(H,H) = 6.5 Hz, 3H+3H, CH_3(*i*-Pr)+CH_3(*i*-Pr)), 1.04 (s, 3H, CH_3), 1.14 (d, ^3J(H,H) = 7.0 Hz, 3H, CH_3(*i*-Pr)), 1.32-1.55 (m, 6H+3H+3H, Ad+CH_3(*i*-Pr)+ CH_3(*i*-Pr)), 1.88 (brs, 3H, Ad), 2.43-2.44 (m, 6H, Ad), 3.08 (brs, 1H, CH(*i*-Pr)), 3.17 (brs, 1H, CH(CH_3)), 3.83 (s, 1H, CH), 4.32 (sep., ^3J(H,H) = 7.0 Hz, 1H, CH(*i*-Pr)); $^{13}C\{^1H\}$ NMR (126 MHz, C_6D_6, 297 K, δ) 20.7 (CH_3(*i*-Pr)), 21.6 (CH_3(*i*-Pr)), 22.0 (CH_3(*i*-Pr)), 22.4 (CH_3(*i*-Pr)), 23.3 (CH_3(*i*-Pr)), 24.0 (CH_3), 26.3 (CH_3+CH_3(*i*-Pr)), 29.7 (CH (Ad)), 36.6 (CH_2 (Ad)), 41.5 (CH_2 (Ad)), 43.4 (CH(*i*-Pr)), 49.9 (CH(*i*-Pr)), 50.1 (CH(*i*-Pr)), 56.4 (C(Ad)), 65.0 (CH), 75.9 (C), 96.1 (C=*C*), 153.9 (*C*=C), 169.9 (CO), 170.1 (CO); IR [cm^{-1}]: ῦ (KBr) = 2962, 2937, 2912, 2891, 2852, 1836, 1707, 1666, 1466, 1417, 1365, 1329, 1306, 1246, 1186, 1122, 1095, 1047, 1026, 1007, 987, 939, 893, 837, 812, 789, 673, 579, 555, 474; UV-vis (hexane, 293 K) λ_{max}/nm (*ε*) 289 (2.3× 10^4); HRMS (APCI-positive) (*m*/*z*): Calcd for $C_{27}H_{43}N_3O_2$ [M]$^+$ 441.33498, Found. 441.33501; Elem. Anal. Calcd for $C_{27}H_{43}N_3O_2$: C, 73.43%; H, 9.81%; N, 9.51%. Found: C, 73.43%; H, 9.90%; N, 9.41%.

Reaction of Methyleneketenimine 1_{Naph} with CO_2

In a J. Young NMR tube, C_6D_6 (0.60 mL) was added to a mixture of **BAC** (32 mg, 0.14 mmol) and 2-naphthyl isocyanide (21 mg, 0.13 mmol) and placed at room temperature for 3 hours. The formation of methyleneketenimine $\mathbf{1_{Naph}}$ (60% yield) was confirmed by NMR spectra analysis. After freeze-pump-thaw, CO_2 gas (1 atm) was introduced to the tube and the resulting reddish-brown solution was placed at room temperature for 15.5 hours. The formation of azetidine-2,4-dione $\mathbf{7_{Naph}}$ (59% yield) was confirmed by NMR spectra analysis. The yield was determined by ^{1}H NMR integrals using 1,3,5-tri-*tert*-butylbenzene as an internal standard.

Isolation of Azetidine-2,4-dione 7_{Naph}

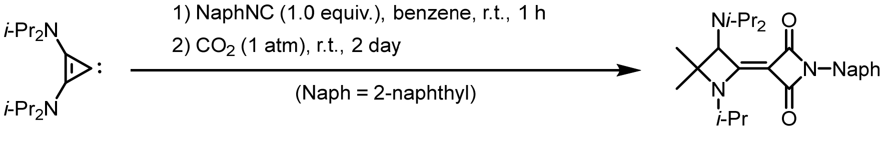

BAC $\mathbf{7_{Naph}}$

In a J. Young tube (50 mL) equipped with a magnetic stir bar, benzene (1.5 mL) was added to a mixture of **BAC** (67 mg, 0.28 mmol) and 2-naphthyl isocyanide (43 mg, 0.28 mmol) and stirred at room temperature for 1 hour. After two cycles of freeze-pump-thaw, CO_2 gas (1

atm) was introduced to the tube and stirred for 5 minutes. After sealing the tube, the solution was stirred for 2 days. The volatiles were removed in vacuo to afford a reddish black oil. Recrystallization from hexane (0.5 mL) at -23 °C gave a brown solid. The brown solid was purified by subjecting to silica gel column chromatography (eluent: toluene). The silica gel was deactivated with toluene containing 6% NEt_3. After the volatiles were removed in vacuo, colorless crystals of $\mathbf{7}_{\mathbf{Naph}}$ in 47% yield (58 mg, 0.13 mmol) were obtained.

$\mathbf{7}_{\mathbf{Naph}}$: colorless crystals; mp 140-142 °C; ^{1}H NMR (500 MHz, C_6D_6, 297 K, δ) 0.75 (brs, 3H, CH_3(*i*-Pr)), 0.87 (s, 3H, CH_3), 0.97 (d+brs, ^3J(H,H) = 7.0 Hz, 3H+3H, CH_3(*i*-Pr)+CH_3(*i*-Pr)), 1.02 (s, 3H, CH_3), 1.08 (d, ^3J(H,H) = 7.0 Hz, 3H, CH_3(*i*-Pr)), 1.25 (brs, 3H, CH_3(*i*-Pr)), 1.46 (brs, 3H, CH_3(*i*-Pr)), 3.07 (sep, ^3J(H,H) = 6.5 Hz, 2H, CH(*i*-Pr)), 3.83 (s., 1H, CH), 4.31 (sep, ^3J(H,H) = 7.0 Hz, 1H, CH(*i*-Pr)), 7.12-7.14 (m, 2H CH(Ar)), 7.49-7.51 (m, 1H, CH(Ar)), 7.55-7.58 (m, 2H, CH(Ar)), 8.45-8.52 (m, 1H, CH(Ar)), 8.70 (brs, 1H, CH(Ar)); ^{13}C{^{1}H} NMR (126 MHz, C_6D_6, 298 K, δ) 20.6 (CH_3(*i*-Pr)), 21.6 (CH_3(*i*-Pr)), 22.0 (CH_3(*i*-Pr)), 22.4 (CH_3(*i*-Pr)), 23.3 (CH_3(*i*-Pr)), 23.8 (CH_3), 26.1 (CH_3), 26.3 (CH_3(*i*-Pr)), 43.4 (CH(*i*-Pr)), 50.4 (CH(*i*-Pr)), 65.3 (CH), 77.1 (C), 95.9 (C=*C*), 113.7 (CH), 117.5 (CH), 124.9 (CH), 126.8 (CH), 128.1 (CH), 128.2 (CH) 129.5 (CH), 130.8 (C), 134.7 (C), 136.2 (C), 157.3 (*C*=C), 167.4 (CO), 167.5 (CO); HRMS (FD+eiFi): Calcd for $C_{27}H_{35}N_3O_2$ $[M]^+$ 433.27293, Found. 433.27279; Elem. Anal. Calcd for $C_{27}H_{35}N_3O_2$: C, 74.79%; H, 8.14%; N, 9.69%. Found: C, 74.76%; H, 8.13%; N, 9.50%.

Reaction of Methyleneketenimine $\mathbf{1}_{\mathbf{Dipp}}$ with CO_2

In a J. Young NMR tube, C_6D_6 (0.50 mL) was added to a mixture of **BAC** (32 mg, 0.14 mmol) and diisopropylphenyl isocyanide (25 mg, 0.14 mmol) and placed at room temperature for 3 hours. The formation of methyleneketenimine $\mathbf{1}_{\mathbf{Dipp}}$ (63% yield) was confirmed by NMR spectra analysis. After freeze-pump-thaw, CO_2 gas (1 atm) was introduced to the tube and the resulting orange solution was heated at 65 °C for 16.5 hours. The formation of azetidine-2,4-dione $\mathbf{7}_{\mathbf{Dipp}}$ (62% yield) was confirmed by NMR spectra analysis. The yield was determined by ^{1}H NMR integrals using 1,3,5-tri-*tert*-butylbenzene as an internal standard.

Isolation of Azetidine-2,4-dione $\mathbf{7}_{\mathbf{Dipp}}$

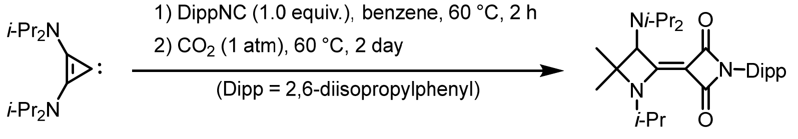

BAC $\mathbf{7}_{\mathbf{Dipp}}$

In a J. Young tube (50 mL) equipped with a magnetic stir bar, a benzene (2.0 mL) solution of 2,6-diisopropylphenyl isocyanide (97 mg, 0.52 mmol) was added to **BAC** (124 mg, 0.525 mmol) and stirred for 5 hours at room temperature. After freeze-pump-thaw, CO_2 gas (1 atm) was introduced to the tube and stirred for 5 minutes. After sealing the tube, the solution was stirred for 19 hours at 60 °C. The volatiles were removed in vacuo to afford a brown oil. The brown oil was purified by subjecting the crude mixture to silica gel column chromatography (eluent: CH_2Cl_2). The silica gel was deactivated with toluene containing 5% NEt_3. After the volatiles were removed in vacuo, the resulting pale orange solid was washed with cold hexane (0.1 mL) to afford colorless crystals of $\mathbf{7}_{\mathbf{Dipp}}$ in 45% yield (99 mg, 0.23 mmol).

$\mathbf{7}_{\mathbf{Dipp}}$: colorless crystals; mp 135 °C (decomp.); ^{1}H NMR (500 MHz, C_6D_6, 295 K, δ) 0.72 (brs, 3H, CH_3(*i*-Pr)), 0.87 (s, 3H, CH_3), 0.98-1.03 (m, 3H+3H+3H, CH_3(*i*-Pr)+CH_3(*i*-Pr)+CH_3), 1.16 (d, ^3J(H,H) = 7.0 Hz, 3H, CH_3(*i*-Pr)), 1.23 (d, ^3J(H,H) = 7.0 Hz, 3H, CH_3(*i*-Pr)), 1.27-1.32 (m, 3H+3H+3H, CH_3(i-Pr)+CH_3(*i*-Pr)), 1.37-1.40 (m, 3H+3H, CH_3(*i*-Pr)+CH_3(*i*-Pr)), 3.06 (brs, 1H, CH), 3.26 (brs. 1H, CH), 3.48 (sep+sep, ^3J(H,H) = 6.5 Hz, 1H+1H, CH(Ar)), 3.92 (s, 1H, CH), 4.42 (sep, ^3J(H,H) = 6.5 Hz, CH), 7.09-7.12 (m, 2 H, CH(Ar)), 7.19-7,21 (m, 1H, CH(Ar)); ^{13}C{^{1}H} NMR (126 MHz, C_6D_6, 297 K, δ) 20.4 (CH_3(*i*-Pr)), 21.4 (CH_3(*i*-Pr)), 22.2 (CH_3(*i*-Pr)), 23.3 (CH_3(*i*-Pr)), 23.9 (CH_3), 24.0 (CH_3(*i*-Pr)), 24.1 (CH_3(*i*-Pr)), 24.3 (CH_3(*i*-Pr)), 24.6 (CH_3(*i*-Pr)), 26.2 (CH_3), 26.3 (CH_3(*i*-Pr)), 30.08 (CH(Ar)), 30.13 (CH(Ar)), 43.4 (CH(*i*-Pr)), 50.2 (CH(*i*-Pr)+CH(*i*-Pr)*), 65.1 (CH),76.8 (CMe_2), 94.1 (C=*C*), 123.88 (CH(Ar)), 123.91 (CH(Ar)), 129.2 (C(Ar)), 129.4 (CH(Ar)), 148.30 (C(Ar)), 148.32 (C(Ar)), 156.2 (*C*=C), 169.7 (CO), 169.8 (CO). One CH_3(*i*-Pr) signal could not be determined possibly due to signal overlap. *Two CH(*i*-Pr) signals are overlapped; HRMS (FD+eiFi): Calcd for $C_{29}H_{45}N_3O_2$ $[M]^+$ 467.35118, Found. 467.35104; Elem. Anal. Calcd for $C_{29}H_{45}N_3O_2$: C, 74.48%; H, 9.70%; N, 8.98%. Found: C, 74.46%; H, 9.73%; N, 8.98%.

Reaction of Methyleneketenimine $\mathbf{1}_{\mathbf{Ad}}$ with H_2O

In a Schlenk tube (50 mL) equipped with a magnetic stir bar, a THF:H_2O (10:1) solution (6.6 mL) was added to methyleneketenimine $\mathbf{1}_{\mathbf{Ad}}$ (85 mg, 0.21 mmol). After stirring for an hour, a yellowish orange solution was obtained. The formation of **13** (99% yield) was confirmed by NMR spectra. The yield was determined by ^{1}H NMR integrals using 1,3,5-tri-*tert*-butylbenzene as an internal standard.

Isolation of the *E*/*Z*-Isomeric Mixture of Acrylamide 13

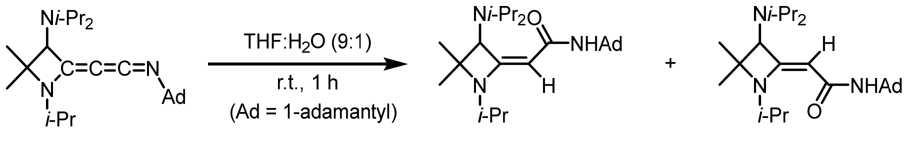

$\mathbf{1_{Ad}}$ (*E*)-**13** (*Z*)-**13**

mixture of *E*/*Z* isomer
(***E***:***Z*** = 0.7:1)

In a Schlenk tube (50 mL) equipped with a magnetic stir bar, a THF:H_2O (9:1) solution (6.9 mL) was added to methyleneketenimine $\mathbf{1_{Ad}}$ (70 mg, 0.18 mmol). After stirring for an hour, a reddish orange solution was obtained. The volatiles were removed in vacuo the resulting pale-yellow solid was washed with cold hexane (0.1 mL×2), affording a pale yellow solid of **13** in 78% yield (57 mg, 0.14 mmol).

13: pale yellow solid; mp 82-94 °C (decomp.); ^{1}H NMR (500 MHz, C_6D_6, 295 K, δ) 0.88 (brs. 3H, CH_3(*i*-Pr) of (*Z*)-**13**), 0.91 (d, 3H, ^3J(H,H) = 6.5 Hz, CH_3(*i*-Pr) of (*Z*)-**13**), 0.98-1.01 (m, 3H+3H+3H, CH_3 of (*Z*)-**13**+CH_3(*i*-Pr) of (*Z*)-**13**+CH_3(*i*-Pr) of (*E*)-**13**), 1.02 (d, ^3J(H,H) = 6.5 Hz, 3H, CH_3(*i*-Pr) of (*Z*)-**13**), 1.14 (s, 3H+3H+3H, CH_3 of (*Z*)-**13**+CH_3 of (*Z*)-**13**+CH_3 of (*E*)-**13**), 1.17 (d, ^3J(H,H) = 6.0 Hz, 3H, CH_3(*i*-Pr) of (*E*)-**13**), 1.18 (d, ^3J(H,H) = 7.0 Hz, 3H, CH_3(*i*-Pr) of (*E*)-**13**), 1.214 (s, 3H, CH_3 of (*E*)-**13**), 1.26 (d, ^3J(H,H) = 7.0 Hz, 3H, CH_3(*i*-Pr) of (*E*)-**13**), 1.38 (d, ^3J(H,H) = 6.5 Hz, 3H, CH_3(*i*-Pr) of (*Z*)-**13**), 1.52-1.64 (m, 6H+6H+3H, Ad of (*Z*)-**13**+Ad of (*E*)-**13**+CH_3(*i*-Pr) of (*E*)-**13**), 1.96 (brs, 3H+3H, Ad of (*Z*)-**13**+Ad of (*E*)-**13**), 2.13-2.19 (m, 6H+6H, Ad of (*Z*)-**13**+Ad of (*E*)-**13**), 3.07 (sep. ^3J(H,H) = 6.5 Hz, 1H, CH(*i*-Pr) of (*Z*)-**13**), 3.24-3.27 (m, 1H+1H, CH(*i*-Pr) of (*Z*)-**13**+CH(*i*-Pr) of (*E*)-**13**), 3.53-3.55 (m, 1H, CH(*i*-Pr) of (*Z*)-**13**), 3.57 (s, 1H, CH of (*E*)-**13**), 4.34 (s, 1H, C=CH of (*E*)-**13**), 4.37 (s, 1H, CH of (*Z*)-**13**), 4.45 (s, CH, C=CH of (*Z*)-**13**), 4.60 (s, 1H, NH of (*Z*)-**13**), 4.65 (s, 1H, NH of (*E*)-**13**), 5.61 (sep. ^3J(H,H) = 7.0 Hz, 1H, CH(*i*-Pr) of (*E*)-**13**). One CH_3(*i*-Pr) of (*E*)-**13**, one CH_3 of (*E*)-**13**, and two C*H*(*i*-Pr) of (*Z*)-**13** and (*E*)-**13** could not be determined possibly due to signal broadening and overlapping; ^{13}C{^{1}H} NMR (126 MHz, C_6D_6, 296 K, δ) 20.1 (CH_3(*i*-Pr) of (*Z*)-**13**), 20.5 (CH_3(*i*-Pr) of (*E*)-**13**), 21.3 (CH_3(*i*-Pr) of (*Z*)-**13**), 22.4 (CH_3(*i*-Pr) of (*E*)-**13**), 22.5 (CH_3(*i*-Pr) of (*Z*)-**13**), 23.3 (CH_3(*i*-Pr) of (*Z*)-**13**), 23.8 (CH_3(*i*-Pr) of (*E*)-**13**), 24.4 (CH_3 of (*Z*)-**13**), 25.0 (CH_3 of (*E*)-**13**), 25.7 (CH_3 of (*Z*)-**13**), 26.7 (CH_3(*i*-Pr) of (*Z*)-**13**), 27.8 (CH_3 of (*E*)-**13**), 30.2 (CH(Ad of (*Z*)-**13**)+CH(Ad of (*E*)-**13**)), 37.1 (CH_2(Ad of (*Z*)-**13**)+CH_2(Ad of (*E*)-**13**)), 42.7 (CH_2(Ad of (*E*)-**13**)), 42.9 (CH_2(Ad of (*Z*)-**13**)), 43.7 (CH(*i*-Pr) of (*Z*)-**13**), 45.7 (CH(*i*-Pr) of (*Z*)-**13**), 49.6 (CH(*i*-Pr) of (*E*)-**13**), 50.0 (CH(*i*-Pr) of (*Z*)-**13**), 51.0 (C(Ad of (*Z*)-**13**)), 51.1 (C(Ad of (*E*)-**13**)), 65.0 (CH of (*E*)-**13**), 67.0 (CH of (*Z*)-**13**), 73.6 (CMe_2 of (*E*)-**13**), 74.3 (CMe_2 of (*Z*)-**13**), 83.6 (C=*C* of (*E*)-**13**), 85.2 (C=*C* of (*Z*)-**13**), 160.0 (*C*=C of (*E*)-**13**), 163.0 (*C*=C of (*Z*)-**13**), 167.2 (CO of (*E*)-**13**), 167.3 (CO of (*Z*)-**13**). Three *C*H_3(*i*-Pr) signals and three *C*H(*i*-Pr) signals of (*E*)-**13** and one *C*H_3(*i*-Pr) signal of (*Z*)-**13** could not be observed, possibly due to signal broadening and overlapping; IR (KBr) ῦ/cm^{-1} 3311, 3286 (NH), 2962, 2906, 2857, 2848, 1657 (CO), 1601, 1531, 1493, 1456, 1437, 1404, 1394, 1385, 1362, 1342, 1309, 1290, 1278, 1269, 1248, 1211, 1186, 1169, 1155, 1146, 1122, 1115, 1101; HRMS (FD+eiFi): Calcd for $C_{26}H_{45}N_3O$ [M]$^+$ 415.35626, Found. 415.35616; Elem. Anal. Calcd for $C_{26}H_{45}N_3O$: C, 75.13%; H, 10.91%; N, 10.11%. Found: C, 67.481%; H, 9.640%; N, 8.590%. Numerous attempts of CHN analysis of **13** failed due to its thermal instability.

Reaction of Methyleneketenimine $\mathbf{1_{Ad}}$ with Xylyl Isocyanate

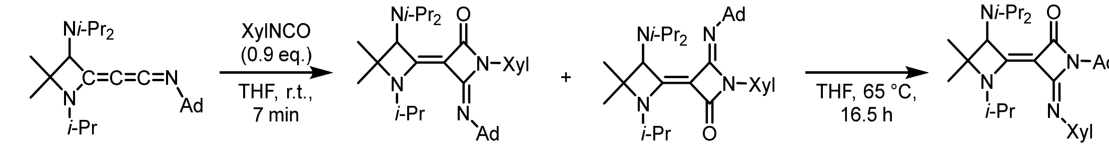

$\mathbf{1_{Ad}}$ (*E*)-**8** (67%*) (*Z*)-**8** (28%*) **9** (92%*)

(Ad = 1-adamantyl)
(Xyl = 2,6-dimethylphenyl)

In a J. Young tube (20 mL), methyleneketenimine $\mathbf{1_{Ad}}$ (80 mg, 0.20 mmol) was added to a THF (1.0 mL) solution of xylyl isocyanate (27 mg, 0.18 mmol). After stirring for 10 minutes at room temperature, a reddish orange solution was obtained. The formation of (*E*)-**8** (67%*) and (*Z*)-**8** (28% yield) was confirmed by NMR spectra analysis. After stirring for 16.5 hours at 65 °C, a brown solution was obtained. The formation of **9** (92%*) was confirmed by NMR spectra analysis. The yields were determined by ^{1}H NMR integrals using ferrocene as an internal standard.

Isolation of Azetidine-2-imine-4-one 8

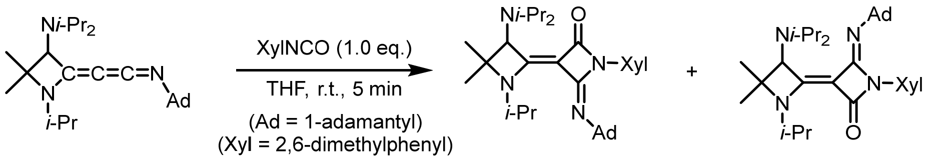

$\mathbf{1_{Ad}}$ (*E*)-**8** (*Z*)-**8**

In a sample tube (10 mL), methyleneketenimine $\mathbf{1_{Ad}}$ (95 mg, 0.24 mmol) was added to a THF (1.0 mL) solution of xylyl isocyanate (35 mg, 0.24 mmol). After the reaction mixture was stirred for 5 minutes at room temperature, the volatiles were removed. The resulting yellow solid was washed with hexane (1.0 mL) to give **8** as a pale-yellow solid in 55% yield (71 mg, 0.13 mmol).

8: pale yellow solid; mp 121-122 °C (decomp.); ^{1}H NMR (500 MHz, $CDCl_3$, 295 K, δ) 0.91 (brs. 3H+3H, CH_3(*i*-Pr) of (*E*)-**8**+CH_3(*i*-Pr) of (*Z*)-**8**), 1.12 (brs, 3H+3H+3H+3H, CH_3(*i*-Pr) of (*E*)-**8**+CH_3(*i*-Pr) of (*E*)-**8**+CH_3(*i*-Pr) of (*Z*)-**8**+CH_3(*i*-Pr) of (*Z*)-**8**), 1.24-1.28 (m, 3H+3H+3H+3H+3H+3H, CH_3(*i*-Pr) of (*E*)-**8**+CH_3(*i*-Pr) of (*E*)-**8**+CH_3(*i*-Pr) of (*E*)-**8**+CH_3(*i*-Pr) of (*Z*)-**8**+CH_3(*i*-Pr) of (*Z*)-**8**+CH_3(*i*-Pr) of (*Z*)-**8**) 1.35 (s, 3H, CH_3 of (*Z*)-**8**), 1.36 (s, 3H, CH_3 of (*E*)-**8**), 1.41 (s, 3H+3H, CH_3 of (*E*)-**8**+CH_3 of (*Z*)-**8**), 1.44-1.46 (m, 6H+6H, Ad of (*E*)-**8**+Ad of (*Z*)-**8**), 1.53-1.60 (m, 6H+6H, Ad of (*E*)-**8**+Ad of (*Z*)-**8**), 1.75-1.82 (m, 3H+3H, Ad of (*E*)-**8**+Ad of (*Z*)-**8**), 2.21 (s, 3H, CH_3(Ar) of (*E*)-**8**), 2.22 (s, 3H, CH_3(Ar) of (*Z*)-**8**), 2.25 (s, 3H, CH_3(Ar) of (*Z*)-**8**), 2.27 (s, 3H, CH_3(Ar) of (*E*)-**8**), 3.22-3.23 (m, 1H+1H, CH(*i*-Pr) of (*E*)-**8**+CH(*i*-Pr) of (*Z*)-**8**), 3.29-3.41 (m, 1H+1H, CH(*i*-Pr) of (*E*)-**8**+CH(*i*-Pr) of (*Z*)-**8**), 4.01 (s, 1H, CH of (*E*)-**8**), 4.06 (s, 1H, CH of (*Z*)-**8**), 4.57 (sep., 1H, ^3J(H,H) = 6.5 Hz, CH(*i*-Pr) of (*Z*)-**8**), 4.94 (sep., 1H, ^3J(H,H) = 6.5 Hz, CH(*i*-Pr) of (*E*)-**8**), 6.99-7.11 (m, 3H+3H, CH(Ar of (*E*)-**8**+CH(Ar of (*Z*)-**8**)); $^{13}C\{^1H\}$ NMR (126 MHz, $CDCl_3$, 296 K, δ) 18.7 (CH_3(Ar) of (*E*)-**8**), 18.8 (CH_3(Ar) of (*Z*)-**8**), 18.9 (CH_3(Ar) of (*Z*)-**8**), 19.0 (CH_3(Ar) of (*E*)-**8**), 20.5 (CH_3(*i*-Pr) of (*E*)-**8**), 20.8 (CH_3(*i*-Pr) of (*Z*)-**8**), 21.2 (CH_3(*i*-Pr) of (*Z*)-**8**), 21.6 (CH_3(*i*-Pr) of (*E*)-**8**), 22.2 (CH_3(*i*-Pr) of (*E*)-**8**), 22.4 (CH_3(*i*-Pr) of (*E*)-**8**), 22.6 (CH_3(*i*-Pr) of (*Z*)-**8**), 22.8 (CH_3(*i*-Pr) of (*Z*)-**8**), 23.1 (CH_3(*i*-Pr) of (*E*)-**8**), 23.2 (CH_3(*i*-Pr) of (*Z*)-**8**), 24.5 (CH_3 of (*E*)-**8**), 24.6 (CH_3 of (*Z*)-**8**), 26.4 (CH_3(*i*-Pr) of (*E*)-**8**), 26.6 (CH_3(*i*-Pr) of (*Z*)-**8**), 26.8 (CH_3 of (*Z*)-**8**), 26.9 (CH_3 of (*E*)-**8**), 29.71 (CH(Ad of (*E*)-**8**)), 29.74 (CH(Ad of (*Z*)-**8**)), 36.31 (CH_2(Ad of (*Z*)-**8**)), 36.33 (CH_2(Ad of (*E*)-**8**)), 43.0 (CH(*i*-Pr) of (*E*)-**8**+CH(*i*-Pr) of (*Z*)-**8**), 43.6 (CH_2(Ad of (*Z*)-**8**)), 43.8 (CH_2(Ad of (*E*)-**8**)), 49.6 (CH(*i*-Pr) of (*E*)-**8**), 49.7 (CH(*i*-Pr) of (*Z*)-**8**), 49.8 (CH(*i*-Pr) of (*Z*)-**8**), 50.2 (CH(*i*-Pr) of (*E*)-**8**), 53.9 (C(Ad of (*E*)-**8**)), 54.5 (C(Ad of (*Z*)-**8**)), 64.6 (CH of (*E*)-**8**+CH of (*Z*)-**8**), 76.0 (*C*Me$_2$ of (*Z*)-**8**), 76.1 (*C*Me$_2$ of (*E*)-**8**), 96.1 (C=*C* of (*Z*)-**8**), 96.4 (C=*C* of (*E*)-**8**), 127.97 (CH(Ar of (*E*)-**8**+Ar of (*Z*)-**8**)), 128.01 (CH(Ar of (*E*)-**8**+Ar of (*Z*)-**8**)), 128.04 (CH(Ar of (*E*)-**8**+Ar of (*Z*)-**8**)), 136.2 (C(Ar of (*E*)-**8**)), 136.6 (C(Ar of (*Z*)-**8**)), 138.21 (C(Ar of (*E*)-**8**)), 138.24 (C(Ar of (*Z*)-**8**)), 138.3 (C(Ar of (*E*)-**8**+Ar of (*Z*)-**8**)), 144.4 (*C*=N of (*Z*)-**8**), 144.6 (*C*=N of (*E*)-**8**), 154.2 (*C*=C of (*E*)-**8**+*C*=C of (*Z*)-**8**), 170.5 (*C*=O of (*Z*)-**8**), 171.0 (*C*=O of (*E*)-**8**); IR [cm^{-1}]: ṽ (KBr) = 2956, 2931, 2902, 2850, 1788, 1672, 1649, 1591, 1466, 1408, 1363, 1308, 1242, 1213, 1184, 1126, 1095, 1065, 1030, 1001, 980, 906, 862, 818, 796, 661, 548, 526, 472; HRMS (FD+eiFi): Calcd for $C_{35}H_{52}N_4O$ $[M]^+$ 544.41411, Found. 544.41402; Elem. Anal. Calcd for $C_{35}H_{52}N_4O$: C, 77.16%; H, 9.62%; N, 10.28%. Found: C, 76.78%; H, 9.74%; N, 10.28%.

Isolation of Azetidine-2-imine-4-one 9

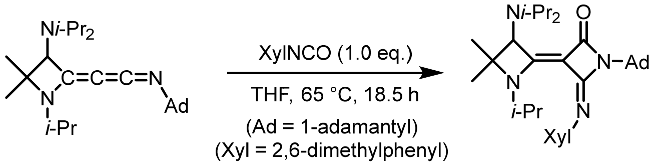

$\mathbf{1_{Ad}}$ **9**

In a J. Young tube (20 mL), methyleneketenimine $\mathbf{1_{Ad}}$ (120 mg, 0.30 mmol) was added to a THF (1.0 mL) solution of xylyl isocyanate (44 mg, 0.30 mmol). After the reaction mixture was stirred for 18.5 hours at 65 °C, the volatiles were removed. Recrystallization from hexamethyldisiloxane (0.5 mL) at -30 °C provided **9** as a pale-yellow solid in 51% yield (84 mg, 0.15 mmol).

9: pale yellow solid; mp 117-119 °C (decomp.); ^{1}H NMR (500 MHz, $CDCl_3$, 295 K, δ) 0.70 (brs, 3H, CH_3(*i*-Pr)), 0.88 (brs, 3H+3H, CH_3(*i*-Pr)+CH_3(*i*-Pr)), 1.09 (d, ^3J(H,H) = 6.0 Hz, 3H, CH_3(*i*-Pr)), 1.14 (d, ^3J(H,H) = 6.0 Hz, 3H, CH_3(*i*-Pr)), 1.24-1.28 (m, 3H+3H+3H, CH_3+CH_3+CH_3(*i*-Pr)), 1.58-1.66 (m, 6H, Ad), 2.01 (brs, 3H, Ad), 2.16 (s, 3H, CH_3(Ar)), 2.18 (s, 3H, CH_3(Ar)), 2.26 (brs, 6H, Ad), 3.23-3.24 (m, 2H, CH(*i*-Pr)), 3.99 (s, 1H, CH), 6.77 (t, ^3J(H,H) = 7.5 Hz, 1H, CH(Ar)), 6.95 (t, ^3J(H,H) = 7.5 Hz, 1H, CH(Ar)), 6.98 (d, ^3J(H,H) = 7.5 Hz, 1H, CH(Ar)). One C*H*(*i*-Pr) of **9** could not be detected; $^{13}C\{^1H\}$ NMR (126 MHz, $CDCl_3$, 296 K, δ) 18.0 (CH_3(Ar)), 19.0 (CH_3(Ar)), 20.5 (CH_3(*i*-Pr)), 21.5 (CH_3(*i*-Pr)), 22.0 (CH_3(*i*-Pr)), 22.4 (CH_3(*i*-Pr)), 23.1 (CH_3(*i*-Pr)), 24.6 (CH_3), 26.6 (CH_3(*i*-Pr)), 26.8 (CH_3), 29.5 (CH(Ad)), 36.4 (CH_2(Ad)), 41.0 (CH_2(Ad)), 43.0 (CH(*i*-Pr)), 49.5 (CH(*i*-Pr)), 56.8 (C(Ad)), 65.2 (CH), 76.6 (*C*Me$_2$), 95.1 (C=*C*), 122.3 (CH(Ar)), 127.2 (C(Ar)), 127.5 (CH(Ar)), 128.1 (CH(Ar)), 129.6 (C(Ar)), 146.8 (C(Ar)), 151.4 (*C*=N), 153.6 (*C*=C), 170.6 (*C*=O). One CH signal could not be detected; IR [cm^{-1}]: ṽ (KBr) = 2964, 2933, 2906, 2850, 1792, 1666, 1591, 1466, 1406, 1362, 1336, 1308, 1242, 1209, 1186, 1122, 1051, 1024, 972, 926, 893, 839, 814, 760, 735, 714, 681, 642, 552, 476; HRMS (APCI (positive)): Calcd for $C_{35}H_{52}N_4O$ $[M]^+$ 544.41356, Found. 544.41359; Elem. Anal. Calcd for $C_{35}H_{52}N_4O$: C, 77.16%; H, 9.62%; N, 10.28%. Found: C, 77.06%; H, 9.74%; N, 10.12%.

Reaction of *M*ethyleneketenimine 1_{Ad} with Xylyl Azide

In a sample tube (10 mL) equipped with a magnetic stir bar, a THF (1.0 mL) solution of xylyl azide (14 mg, 0.097 mmol) was added to methyleneketenimine $\mathbf{1_{Ad}}$ (38 mg, 0.097 mmol). After stirring for 21.5 hours at 65 °C, a reddish orange solution was obtained. The formation of triazole **10** (38% yield) was confirmed by NMR spectra analysis. The yield was determined by ^{1}H NMR integrals using 1,3,5-tri-*tert*-butylbenzene as an internal standard.

Isolation of Triazole 10

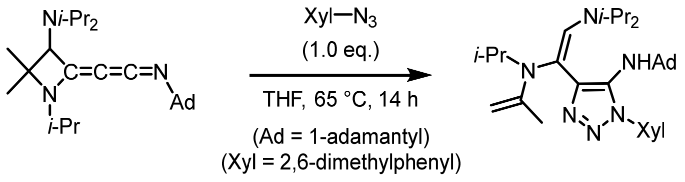

$\mathbf{1_{Ad}}$ **10**

In a J. Young tube (20 mL), methyleneketenimine $\mathbf{1_{Ad}}$ (91 mg, 0.23 mmol) was added to a THF (1.0 mL) solution of xylyl azide (33 mg, 0.23 mmol). After the reaction mixture was stirred for 14 hours at 65 °C, the volatiles were removed and a reddish-brown oil was obtained. Recrystallization from hexane (0.2 mL) at –35°C gave **10** as a pale-yellow solid in 21% yield (26 mg, 0.047 mmol).

10: pale yellow solid; mp 120 °C (decomp.); ^{1}H NMR (500 MHz, C_6D_6, 295 K, δ) 1.04 (d,^3J(H,H) = 7.0 Hz, 12H, CH_3(*i*-Pr)), 1.26 (d, ^3J(H,H) = 6.5 Hz, 6H, CH_3(*i*-Pr)), 1.34-1.42 (m, 6H, Ad), 1.527-1.531 (m, 6H, Ad), 1.80 (brs, 3H, Ad), 2.21 (s, 3H, CH_3), 2.22 (s, 6H, CH_3(Ar)), 3.51 (sep, 2H, ^3J(H,H) = 6.5 Hz, CH), 3.98-4.05 (sep+s, 1H+1H, ^3J(H,H) = 7.0 Hz, CH+C*H*H), 4.24 (s, 1H, NH), 4.29 (s, 1H, CH*H*), 6.23 (s, 1H, CH), 6.89 (d, 2H, ^3J(H,H) = 7.5 Hz, CH(Ar)), 7.00 (t, 1H, ^3J(H,H) = 7.5 Hz, CH(Ar)); ^{13}C{^{1}H} NMR (126 MHz, C_6D_6, 297 K, δ) 18.9 (CH_3(Ar)), 21.4 (CH_3), 22.6 (CH_3), 23.9 (CH_3), 30.4 (CH(Ad)), 36.5 (CH_2(Ad)), 44.0 (CH_2(Ad)), 47.0 (CH(*i*-Pr)), 50.1 (CH(*i*-Pr)), 54.9 (C(Ad)), 83.3 (CH_2), 104.0 (*C*=CH_2), 128.8 (CH(Ar)), 129.4 (CH(Ar)), 136.6 (C(Ar)), 137.4 (C=*C*HN(*i*-Pr)$_2$), 137.6 (C(Ar)), 137.7 (*C*=C), 139.9 (C=*C*), 150.4 (*C*=CHN(*i*-Pr)$_2$); HRMS (FD+eiFi): Calcd for $C_{34}H_{52}N_6$ [M]$^+$ 544.42534, Found. 544.42523; Elem. Anal. Calcd for $C_{34}H_{52}N_6$: C, 74.95%; H, 9.62%; N, 15.43%. Found: C, 74.92%; H, 9.70%; N, 15.20%.

Reaction of Methyleneketenimine 1_{Ad} with Dimethyl Acetylenedicarboxylate

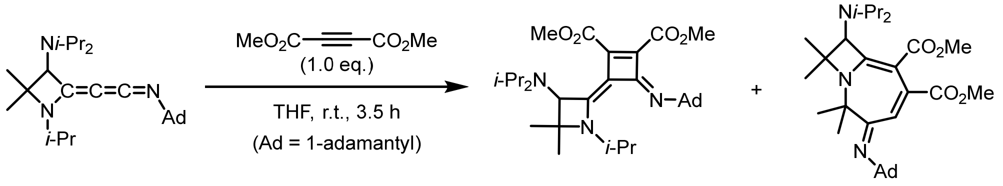

$\mathbf{1_{Ad}}$ **11** (26%*) **12** (11%*)

In a sample tube (10 mL) equipped with a magnetic stir bar, a THF (1.0 mL) solution of dimethyl acetylenedicarboxylate (42 mg, 0.29 mmol) was added to methyleneketenimine $\mathbf{1_{Ad}}$ (117 mg, 0.29 mmol). After stirring for 3.5 hours at room temperature, a reddish-brown solution was obtained. The formation of **11** (26% yield) and **12** (11% yield) was confirmed by NMR spectra analysis. The yield was determined by ^{1}H NMR integrals using 1,3,5-tri-*tert*-butylbenzene as an internal standard. The low yield of **12** was due to the generation of **11** and other unidentifiable minor byproducts. Further purification of **11** failed due to its instability in silica gel column.

11: ^{1}H NMR (500 MHz, C_6D_6, 293 K, δ) 0.77 (d,^3J(H,H) = 6.5 Hz, 3H, CH_3(*i*-Pr)), 0.87-0.91 (m, 3H, CH_3(*i*-Pr)), 1.04-1.06 (m, 3H+3H+3H, CH_3(*i*-Pr)+CH_3(*i*-Pr)+CH_3), 1.13-1.16 (m, 3H+3H+3H, CH_3(*i*-Pr)+CH_3(*i*-Pr)+CH_3),1.67-1.69 (m, 6H, Ad), 2.17 (brs, 3H, Ad), 2.31-2.47 (m, 6H, Ad), 3.02 (sep, 1H, ^3J(H,H) = 7.0 Hz, CH), 3.29 (sep, 1H, ^3J(H,H) = 6.5 Hz, CH), 3.38 (s, 3H, COOMe), 3.57 (s, 3H, COOMe), 3.95 (s, 1H, CH), 5.35 (sep, 1H, ^3J(H,H) = 7.0 Hz, CH); ^{13}C{^{1}H} NMR (126 MHz, C_6D_6, 295 K, δ) 20.9 (CH_3), 22.0 (CH_3), 22.70 (CH_3), 22.72 (CH_3), 24.4 (CH_3), 24.8 (CH_3), 25.8 (CH_3), 27.2 (CH_3), 30.8 (CH(Ad)), 36.8 (CH_2(Ad)), 43.7 (CH_2(Ad)), 43.9 (CH(*i*-Pr)), 48.7 (CH(*i*-Pr)), 50.5 (CH(*i*-Pr)), 51.0 (OMe), 51.7 (OMe), 56.7 (C(Ad)), 65.0 (*C*H), 74.4 (*C*Me$_2$), 105.9 (*C*), 121.3 (*C*), 149.0 (*C*), 153.2 (*C*), 161.2 (*C*O), 165.1 (*C*O), 168.6 (*C*); HRMS (FD+eiFi): Calcd for $C_{32}H_{49}N_3O_4$ [M]$^+$ 539.37231, Found. 539.37231.

Isolation of Azepin-3-imine 12

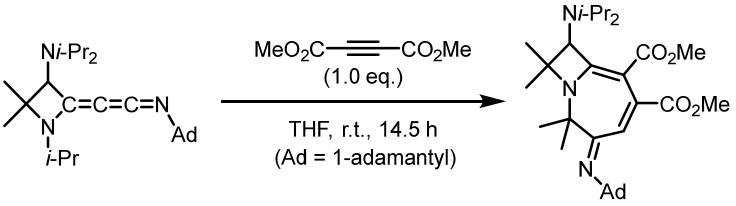

$\mathbf{1_{Ad}}$ **12**

In a sample tube (10 mL) equipped with a magnetic stir bar, a THF (1.0 mL) solution of dimethyl acetylenedicarboxylate (28 mg, 0.20 mmol) was added to methyleneketenimine $\mathbf{1_{Ad}}$ (81 mg, 0.20 mmol). After stirring for 14.5 hours at room temperature, a reddish-brown solution was obtained. After the volatiles were removed in vacuo, the resulting brown oil was recrystallized from Et_2O (0.5 mL) at -35 °C to afford colorless crystals of **12** in 6% yield (6.6 mg).

12: colorless crystals; mp 140 °C (decomp.); ^{1}H NMR (500 MHz, C_6D_6, 293 K, δ) 0.82 (d,^3J(H,H) = 6.5 Hz, 3H, CH_3(*i*-Pr)), 1.03-1.06 (m, 3H+3H+3H, CH_3(*i*-Pr)+CH_3(*i*-Pr)+CH_3), 1.11 (s, 3H, CH_3), 1.25 (s, 3H, CH_3), 1.31 (d,^3J(H,H) = 6.5 Hz, 3H, CH_3(*i*-Pr)), 1.58-1.66 (m, 3H+6H, CH_3+Ad), 1.97-2.09 (m, 9H, Ad), 3.07 (sep, 1H, ^3J(H,H) = 6.5 Hz, CH), 3.25 (sep, 1H, ^3J(H,H) = 6.5 Hz, CH), 3.47 (s, 3H, COOMe), 3.51 (s, 3H, COOMe), 4.40 (s, 1H, CH), 6.76 (s, 1H, CH); ^{13}C{^{1}H} NMR (126 MHz, C_6D_6, 294 K, δ) 22.6 (CH_3), 22.9 (CH_3), 23.3 (CH_3), 23.4 (CH_3), 24.7 (CH_3), 26.4 (CH_3), 26.9 (CH_3), 27.0 (CH_3), 30.3 (CH(Ad)), 37.0 (CH_2(Ad)), 43.36 (CH_2(Ad)), 43.45 (CH(*i*-Pr)), 49.1 (CH(*i*-Pr)), 50.9 (CH_3), 52.0 (CH_3), 56.8(C(Ad)), 66.5 (C=*C*CO_2Me), 66.7 (CH), 80.3 (*C*Me_2), 94.7 (HC=*C*), 119.9 (H*C*=C), 133.8 (CN), 158.6 (CMe_2), 163.2 (*C*=CCO_2Me), 167.3 (*C*O), 170.8 (*C*O); HRMS (FD+eiFi): Calcd for $C_{32}H_{49}N_3O_4$ $[M]^+$ 539.37231, Found. 539.37223; Elem. Anal. Calcd for $C_{32}H_{49}N_3O_4$: C, 71.21%; H, 9.15%; N, 7.79%. Found: C, 71.16%; H, 9.23%; N, 7.69%.

2. NMR spectra

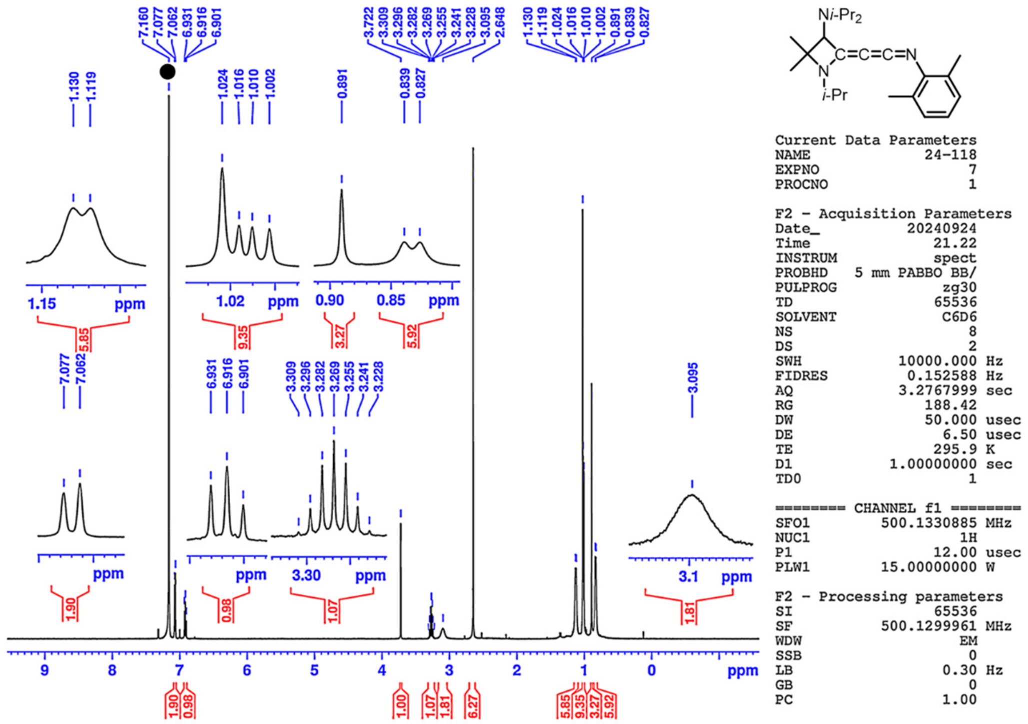

Figure S1. ^{1}H NMR spectrum of $\mathbf{1}_{\mathbf{Xyl}}$ in C_6D_6 at 296 K (● = C_6D_5H).

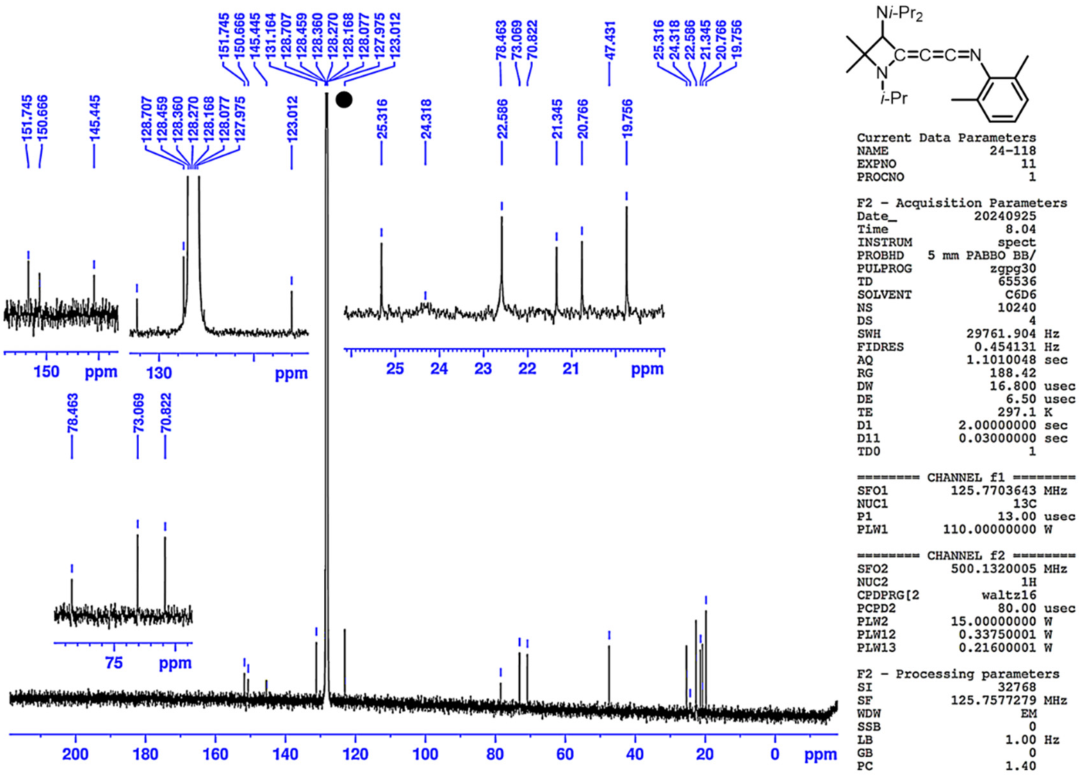

Figure S2. $^{13}C\{^{1}H\}$ NMR spectrum of $\mathbf{1}_{\mathbf{Xyl}}$ in C_6D_6 at 297 K (● = C_6D_6).

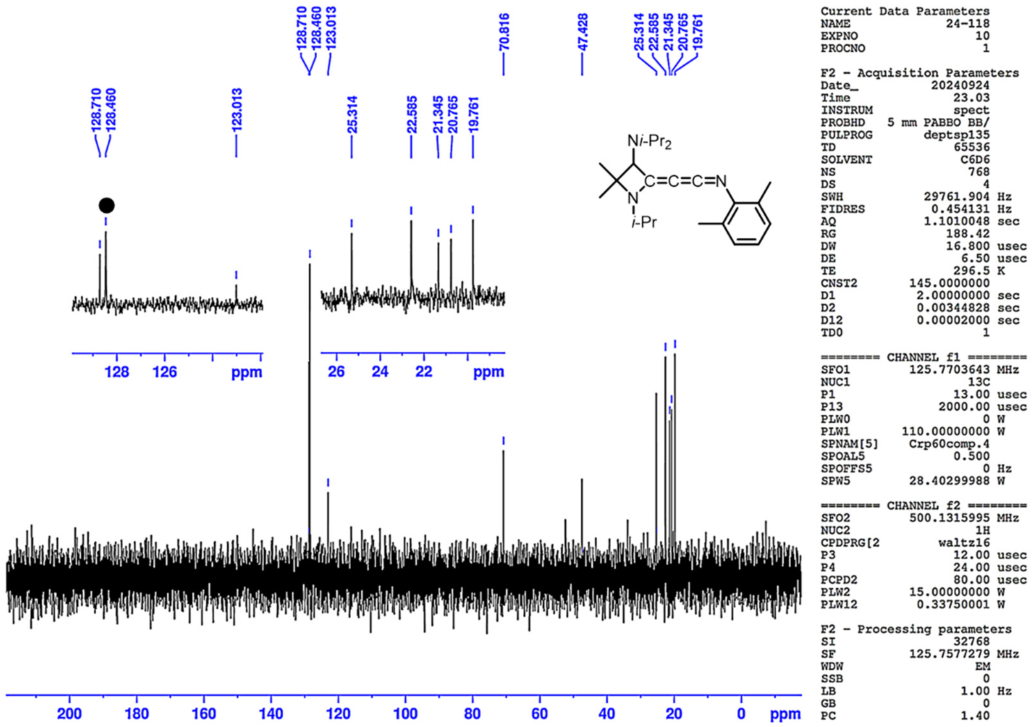

Figure S3. $^{13}C\{^{1}H\}$ (DEPT135) NMR spectrum of $\mathbf{1}_{Xyl}$ in C_6D_6 at 297 K (● = C_6D_5H).

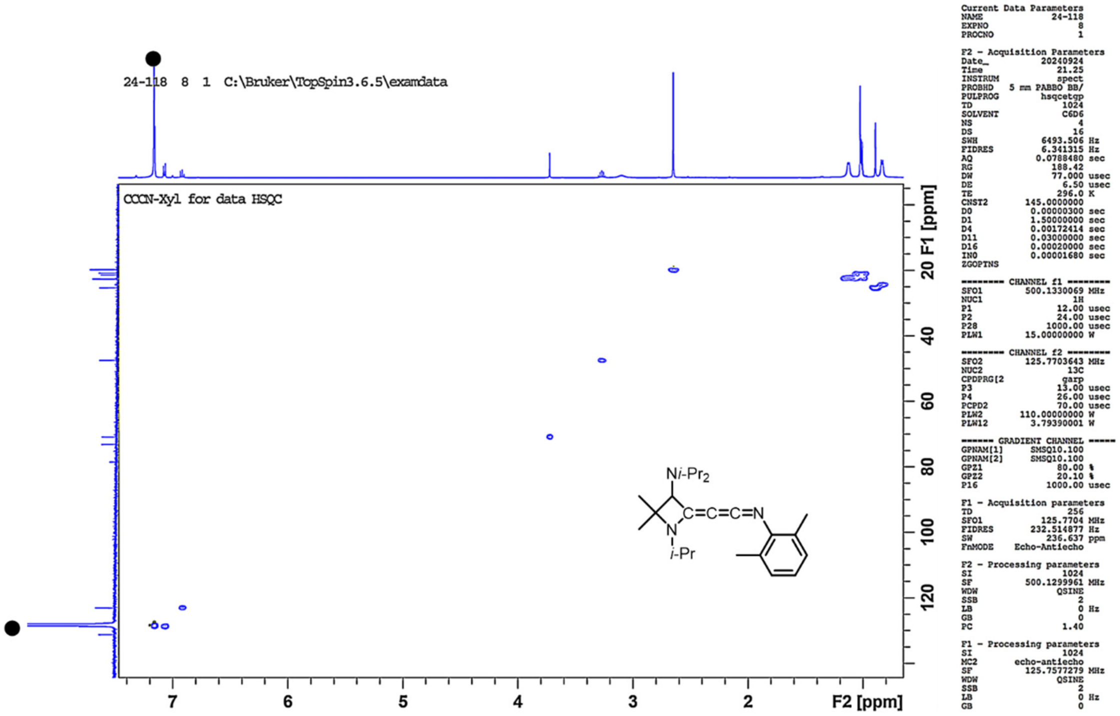

Figure S4. ^{1}H-^{13}C HSQC NMR spectrum of $\mathbf{1}_{Xyl}$ in C_6D_6 at 296 K (● = C_6D_5H & C_6D_6).

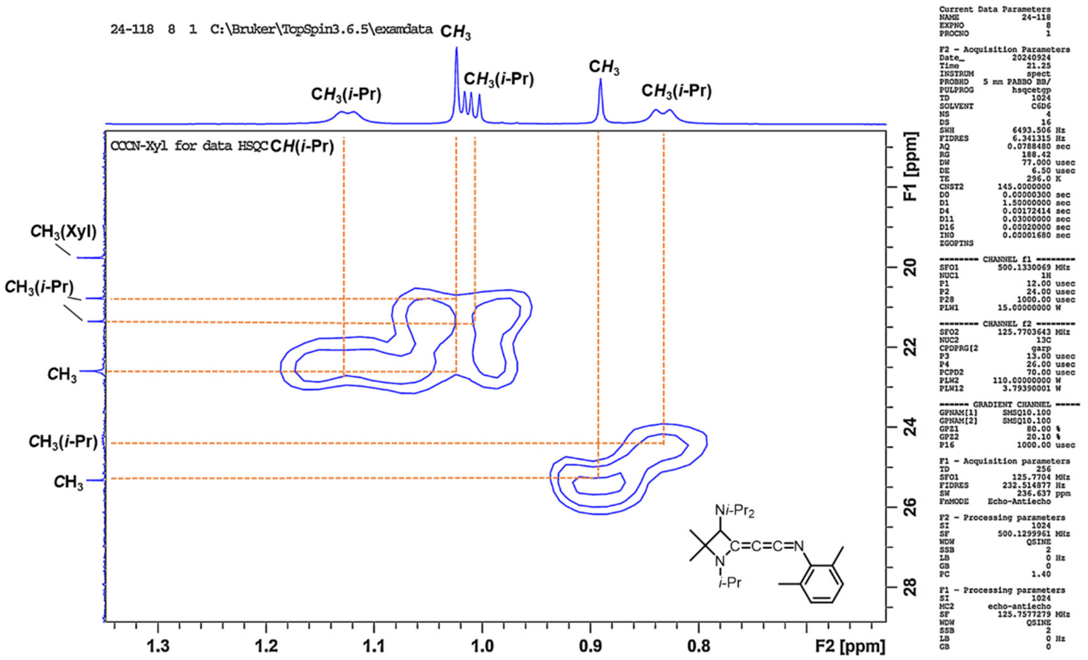

Figure S5. ^{1}H-^{13}C HSQC (magnified) NMR spectrum of $\mathbf{1}_{\mathbf{Xyl}}$ in C_6D_6 at 296 K.

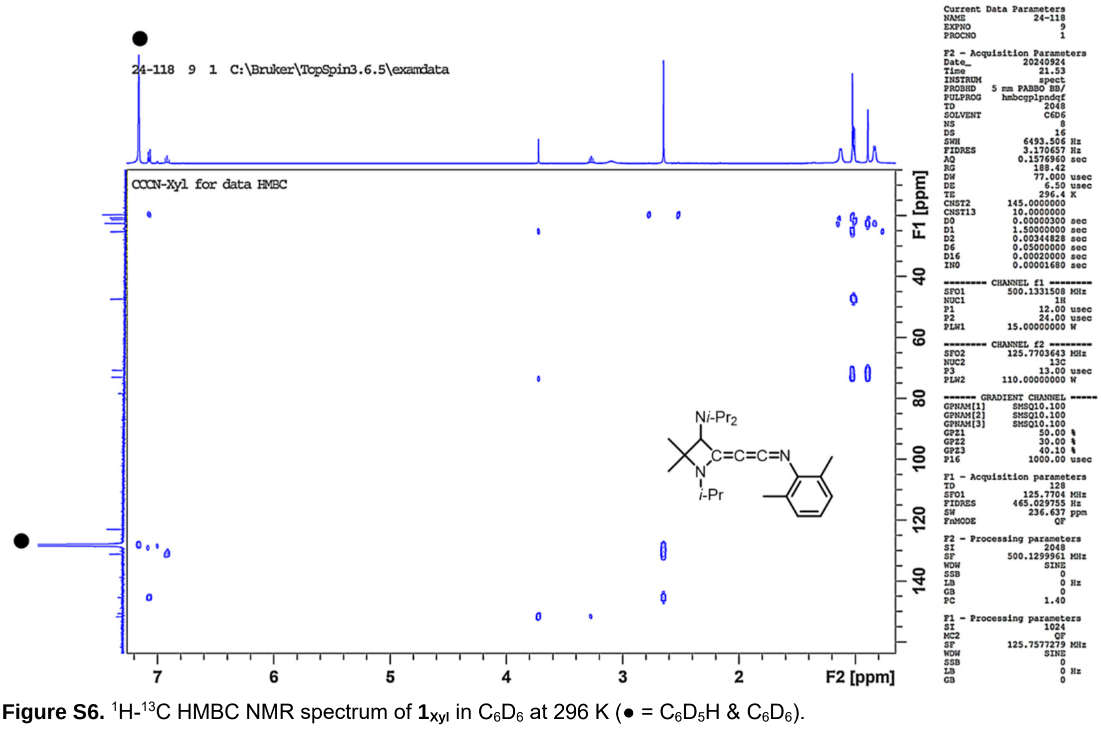

Figure S6. ^{1}H-^{13}C HMBC NMR spectrum of $\mathbf{1}_{\mathbf{Xyl}}$ in C_6D_6 at 296 K (● = C_6D_5H & C_6D_6).

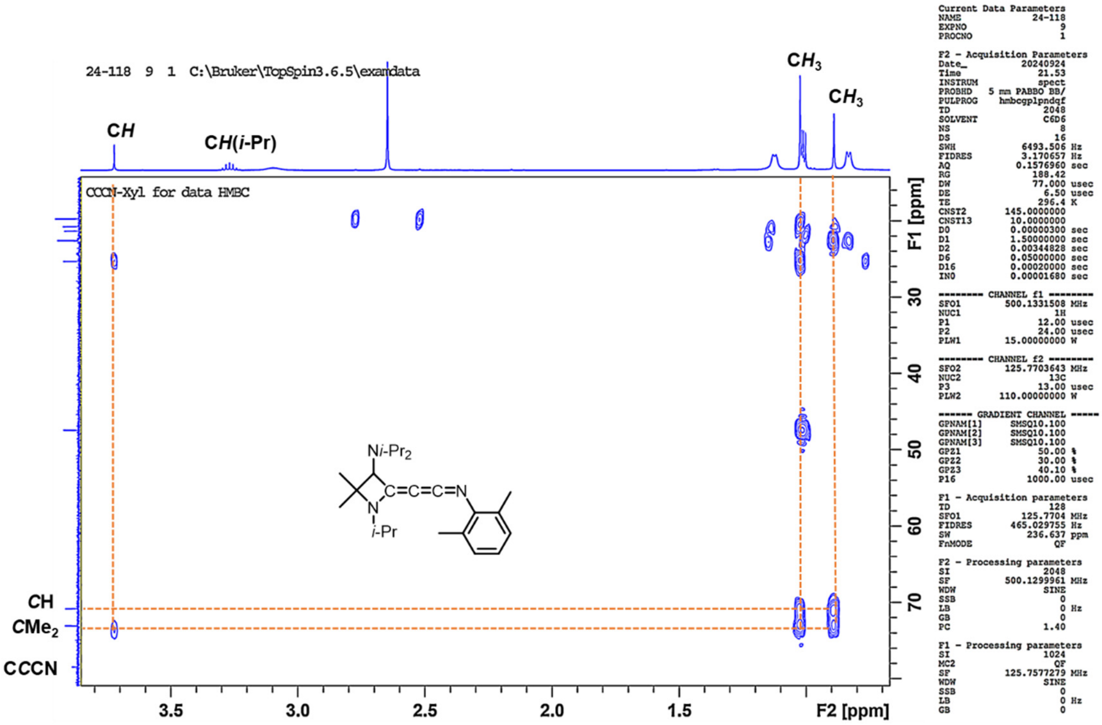

Figure S7. ^{1}H-^{13}C HMBC (magnified) NMR spectrum of $\mathbf{1}_{\mathbf{Xyl}}$ in C_6D_6 at 296 K.

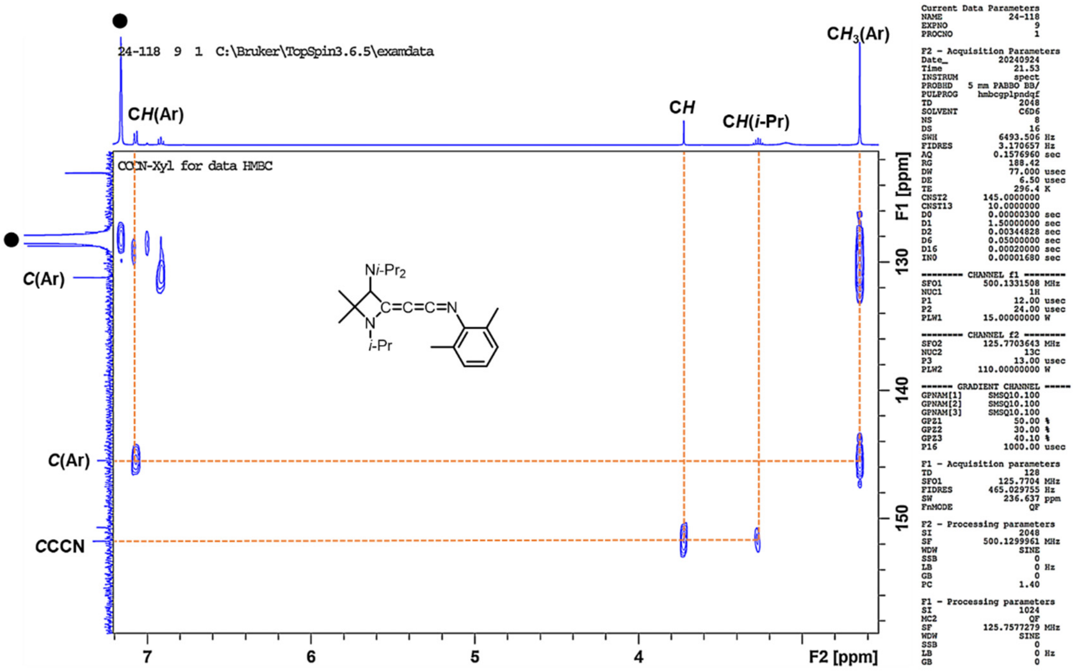

Figure S8. ^{1}H-^{13}C HMBC (magnified) NMR spectrum of $\mathbf{1}_{\mathbf{Xyl}}$ in C_6D_6 at 296 K (● = C_6D_5H & C_6D_6).

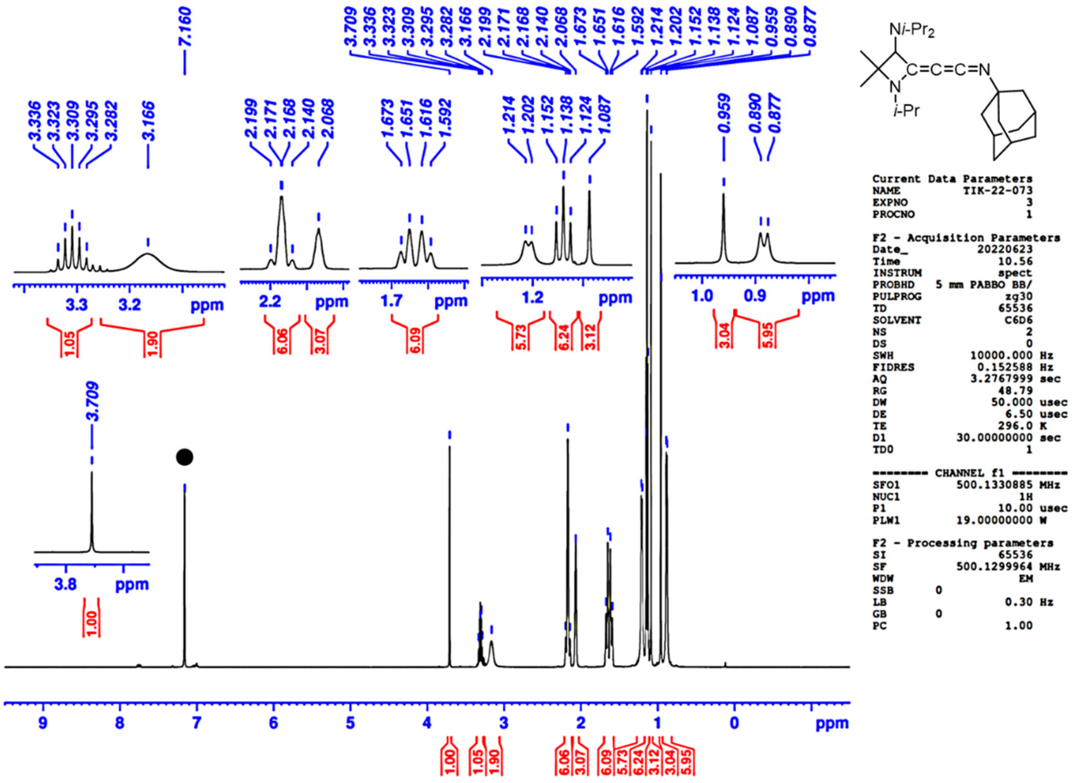

Figure S9. ^{1}H NMR spectrum of $\mathbf{1_{Ad}}$ in C_6D_6 at 296 K (● = C_6D_5H).

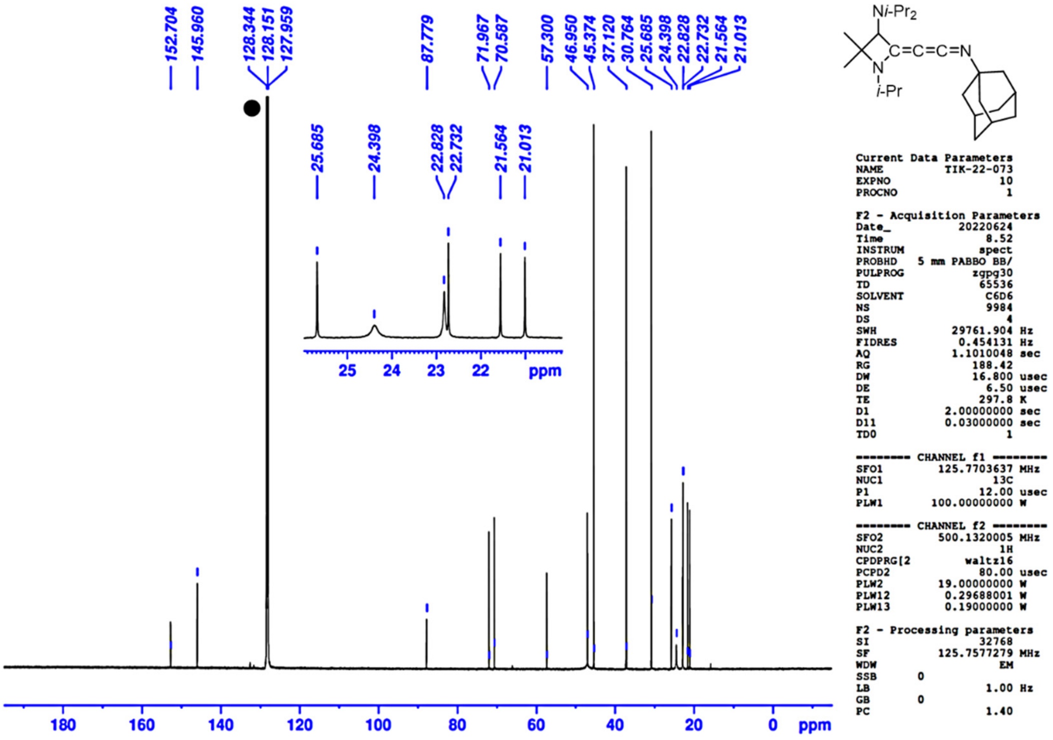

Figure S10. $^{13}C\{^{1}H\}$ NMR spectrum of $\mathbf{1_{Ad}}$ in C_6D_6 at 298 K (● = C_6D_6).

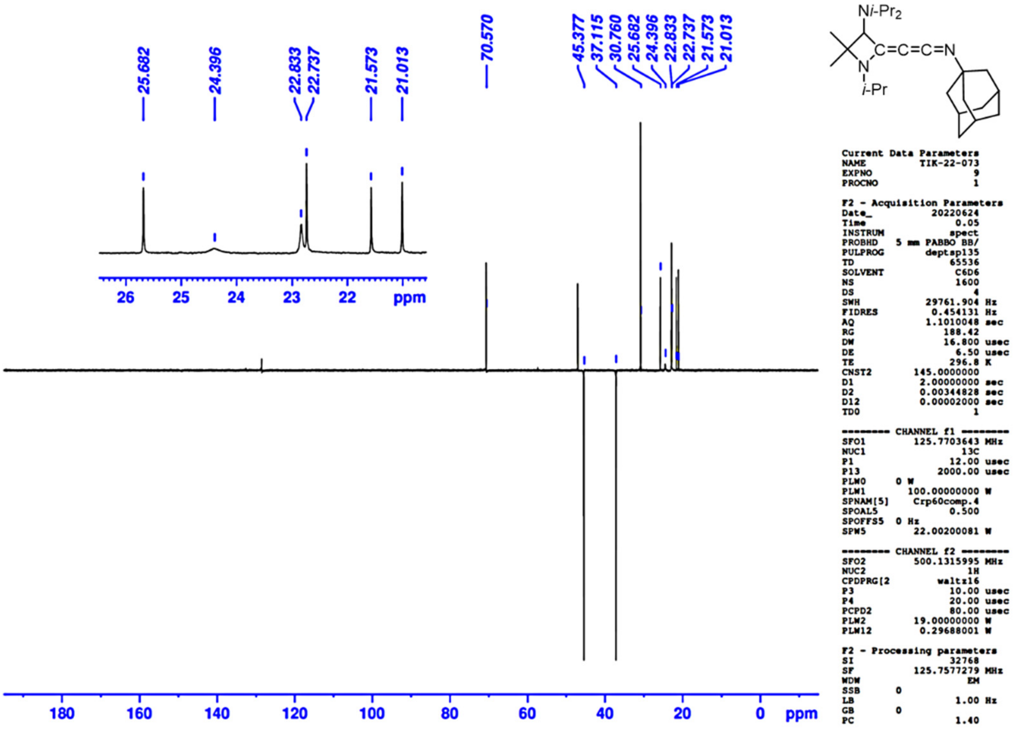

Figure S11. $^{13}C\{^{1}H\}$ (DEPT135) NMR spectrum of $\mathbf{1}_{\mathbf{Ad}}$ in C_6D_6 at 297 K.

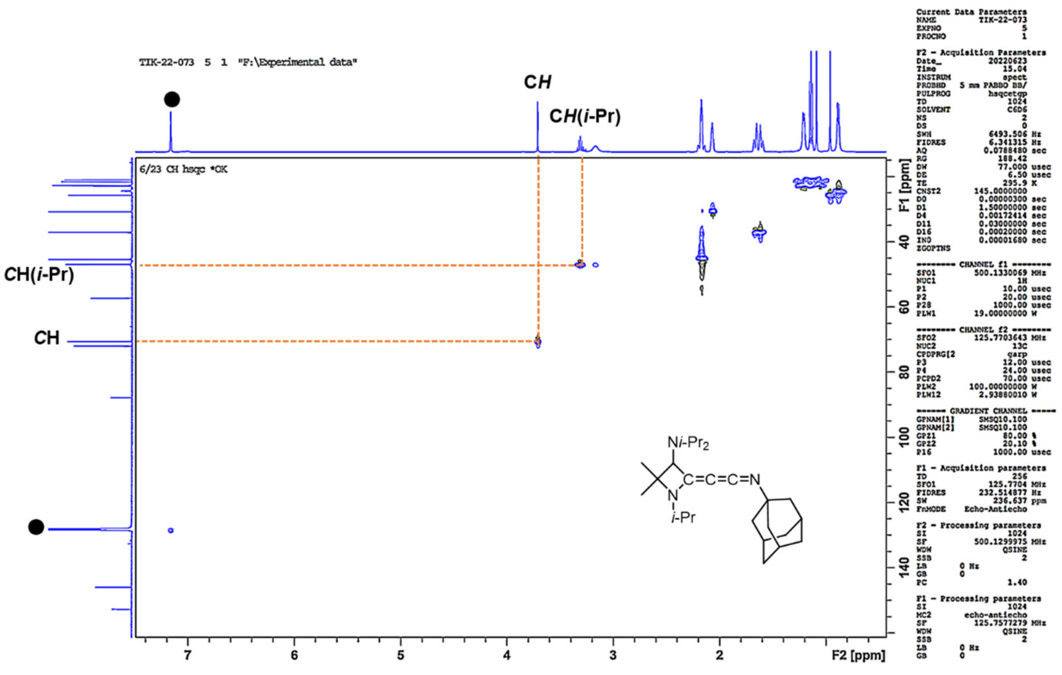

Figure S12. ^{1}H-^{13}C HSQC NMR spectrum of $\mathbf{1}_{\mathbf{Ad}}$ in C_6D_6 at 296 K (● = C_6D_5H & C_6D_6).

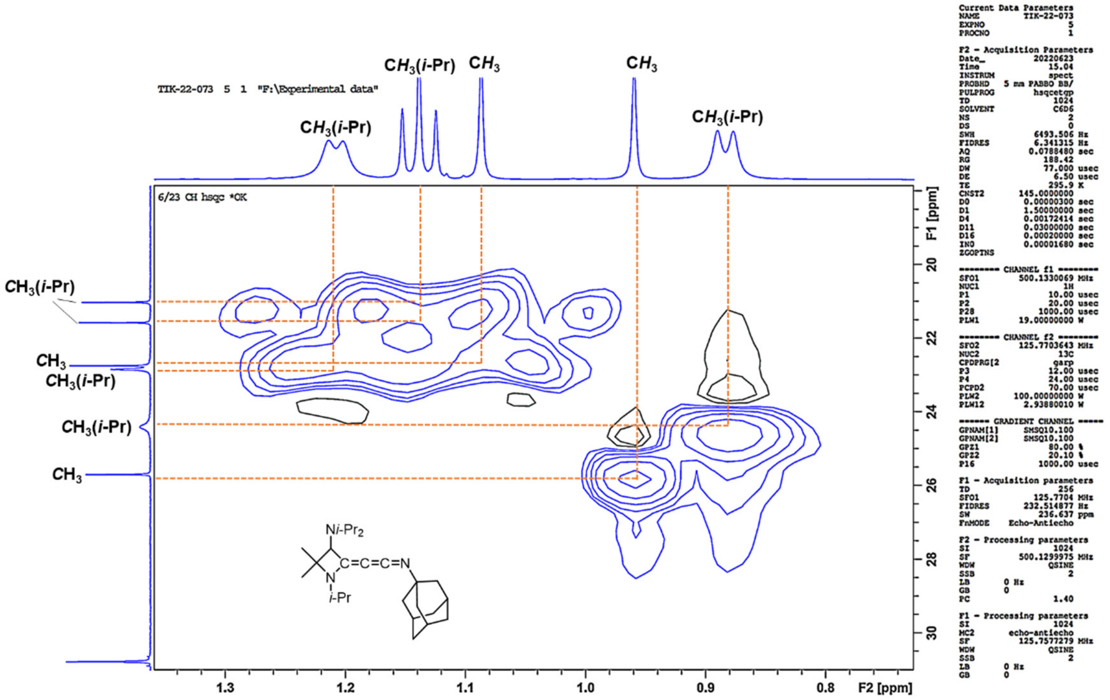

Figure S13. ^{1}H-^{13}C HSQC (magnified) NMR spectrum of $\mathbf{1_{Ad}}$ in C_6D_6 at 296 K.

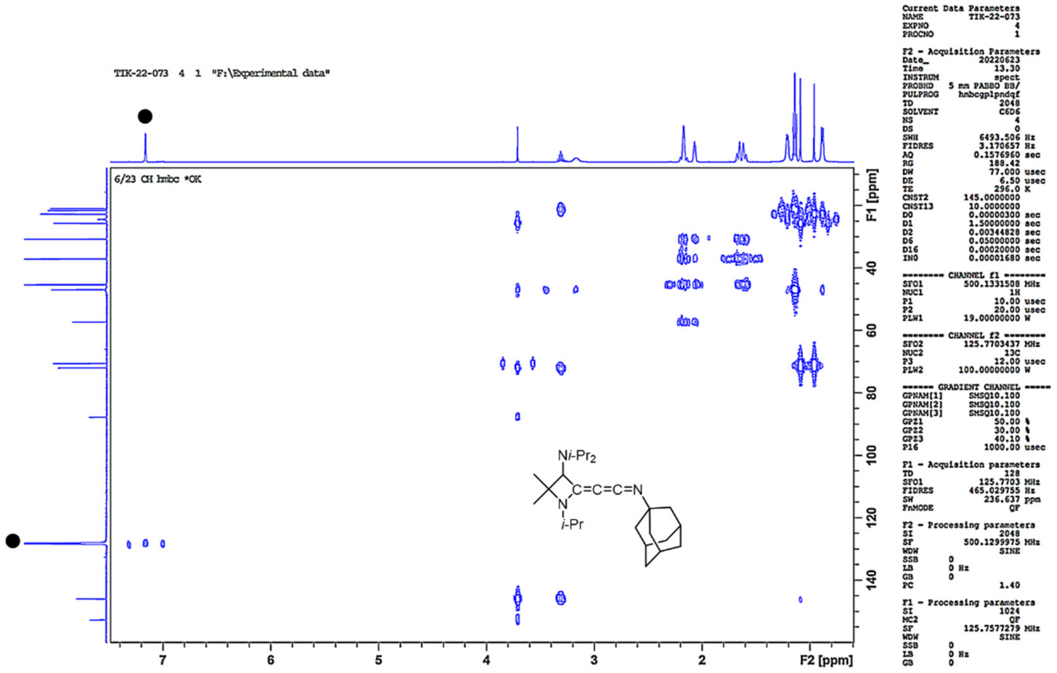

Figure S14. ^{1}H-^{13}C HMBC NMR spectrum of $\mathbf{1_{Ad}}$ in C_6D_6 at 296 K (● = C_6D_5H & C_6D_6).

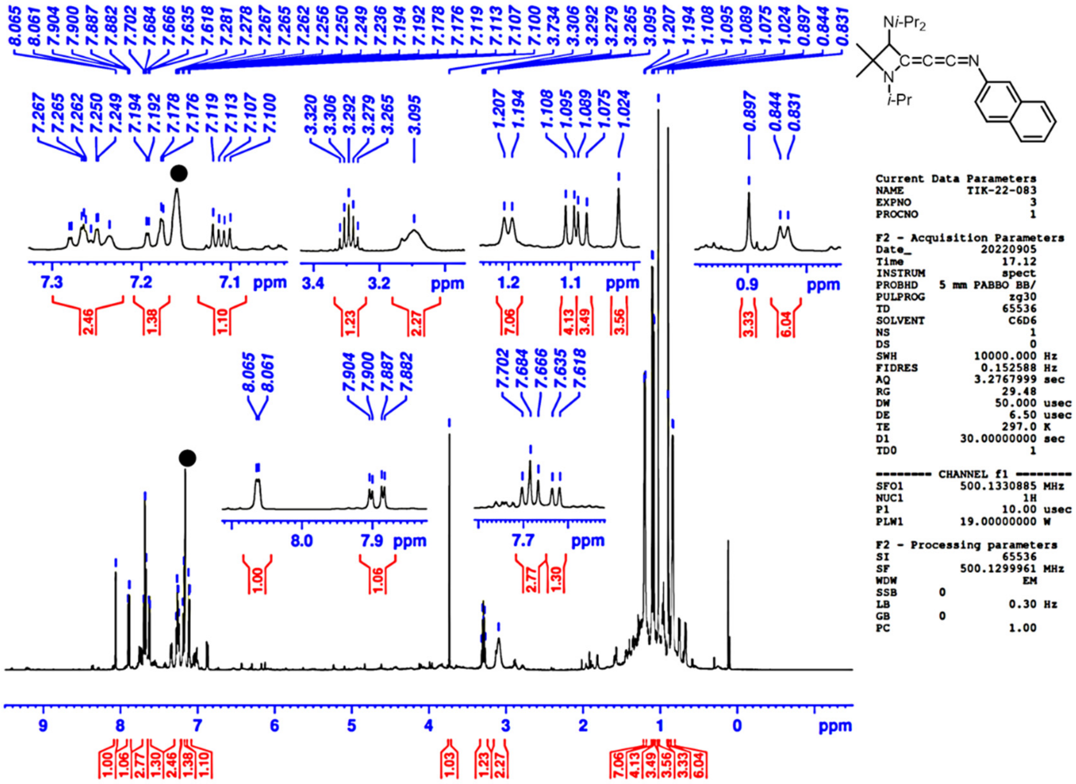

Figure S15. ^{1}H NMR spectrum of crude $\mathbf{1_{Naph}}$ in C_6D at 297 K (● = C_6D_5H).

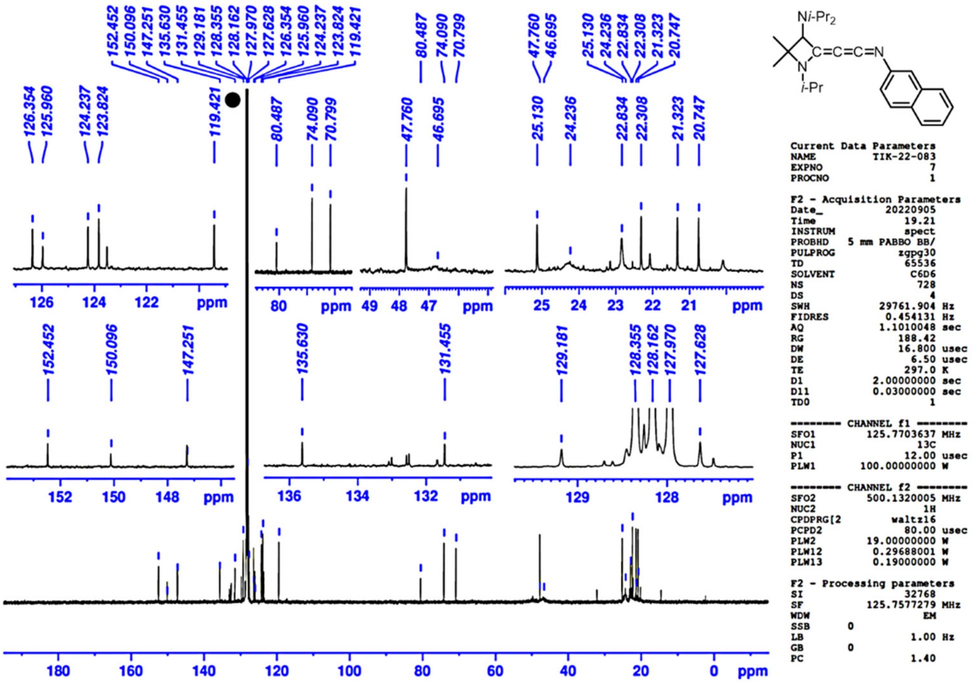

Figure S16. ^{13}C{^{1}H} NMR spectrum of crude $\mathbf{1_{Naph}}$ in C_6D at 297 K (● = C_6D_6).

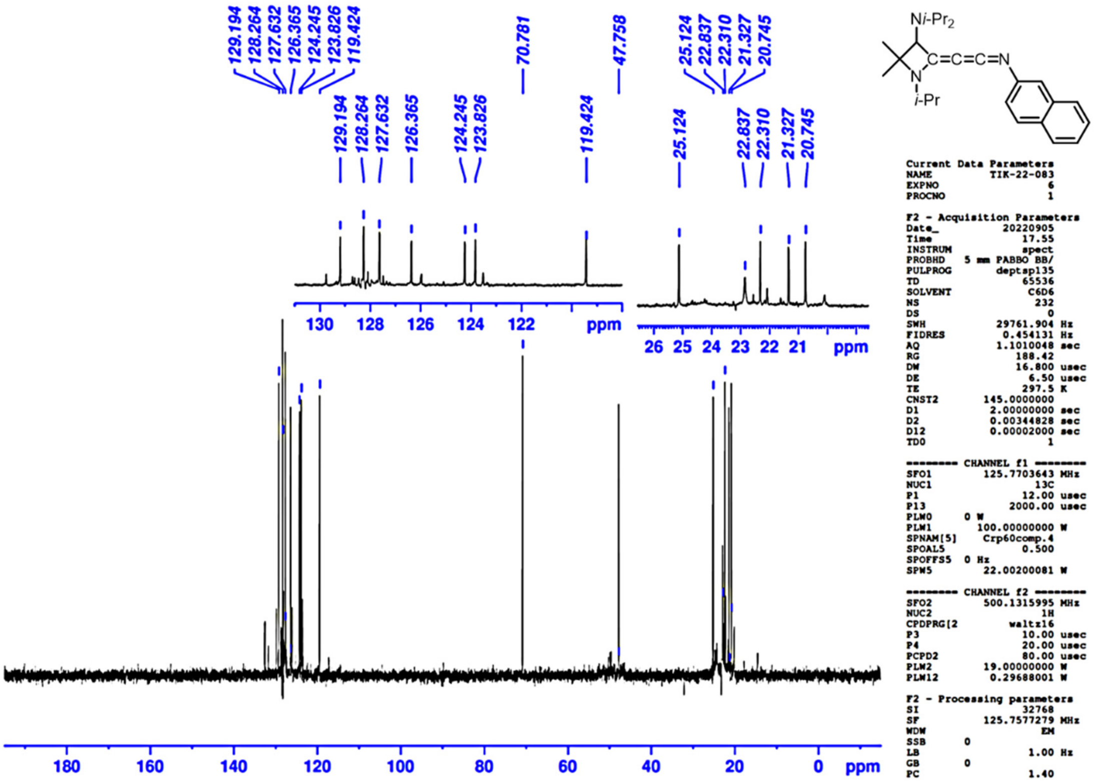

Figure S17. $^{13}C\{^{1}H\}$ NMR (DEPT135) spectrum of crude $\mathbf{1_{Naph}}$ in C_6D_6 at 298 K.

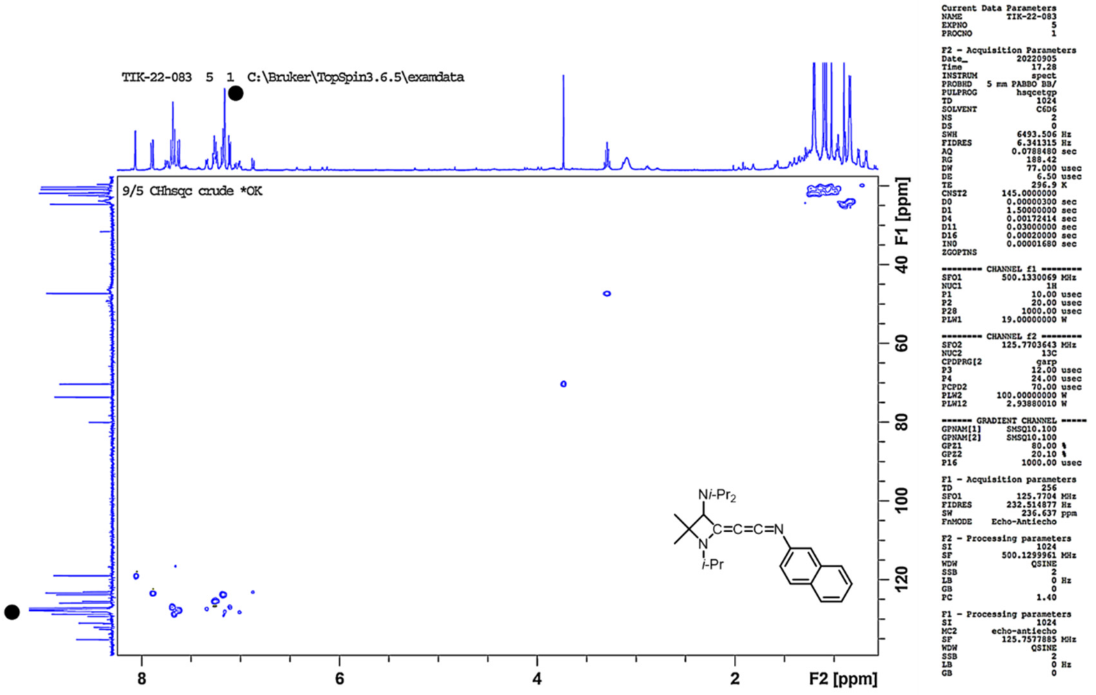

Figure S18. ^{1}H-^{13}C HSQC NMR spectrum of crude $\mathbf{1_{Naph}}$ in C_6D_6 at 297 K (● = C_6D_5H & C_6D_6).

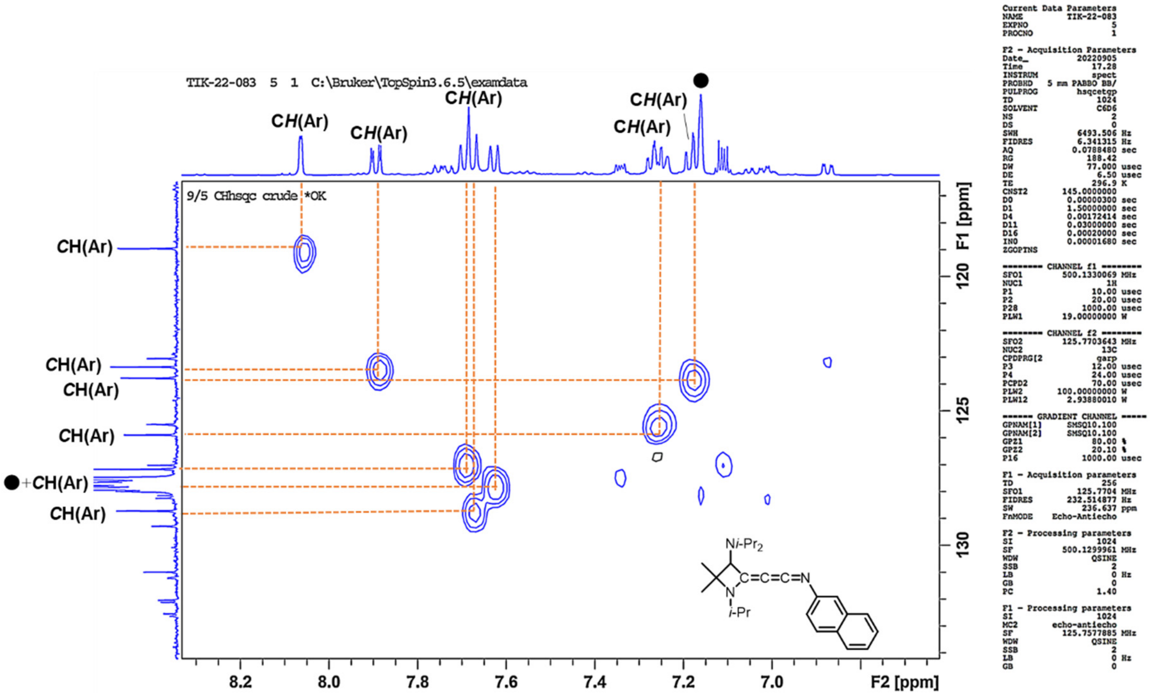

Figure S19. ^{1}H-^{13}C HSQC (magnified) NMR spectrum of crude $\mathbf{1}_{\mathbf{Naph}}$ in C_6D_6 at 297 K (● = C_6D_5H & C_6D_6).

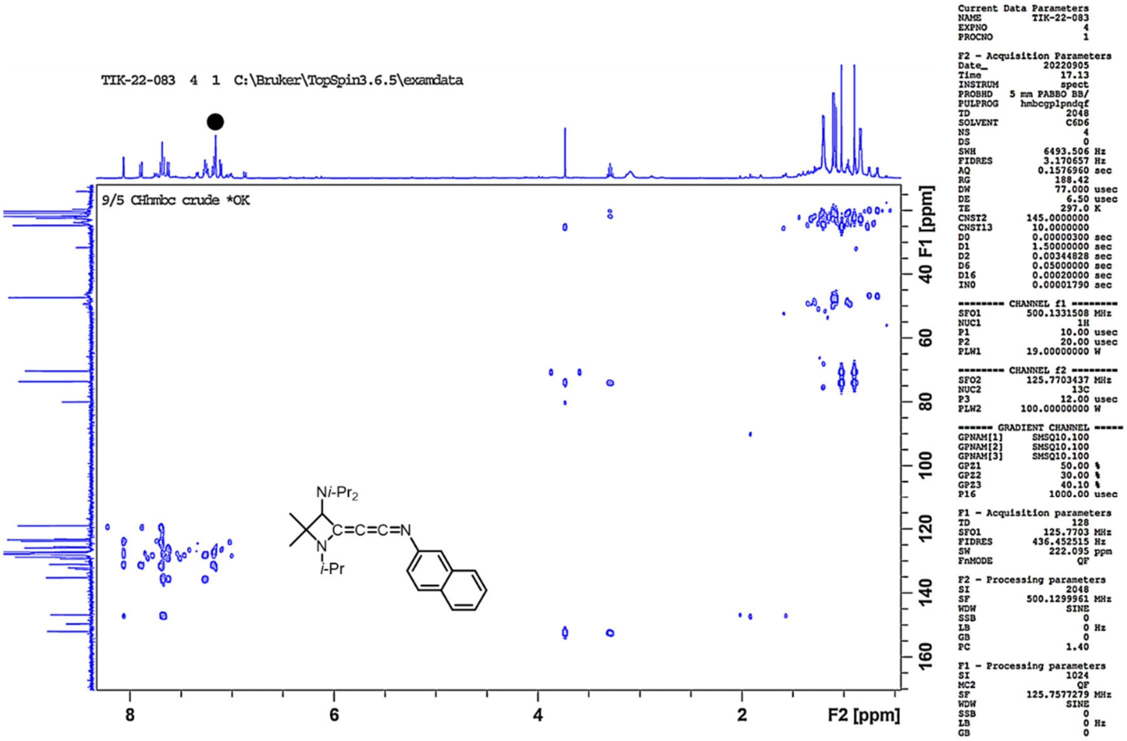

Figure S20. ^{1}H-^{13}C HMBC NMR spectrum of crude $\mathbf{1}_{\mathbf{Naph}}$ in C_6D_6 at 297 K (● = C_6D_5H).

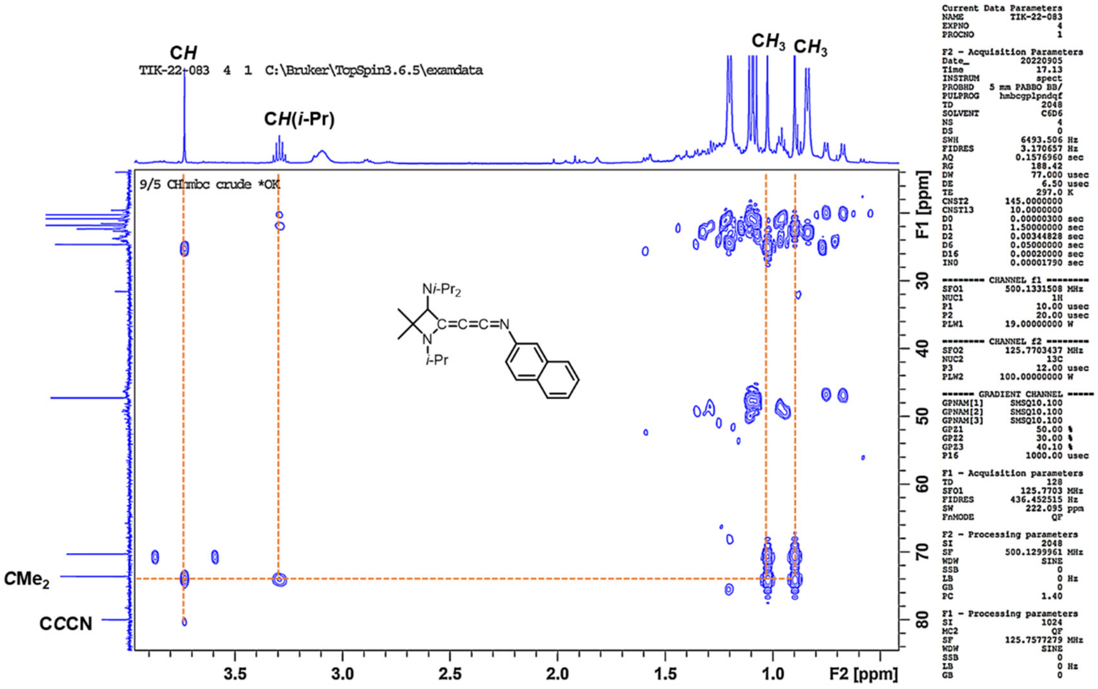

Figure S21. ^{1}H-^{13}C HMBC (magnified) NMR spectrum of crude $\mathbf{1}_{\mathbf{Naph}}$ in C_6D_6 at 297 K.

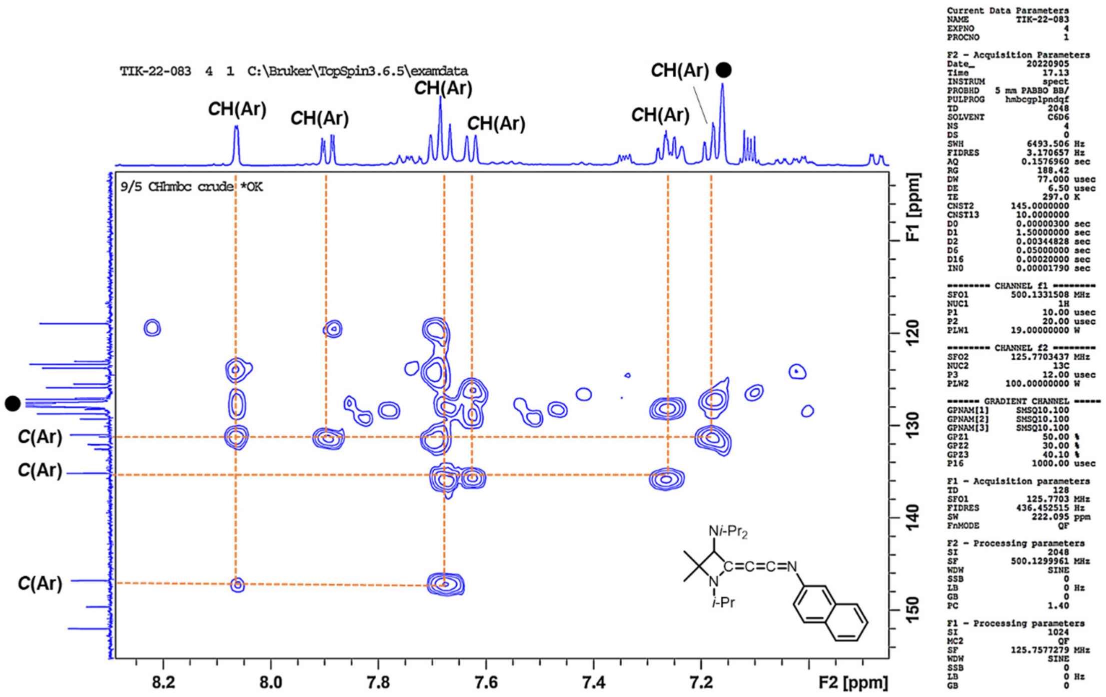

Figure S22. ^{1}H-^{13}C HMBC (magnified) NMR spectrum of crude $\mathbf{1}_{\mathbf{Naph}}$ in C_6D_6 at 297 K (● = C_6D_5H & C_6D_6).

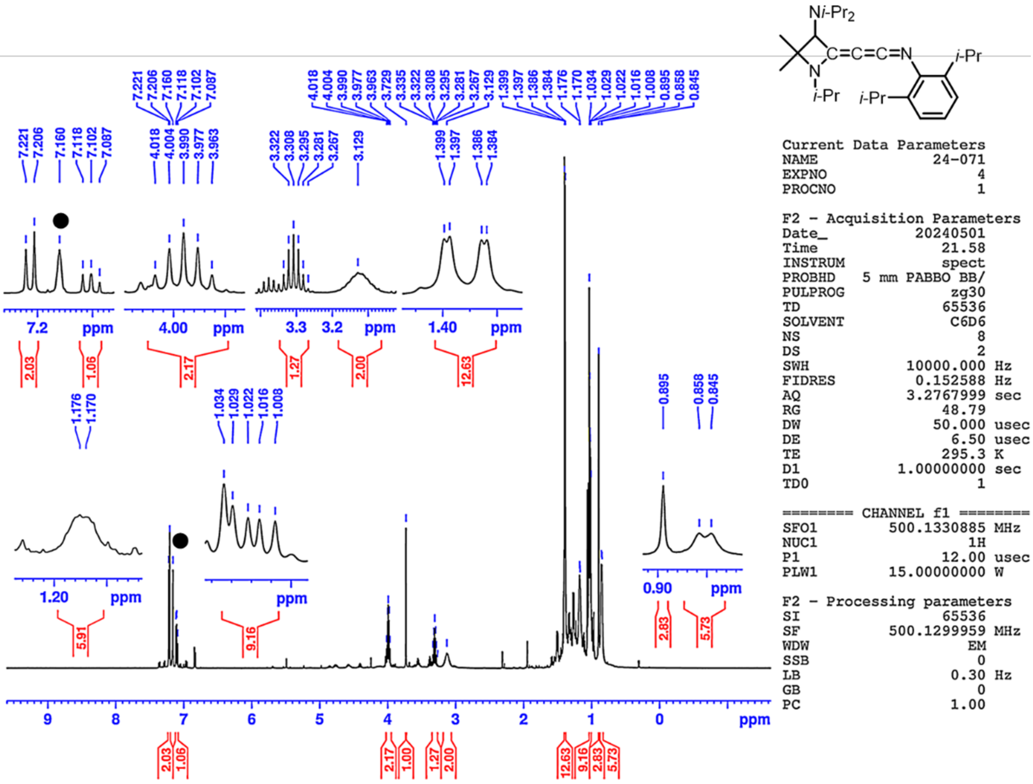

Figure S23. ^{1}H NMR spectrum of crude $\mathbf{1}_{\mathbf{Dipp}}$ in C_6D_6 at 295 K (● = C_6D_5H).

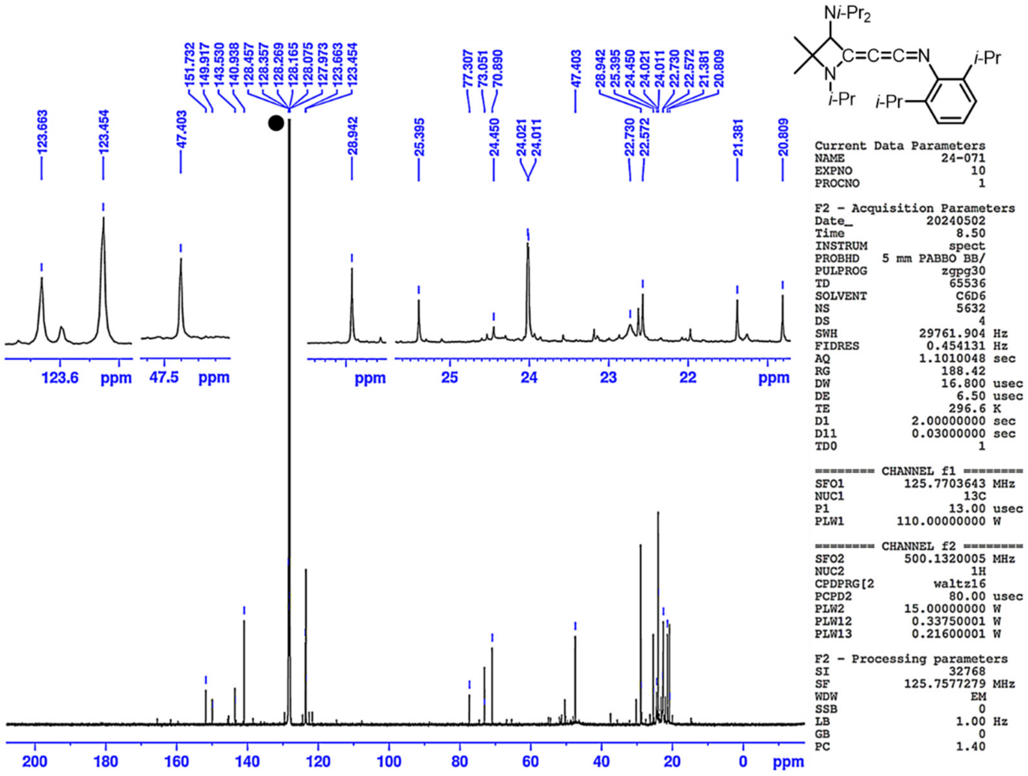

Figure S24. ^{13}C{^{1}H} NMR spectrum of crude $\mathbf{1}_{\mathbf{Dipp}}$ in C_6D_6 at 297 K (● = C_6D_6).

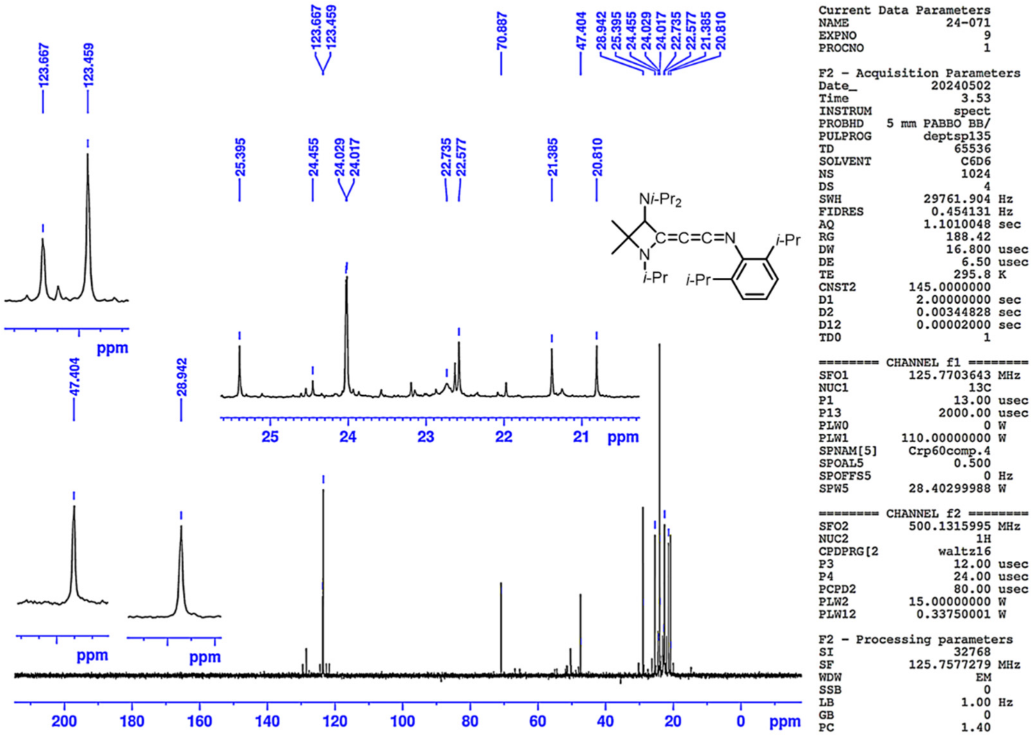

Figure S25. ^{13}C (DEPT135) NMR spectrum of crude $\mathbf{1}_{\mathbf{Dipp}}$ in C_6D_6 at 296 K.

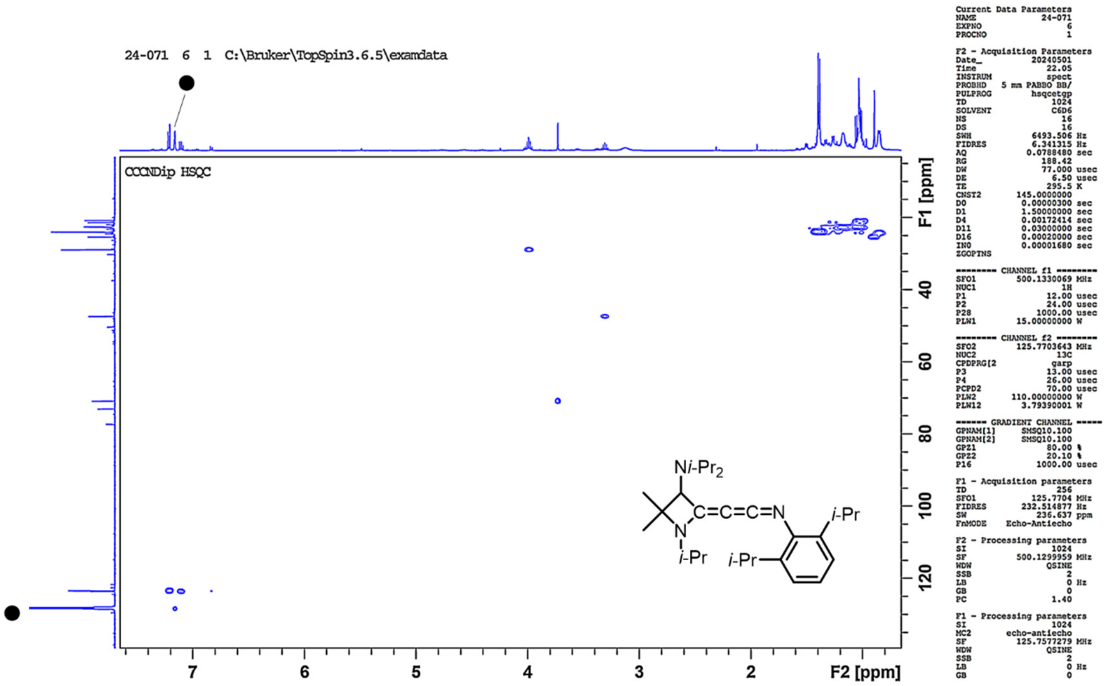

Figure S26. ^{1}H-^{13}C HSQC NMR spectrum of crude $\mathbf{1}_{\mathbf{Dipp}}$ in C_6D_6 at 296 K (● = C_6D_5H & C_6D_6).

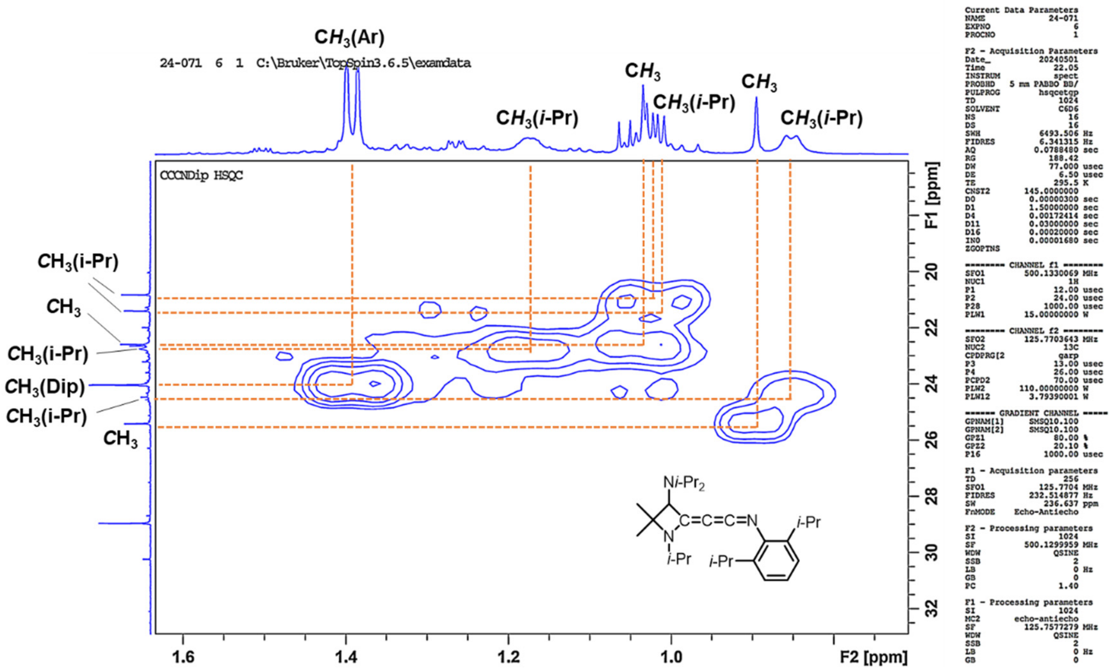

Figure S27. ^{1}H-^{13}C HSQC (magnified) NMR spectrum of crude $\mathbf{1}_{\mathbf{Dipp}}$ in C_6D_6 at 296 K.

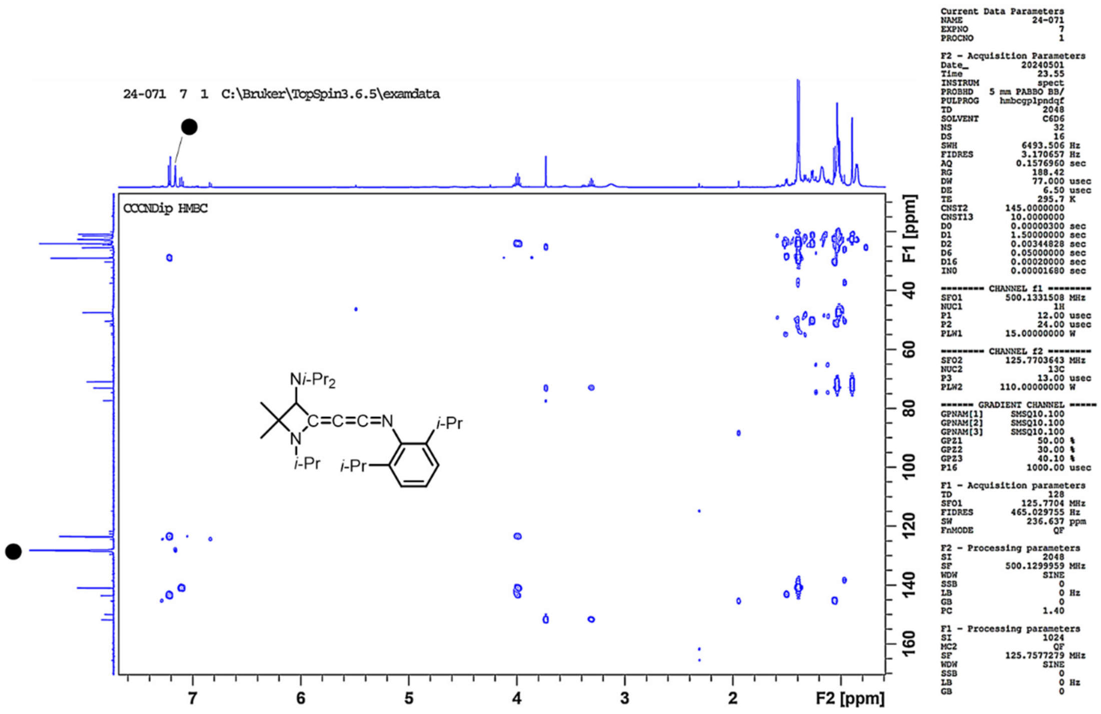

Figure S28. ^{1}H-^{13}C HMBC NMR spectrum of crude $\mathbf{1}_{\mathbf{Dipp}}$ in C_6D_6 at 296 K (● = C_6D_5H & C_6D_6).

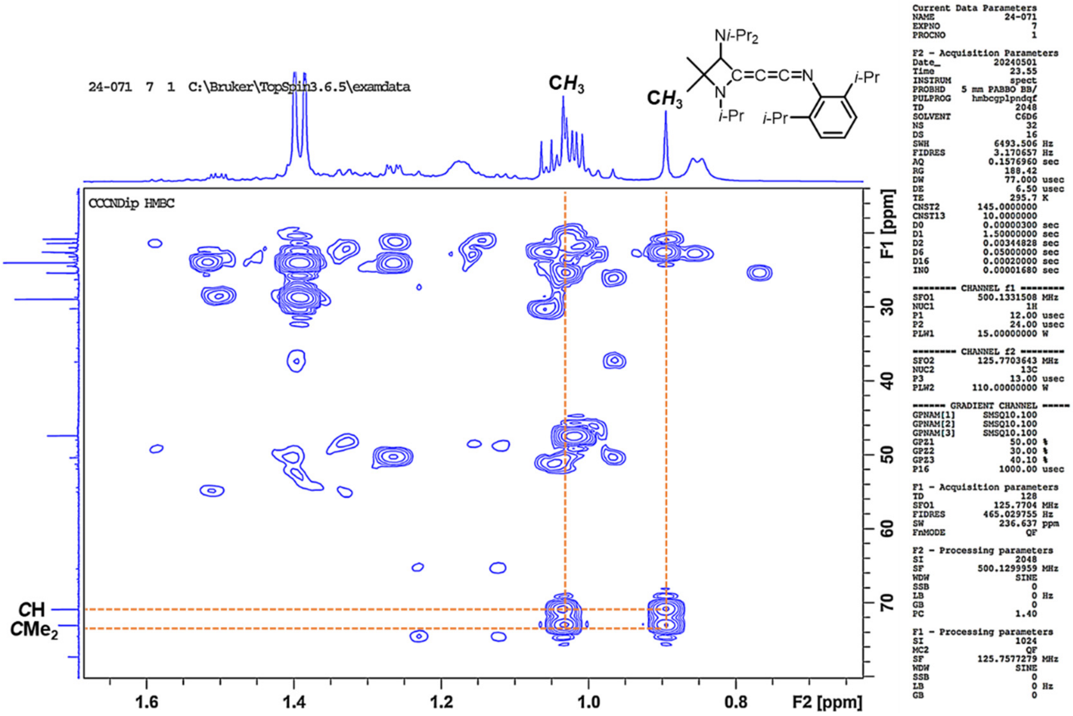

Figure S29. ^{1}H-^{13}C HMBC (magnified) NMR spectrum of crude $\mathbf{1}_{\mathbf{Dipp}}$ in C_6D_6 at 296 K.

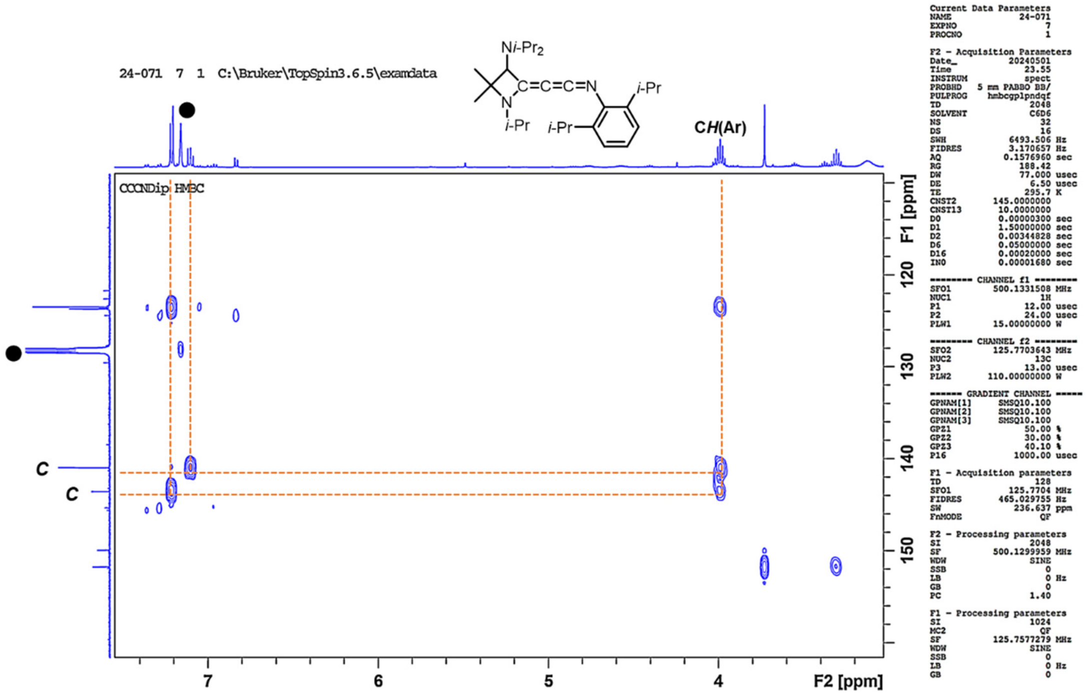

Figure S30. ^{1}H-^{13}C HMBC (magnified) NMR spectrum of crude $\mathbf{1}_{\mathbf{Dipp}}$ in C_6D_6 at 296 K (● = C_6D_5H & C_6D_6).

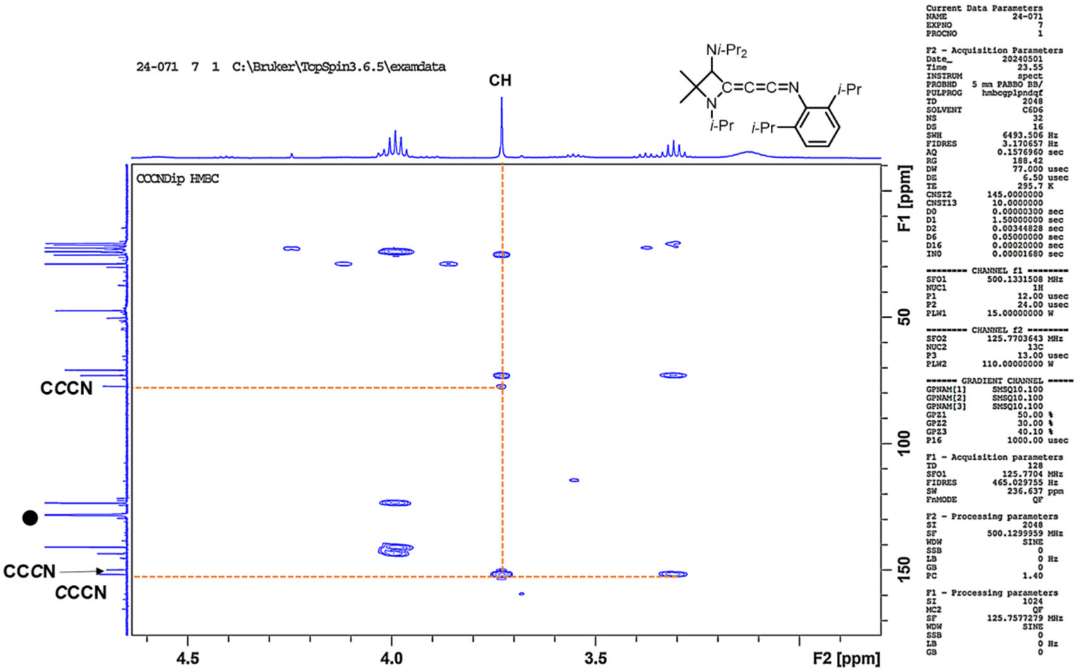

Figure S31. ^{1}H-^{13}C HMBC (magnified) NMR spectrum of crude **1**$_{\mathbf{Dipp}}$ in C_6D_6 at 296 K (● = C_6D_6).

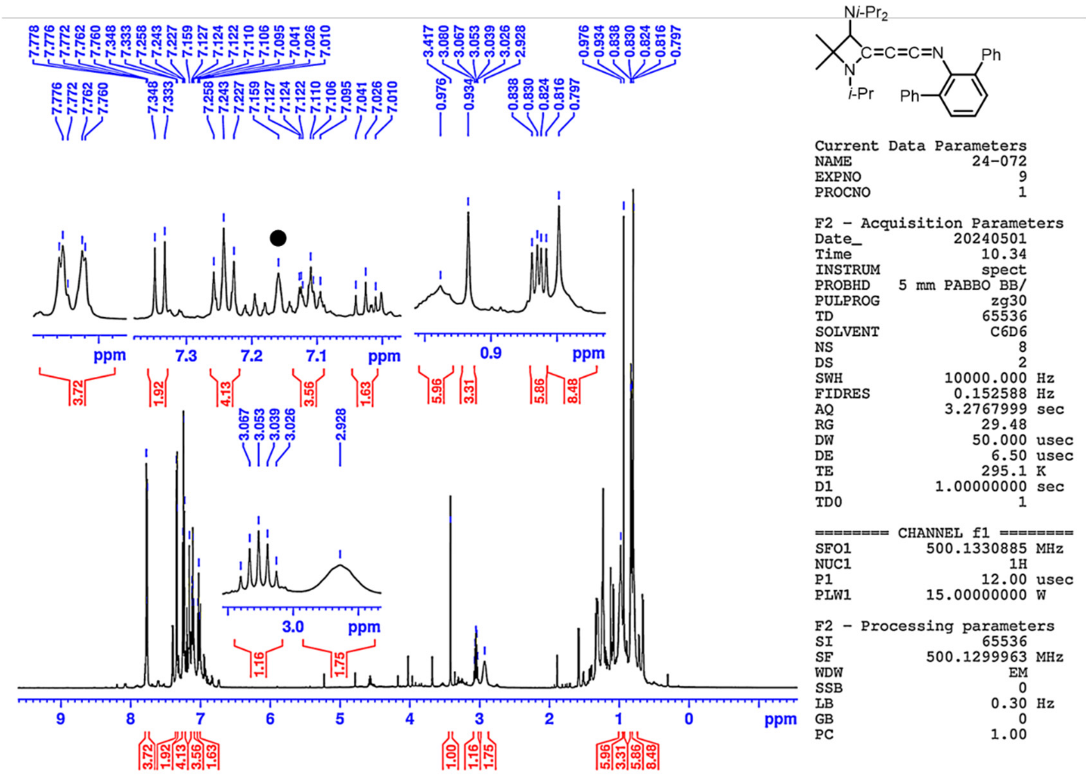

Figure S32. ^{1}H NMR spectrum of crude **1**$_{\mathbf{TerPh}}$ in C_6D_6 at 295 K (● = C_6D_5H).

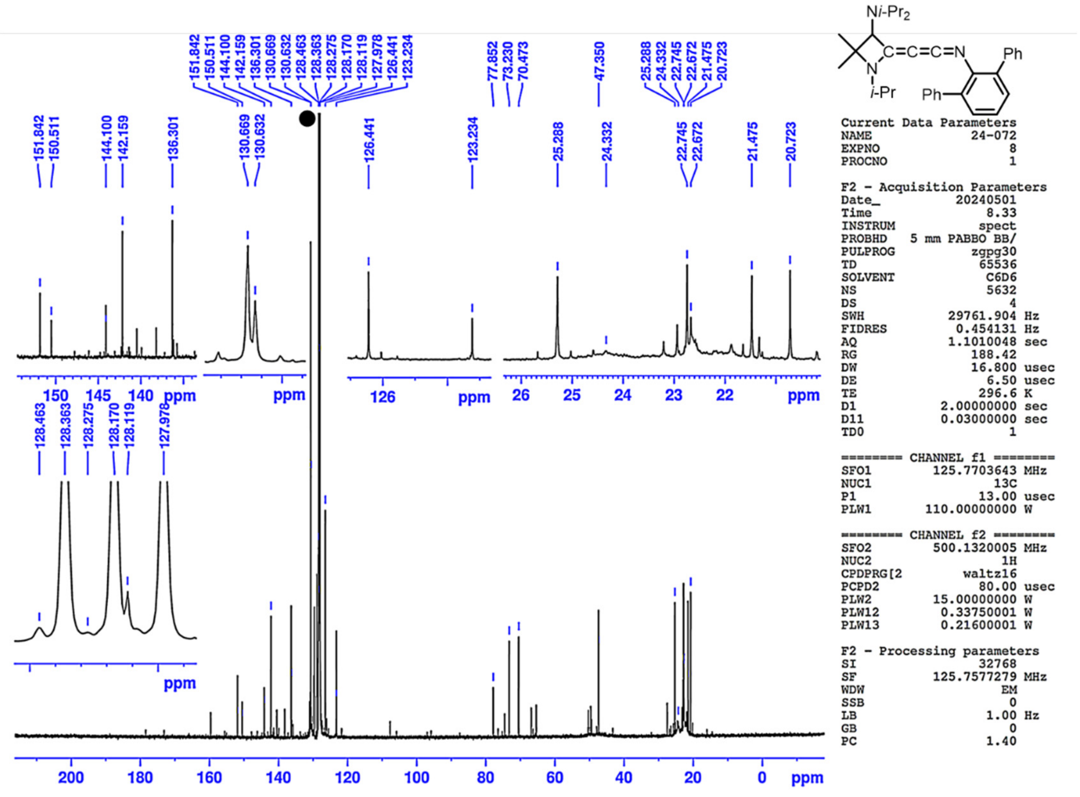

Figure S33. $^{13}C\{^1H\}$ NMR spectrum of crude $\mathbf{1}_{\mathbf{TerPh}}$ in C_6D_6 at 297 K (● = C_6D_6).

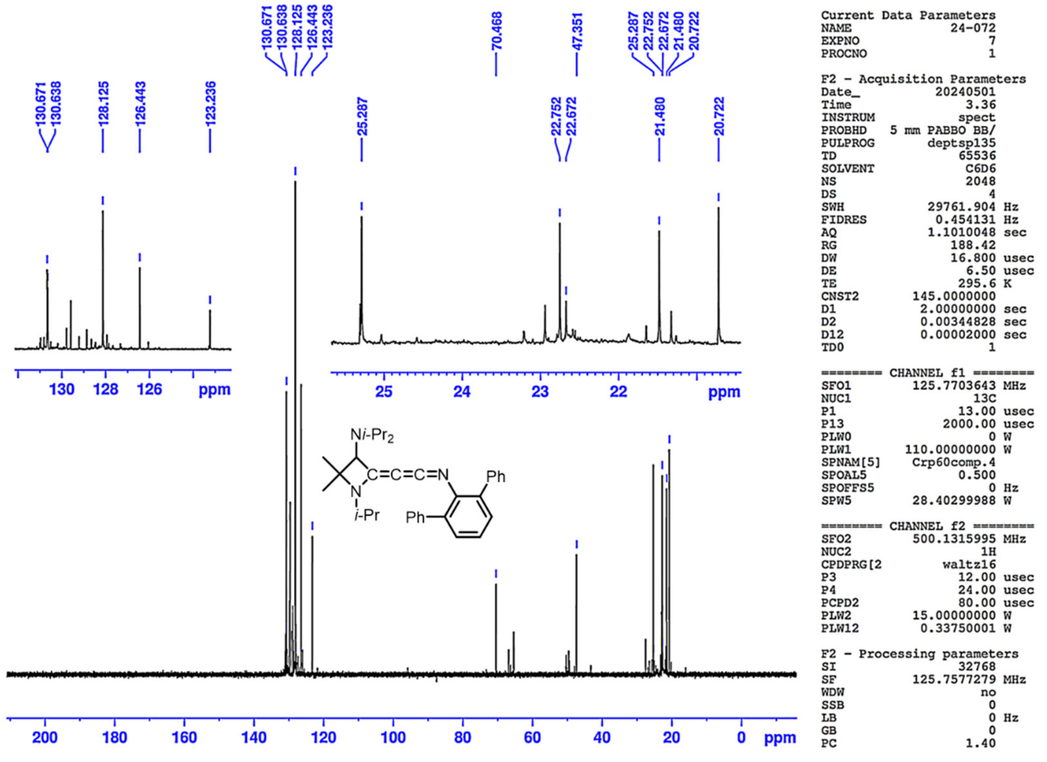

Figure S34. $^{13}C\{^1H\}$ (DEPT135) NMR spectrum of crude $\mathbf{1}_{\mathbf{TerPh}}$ in C_6D_6 at 296 K.

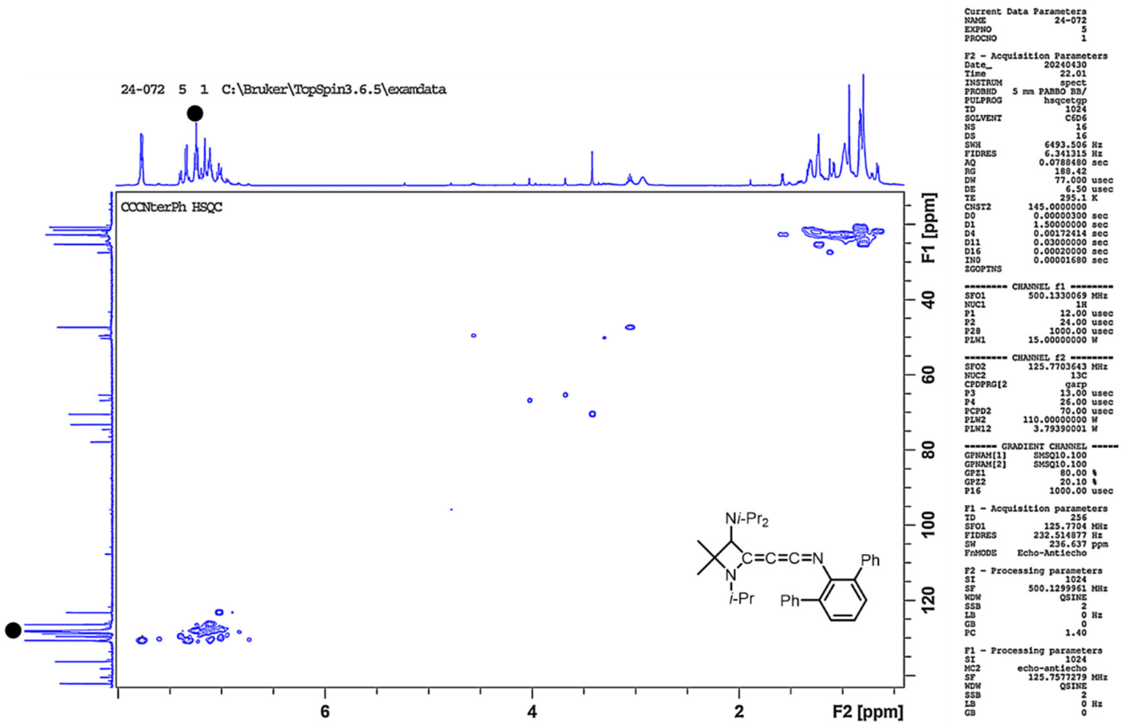

Figure S35. ^{1}H-^{13}C HSQC NMR spectrum of crude $\mathbf{1}_{\mathbf{TerPh}}$ in C_6D_6 at 295 K (● = C_6D_5H & C_6D_6).

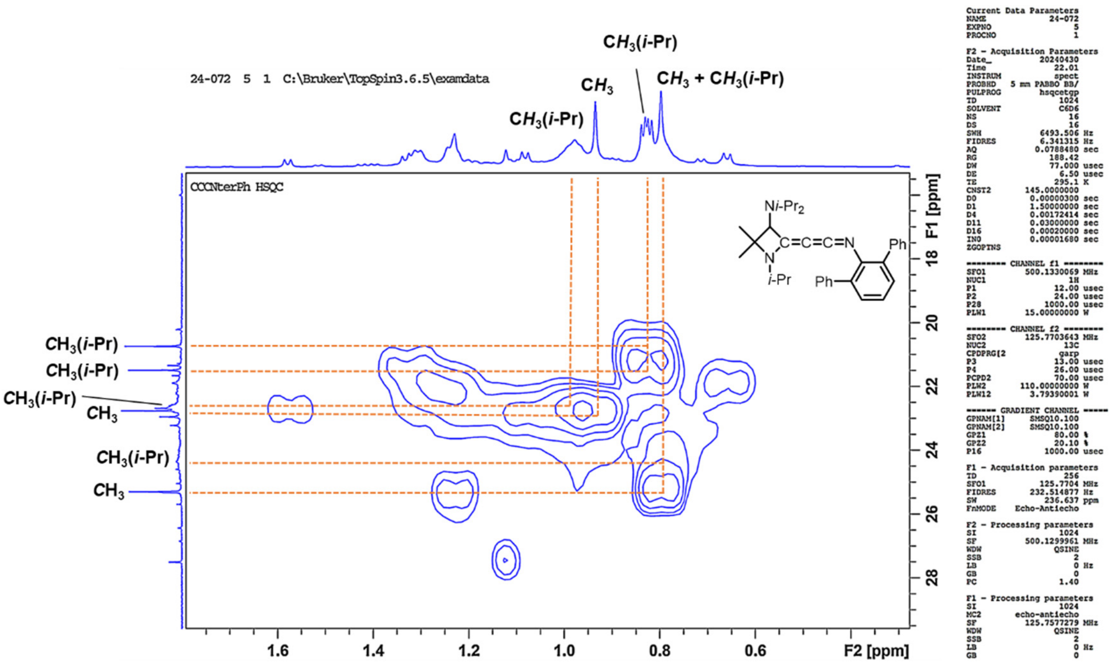

Figure S36. ^{1}H-^{13}C HSQC (magnified) NMR spectrum of crude $\mathbf{1}_{\mathbf{TerPh}}$ in C_6D_6 at 295 K.

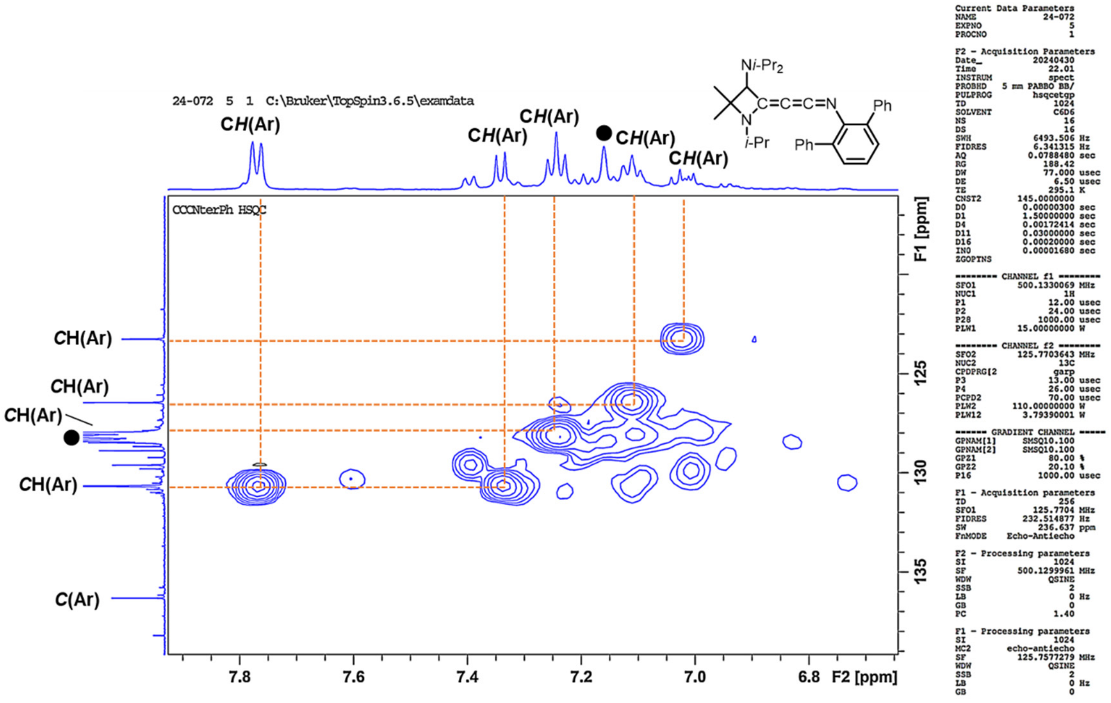

Figure S37. ^{1}H-^{13}C HSQC (magnified) NMR spectrum of crude $\mathbf{1}_{\mathbf{TerPh}}$ in C_6D_6 at 295 K (● = C_6D_5H & C_6D_6).

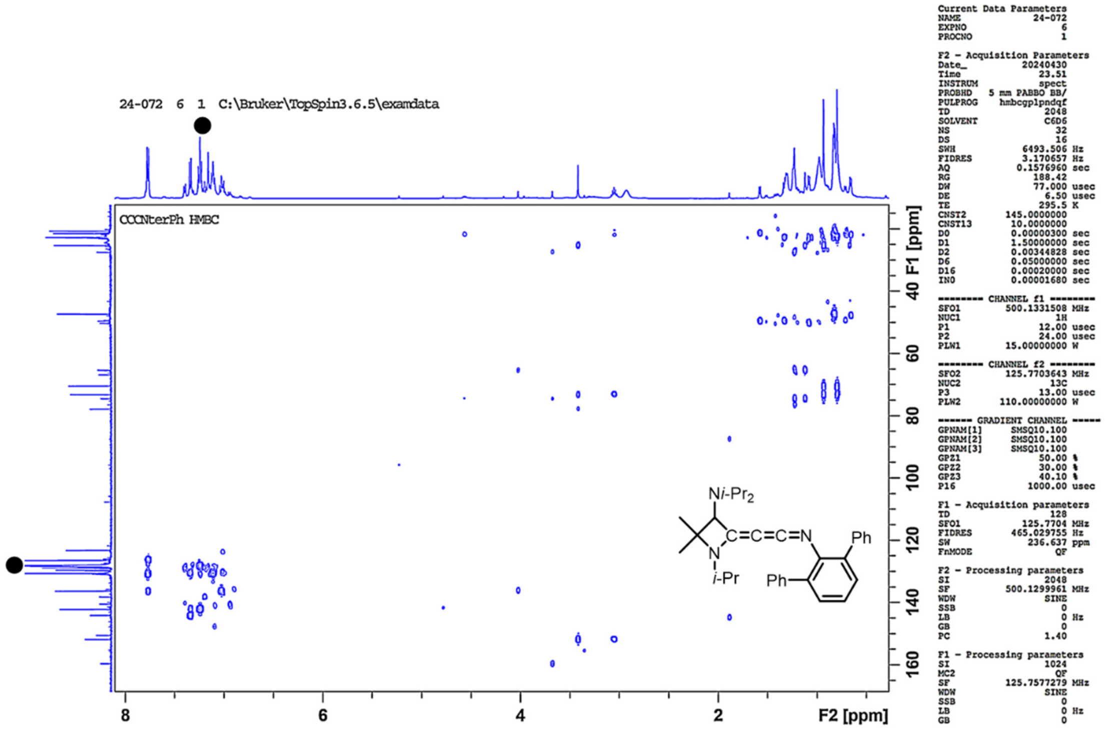

Figure S38. ^{1}H-^{13}C HMBC NMR spectrum of crude $\mathbf{1}_{\mathbf{TerPh}}$ in C_6D_6 at 296 K (● = C_6D_5H & C_6D_6).

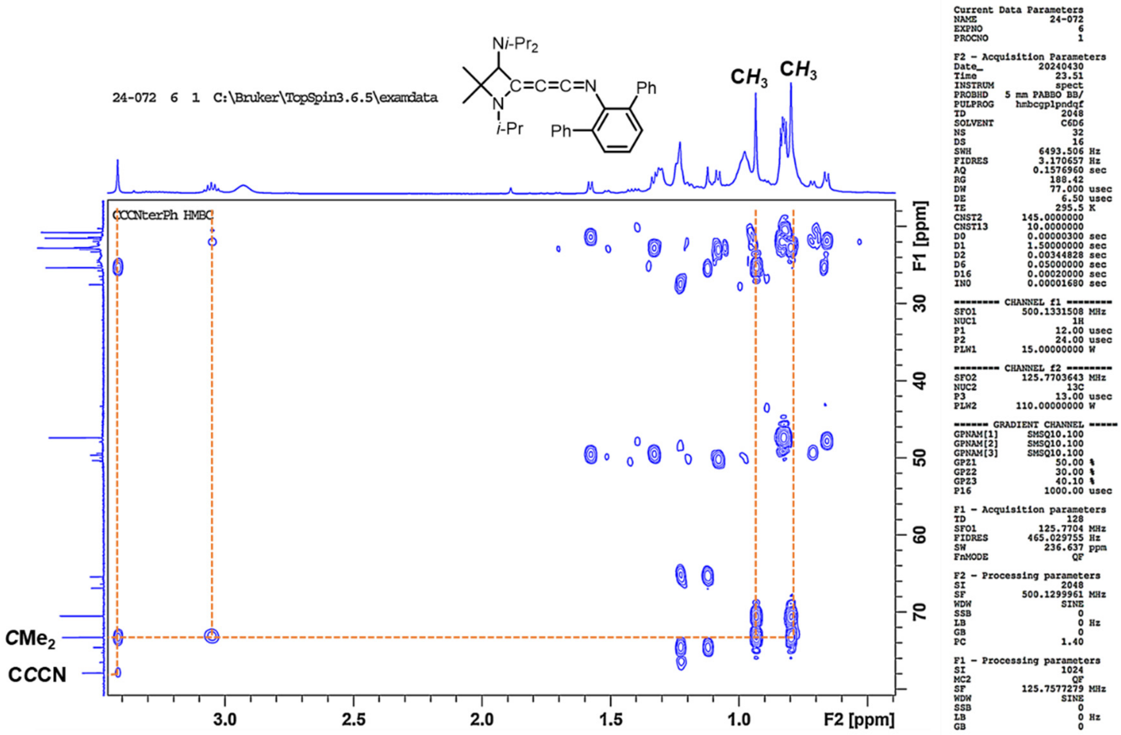

Figure S39. ^{1}H-^{13}C HMBC (magnified) NMR spectrum of crude $\mathbf{1}_{\mathbf{TerPh}}$ in C_6D_6 at 296 K.

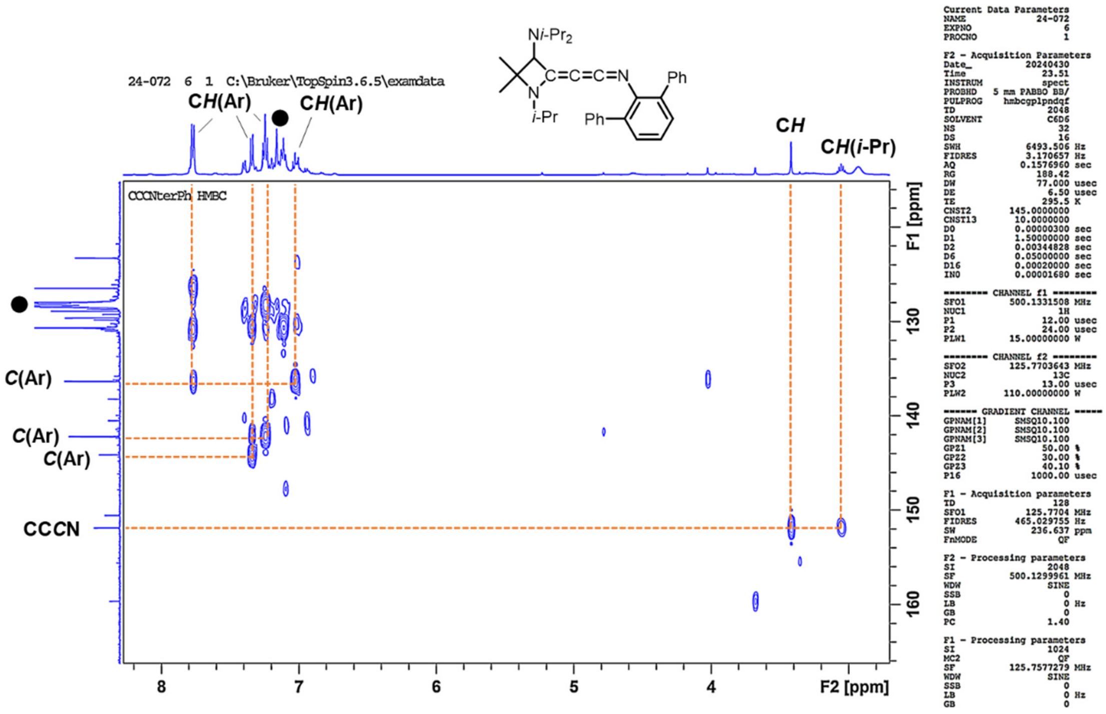

Figure S40. ^{1}H-^{13}C HMBC (magnified) NMR spectrum of crude $\mathbf{1}_{\mathbf{TerPh}}$ in C_6D_6 at 296 K (● = C_6D_5H & C_6D_6).

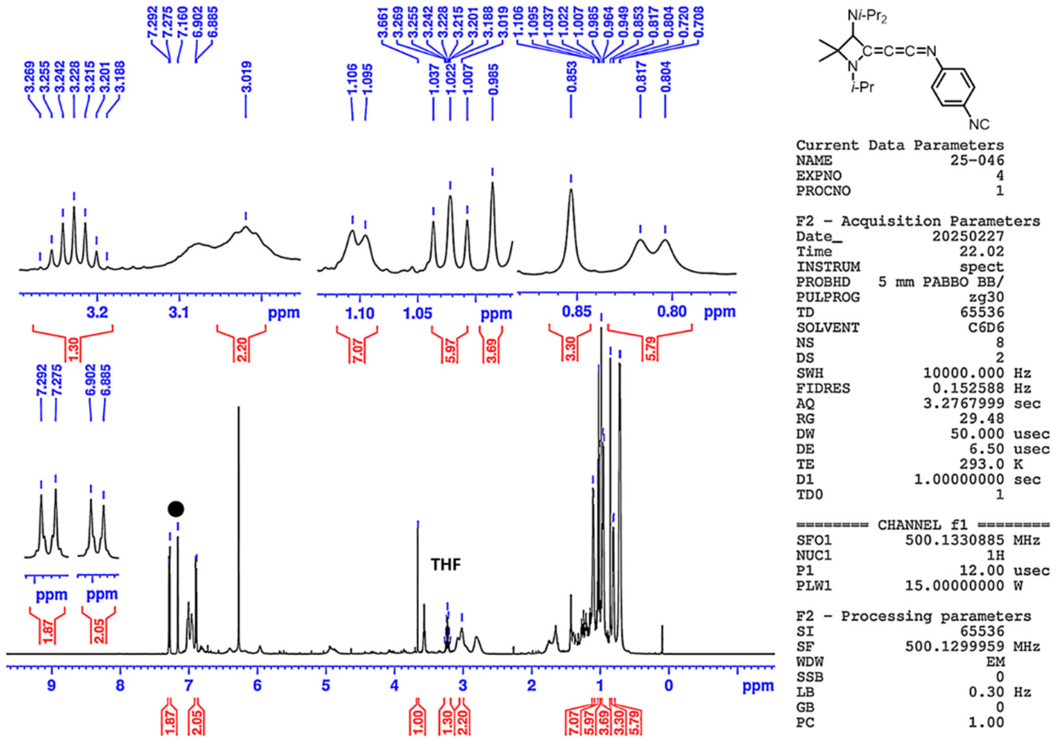

Figure S41. ^{1}H NMR spectrum of crude $\mathbf{1}_{\mathbf{PhNC}}$ in C_6D_6 at 293 K (● = C_6D_6).

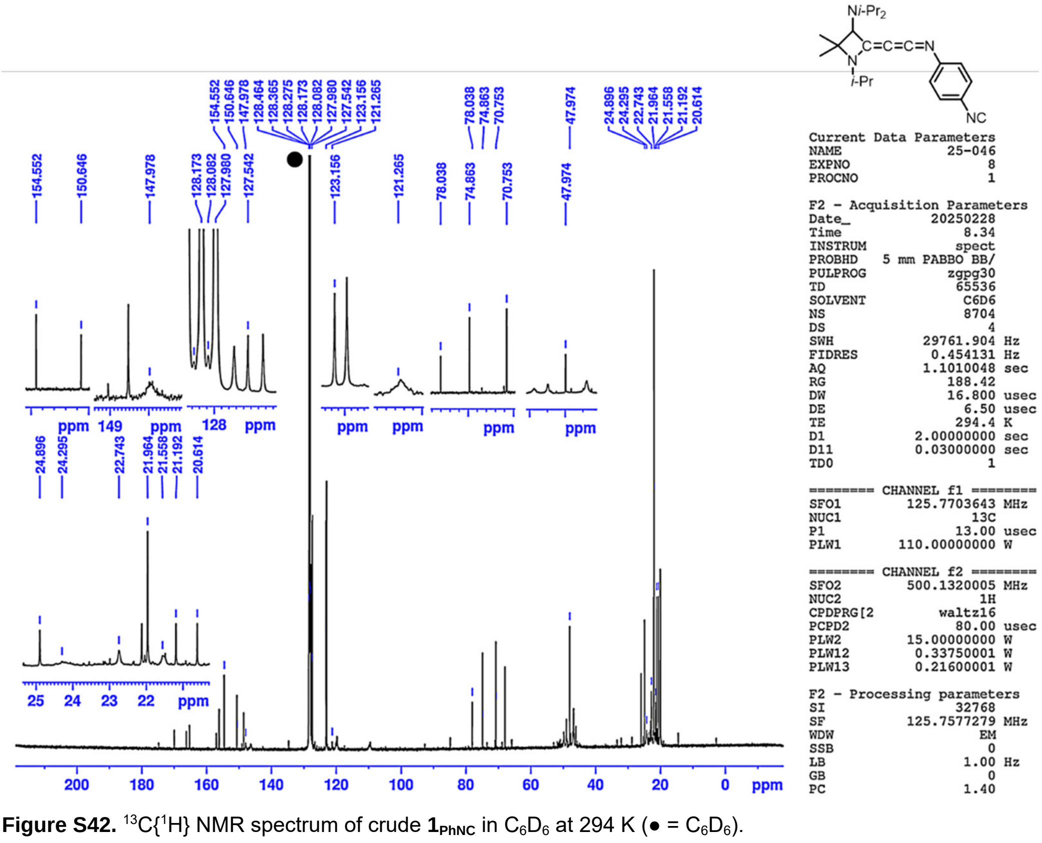

Figure S42. $^{13}C\{^{1}H\}$ NMR spectrum of crude $\mathbf{1}_{\mathbf{PhNC}}$ in C_6D_6 at 294 K (● = C_6D_6).

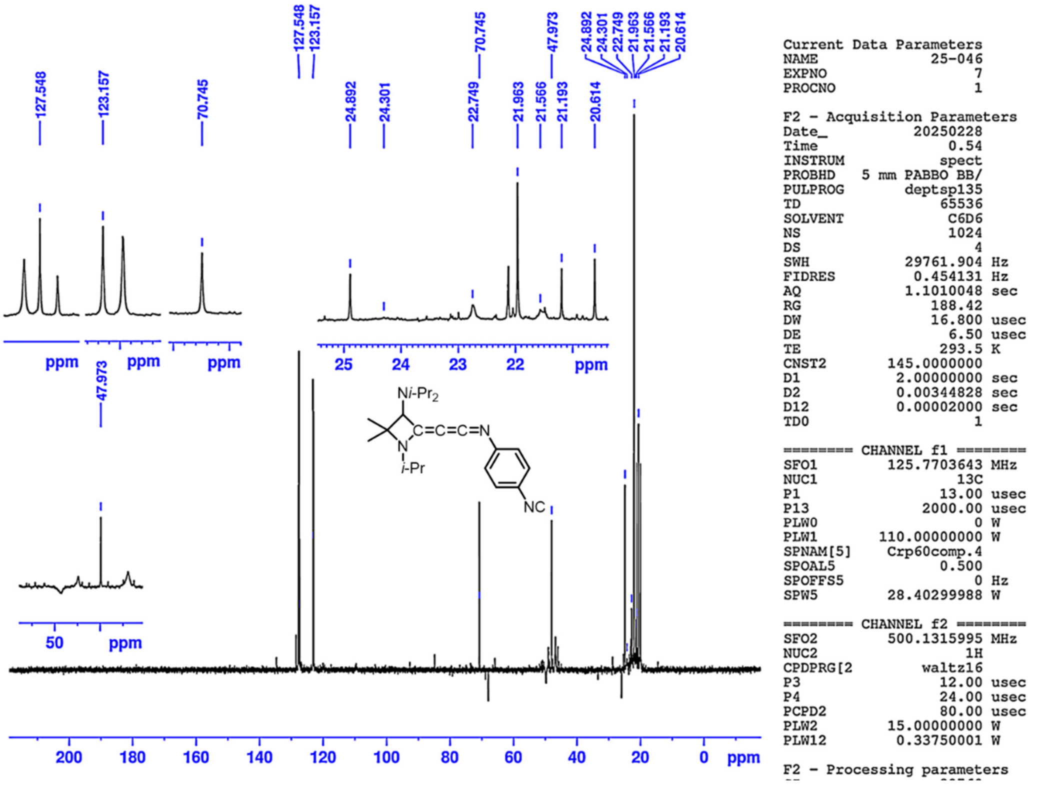

Figure S43. $^{13}C\{^{1}H\}$(DEPT 135) NMR spectrum of crude $\mathbf{1}_{\mathbf{PhNC}}$ in C_6D_6 at 294 K.

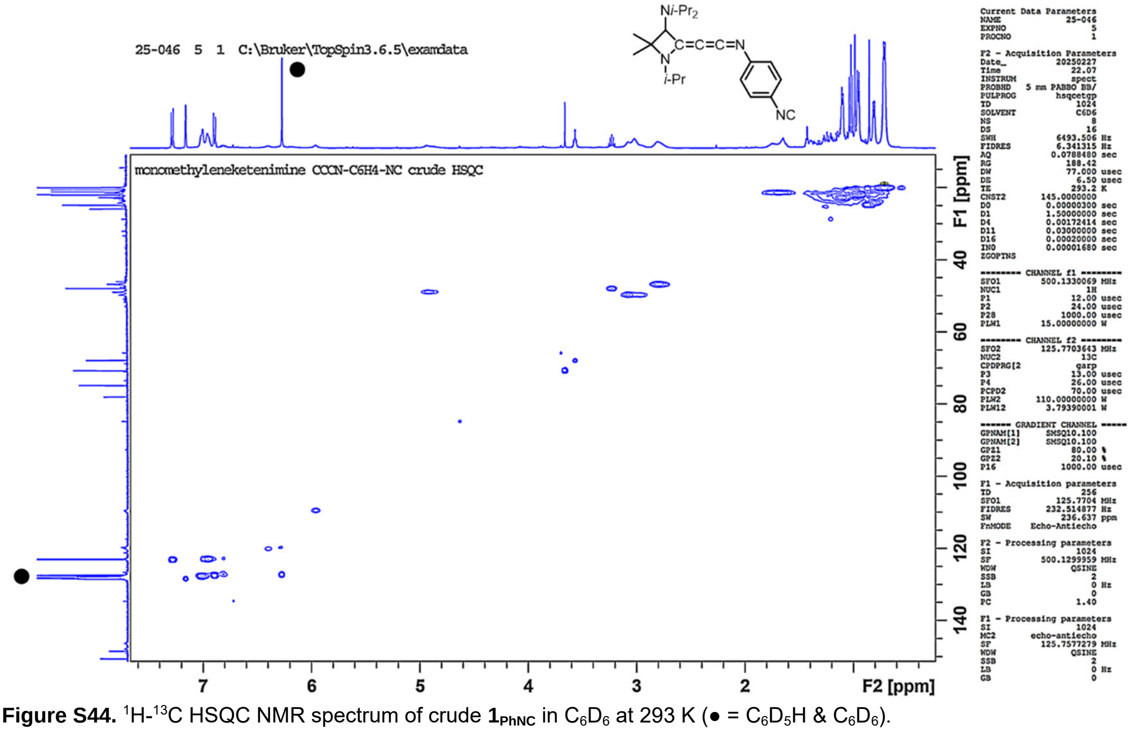

Figure S44. ^{1}H-^{13}C HSQC NMR spectrum of crude $\mathbf{1}_{\mathbf{PhNC}}$ in C_6D_6 at 293 K (● = C_6D_5H & C_6D_6).

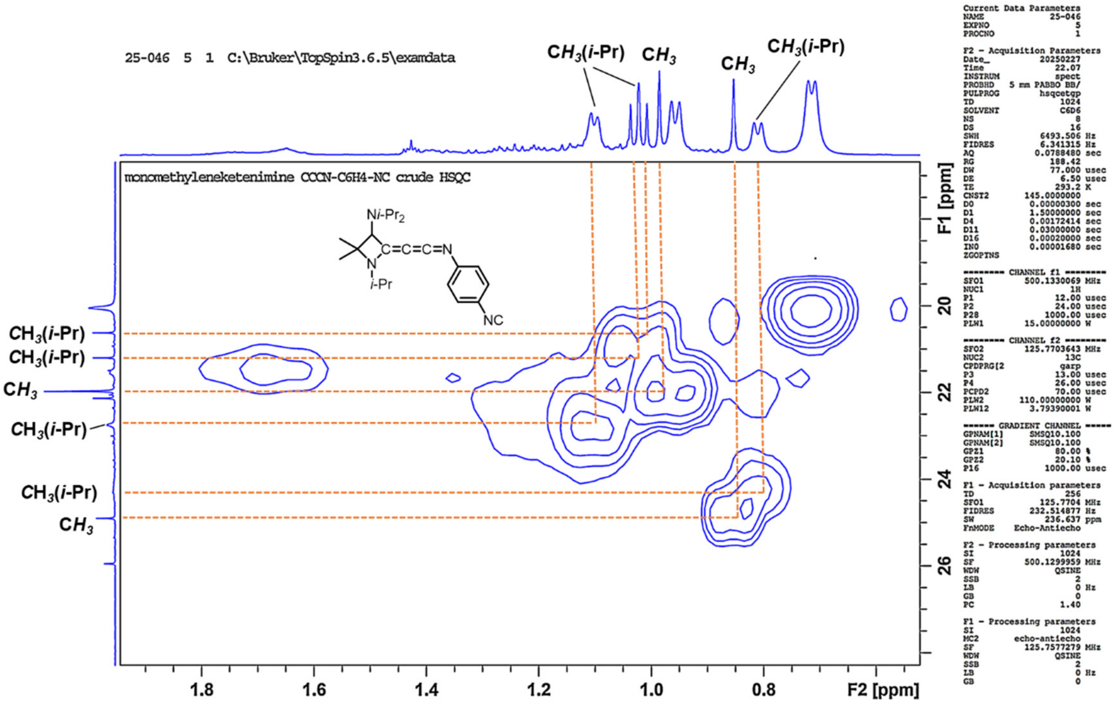

Figure S45. ^{1}H-^{13}C HSQC (magnified) NMR spectrum of crude $\mathbf{1}_{\mathbf{PhNC}}$ in C_6D_6 at 293 K.

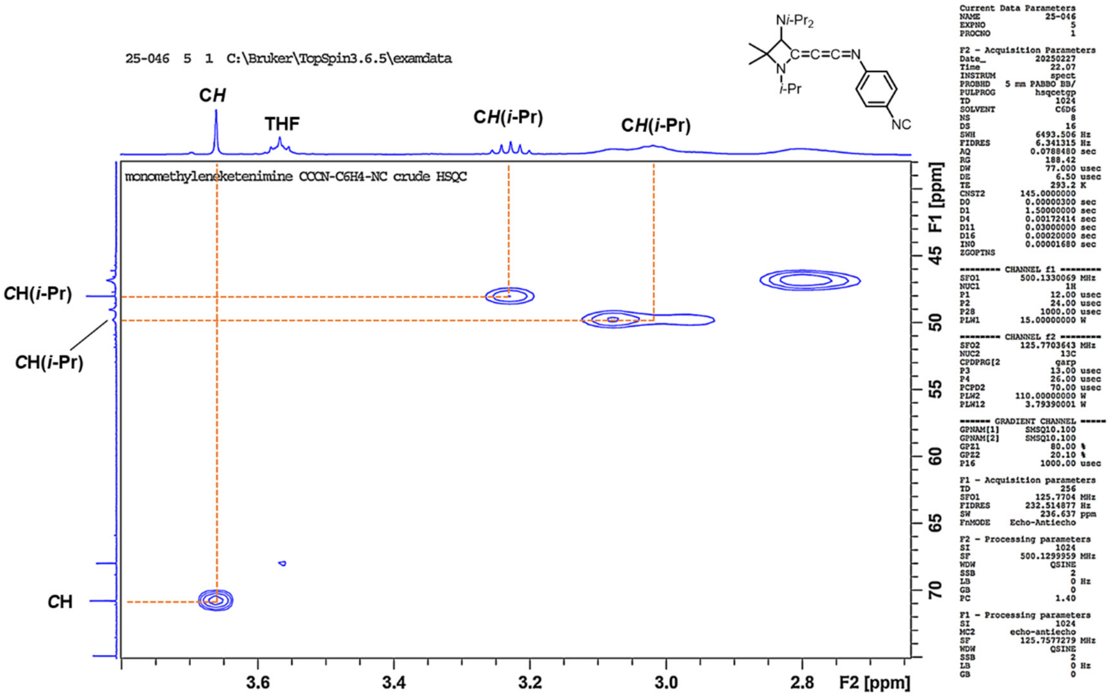

Figure S46. ^{1}H-^{13}C HSQC (magnified) NMR spectrum of crude $\mathbf{1}_{\mathbf{PhNC}}$ in C_6D_6 at 293 K.

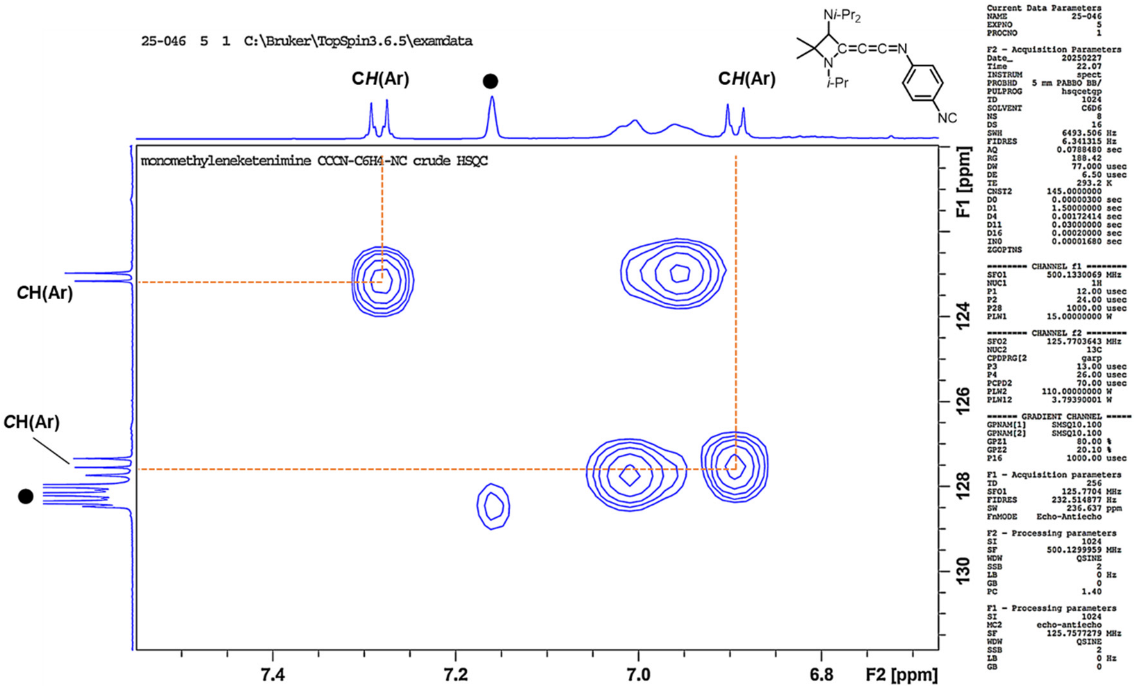

Figure S47. ^{1}H-^{13}C HSQC (magnified) NMR spectrum of crude $\mathbf{1}_{\mathbf{PhNC}}$ in C_6D_6 at 293 K (● = C_6D_5H & C_6D_6).

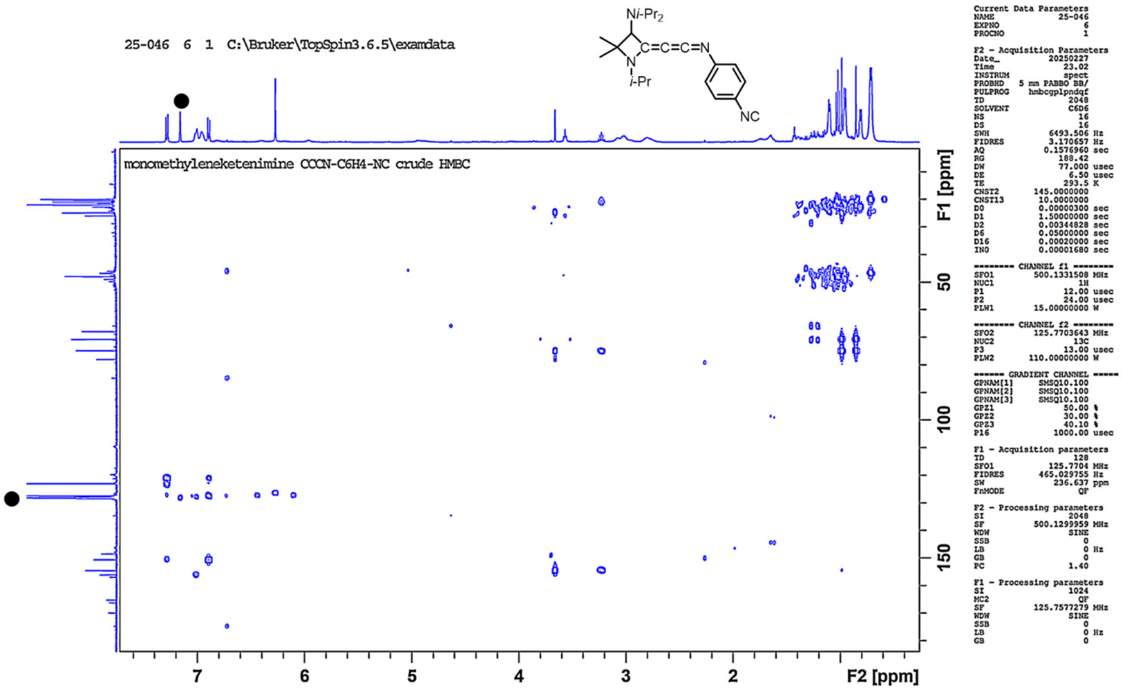

Figure S48. ^{1}H-^{13}C HMBC NMR spectrum of crude $\mathbf{1}_{\mathbf{PhNC}}$ in C_6D_6 at 294 K (● = C_6D_5H & C_6D_6).

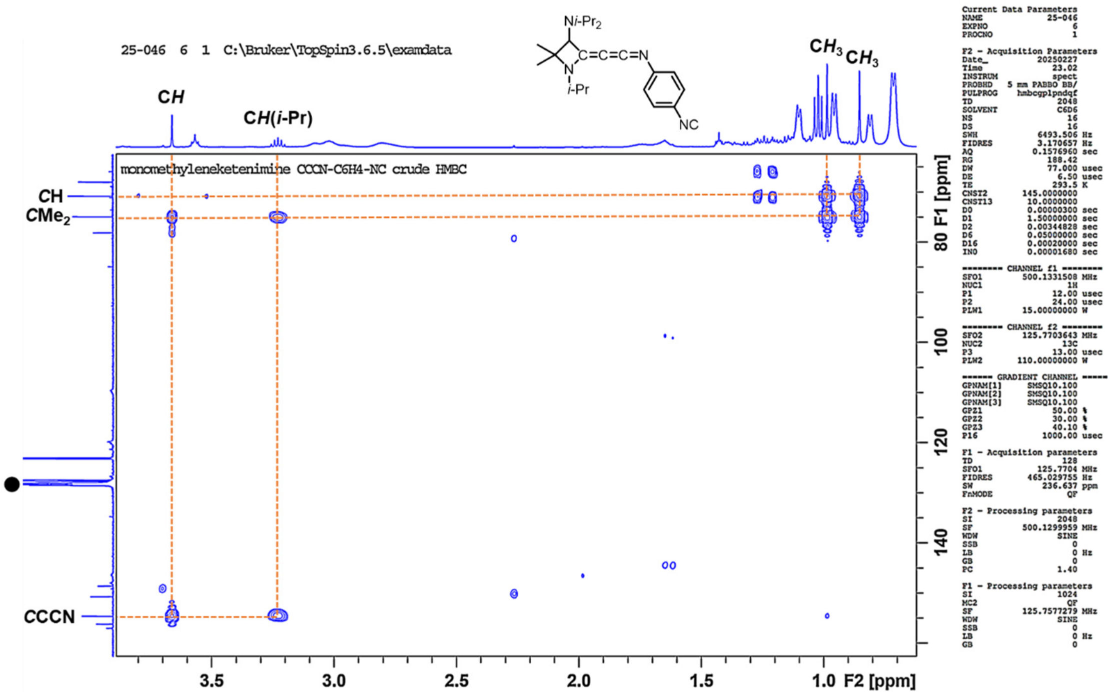

Figure S49. ^{1}H-^{13}C HMBC (magnified) NMR spectrum of crude $\mathbf{1}_{\mathbf{PhNC}}$ in C_6D_6 at 294 K (● = C_6D_6).

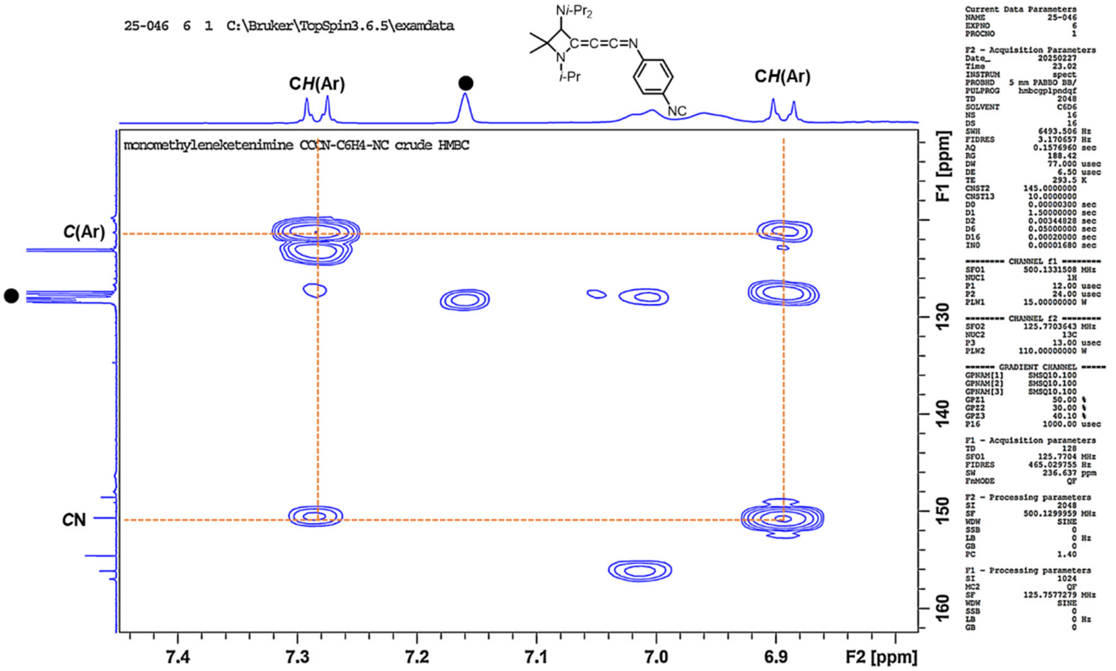

Figure S50. ^{1}H-^{13}C HMBC (magnified) NMR spectrum of crude $\mathbf{1}_{\mathbf{PhNC}}$ in C_6D_6 at 294 K (● = C_6D_5H & C_6D_6).

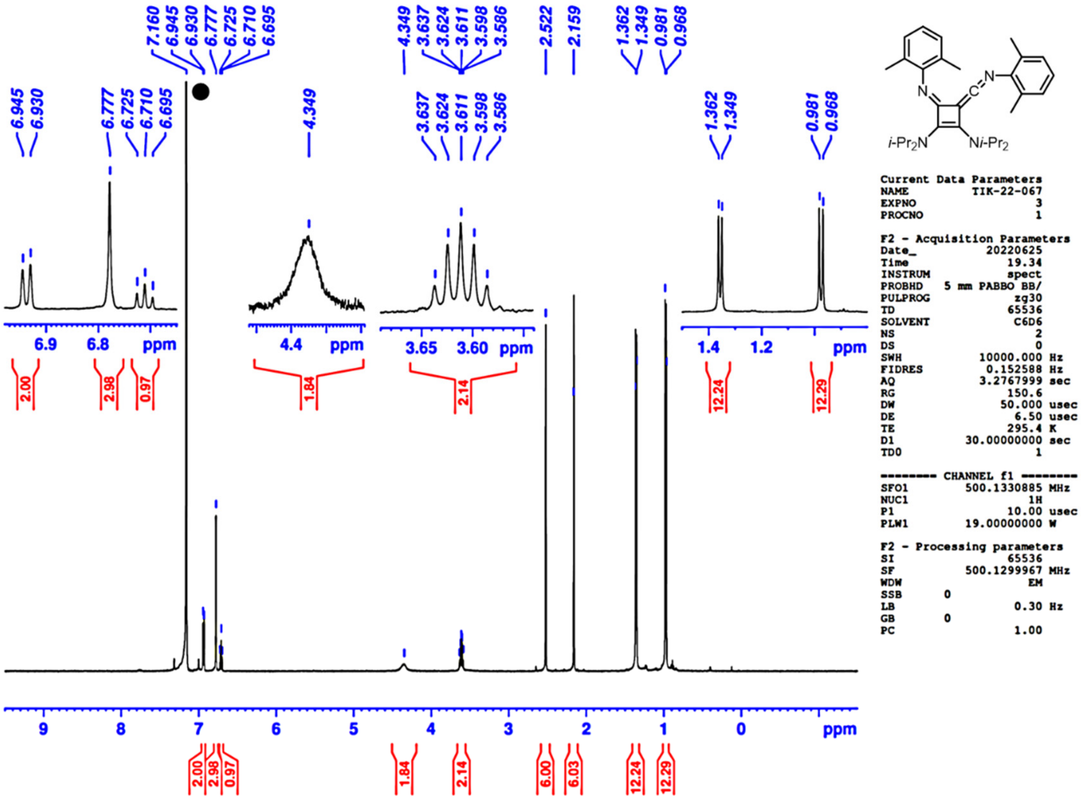

Figure S51. ^{1}H NMR spectrum of **2** in C_6D_6 at 295 K (● = C_6D_5H).

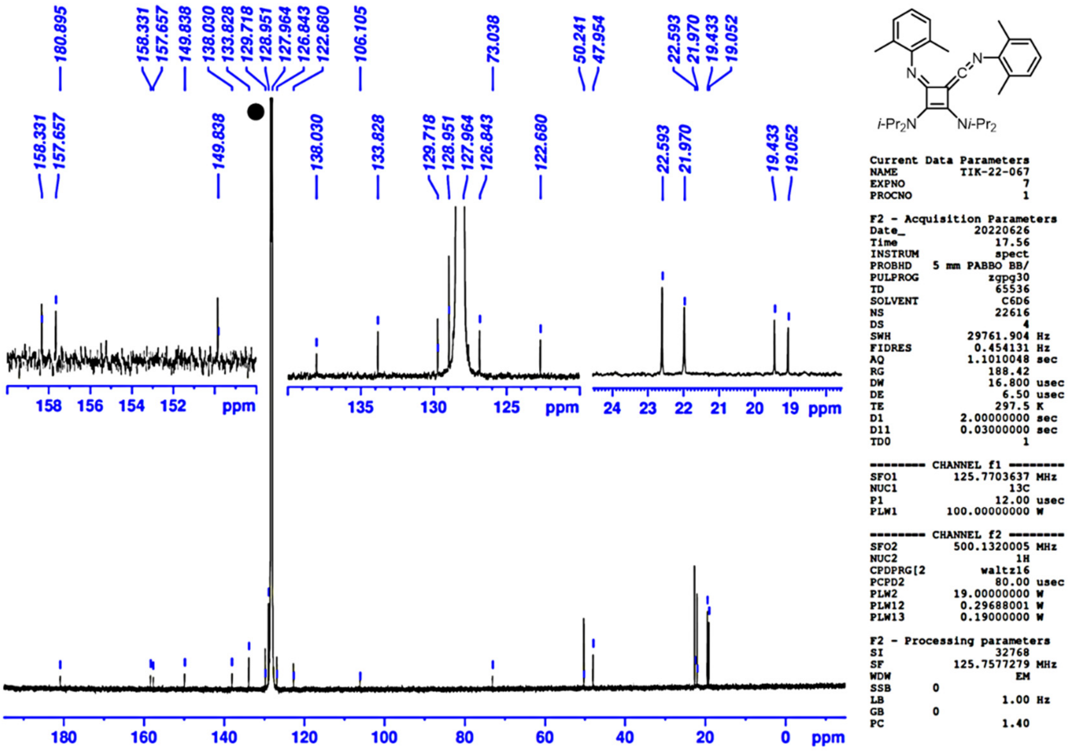

Figure S52. $^{13}C\{^{1}H\}$ NMR spectrum of **2** in C_6D_6 at 298 K (● = C_6D_6).

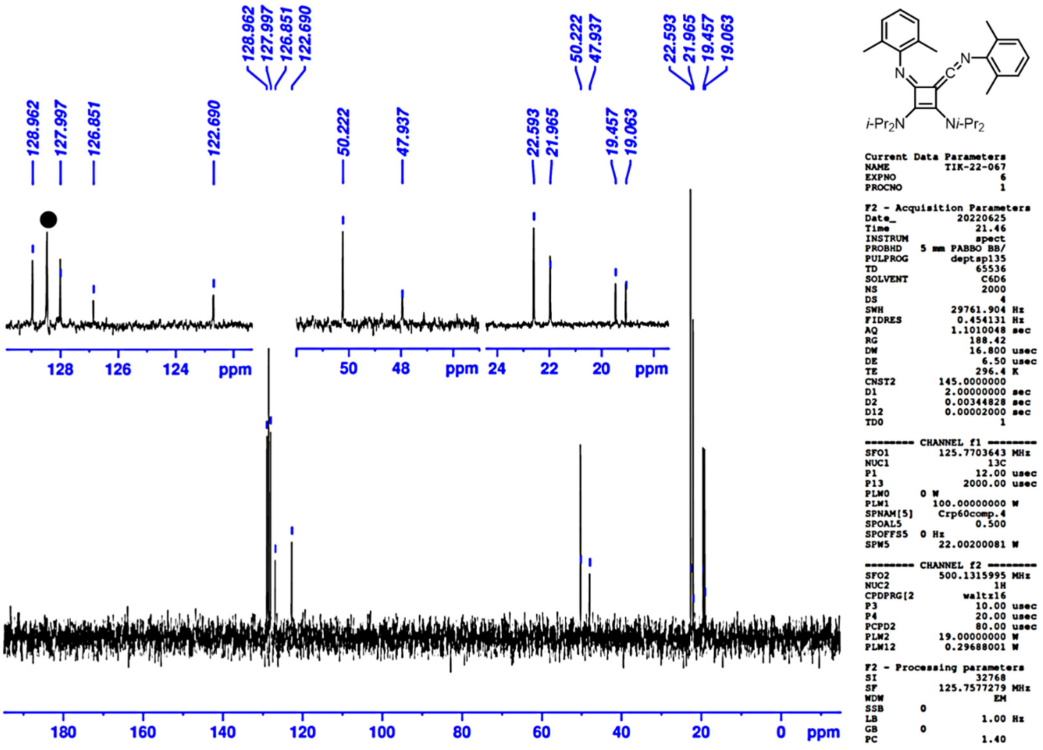

Figure S53. $^{13}C\{^1H\}$ (DEPT135) NMR spectrum of **2** in C_6D_6 at 296 K (● = C_6D_5H).

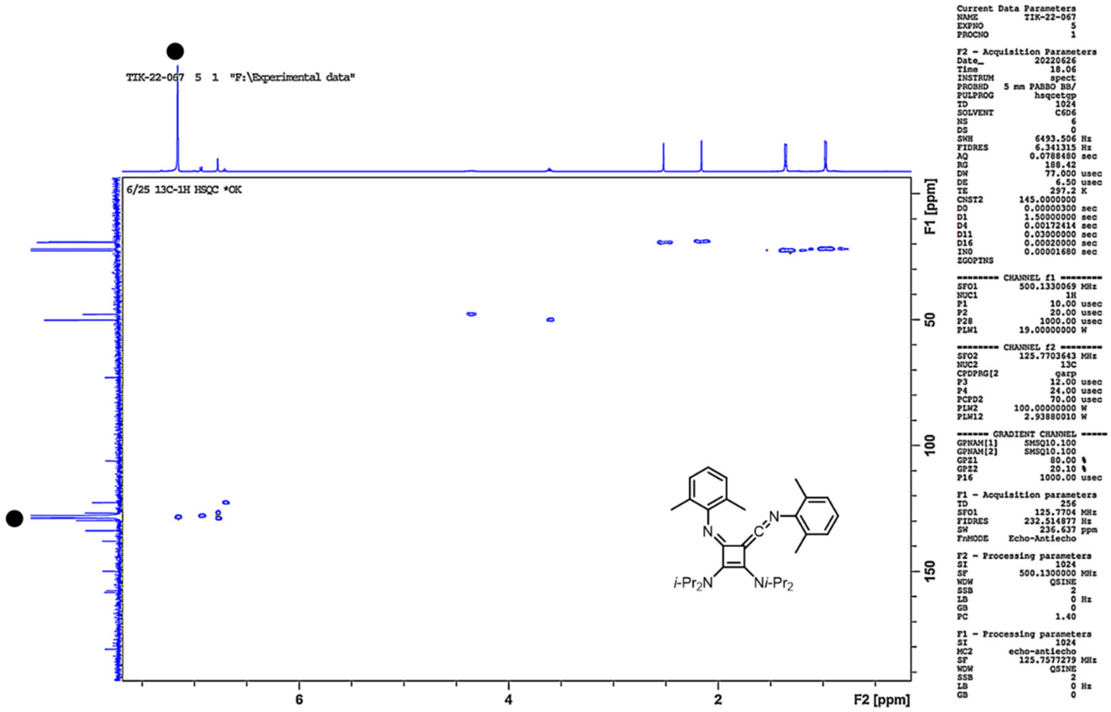

Figure S54. ^1H-^{13}C HSQC NMR spectrum of **2** in C_6D_6 at 297 K (● = C_6D_5H & C_6D_6).

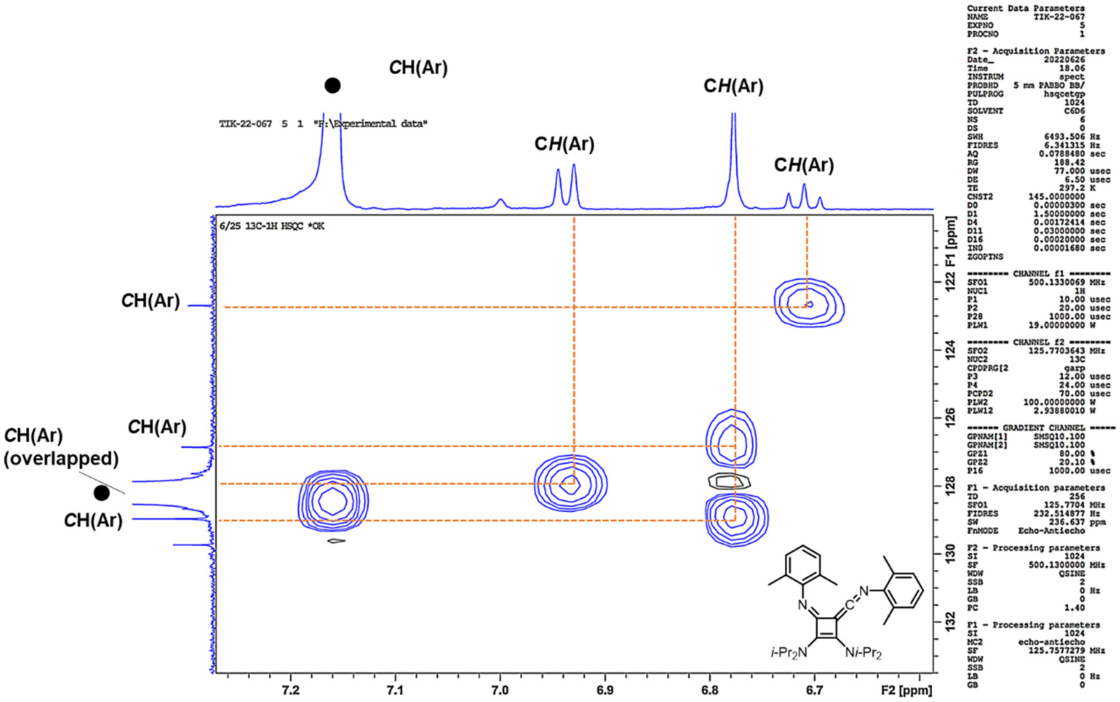

Figure S55. ^{1}H-^{13}C HSQC (magnified) NMR spectrum of **2** in C_6D_6 at 297 K (● = C_6D_5H & C_6D_6).

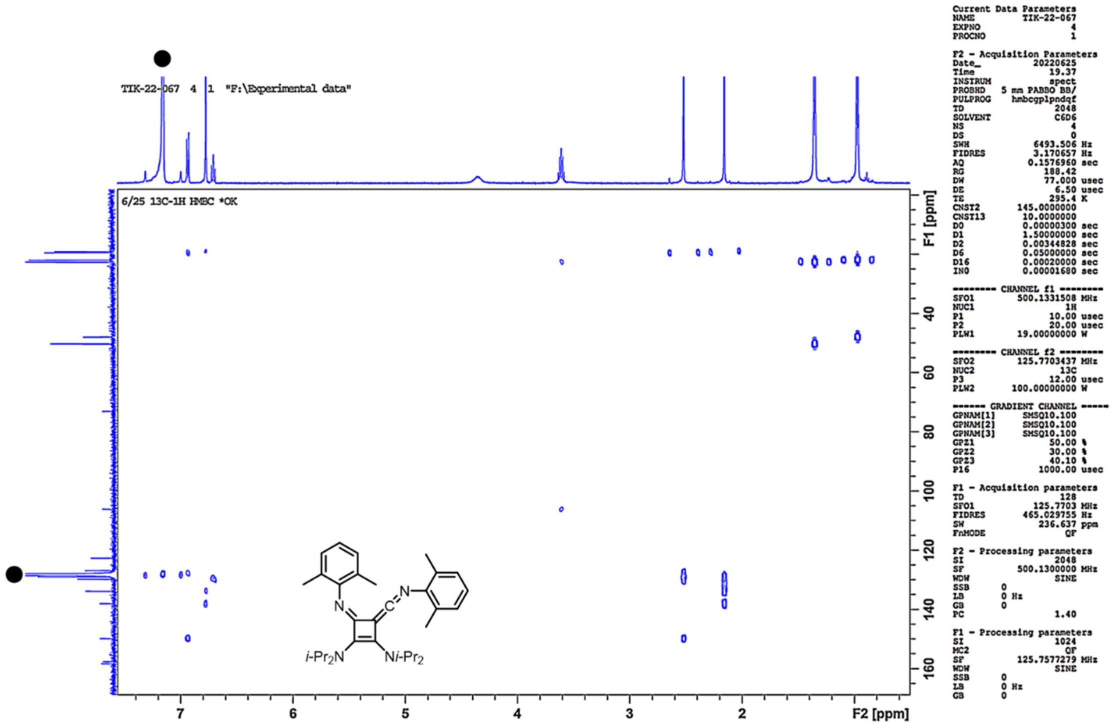

Figure S56. ^{1}H-^{13}C HMBC NMR spectrum of **2** in C_6D_6 at 295 K (● = C_6D_5H & C_6D_6).

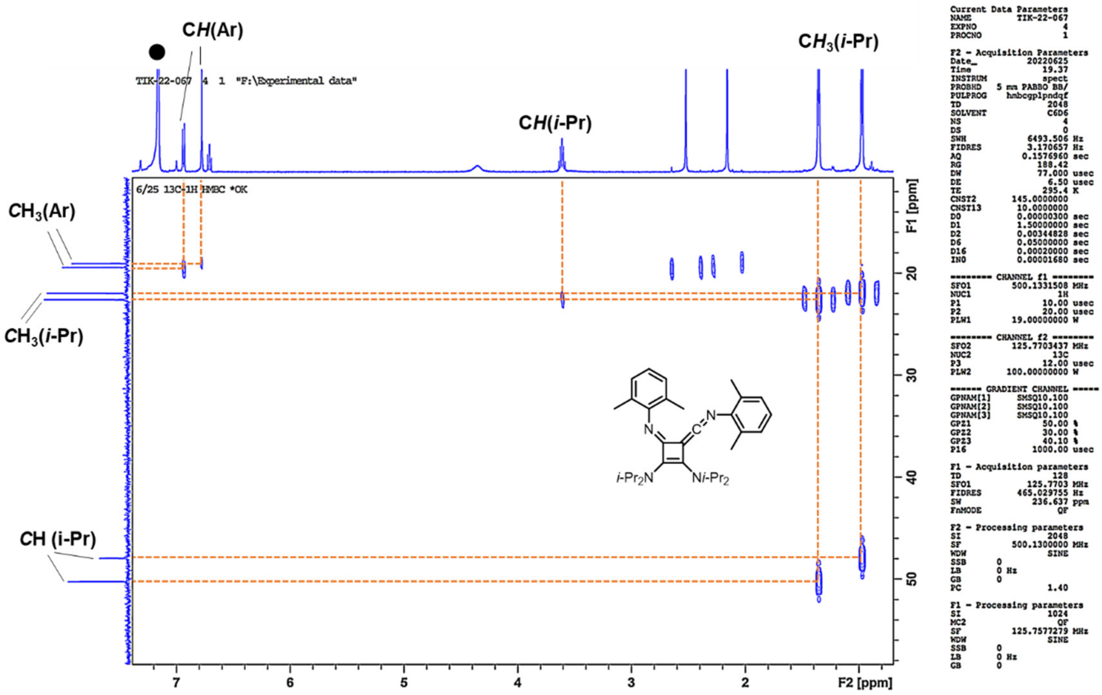

Figure S57. ^{1}H-^{13}C HMBC (magnified) NMR spectrum of **2** in C_6D_6 at 295 K (● = C_6D_5H).

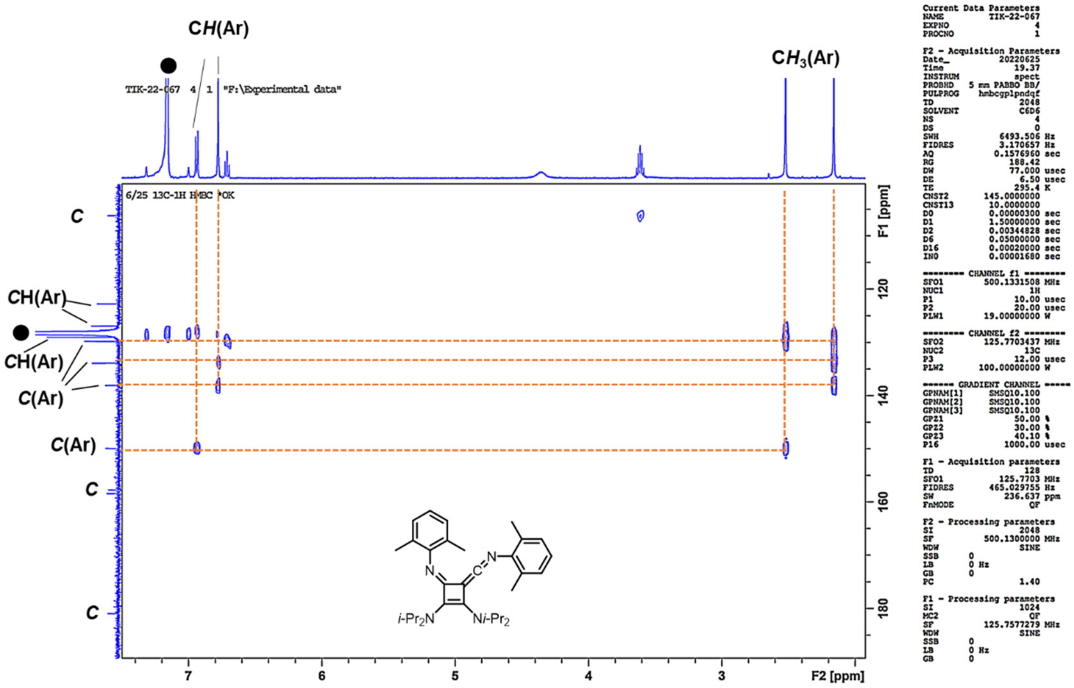

Figure S58. ^{1}H-^{13}C HMBC (magnified) NMR spectrum of **2** in C_6D_6 at 295 K (● = C_6D_5H & C_6D_6).

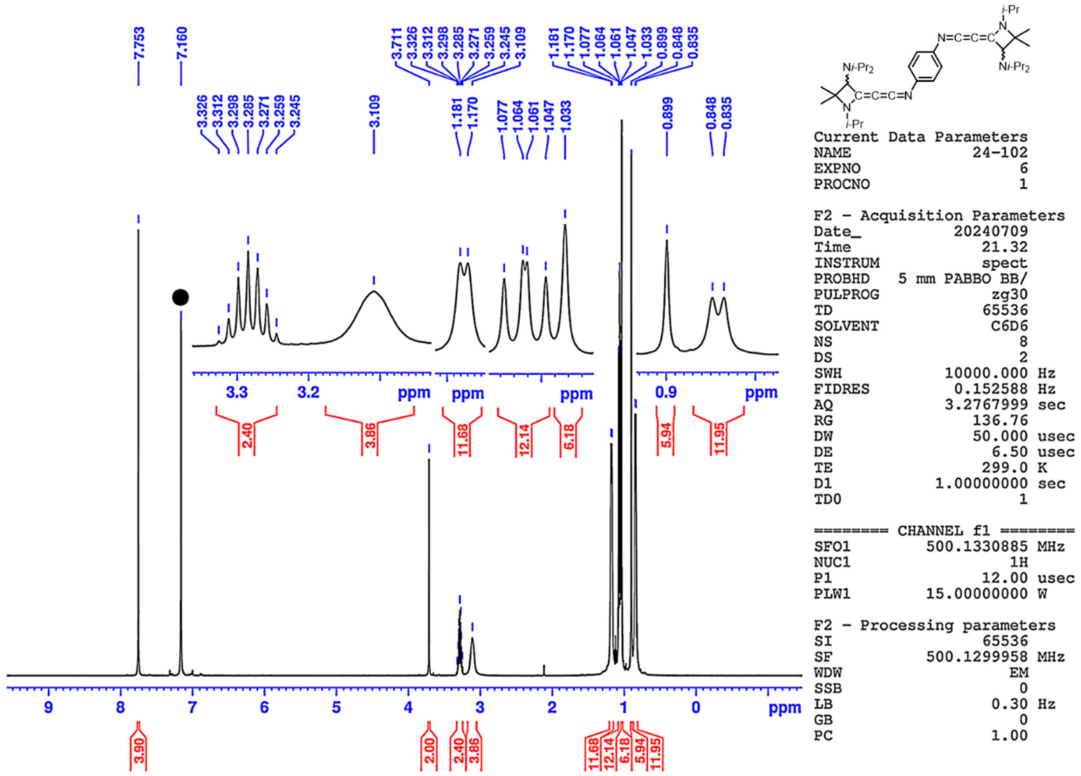

Figure S59. ^{1}H NMR spectrum of **4** in C_6D_6 at 299 K (● = C_6D_5H).

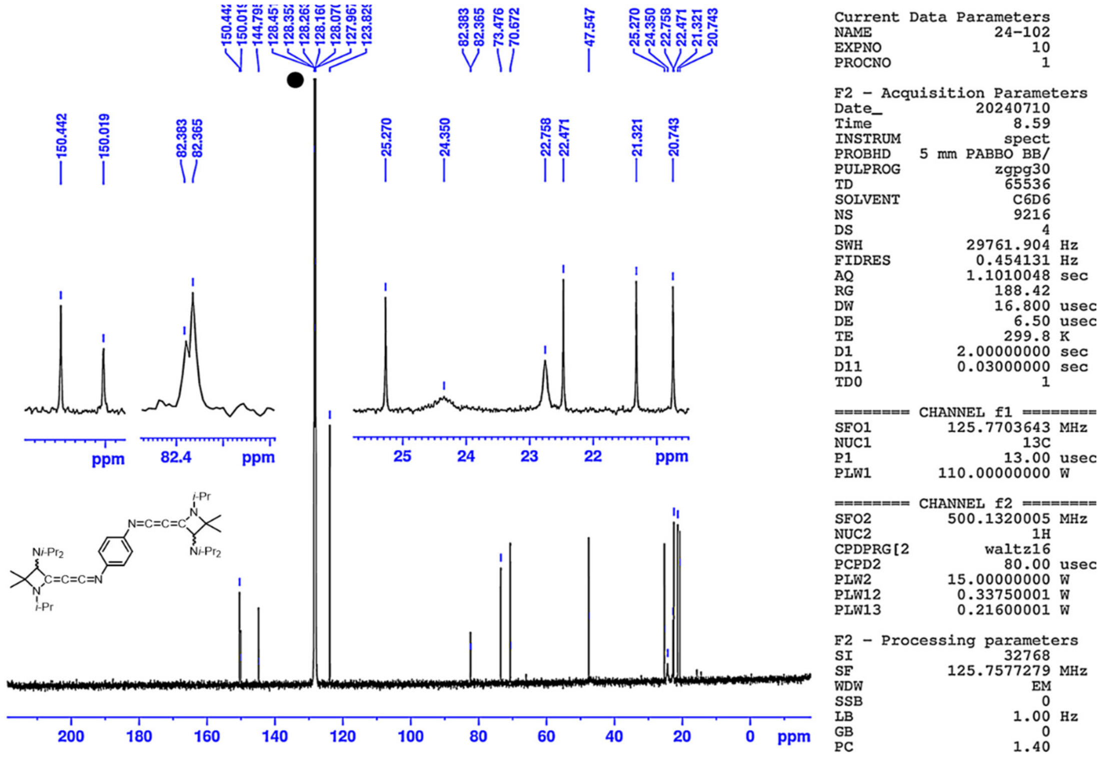

Figure S60. $^{13}C\{^{1}H\}$ NMR spectrum of **4** in C_6D_6 at 300 K (● = C_6D_6).

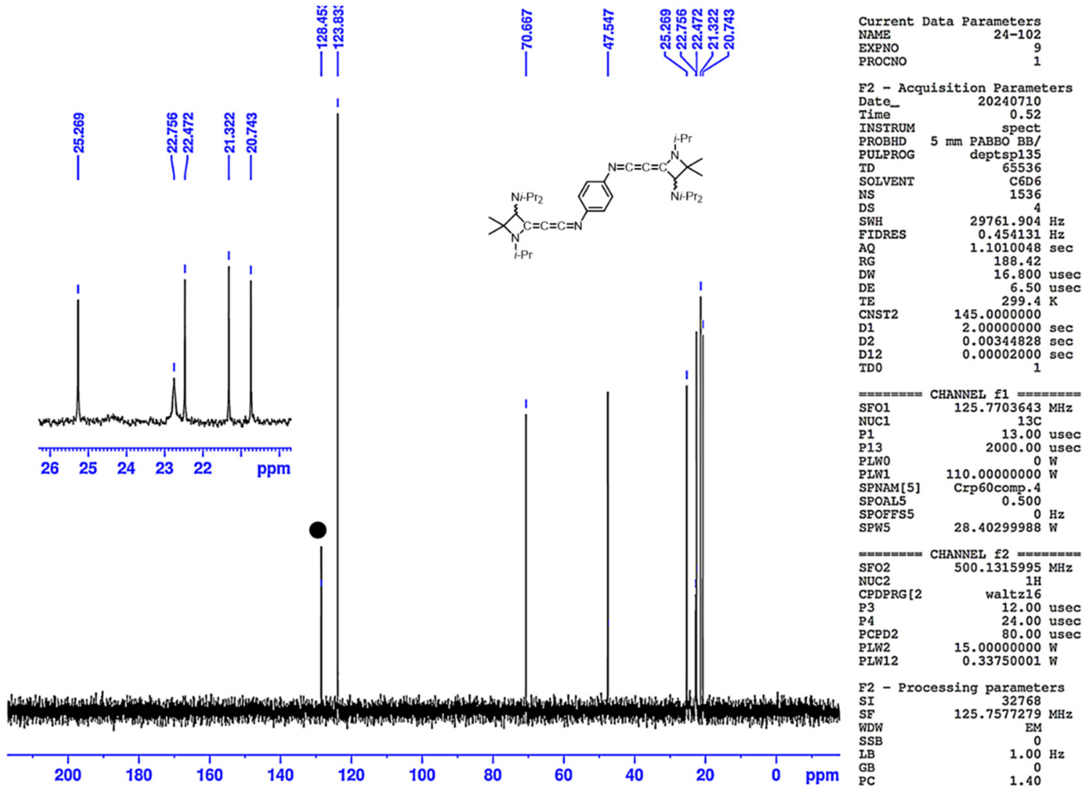

Figure S61. $^{13}C\{^{1}H\}$ (DEPT135) NMR spectrum of **4** in C_6D_6 at 299 K (● = C_6D_5H).

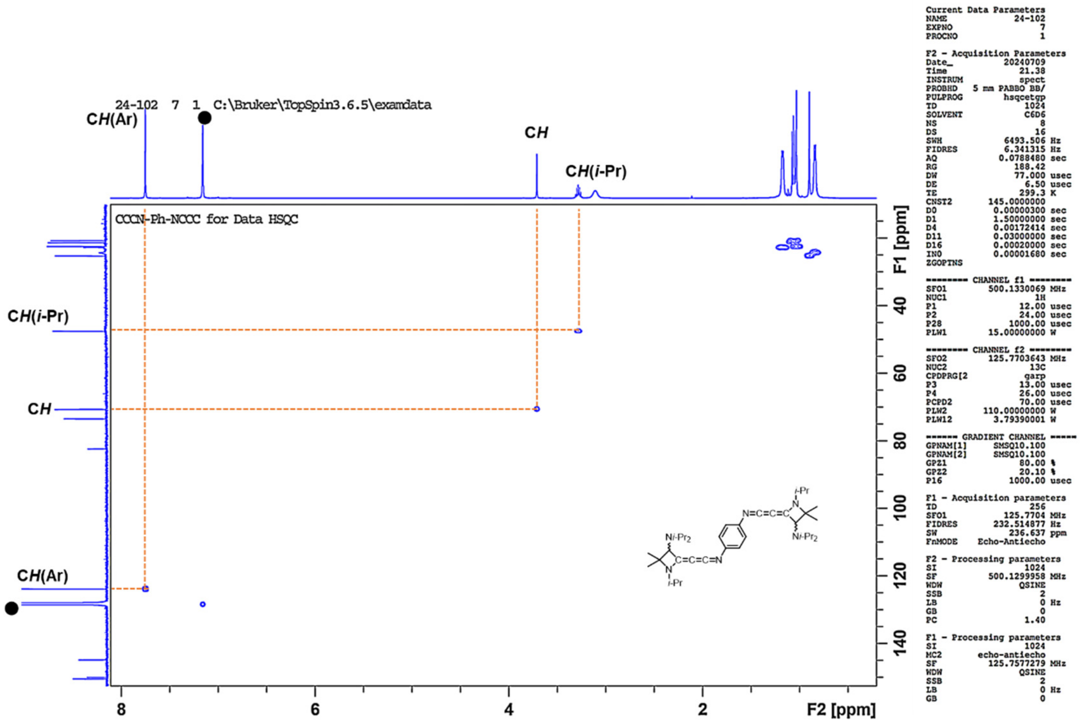

Figure S62. ^{1}H-^{13}C HSQC NMR spectrum of **4** in C_6D_6 at 299 K (● = C_6D_5H & C_6D_6).

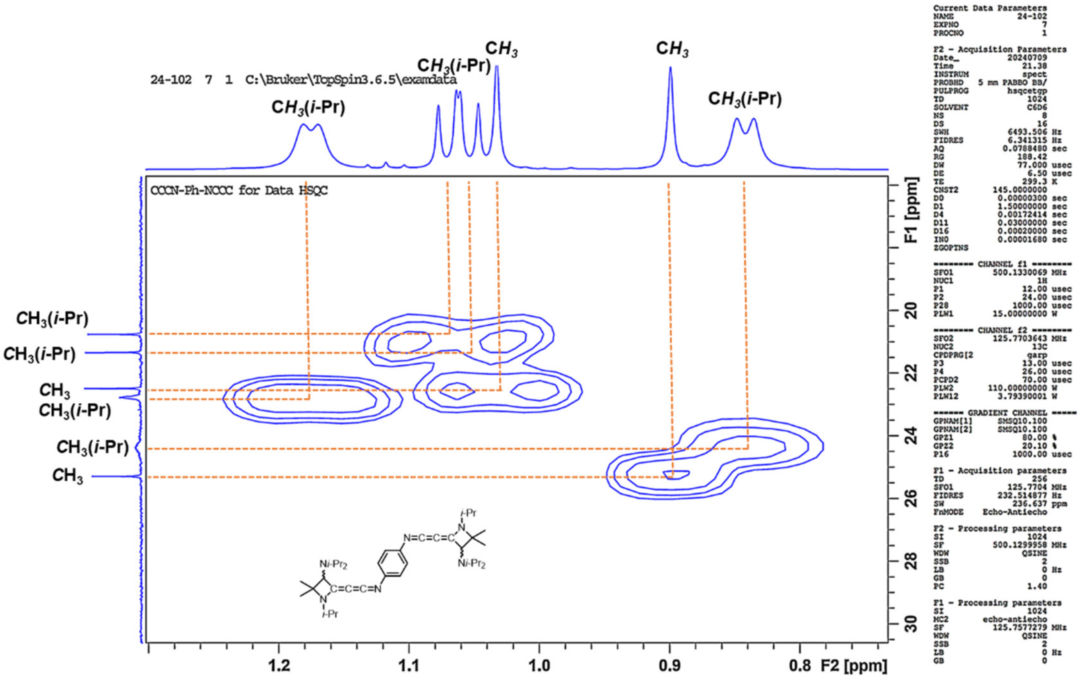

Figure S63. ^{1}H-^{13}C HSQC (magnified) NMR spectrum of **4** in C_6D_6 at 299 K.

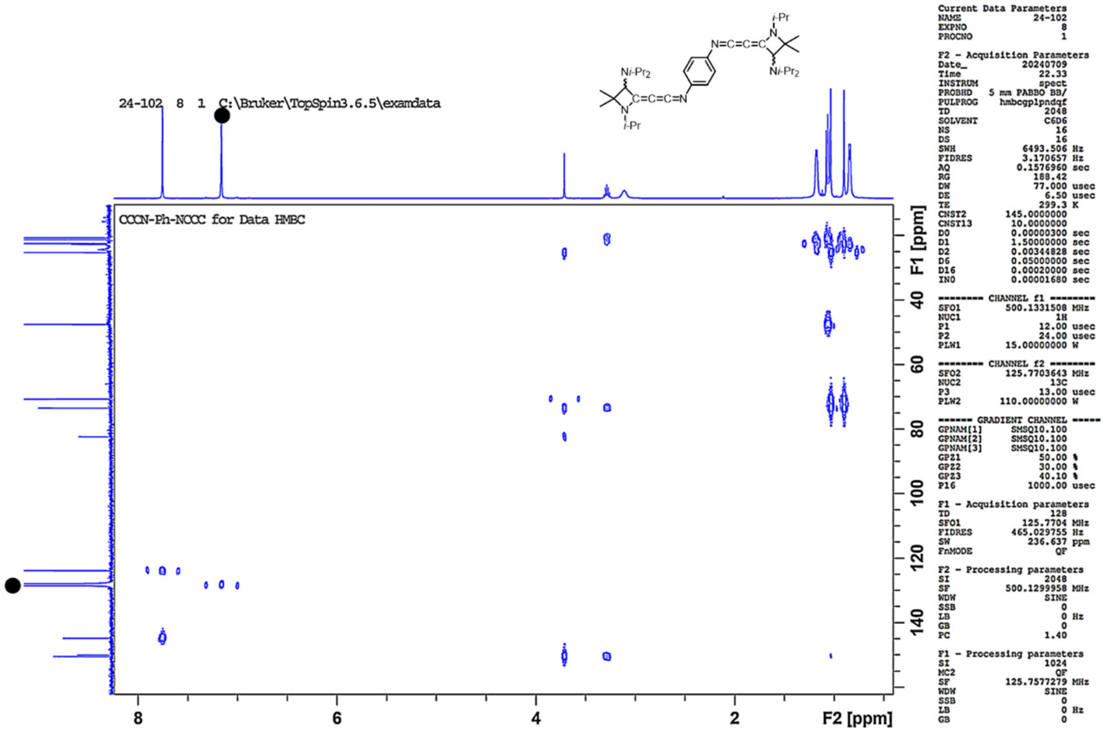

Figure S64. ^{1}H-^{13}C HMBC NMR spectrum of **4** in C_6D_6 at 299 K (● = C_6D_5H & C_6D_6).

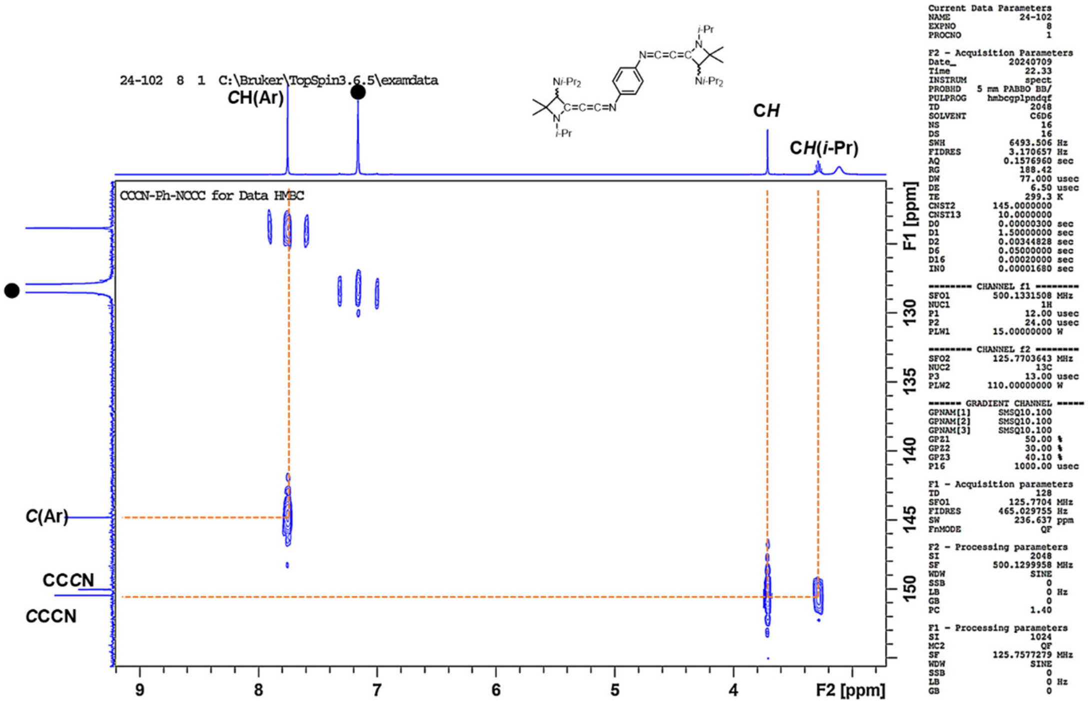

Figure S65. ^{1}H-^{13}C HMBC (magnified) NMR spectrum of **4** in C_6D_6 at 299 K.

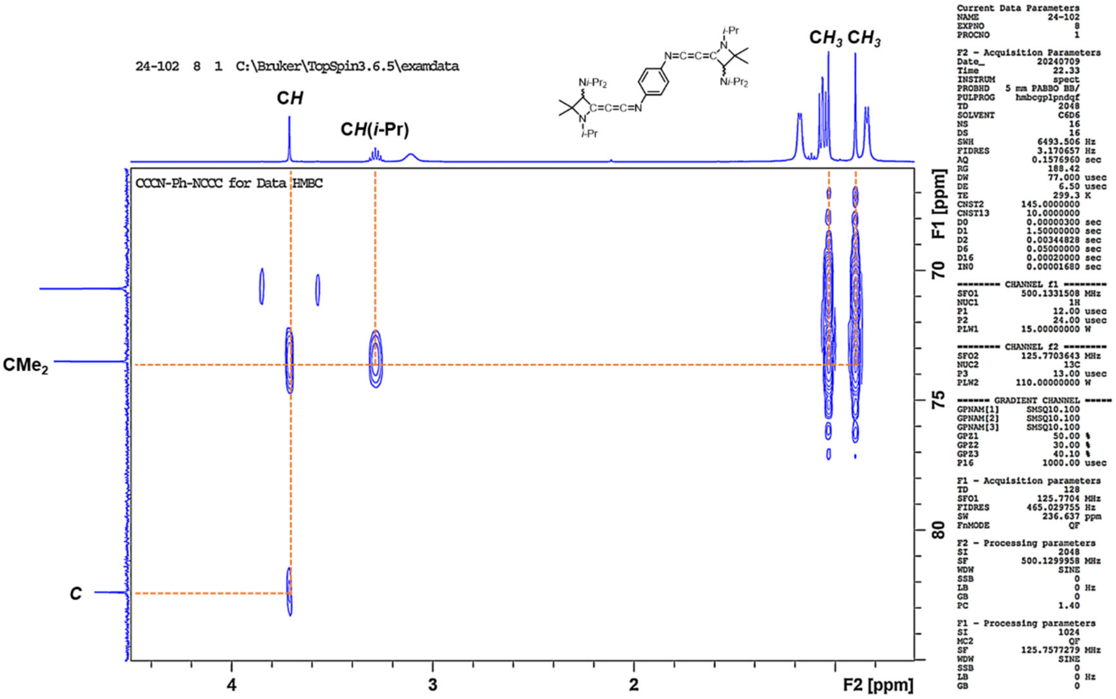

Figure S66. ^{1}H-^{13}C HMBC (magnified) NMR spectrum of **4** in C_6D_6 at 299 K.

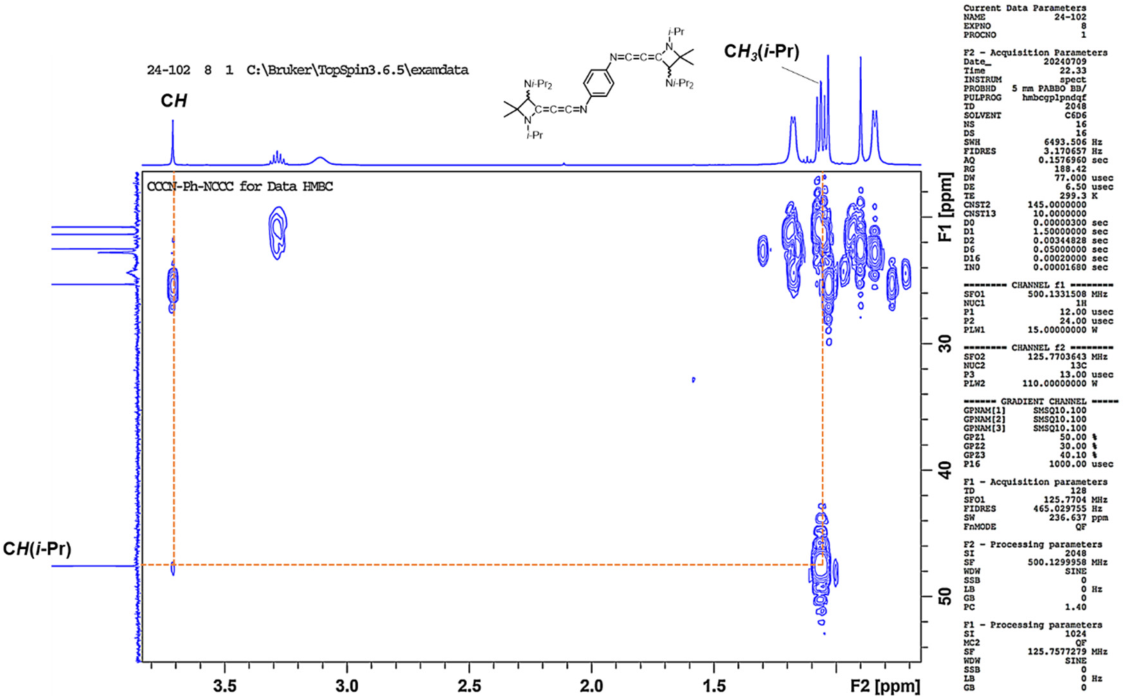

Figure S67. ^{1}H-^{13}C HMBC (magnified) NMR spectrum of **4** in C_6D_6 at 299 K.

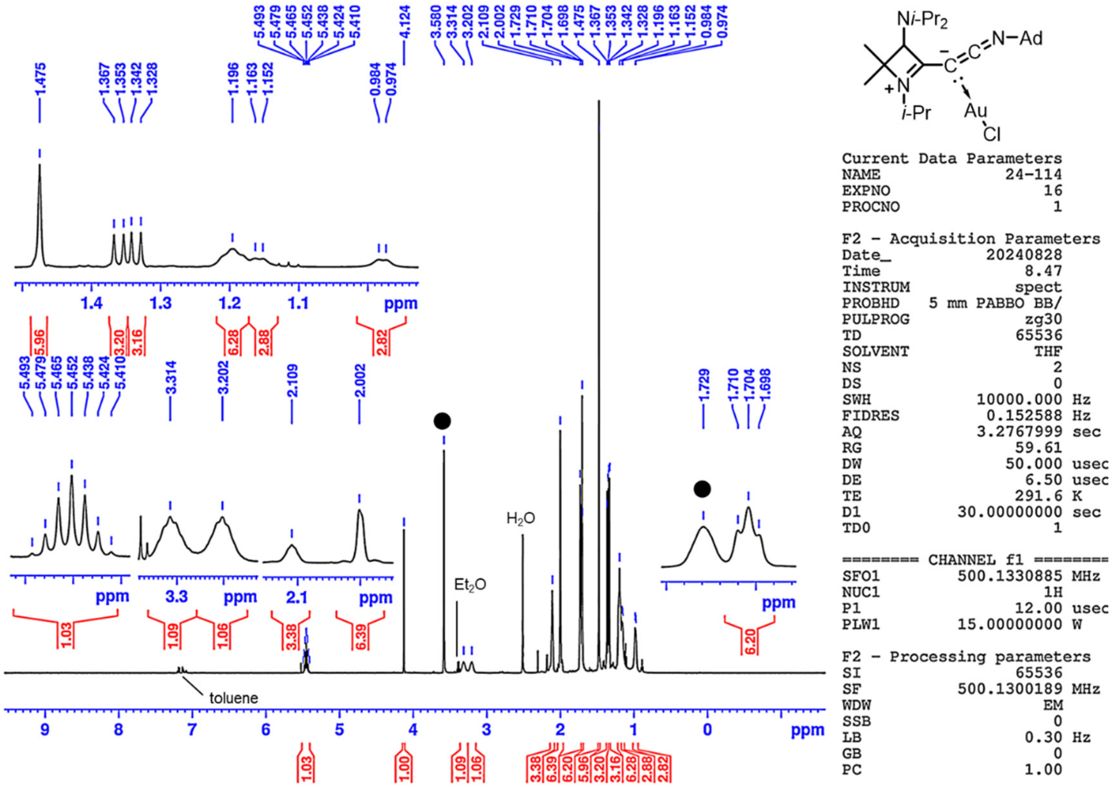

Figure S68. ^{1}H NMR spectrum of **5** in THF-d_8 at 292 K (● = C_4D_7HO).

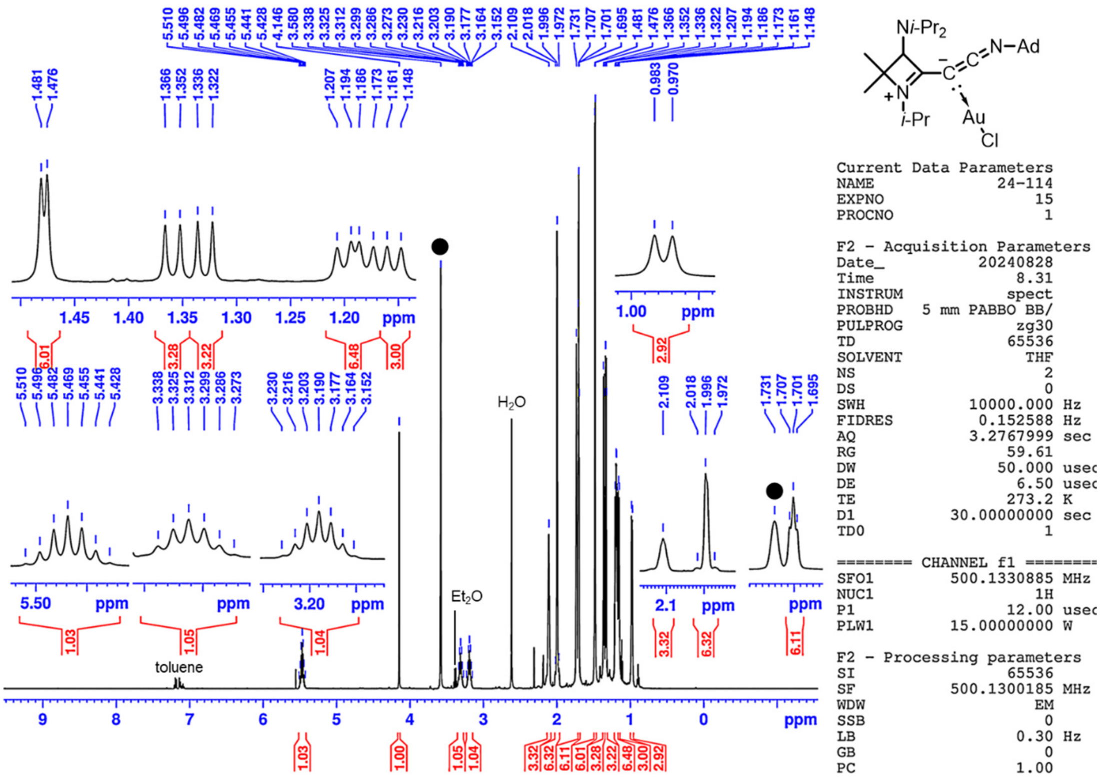

Figure S69. ^{1}H NMR spectrum of **5** in THF-d_8 at 273 K (● = C_4D_7HO).

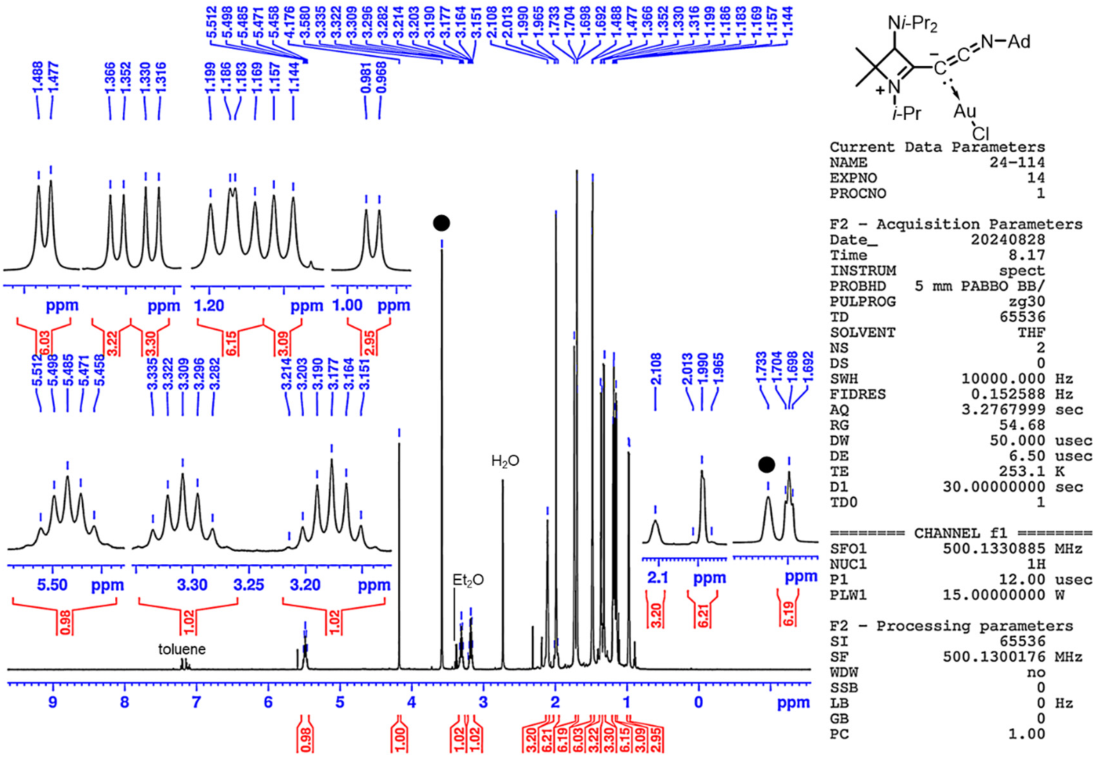

Figure S70. ^{1}H NMR spectrum of **5** in THF-d_8 at 253 K (● = C_4D_7HO).

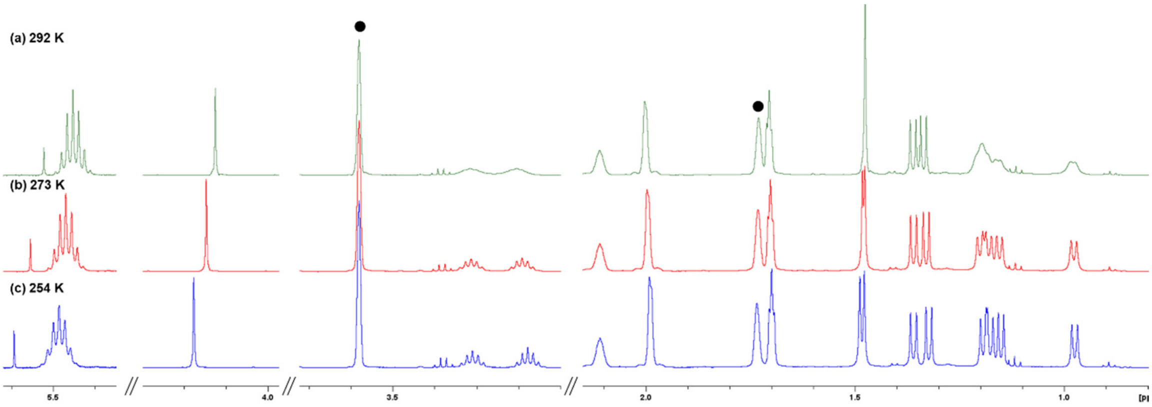

Figure S71. ^{1}H NMR spectrum of **5** in THF-d_8 at various temperatures (● = C_4D_7HO).

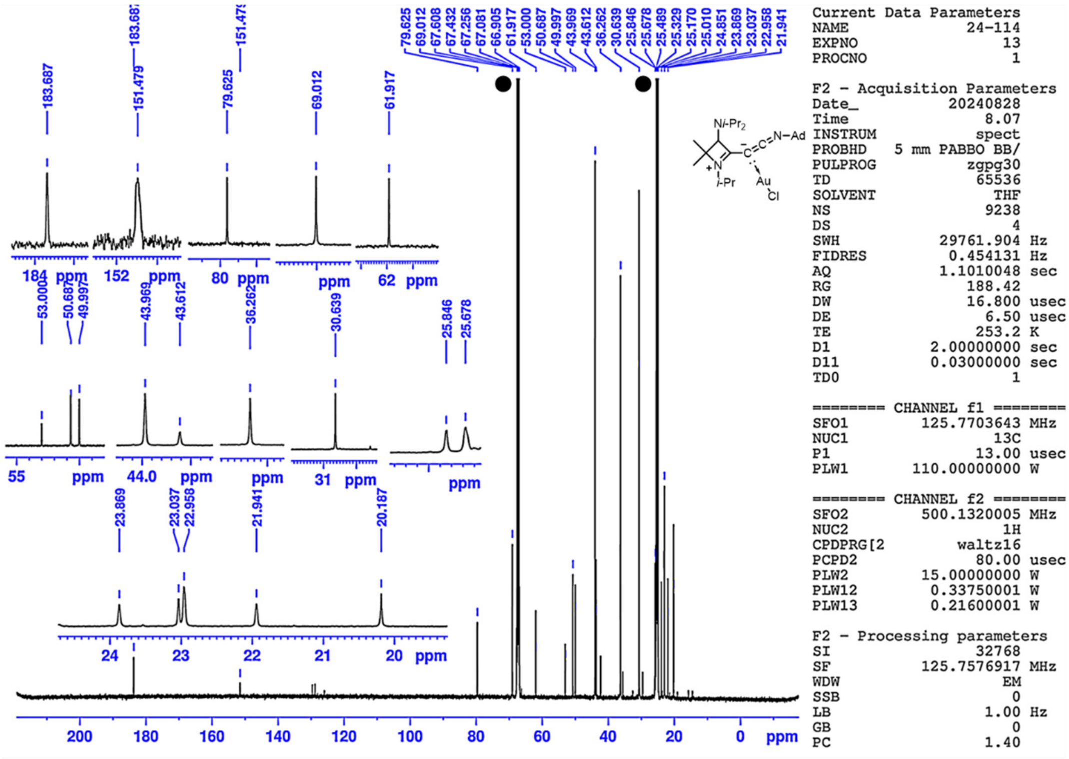

Figure S72. $^{13}C\{^{1}H\}$ NMR spectrum of **5** in THF-d_8 at 253 K (● = C_4D_8O).

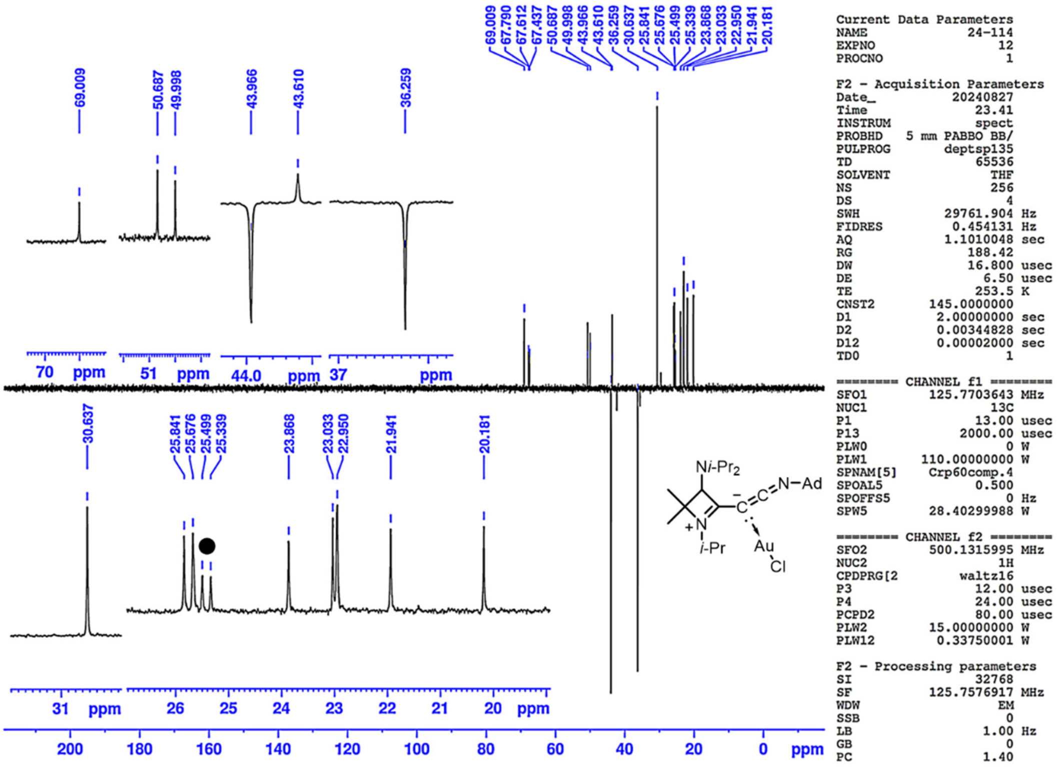

Figure S73. $^{13}C\{^{1}H\}$ (DEPT135) NMR spectrum of **5** in THF-d_8 at 254 K (● = C_4D_7HO).

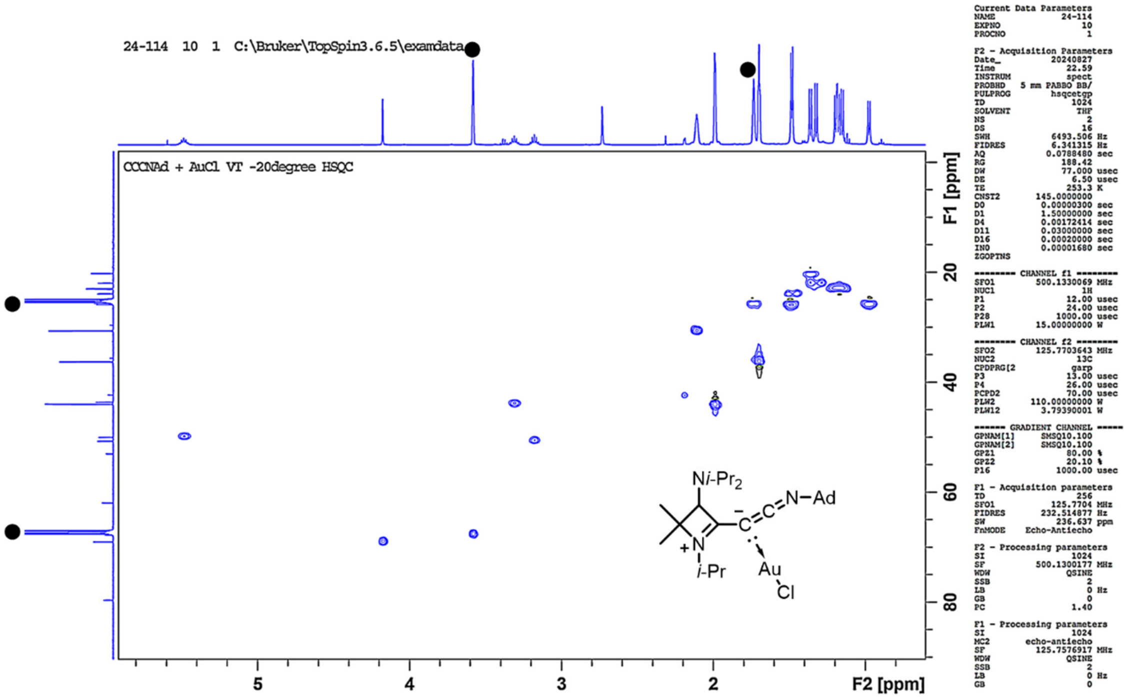

Figure S74. ^{1}H-^{13}C HSQC NMR spectrum of **5** in THF-d_8 at 253 K (● = C_4D_7HO & C_4D_8O).

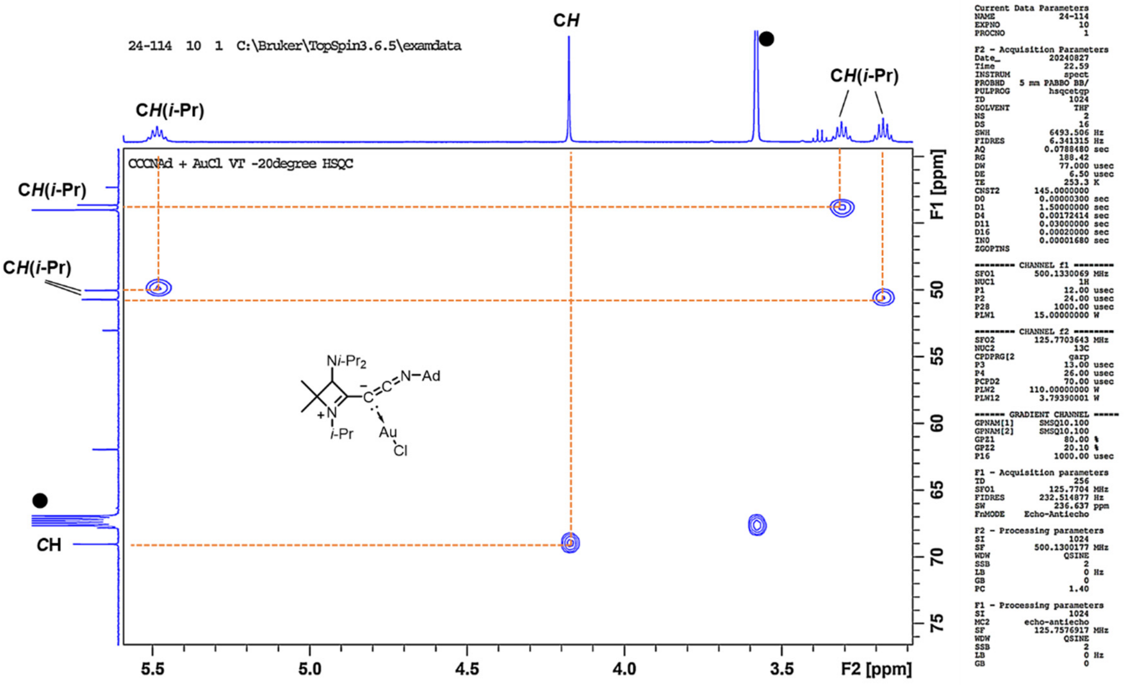

Figure S75. ^{1}H-^{13}C HSQC (magnified) NMR spectrum of **5** in THF-d_8 at 253 K (● = C_4D_7HO & C_4D_8O).

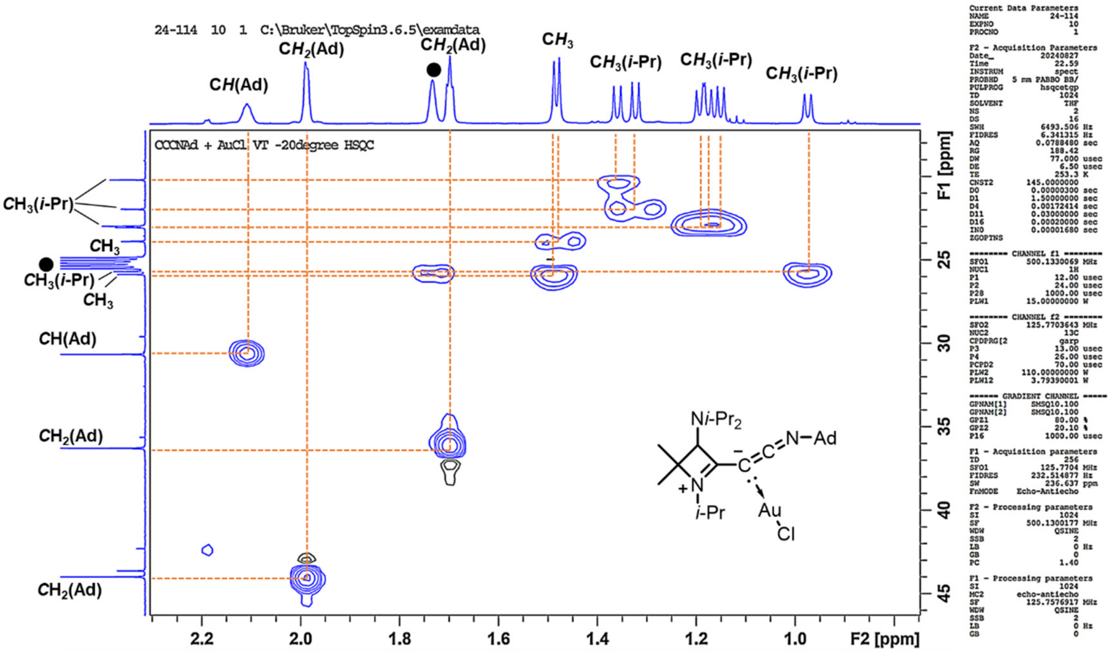

Figure S76. ^{1}H-^{13}C HSQC (magnified) NMR spectrum of **5** in THF-d_8 at 253 K (● = C_4D_7HO & C_4D_8O).

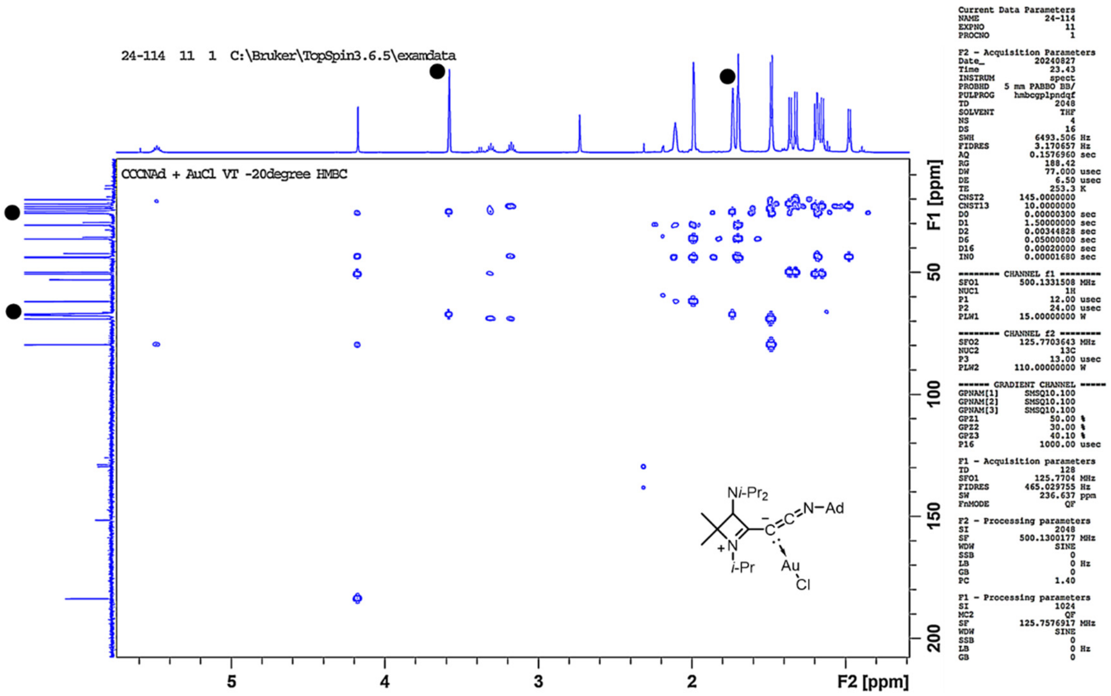

Figure S77. ^{1}H-^{13}C HMBC NMR spectrum of **5** in THF-d_8 at 253 K (● = C_4D_7HO & C_4D_8O).

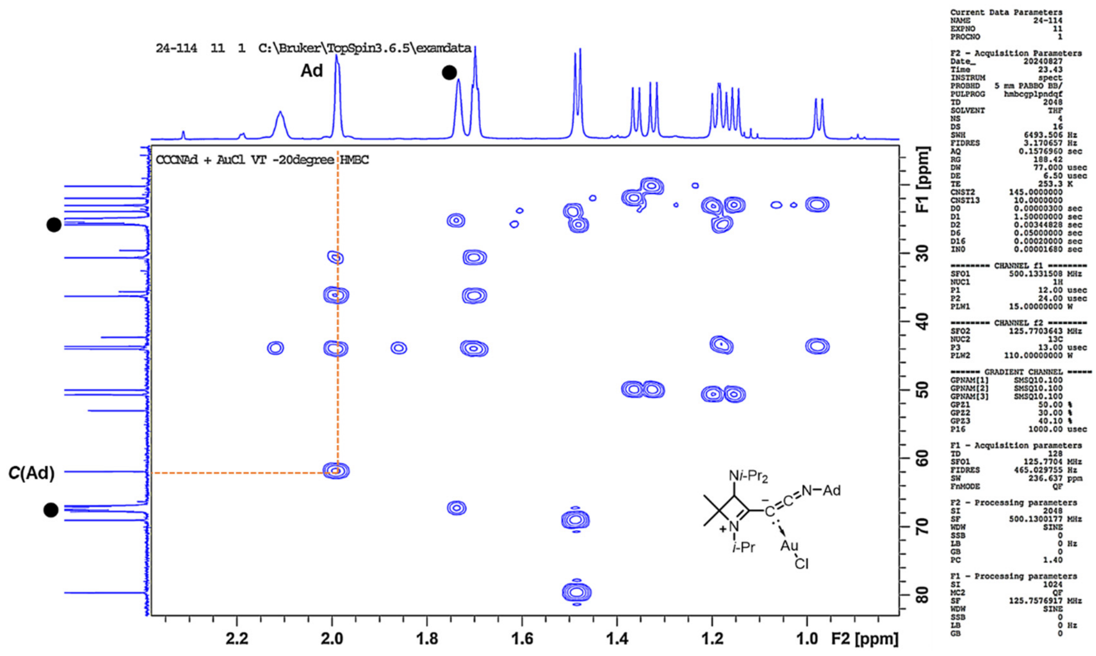

Figure S78. ^{1}H-^{13}C HMBC (magnified) NMR spectrum of **5** in THF-d_8 at 253 K (● = C_4D_7HO & C_4D_8O).

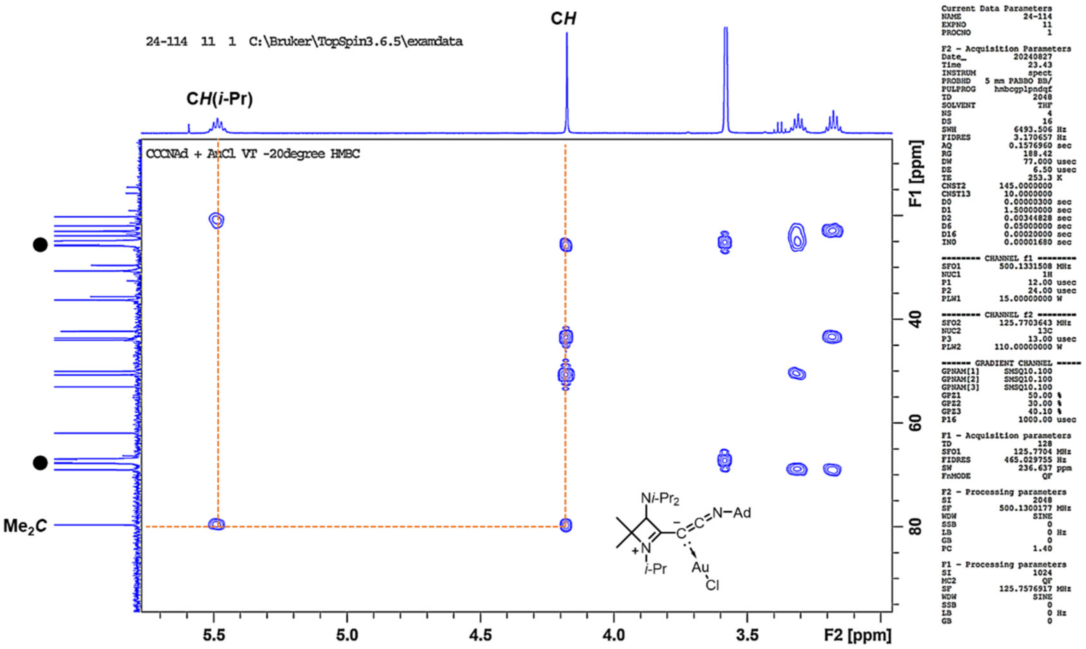

Figure S79. ^{1}H-^{13}C HMBC (magnified) NMR spectrum of **5** in THF-d_8 at 253 K (● = C_4D_7HO & C_4D_8O).

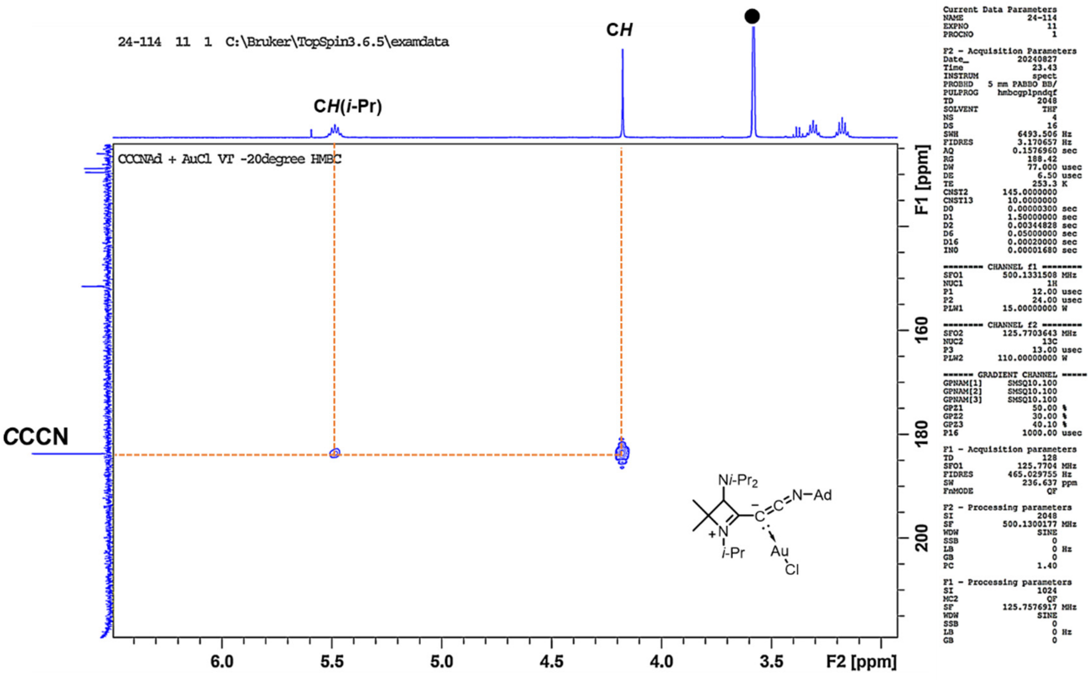

Figure S80. ^{1}H-^{13}C HMBC (magnified) NMR spectrum of **5** in THF-d_8 at 253 K (● = C_4D_7HO).

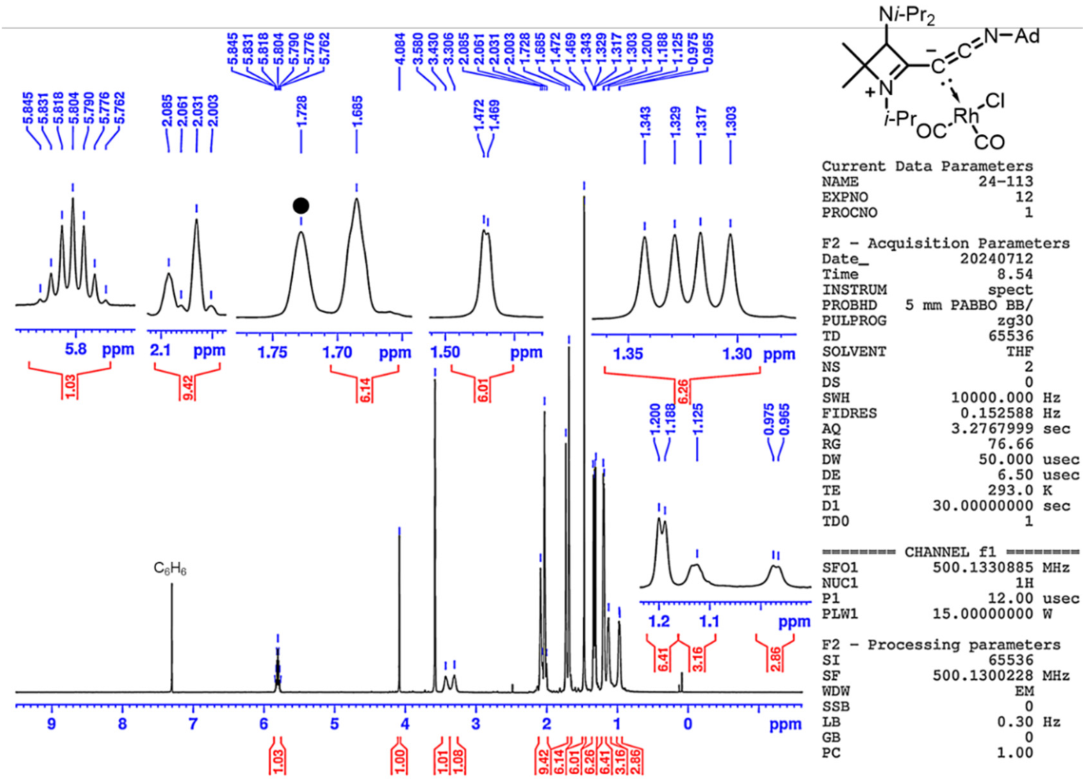

Figure S81. ^{1}H NMR spectrum of **6** in THF-d_8 at 293 K (● = C_4D_7HO).

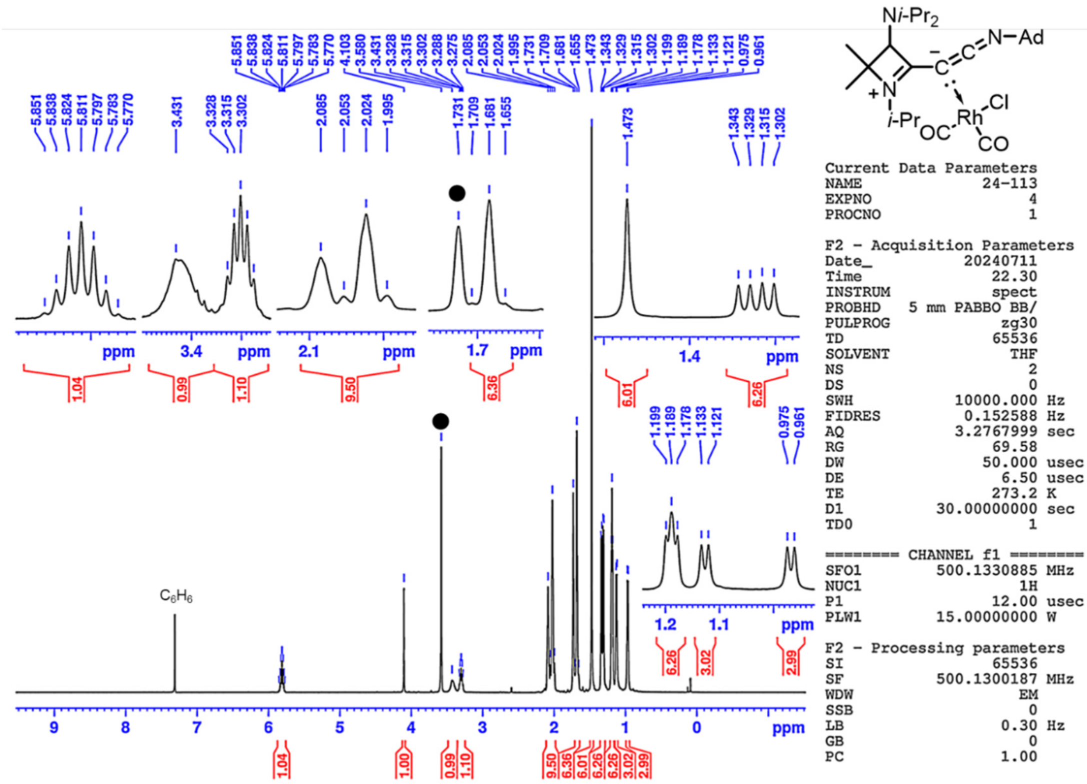

Figure S82. ^{1}H NMR spectrum of **6** in THF-d_8 at 273 K (● = C_4D_7HO).

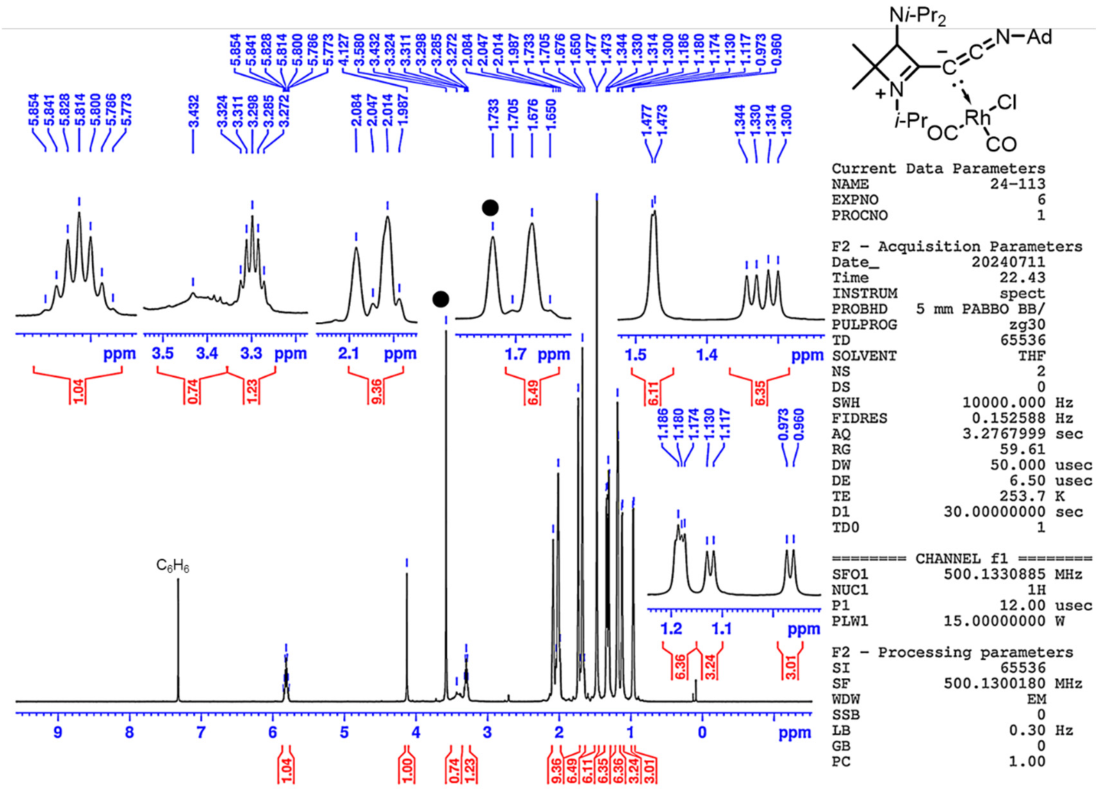

Figure S83. ^{1}H NMR spectrum of **6** in THF-d_8 at 254 K (● = C_4D_7HO).

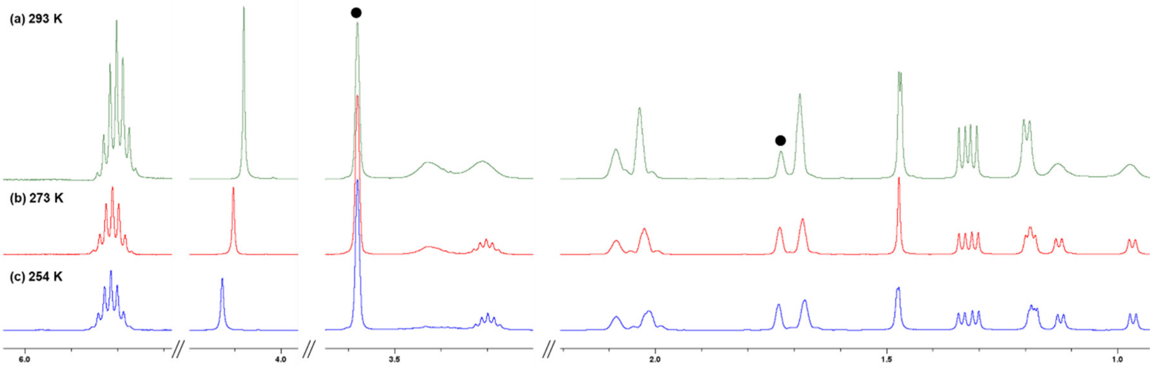

Figure S84. ^{1}H NMR spectrum of **6** in THF-d_8 at various temperatures (● = C_4D_7HO).

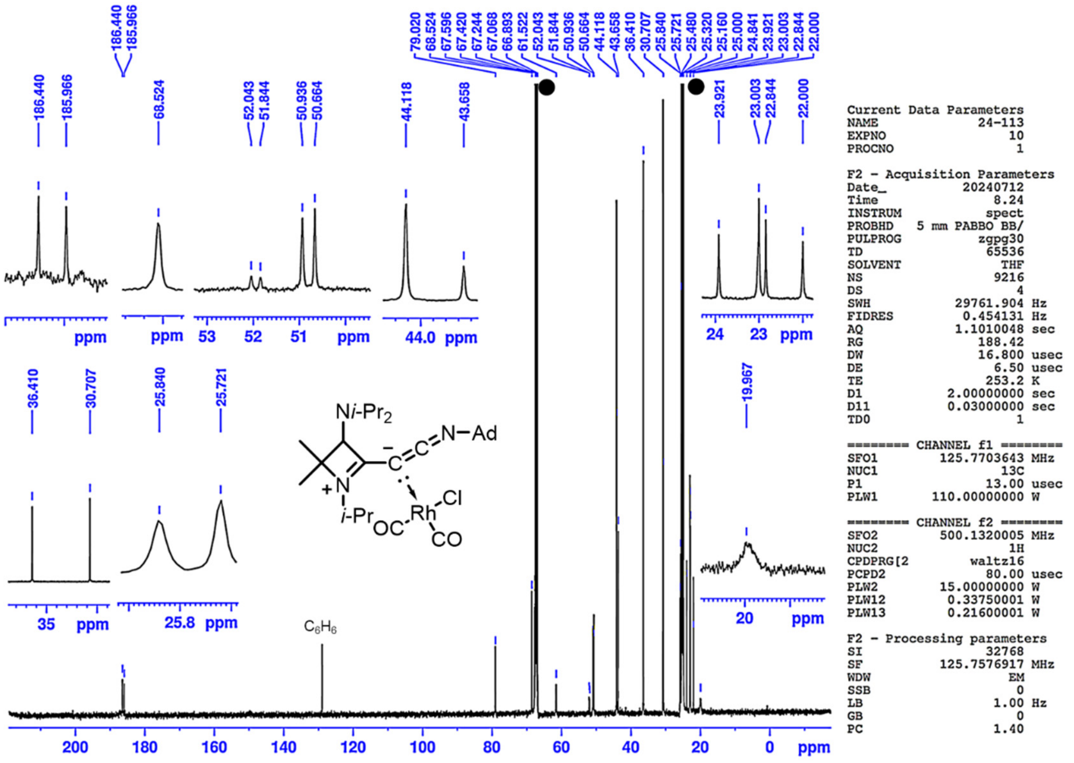

Figure S85. $^{13}C\{^1H\}$ NMR spectrum of **6** in THF-d_8 at 253 K (● = C_4D_7HO).

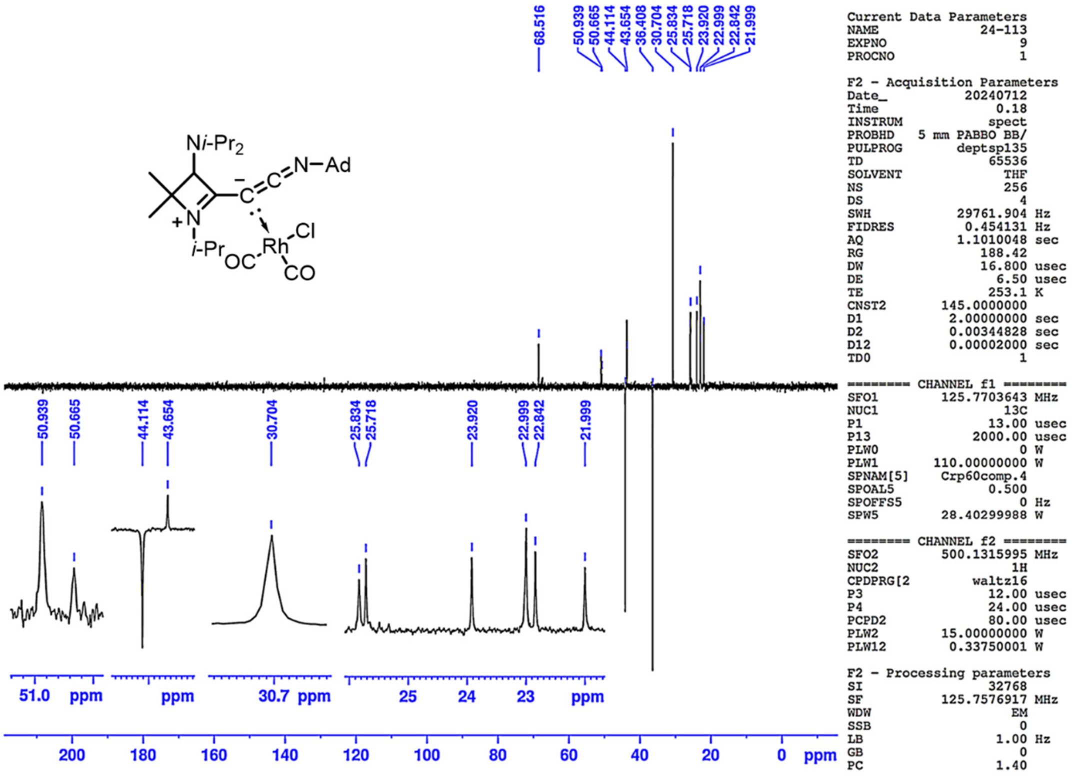

Figure S86. $^{13}C\{^1H\}$ (DEPT135) NMR spectrum of **6** in THF-d_8 at 253 K.

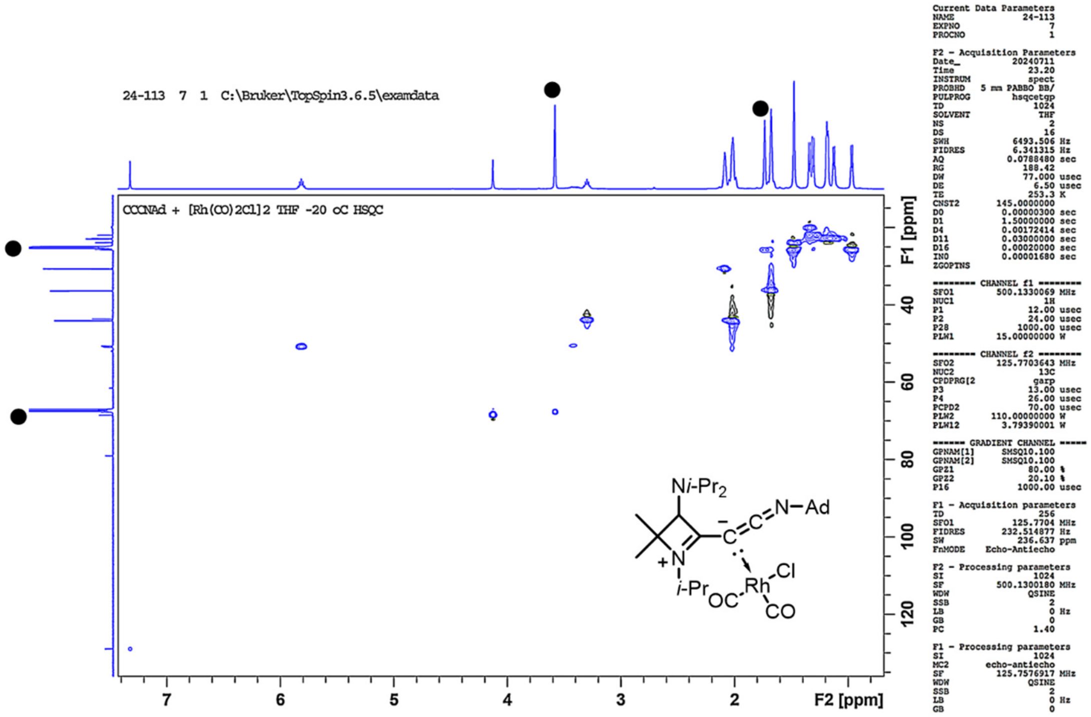

Figure S87. ^{1}H-^{13}C HSQC NMR spectrum of **6** in THF-d_8 at 253 K (● = C_4D_7HO & C_4D_8O).

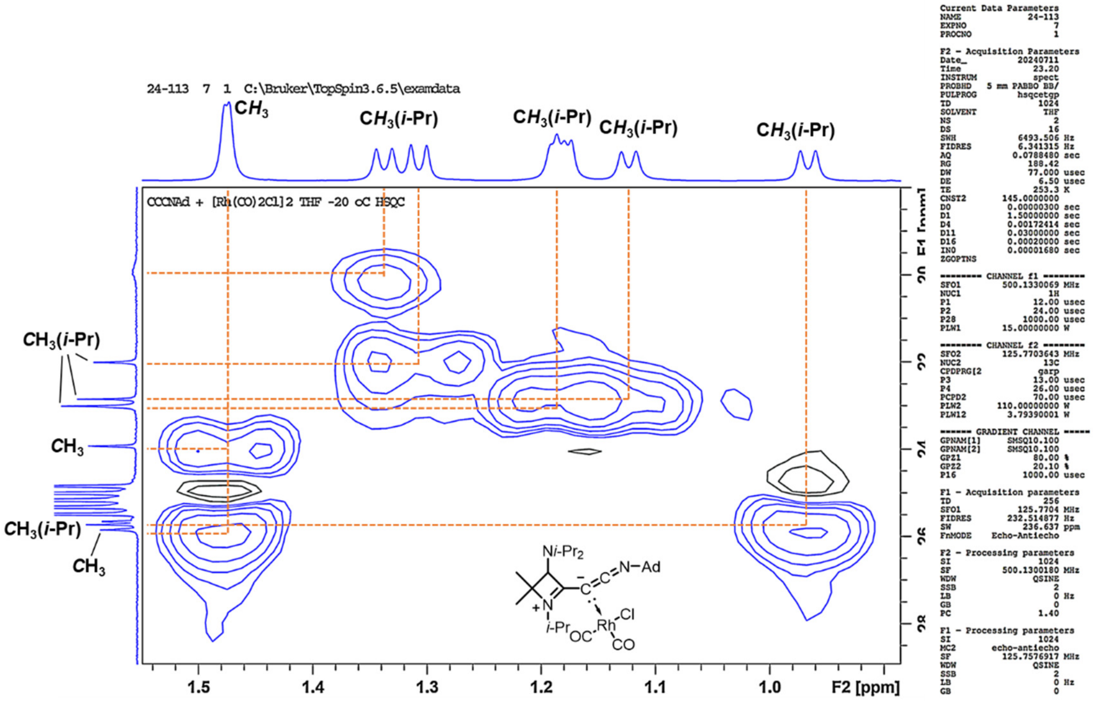

Figure S88. ^{1}H-^{13}C HSQC (magnified) NMR spectrum of **6** in THF-d_8 at 253 K.

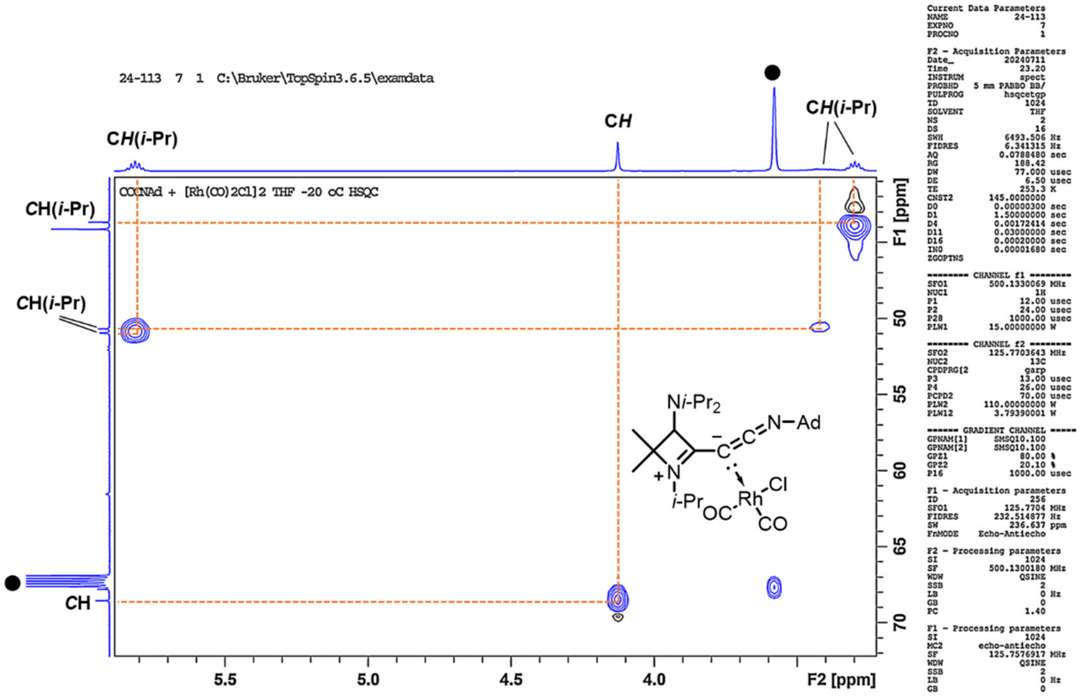

Figure S89. ^1H-^{13}C HSQC (magnified) NMR spectrum of **6** in THF-d_8 at 253 K (● = C_4D_7HO & C_4D_8O).

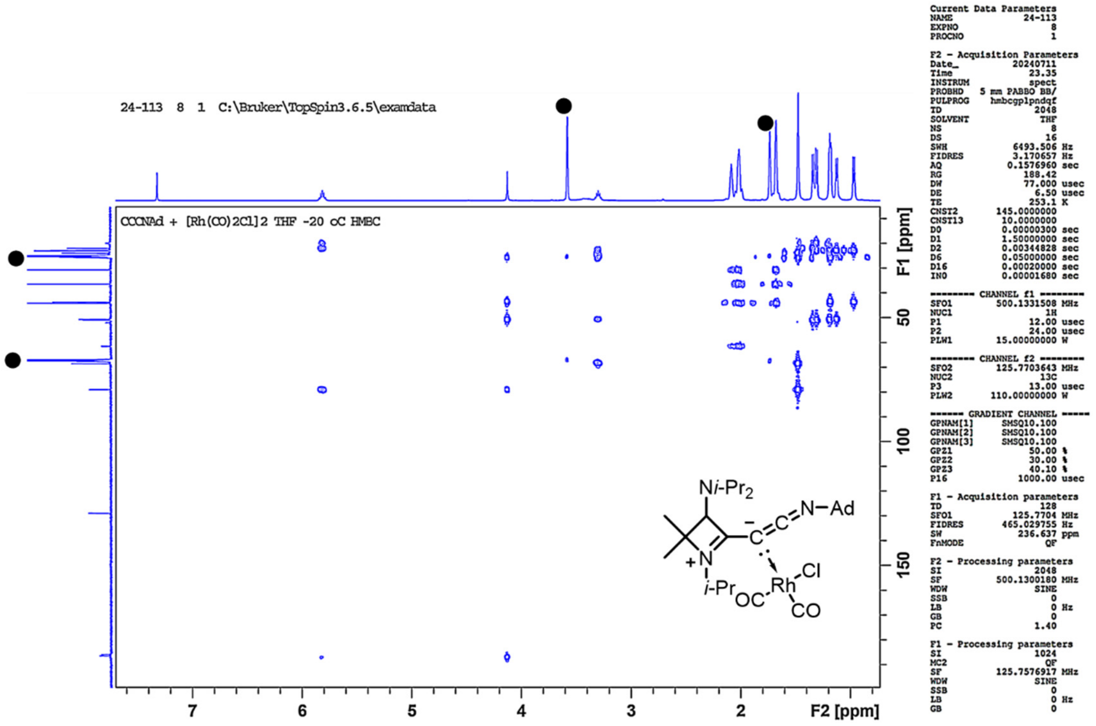

Figure S90. ^1H-^{13}C HMBC NMR spectrum of **6** in THF-d_8 at 253 K (● = C_4D_7HO & C_4D_8O).

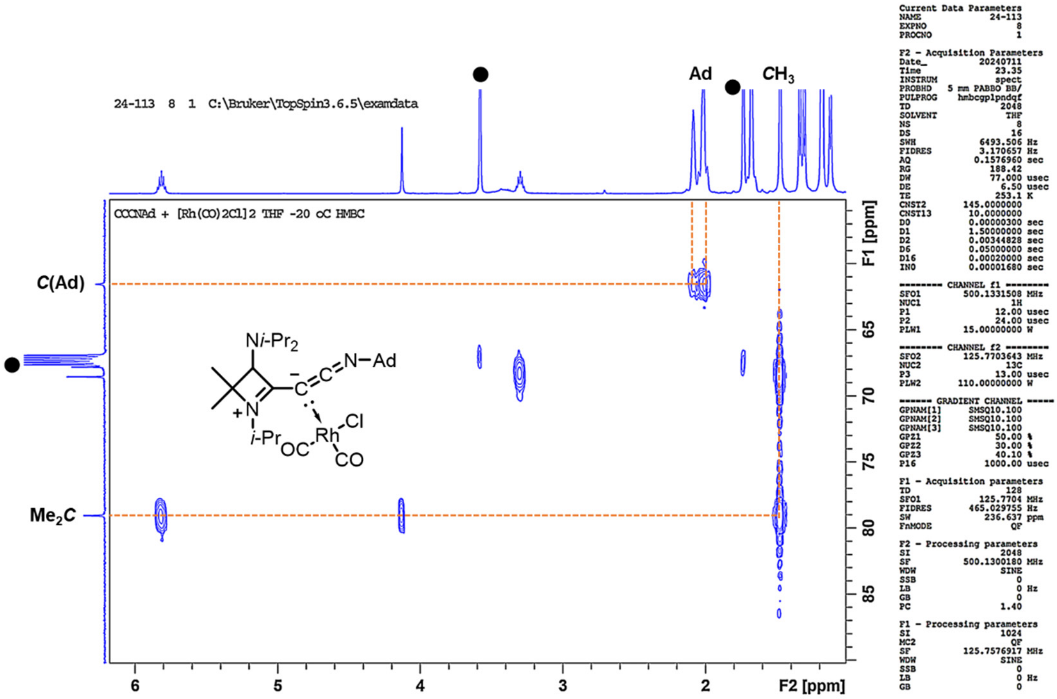

Figure S91. ^{1}H-^{13}C HMBC (magnified) NMR spectrum of **6** in THF-d_8 at 253 K (● = C_4D_7HO & C_4D_8O).

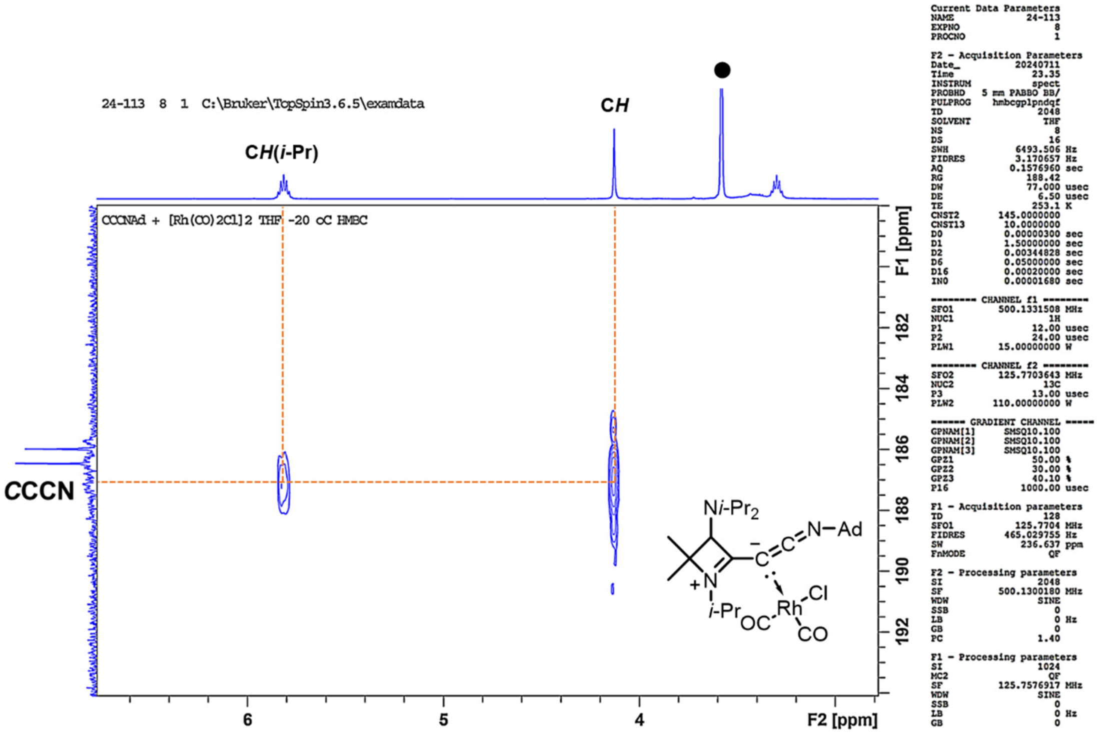

Figure S92. ^{1}H-^{13}C HMBC (magnified) NMR spectrum of **6** in THF-d_8 at 253 K (● = C_4D_7HO).

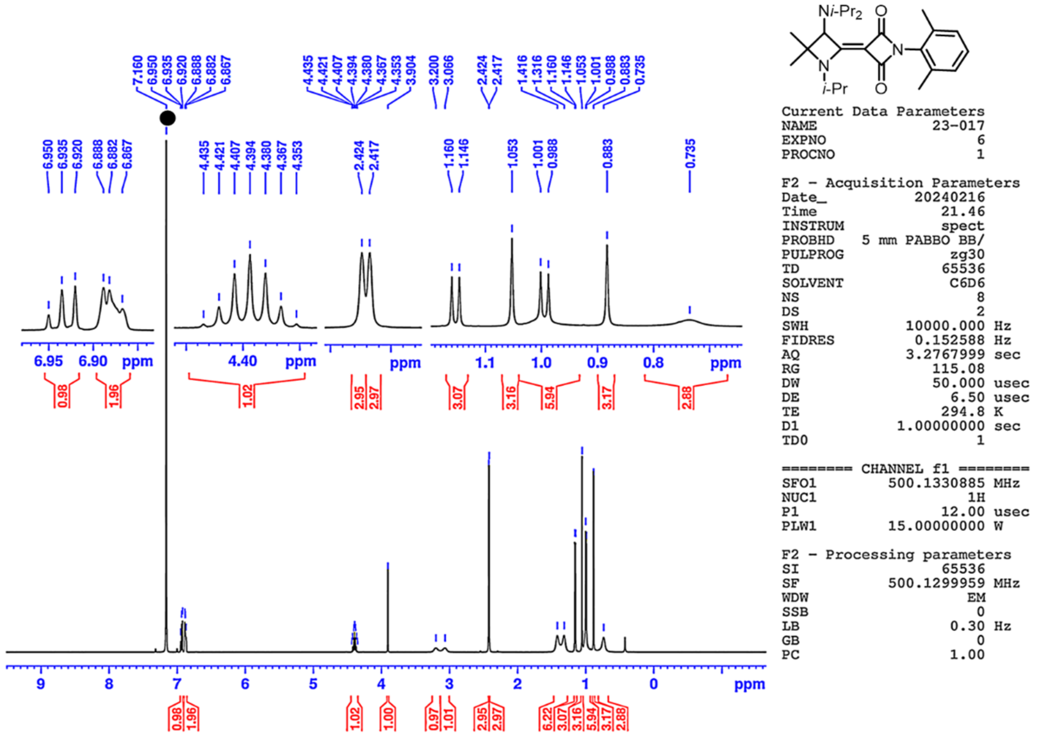

Figure S93. ^{1}H NMR spectrum of $\mathbf{7}_{\mathbf{Xyl}}$ in C_6D_6 at 295 K (● = C_6D_5H).

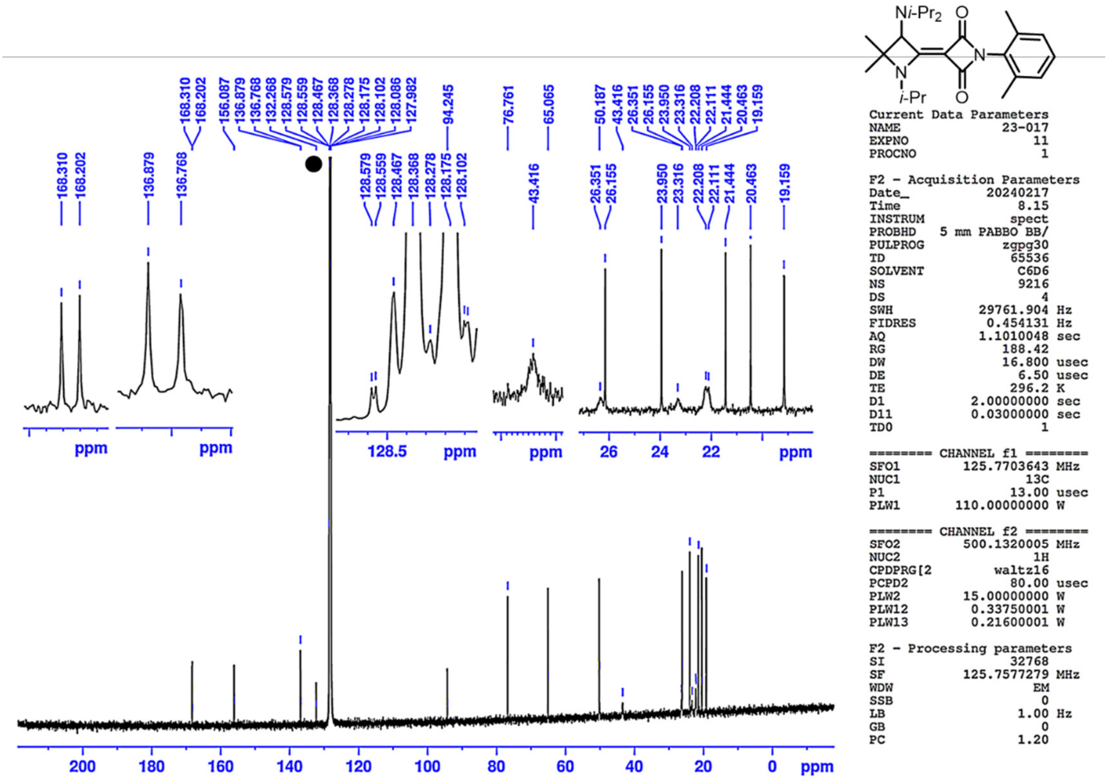

Figure S94. ^{13}C{^{1}H} NMR spectrum of $\mathbf{7}_{\mathbf{Xyl}}$ in C_6D_6 at 296 K (● = C_6D_6).

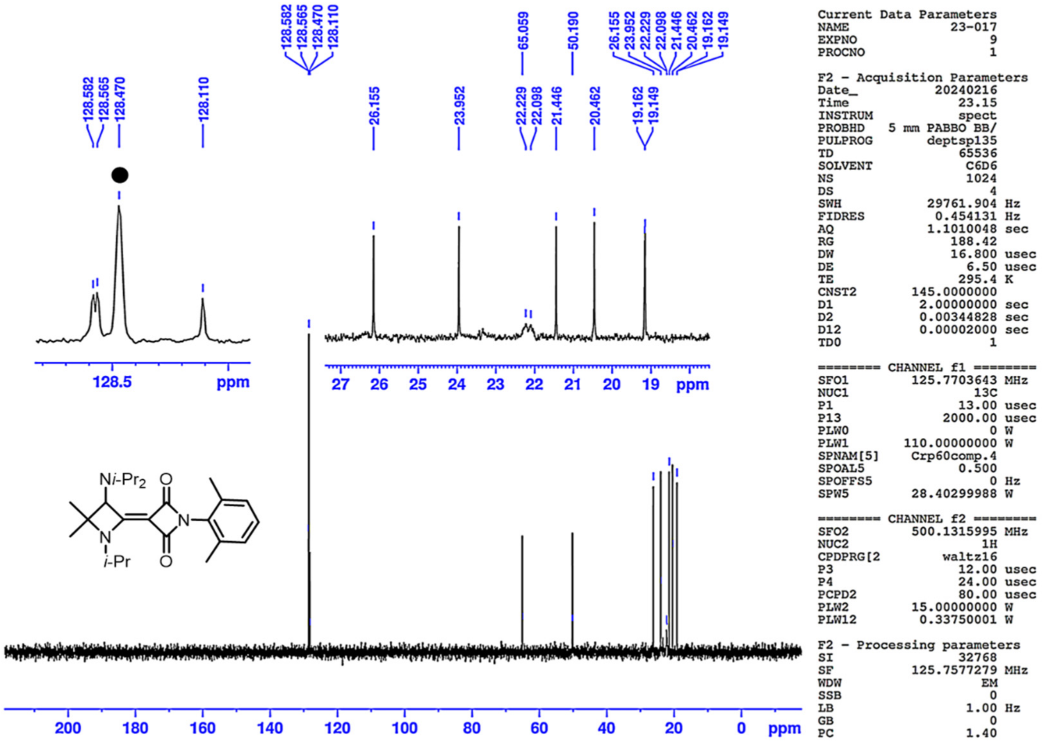

Figure S95. $^{13}C\{^{1}H\}$ (DEPT135) NMR spectrum of $\mathbf{7}_{Xyl}$ in C_6D_6 at 295 K (● = C_6D_5H).

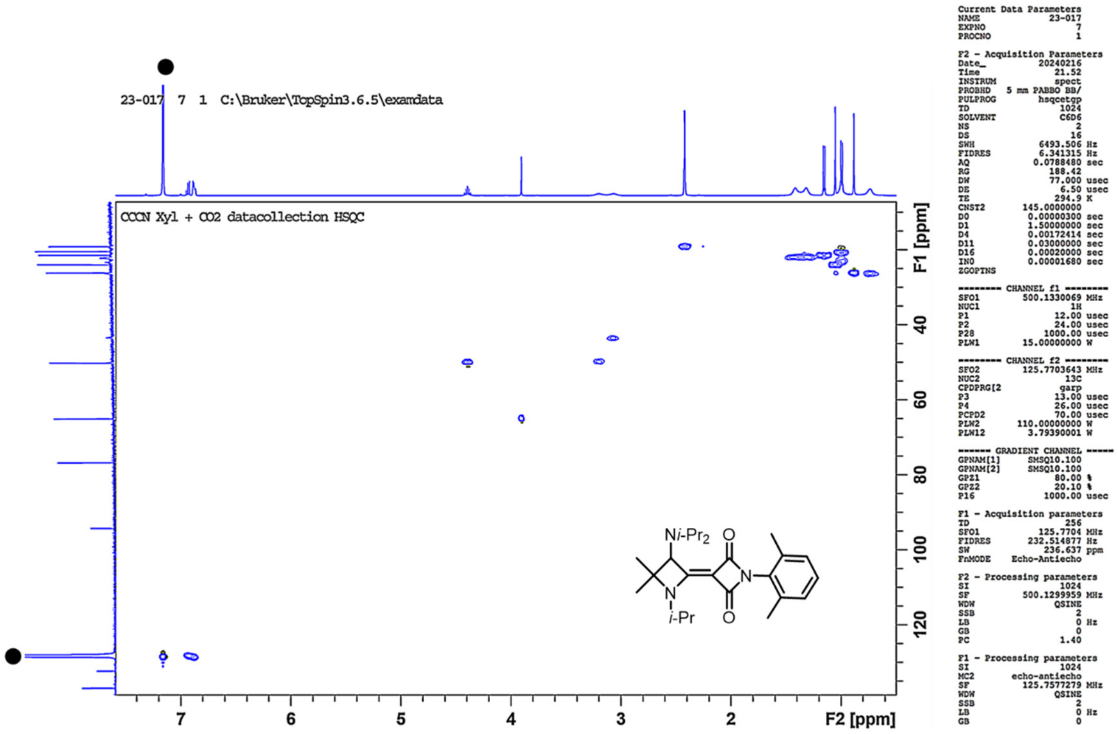

Figure S96. ^{1}H-^{13}C HSQC NMR spectrum of $\mathbf{7}_{Xyl}$ in C_6D_6 at 295 K (● = C_6D_5H & C_6D_6).

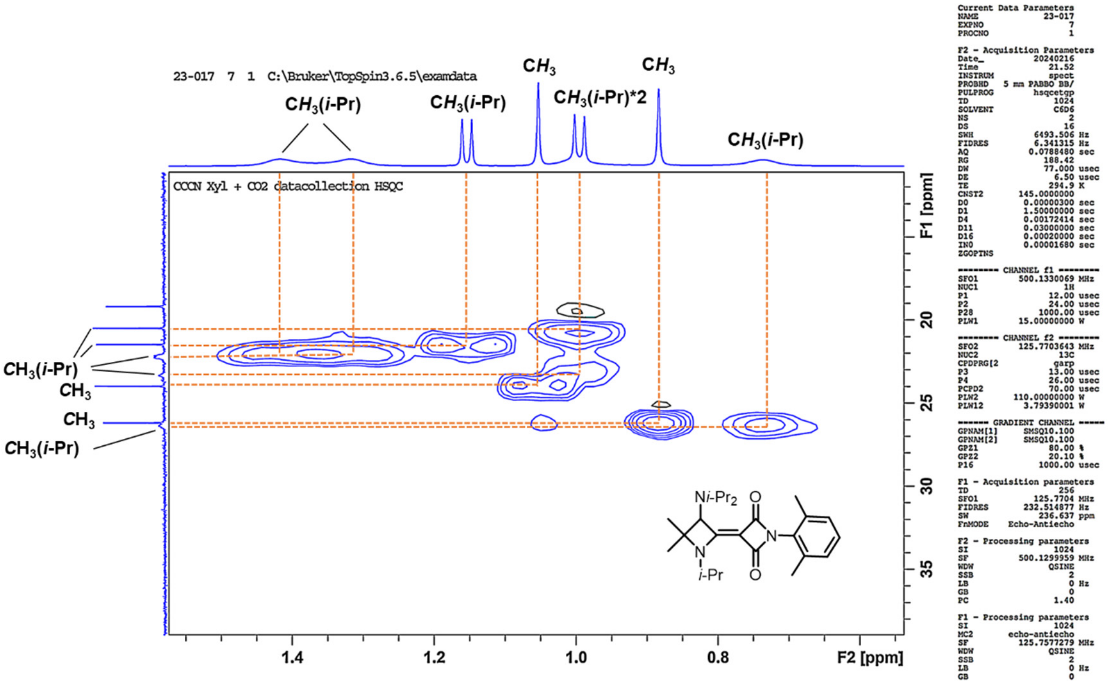

Figure S97. ^{1}H-^{13}C HSQC (magnified) NMR spectrum of $\mathbf{7}_{\mathbf{Xyl}}$ in C_6D_6 at 295 K.

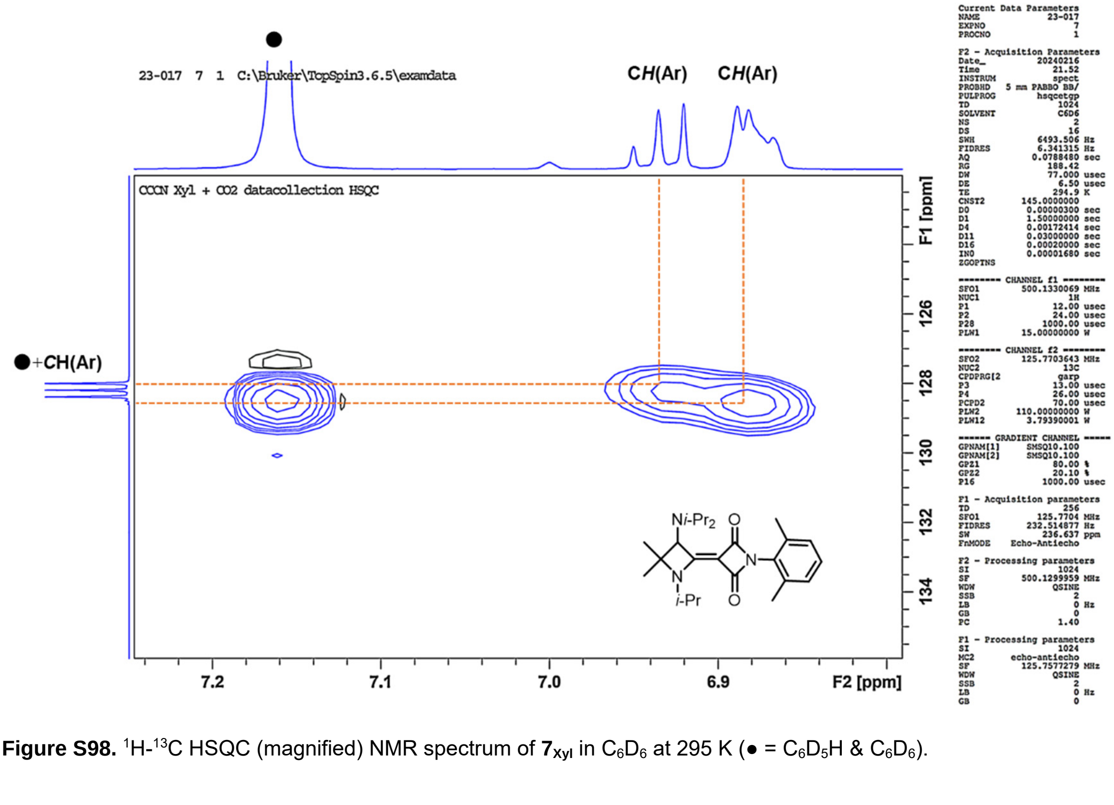

Figure S98. ^{1}H-^{13}C HSQC (magnified) NMR spectrum of $\mathbf{7}_{\mathbf{Xyl}}$ in C_6D_6 at 295 K (● = C_6D_5H & C_6D_6).

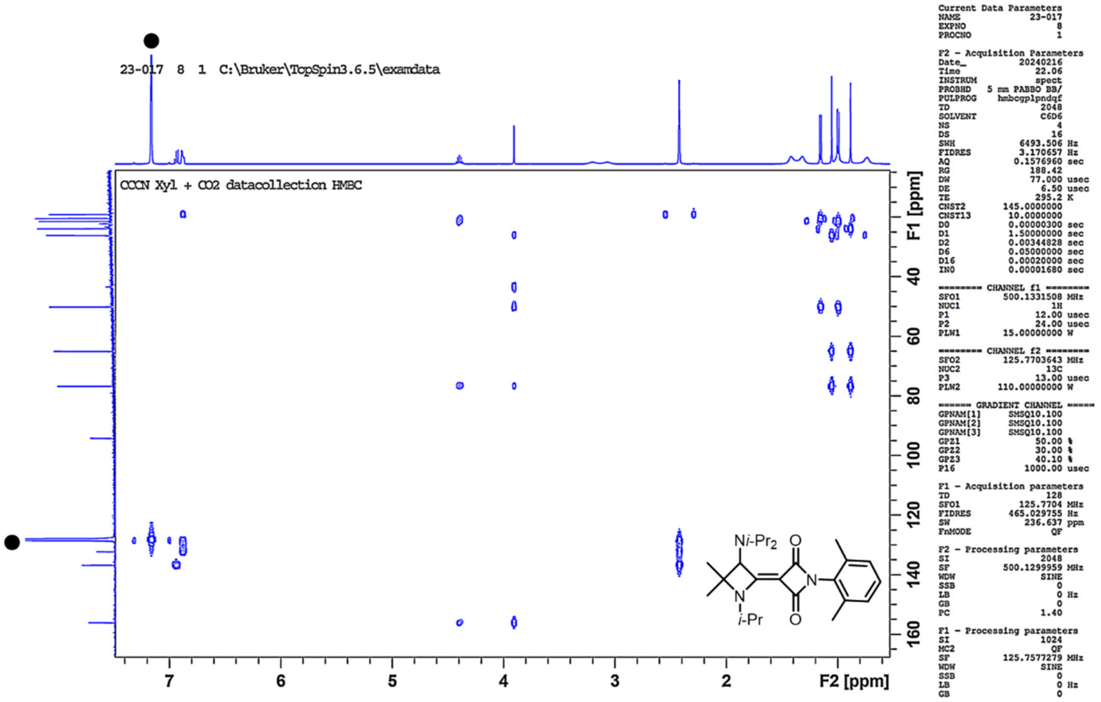

Figure S99. ^{1}H-^{13}C HMBC NMR spectrum of $\mathbf{7_{Xyl}}$ in C_6D_6 at 295 K (● = C_6D_5H & C_6D_6).

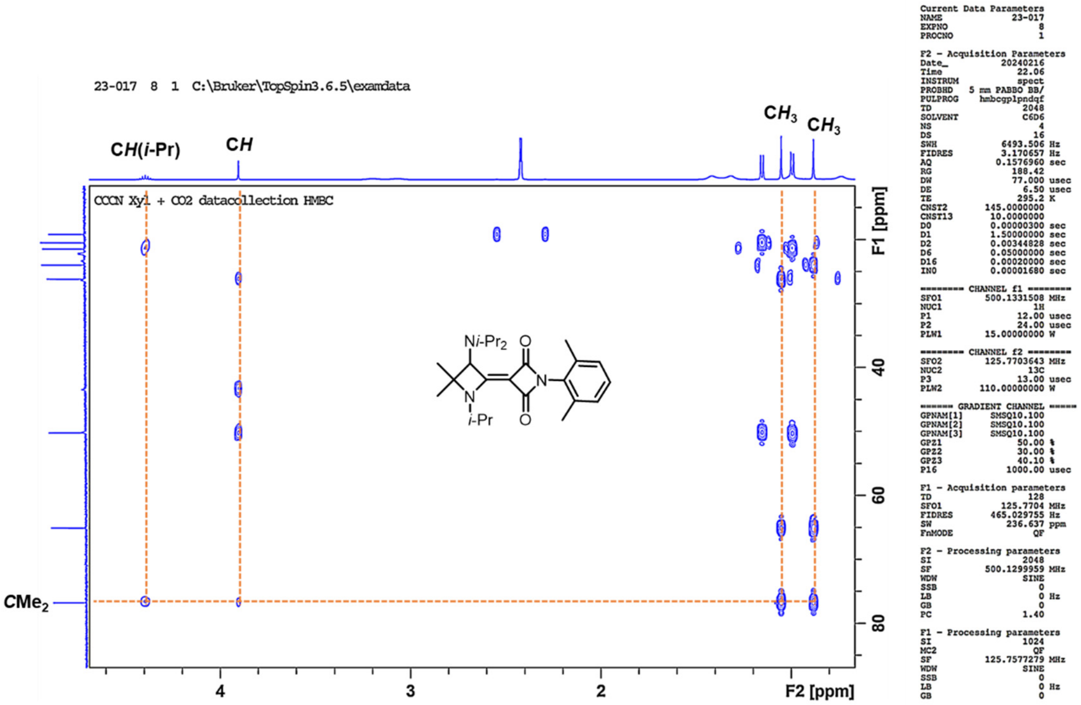

Figure S100. ^{1}H-^{13}C HMBC (magnified) NMR spectrum of $\mathbf{7_{Xyl}}$ in C_6D_6 at 295 K.

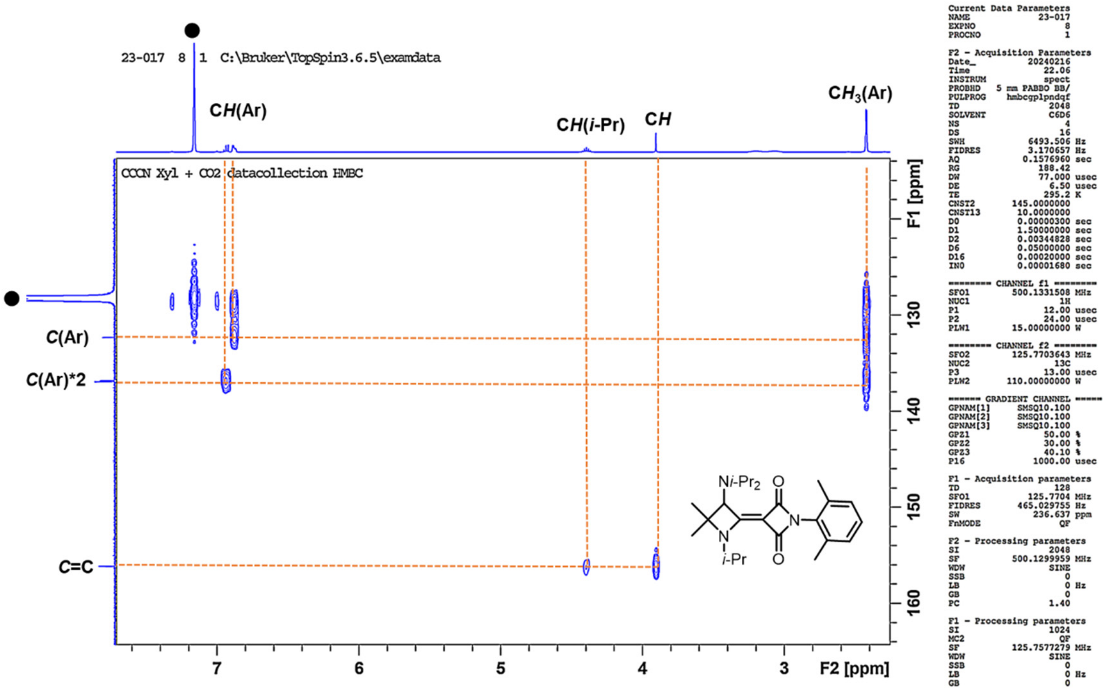

Figure S101. ^{1}H-^{13}C HMBC (magnified) NMR spectrum of $\mathbf{7_{Xyl}}$ in C_6D_6 at 295 K (● = C_6D_5H & C_6D_6).

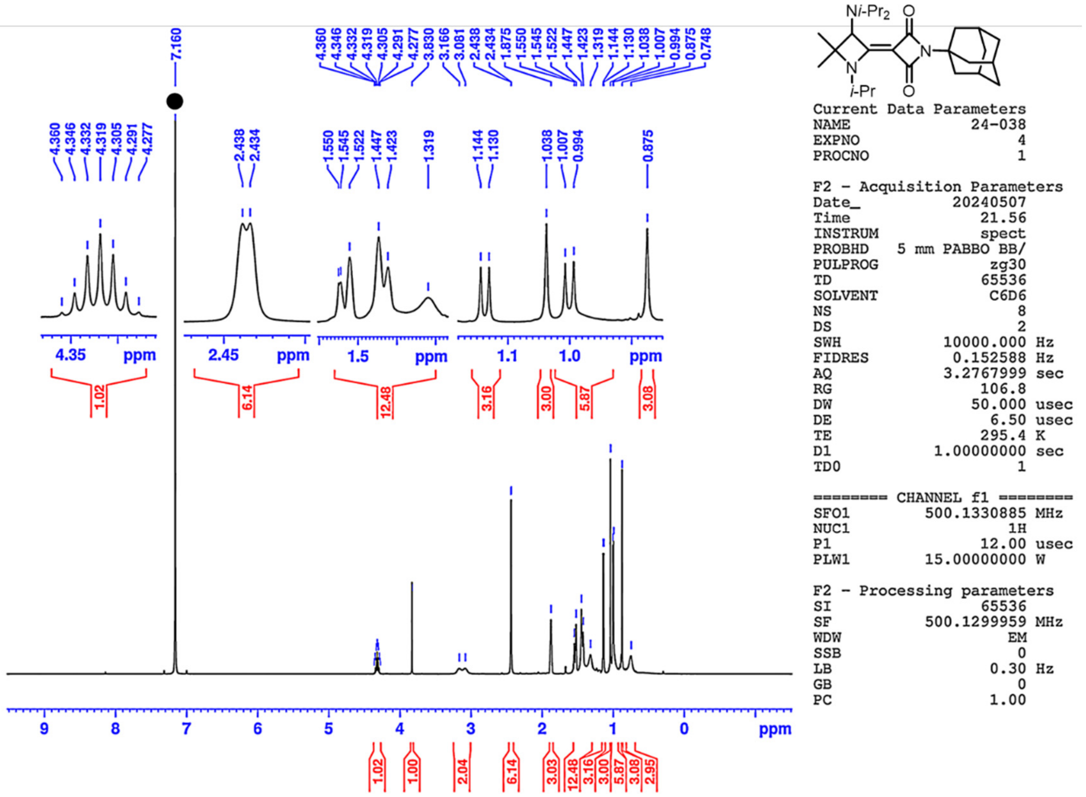

Figure S102. ^{1}H NMR spectrum of $\mathbf{7_{Ad}}$ in C_6D_6 at 295 K (● = C_6D_5H).

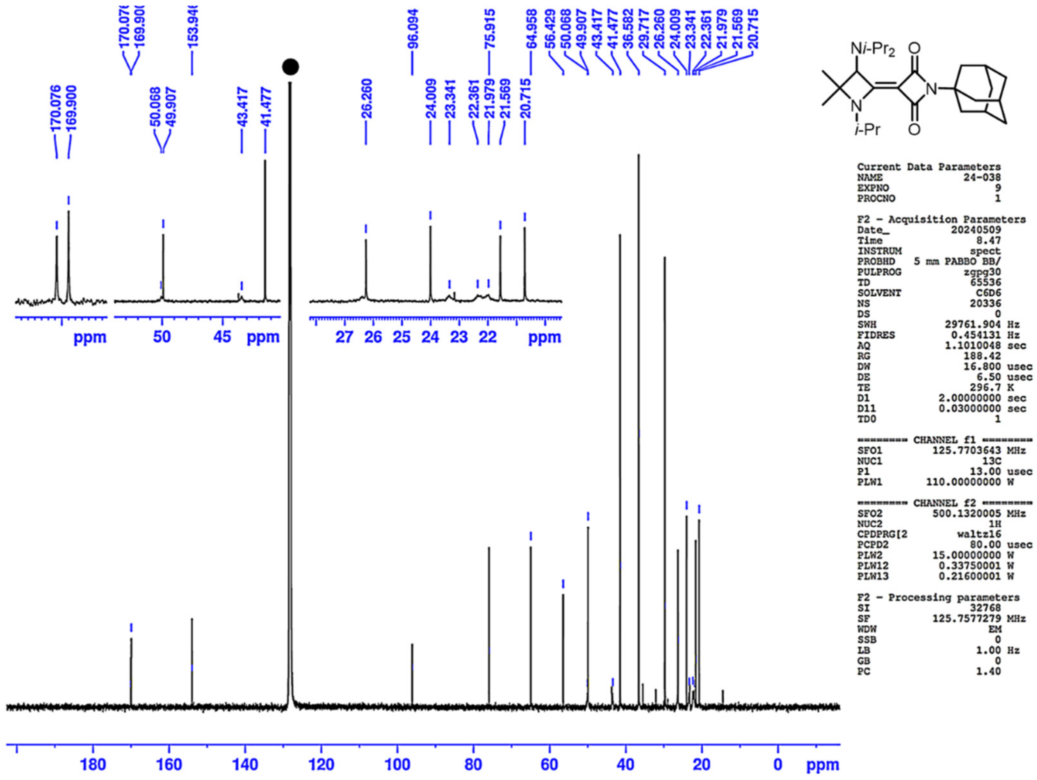

Figure S103. $^{13}C\{^1H\}$ NMR spectrum of $\mathbf{7_{Ad}}$ in C_6D_6 at 297 K (● = C_6D_6).

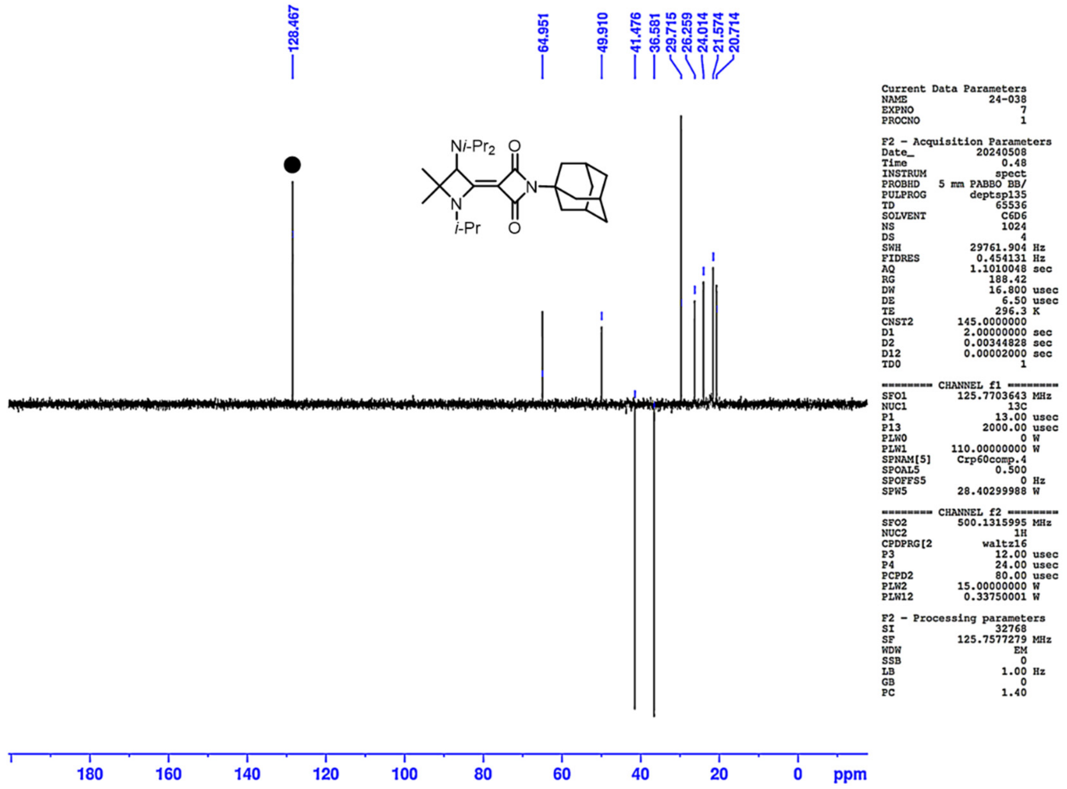

Figure S104. $^{13}C\{^1H\}$ (DEPT135) NMR spectrum of $\mathbf{7_{Ad}}$ in C_6D_6 at 296 K (● = C_6D_5H).

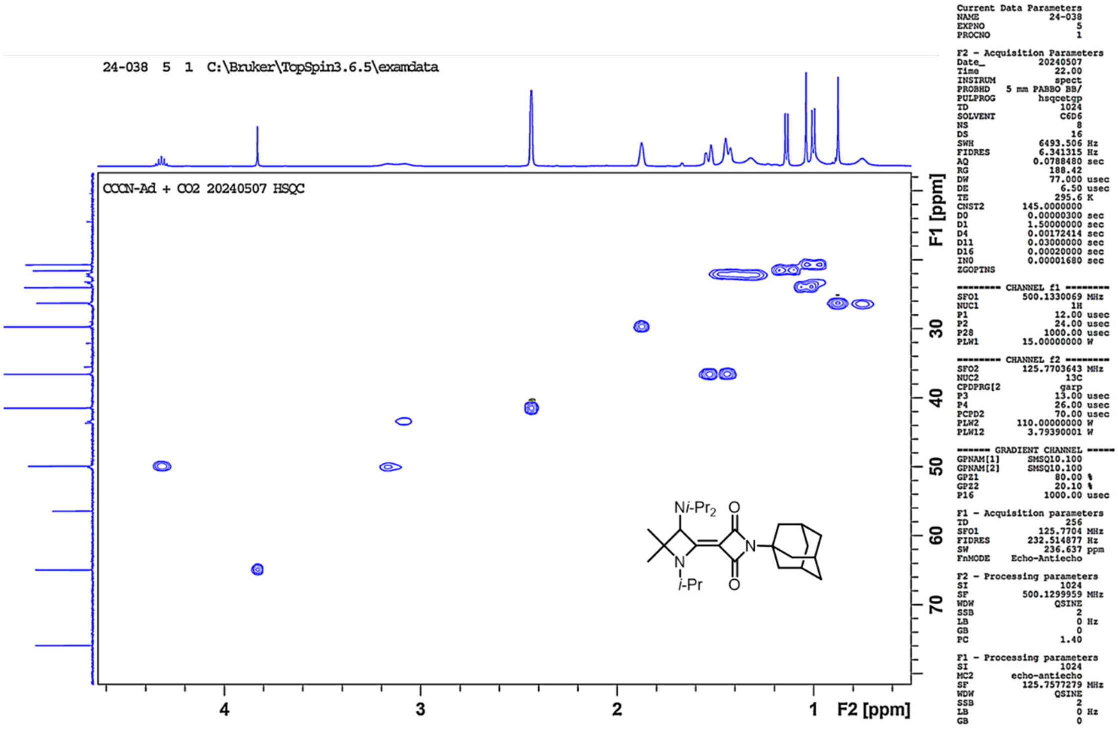

Figure S105. ^{1}H-^{13}C HSQC NMR spectrum of **7_{Ad}** in C_6D_6 at 296 K.

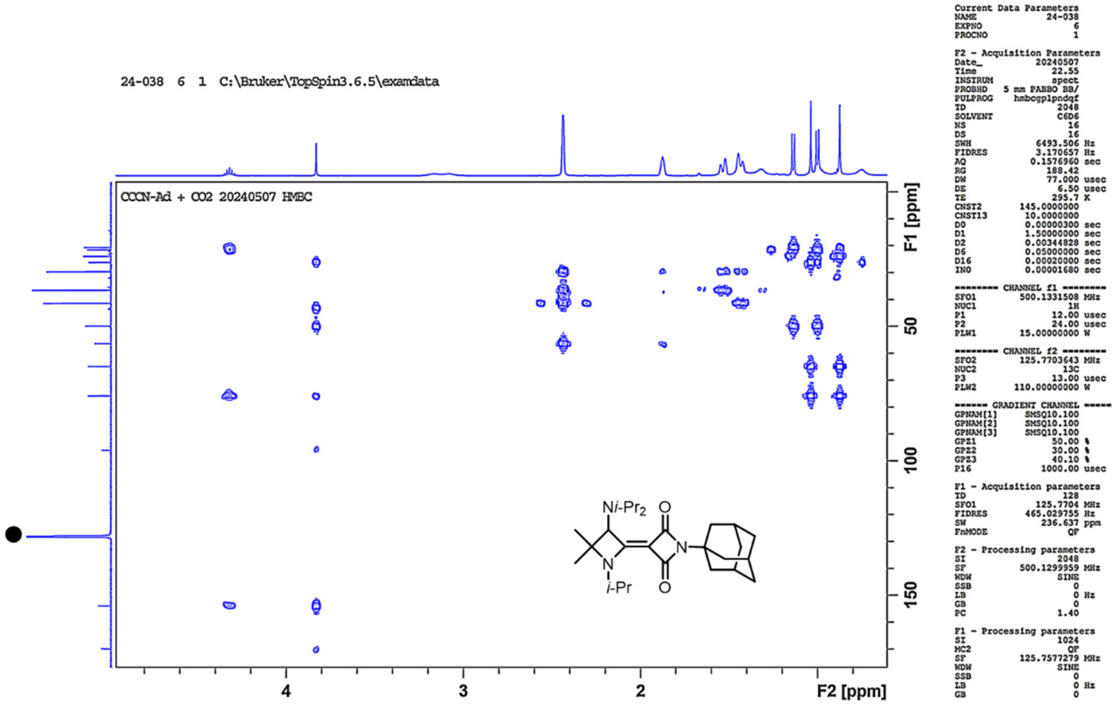

Figure S106. ^{1}H-^{13}C HMBC NMR spectrum of **7_{Ad}** in C_6D_6 at 296 K (● = C_6D_6).

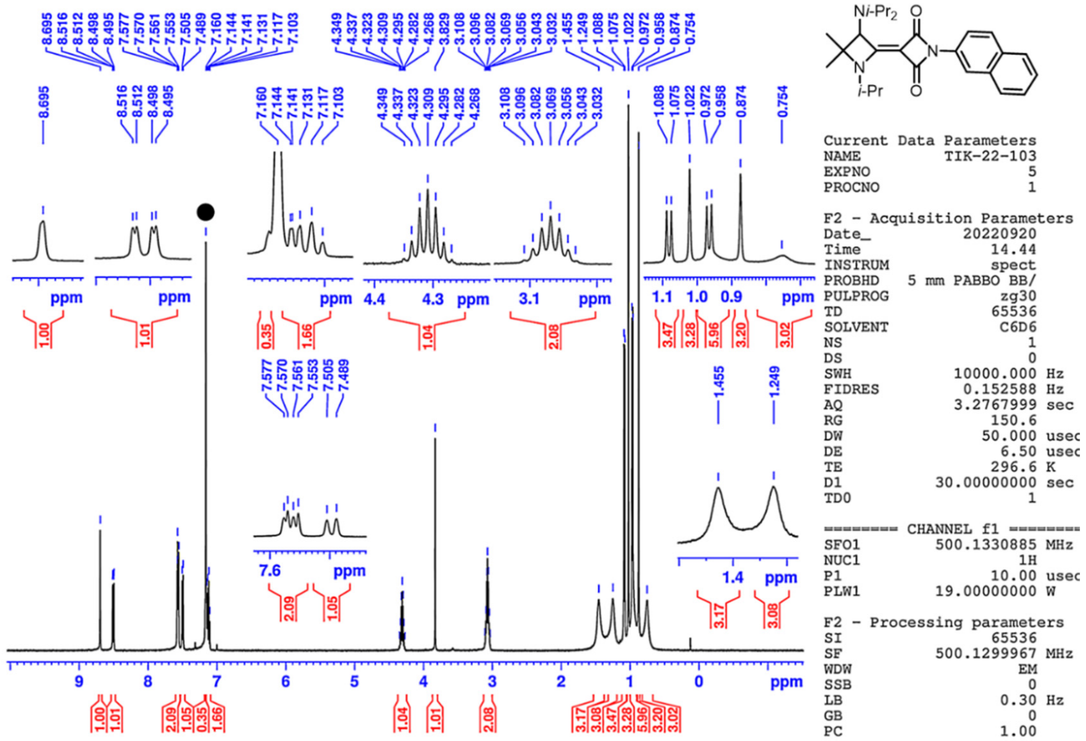

Figure S107. ^{1}H NMR spectrum of $\mathbf{7_{Naph}}$ in C_6D_6 at 297 K (● = C_6D_5H).

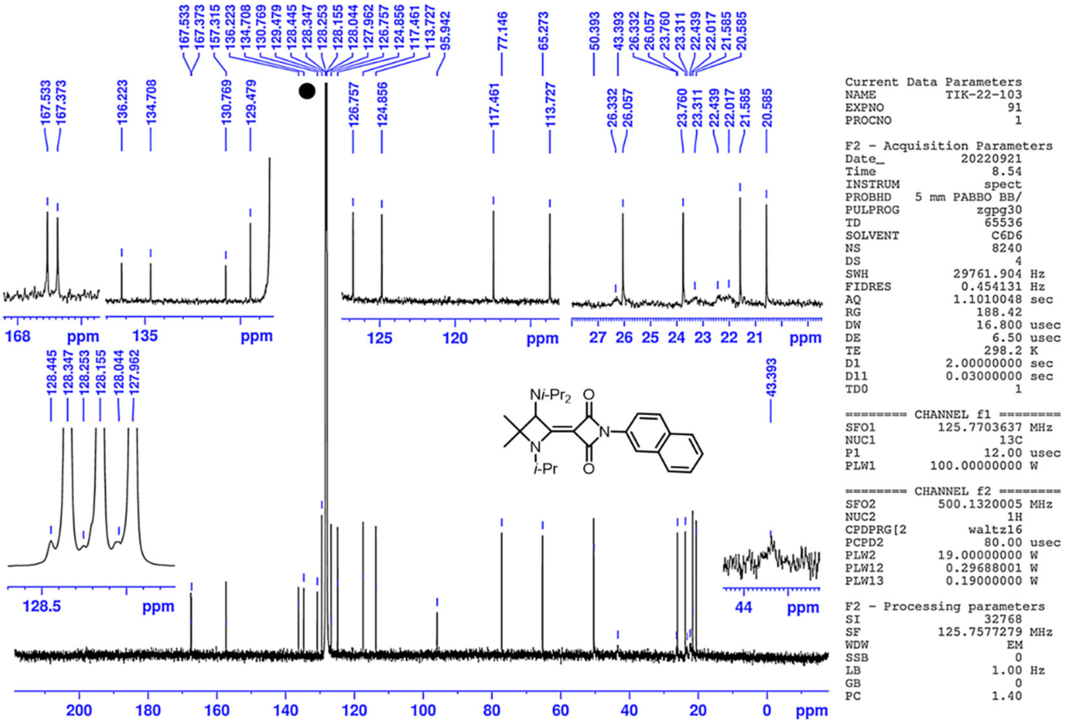

Figure S108. $^{13}C\{^{1}H\}$ NMR spectrum of $\mathbf{7_{Naph}}$ in C_6D_6 at 298 K (● = C_6D_6).

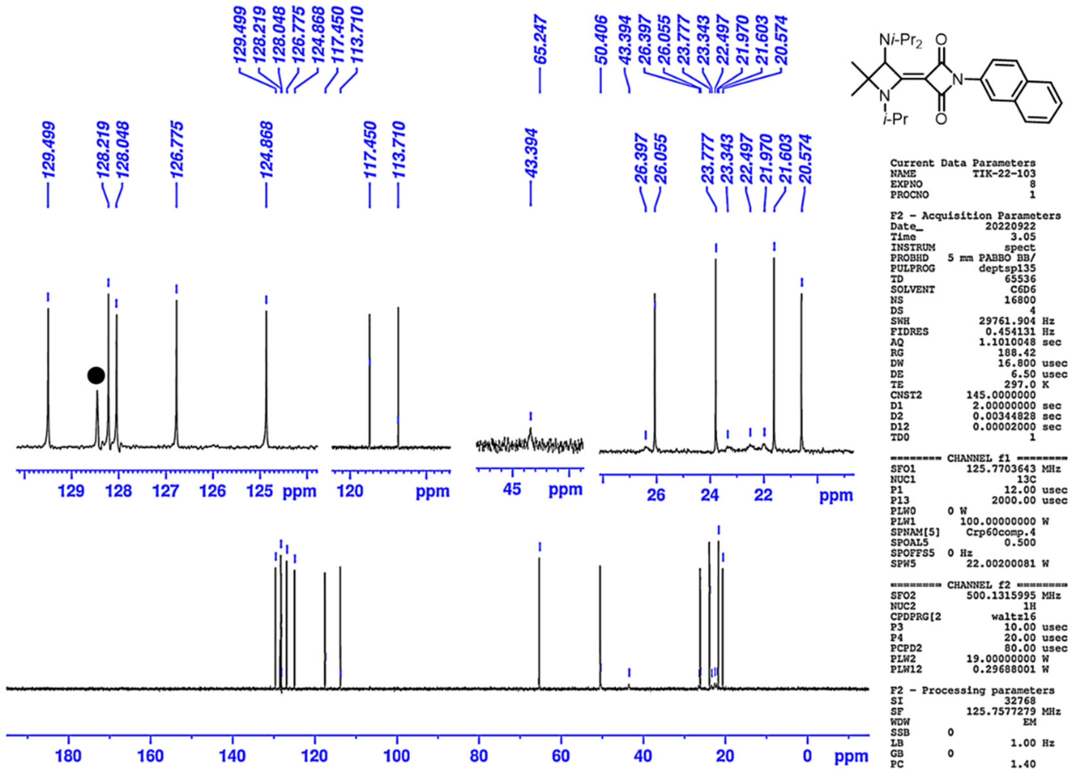

Figure S109. $^{13}C\{^{1}H\}$ (DEPT135) NMR spectrum of $\mathbf{7}_{\mathbf{Naph}}$ in C_6D_6 at 297 K (● = C_6D_5H).

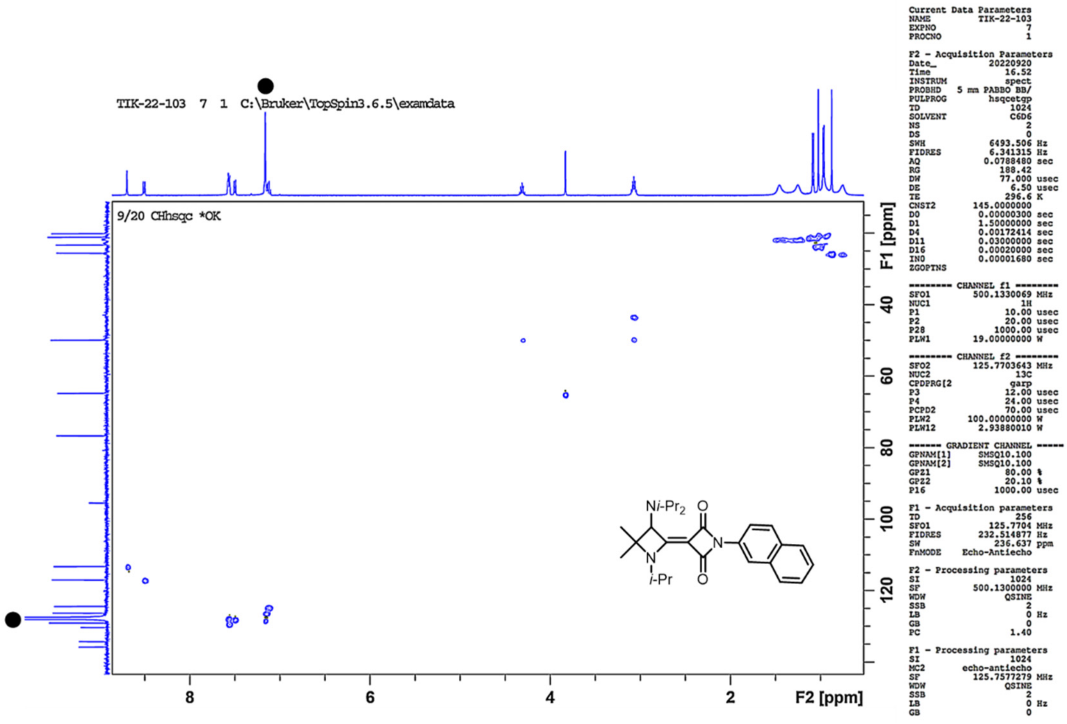

Figure S110. ^{1}H-^{13}C HSQC NMR spectrum of $\mathbf{7}_{\mathbf{Naph}}$ in C_6D_6 at 297 K (● = C_6D_5H & C_6D_6).

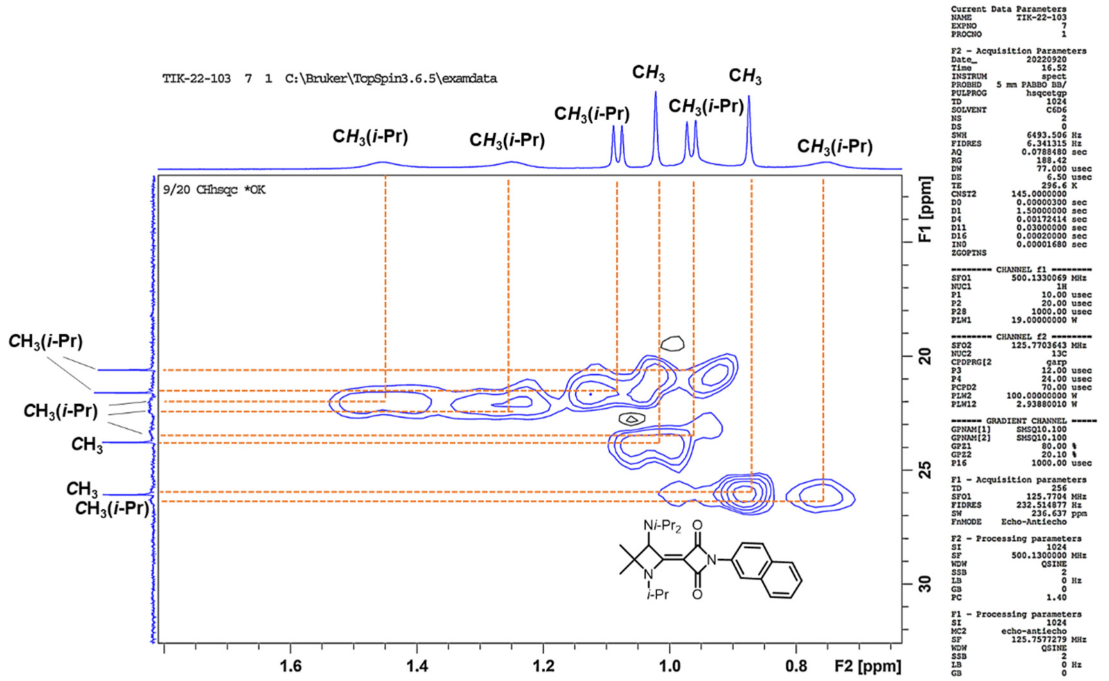

Figure S111. ^{1}H-^{13}C HSQC (magnified) NMR spectrum of $\mathbf{7_{Naph}}$ in C_6D_6 at 297 K.

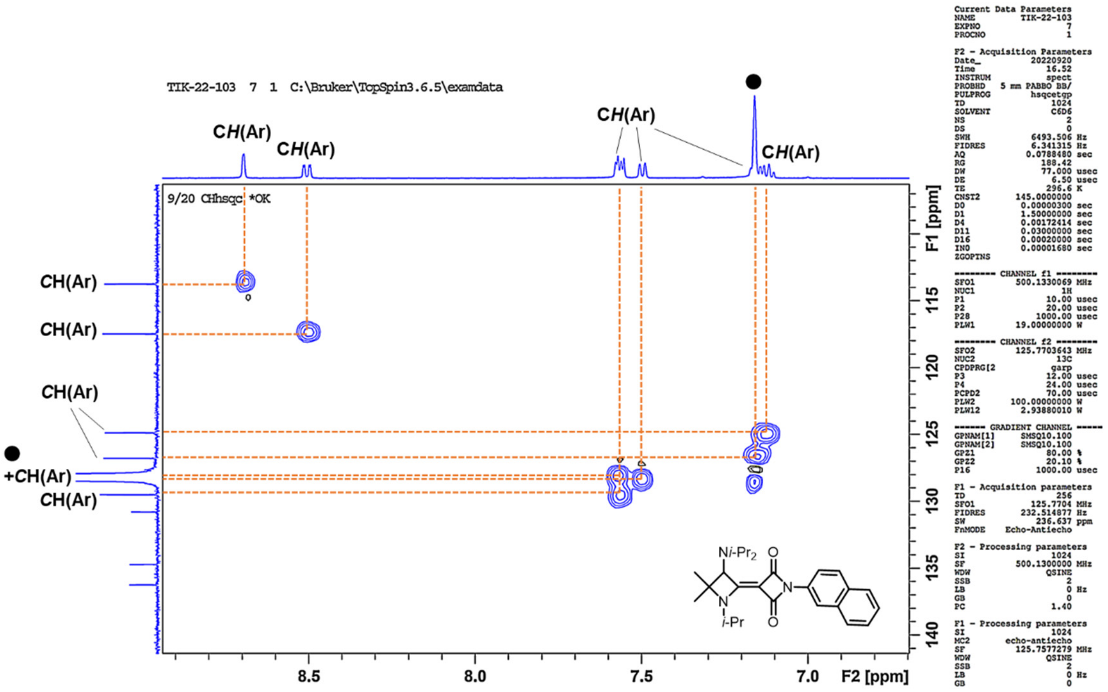

Figure S112. ^{1}H-^{13}C HSQC (magnified) NMR spectrum of $\mathbf{7_{Naph}}$ in C_6D_6 at 297 K (● = C_6D_5H & C_6D_6).

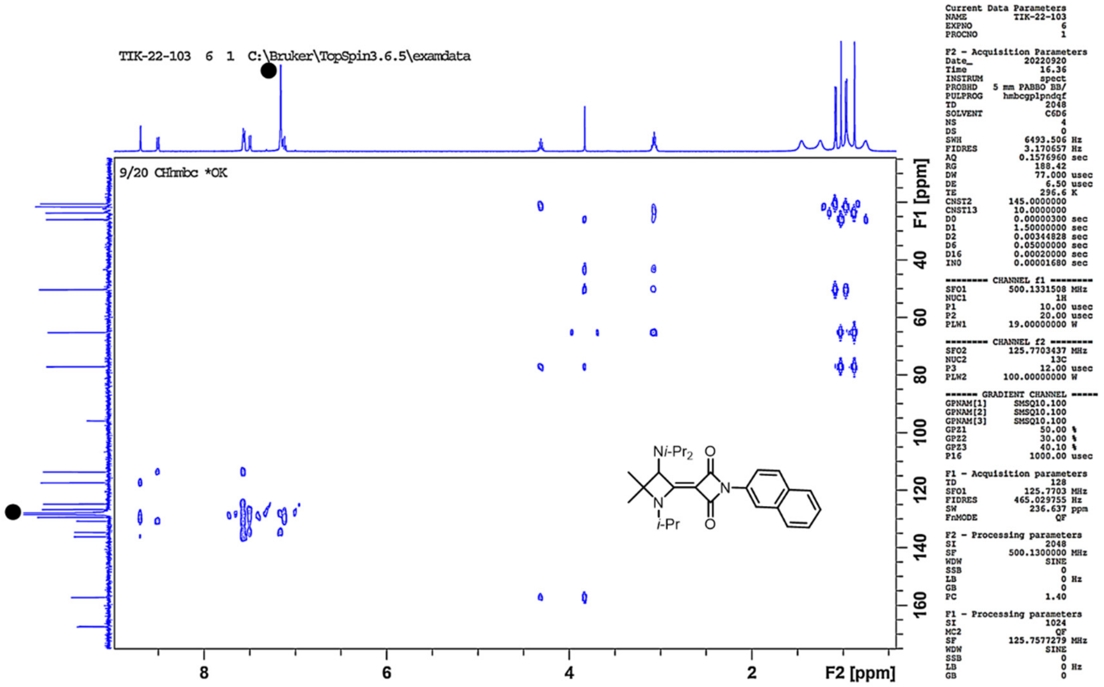

Figure S113. ^{1}H-^{13}C HMBC NMR spectrum of $\mathbf{7}_{\mathbf{Naph}}$ in C_6D_6 at 297 K (● = C_6D_5H & C_6D_6).

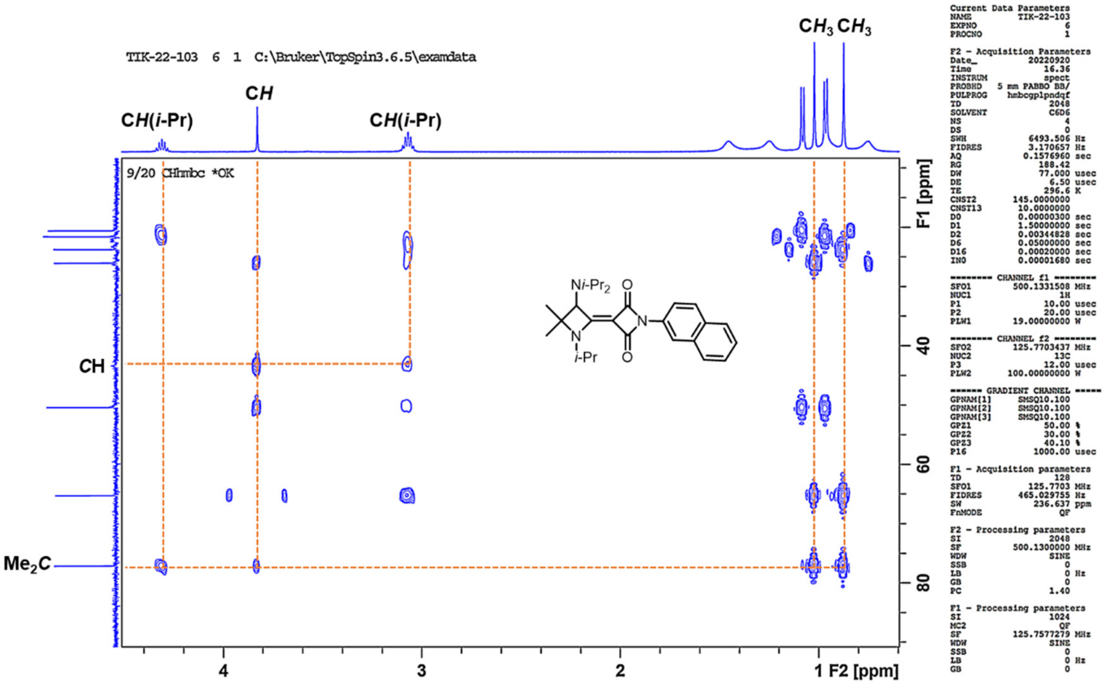

Figure S114. ^{1}H-^{13}C HMBC (magnified) NMR spectrum of $\mathbf{7}_{\mathbf{Naph}}$ in C_6D_6 at 297 K.

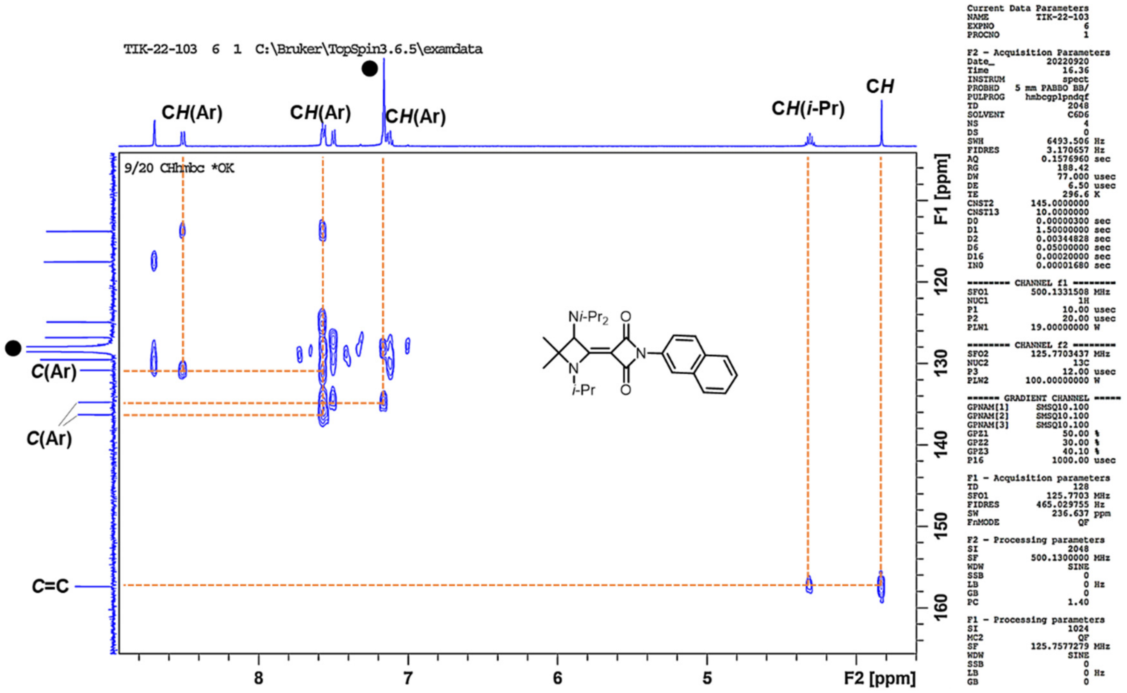

Figure S115. ^{1}H-^{13}C HMBC (magnified) NMR spectrum of $\mathbf{7}_{\mathbf{Naph}}$ in C_6D_6 at 297 K (● = C_6D_5H & C_6D_6).

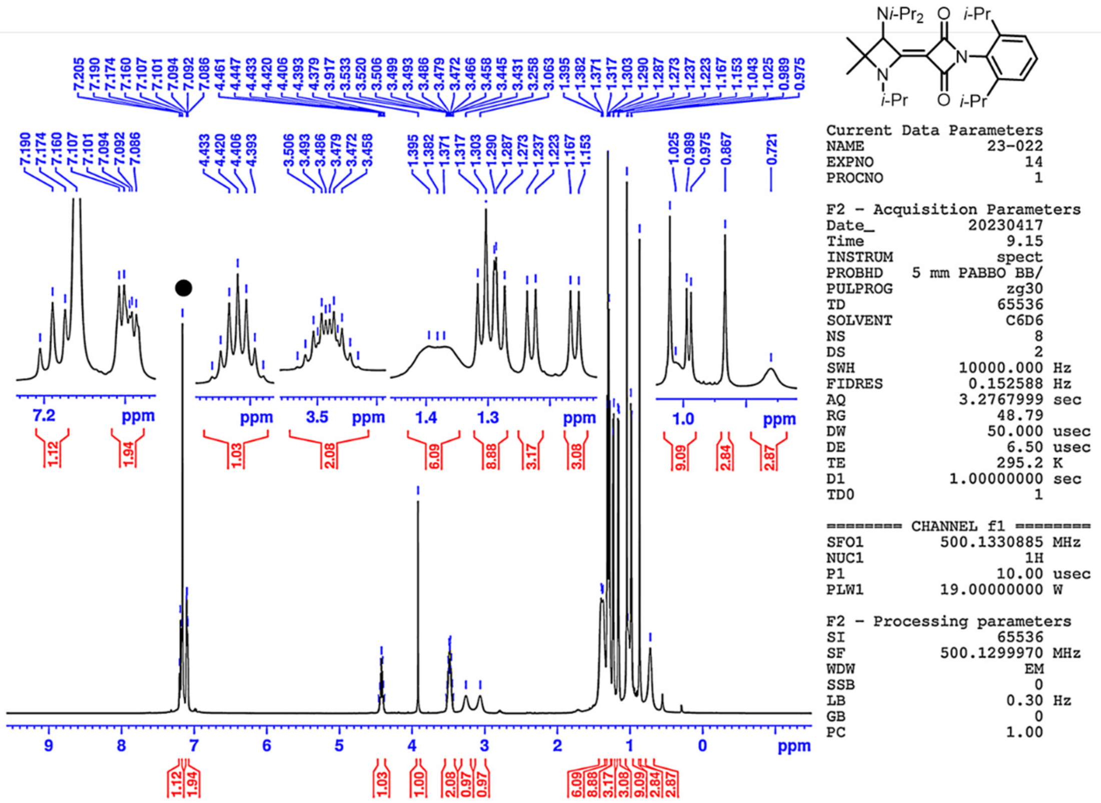

Figure S116. ^{1}H NMR spectrum of $\mathbf{7}_{\mathbf{Dipp}}$ in C_6D_6 at 295 K (● = C_6D_5H).

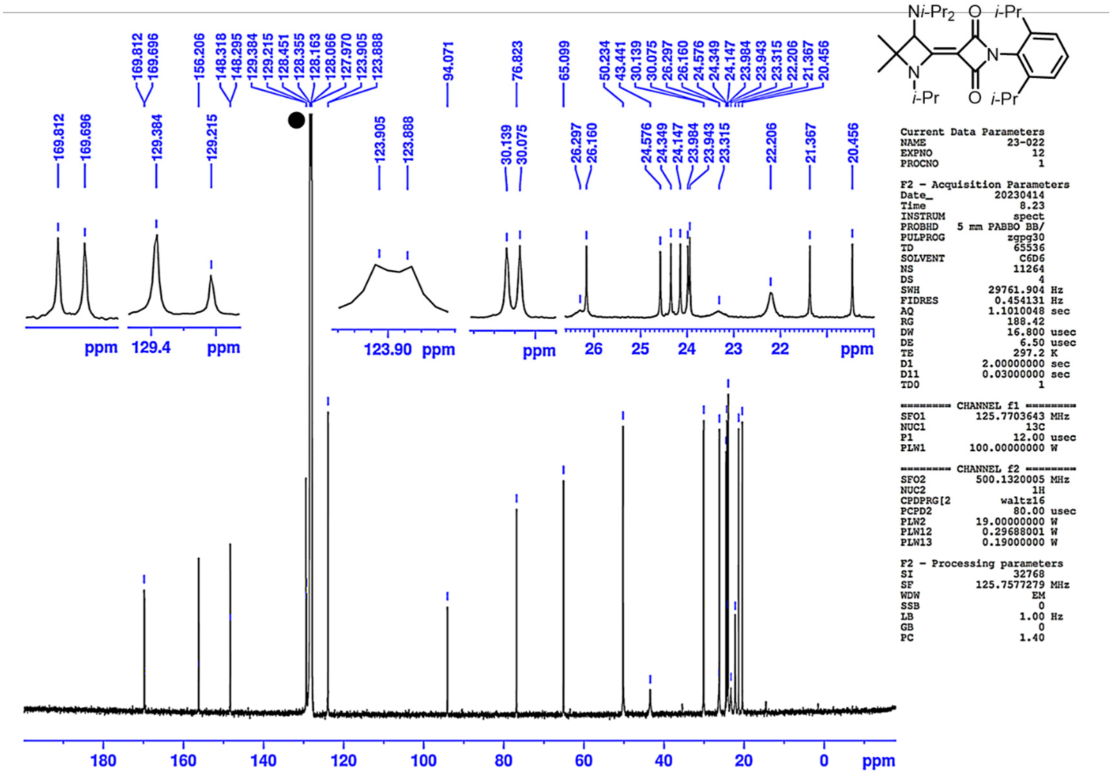

Figure S117. $^{13}C\{^{1}H\}$ NMR spectrum of $\mathbf{7}_{\mathbf{Dipp}}$ in C_6D_6 at 297 K (● = C_6D_6).

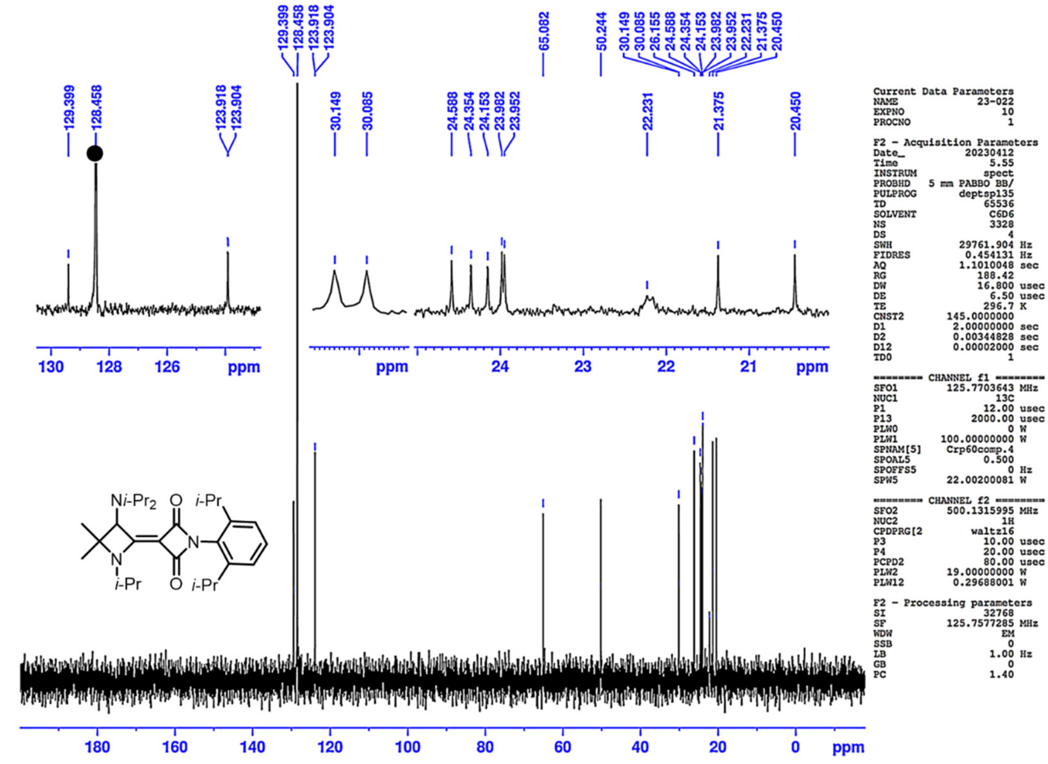

Figure S118. $^{13}C\{^{1}H\}$ (DEPT135) NMR spectrum of $\mathbf{7}_{\mathbf{Dipp}}$ in C_6D_6 at 297 K (● = C_6D_5H).

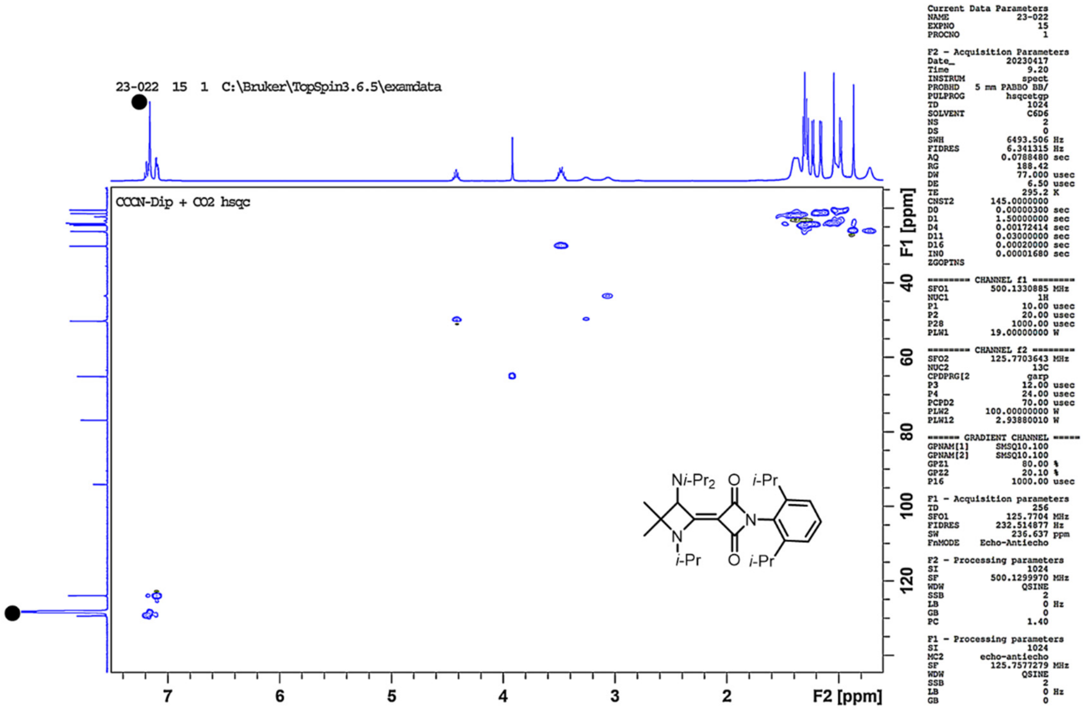

Figure S119. ^{1}H-^{13}C HSQC NMR spectrum of $\mathbf{7}_{\mathbf{Dipp}}$ in C_6D_6 at 295 K (● = C_6D_5H & C_6D_6).

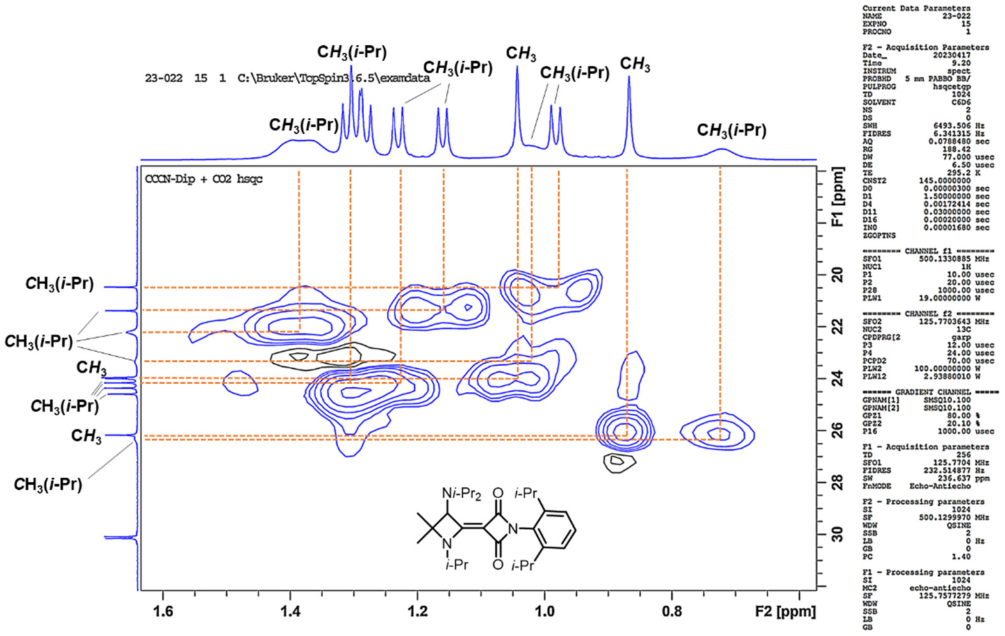

Figure S120. ^{1}H-^{13}C HSQC (magnified) NMR spectrum of $\mathbf{7}_{\mathbf{Dipp}}$ in C_6D_6 at 295 K.

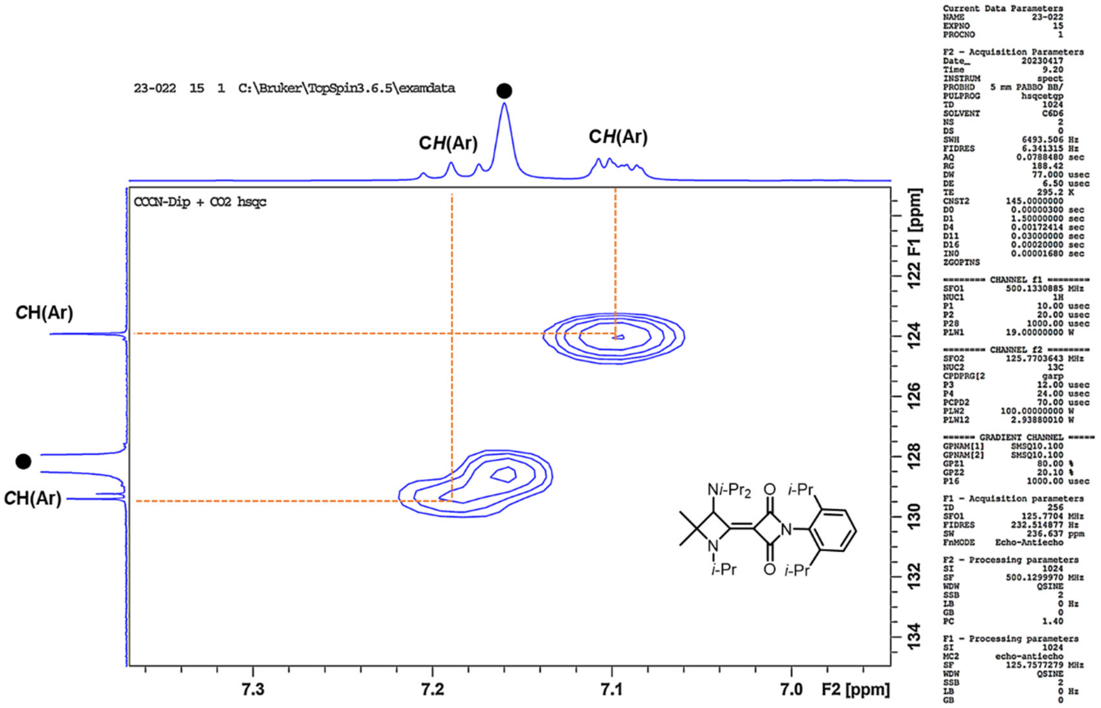

Figure S121. ^{1}H-^{13}C HSQC (magnified) NMR spectrum of $\mathbf{7_{Dipp}}$ in C_6D_6 at 295 K (● = C_6D_6).

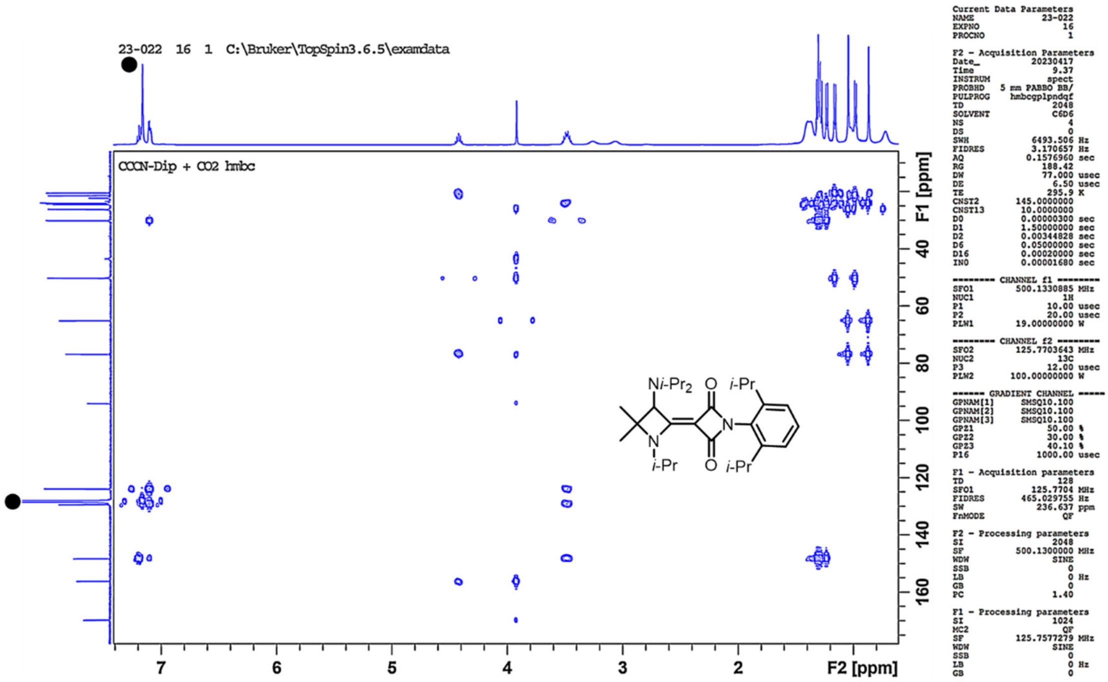

Figure S122. ^{1}H-^{13}C HMBC NMR spectrum of $\mathbf{7_{Dipp}}$ in C_6D_6 at 296 K (● = C_6D_5H & C_6D_6).

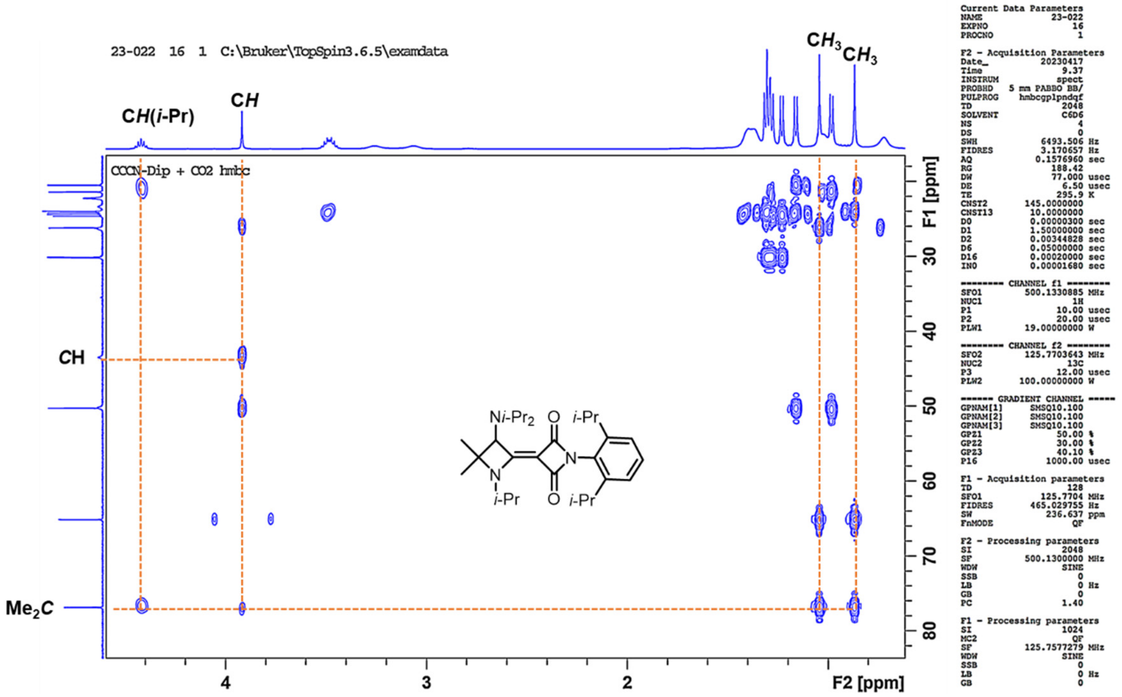

Figure S123. ^{1}H-^{13}C HMBC (magnified) NMR spectrum of $\mathbf{7_{Dipp}}$ in C_6D_6 at 296 K.

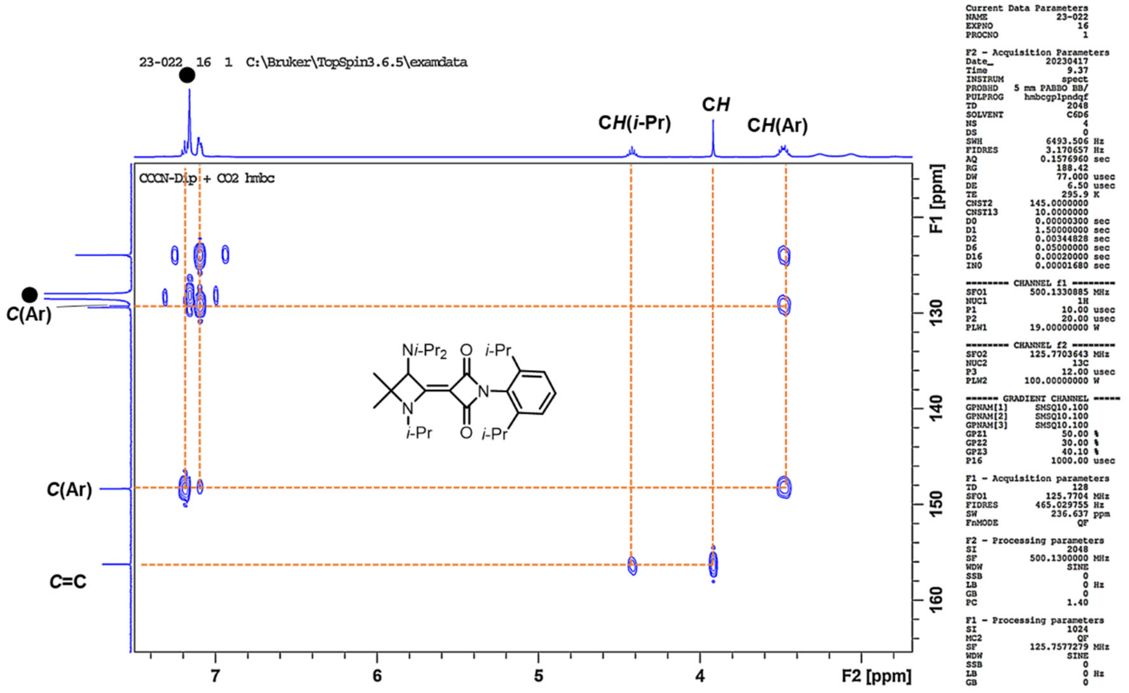

Figure S124. ^{1}H-^{13}C HMBC (magnified) NMR spectrum of $\mathbf{7_{Dipp}}$ in C_6D_6 at 296 K (● = C_6D_5H & C_6D_6).

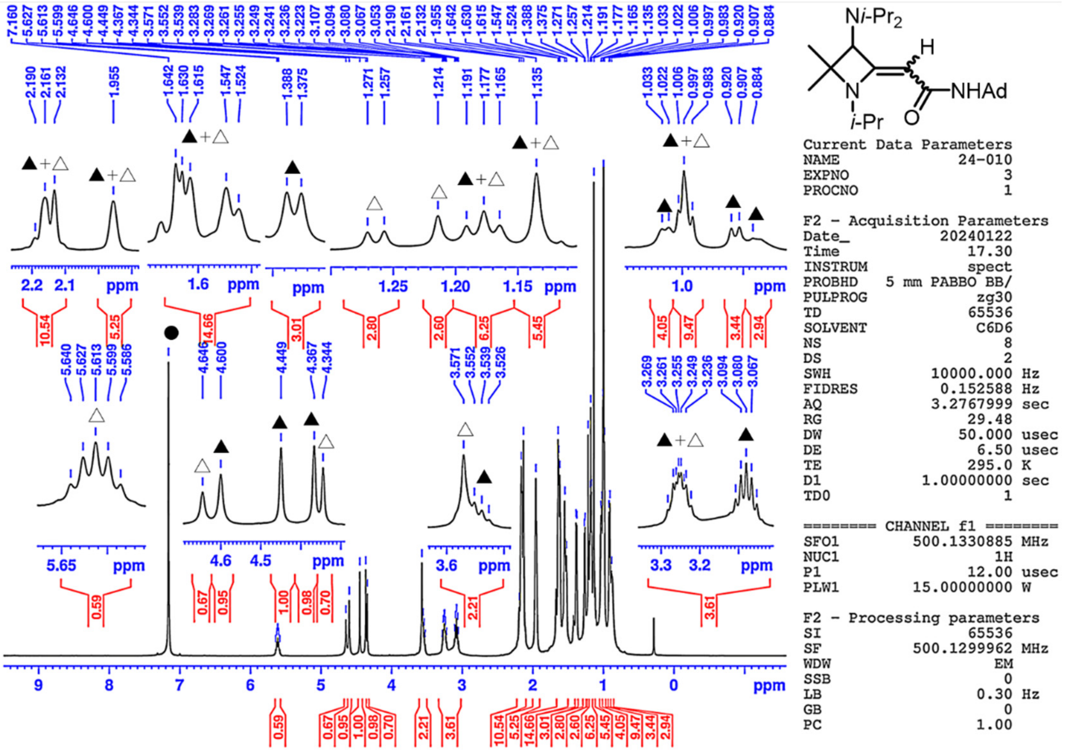

Figure S125. ^{1}H NMR spectrum of **13** in C_6D_6 at 295 K (● = C_6D_5H, △= (*E*)-**13**, ▲= (*Z*)-**13**).

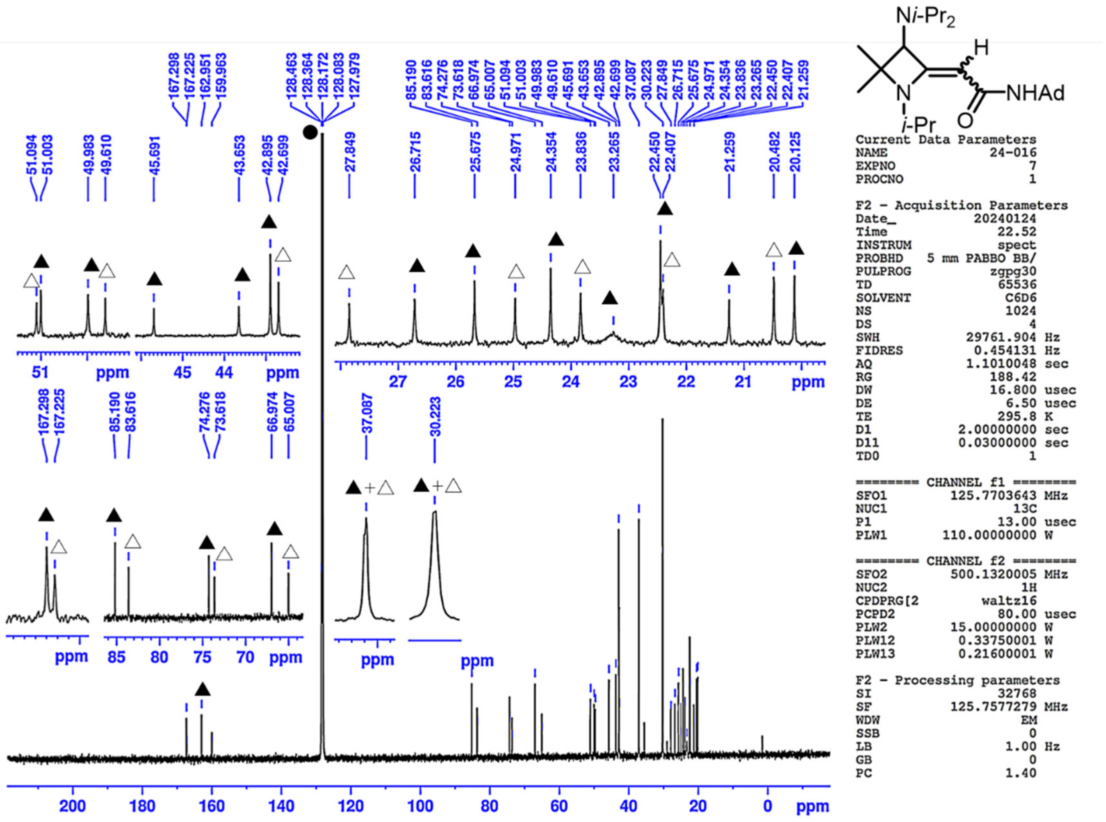

Figure S126. $^{13}C\{^{1}H\}$ NMR spectrum of **13** in C_6D_6 at 296 K (● = C_6D_6, △= (*E*)-**13**, ▲= (*Z*)-**13**).

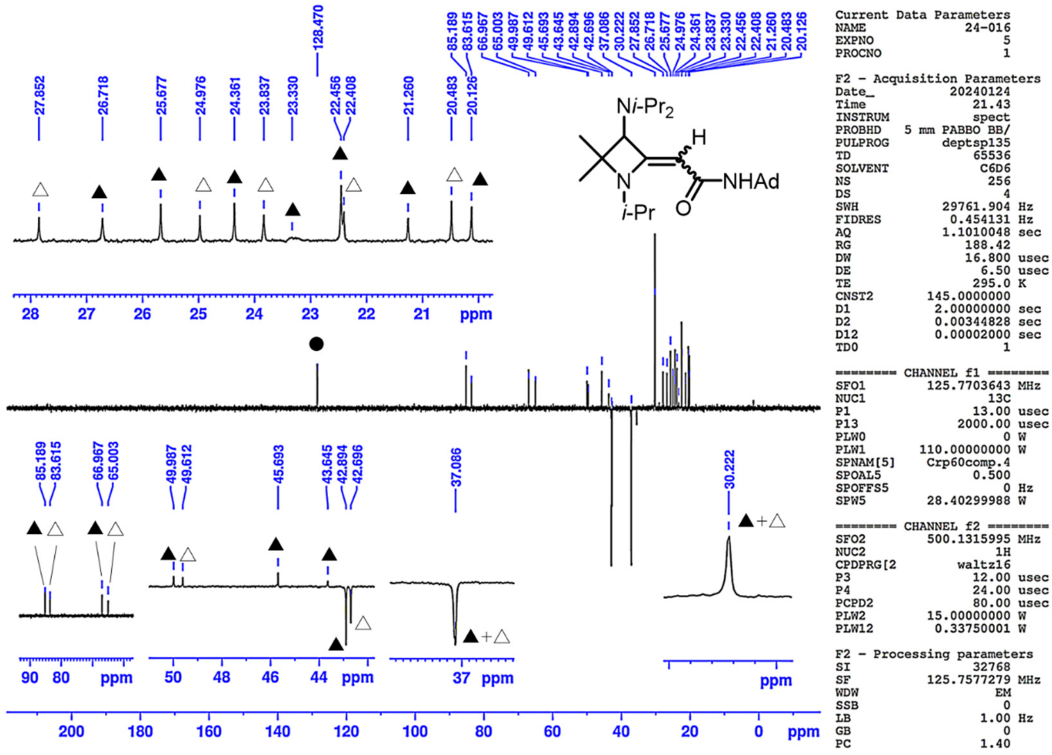

Figure S127. $^{13}C\{^1H\}$ (DEPT135) NMR spectrum of **13** in C_6D_6 at 295 K (● = C_6D_5H, △= (*E*)-**13**, ▲= (*Z*)-**13**).

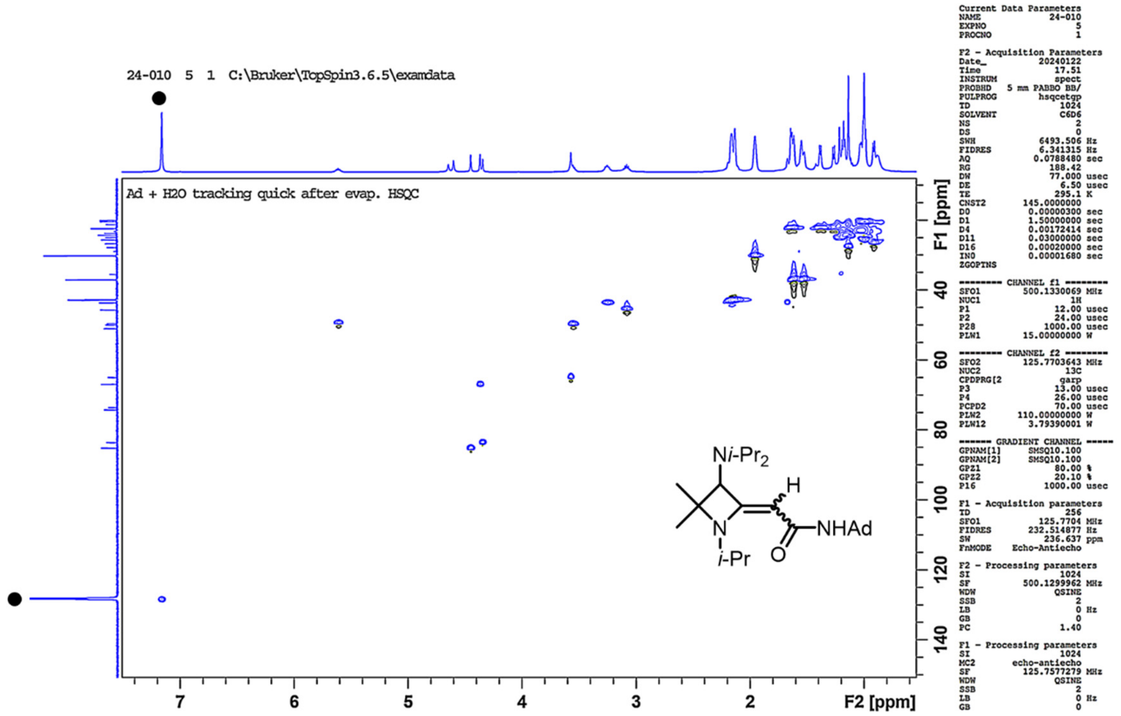

Figure S128. ^1H-^{13}C HSQC NMR spectrum of **13** in C_6D_6 at 295 K (● = C_6D_5H & C_6D_6).

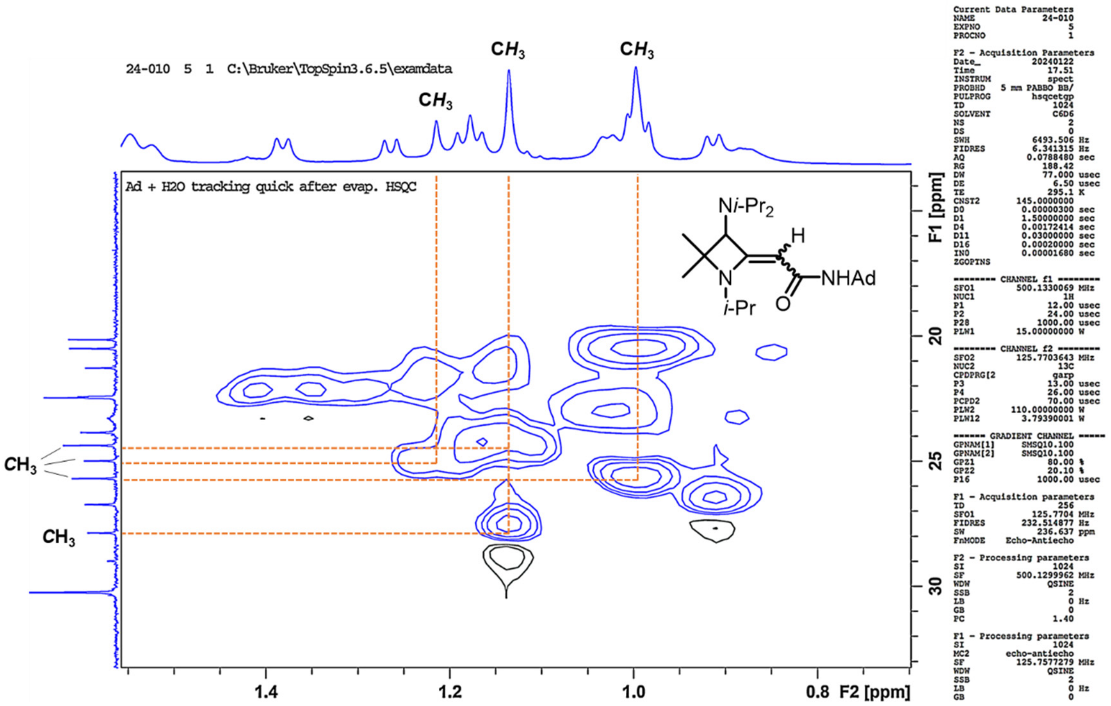

Figure S129. ^{1}H-^{13}C HSQC (magnified) NMR spectrum of **13** in C_6D_6 at 295 K.

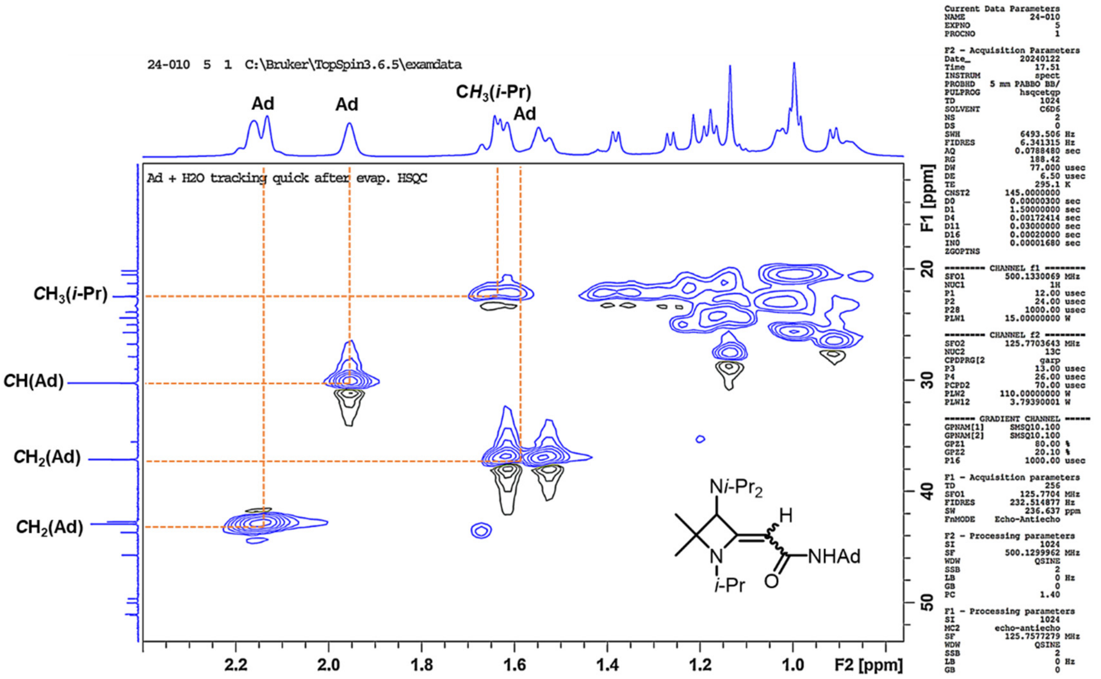

Figure S130. ^{1}H-^{13}C HSQC (magnified) NMR spectrum of **13** in C_6D_6 at 295 K.

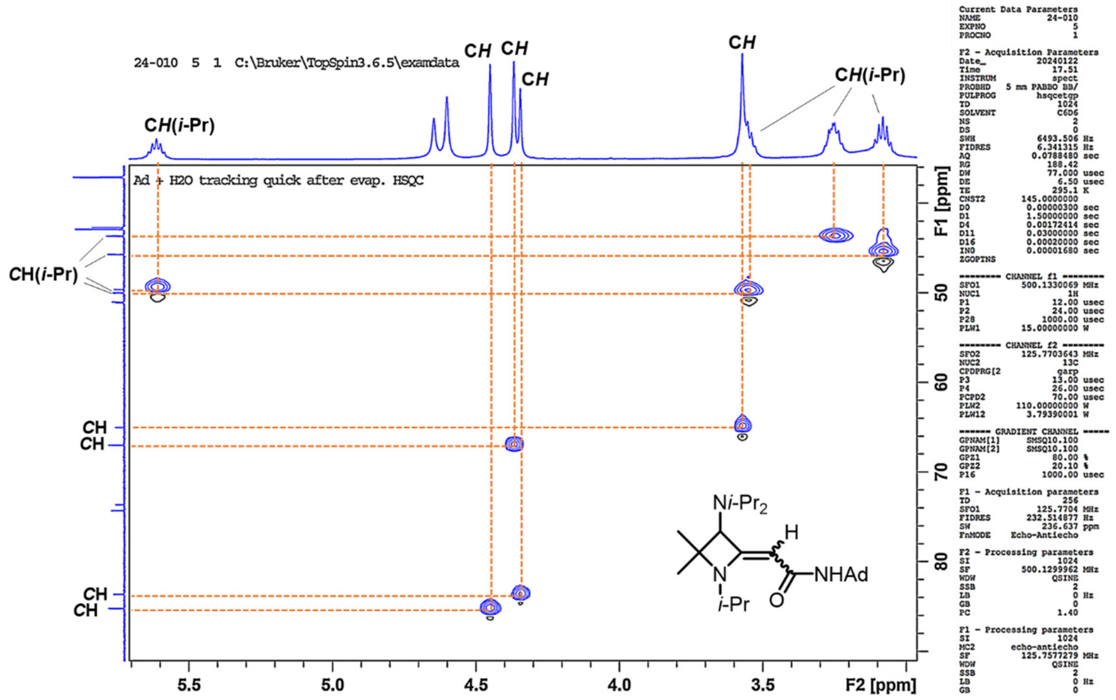

Figure S131. ^{1}H-^{13}C HSQC (magnified) NMR spectrum of **13** in C_6D_6 at 295 K.

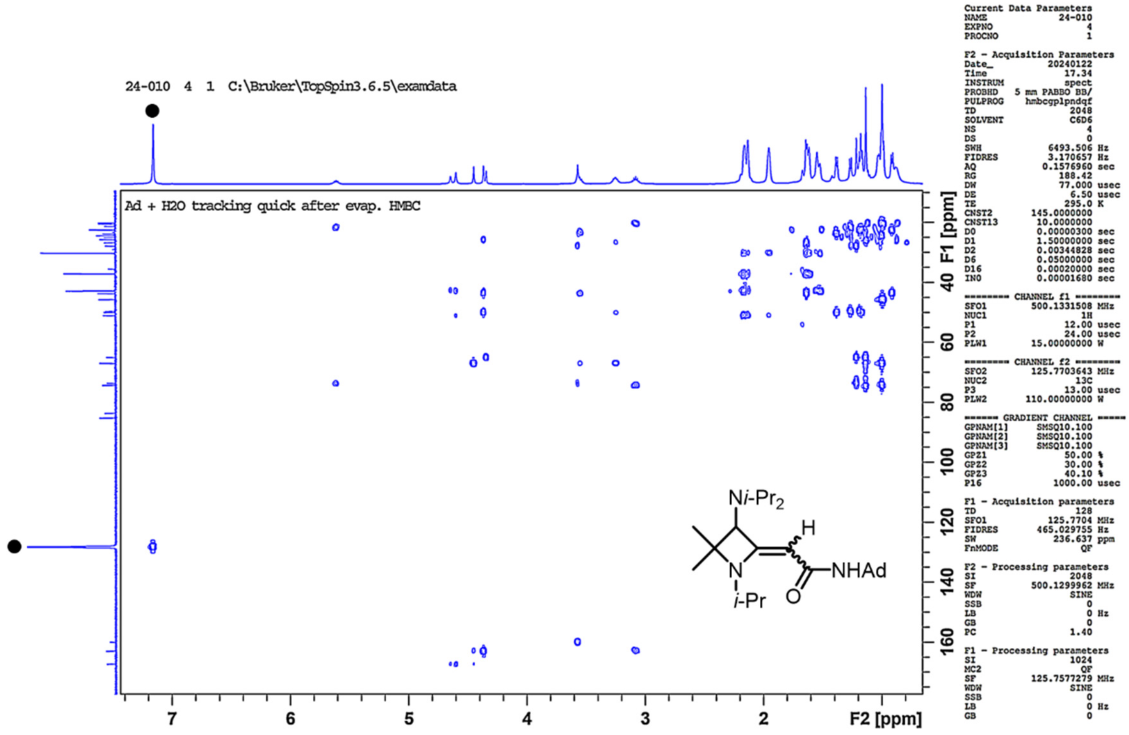

Figure S132. ^{1}H-^{13}C HMBC NMR spectrum of **13** in C_6D_6 at 295 K (● = C_6D_5H & C_6D_6).

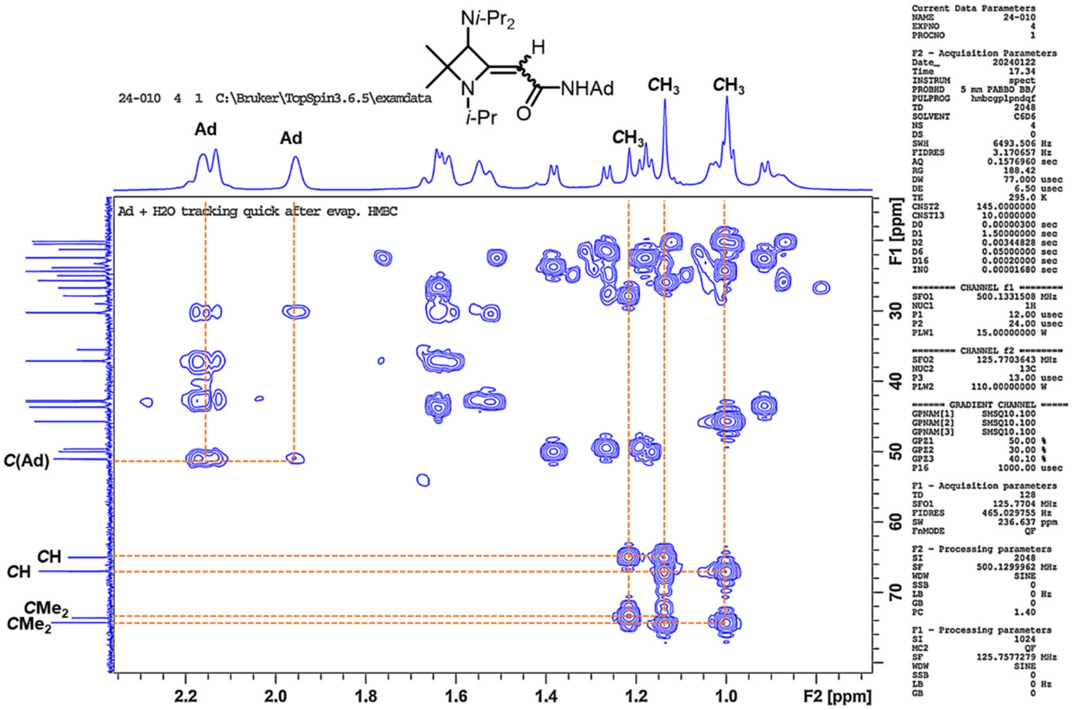

Figure S133. ^{1}H-^{13}C HMBC (magnified) NMR spectrum of **13** in C_6D_6 at 295 K.

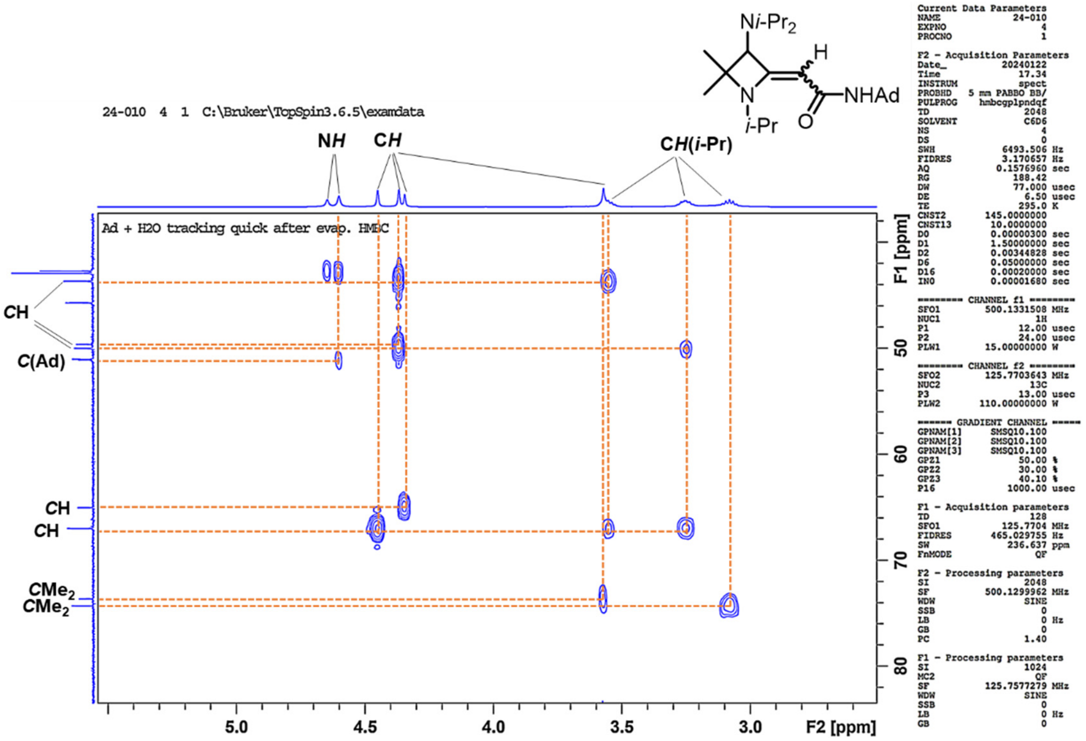

Figure S134. ^{1}H-^{13}C HMBC (magnified) NMR spectrum of **13** in C_6D_6 at 295 K.

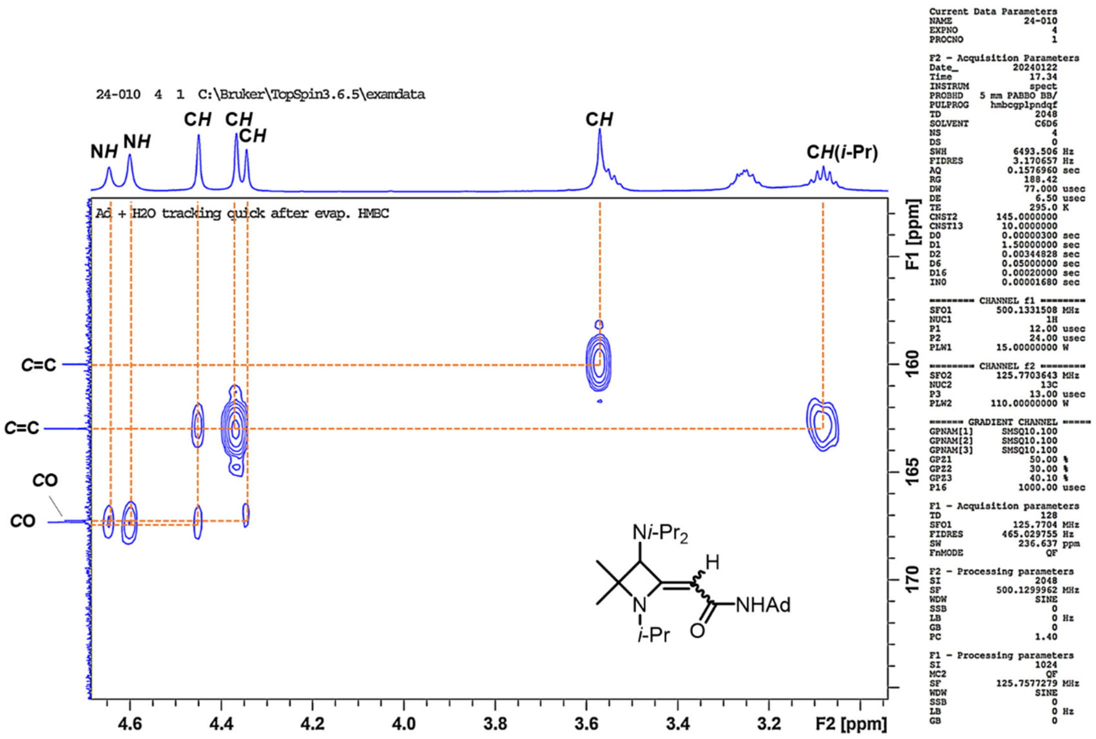

Figure S135. ^{1}H-^{13}C HMBC (magnified) NMR spectrum of **13** in C_6D_6 at 295 K.

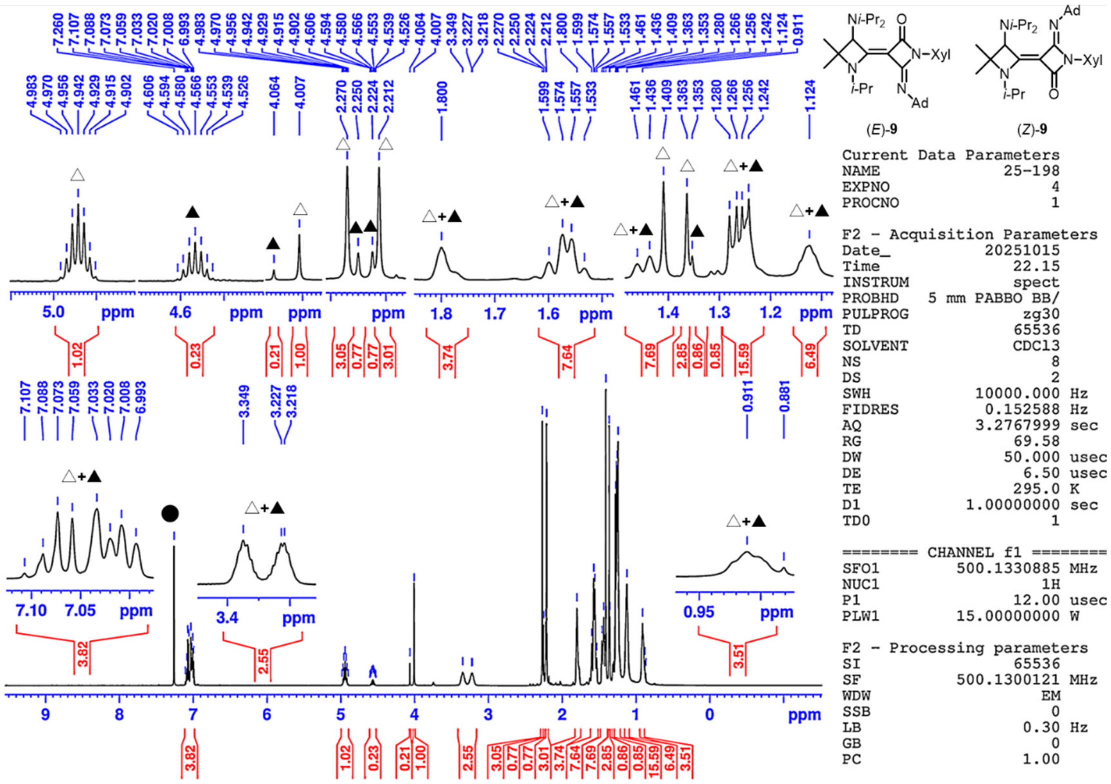

Figure S136. ^{1}H NMR spectrum of **8** in $CDCl_3$ at 295 K (● = $CHCl_3$, △= (*E*)-**8**, ▲= (*Z*)-**8**).

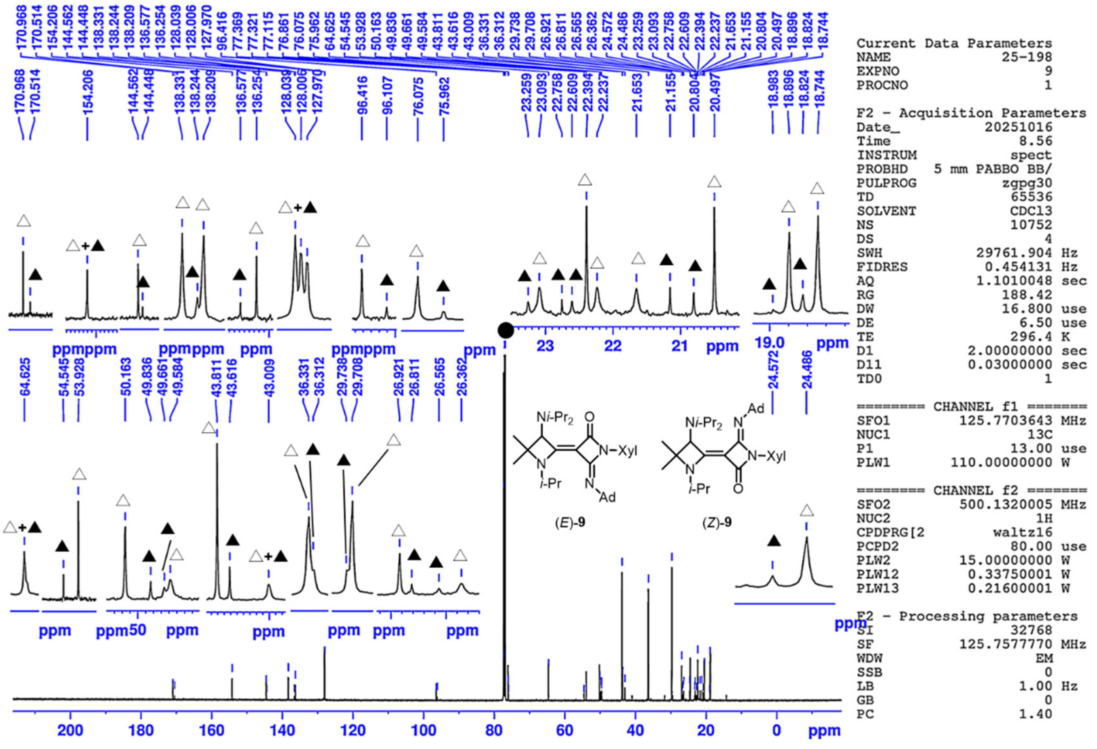

Figure S137. $^{13}C\{^1H\}$ NMR spectrum of **8** in $CDCl_3$ at 296 K (● = $CDCl_3$, △= (*E*)-**8**, ▲= (*Z*)-**8**).

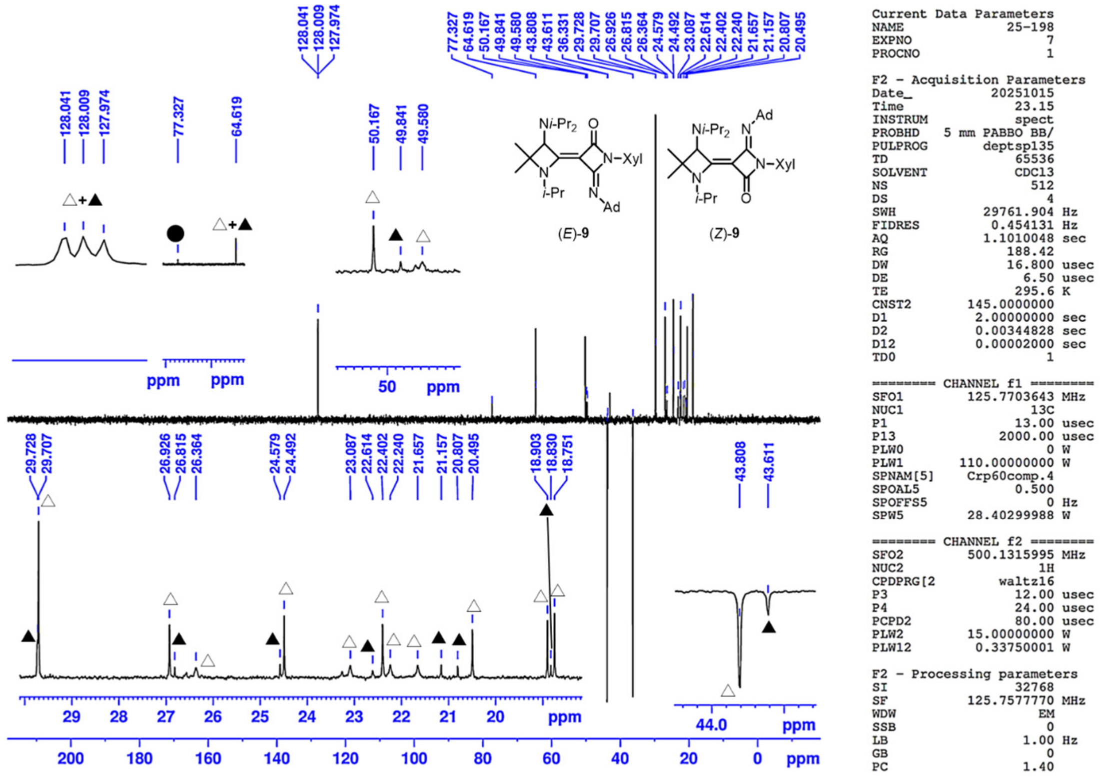

Figure S138. $^{13}C\{^1H\}$ (DEPT135) NMR spectrum of **8** in $CDCl_3$ at 296 K (● = $CDCl_3$, △= (*E*)-**8**, ▲= (*Z*)-**8**).

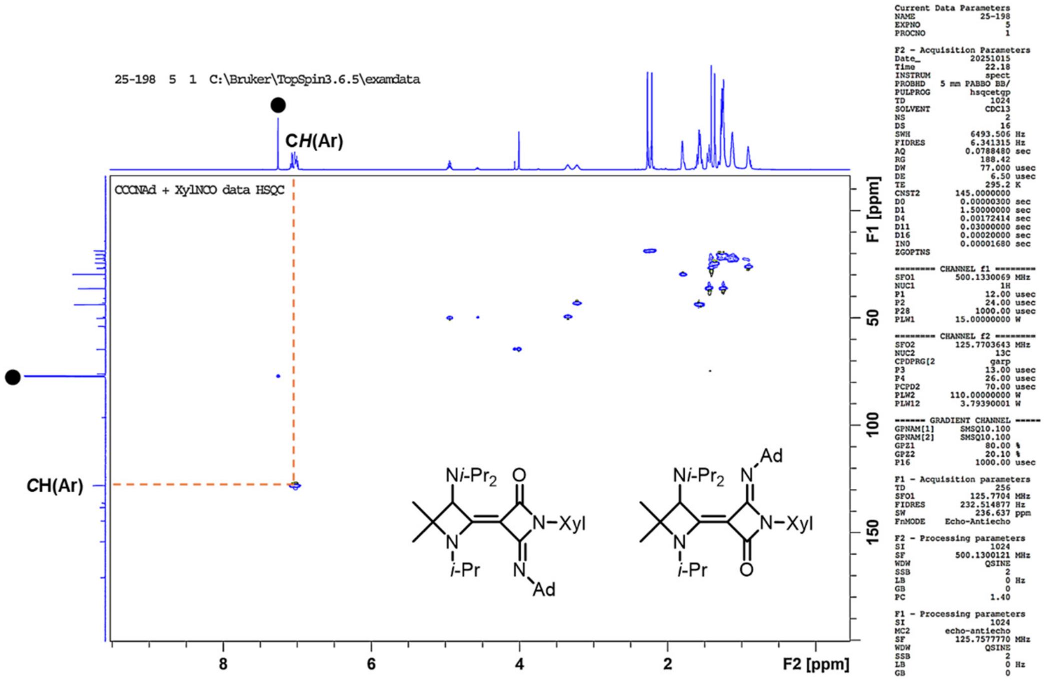

Figure S139. ^{1}H-^{13}C HSQC NMR spectrum of **8** in $CDCl_3$ at 295 K (● = $CHCl_3$ & $CDCl_3$).

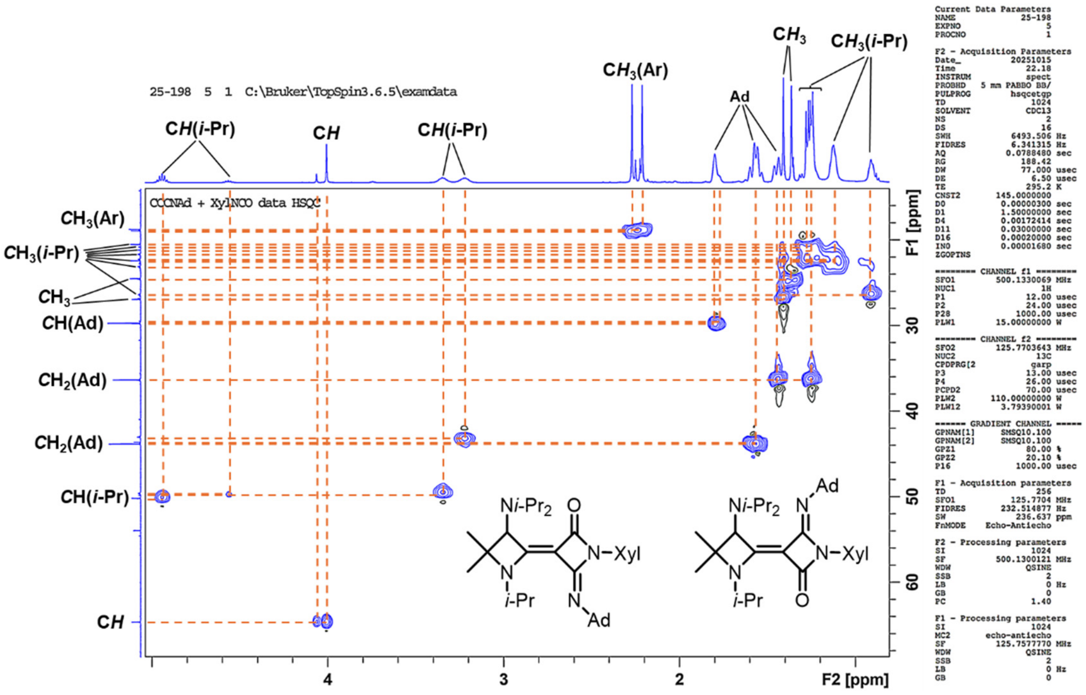

Figure S140. ^{1}H-^{13}C HSQC (magnified) NMR spectrum of **8** in $CDCl_3$ at 295 K.

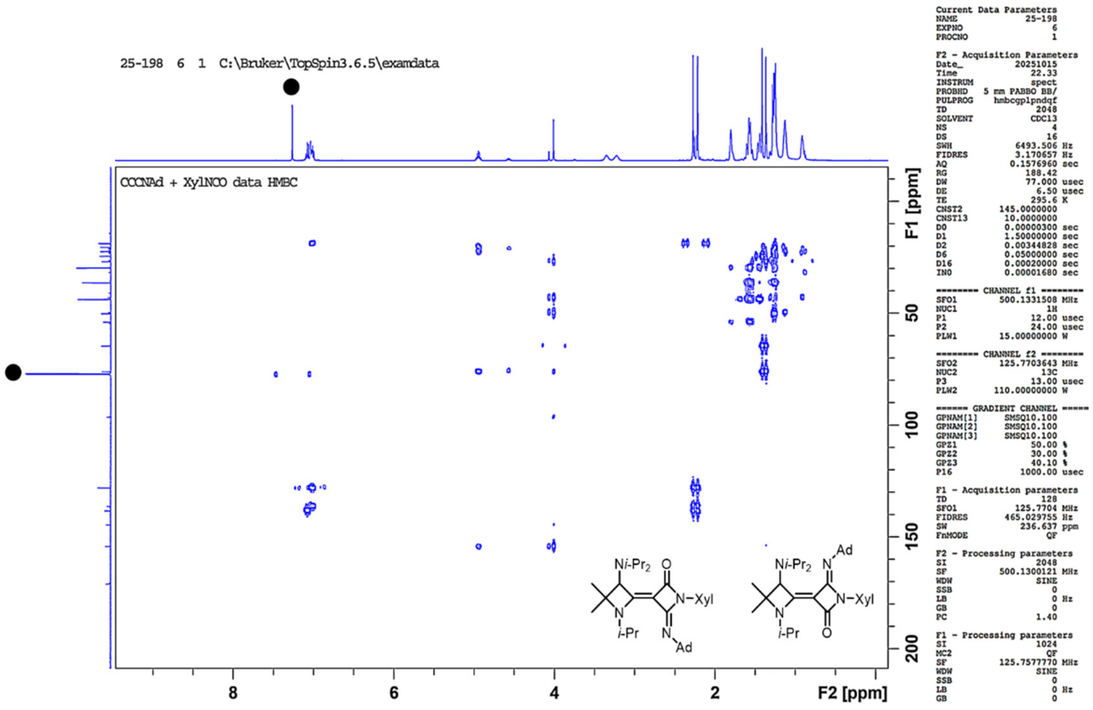

Figure S141. ^{1}H-^{13}C HMBC NMR spectrum of **8** in $CDCl_3$ at 296 K (● = $CHCl_3$ & $CDCl_3$).

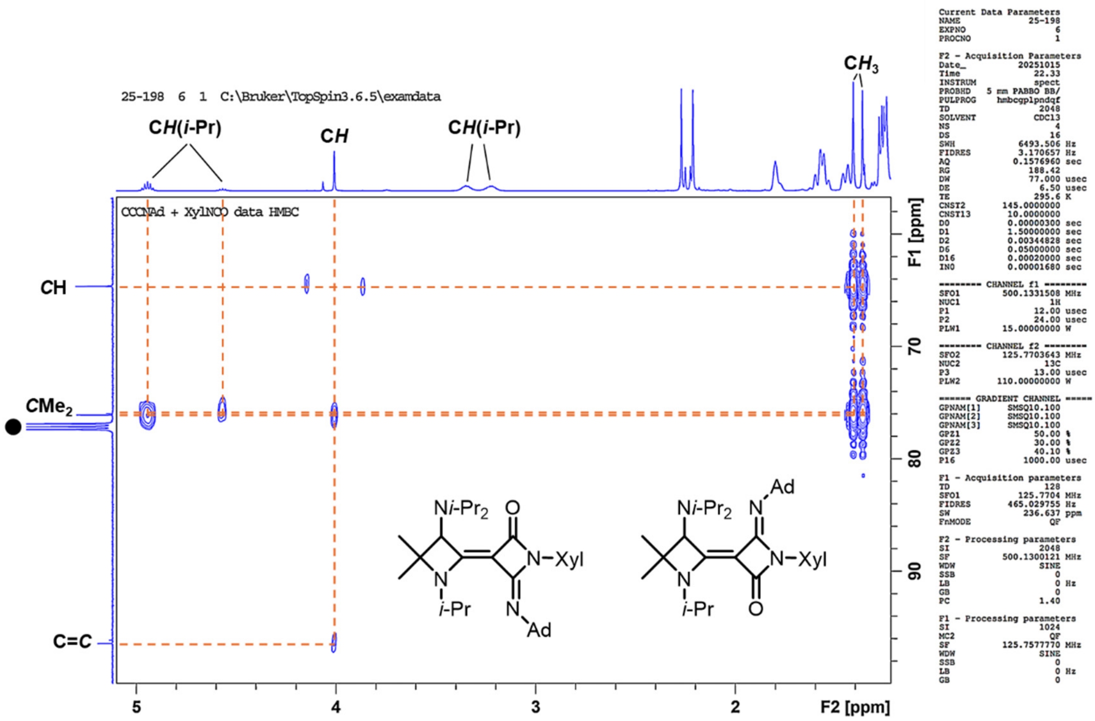

Figure S142. ^{1}H-^{13}C HMBC (magnified) NMR spectrum of **8** in $CDCl_3$ at 296 K (● = $CDCl_3$).

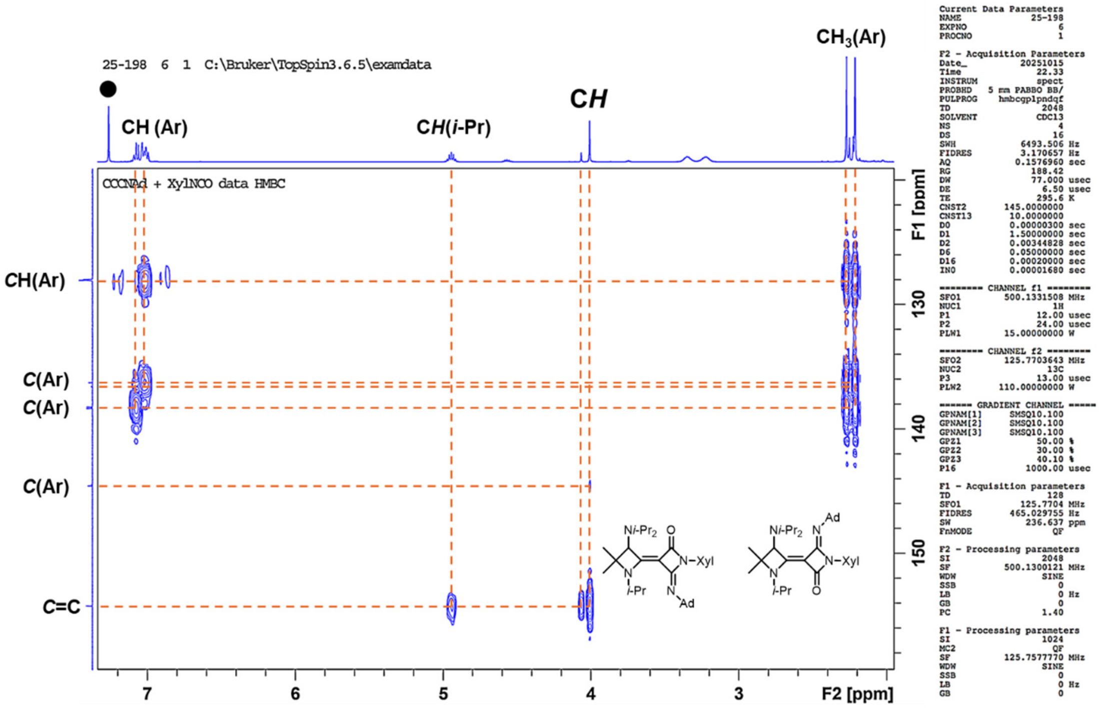

Figure S143. ^{1}H-^{13}C HMBC (magnified) NMR spectrum of **8** in $CDCl_3$ at 296 K (● = $CHCl_3$).

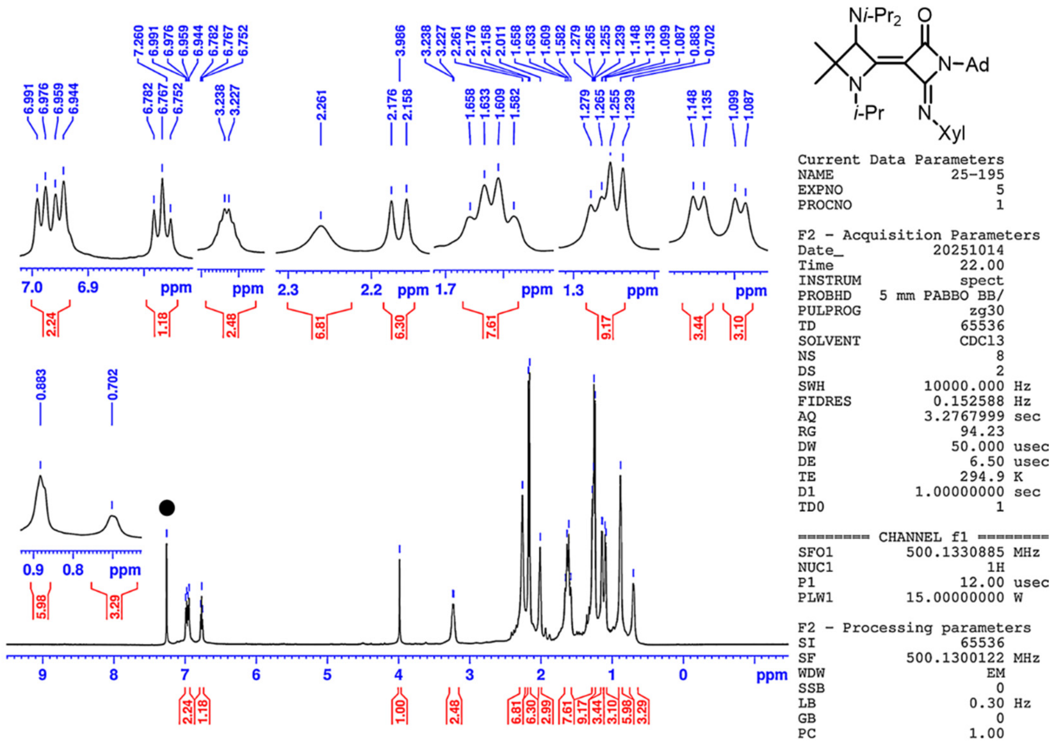

Figure S144. ^{1}H NMR spectrum of **9** in $CDCl_3$ at 295 K (● = $CHCl_3$).

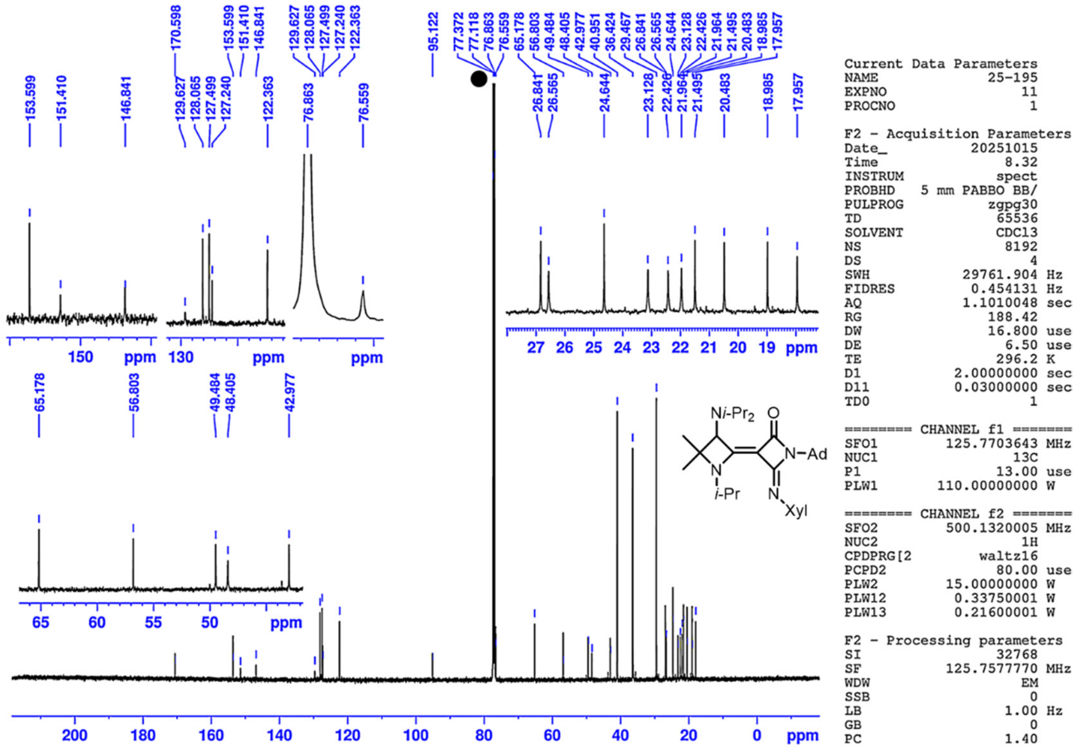

Figure S145. $^{13}C\{^1H\}$ NMR spectrum of **9** in $CDCl_3$ at 296 K (● = $CDCl_3$).

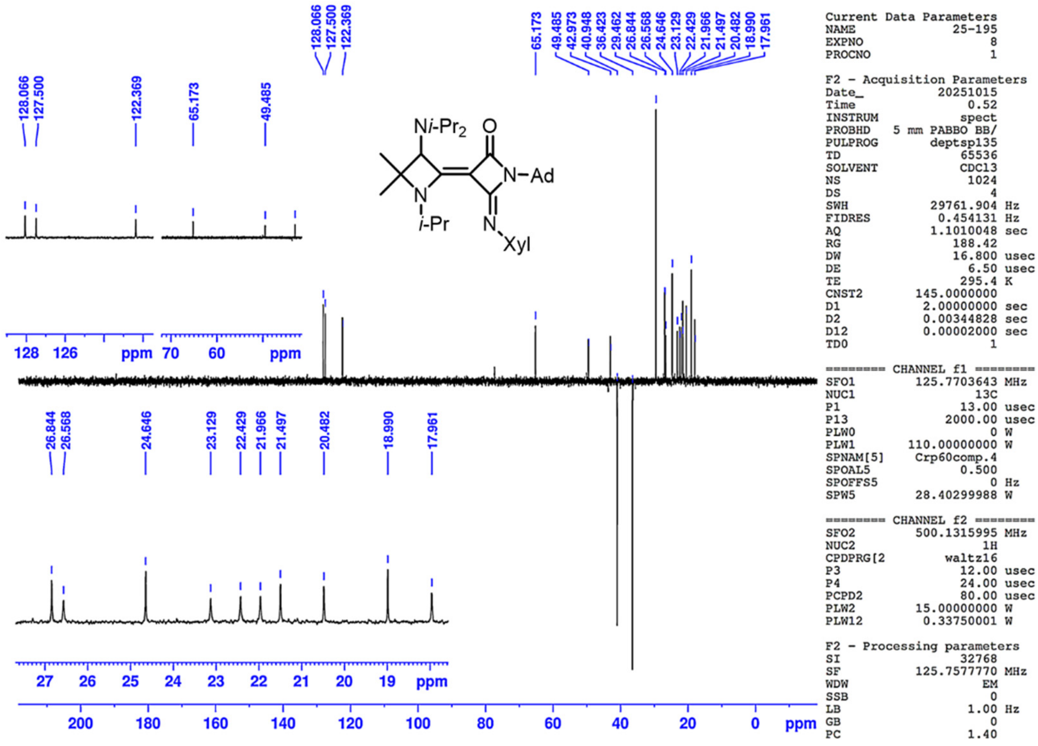

Figure S146. $^{13}C\{^1H\}$ (DEPT135) NMR spectrum of **9** in $CDCl_3$ at 295 K.

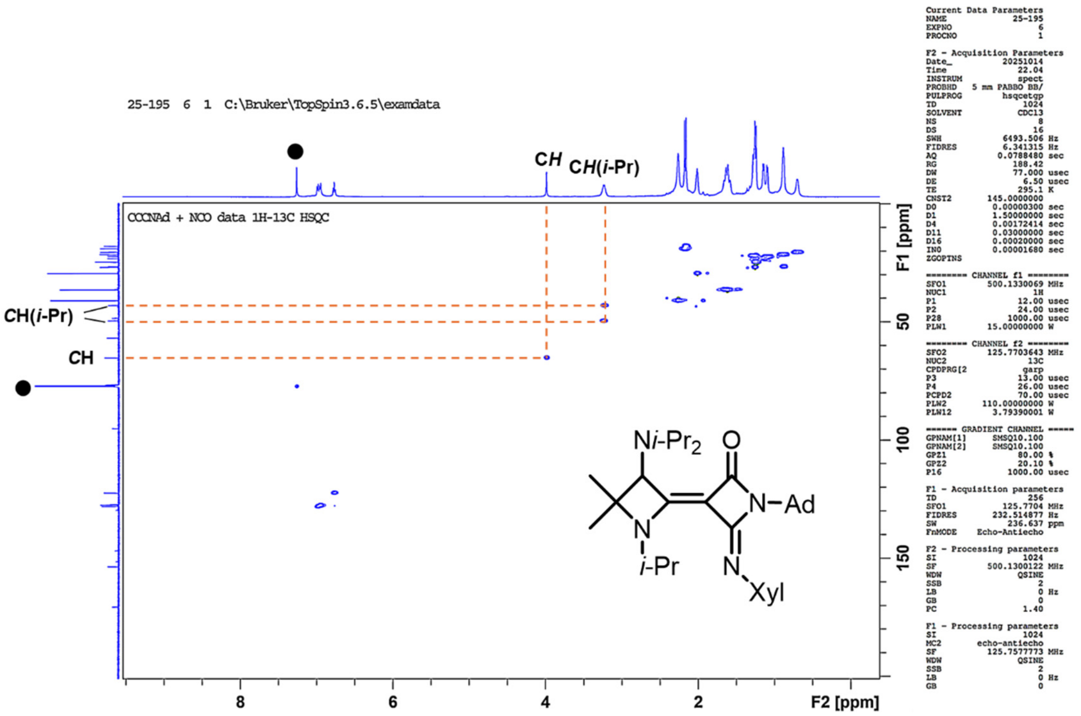

Figure S147. ^{1}H-^{13}C HSQC NMR spectrum of **9** in $CDCl_3$ at 295 K (● = $CHCl_3$ & $CDCl_3$).

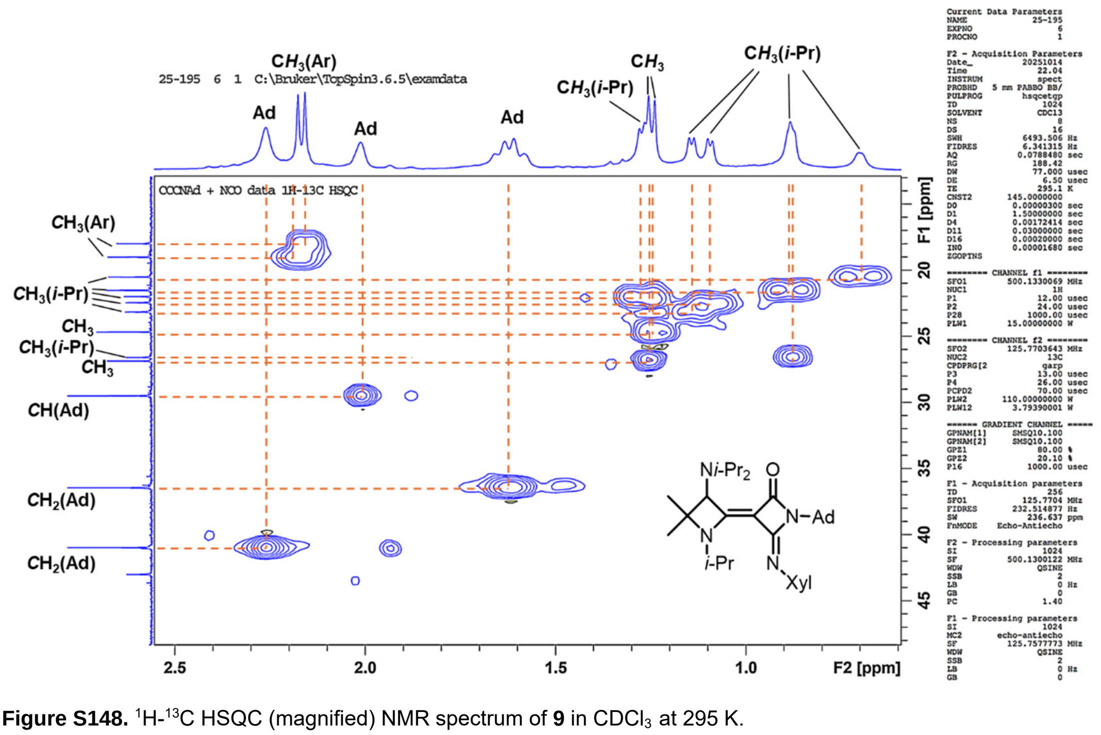

Figure S148. ^{1}H-^{13}C HSQC (magnified) NMR spectrum of **9** in $CDCl_3$ at 295 K.

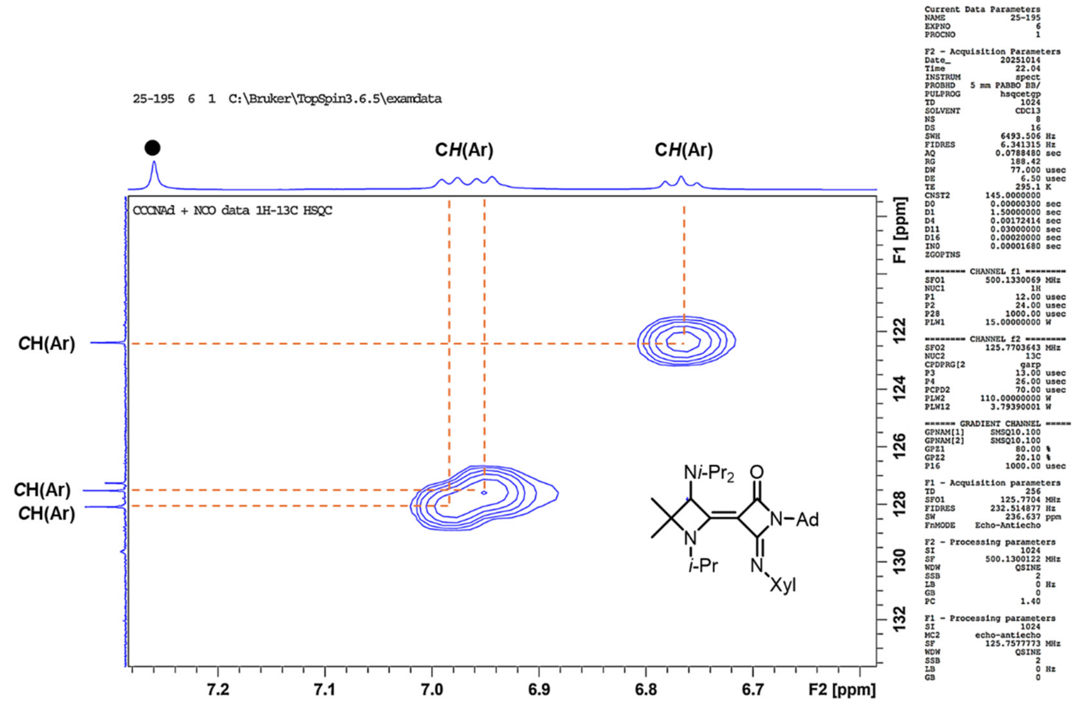

Figure S149. ^{1}H-^{13}C HSQC (magnified) NMR spectrum of **9** in $CDCl_3$ at 295 K (● = $CHCl_3$).

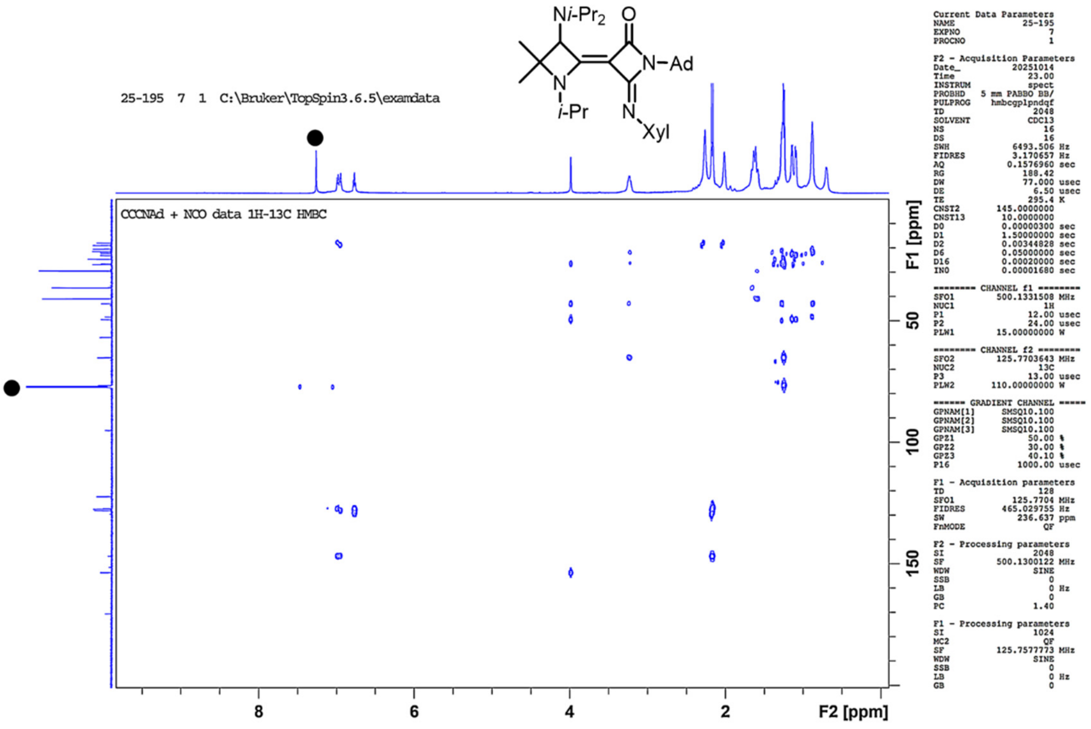

Figure S150. ^{1}H-^{13}C HMBC NMR spectrum of **9** in $CDCl_3$ at 295 K (● = $CHCl_3$ & $CDCl_3$).

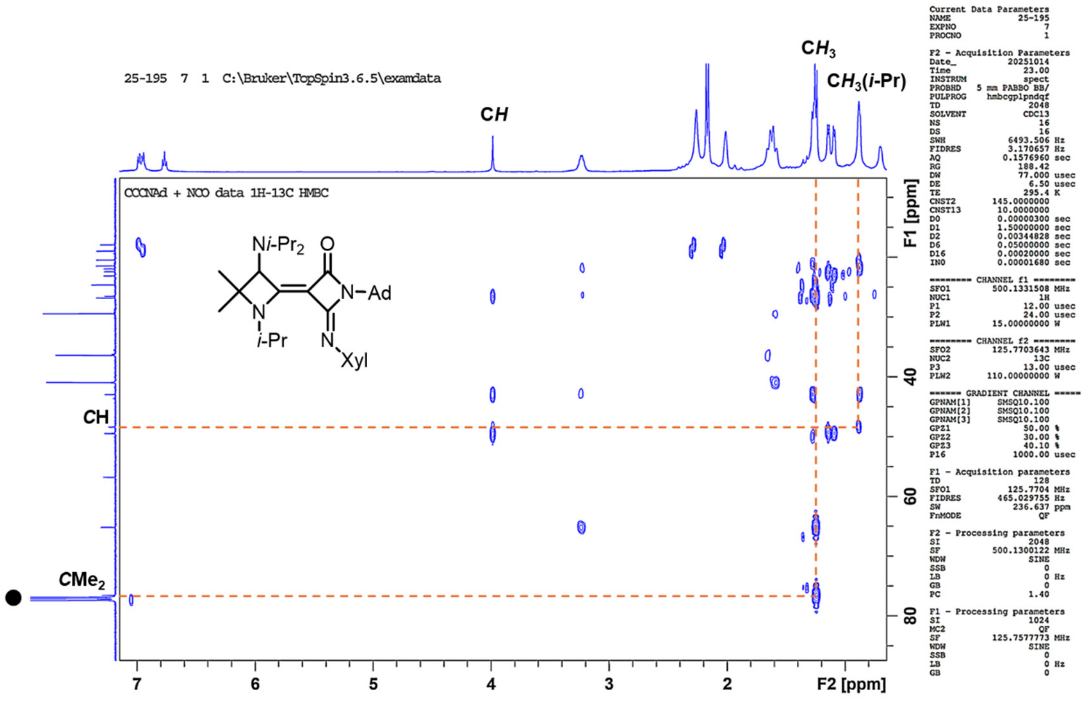

Figure S151. ^{1}H-^{13}C HMBC (magnified) NMR spectrum of **9** in $CDCl_3$ at 295 K (● = $CDCl_3$).

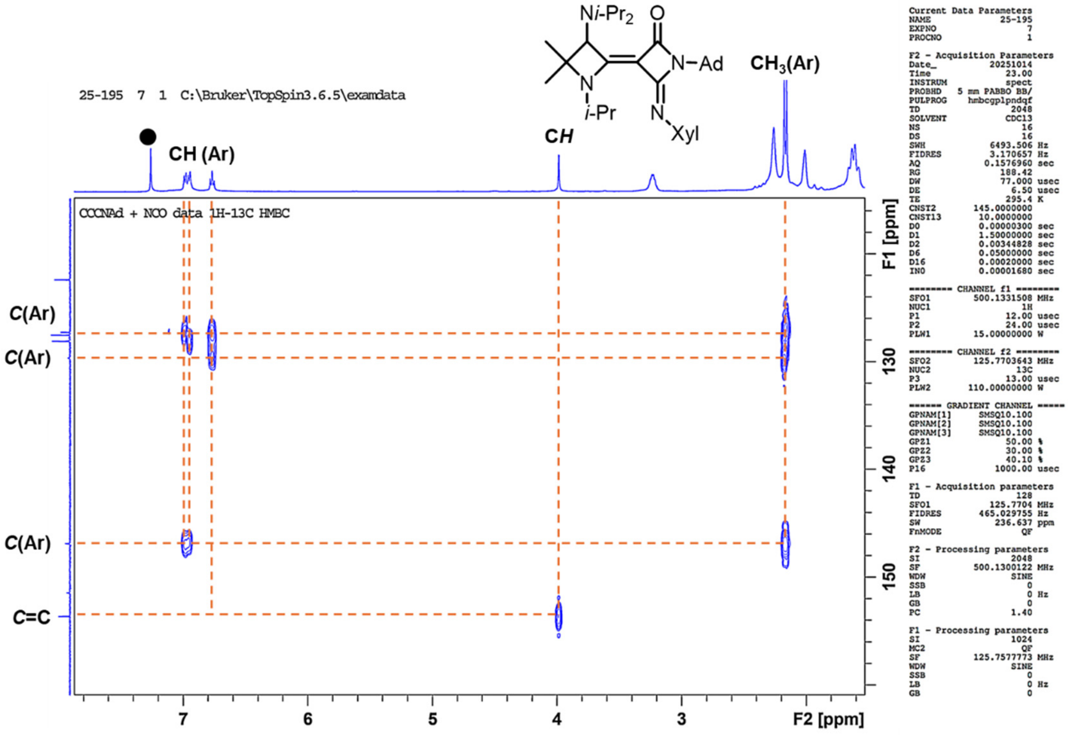

Figure S152. ^{1}H-^{13}C HMBC (magnified) NMR spectrum of **9** in $CDCl_3$ at 295 K (● = $CHCl_3$).

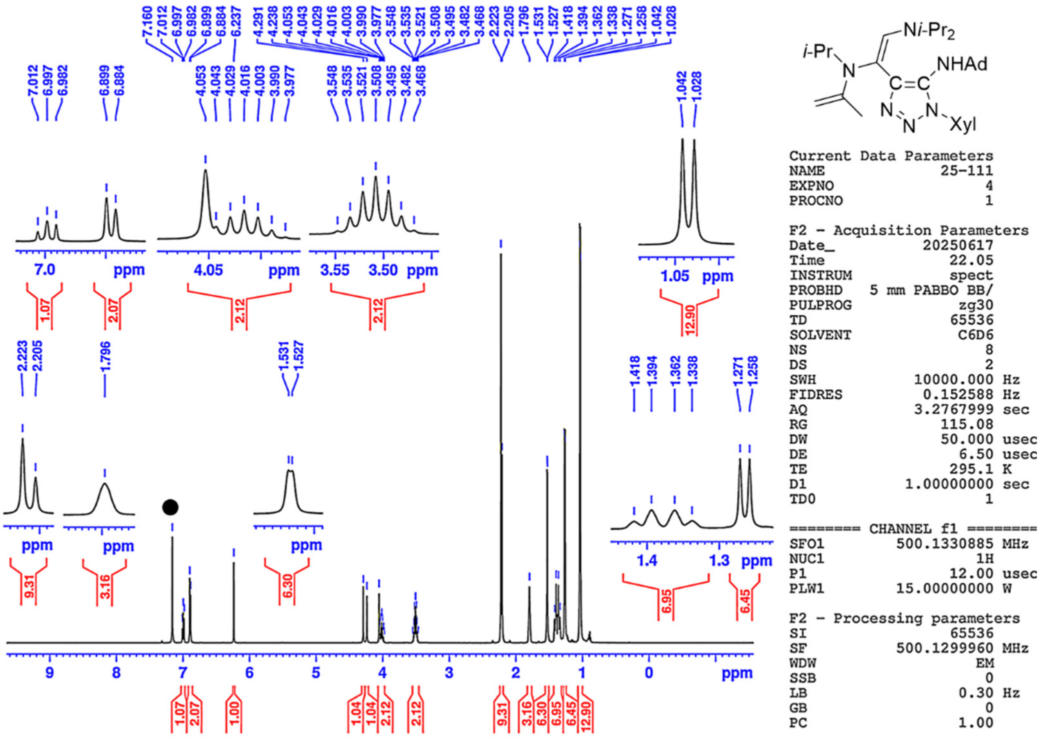

Figure S153. ^{1}H NMR spectrum of the **10** in C_6D_6 at 295 K (● = C_6D_5H).

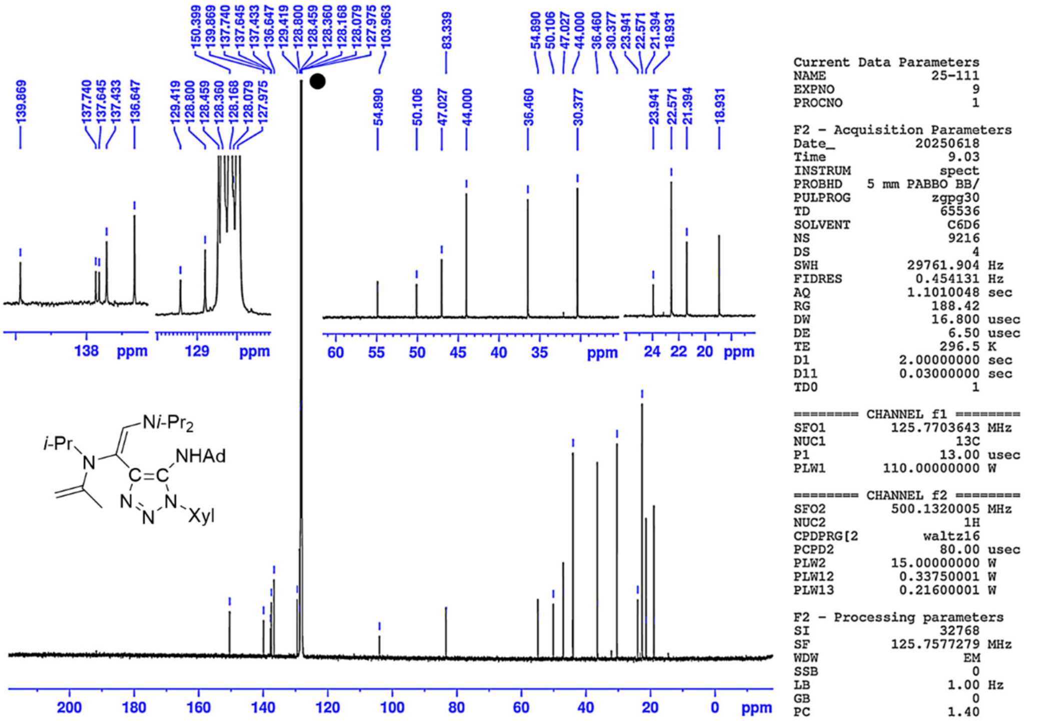

Figure S154. $^{13}C\{^{1}H\}$ NMR spectrum of **10** in C_6D_6 at 297 K (● = C_6D_6).

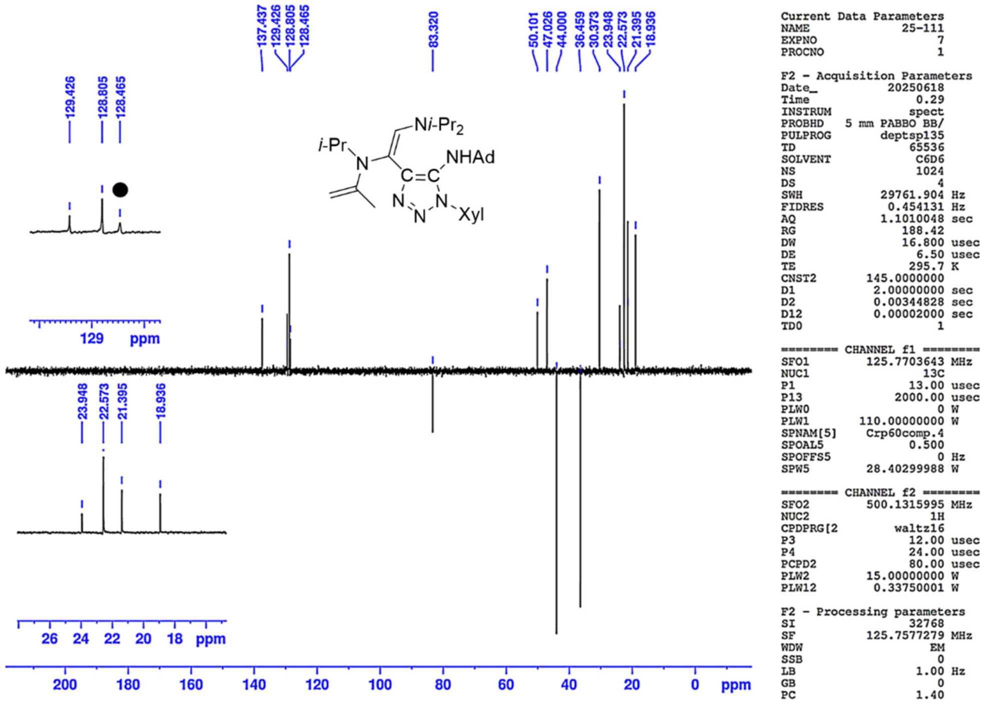

Figure S155. $^{13}C\{^1H\}$ (DEPT135) NMR spectrum of **10** in C_6D_6 at 296 K (● = C_6D_5H).

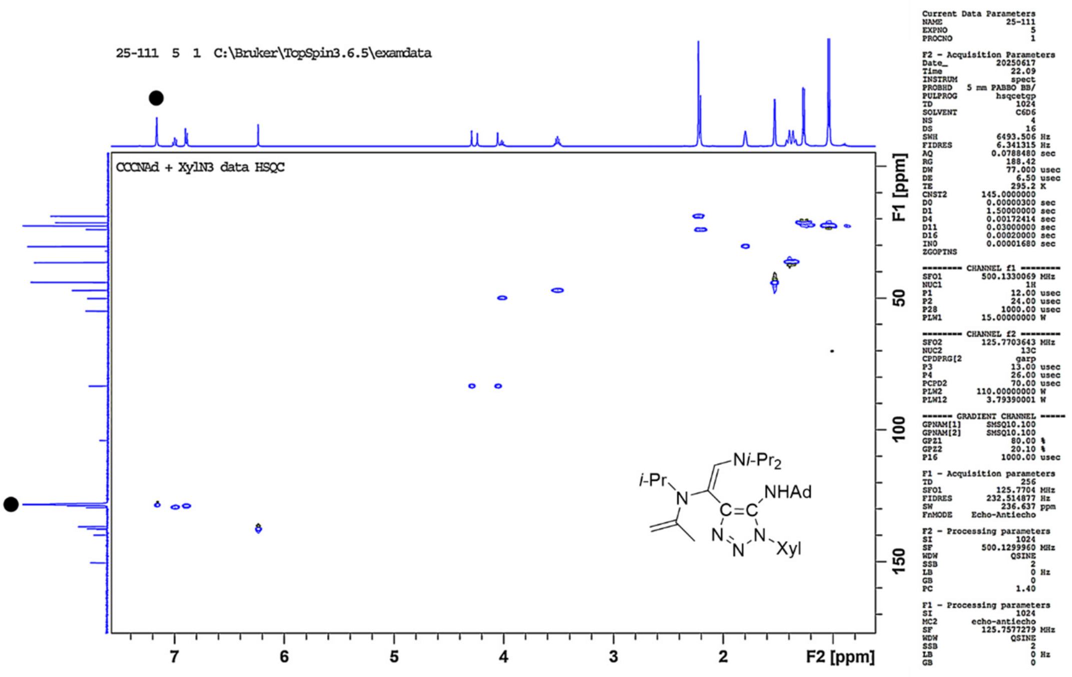

Figure S156. ^1H-^{13}C HSQC NMR spectrum of **10** in C_6D_6 at 295 K (● = C_6D_5H & C_6D_6).

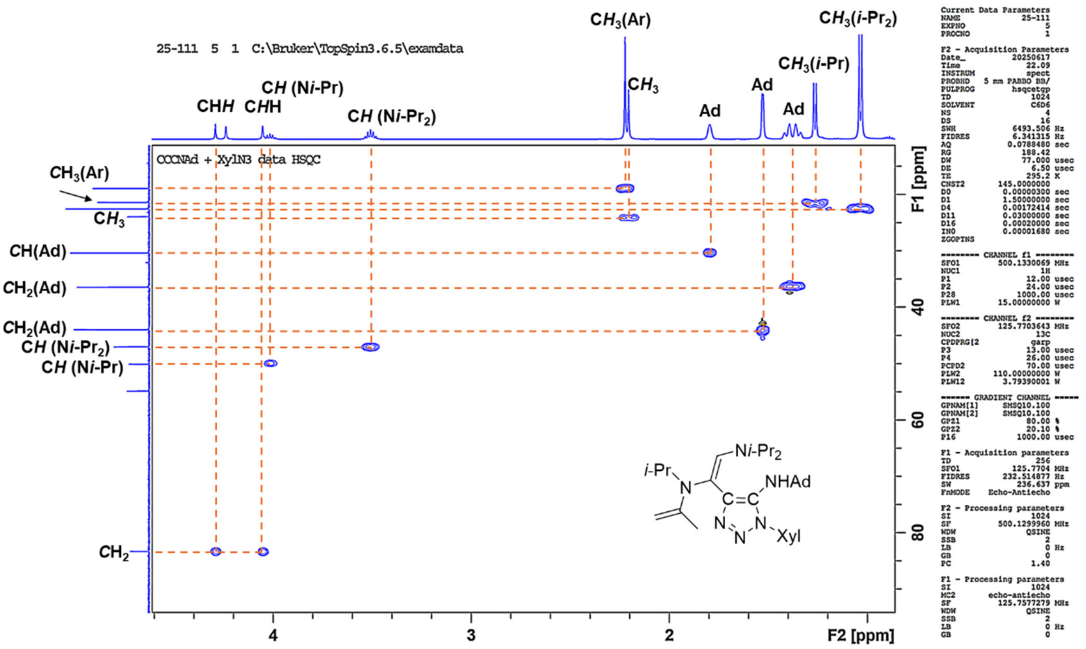

Figure S157. ^{1}H-^{13}C HSQC (magnified) NMR spectrum of **10** in C_6D_6 at 295 K.

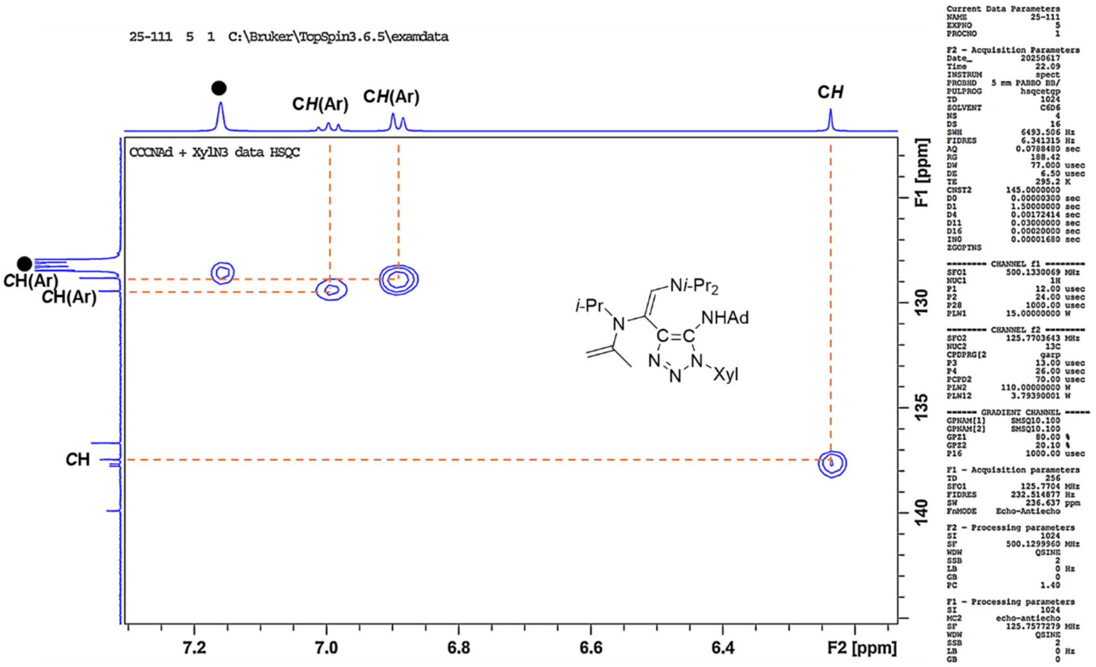

Figure S158. ^{1}H-^{13}C HSQC (magnified) NMR spectrum of **10** in C_6D_6 at 295 K (● = C_6D_5H & C_6D_6).

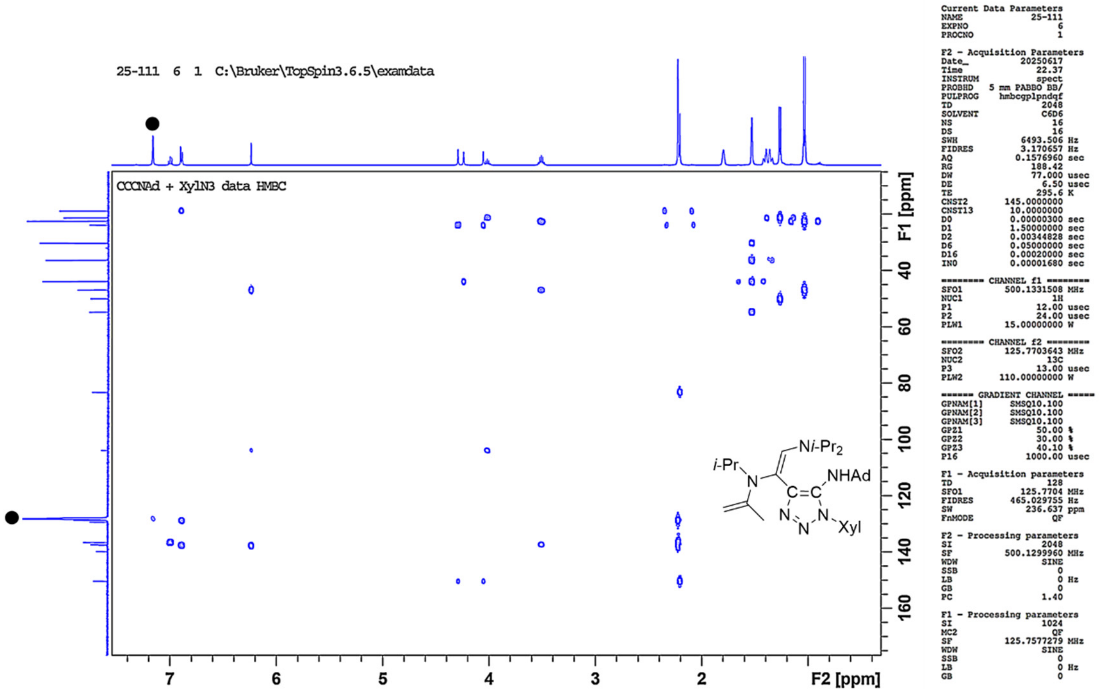

Figure S159. ^{1}H-^{13}C HMBC NMR spectrum of **10** in C_6D_6 at 296 K (● = C_6D_5H & C_6D_6).

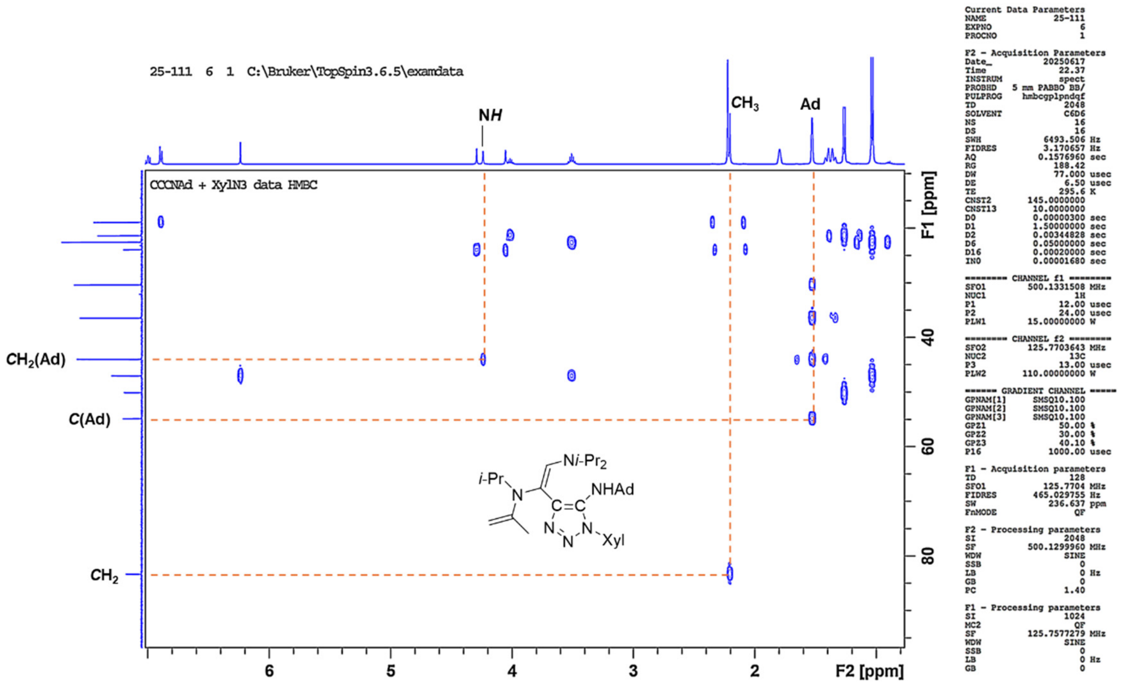

Figure S160. ^{1}H-^{13}C HMBC (magnified) NMR spectrum of **10** in C_6D_6 at 296 K.

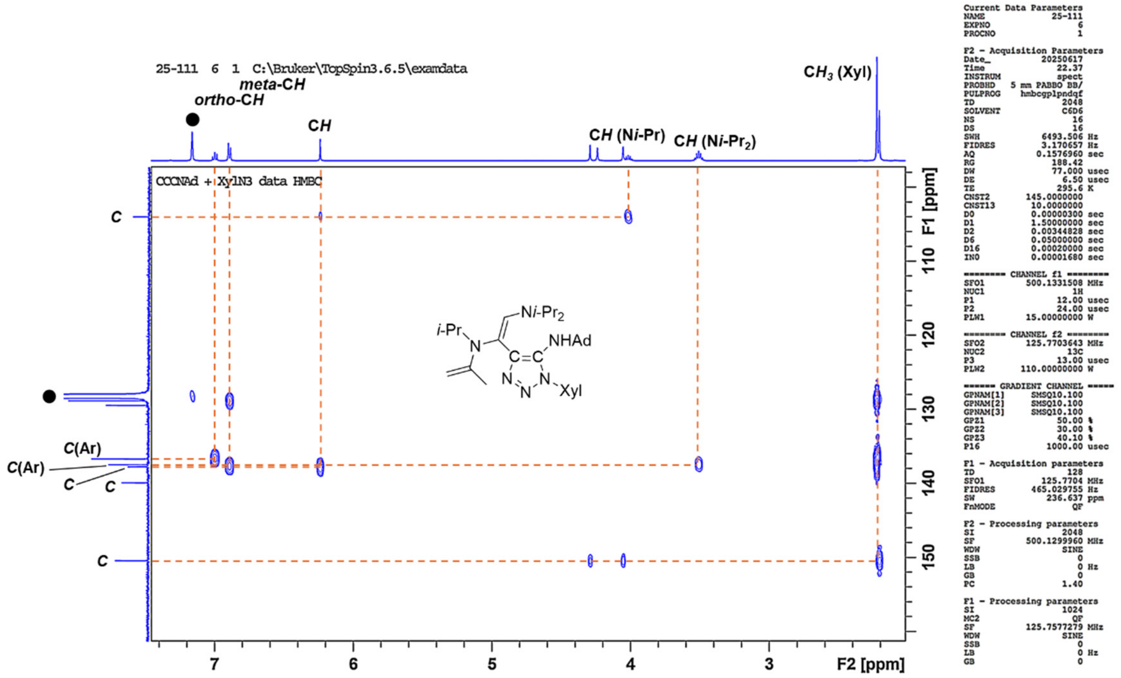

Figure S161. ^{1}H-^{13}C HMBC (magnified) NMR spectrum of **10** in C_6D_6 at 296 K (● = C_6D_5H & C_6D_6).

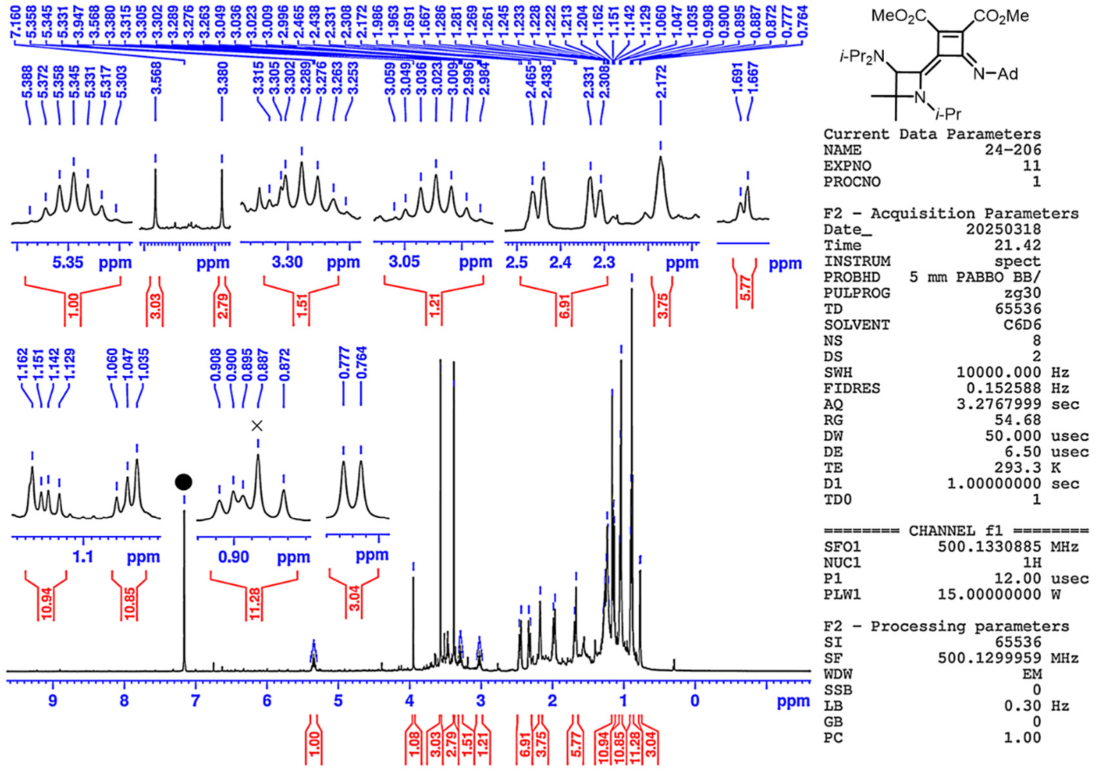

Figure S162. ^{1}H NMR spectrum of crude **11** in C_6D_6 at 293 K (● = C_6D_5H, × = residual hexane).

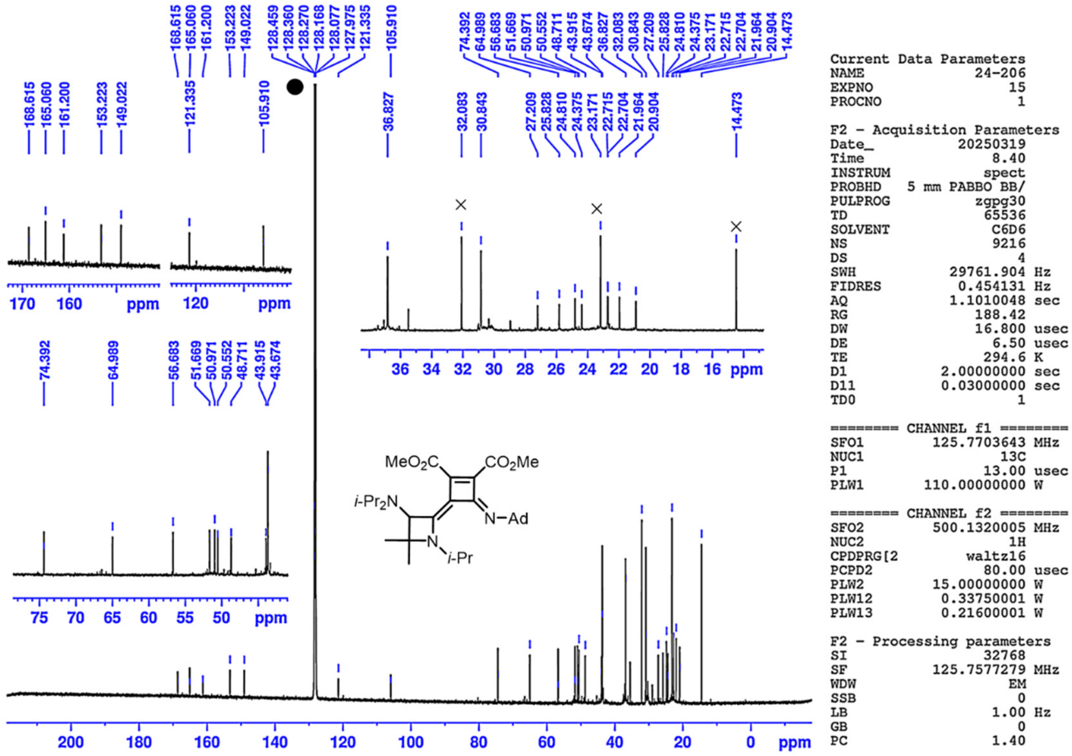

Figure S163. $^{13}C\{^{1}H\}$ NMR spectrum of crude **11** in C_6D_6 at 295 K (● = C_6D_6, × = residual hexane).

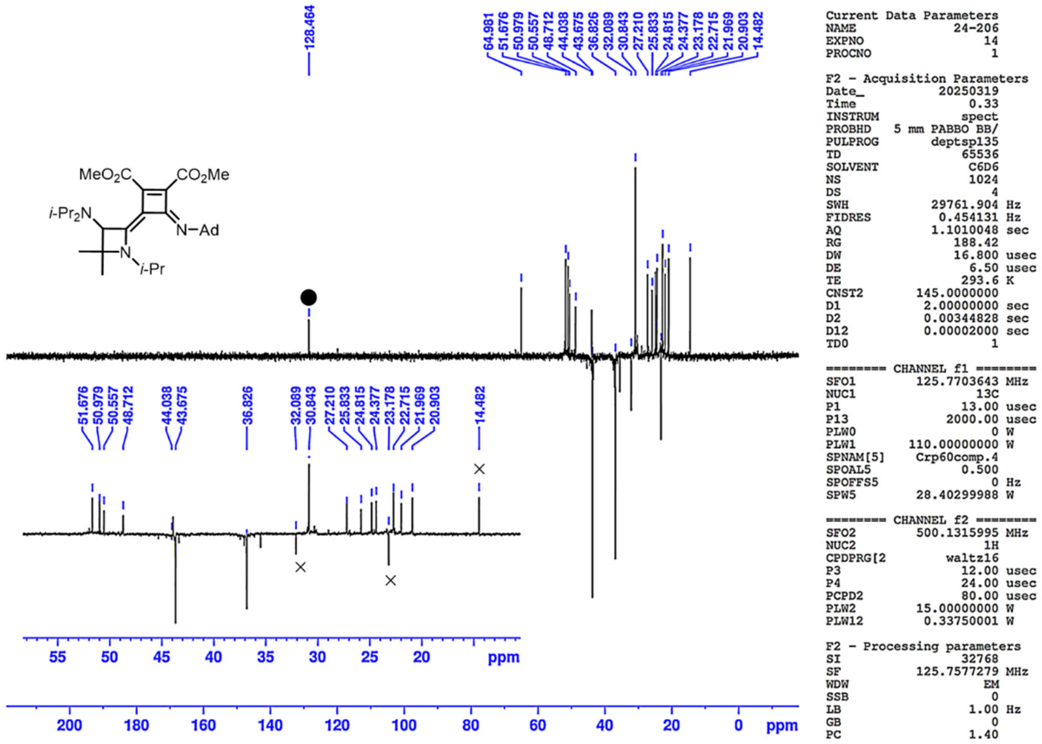

Figure S164. $^{13}C\{^{1}H\}$ (DEPT135) NMR spectrum of crude **11** in C_6D_6 at 294 K (● = C_6D_5H, × = residual hexane).

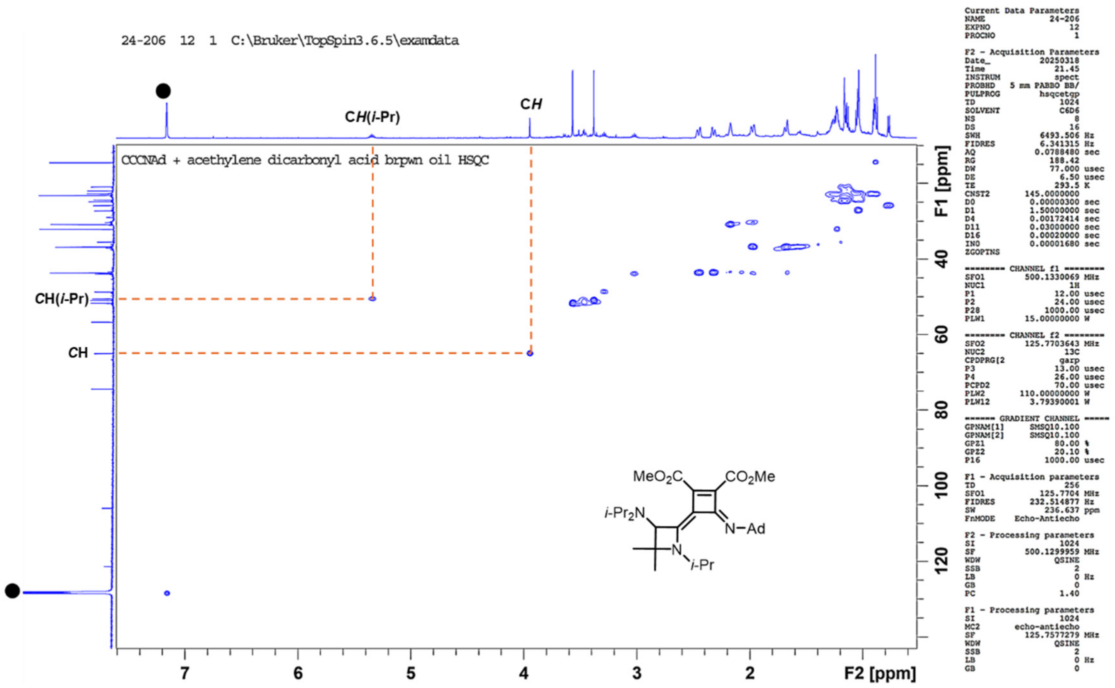

Figure S165. ^1H-^{13}C HSQC NMR spectrum of crude **11** in C_6D_6 at 294 K (● = C_6D_5H & C_6D_6).

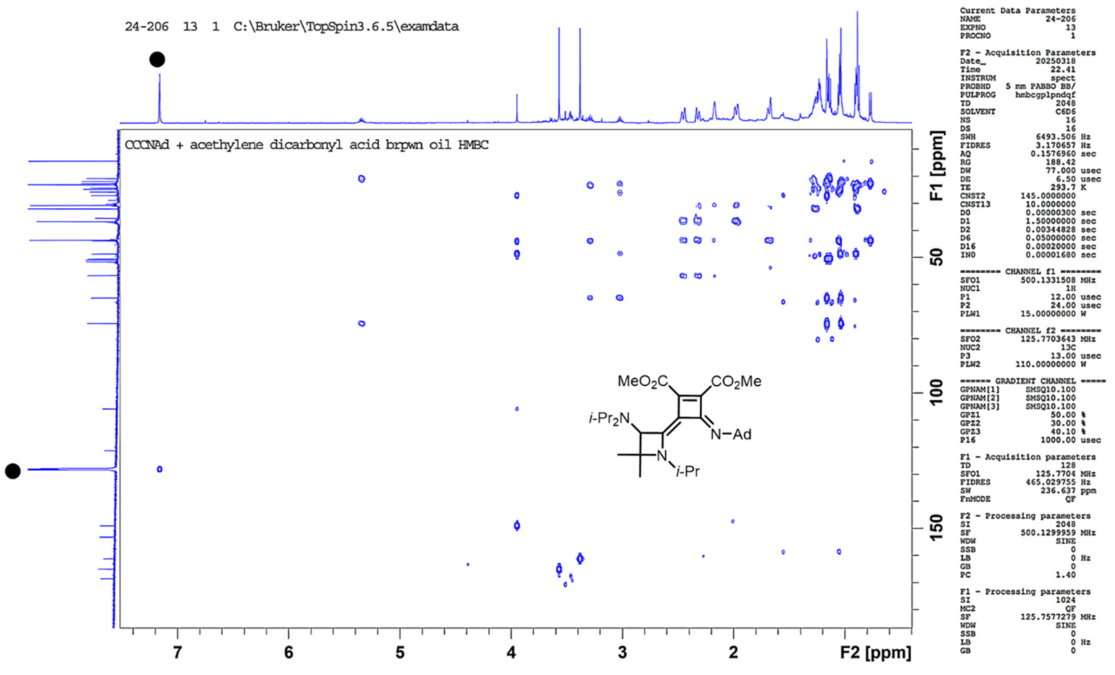

Figure S166. ^1H-^{13}C HMBC NMR spectrum of crude **11** in C_6D_6 at 294 K (● = C_6D_5H & C_6D_6).

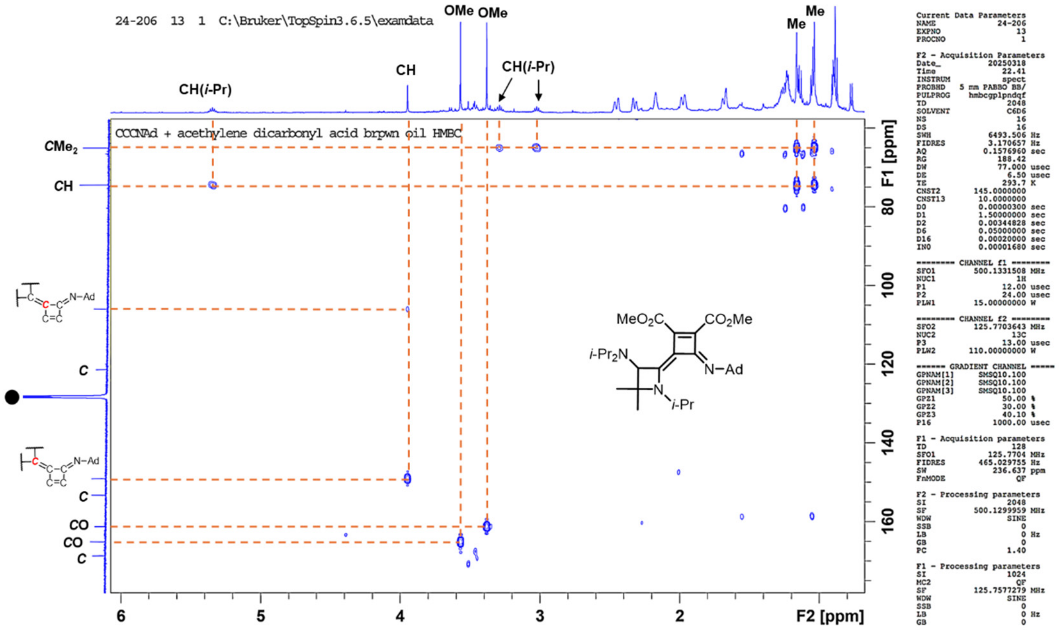

Figure S167. ^1H-^{13}C HMBC (magnified) NMR spectrum of crude **11** in C_6D_6 at 294 K (● = C_6D_6).

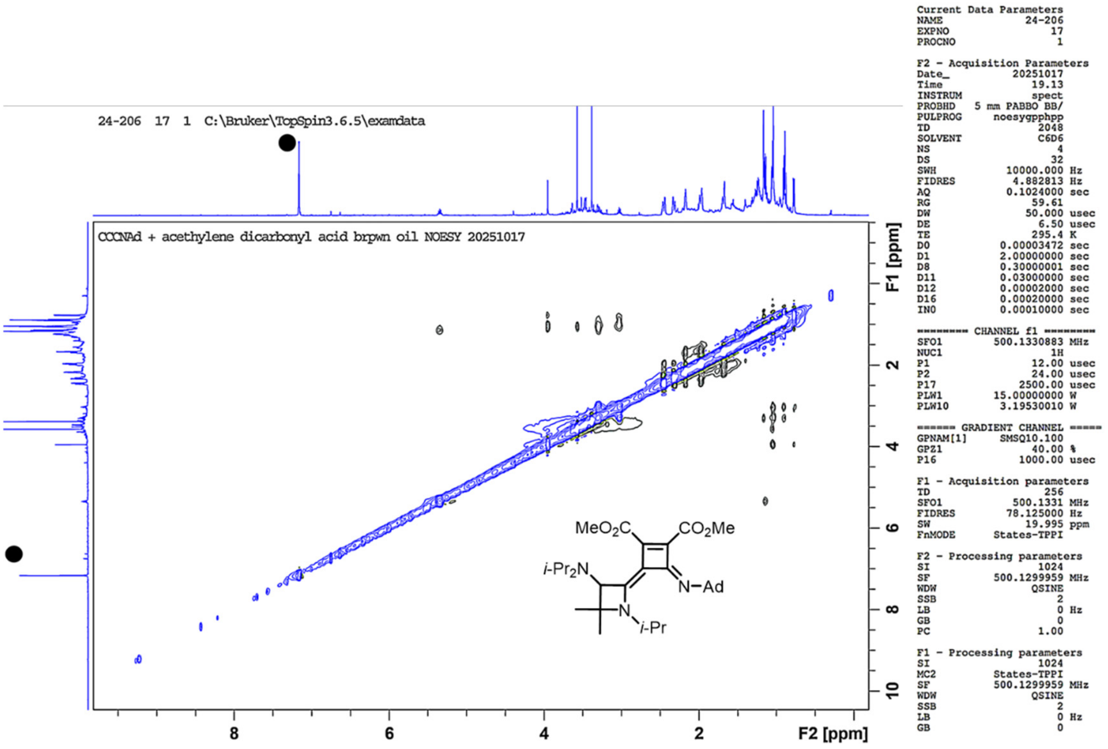

Figure S168. ^1H-^1H NOESY NMR spectrum of crude **11** in C_6D_6 at 295 K (● = C_6D_5H & C_6D_6).

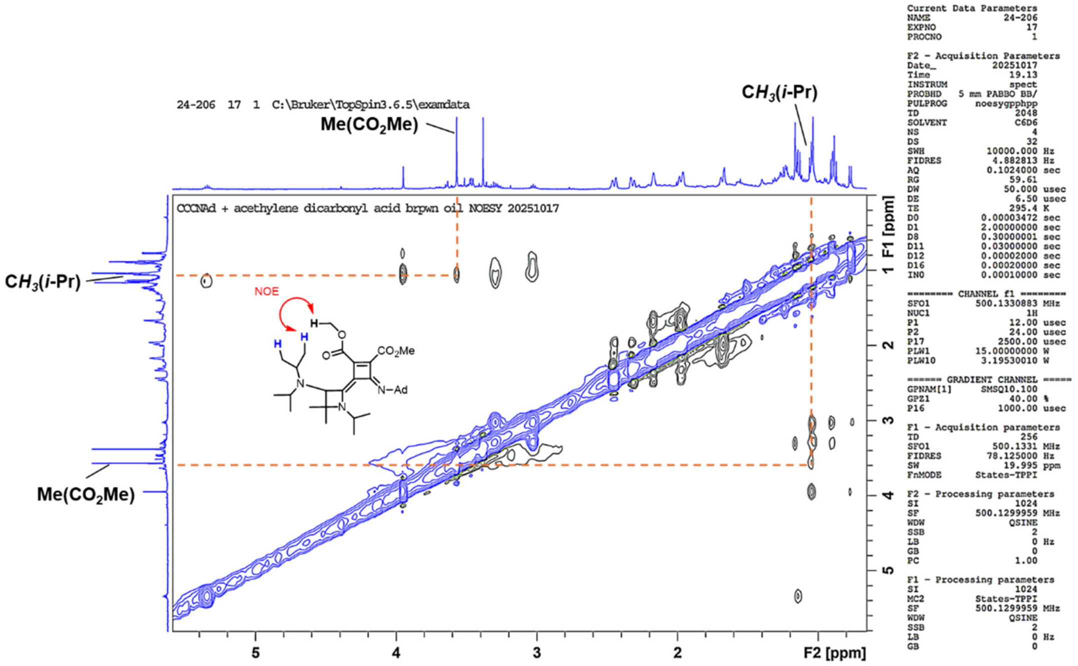

Figure S169. ^{1}H-^{1}H NOESY (magnified) NMR spectrum of crude **11** in C_6D_6 at 295 K.

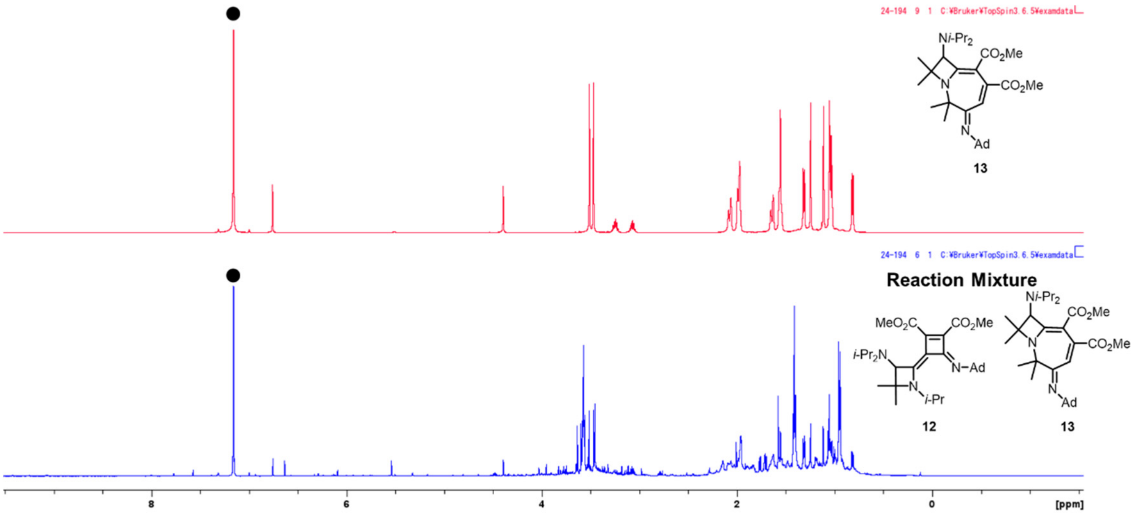

Figure S170. Comparison of ^{1}H NMR spectrum of crude **11** and **12** (● = C_6D_5H).

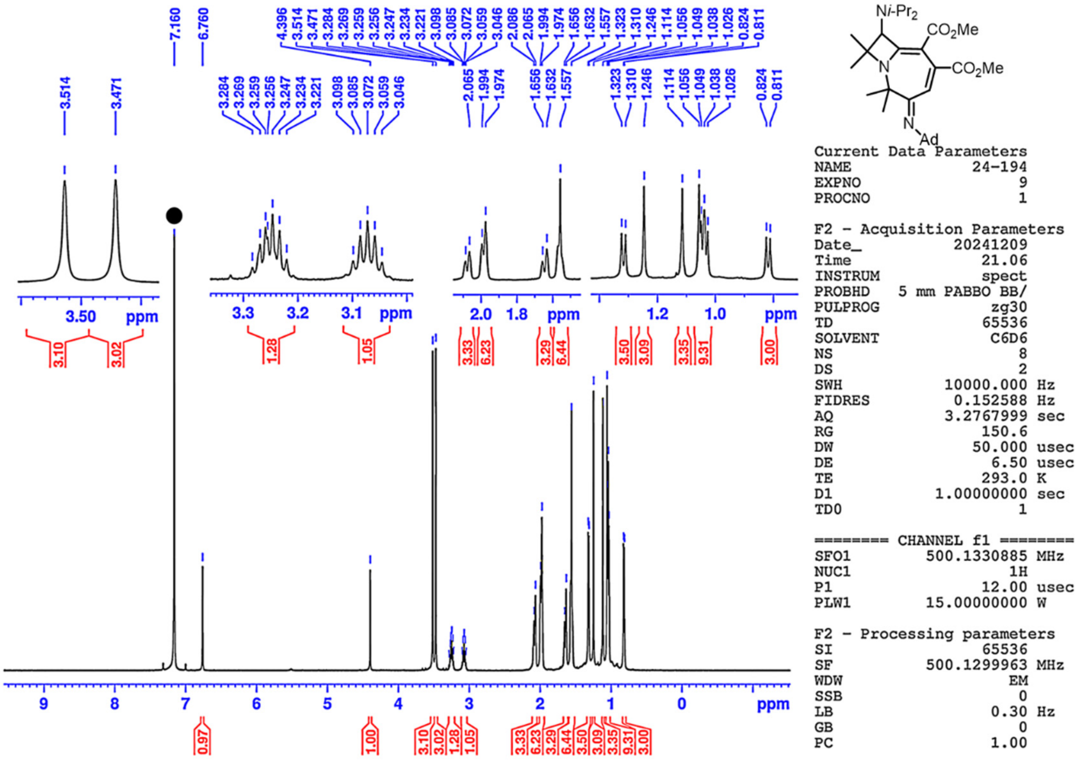

Figure S171. ^{1}H NMR spectrum of **12** in C_6D_6 at 293 K (● = C_6D_5H).

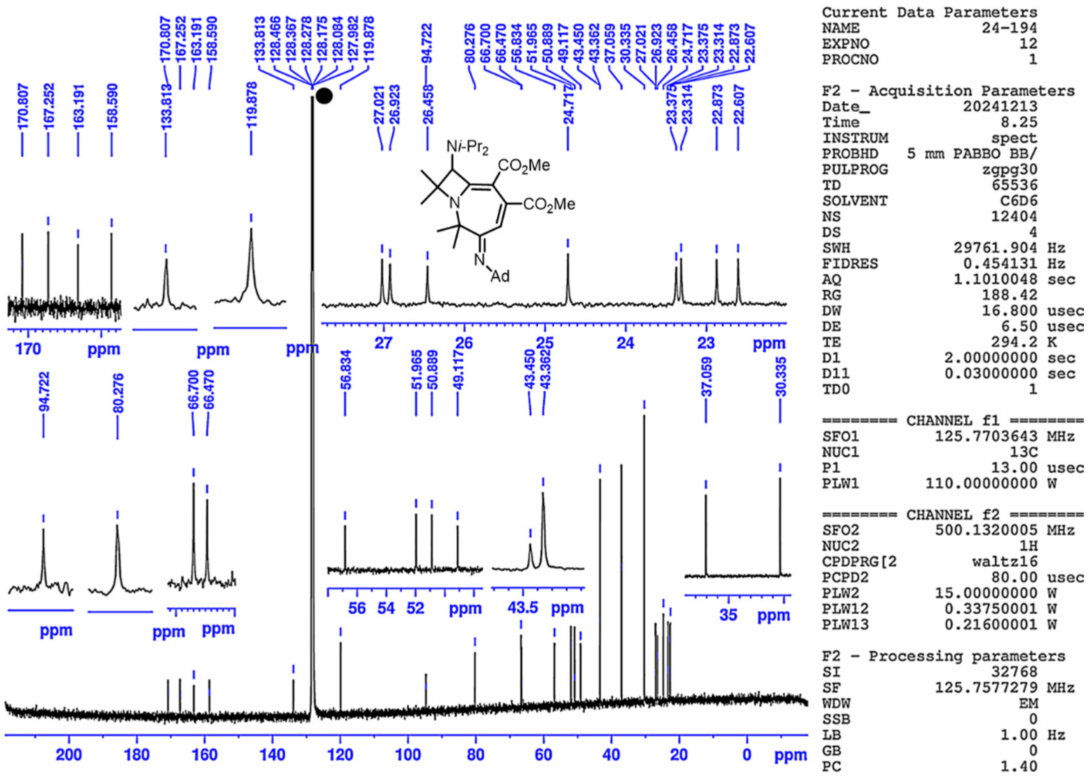

Figure S172. $^{13}C\{^{1}H\}$ NMR spectrum of **12** in C_6D_6 at 294 K (● = C_6D_6).

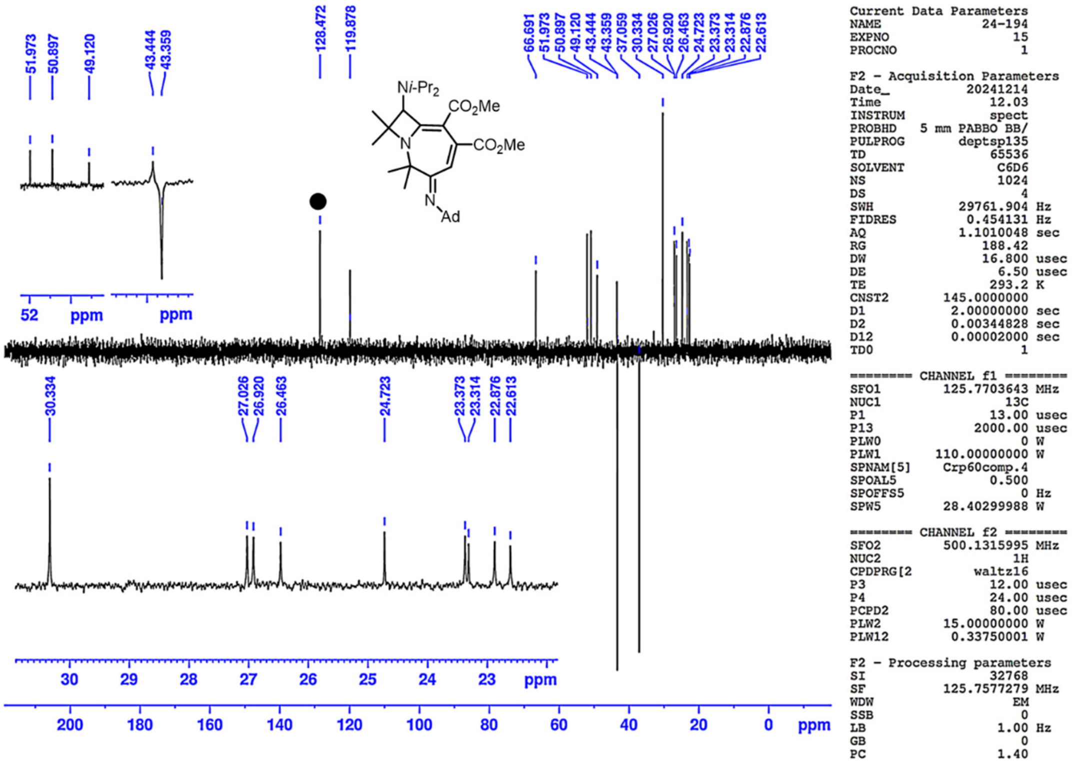

Figure S173. $^{13}C\{^{1}H\}$ (DEPT135) NMR spectrum of **12** in C_6D_6 at 293 K (● = C_6D_5H).

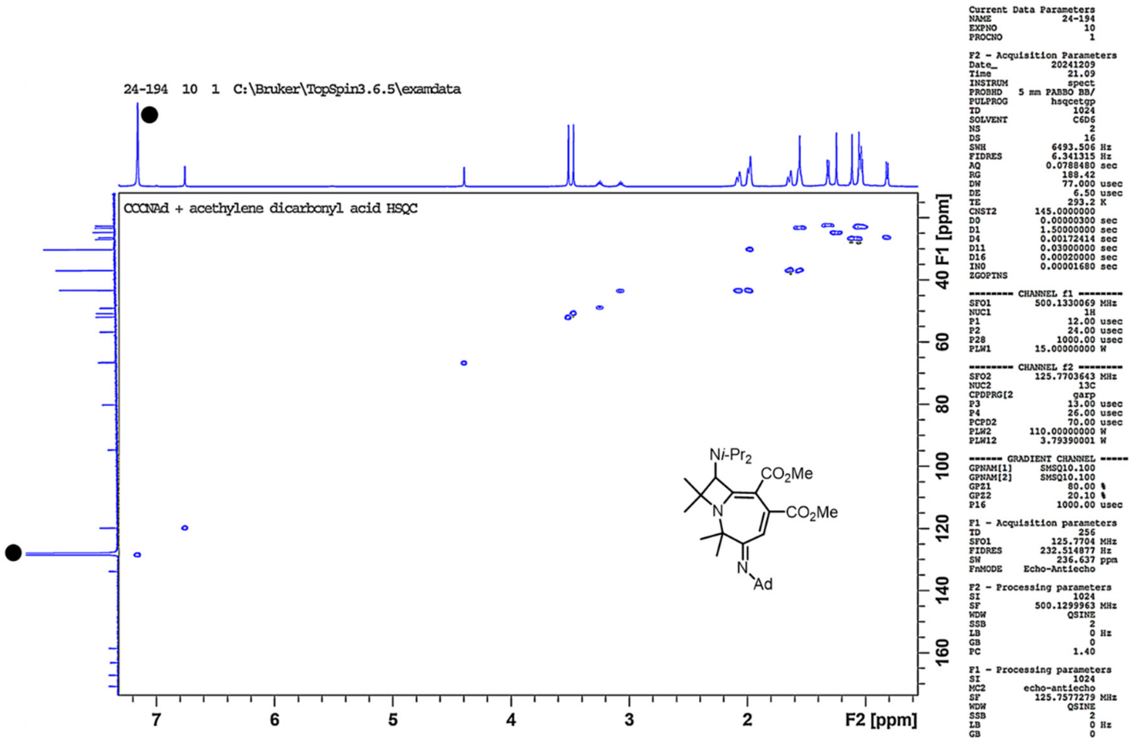

Figure S174. ^{1}H-^{13}C HSQC NMR spectrum of **12** in C_6D_6 at 293 K (● = C_6D_5H & C_6D_6).

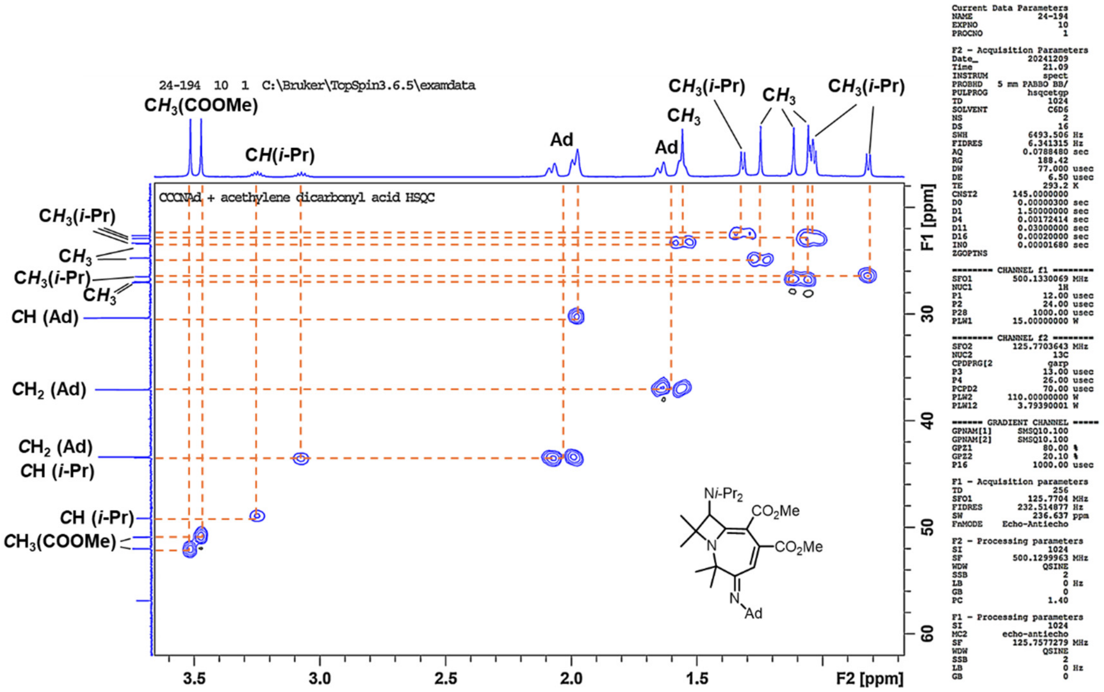

Figure S175. ^{1}H-^{13}C HSQC (magnified) NMR spectrum of **12** in C_6D_6 at 293 K.

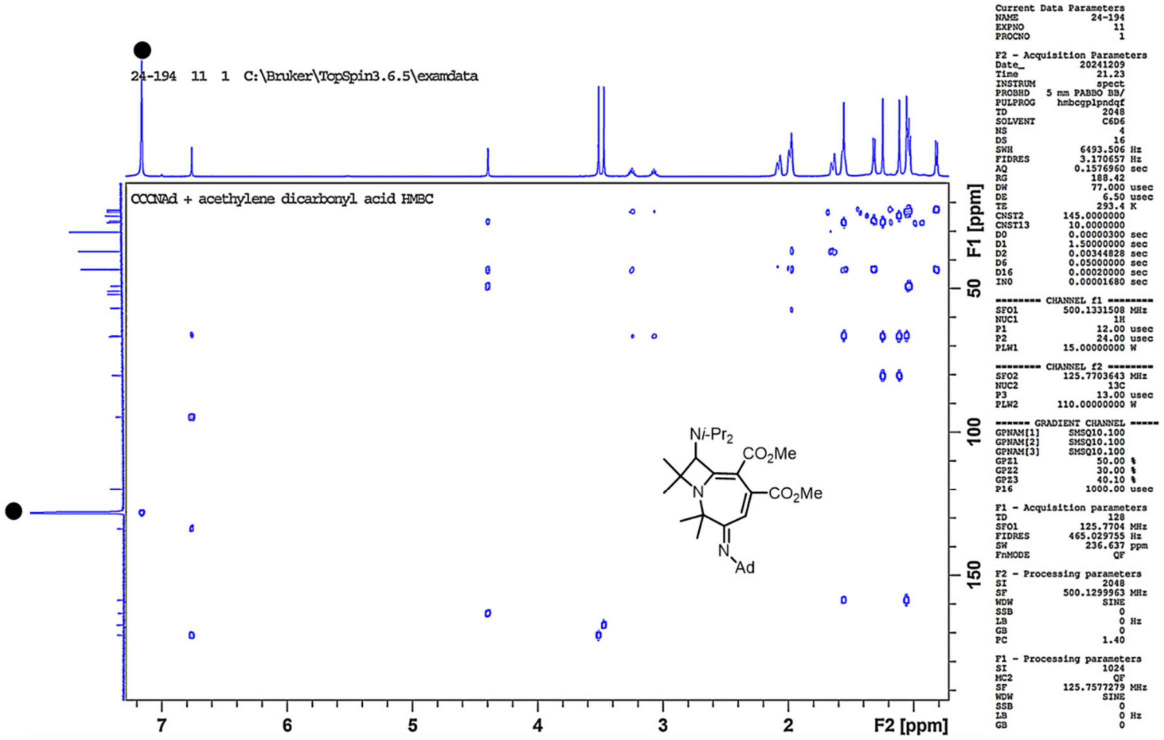

Figure S176. ^{1}H-^{13}C HMBC NMR spectrum of **12** in C_6D_6 at 293 K (● = C_6D_5H & C_6D_6).

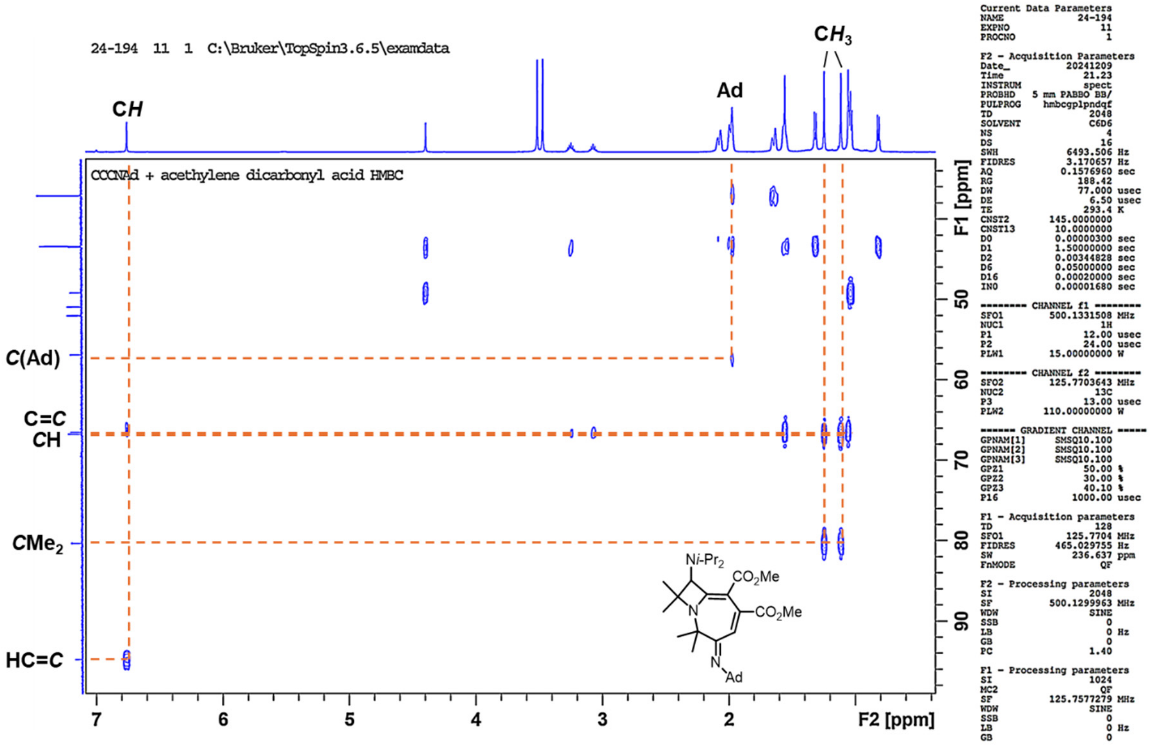

Figure S177. ^{1}H-^{13}C HMBC (magnified) NMR spectrum of **12** in C_6D_6 at 293 K.

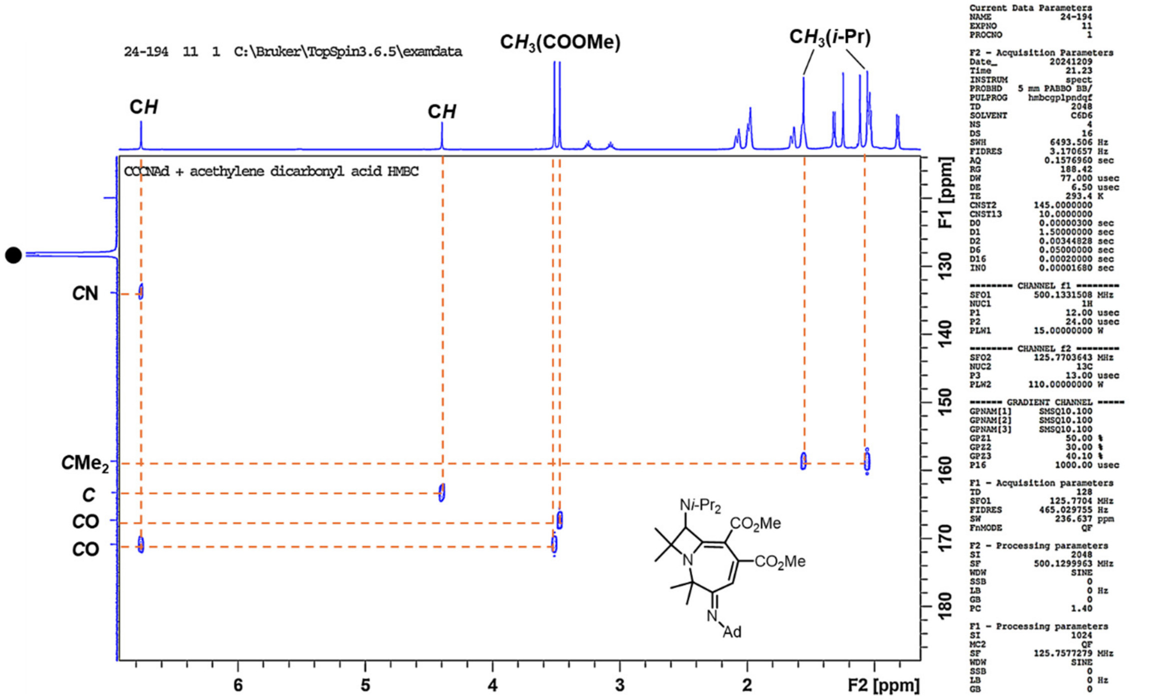

Figure S178. ^{1}H-^{13}C HMBC (magnified) NMR spectrum of **12** in C_6D_6 at 293 K (● = C_6D_6).

3. HRMS spectra

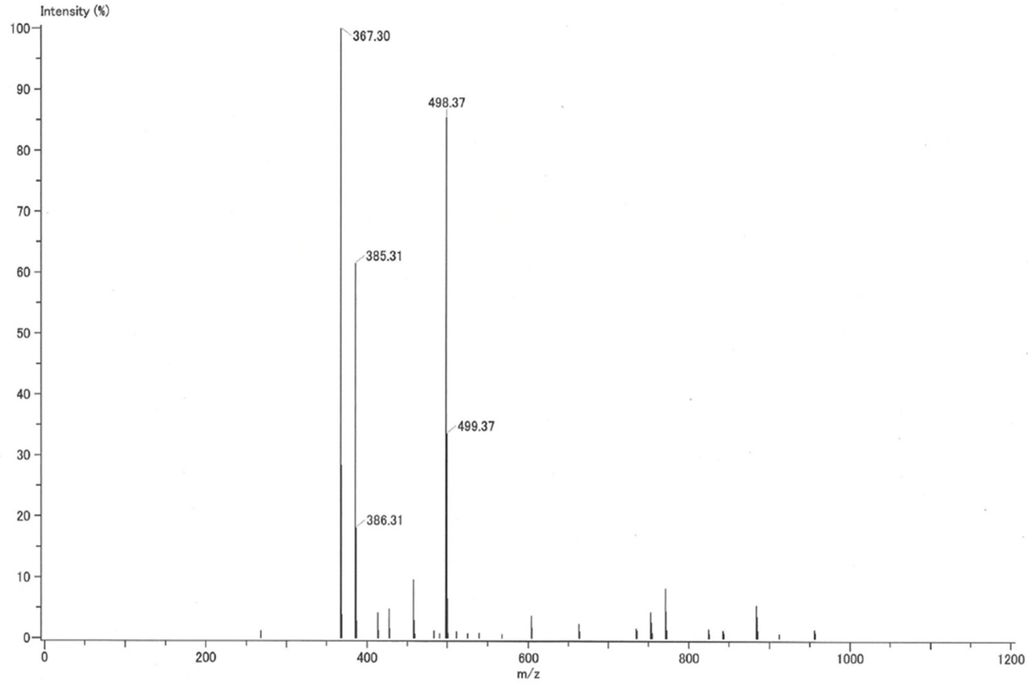

Figure S179. HRMS (FD+eiFi) spectrum of the mixture for the reaction of **BAC** with XylNC (1/4).

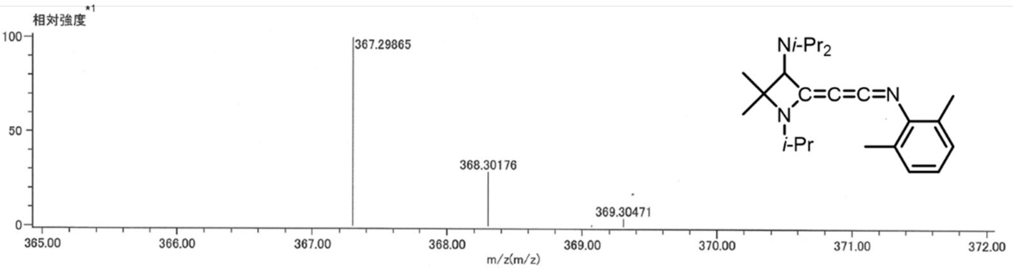

質量*2	強度*3	計算質量*4	質量差*5 mmu	質量差*6 ppm	推定組成式*7	不飽和数*8
367.29865	185893.48	367.29875	−0.10	−0.27	$^{12}C_{24}{}^{1}H_{37}{}^{14}N_3$	8.0

*1: Relative Intensity, *2: Measured Mass, *3: Intensity, *4: Calculated Mass, *5: Difference in Mass (mmu), *6: Difference in Mass (ppm), *7: Estimated Formula, *8: Degree of Unsaturation

Figure S180. HRMS (FD+eiFi) spectrum of the mixture for the reaction of **BAC** with XylNC (2/4).

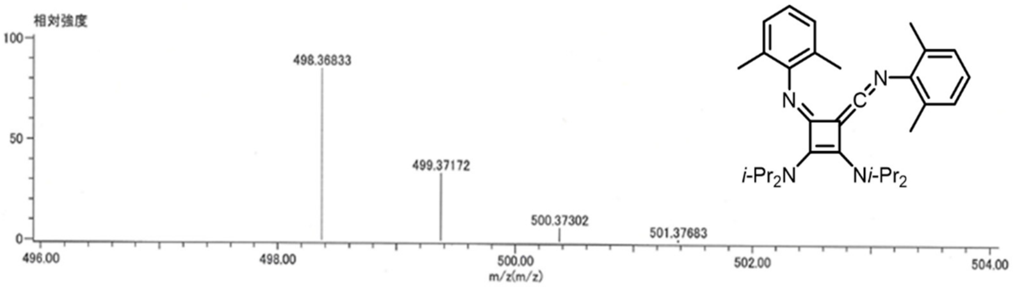

質量	強度	計算質量	質量差 mmu	質量差 ppm	推定組成式	不飽和数
498.36833	158775.64	498.37225	−3.92	−7.86	$^{12}C_{33}{}^{1}H_{46}{}^{14}N_4$	13.0

Figure S181. HRMS (FD+eiFi) spectrum of the mixture for the reaction of **BAC** with XylNC (3/4).

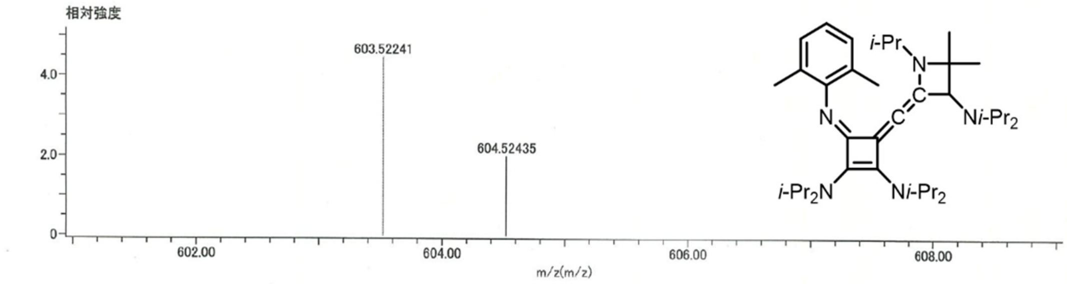

質量	強度	計算質量	質量差 mmu	質量差 ppm	推定組成式	不飽和数
603.52241	24234.88	603.52400	−1.58	−2.62	$^{12}C_{39}{}^{1}H_{65}{}^{14}N_5$	10.0

Figure S182. HRMS (FD+eiFi) spectrum of the mixture for the reaction of **BAC** with XylNC (4/4).

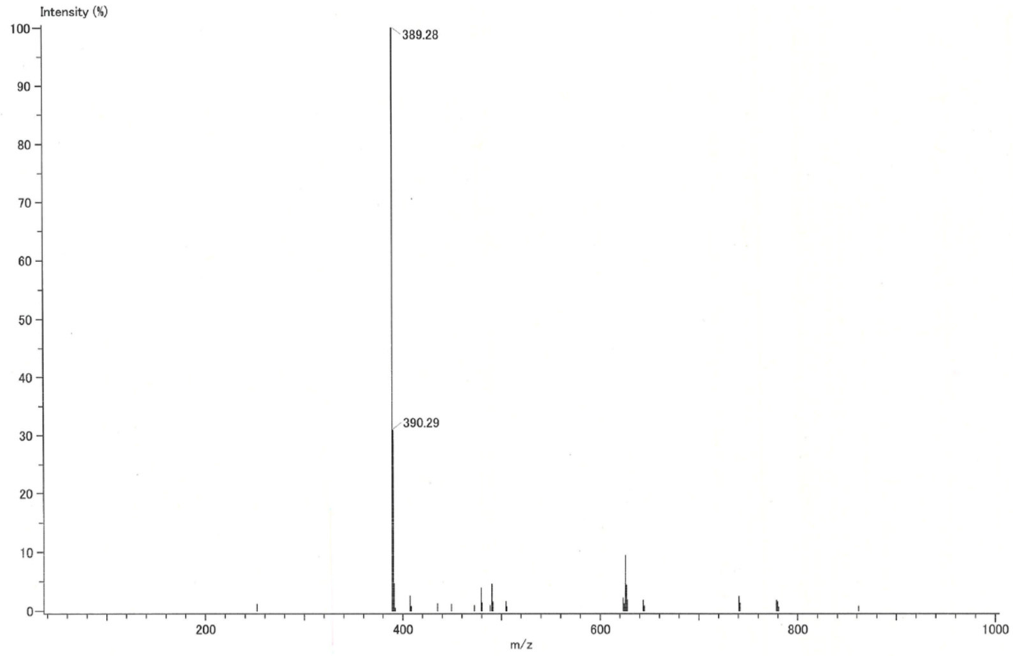

Figure S183. HRMS (FD+eiFi) spectrum of the mixture for the reaction of **BAC** with 2-naphthyl isocyanide (1/3).

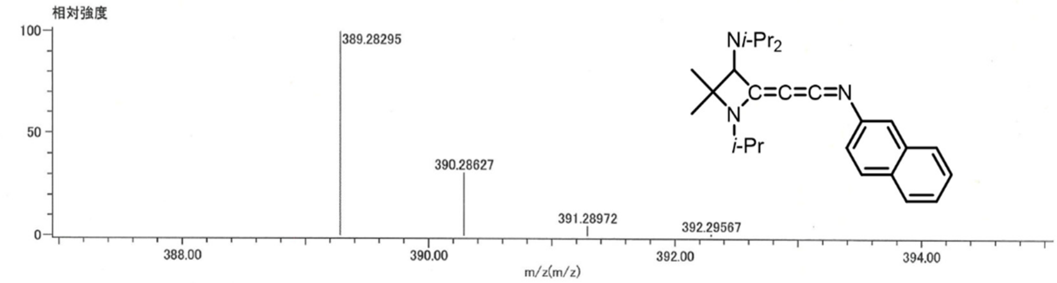

質量	強度	計算質量	質量差 mmu	質量差 ppm	推定組成式	不飽和数
389.28295	547906.98	389.28310	−0.15	−0.38	$^{12}C_{26}{}^{1}H_{35}{}^{14}N_3$	11.0

Figure S184. HRMS (FD+eiFi) spectrum of the mixture for the reaction of **BAC** with 2-naphthyl isocyanide (2/3).

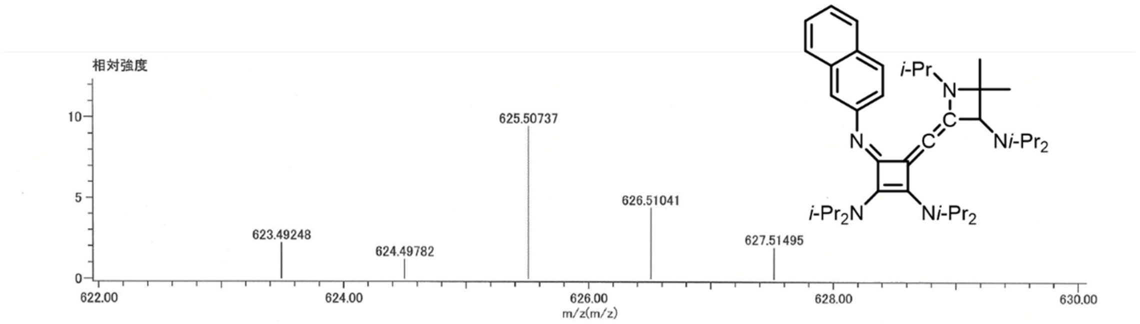

質量	強度	計算質量	質量差 mmu	質量差 ppm	推定組成式	不飽和数
625.50737	51665.83	625.50835	−0.98	−1.57	$^{12}C_{41}{}^{1}H_{63}{}^{14}N_5$	13.0

Figure S185. HRMS (FD+eiFi) spectrum of the mixture for the reaction of **BAC** with 2-naphthyl isocyanide (3/3).

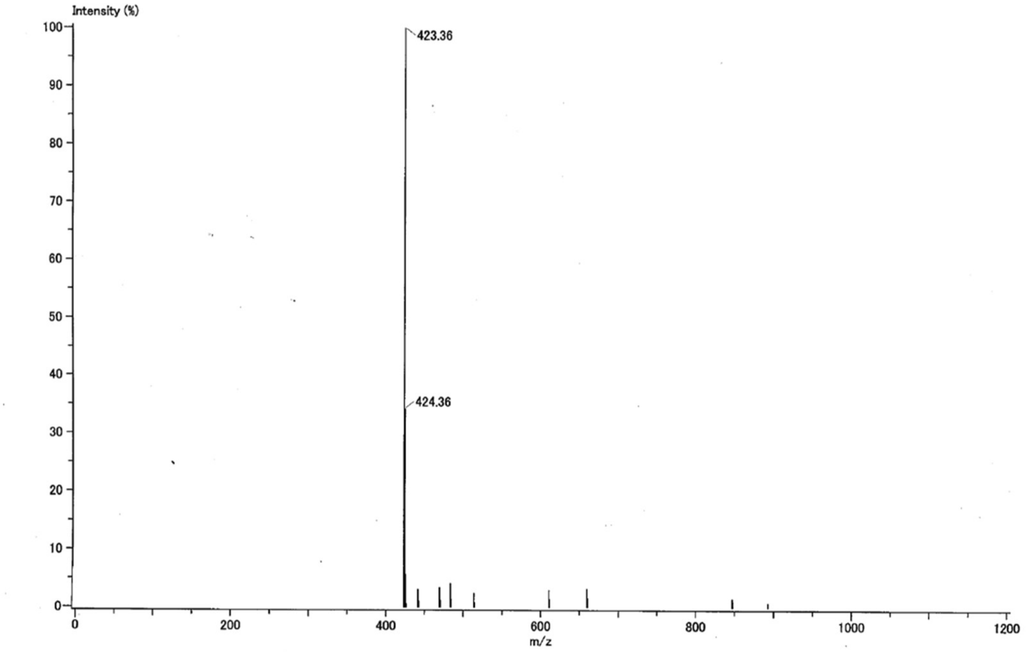

Figure S186. HRMS (FD+eiFi) spectrum of the mixture for the reaction of **BAC** with DippNC (1/4).

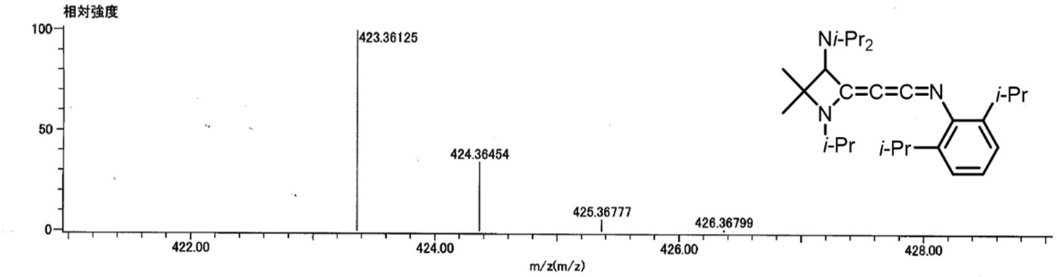

質量	強度	計算質量	質量差 mmu	質量差 ppm	推定組成式	不飽和数
423.36125	459556.33	423.36135	−0.10	−0.24	$^{12}C_{28}{}^{1}H_{45}{}^{14}N_3$	8.0

Figure S187. HRMS (FD+eiFi) spectrum of the mixture for the reaction of **BAC** with DippNC (2/4).

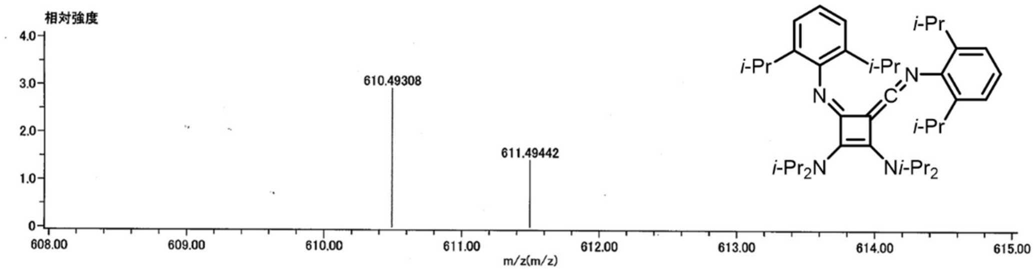

質量	強度	計算質量	質量差 mmu	質量差 ppm	推定組成式	不飽和数
610.49308	13402.66	610.49342	−0.34	−0.56	$^{12}C_{36}{}^{1}H_{62}{}^{14}N_{6}{}^{16}O_{2}$	9.0
		610.49477	−1.68	−2.76	$^{12}C_{38}{}^{1}H_{64}{}^{14}N_{3}{}^{16}O_{3}$	8.5
		` 610.49611	−3.02	−4.95	$^{12}C_{40}{}^{1}H_{66}{}^{16}O_{4}$	8.0
		610.49745	−4.36	−7.15	$^{12}C_{41}{}^{1}H_{62}{}^{14}N_{4}$	13.0
		610.49879	−5.70	−9.34	$^{12}C_{43}{}^{1}H_{64}{}^{14}N_{1}{}^{16}O_{1}$	12.5

Figure S188. HRMS (FD+eiFi) spectrum of the mixture for the reaction of **BAC** with DipNC (3/4).

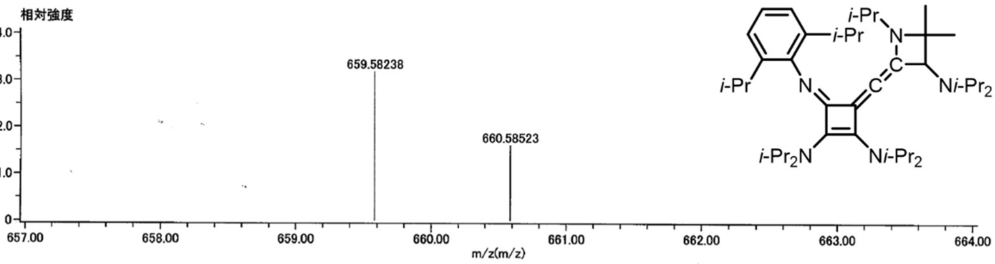

質量	強度	計算質量	質量差 mmu	質量差 ppm	推定組成式	不飽和数
659.58238	14571.44	659.58391	−1.54	−2.33	$^{12}C_{40}{}^{1}H_{75}{}^{14}N_{4}{}^{16}O_{3}$	5.5
		659.58526	−2.88	−4.37	$^{12}C_{42}{}^{1}H_{77}{}^{14}N_{1}{}^{16}O_{4}$	5.0
		` 659.58660	−4.22	−6.40	$^{12}C_{43}{}^{1}H_{73}{}^{14}N_{5}$	10.0
		659.58794	−5.56	−8.43	$^{12}C_{45}{}^{1}H_{75}{}^{14}N_{2}{}^{16}O_{1}$	9.5
		659.57670	5.67	8.60	$^{12}C_{46}{}^{1}H_{75}{}^{16}O_{2}$	9.5

Figure S189. HRMS (FD+eiFi) spectrum of the mixture for the reaction of **BAC** with DipNC (4/4).

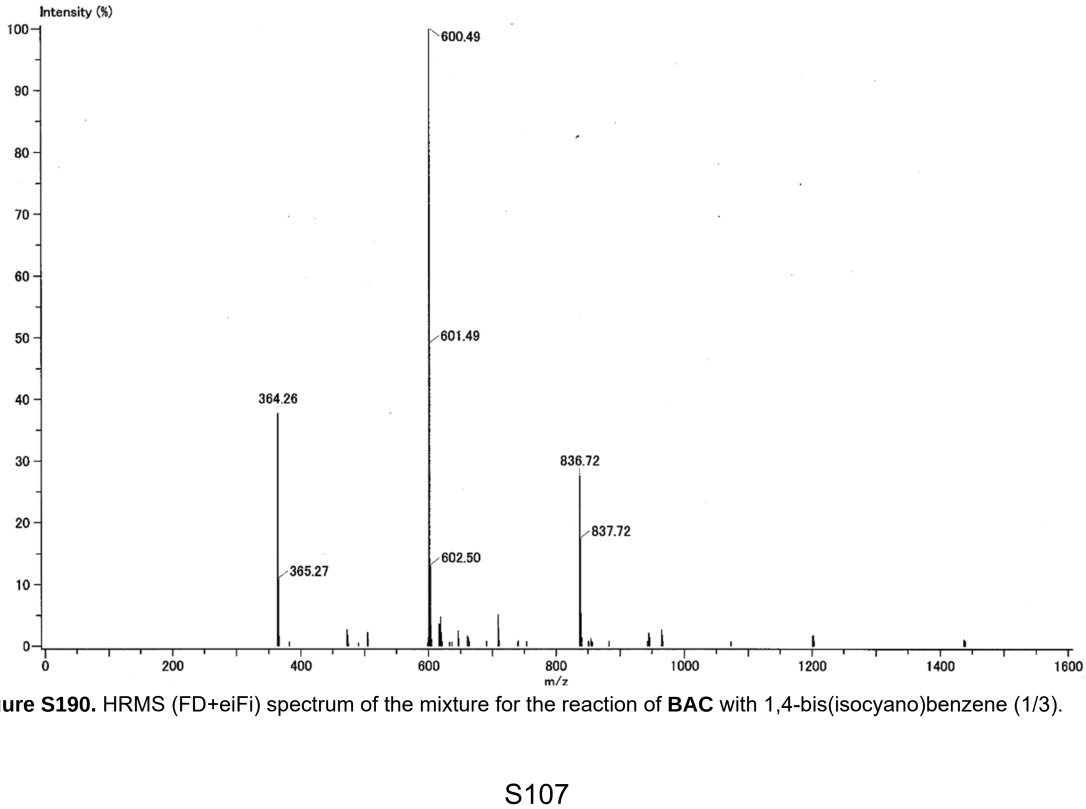

Figure S190. HRMS (FD+eiFi) spectrum of the mixture for the reaction of **BAC** with 1,4-bis(isocyano)benzene (1/3).

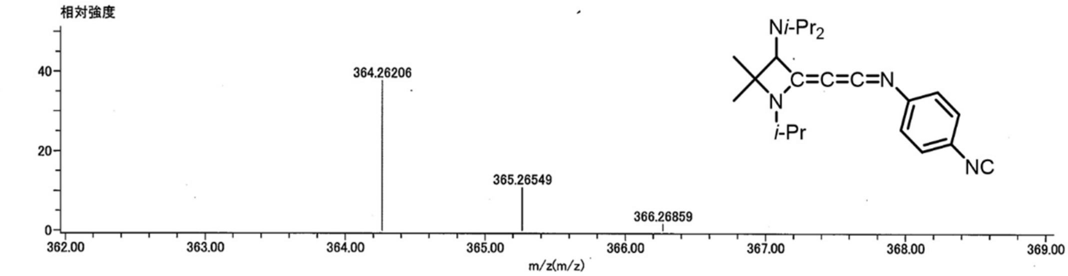

質量	強度	計算質量	質量差 mmu	質量差 ppm	推定組成式	不飽和数
364.26206	71150.84	364.26270	−0.63	−1.74	$^{12}C_{23}{}^{1}H_{32}{}^{14}N_4$	10.0

Figure S191. HRMS (FD+eiFi) spectrum of the mixture for the reaction of **BAC** with 1,4-bis(isocyano)benzene (2/3).

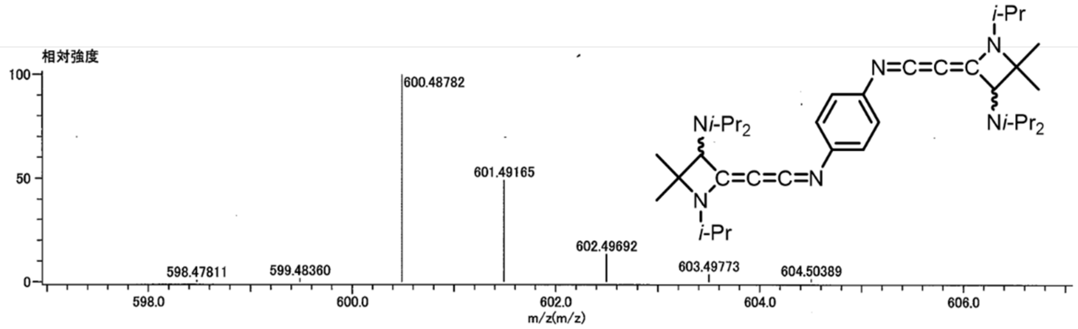

質量	強度	計算質量	質量差 mmu	質量差 ppm	推定組成式	不飽和数
600.48782	189039.99	600.48794	−0.12	−0.20	$^{12}C_{38}{}^{1}H_{60}{}^{14}N_6$	12.0

Figure S192. HRMS (FD+eiFi) spectrum of the mixture for the reaction of **BAC** with 1,4-bis(isocyano)benzene (3/3).

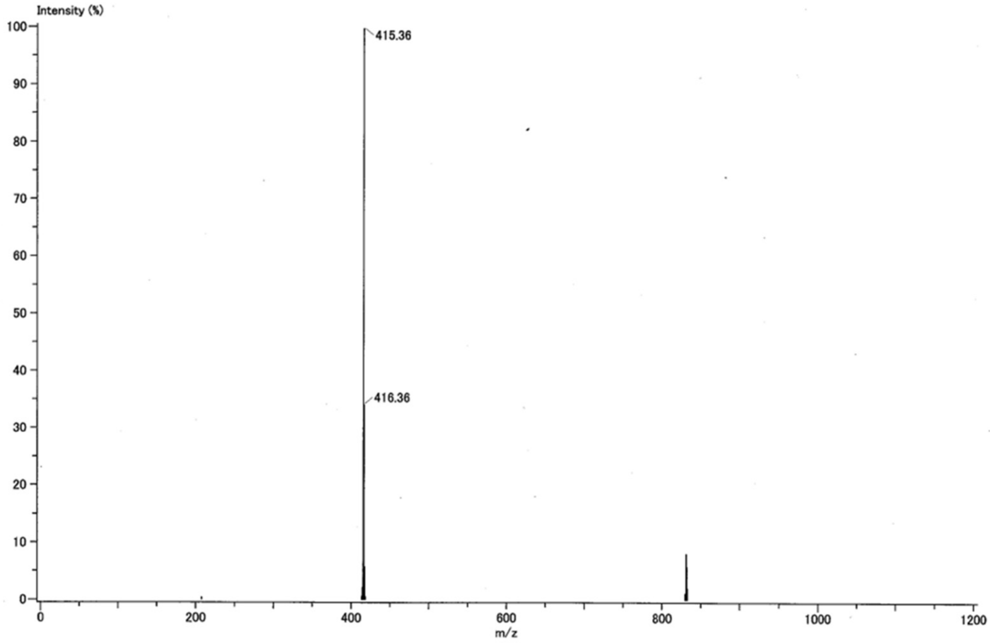

Figure S193. HRMS (FD+eiFi) spectrum of the mixture for the reaction of $\mathbf{1_{Ad}}$ with H_2O (1/2).

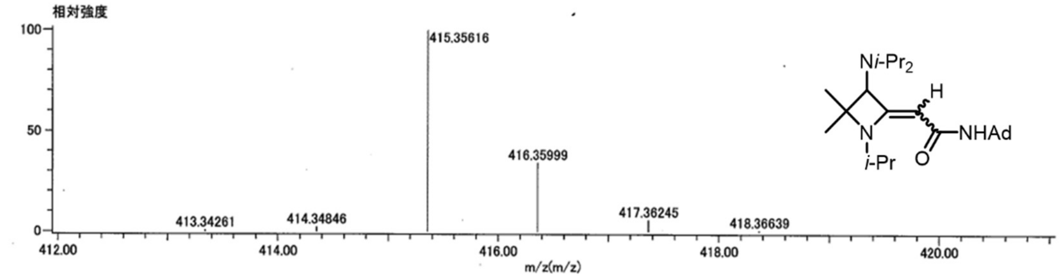

質量	強度	計算質量	質量差 mmu	質量差 ppm	推定組成式	不飽和数
415.35616	194374.53	415.35626	−0.10	−0.25	$^{12}C_{26}{}^{1}H_{45}{}^{14}N_{3}{}^{16}O_{1}$	6.0

Figure S194. HRMS (FD+eiFi) spectrum of the mixture for the reaction of $\mathbf{1}_{\mathbf{Ad}}$ with H_2O (2/2).

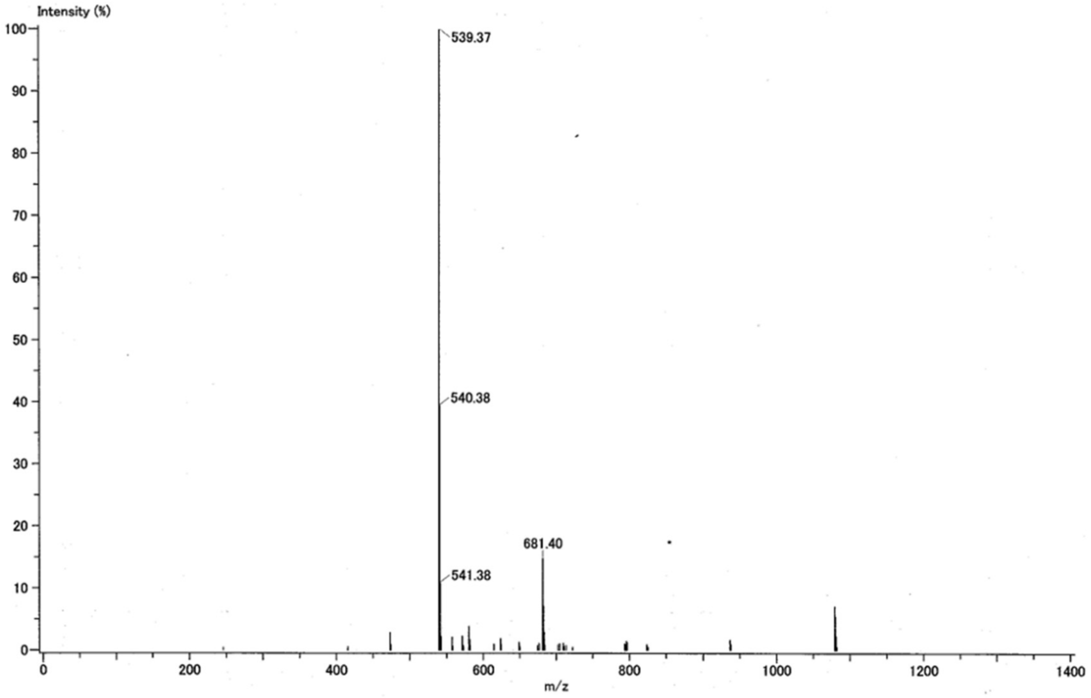

Figure S195. HRMS (FD+eiFi) spectrum of the mixture for the reaction of $\mathbf{1}_{\mathbf{Ad}}$ with dimethyl acetylenedicarboxylate (1/2).

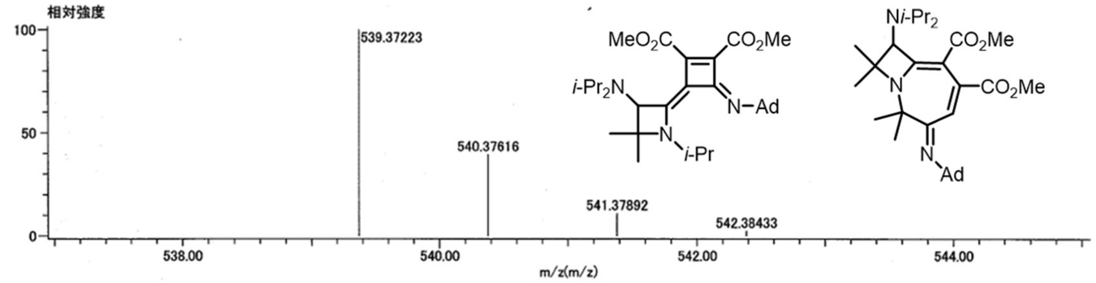

質量	強度	計算質量	質量差 mmu	質量差 ppm	推定組成式	不飽和数
539.37223	166118.39	539.37231	−0.08	−0.14	$^{12}C_{32}{}^{1}H_{49}{}^{14}N_{3}{}^{16}O_{4}$	10.0

Figure S196. HRMS (FD+eiFi) spectrum of the mixture for the reaction of $\mathbf{1}_{\mathbf{Ad}}$ with dimethyl acetylenedicarboxylate (2/2).

4. X-ray analysis

Single crystals suitable for X-ray diffraction analysis were obtained by recrystallization using the following conditions: from hexane at −23 °C in argon atmosphere for **1**$_{Ad}$; from hexane:$(SiMe_3)_2O$ (1:1) at −35 °C in argon atmosphere for **1**$_{Xyl}$; from the reaction mixture in hexane:Et_2O (1:1) solution at −23 °C in argon atmosphere for **2** and **3**; from the reaction mixture in Et_2O solution at room temperature in argon atmosphere for **4**; from CH_2Cl_2:THF (1:1) at −25 °C in argon atmosphere for **5**; from Et_2O at −23 °C in argon atmosphere for **6**; from hexane at −35 °C for **7**$_{Ad}$; from toluene at r.t. for **7**$_{Naph}$; from toluene at r.t. for **7**$_{Dip}$; from CH_2Cl_2 by vapor diffusion of hexane at room temperature for (*E*)-**8**; from hot hexane at room temperature for **9**; from toluene:hexane (1:3) at −35 °C in argon atmosphere for **10**; from Et_2O at −35 °C in argon atmosphere for **12**.

A single crystal for data collection coated by Paratone® N oil or Apiezon® grease and mounted on the glass fiber and then transferred to the cold gas stream of the diffractometer. X-ray diffraction data were collected on a Bruker D8 DISCOVER diffractometer with graphite monochromated Mo-Kα radiation or collected on Rigaku XtaLAB AFC10 diffractometer with a HyPix-6000 hybrid pixel array detector, graphite monochromated Mo-Kα radiation or collected on Rigaku XtaLAB Synergy diffractometer with HyPix-6000 hybrid pixel array detector, graphite monochromated Mo-Kα or Cu-Kα radiation. An empirical absorption correction based on the multiple measurements of equivalent reflections was applied using the program SADABS[74] or based on the CrysAlisPro 1.171.40.61a (Rigaku Oxford Diffraction, 2019) empirical absorption correction using spherical harmonics, implemented in SCALE3 ABSPACK scaling algorithm or CrysAlisPro 1.171.42.98a (Rigaku Oxford Diffraction, 2023) empirical absorption correction using spherical harmonics, implemented in SCALE3 ABSPACK scaling algorithm. The structures were solved by direct methods and refined by full-matrix least squares against F^2 using all data (SHELXL-2018, SHELXL-2019).[75] Molecular structures were analyzed by Yadokari-XG software.[76,77]

Crystal data of **1**$_{Xyl}$ (100 K): $C_{48}H_{74}N_6$; Fw 735.13; triclinic; space group $P\bar{1}$, a = 9.3480(2) Å, b = 13.7856(2) Å, c = 19.9212(3) Å, α = 70.460(1)°, β = 83.008(1)°, γ = 71.589(2)°, V = 2295.15(7), Z = 2, R1 = 0.0536, wR2 = 0.1313, GOF = 1.027.

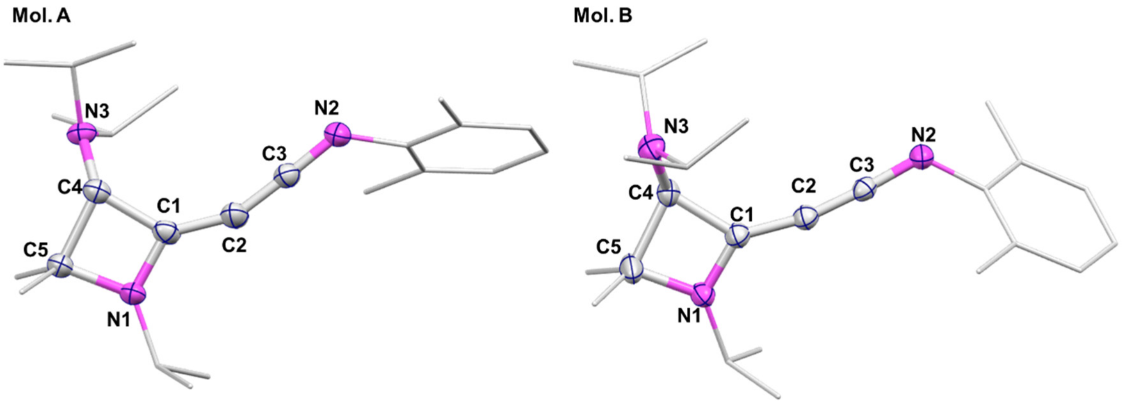

Figure S197. Molecular structure of **1**$_{Xyl}$ with thermal ellipsoids set at 50% probability. H atoms are omitted for clarity. Two crystallographically independent molecules exist in the asymmetric unit.

Crystal data of $\mathbf{1_{Ad}}$ (100 K): $C_{26}H_{43}N_3$; Fw 397.63; Monoclinic; $P2_1/a$, a = 16.176(2) Å, b = 7.4833(10) Å, c = 20.441(3) Å, β = 97.914(4)°, V = 2452.5(6), Z = 4, $R1$ = 0.0977 ($I > 2\sigma(I)$), $wR2$ = 0.2693, GOF = 1.067.

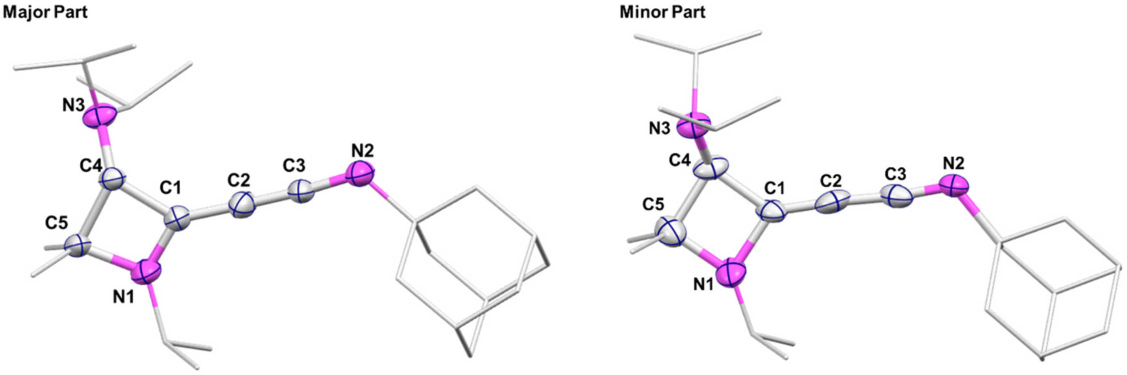

Figure S198. Molecular structure of $\mathbf{1_{Ad}}$ with thermal ellipsoids set at 50% probability. H atoms are omitted for clarity. Disorders were observed in the core structure (major part: 66%, minor part: 34%).

Crystal data of **2** (100 K): $C_{66}H_{92}N_8$; Fw 997.47; Monoclinic; $P2_1/c$, a = 15.6356(3) Å, b = 18.1248(3) Å, c = 22.3597(3) Å, β = 104.852(2)°, V = 6124.86(18) Å^3, Z = 4, $R1$ = 0.0394 ($I > 2\sigma(I)$), $wR2$ = 0.1076 (all data), GOF = 1.022.

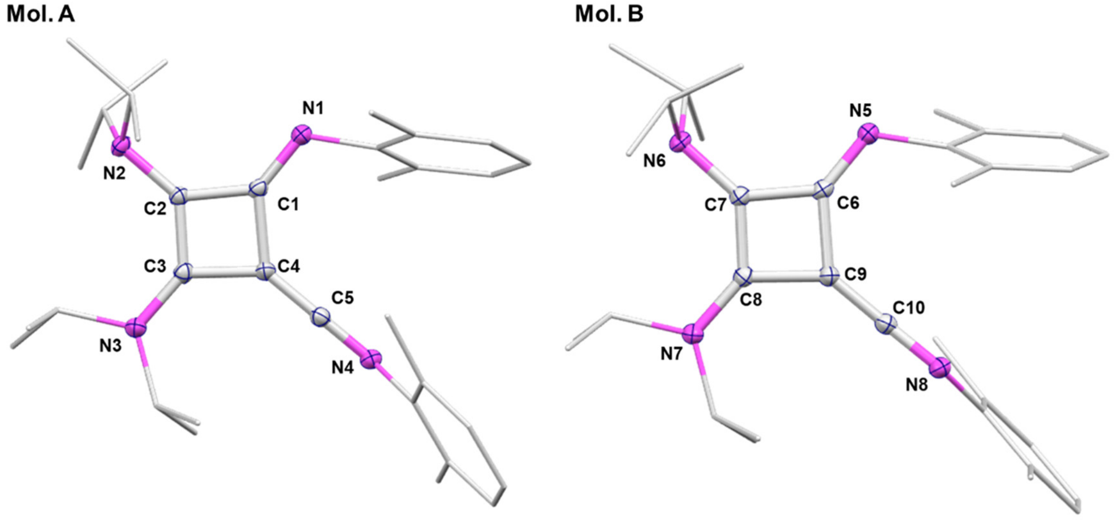

Figure S199. Molecular structure of **2** with thermal ellipsoids set at 50% probability. H atoms are omitted for clarity. Two crystallographically independent molecules exist in the asymmetric unit.

Crystal data of **3** (100 K): $C_{39}H_{65}N_5$; Fw 603.96; Triclinic; $P\bar{1}$, *a* = 11.4591(3) Å, *b* = 12.3744(3) Å, *c* = 13.9601(4) Å, *α* = 85.587(2)°, *β* = 79.321(2)°, *γ* = 82.005(2)°, *V* = 1923.68(9) Å^3, *Z* = 2, *R*1 = 0.0496 (*I* > 2σ(*I*)), *wR*2 = 0.1394 (all data), GOF = 1.044.

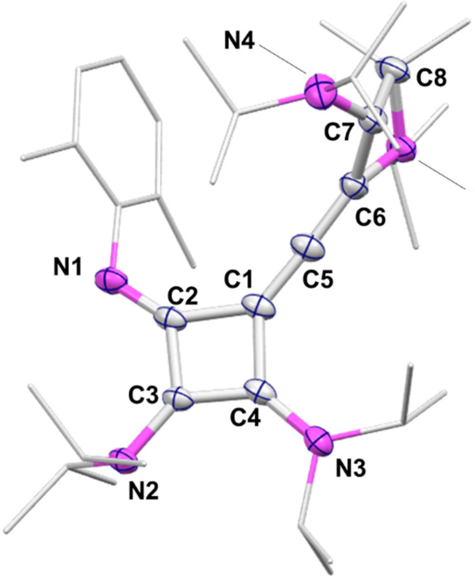

Figure S200. Molecular structure of **3** with thermal ellipsoids set at 50% probability. H atoms are omitted for clarity. Disorder was observed for the isopropyl substituents.

Crystal data of **4** (120 K): $C_{38}H_{60}N_6$; Fw 600.92; Monoclinic; $P2_1/c$, *a* = 9.8774(2) Å, *b* = 15.3884(3) Å, *c* = 12.3541(2) Å, *β* = 94.978(2)°, *V* = 1870.71(6) Å^3, *Z* = 2, *R*1 = 0.0683 (*I* > 2σ(*I*)), *wR*2 = 0.1916, GOF = 1.076.

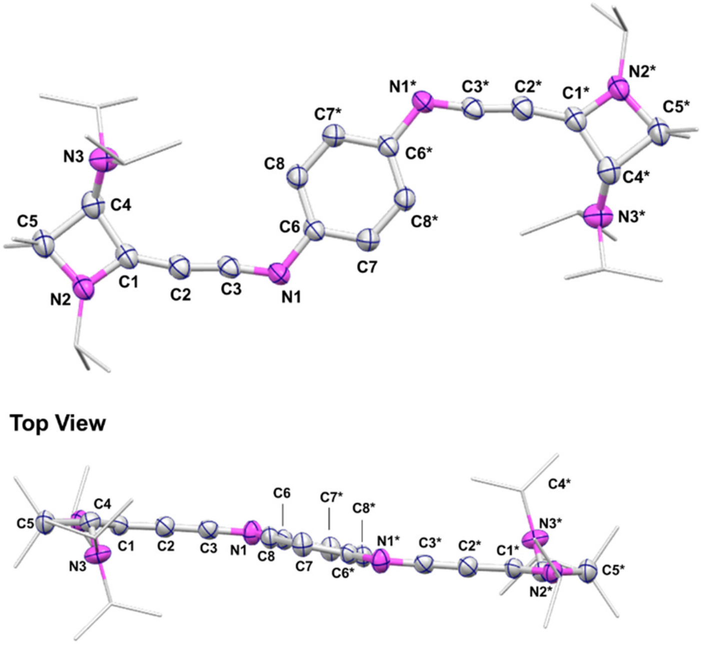

Figure S201. Molecular structure of **4** with thermal ellipsoids set at 50% probability. H atoms are omitted for clarity.

Crystal data of **5** (200 K): $C_{64}H_{110}Au_2Cl_2N_3$; Fw 1476.41; Triclinic; $P\bar{1}$, a = 13.7907(3) Å, b = 16.4400(4) Å, c = 18.3132(3) Å, α = 64.677(2)°, β = 89.598(2)°, γ = 66.253(2)° V = 3366.05(14) Å^3, Z = 2, R1 = 0.0274 ($I > 2\sigma(I)$), wR2 = 0.0701, GOF = 1.024.

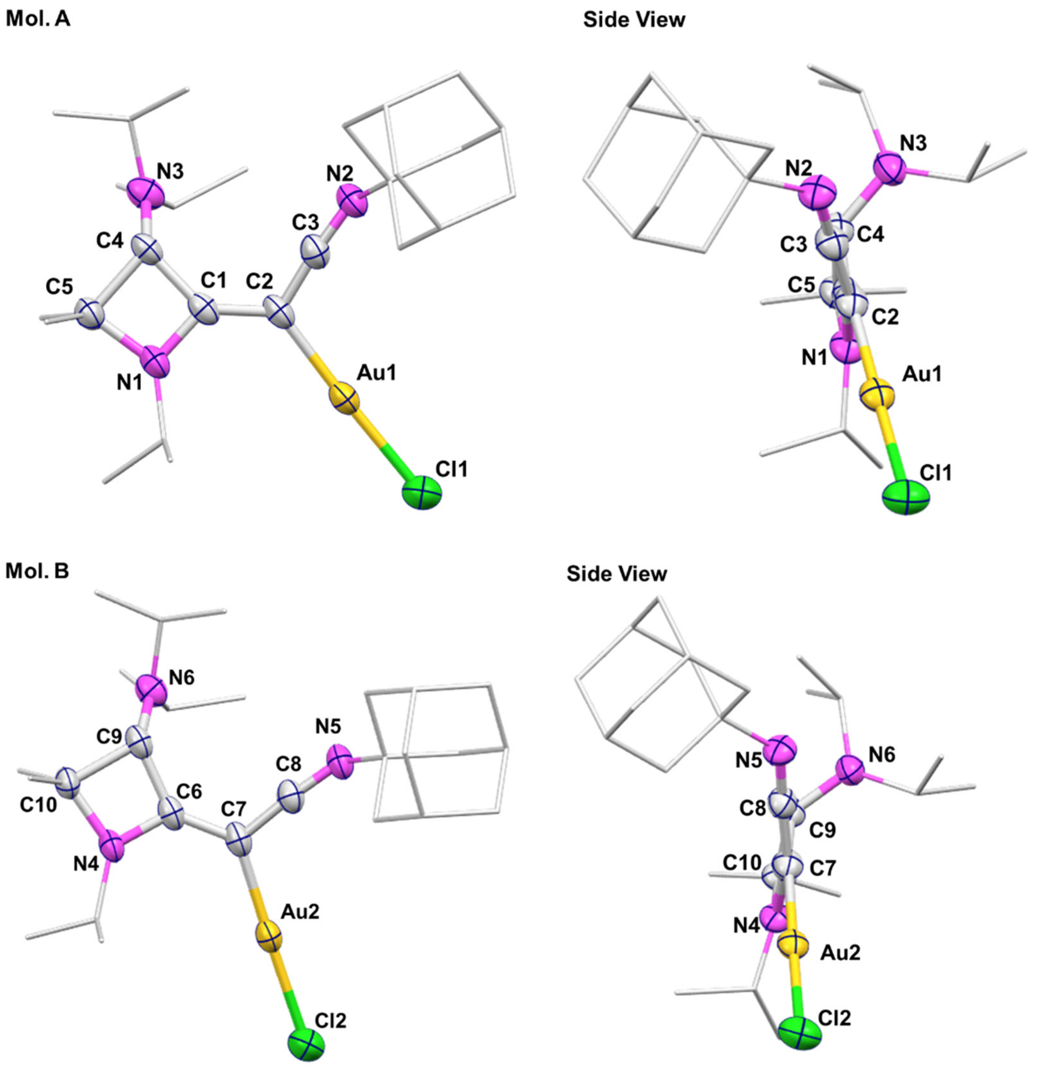

Figure S202. Molecular structure of **5** with thermal ellipsoids set at 50% probability. H atoms are omitted for clarity. Two crystallographically independent molecules exist in the asymmetric unit.

Crystal data of **6** (100 K): $C_{28}H_{43}ClN_3O_2Rh$; Fw 592.01; Monoclinic; $P2_1/c$, a = 10.8388(2) Å, b = 13.1607(2) Å, c = 20.5434(3) Å, β = 101.1890(10)°, V = 2874.74(8) Å^3, Z = 4, $R1$ = 0.0236 ($I > 2\sigma(I)$), $wR2$ = 0.0590, GOF = 1.054.

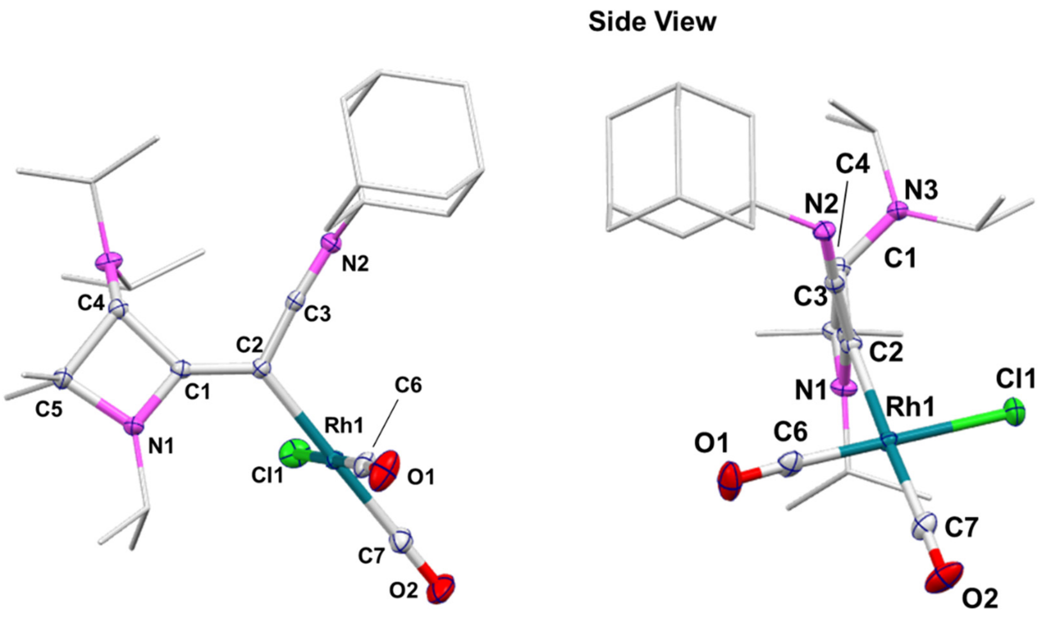

Figure S203. Molecular structure of **6** with thermal ellipsoids set at 50% probability. H atoms are omitted for clarity.

Crystal data of $\mathbf{7_{Ad}}$ (100 K): $C_{27}H_{43}N_3O_2$; Fw 441.64; Monoclinic; $P2_1/c$, a = 11.1789(2) Å, b = 22.2670(5) Å, c = 10.0623(2) Å, β = 95.307(2)°, V = 2493.98(9) Å^3, Z = 4, $R1$ = 0.0418 ($I > 2\sigma(I)$), $wR2$ = 0.1122, GOF = 1.067.

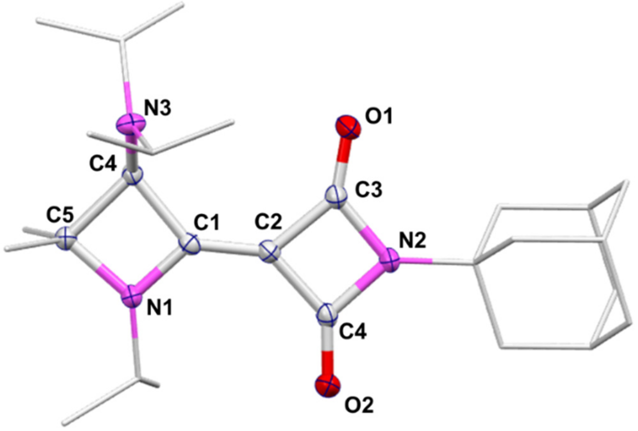

Figure S204. Molecular structure of $\mathbf{7_{Ad}}$ with thermal ellipsoids set at 50% probability. H atoms are omitted for clarity.

Crystal data of $\mathbf{7}_{\mathbf{Naph}}$ (120 K): $C_{27}H_{35}N_3O_2$; Fw 433.58; Triclinic; $P\bar{1}$, a = 9.9583(3) Å, b = 11.2228(4) Å, c = 11.9263(3) Å, α = 70.269(3)°, β = 76.379(2)°, γ = 86.134(2)°, V = 1219.23(7) Å^3, Z = 2, R1 = 0.0393 ($I > 2\sigma(I)$), wR2 = 0.1077, GOF = 1.065.

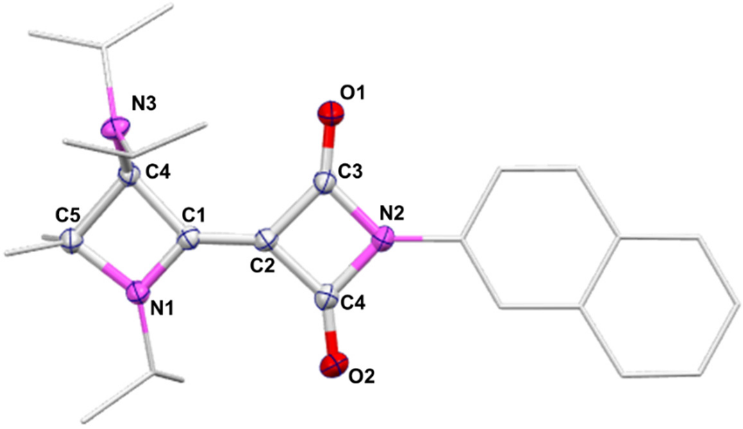

Figure S205. Molecular structure of $\mathbf{7}_{\mathbf{Naph}}$ with thermal ellipsoids set at 50% probability. H atoms are omitted for clarity.

Crystal data of $\mathbf{7}_{\mathbf{Dipp}}$ (100 K): $C_{29}H_{45}N_3O_2$; Fw 467.68; Monoclinic; $P2_1/c$, a = 11.0241(5) Å, b = 17.8554(8) Å, c = 14.0383(6) Å, β = 97.460(2)°, V = 2739.8(2) Å^3, Z = 4, R1 = 0.0553 ($I > 2\sigma(I)$), wR2 = 0.1529, GOF = 1.040.

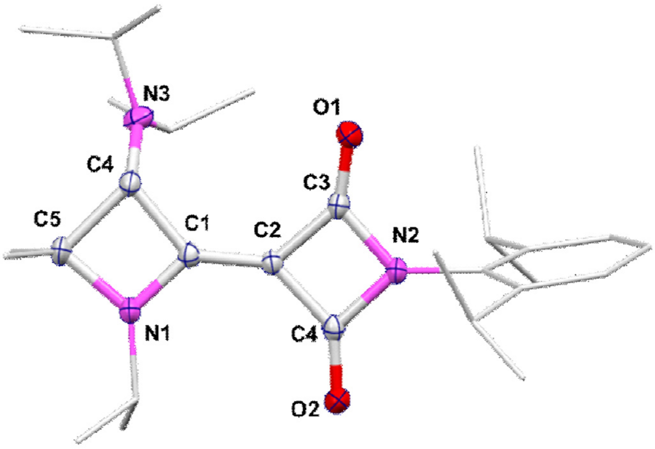

Figure S206. Molecular structure of $\mathbf{7}_{\mathbf{Dipp}}$ with thermal ellipsoids set at 50% probability. H atoms are omitted for clarity.

Crystal data of **8** (100 K): $C_{35}H_{52}N_4O$; Fw 544.80; Orthorhombic; space group $P2_12_12_1$, a = 12.2620(2) Å, b = 13.6639(4) Å, c = 18.9294(7) Å, V = 3171.56(16), Z = 4, $R1$ = 0.0285 ($I > 2\sigma(I)$), $wR2$ = 0.0752, GOF = 1.034.

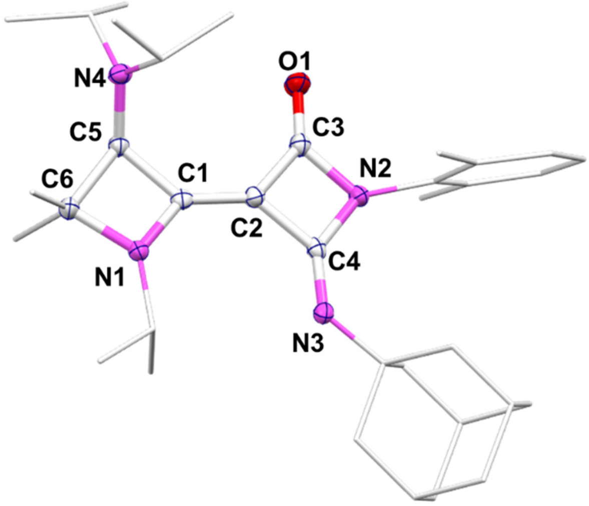

Figure S207. Molecular structure of **8** with thermal ellipsoids set at 50% probability. H atoms are omitted for clarity.

Crystal data of **9** (100 K): $C_{35}H_{52}N_4O$; Fw 544.80; Monoclinic; space group $P2_1/c$, a = 24.2277(4) Å, b = 15.2314(2) Å, c = 17.1128(5) Å, β = 94.405(2)°, V = 6296.3(2), Z = 8, $R1$ = 0.0366 ($I > 2\sigma(I)$), $wR2$ = 0.0998, GOF = 1.013.

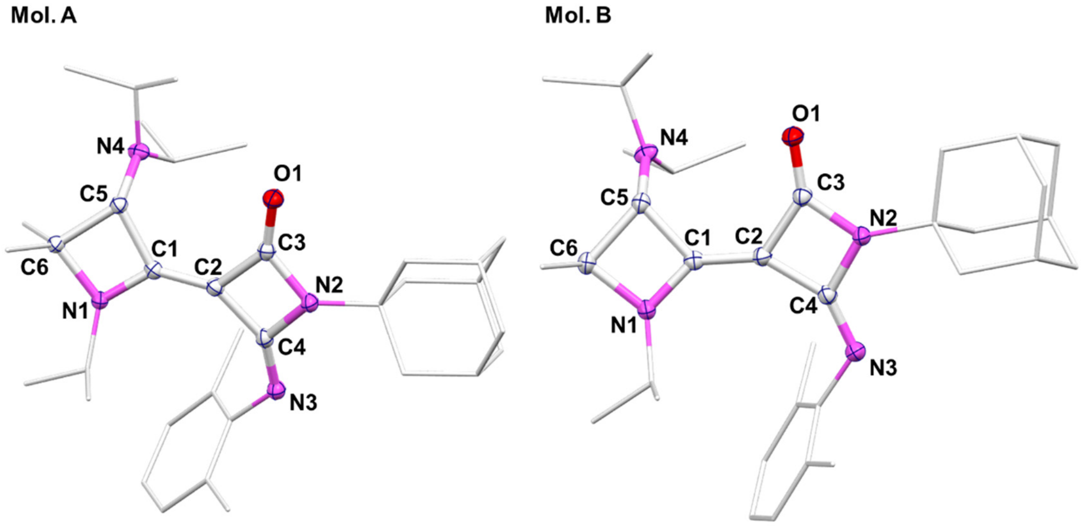

Figure S208. Molecular structure of **9** with thermal ellipsoids set at 50% probability. H atoms are omitted for clarity. Two crystallographically independent molecules exist in the asymmetric unit.

Crystal data of **10** (100 K): $C_{37.5}H_{56}N_6$; Fw 590.88; Triclinic; space group $P\bar{1}$, a = 9.0685(2) Å, b = 11.9619(3) Å, c = 16.2345(4) Å, α = 79.823(2)°, β = 88.108(2)°, γ = 83.596(2)°, V = 1722.38(7), Z = 2, $R1$ = 0.0415 ($I > 2\sigma(I)$), $wR2$ = 0.1109, GOF = 1.052.

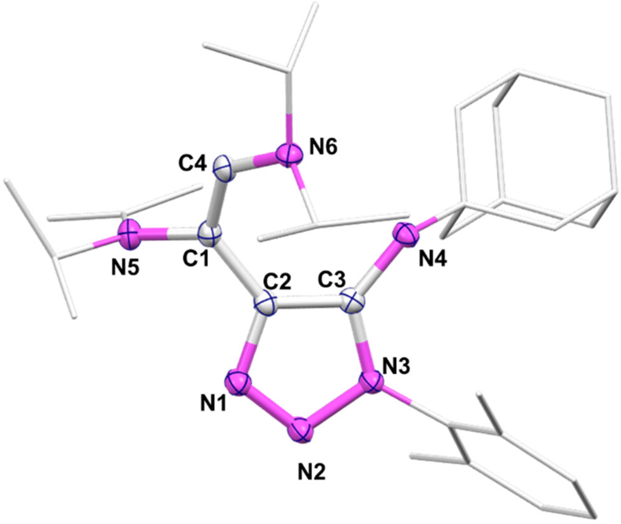

Figure S209. Molecular structure of **10** with thermal ellipsoids set at 50% probability. H atoms and the 0.5 toluene molecule are omitted for clarity.

Crystal data of **12** (100 K): $C_{32}H_{49}N_3O_4$; Fw 539.74; Triclinic; space group $P\bar{1}$, a = 11.4200(2) Å, b = 11.7526(2) Å, c = 12.3832(1) Å, α = 71.918(1)°, β = 89.881(1)°, γ = 71.233(2)°, V = 1487.08(4), Z = 2, $R1$ = 0.0412 ($I > 2\sigma(I)$), $wR2$ = 0.1193, GOF = 1.031.

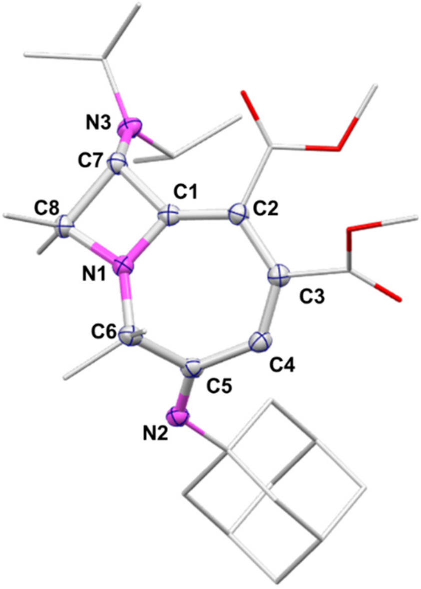

Figure S210. Molecular structure of **12** with thermal ellipsoids set at 50% probability. H atoms are omitted for clarity.

5. UV-vis Absorption Spectra

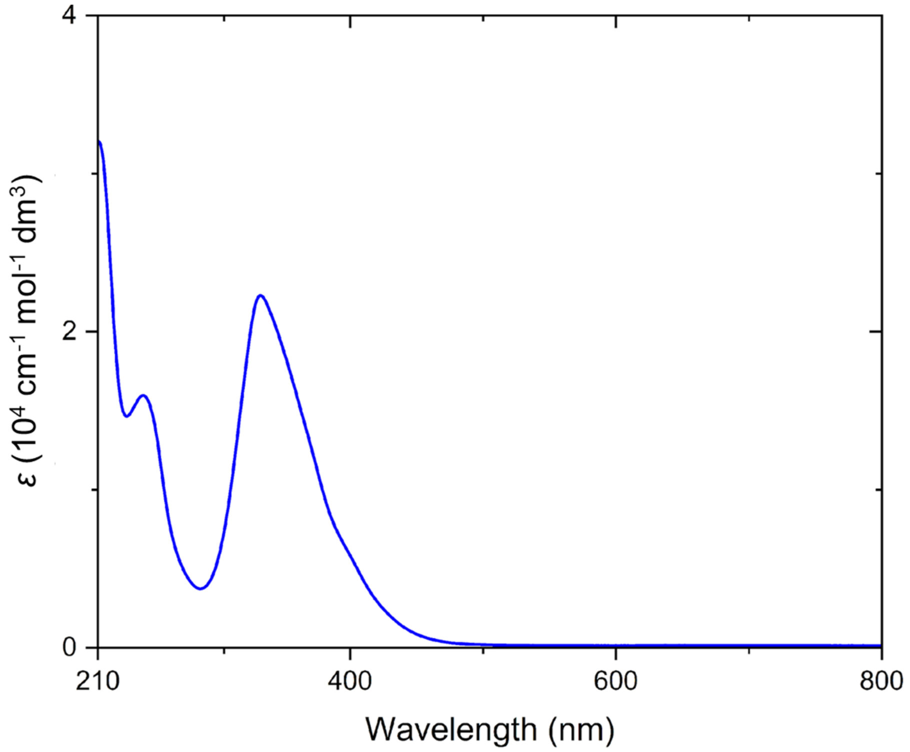

Figure S211. UV-vis absorption spectrum of $\mathbf{1}_{Xyl}$ in THF at 293 K.

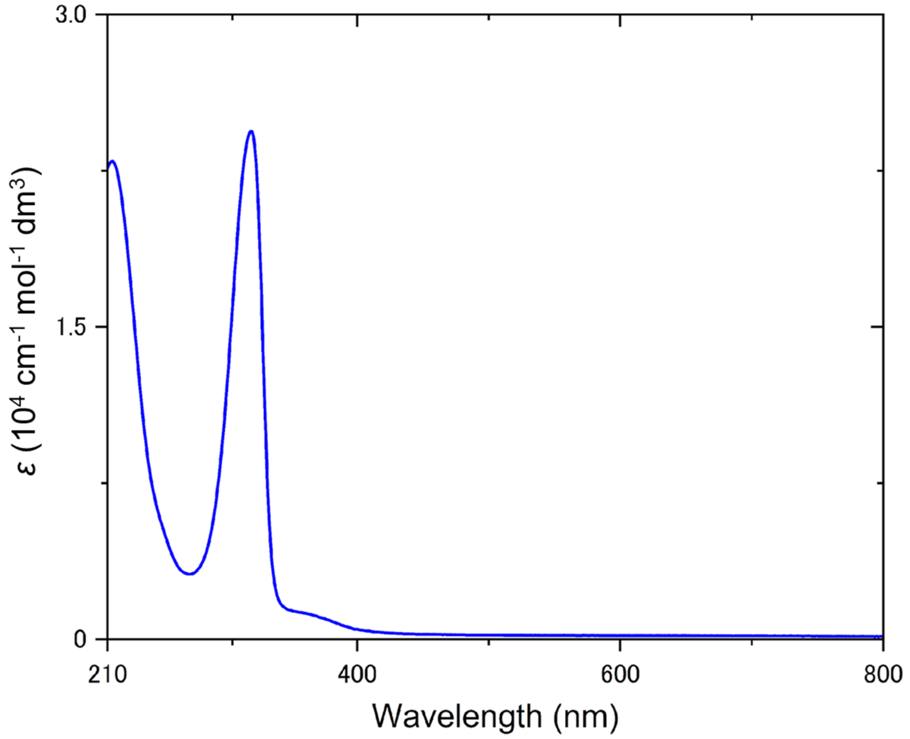

Figure S212. UV-vis absorption spectrum of $\mathbf{1}_{Ad}$ in hexane at 293 K.

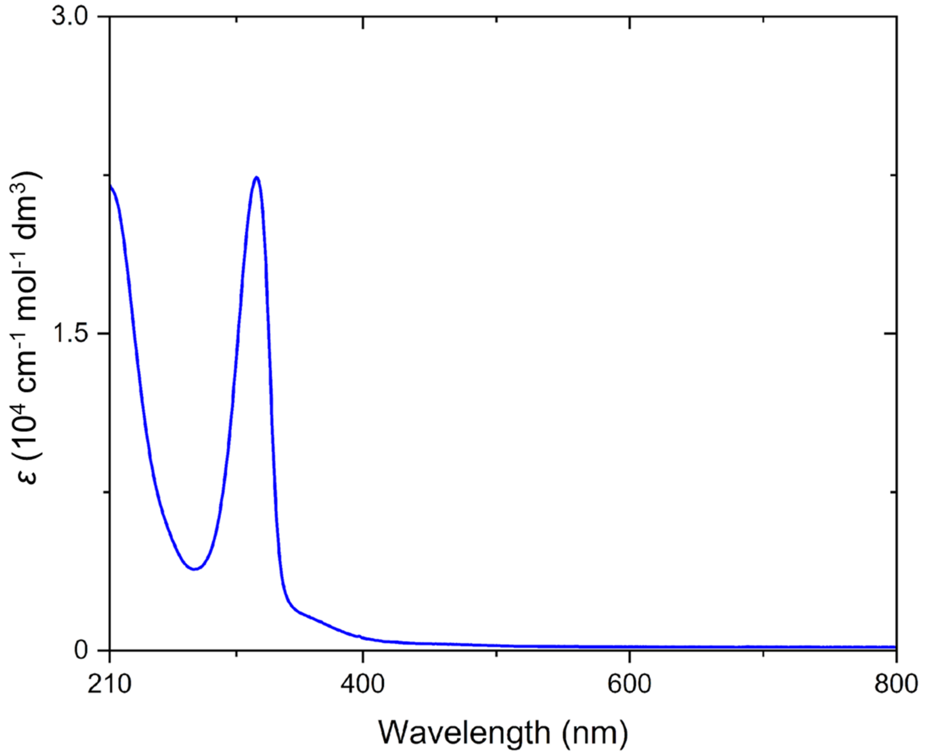

Figure S213. UV-vis absorption spectrum of $\mathbf{1_{Ad}}$ in THF at 293 K.

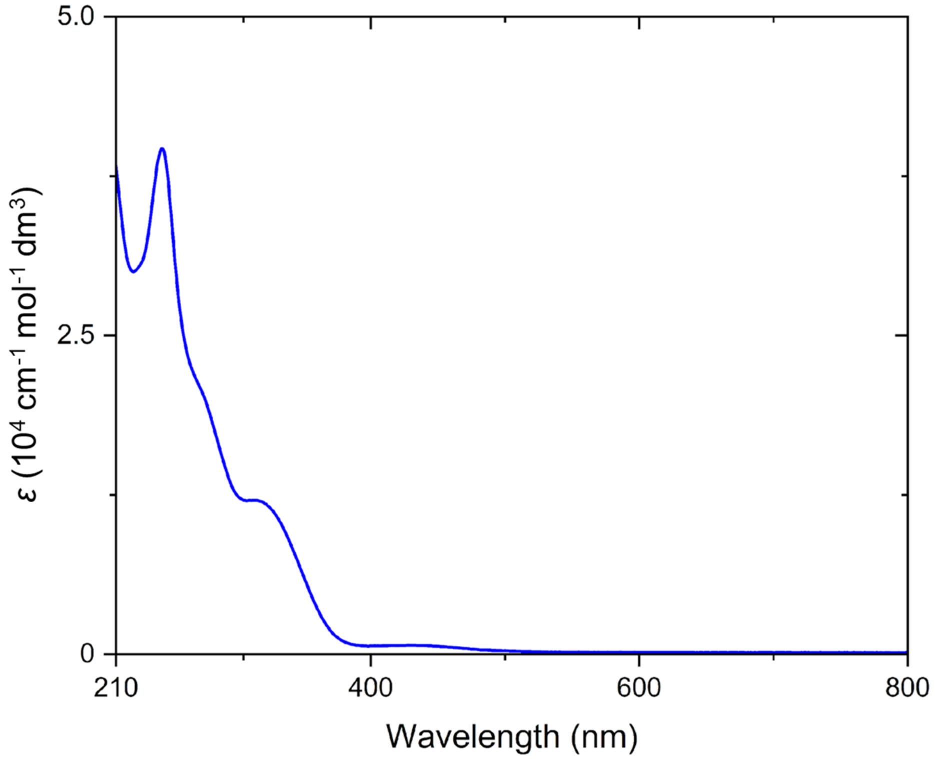

Figure S214. UV-vis absorption spectrum of **2** in hexane at 293 K.

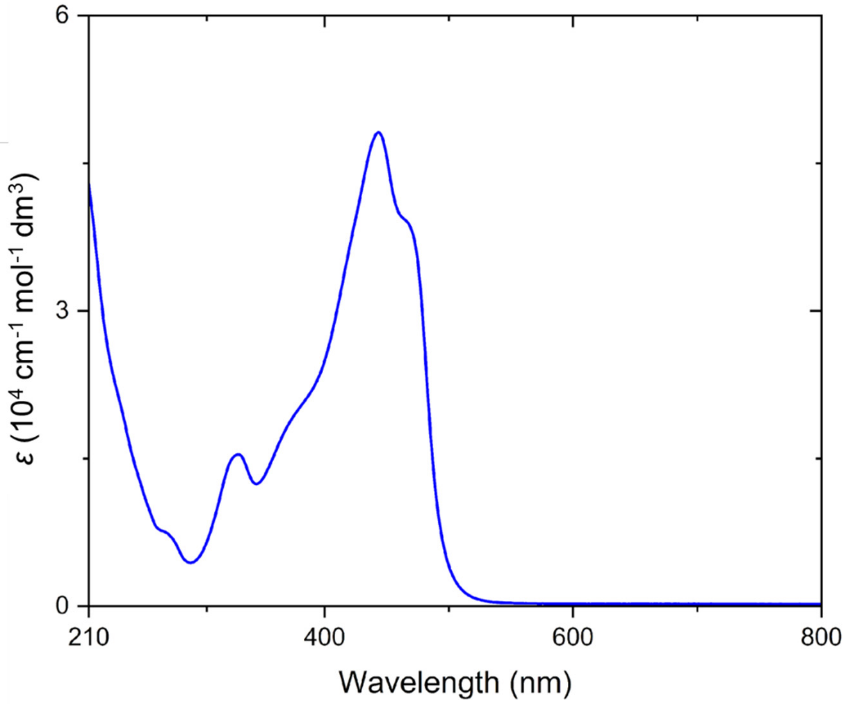

Figure S215. UV-vis absorption spectrum of **4** in THF at 293 K.

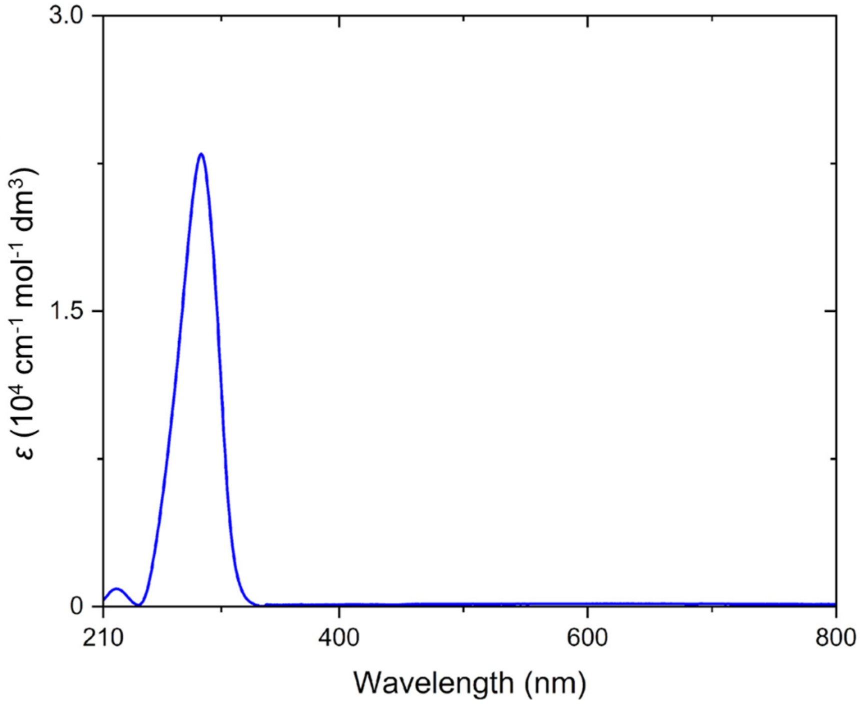

Figure S216. UV-vis absorption spectrum of $\mathbf{7}_{\mathbf{Ad}}$ in hexane at 293 K.

6. FT-IR spectra

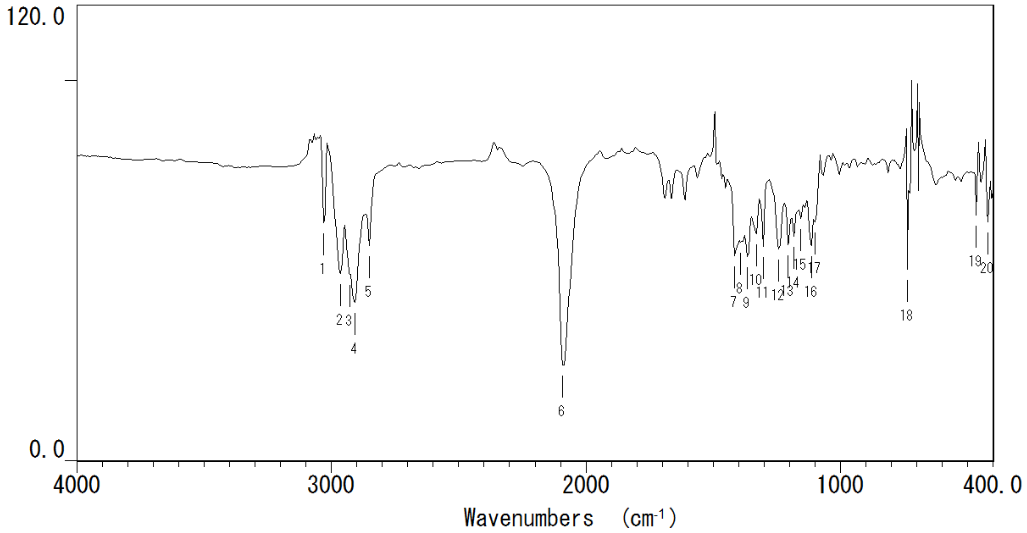

Peak number	Wavenumbers[cm^{-1}]	Transmittance[%]
1	3031.55	62.5700
2	2962.13	49.3029
3	2927.41	49.0305
4	2908.13	41.6622
5	2850.27	56.7422
6	2090.46	25.2478
7	1415.49	53.9814
8	1392.35	57.6448
9	1365.35	53.7481
10	1330.64	59.7321
11	1303.64	56.4327
12	1241.93	55.8745
13	1207.22	56.9503
14	1184.08	59.0582
15	1157.08	63.8282
16	1114.65	56.6806
17	1099.23	62.8251
18	736.674	50.4420
19	466.689	64.5822
20	420.406	63.0010

Figure S217. FT-IR spectrum of $\mathbf{1}_{\mathbf{Ad}}$ in toluene at room temperature.

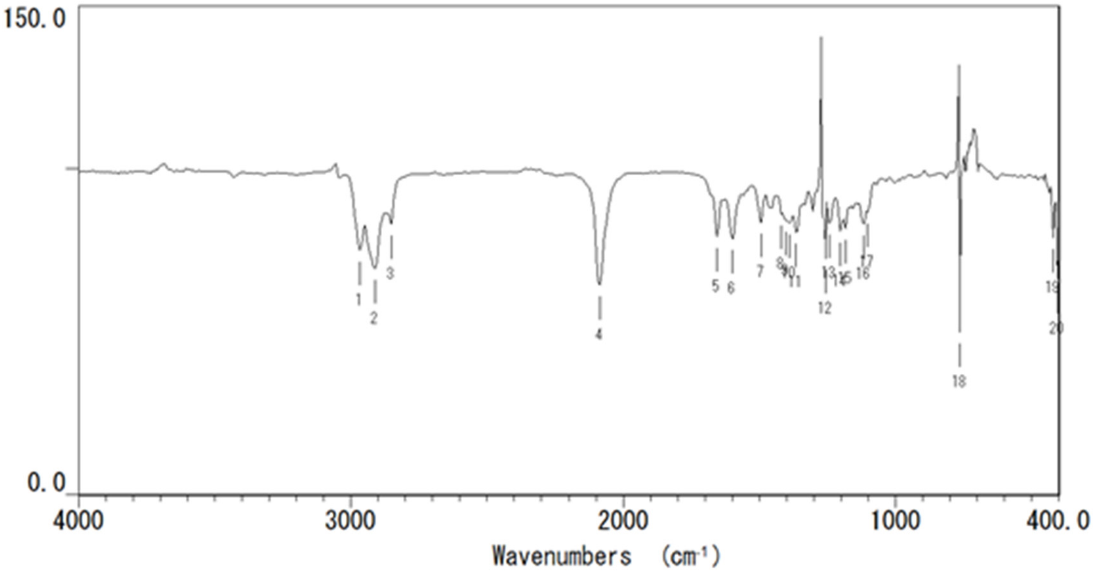

Peak number	Wavenumbers[cm^{-1}]	Transmittance[%]
1	2965.98	75.0432
2	2911.98	69.3177
3	2850.27	83.0774
4	2086.60	64.4887
5	1654.62	79.0408
6	1600.63	78.48
7	1492.63	83.5077
8	1419.35	86.1854
9	1403.92	84.1943
10	1388.50	83.447
11	1365.35	80.6899
12	1257.36	72.6314
13	1241.93	83.277
14	1203.36	80.7153
15	1184.08	81.6744
16	1118.51	82.9718
17	1103.08	86.7938
18	763.673	49.9345
19	420.406	78.9953
20	404.978	66.3834

Figure S218. FT-IR spectrum of $\mathbf{1}_{\mathbf{Ad}}$ in CH_2Cl_2 at room temperature.

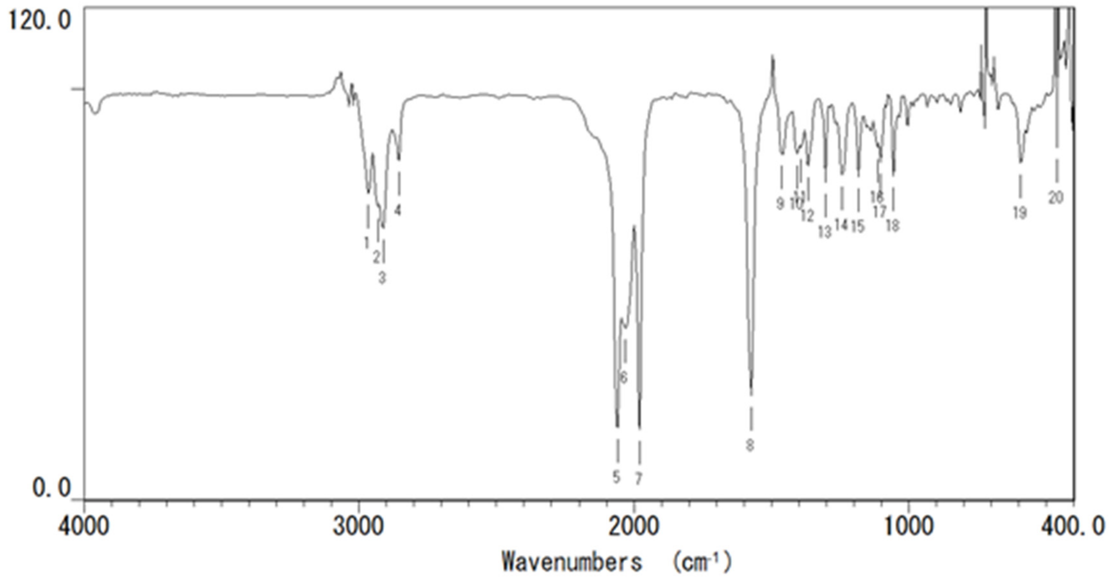

Peak number	Wavenumbers[cm^{-1}]	Transmittance[%]
1	2965.98	74.7039
2	2931.27	71.3461
3	2911.98	66.1453
4	2854.13	82.7349
5	2059.6	17.5884
6	2032.6	41.8633
7	1978.61	17.2412
8	1573.63	25.5761
9	1461.78	84.2084
10	1407.78	84.347
11	1392.35	85.8982
12	1365.35	81.4746
13	1303.64	77.4212
14	1241.93	79.1562
15	1184.08	78.7091
16	1114.65	85.9127
17	1103.08	82.3045
18	1056.8	79.007
19	593.968	82.1436
20	462.832	85.8037

Figure S219. FT-IR spectrum of **6** in toluene at room temperature.

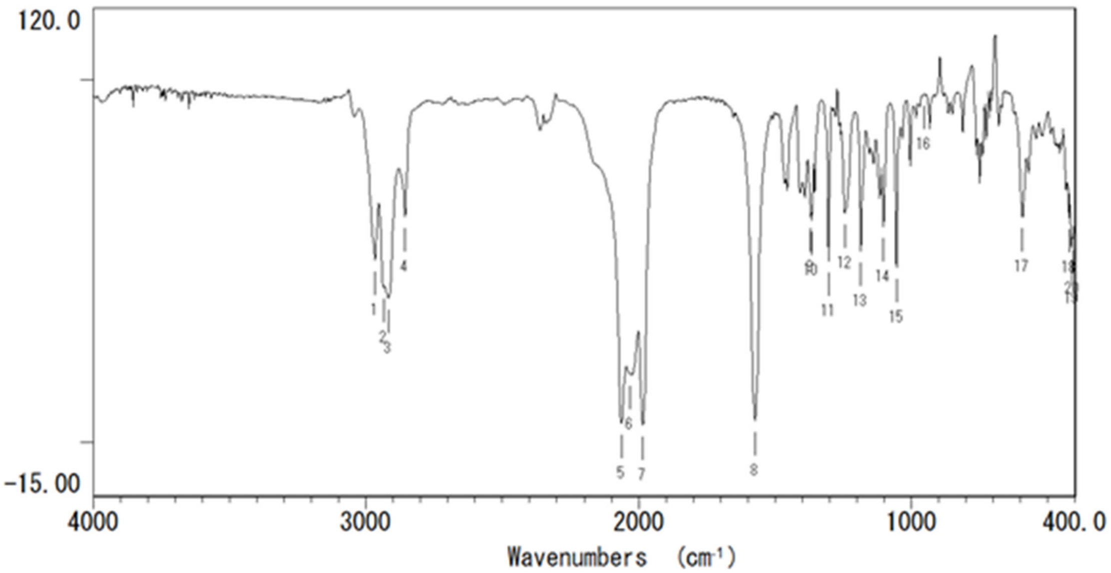

Peak number	Wavenumbers[cm^{-1}]	Transmittance[%]
1	2965.98	50.3109
2	2935.13	42.711
3	2917.77	39.6626
4	2856.06	62.1787
5	2063.46	5.09668
6	2030.68	18.6123
7	1984.39	4.72532
8	1573.63	5.81104
9	1369.21	62.2033
10	1365.35	61.3649
11	1303.64	49.9521
12	1243.86	63.237
13	1186.01	52.6969
14	1103.08	59.5644
15	1054.87	48.275
16	954.591	96.0719
17	593.968	62.2007
18	422.334	62.1039
19	412.692	53.564
20	406.907	56.2906

Figure S220. FT-IR spectrum of **6** in CH_2Cl_2 at room temperature. The TEP value was calculated by correlation of the CO stretching frequency values of **6** (1984, 2063 cm^{-1} [CH_2Cl_2]) using the Rh-to-Ni linear regression equation (TEP [cm^{-1}] = 0.8001 × $\tilde{v}$CO [cm^{-1}] + 420.0 [cm^{-1}], where $\tilde{v}$CO is the average CO stretch-vibration frequency of the LRh(CO)$_2$Cl complex)

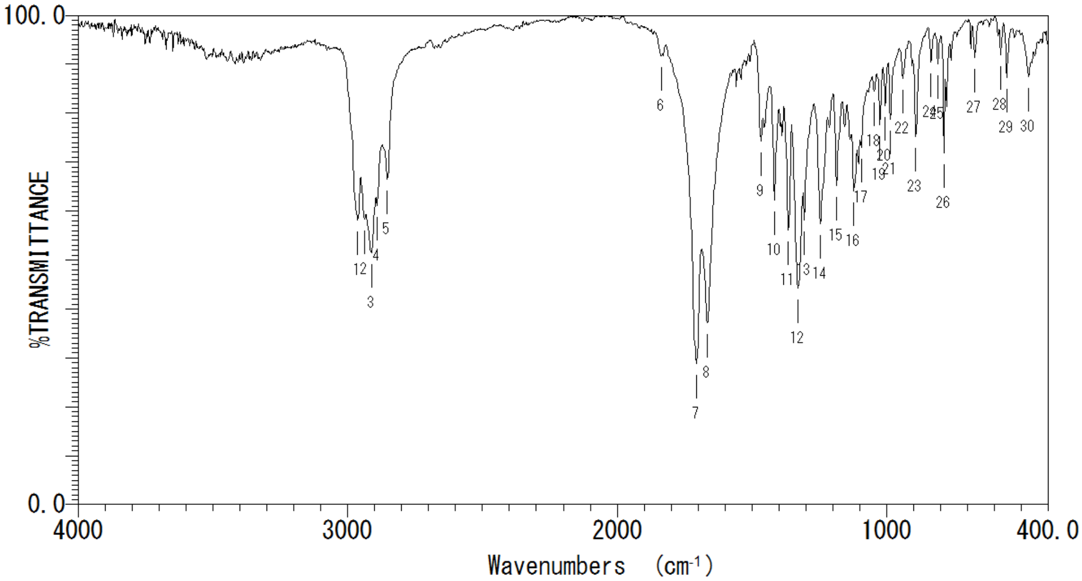

Peak number	Wavenumbers[cm-1]	Transmittance[%]
1	2962.13	58.2313
2	2937.06	58.4347
3	2911.98	51.489
4	2890.77	61.166
5	2852.2	66.5387
6	1835.9	91.7231
7	1706.69	28.8403
8	1666.2	37.2663
9	1465.63	74.3013
10	1417.42	62.1782
11	1365.35	56.1006
12	1328.71	44.2845
13	1305.57	58.1991
14	1245.79	57.4719
15	1186.01	65.1269
16	1122.37	64.0662
17	1095.37	73.0878
18	1047.16	84.4793
19	1025.94	77.615
20	1006.66	81.4547
21	987.375	78.6988

22	939.163	87.0676
23	892.88	75.3181
24	836.955	90.3528
25	811.885	90.1068
26	788.743	71.8263
27	673.035	91.3567
28	578.54	91.9137
29	555.398	87.2589
30	474.403	87.4125

Figure S221. FT-IR spectrum of $\mathbf{7}_{\mathbf{Ad}}$ (KBr matrix) at room temperature.

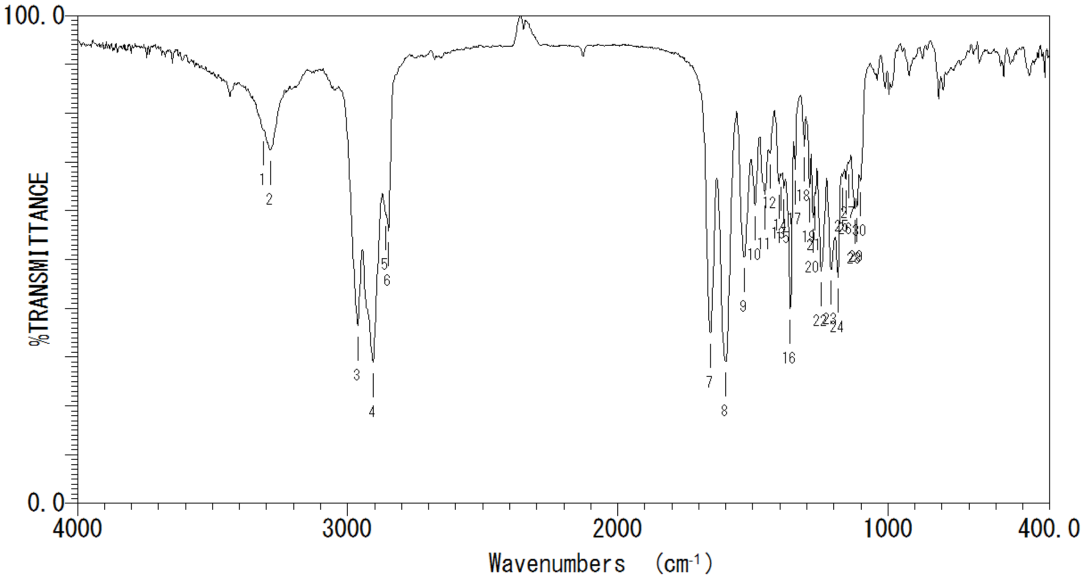

Peak number	Wavenumbers[cm^{-1}]	Transmittance[%]
1	3311.18	76.4473
2	3286.11	72.4646
3	2962.13	36.5163
4	2906.2	28.8965
5	2857.99	59.1772
6	2848.35	55.8425
7	1656.55	34.9341
8	1600.63	29.1382
9	1531.2	50.4818
10	1492.63	61.152
11	1455.99	63.3237
12	1436.71	71.7036
13	1403.92	65.6171
14	1394.28	67.2612
15	1384.64	64.69
16	1361.5	39.9596
17	1342.21	68.5467
18	1309.43	73.1626
19	1290.14	64.8524
20	1276.65	58.5171
21	1268.93	63.2265

22	1247.72	47.6756
23	1211.08	47.9878
24	1186.01	46.3462
25	1168.65	67.0787
26	1155.15	66.4148
27	1145.51	69.7354
28	1122.37	60.3634
29	1114.65	60.8239
30	1101.15	66.0615

Figure S222. FT-IR spectrum of **13** (KBr matrix) at room temperature.

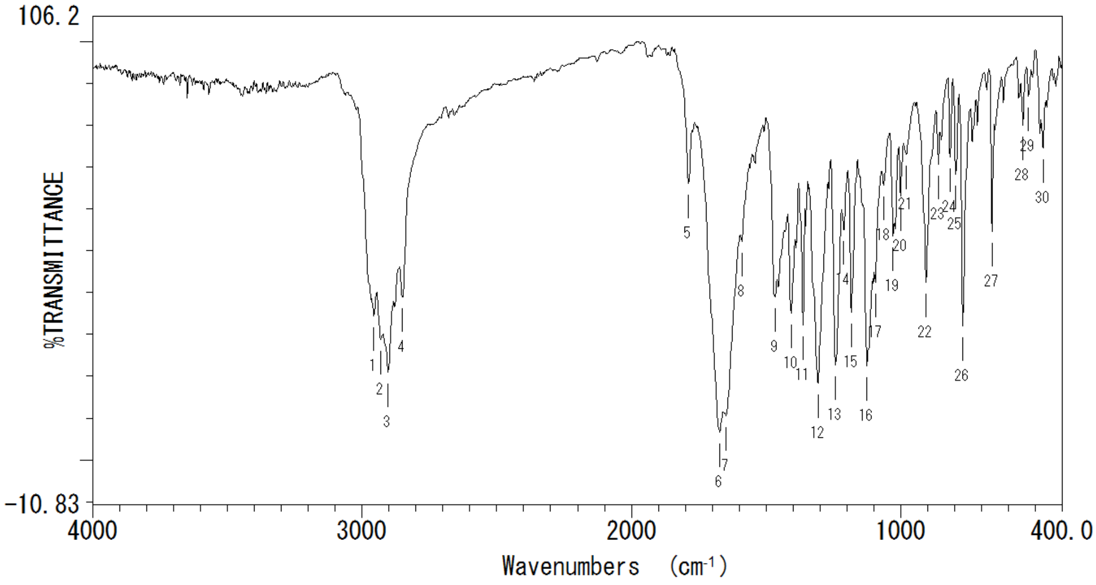

Peak number	Wavenumbers[cm^{-1}]	Transmittance[%]
1	2956.34	34.5113
2	2931.27	28.6869
3	2902.34	21.0289
4	2850.27	38.9965
5	1787.69	66.0885
6	1671.98	6.70649
7	1648.84	10.5973
8	1590.99	52.1628
9	1465.63	39.0238
10	1407.78	35.0072
11	1363.43	32.2858
12	1307.5	18.3003
13	1241.93	22.6752
14	1213.01	55.035
15	1184.08	35.187
16	1126.22	22.4809
17	1095.37	42.4865
18	1064.51	65.6188
19	1029.8	53.5457
20	1000.87	63.089
21	979.661	73.0924

22	906.379	42.4505
23	862.025	70.6468
24	817.67	72.4593
25	796.457	68.3373
26	769.458	32.1274
27	661.464	54.5708
28	547.685	80.0115
29	526.471	86.9959
30	472.474	74.5853

Figure S223. FT-IR spectrum of **8** (KBr matrix) at room temperature.

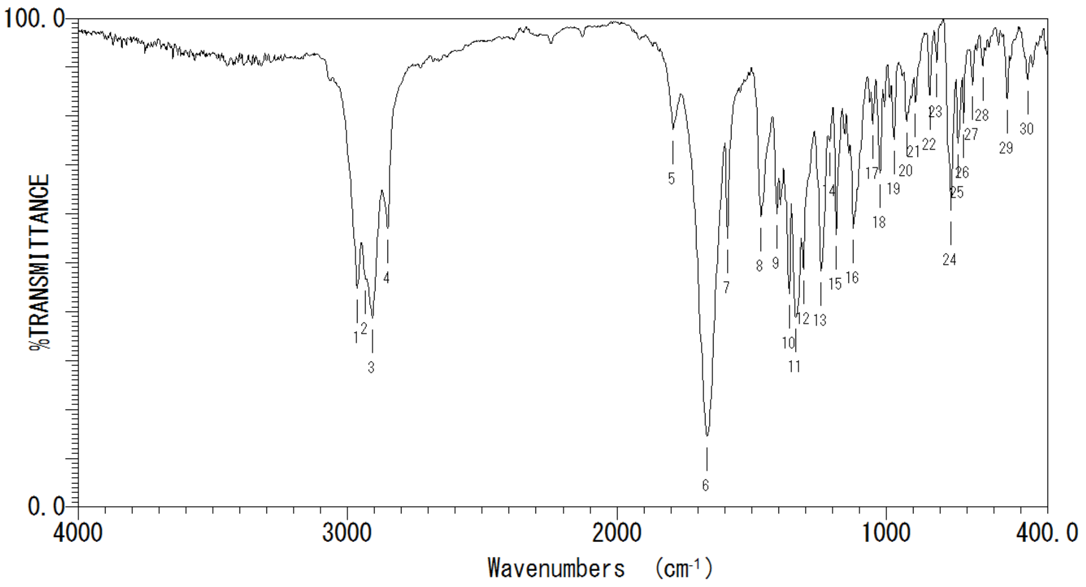

Peak number	Wavenumbers[cm^{-1}]	Transmittance[%]
1	2964.05	44.8266
2	2933.2	46.5997
3	2906.2	38.5999
4	2850.27	57.0902
5	1791.55	77.2223
6	1666.2	14.6248
7	1590.99	55.0452
8	1465.63	59.4683
9	1405.85	59.927
10	1361.5	43.7169
11	1336.43	38.8585
12	1307.5	48.7682
13	1241.93	48.3641
14	1209.15	74.7804
15	1186.01	55.7532
16	1122.37	57.0433
17	1051.01	78.4395
18	1024.02	68.3016
19	971.947	75.3204
20	925.664	78.925
21	892.88	82.7744

22	838.883	84.3613
23	813.813	90.9124
24	759.816	60.6688
25	734.746	74.4392
26	713.533	78.5233
27	680.749	86.4099
28	642.179	90.3211
29	551.542	83.5452
30	476.331	87.4728

Figure S224. FT-IR spectrum of **9** (KBr matrix) at room temperature.

7. Cyclic Voltammetry

Cyclic voltammetry (CV) was performed on an ALS/chi-600A electrochemical analyzer. The CV cell consisted of a glassy carbon electrode, a Pt wire counter electrode, and an Ag/$AgNO_3$ reference electrode. Samples were prepared with 0.10 M Bu_4NPF_6 in dry, degassed acetonitrile in Ar atmosphere. Reduction potentials were measured by scanning potentials in the negative direction and oxidation potential in the positive direction. The glassy carbon electrode was polished between each scan.

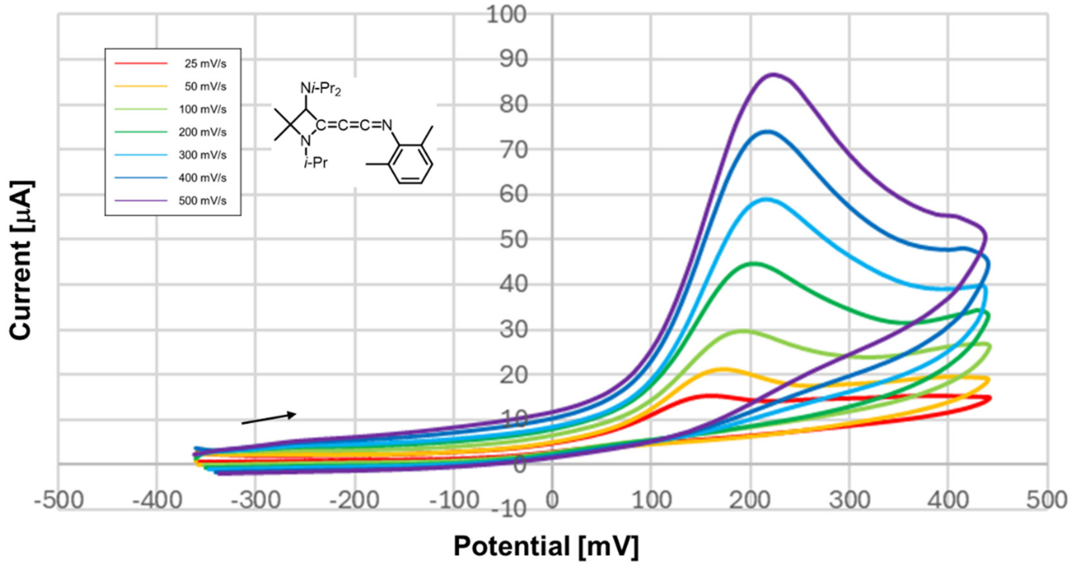

Figure S225. Cyclic voltammogram of methyleneketenimine **1**$_{\mathbf{Xyl}}$ in MeCN vs ferrocene/ferrocenium couple at room temperature containing 0.10 M Bu_4NPF_6, measured at various scan rates (sweep range: –358 ~ 441 mV). The first oxidation potential of **1**$_{\mathbf{Xyl}}$ is 172 mV at a scan rate of 50 mV/s.

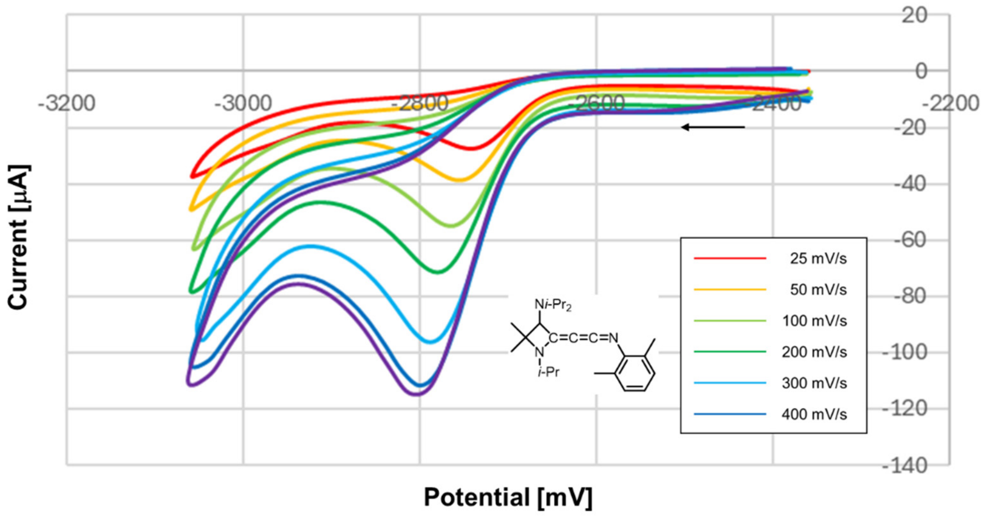

Figure S226. Cyclic voltammogram of methyleneketenimine **1**$_{\mathbf{Xyl}}$ in MeCN vs ferrocene/ferrocenium couple at room temperature containing 0.10 M Bu_4NPF_6, measured at various scan rates (sweep range: –2358 to –3059 mV). The first reduction potential of **1**$_{\mathbf{Xyl}}$ is –2756 mV at a scan rate of 50 mV/s.

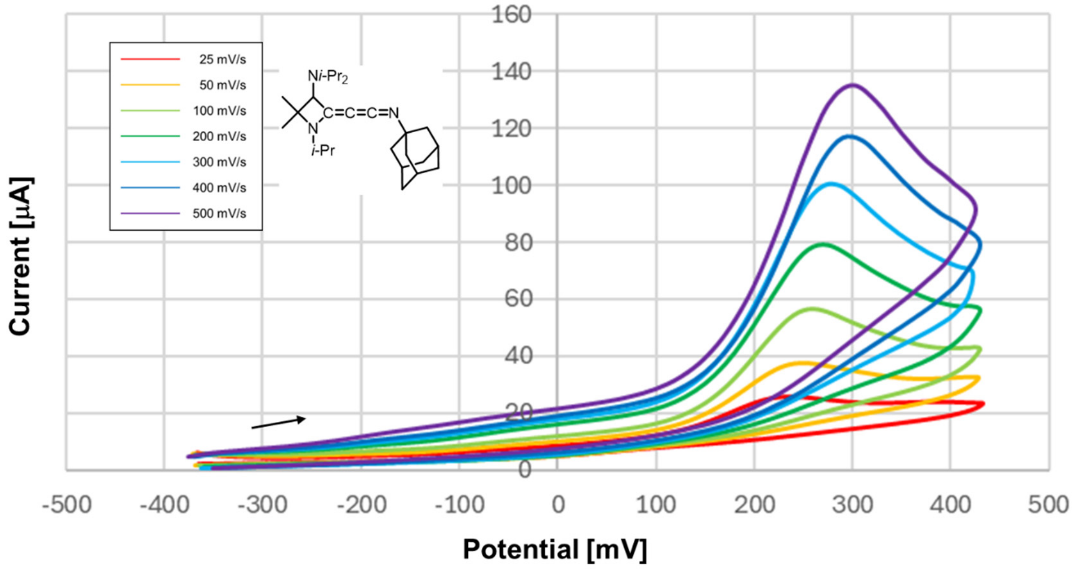

Figure S227. Cyclic voltammogram of methyleneketenimine **1Ad** in MeCN vs ferrocene/ferrocenium couple at room temperature containing 0.10 M Bu_4NPF_6, measured at various scan rates (sweep range: –368 ~ 433 mV). The first oxidation potential of **1Ad** is 250 mV at a scan rate of 50 mV/s.

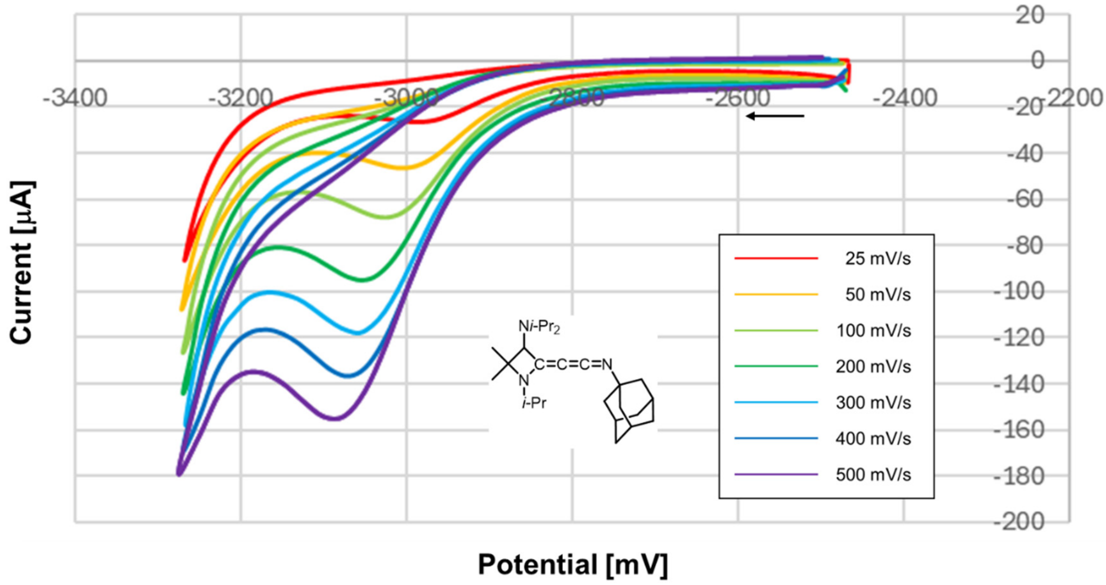

Figure S228. Cyclic voltammogram of methyleneketenimine **1Ad** in MeCN vs ferrocene/ferrocenium couple at room temperature containing 0.10 M Bu_4NPF_6, measured at various scan rates (sweep range: –2468 ~ –3268 mV). The first reduction potential of **1Ad** is –3008 mV at a scan rate of 50 mV/s.

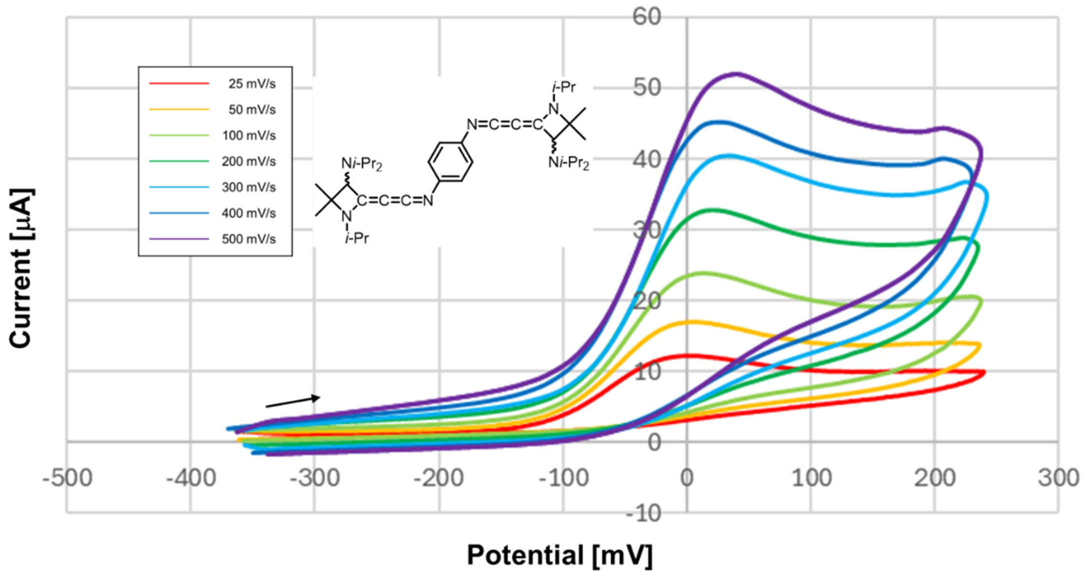

Figure S229. Cyclic voltammogram of methyleneketenimine **4** in MeCN vs ferrocene/ferrocenium couple at room temperature containing 0.10 M Bu_4NPF_6, measured at various scan rates (sweep range: –358 ~ 240 mV). The first oxidation potential of **4** is 3.6 mV at a scan rate of 50 mV/s.

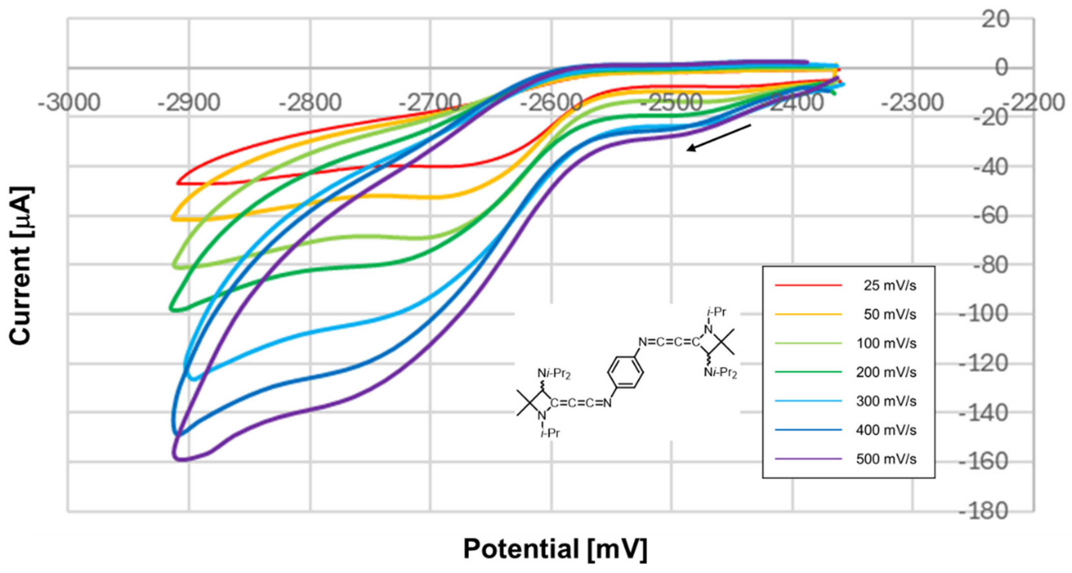

Figure S230. Cyclic voltammogram of methyleneketenimine **4** in MeCN vs ferrocene/ferrocenium couple at room temperature containing 0.10 M Bu_4NPF_6, measured at various scan rates (sweep range: –2358 ~ –2910 mV). The first reduction potential of **4** is –2701 mV at a scan rate of 50 mV/s.

8. Computational Studies

All theoretical calculations were performed using Gaussian 16[78] or ORCA (6.1.0 program).[79-81] Geometry optimization and frequency analysis for all structures were performed at the r^{2}SCAN-3c level of theory. Excitation energies and oscillator strengths of **1**$_{\mathbf{Xyl}}$, **1**$_{\mathbf{Ad}}$ and **4** were calculated at the B3LYP/6-311++G(d,p)//r^{2}SCAN-3c level. Energy levels of the molecular orbitals of all compounds were calculated on the same level. Isotropic chemical shifts of **12** were calculated at the M06-L/def2-TZVP//r^{2}SCAN-3c level and are in accordance with the experimental values. Mayer bond order (MBO) was calculated using the Multifunctional Wavefunction Analyzer (Multiwfn) 3.8 program.[82] Wiberg bond indices (WBI) calculation, Natural Localized Molecular orbital (NLMO) analysis, Natural Population Analysis (NPA) charge calculation, and Natural Resonance Theory (NRT) Analysis were conducted by the NBO 7.0 program.[83] Imaginary frequencies were not found in any of the optimized equilibrium structures. One imaginary frequency is observed for each transition state structure. Reaction routes were investigated using the NEB-TS method in combination with IRC calculations implemented in ORCA 6 at the r^{2}SCAN-3c (SMD = THF) level.[84]

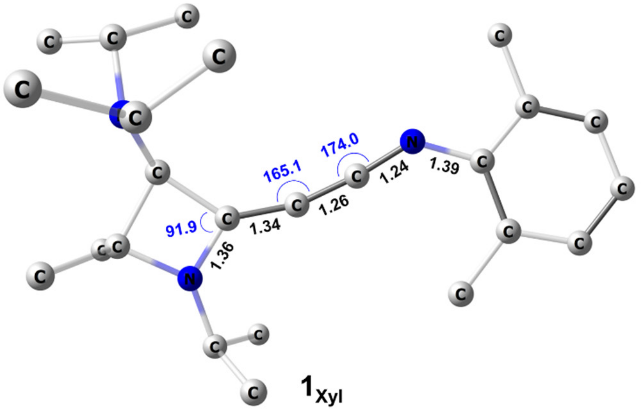

1$_{\mathbf{Xyl}}$

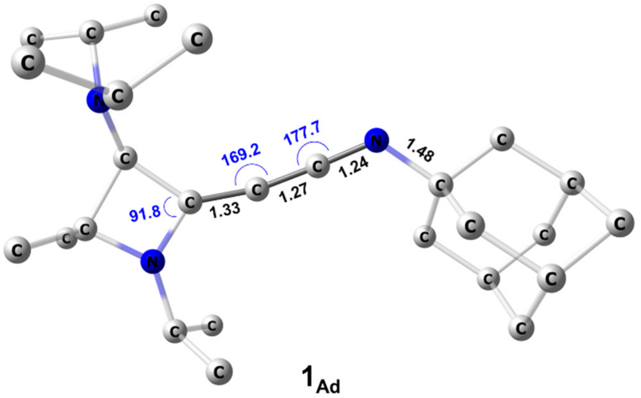

1$_{\mathbf{Ad}}$

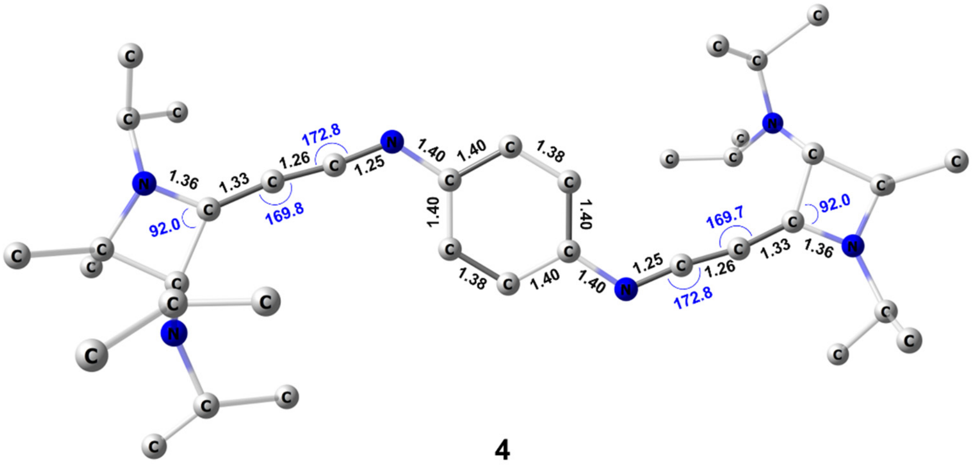

4

Figure S231. Selected structural parameters of methyleneketenimines calculated at the r^{2}SCAN-3c level.

Electronic Properties of the Calculated Methyleneketenimines

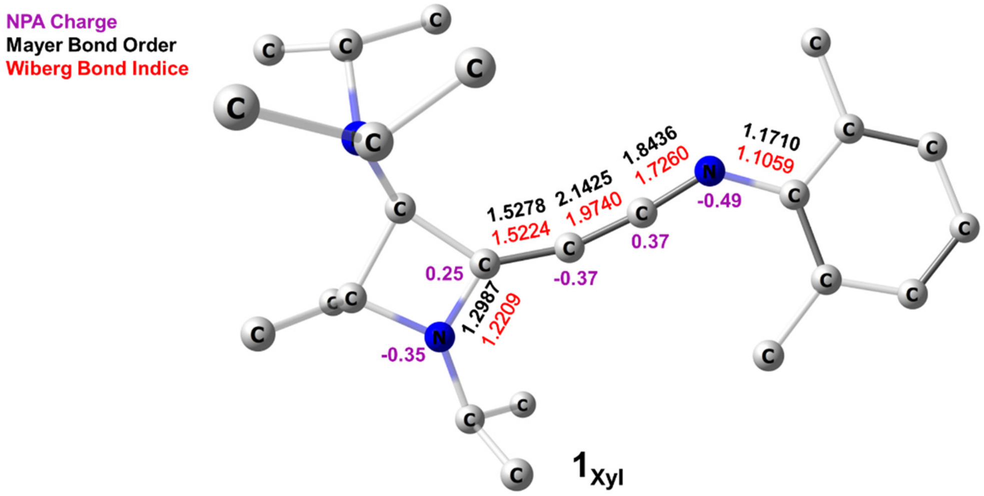

1_{Xyl}

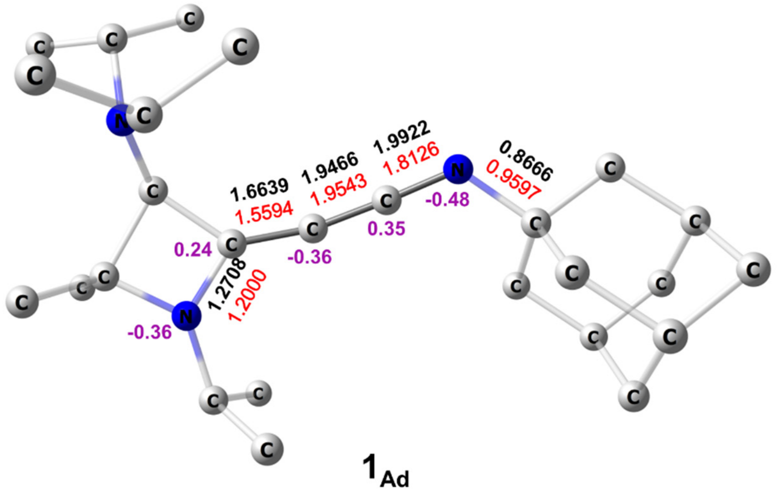

1_{Ad}

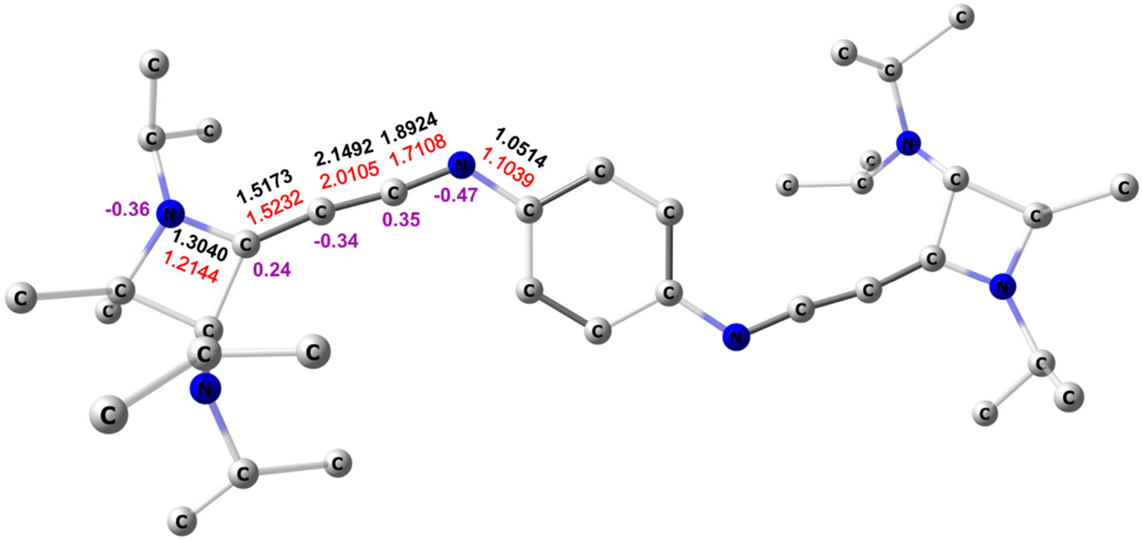

4

Figure S232. Wiberg bond indices, Mayer bond order and NPA charges of the calculated methyleneketenimines.

Kohn-Sham Orbitals

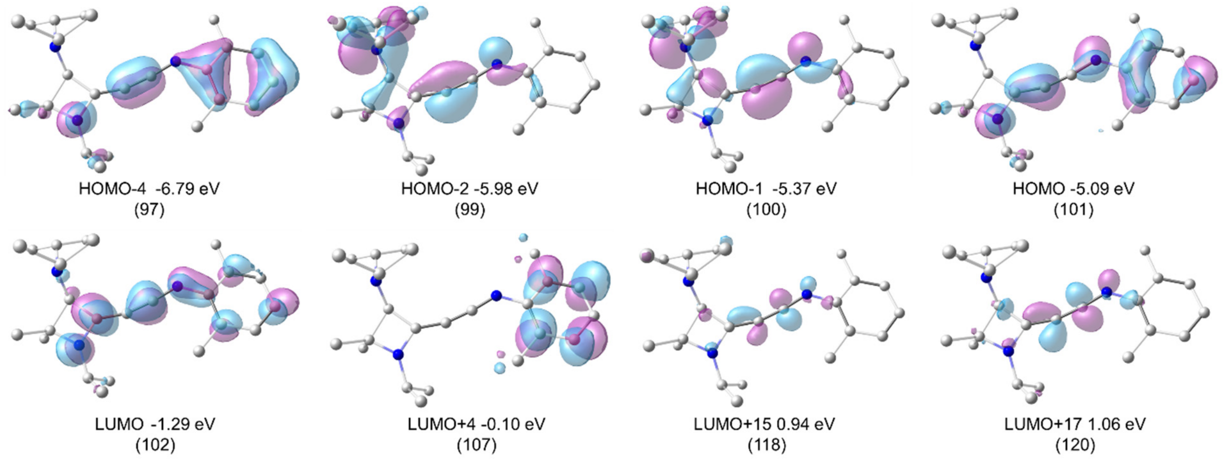

Figure S233. Kohn-Sham orbitals of methyleneketenimine $\mathbf{1}_{\mathbf{Xyl}}$ calculated at the B3LYP/6-311++G(d,p)//r^{2}SCAN-3c level (isosurface: 0.04).

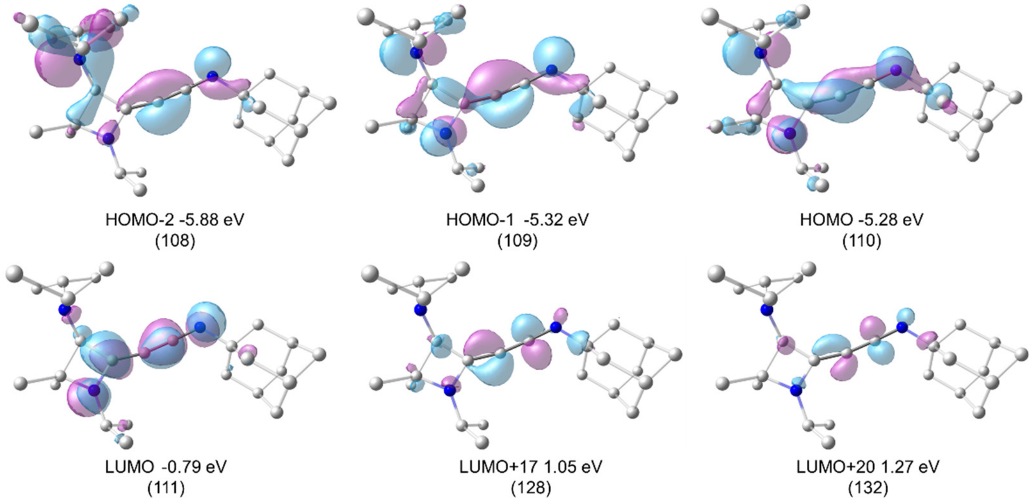

Figure S234. Kohn-Sham orbitals of methyleneketenimine $\mathbf{1}_{\mathbf{Ad}}$ calculated at the B3LYP/6-311++G(d,p)//r^{2}SCAN-3c level (isosurface: 0.04).

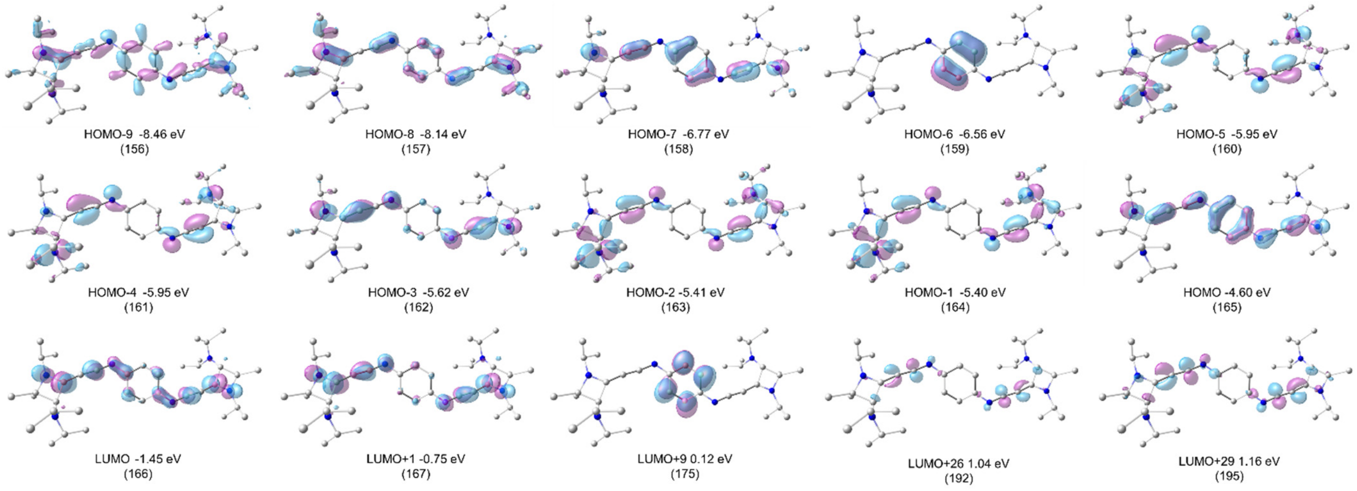

Figure S235. Kohn-Sham orbitals of methyleneketenimine **4** calculated at the B3LYP/6-311++G(d,p)//r^{2}SCAN-3c level (isosurface: 0.04).

NBO and NLMO Analysis

(a) NLMOs

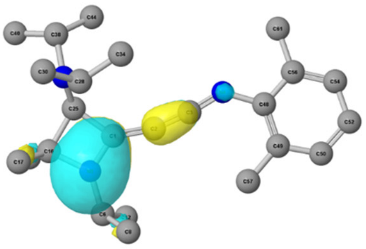

LP1 (N5) occ = 2.0 e-
(N5 80%, C1 10%, C2 3% C3 2%)

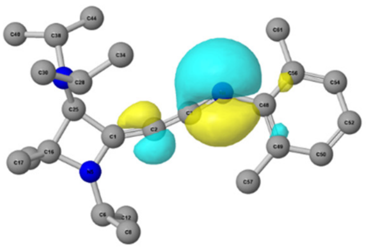

LP1 (N4) occ = 2.0 e-
(N4 87%, C2 4%, C3 7%)

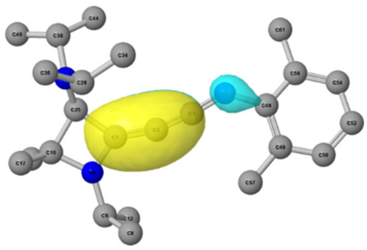

BD2 (C1-C2) 2.0 e-
(C2 53%, C1 33%,
C3 9%, N4 4%)

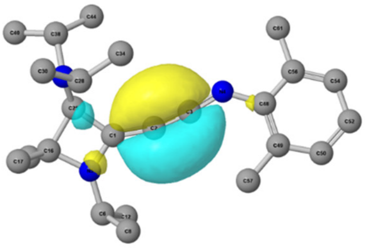

BD2 (C2-C3) 2.0 e-
(C2 57%, C3 39%, C1 2%)

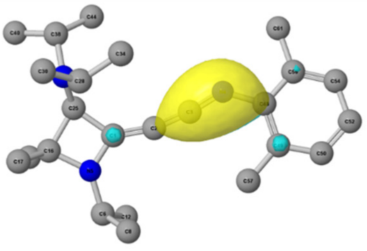

BD2 (C3-N4) 2.0 e-
(N4 62%, C3 32%)

(b) NBOs

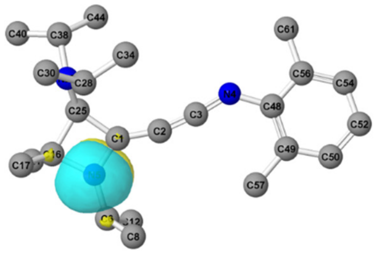

LP1 (N5, p) occ. = 1.61 el.

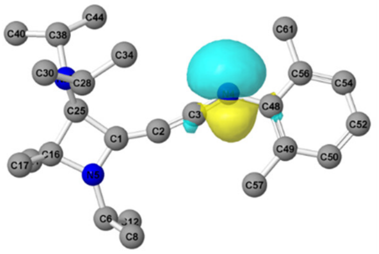

LP1 (N4, sp3.05) occ. = 1.74 el.

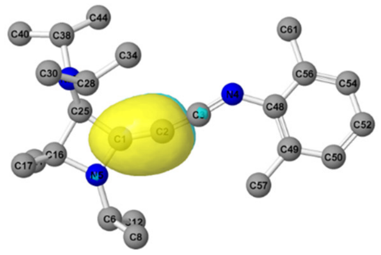

BD2 [C1(p 51%)-C2(p 49%)]
occ. = 1.98 e-

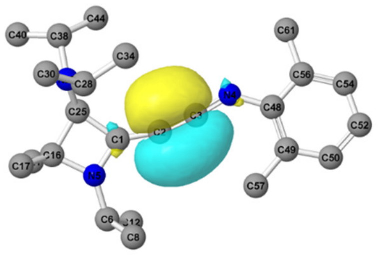

BD2 [C2(p 59%)-C3(p 41%)]
occ. = 1.91 e-

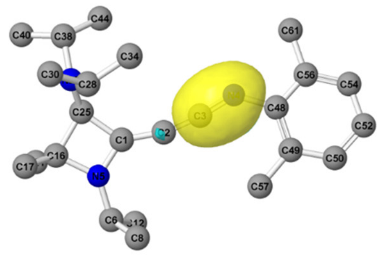

BD2 [C3(p 34%)-N4(p 66%)]
occ. = 1.88 e-

Figure S236. Selected (a) NLMOs and (b) NBOs of methyleneketenimine $\mathbf{1}_{\mathrm{xyl}}$ involving the distribution of the electrons across the N–C–C–C–N skeleton.

(a) NLMOs

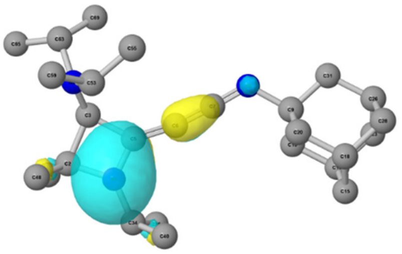

LP1 (N1) occ = 2.0 e-
(N1 81%, C5 9%, C6 3% C7 2%)

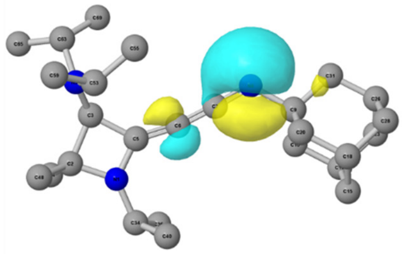

LP1 (N8) occ = 2.0 e-
(N8 89%, C7 5%, C6 3%)

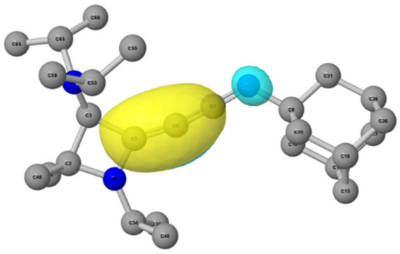

BD2 (C5-C6) 2.0 e-
(C6 53%, C5 35%,
C7 7%, N8 4%)

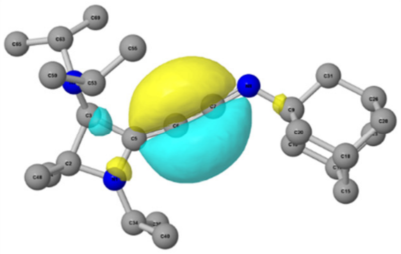

BD2 (C6-C7) 2.0 e-
(C2 55%, C7 40%, C5 2%)

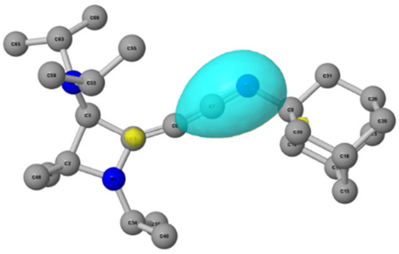

BD2 (C7-N8) 2.0 e-
(N8 62%, C7 35%)

(b) NBOs

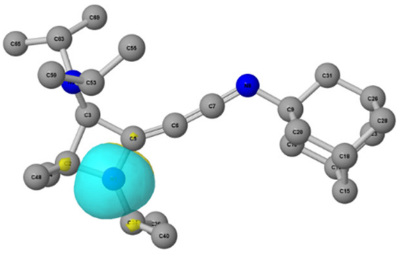

LP1 (N1, sp75.5) occ. = 1.63 el.

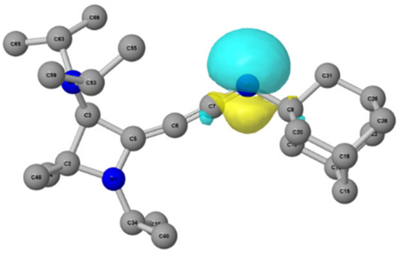

LP1 (N8, sp2.02) occ. = 1.79 el.

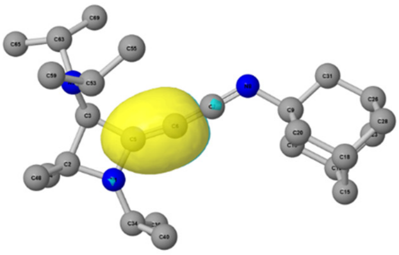

BD2 [C5(p 42%)-C6(p 58%)]
occ. = 1.77 e-

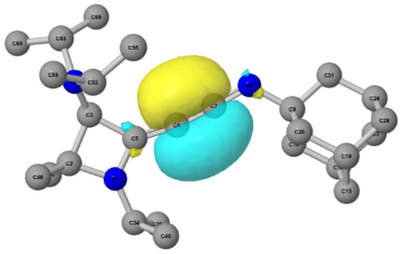

BD2 [C6(p 58%)-C7(p 42%)]
occ. = 1.91 e-

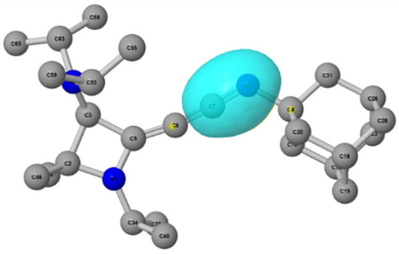

BD2 [C7(p 36%)-N8(p 64%)]
occ. = 1.93 e-

Figure S237. Selected (a) NLMOs and (b) NBOs of methyleneketenimine $\mathbf{1}_{\mathbf{Ad}}$ involving the distribution of the electrons across the N–C–C–C–N skeleton.

(a) NLMOs

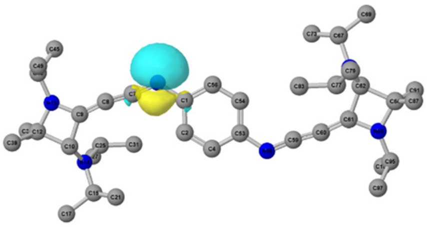

LP1 (N6, sp2.43) occ. = 1.78 el.

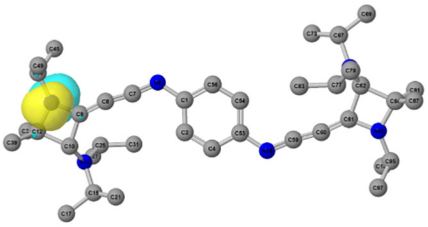

LP1 (N13, p) occ. = 1.62 el.

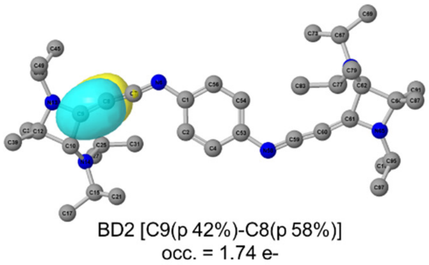

BD2 [C9(p 42%)-C8(p 58%)]
occ. = 1.74 e-

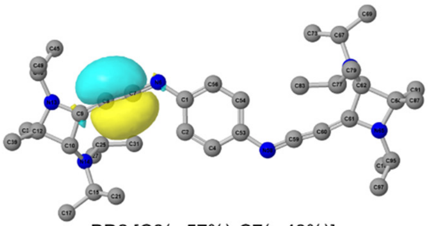

BD2 [C8(p 57%)-C7(p 43%)]
occ. = 1.92 e-

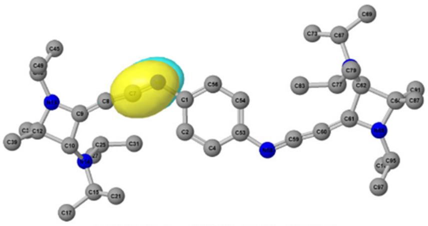

BD2 [C7(p 35%)-N6(p 65%)]
occ. = 1.88 e-

(b) NBOs

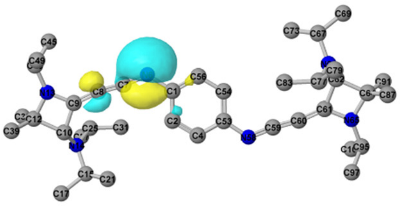

LP1 (N6) occ = 2.0 e-
(N6 89%, C7 5%, C8 3%)

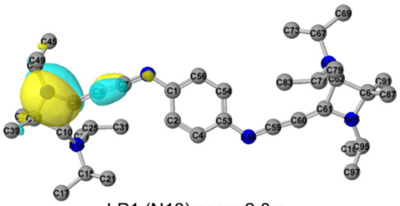

LP1 (N13) occ = 2.0 e-
(N13 80%, C9 10%, C8 3% C7 2%)

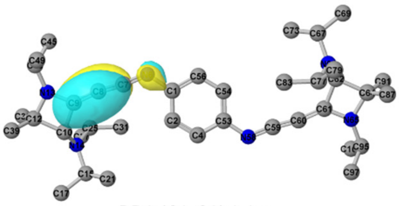

BD2 (C9-C8) 2.0 e-
(C8 53%, C9 33%, C7 8%, N6 4%)

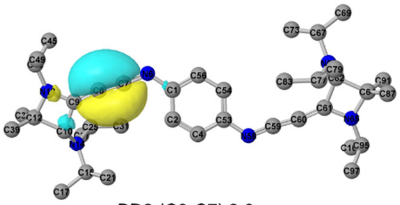

BD2 (C8-C7) 2.0 e-
(C8 55%, C7 40%, C9 2%)

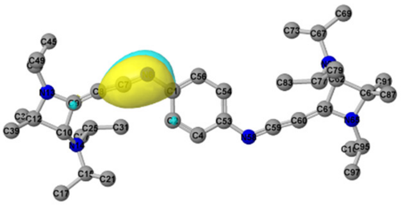

BD2 (C7-N6) 2.0 e-
(N6 61%, C7 33%, C1 2%)

Figure S238. Selected (a) NLMOs and (b) NBOs of methyleneketenimine **4** involving the distribution of the electrons across the N–C–C–C–N skeleton.

NRT Calculations of Selected Methyleneketenimines

1_{Xyl}

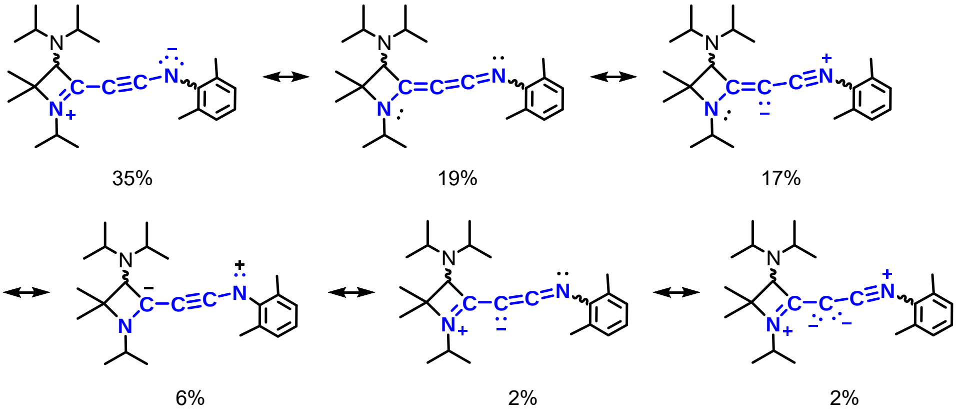

1_{Ad}

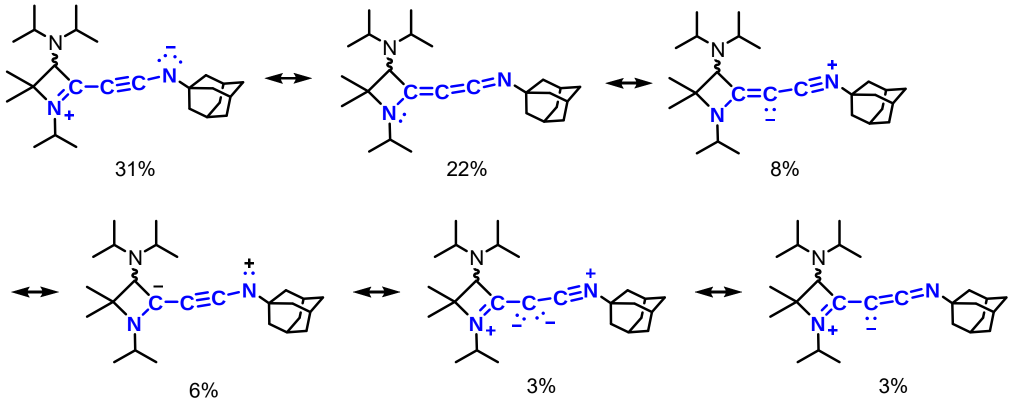

Figure S239. Major canonical structures of calculated methyleneketeninines obtained by NRT analysis.

GIAO Calculation of Cyclobutene 12

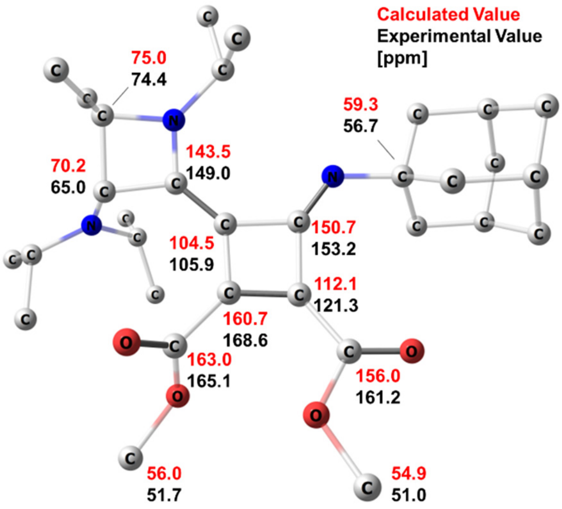

Figure S240. Comparison of experimental and calculated (M06-L/def2-TZVP//r^{2}SCAN-3c level) ^{13}C NMR chemical shifts of cyclobutene **11**.

TD-DFT Calculations

Table S1. Calculated electronic transition of $\mathbf{1}_{\mathbf{Xyl}}$ (tk43e12a). The 101th orbital is the HOMO.

```
xcited State   1:      Singlet-A      3.0069 eV  412.34 nm  f=0.0040  <S**2>=0.000
     99 ->102        0.20131
    100 ->102        0.66059
    101 ->102       -0.13110

 Excited State   2:      Singlet-A      3.5484 eV  349.41 nm  f=0.6789  <S**2>=0.000
     99 ->102       -0.13433
    100 ->102        0.17340
    101 ->102        0.65741

 Excited State   3:      Singlet-A      3.8395 eV  322.92 nm  f=0.0604  <S**2>=0.000
     99 ->102        0.65876
    100 ->102       -0.16785
    101 ->102        0.16966

 Excited State   4:      Singlet-A      4.0935 eV  302.88 nm  f=0.0013  <S**2>=0.000
    101 ->103        0.69649

 Excited State   5:      Singlet-A      4.3001 eV  288.33 nm  f=0.0002  <S**2>=0.000
     98 ->102        0.56735
    101 ->107       -0.38044

 Excited State   6:      Singlet-A      4.3191 eV  287.06 nm  f=0.0011  <S**2>=0.000
    100 ->103        0.69424

 Excited State   7:      Singlet-A      4.4781 eV  276.87 nm  f=0.0004  <S**2>=0.000
    101 ->104        0.49093
    101 ->105       -0.31586
    101 ->106        0.35751

 Excited State   8:      Singlet-A      4.4904 eV  276.11 nm  f=0.0024  <S**2>=0.000
    101 ->104        0.44070
    101 ->105        0.51611
    101 ->106       -0.14129

 Excited State   9:      Singlet-A      4.5381 eV  273.21 nm  f=0.0030  <S**2>=0.000
    101 ->104       -0.20875
    101 ->105        0.32850
    101 ->106        0.56517

 Excited State  10:      Singlet-A      4.6544 eV  266.38 nm  f=0.0012  <S**2>=0.000
     99 ->103       -0.10184
    100 ->104        0.67291
    100 ->105       -0.10752

 Excited State  11:      Singlet-A      4.6972 eV  263.95 nm  f=0.0012  <S**2>=0.000
    100 ->105        0.67047
    100 ->106        0.14989

 Excited State  12:      Singlet-A      4.7257 eV  262.36 nm  f=0.0110  <S**2>=0.000
     97 ->102       -0.11328
     98 ->102       -0.17990
    101 ->107       -0.35190
    101 ->108        0.44110
    101 ->109        0.20777
    101 ->112       -0.16437
    101 ->118       -0.11686
    101 ->120        0.11397

 Excited State  13:      Singlet-A      4.7779 eV  259.50 nm  f=0.0055  <S**2>=0.000
     99 ->103        0.21172
    100 ->105       -0.13269
    100 ->106        0.61476
    101 ->108        0.11000

 Excited State  14:      Singlet-A      4.7958 eV  258.52 nm  f=0.0524  <S**2>=0.000
     98 ->102        0.31057
    100 ->106       -0.12688
    101 ->107        0.40956
    101 ->108        0.36035
    101 ->109        0.19997

 Excited State  15:      Singlet-A      4.8546 eV  255.40 nm  f=0.0108  <S**2>=0.000
     97 ->102        0.18797
     99 ->107        0.14234
    100 ->107        0.62397
    101 ->108        0.11051

 Excited State  16:      Singlet-A      4.8717 eV  254.50 nm  f=0.0017  <S**2>=0.000
     97 ->102       -0.27262
    100 ->107        0.19044
    101 ->107        0.10655
    101 ->108       -0.27075
    101 ->109        0.48833
    101 ->112       -0.11825

 Excited State  17:      Singlet-A      4.8847 eV  253.82 nm  f=0.0016  <S**2>=0.000
    101 ->110        0.67720

 Excited State  18:      Singlet-A      4.9094 eV  252.54 nm  f=0.0192  <S**2>=0.000
     97 ->102        0.38830
     98 ->102       -0.13300
    100 ->107       -0.12644
    101 ->107       -0.13377
    101 ->109        0.36896
    101 ->111       -0.14503
    101 ->112        0.26416
    101 ->114       -0.12407

 Excited State  19:      Singlet-A      4.9559 eV  250.18 nm  f=0.0009  <S**2>=0.000
     99 ->103        0.65018
    100 ->104        0.11843
    100 ->106       -0.19829

 Excited State  20:      Singlet-A      4.9814 eV  248.89 nm  f=0.0007  <S**2>=0.000
     97 ->102       -0.10270
    101 ->108        0.17690
    101 ->109        0.10766
    101 ->111        0.44687
    101 ->112        0.28636
    101 ->113       -0.13998
    101 ->115       -0.12038
    101 ->116        0.10090
    101 ->118        0.17217
    101 ->120       -0.20114
    101 ->123        0.10647

 Excited State  21:      Singlet-A      5.0272 eV  246.63 nm  f=0.0638  <S**2>=0.000
     97 ->102        0.21459
    100 ->109       -0.14054
    100 ->112        0.17498
    101 ->111        0.45253
    101 ->112       -0.25107
    101 ->113        0.11179
    101 ->118       -0.11427
    101 ->120        0.11734

 Excited State  22:      Singlet-A      5.0638 eV  244.85 nm  f=0.0125  <S**2>=0.000
    100 ->109        0.47755
    100 ->112       -0.26909
    100 ->113       -0.13650
    100 ->114        0.11950
    101 ->111        0.17255
    101 ->113        0.14201
    101 ->114       -0.13038

 Excited State  23:      Singlet-A      5.0808 eV  244.03 nm  f=0.0363  <S**2>=0.000
     99 ->104       -0.16752
    100 ->108        0.41160
    100 ->109       -0.25287
    100 ->110       -0.18775
    100 ->111        0.26284
    100 ->112       -0.17916
    101 ->113        0.11653

 Excited State  24:      Singlet-A      5.1321 eV  241.58 nm  f=0.0311  <S**2>=0.000
     99 ->105        0.15188
    100 ->109       -0.22554
    100 ->110        0.48637
    100 ->111        0.23559
    100 ->112       -0.13687
    100 ->113       -0.12310
    100 ->114        0.14852
    101 ->112       -0.11092
    101 ->113       -0.13748

 Excited State  25:      Singlet-A      5.1465 eV  240.91 nm  f=0.0051  <S**2>=0.000
     99 ->106       -0.11933
    100 ->106       -0.10146
    100 ->108        0.46448
```

100 ->109 0.19248
100 ->110 0.32796
100 ->111 -0.16359
100 ->112 0.16209
100 ->114 -0.13282

Excited State 26: Singlet-A 5.1697 eV 239.83 nm f=0.0010 <S**2>=0.000
100 ->110 0.10266
101 ->113 0.51876
101 ->114 0.43128

Excited State 27: Singlet-A 5.1827 eV 239.22 nm f=0.0059 <S**2>=0.000
100 ->108 -0.14948
100 ->109 -0.15261
100 ->110 0.11450
100 ->111 -0.10305
101 ->112 0.36673
101 ->115 0.44266
101 ->118 -0.14333
101 ->122 -0.10936

Excited State 28: Singlet-A 5.2311 eV 237.01 nm f=0.0177 <S**2>=0.000
99 ->104 -0.14181
99 ->105 0.13827
100 ->109 0.19084
100 ->111 0.45775
100 ->112 0.28208

100 ->114 -0.11320
101 ->113 -0.10008
101 ->114 0.19875
101 ->115 0.15281

Excited State 29: Singlet-A 5.2814 eV 234.76 nm f=0.0101 <S**2>=0.000
97 ->102 0.13102
100 ->110 -0.12210
100 ->111 -0.15746
100 ->113 -0.16573
101 ->113 -0.27669
101 ->114 0.36533
101 ->115 -0.18426
101 ->116 -0.10597
101 ->117 0.16319
101 ->118 -0.16000
101 ->120 0.20210
101 ->123 -0.10086

Excited State 30: Singlet-A 5.2938 eV 234.20 nm f=0.0054 <S**2>=0.000
99 ->104 0.60983
99 ->105 -0.13588
100 ->108 0.13226
100 ->110 -0.13429
100 ->111 0.18173

Table S2. Calculated electronic transition of $\mathbf{1_{Ad}}$ (tk44e1g). The 110th orbital is the HOMO.

Excited State 1: Singlet-A 3.3967 eV 365.01 nm f=0.0015 <S**2>=0.000
108 ->111 0.20016
109 ->111 0.54770
110 ->111 0.39105

Excited State 2: Singlet-A 4.1194 eV 300.98 nm f=0.0985 <S**2>=0.000
108 ->111 0.55459
110 ->111 -0.41011

Excited State 3: Singlet-A 4.2604 eV 291.01 nm f=0.0957 <S**2>=0.000
108 ->111 -0.22092
109 ->111 0.25458
110 ->111 -0.23363
110 ->112 0.54747

Excited State 4: Singlet-A 4.2710 eV 290.29 nm f=0.1180 <S**2>=0.000
108 ->111 0.28774
109 ->111 -0.30255
109 ->112 -0.26951
110 ->111 0.27418
110 ->112 0.38091

Excited State 5: Singlet-A 4.3236 eV 286.76 nm f=0.0091 <S**2>=0.000
108 ->111 0.10821
109 ->112 0.63791
110 ->112 0.20620

Excited State 6: Singlet-A 4.5432 eV 272.90 nm f=0.0008 <S**2>=0.000
109 ->113 -0.21361
110 ->113 0.64733

Excited State 7: Singlet-A 4.5924 eV 269.98 nm f=0.0224 <S**2>=0.000
108 ->112 0.13681
109 ->113 0.60284
110 ->113 0.20662
110 ->114 -0.21009

Excited State 8: Singlet-A 4.6426 eV 267.05 nm f=0.0018 <S**2>=0.000
109 ->113 0.20791
109 ->114 0.29575
110 ->114 0.56229
110 ->115 -0.14478

Excited State 9: Singlet-A 4.7067 eV 263.42 nm f=0.0037 <S**2>=0.000
109 ->113 -0.11014
109 ->114 0.50244
110 ->114 -0.31500
110 ->115 -0.32071

Excited State 10: Singlet-A 4.7265 eV 262.32 nm f=0.0141 <S**2>=0.000
109 ->114 0.33847
109 ->115 0.24248
110 ->115 0.52924
110 ->116 -0.10417

Excited State 11: Singlet-A 4.7642 eV 260.24 nm f=0.0033 <S**2>=0.000
109 ->115 0.63801
110 ->115 -0.23799

Excited State 12: Singlet-A 4.9045 eV 252.80 nm f=0.0036 <S**2>=0.000
108 ->112 0.61004
109 ->113 -0.11189
109 ->116 -0.10065
110 ->116 -0.24878

Excited State 13: Singlet-A 4.9339 eV 251.29 nm f=0.0009 <S**2>=0.000
108 ->112 0.18856
109 ->116 -0.25547
110 ->116 0.53822
110 ->119 -0.13583
110 ->122 0.11901
110 ->128 -0.10234

Excited State 14: Singlet-A 4.9961 eV 248.16 nm f=0.0070 <S**2>=0.000
108 ->112 0.22481
109 ->116 0.50510
110 ->116 0.24580
110 ->117 -0.18571
110 ->118 -0.16337

Excited State 15: Singlet-A 5.0155 eV 247.20 nm f=0.0075 <S**2>=0.000
108 ->114 0.12783
109 ->116 0.21321
110 ->117 0.56952
110 ->118 0.14123
110 ->119 0.17711
110 ->120 -0.11115

Excited State 16: Singlet-A 5.0496 eV 245.53 nm f=0.0003 <S**2>=0.000
109 ->116 -0.11313
109 ->117 0.47977
109 ->118 0.20241
110 ->116 0.11386
110 ->118 -0.23028
110 ->119 0.18528
110 ->128 0.10730

Excited State 17: Singlet-A 5.0779 eV 244.17 nm f=0.0142 <S**2>=0.000
109 ->116 0.25669
109 ->117 0.38021
109 ->119 0.19364
110 ->117 -0.13922
110 ->118 0.30290
110 ->119 -0.28415

Excited State 18: Singlet-A 5.1107 eV 242.60 nm f=0.0035 <S**2>=0.000

108 ->115 0.17205
109 ->118 0.17599
110 ->117 -0.18882
110 ->118 0.46694
110 ->119 0.37828

Excited State 19: Singlet-A 5.1300 eV 241.68 nm f=0.0281 <S**2>=0.000
108 ->113 -0.10258
109 ->118 0.51325
109 ->119 -0.22461
110 ->117 0.11166
110 ->119 -0.31651

Excited State 20: Singlet-A 5.1558 eV 240.48 nm f=0.0149 <S**2>=0.000
108 ->115 0.11061
109 ->117 -0.18310
109 ->118 0.27687
109 ->119 0.55767
110 ->118 -0.13326

Excited State 21: Singlet-A 5.2108 eV 237.94 nm f=0.0082 <S**2>=0.000
108 ->113 0.62293
108 ->115 -0.11315
109 ->118 0.14641
109 ->119 -0.10984
110 ->120 -0.10936

Excited State 22: Singlet-A 5.2313 eV 237.01 nm f=0.0025 <S**2>=0.000
108 ->113 0.16118
108 ->114 -0.18245
110 ->117 0.20112
110 ->118 0.11521
110 ->119 -0.11711
110 ->120 0.47780
110 ->122 -0.11286
110 ->128 0.13810

Excited State 23: Singlet-A 5.2379 eV 236.71 nm f=0.0003 <S**2>=0.000
109 ->117 0.11130
109 ->118 0.10959
109 ->119 -0.16429
109 ->128 0.10928
110 ->118 -0.14417
110 ->119 0.21646
110 ->120 0.42161
110 ->122 0.17413
110 ->124 0.11400
110 ->126 0.10917
110 ->127 0.11348
110 ->128 -0.14015
110 ->132 -0.11027

Excited State 24: Singlet-A 5.2605 eV 235.69 nm f=0.0017 <S**2>=0.000
108 ->114 0.59375
109 ->117 -0.14635
109 ->120 -0.19433
110 ->120 0.18852

Excited State 25: Singlet-A 5.2792 eV 234.85 nm f=0.0025 <S**2>=0.000
108 ->114 0.19071
108 ->115 -0.10486
109 ->119 0.10487
109 ->120 0.63730

Excited State 26: Singlet-A 5.3380 eV 232.27 nm f=0.0025 <S**2>=0.000
108 ->113 0.12156
108 ->115 0.62710
109 ->118 -0.14373
110 ->118 -0.11276

Excited State 27: Singlet-A 5.3963 eV 229.76 nm f=0.0049 <S**2>=0.000
110 ->121 0.62583
110 ->122 0.22827

Excited State 28: Singlet-A 5.4334 eV 228.19 nm f=0.0010 <S**2>=0.000
109 ->121 0.61748
109 ->122 0.23682

Excited State 29: Singlet-A 5.4706 eV 226.64 nm f=0.0445 <S**2>=0.000
108 ->116 -0.14217
109 ->121 -0.20195
109 ->122 0.34536
110 ->121 -0.20380
110 ->122 0.46302

Excited State 30: Singlet-A 5.4946 eV 225.65 nm f=0.0045 <S**2>=0.000
109 ->122 0.15487
109 ->123 0.12323
110 ->123 0.60727
110 ->124 0.10291
110 ->125 0.11687

Table S3. Calculated electronic transition of 4 (tk45e8f). The 165th orbital is the HOMO.

Excited State 1: Singlet-A 2.8767 eV 431.00 nm f=1.4446
<S**2>=0.000
165 -> 166 0.68944

Excited State 2: Singlet-A 3.1209 eV 397.28 nm f=0.0000 <S**2>=0.000
160 -> 167 -0.10599
161 -> 166 -0.20631
163 -> 167 -0.24215
164 -> 166 0.60527

Excited State 3: Singlet-A 3.1488 eV 393.75 nm f=0.1004 <S**2>=0.000
160 -> 166 0.18542
163 -> 166 0.61395
164 -> 167 -0.24161
165 -> 166 0.12678

Excited State 4: Singlet-A 3.5151 eV 352.72 nm f=0.0000 <S**2>=0.000
162 -> 166 0.29893
165 -> 167 0.62726

Excited State 5: Singlet-A 3.6963 eV 335.43 nm f=0.0000 <S**2>=0.000
160 -> 167 0.21871
161 -> 166 0.52754
162 -> 166 0.29835
164 -> 166 0.27640

Excited State 6: Singlet-A 3.7458 eV 330.99 nm f=0.0450 <S**2>=0.000
160 -> 166 0.61250
161 -> 167 0.23744
163 -> 166 -0.24125

Excited State 7: Singlet-A 3.7850 eV 327.56 nm f=0.0000 <S**2>=0.000
165 -> 168 0.69160

Excited State 8: Singlet-A 3.7945 eV 326.75 nm f=0.0019 <S**2>=0.000
165 -> 169 0.69755

Excited State 9: Singlet-A 3.9469 eV 314.13 nm f=0.0000 <S**2>=0.000
161 -> 166 -0.32927
162 -> 166 0.50245
163 -> 167 0.11979
165 -> 167 -0.25706

Excited State 10: Singlet-A 4.0724 eV 304.45 nm f=0.0318 <S**2>=0.000
159 -> 166 -0.30499
164 -> 167 0.16868
165 -> 175 0.59167

Excited State 11: Singlet-A 4.0986 eV 302.50 nm f=0.0230 <S**2>=0.000
160 -> 166 0.12400
163 -> 166 0.22667
164 -> 167 0.61749
165 -> 175 -0.16341

Excited State 12: Singlet-A 4.1154 eV 301.27 nm f=0.0000 <S**2>=0.000
162 -> 166 -0.12999
163 -> 167 0.62222
164 -> 166 0.21346
165 -> 171 0.12704

Excited State 13: Singlet-A 4.1291 eV 300.27 nm f=0.0000 <S**2>=0.000
163 -> 167 -0.12562
165 -> 171 0.65846
165 -> 174 0.17093

Excited State 14: Singlet-A 4.1353 eV 299.82 nm f=0.0004 <S**2>=0.000
165 -> 170 0.69214

Excited State 15: Singlet-A 4.2169 eV 294.01 nm f=0.0092 <S**2>=0.000
165 -> 172 0.69000

Excited State 16: Singlet-A 4.2292 eV 293.16 nm f=0.0000 <S**2>=0.000
165 -> 171 0.15264
165 -> 173 0.55898
165 -> 174 -0.34395
165 -> 183 -0.11596

Excited State 17: Singlet-A 4.2802 eV 289.67 nm f=0.0000 <S**2>=0.000
165 -> 171 -0.13627
165 -> 173 0.39282
165 -> 174 0.52930

Excited State 18: Singlet-A 4.3800 eV 283.07 nm f=0.0036 <S**2>=0.000
165 -> 175 0.10256
165 -> 176 0.64270
165 -> 177 0.20368

Excited State 19: Singlet-A 4.4144 eV 280.86 nm f=0.0040 <S**2>=0.000
163 -> 169 0.47614
164 -> 168 0.49859

Excited State 20: Singlet-A 4.4156 eV 280.79 nm f=0.0000 <S**2>=0.000
163 -> 168 0.48691
164 -> 169 0.48837

Excited State 21: Singlet-A 4.5132 eV 274.72 nm f=0.0567 <S**2>=0.000
160 -> 166 -0.10393
161 -> 167 0.14031
162 -> 167 0.60522
164 -> 167 0.10183
165 -> 176 -0.10104
165 -> 177 0.17847

Excited State 22: Singlet-A 4.5222 eV 274.17 nm f=0.0181 <S**2>=0.000
162 -> 167 -0.20292
165 -> 176 -0.19755
165 -> 177 0.57535
165 -> 180 -0.13407
165 -> 181 -0.10759
165 -> 187 0.10579

Excited State 23: Singlet-A 4.5584 eV 271.99 nm f=0.0000 <S**2>=0.000
165 -> 174 -0.17643
165 -> 178 0.62997
165 -> 179 -0.16234
165 -> 183 0.11615

Excited State 24: Singlet-A 4.6080 eV 269.06 nm f=0.0000 <S**2>=0.000
165 -> 174 0.10755
165 -> 178 0.26769
165 -> 179 0.44531
165 -> 183 -0.37098
165 -> 192 -0.19041

Excited State 25: Singlet-A 4.6273 eV 267.94 nm f=0.0057 <S**2>=0.000
159 -> 166 0.21851
165 -> 175 0.11648
165 -> 177 0.23300
165 -> 180 0.24584
165 -> 181 0.51789
165 -> 195 -0.10549

Excited State 26: Singlet-A 4.6458 eV 266.88 nm f=0.0294 <S**2>=0.000
160 -> 166 -0.22200
161 -> 167 0.61588
162 -> 167 -0.18882

Excited State 27: Singlet-A 4.6473 eV 266.79 nm f=0.0001 <S**2>=0.000
160 -> 167 0.63875
161 -> 166 -0.23228
163 -> 167 -0.11448

Excited State 28: Singlet-A 4.6718 eV 265.39 nm f=0.0045 <S**2>=0.000
165 -> 177 0.10816
165 -> 180 0.59850
165 -> 181 -0.31132

Excited State 29: Singlet-A 4.6883 eV 264.45 nm f=0.0000 <S**2>=0.000
165 -> 179 -0.30479
165 -> 182 0.58722
165 -> 183 -0.13488

Excited State 30: Singlet-A 4.7156 eV 262.93 nm f=0.0000 <S**2>=0.000
165 -> 179 0.39812
165 -> 182 0.33550
165 -> 183 0.43902

Structure and Atomic Coordinates

Table S4. Atomic coordinates of optimized structures calculated at the r²SCAN-3c level.

BAC

Coordinates from ORCA-job
tk43b_DAC_opt_freq_THF
E -697.673729465416

N	-1.86484373210638	0.03021301134486	-0.02751439573254
N	1.86484191778429	0.03024417621500	0.02750648072826
C	-0.00001632744270	1.86578999839914	-0.00002719161584
C	-0.68417033145269	0.63938422979220	-0.01208760888247
C	0.68415626972923	0.63939429079047	0.01206211033979
C	-1.98066020897006	-1.44100657584315	-0.08878827592433
H	-0.95164183092166	-1.80819534319802	-0.14973120185782
C	-2.61491658493940	-2.00795210406930	1.18016368174200
H	-3.65369462487853	-1.67849159358033	1.29578428580271
H	-2.05198053344841	-1.70212761831149	2.06835894552277
H	-2.61829372203503	-3.10256713527779	1.13603216870147
C	-2.71240629510635	-1.89584377674678	-1.35092197072689
H	-2.22665007661941	-1.49975670689263	-2.24879944327454
H	-3.76113644541858	-1.57848650266269	-1.35247095499012
H	-2.69922337298834	-2.98966755135417	-1.40825205277479
C	-3.10237427056993	0.84851483764291	0.01149943344488
H	-3.93020486861253	0.13077477311785	0.01391134525901
C	-3.23215106222889	1.72163343905982	-1.23476843978434
H	-3.18062349819874	1.11601088503253	-2.14577732181179
H	-2.43486029045526	2.47077239590084	-1.27101198902484
H	-4.19395987521956	2.24625208387147	-1.22444674049898
C	-3.18399838853460	1.66798963067362	1.29740013488515
H	-4.14631906466616	2.18932641424583	1.34630169440928
H	-2.38717148854884	2.41752359571948	1.33537850465700
H	-3.09647410501962	1.02359917679946	2.17877575878013
C	1.98068361345973	-1.44097337924415	0.08882378466539
H	0.95167365695273	-1.80817357960882	0.14984195959813
C	2.61486885917209	-2.00794249853252	-1.18015335288924
H	3.65363613360523	-1.67847084224607	-1.29584318706504
H	2.05187590967620	-1.70214228707573	-2.06832078625635
H	2.61826212227771	-3.10255689987696	-1.13599915456588
C	2.71251113886117	-1.89576807068182	1.35092416292709
H	2.22680371162258	-1.49966900802647	2.24882241713319
H	3.76123654380132	-1.57839672969435	1.35240202592474
H	2.69934555443849	-2.98959061788028	1.40827800079758
C	3.10235718673476	0.84856806124767	-0.01153577284485
H	3.93019323226338	0.13083387257861	-0.01403625533043
C	3.18392498095224	1.66808810418920	-1.29741195653038
H	2.38712515195613	2.41765599114132	-1.33530514853988
H	3.09630623729863	1.02374179583285	-2.17880999108642
H	4.14625894191509	2.18939702121218	-1.34634396672169
C	3.23217676503678	1.72163585374789	1.23476072976496
H	3.18074378088287	1.11597940703803	2.14575102751254
H	2.43483741426909	2.47072067910090	1.27107621667557
H	4.19395087570395	2.24631709608737	1.22439921584681

Xyl-NC

Coordinates from ORCA-job
tk43b_XyNC_opt_freq_THF
E -402.998368452780

C	10.52844694385320	11.23604498453363	8.25079968245337
N	11.20028742645165	12.15607470141431	7.97984450134007
C	11.98932150056611	13.24345139042205	7.65871303520818
C	12.87029592283160	13.12925654196207	6.56955085987878
C	13.65430324870707	14.24092419815788	6.26629594851761
H	14.34586894791889	14.18588750257122	5.42989810889003
C	13.55856886789928	15.40813735616792	7.01834159075802
H	14.17845234998313	16.26371348748235	6.76508940463888
C	12.67662317147353	15.48971773242009	8.09196311067548
H	12.60909936373943	16.40491054240427	8.67398324560391
C	11.87000454883933	14.40710595610898	8.43792855841523
C	12.95024906253490	11.85999059775540	5.77904524687603
H	11.98023533602801	11.60940741433523	5.33325770512830
H	13.68586543407319	11.95230642970407	4.97680944987909
H	13.23549339171432	11.01412770631312	6.41584476700918
C	10.91203446927843	14.46447106220456	9.58738084561925
H	9.88229807272109	14.28906800128870	9.25379284686676
H	11.14052639738412	13.69314294491719	10.33252521440080
H	10.95541552452353	15.44043787390169	10.07602590810488

INT1

Coordinates from ORCA-job
tk43b_DACCNXy2_opt_freq_THF
E -1100.699202071192

C	5.56493875326928	10.76816098122177	3.57686427332051
N	5.49230064979127	11.90333971842468	2.97550599279338
C	4.29229842749733	12.64309838427961	3.03634437593138
C	4.18043195075461	13.69593555551737	3.96617397946920
C	3.03012833930468	14.48691380514087	3.95589736385347
H	2.94195339738884	15.29770205781238	4.67642914605291
C	2.00451773869476	14.25262865577204	3.04410901553860
H	1.11544772618893	14.87745922473820	3.04707560057721
C	2.13197057293699	13.21820417117860	2.12156595745669
H	1.33892795946192	13.03408831641729	1.39939174273527
C	3.26605048721537	12.40319860328804	2.10071574693176
C	5.29221760425720	13.96742575032230	4.94004587574167
H	6.24690726371169	14.10996983553188	4.41871720858715
H	5.08035336690721	14.86498593336212	5.52867325946687
H	5.43842566103125	13.13130679912094	5.63346144953838
C	3.40508067215560	11.31127303287062	1.07669113769956
H	4.32432776824680	11.43667950180819	0.49113338905852
H	3.46317587338529	10.32037277666305	1.54031622592496
H	2.55407758849461	11.31805592901416	0.38915260203796
N	7.28100375966149	7.68808677843615	2.40554285464575
N	8.53959886218186	9.53142561518176	5.40641029053113
C	6.72463515514906	9.95164328026391	3.58019658958850
C	7.29677139056891	8.72101941180462	3.23993714597527
C	7.75987668416229	9.39611807819271	4.34083920328603
C	8.32564593654802	6.64410713617232	2.46401681839571
H	9.00130992876491	6.95410023139745	3.26761927284931
C	7.73087877475343	5.28630741938384	2.82905337758081
H	7.04281128739816	4.92797470686682	2.05508532269770
H	7.18979228854559	5.33939755980854	3.77952762134434
H	8.53184935376645	4.54593215717694	2.92924637038729
C	9.13767908334925	6.59263954383918	1.17080934680090
H	9.56889022092490	7.57196086203712	0.93925854494431
H	8.53044561109300	6.26686323318867	0.31930612796614
H	9.95756747587636	5.87543175465745	1.28479654795133
C	6.22863850133180	7.62926489650124	1.36179673075251
H	6.42297543732591	6.70718584423294	0.80370244325523
C	6.33658819489245	8.80889748626493	0.39829020654727
H	7.32198517843947	8.83679527118997	-0.07847387399825
H	6.17631005885948	9.75960061482397	0.91727664231102
H	5.57598992954252	8.72188406752338	-0.38497941107291
C	4.84344287914388	7.52344654941122	1.99604433449255
H	4.08284270001227	7.40377965140176	1.21690033806141
H	4.60582407744709	8.42636161753412	2.57089360176633
H	4.78740195202642	6.66115586417740	2.66923283688196
C	9.50341886343120	8.47688929639140	5.78623770411524
H	9.29409577647374	7.63730532875301	5.11564531966432
C	10.94280101639007	8.93323598526172	5.55891968762594
H	11.20420576509763	9.77408636506572	6.21120679148171
H	11.09701679314263	9.23808106322081	4.51847486925240
H	11.63261293675957	8.11223486120863	5.78252670990639
C	9.26814613641988	7.99413071037144	7.21610590271234
H	8.23740317076390	7.65063135138461	7.34990156025285
H	9.47514000472083	8.77816995116765	7.95250320936294
H	9.93960130939931	7.15576626472236	7.42987452283295
C	8.46185679153636	10.78552952035756	6.19608737191650
H	9.26595497791904	10.71222128847986	6.93613816052982
C	8.72981068193783	12.00807273182595	5.32237719009477
H	7.95531916106284	12.13063271344793	4.55801254801746
H	9.70099756233439	11.92626980476715	4.82247866418380
H	8.73473069708587	12.91144672233920	5.94138078975968
C	7.12819733540649	10.88289829052188	6.93383667298855
H	6.96974560073003	10.00970732146987	7.57519706374796
H	6.29508889273537	10.94447943315320	6.22374133123648
H	7.11033006659046	11.78040151728825	7.56172107028750

TS1

Coordinates from ORCA-job

tk43b_NEB1a_OptTS_true
E -1100.685614686549

C	1.18068613214861	-0.04397148732670	-0.87900569589702
N	2.37568412725408	-0.19496190059928	-1.21591915979708
C	3.46340464803217	-0.32750191109967	-0.32892737221795
C	3.42604344721383	-1.16137014859044	0.80905305911987
C	4.58886162541124	-1.29503687344796	1.57232654505958
H	4.57061890170160	-1.94603283504856	2.44386425189870
C	5.75588122700410	-0.61586048158003	1.23989230752250
H	6.64749824886874	-0.73162664564806	1.85019614627785
C	5.77865537332043	0.20383373135731	0.11451346137000
H	6.68869809864572	0.73611592214671	-0.15307104166003
C	4.64779484882292	0.35106626133425	-0.68668551668137
C	2.18659099024182	-1.91758031325978	1.19798797771507
H	1.70200781796160	-2.37504182960524	0.32824776986543
H	2.43369299567801	-2.70922999346284	1.91160227035519
H	1.44485914753370	-1.26251560524925	1.66857126555491
C	4.67358152862945	1.23004678760025	-1.90382964426658
H	4.40341098109332	0.66710340550537	-2.80493688436999
H	3.95026483491772	2.05021521085758	-1.82158777594489
H	5.66799208772414	1.66210292745570	-2.04773512615796
N	-1.68283661024766	1.91416376570180	-0.00087603051431
N	-2.36068077862653	-1.49828650343597	-0.25143887312566
C	-0.09747922159464	0.03391831142970	-0.78998184408892
C	-1.27834182397188	0.74676289487639	-0.48743168465772
C	-1.64783760394865	-0.50903926556567	-0.83658382575065
C	-3.13474794805083	2.10645719402774	0.21734865102453
H	-3.58775485635139	1.13658344517782	-0.02195602719566
C	-3.44065649292174	2.43225154169172	1.67675101693642
H	-3.01651092244618	3.39819196488473	1.97314479469133
H	-3.04807639823969	1.65833748106502	2.34446324062381
H	-4.52536692630143	2.48895916523794	1.81785413581394
C	-3.70600432351145	3.14947280032862	-0.74101471564281
H	-3.49238896709182	2.88411564085443	-1.78155835250863
H	-3.30092697315904	4.14807915232375	-0.54306905217291
H	-4.79292546283014	3.20226099608370	-0.61727943300681
C	-0.73889778538223	3.03439354284196	0.21977680955563
H	-1.36467566095487	3.88372521309398	0.51498428588039
C	-0.00141481926116	3.41310316997337	-1.06320059266010
H	-0.70616381718379	3.61697929901567	-1.87601296355783
H	0.67868231928790	2.61749256393719	-1.38441567451697
H	0.59534455001470	4.31477390655219	-0.88969220327921
C	0.22141387901506	2.72955438547147	1.36818047810318
H	0.83267150336468	3.61203415090369	1.58694072780661
H	0.89299256162587	1.90413247756242	1.10661425384337
H	-0.32648915119398	2.45564236830608	2.27591296250329
C	-2.60318676778818	-1.48946141270165	1.21219520365283
H	-2.25937302420219	-0.50398185694243	1.55074673951447
C	-4.09327328618823	-1.60662116261394	1.52878629993182
H	-4.49552237327108	-2.57769947431677	1.21746763616129
H	-4.66441972923063	-0.81913381778761	1.02532694175605
H	-4.25578882386440	-1.51295973756152	2.60868835375544
C	-1.78694864012587	-2.55107887475497	1.95281343225330
H	-0.71922037334737	-2.45764651079901	1.72979076965734
H	-2.10967678140027	-3.56515662460788	1.69134425619985
H	-1.91968205188916	-2.43093747548759	3.03421732083457
C	-2.58590016542249	-2.74942118338188	-1.00153134072650
H	-3.09927260576351	-3.43144710650335	-0.31333404513944
C	-3.50596101552697	-2.49912022484906	-2.19452496346165
H	-3.03769226175878	-1.80089091064405	-2.89820816986641
H	-4.46006830110799	-2.07046352842781	-1.86838403783766
H	-3.70926730319720	-3.43520869585816	-2.72671486854642
C	-1.27476876231307	-3.40394790367430	-1.43749258052386
H	-0.61234716569654	-3.57627625932791	-0.58267942918724
H	-0.74594616361518	-2.76893728703412	-2.15758686525668
H	-1.47331973653303	-4.36842183640506	-1.91830057502171

INT2

Coordinates from ORCA-job
tk43b_NEB2a_MEP12_opt_freq_THF_3b
E -1100.711518083263

C	0.37804235702066	-0.32243766119454	1.47394704368198
N	-0.07831643955401	-0.56612717875033	2.61289758095745
C	0.44346080272065	-0.45419527235005	3.89338757279430
C	-0.46325075401411	-0.73107732840275	4.94685770511987
C	-0.02381953359702	-0.64798381586280	6.26576181041301
H	-0.72524460268033	-0.86083388713098	7.06946008852129
C	1.29053817902043	-0.29838832398118	6.56352029948096
H	1.62189450775028	-0.23677095299667	7.59647018025045
C	2.17778786333400	-0.02977367150562	5.52585520068139
H	3.20702859575652	0.24144087198264	5.75175772503615
C	1.78414825113268	-0.10124594459900	4.18864705689659
C	-1.88316070998669	-1.10738863622785	4.63588708362745
H	-2.39126991732832	-0.31788405630195	4.06915290280386
H	-1.93149815263944	-2.01178294441421	4.01763151828037
H	-2.44471482029027	-1.28761483297278	5.55719074302516
C	2.78168363256620	0.18828017970360	3.10442497433625
H	2.90713294494130	-0.66718110794212	2.43115063809789
H	2.47381599283198	1.03684460668576	2.48265121869165
H	3.75645696691274	0.42291442463981	3.54173962879698
N	1.50943591170166	0.63030594339426	-1.85134074402135
N	-1.80459376559013	-0.18403948126732	-1.32353173182400
C	0.75795999100388	-0.19620104908661	0.27634237705806
C	0.51746480162133	0.32572037108985	-0.98666199283020
C	-0.85828527213267	0.68757540342469	-1.18708896722800
C	1.26201305262675	1.21000220896736	-3.19037526729081
H	2.24618233650798	1.23450522609806	-3.66845085774170
C	0.36163557612716	0.32959711110516	-4.05394196020311
H	-0.67854589556263	0.37028181084100	-3.71880154939663
H	0.69828891213509	-0.71238699144573	-4.04275915293921
H	0.38958020018254	0.68861743249031	-5.08848596843244
C	0.75192281433606	2.65080735118260	-3.11669139200843
H	1.41778373157854	3.27469333025066	-2.51281201669038
H	-0.25134703128889	2.69462697985194	-2.68260706744807
H	0.70891902457896	3.07040994016094	-4.12788459115022
C	2.91669928369083	0.42243334209840	-1.44675618713352
H	2.85782937674688	0.02752732003458	-0.42563566973679
C	3.69506697914953	1.73546554505847	-1.40267686841426
H	4.69881123140593	1.54491991233951	-1.00750045603212
H	3.20275658614289	2.46084918906931	-0.74660895618976
H	3.81048300547285	2.18253668495748	-2.39656543651120
C	3.59294593332552	-0.63045505057122	-2.32274935459750
H	3.02840621690689	-1.56889200238271	-2.30869202328497
H	4.59819724966530	-0.83137632485620	-1.93708152620458
H	3.69908336099936	-0.30234370693266	-3.36301038231058
C	-3.22768896184779	0.28917273960938	-1.25475551489379
H	-3.85308006397547	-0.60532460693305	-1.33044382154732
C	-3.49473000814348	0.95391926760682	0.08953160827054
H	-2.90823044231261	1.87311658377458	0.18848913511845
H	-3.23316983709650	0.28620739941492	0.91773748273310
H	-4.55721611893858	1.20697325973188	0.17076216436326
C	-3.53877611473171	1.20457557541656	-2.43128983625966
H	-2.91559241841798	2.10444039250523	-2.38992778548128
H	-4.59022035850338	1.50857623206448	-2.39505342721753
H	-3.36033301775268	0.69920246233624	-3.38641575340391
C	-1.60301005469240	-1.66208563118684	-1.49305568028994
H	-0.51807252024131	-1.78648261602209	-1.57539596788774
C	-2.09251085793011	-2.41920857466266	-0.26166635169118
H	-1.62239541074398	-2.04098262208414	0.65088316986944
H	-1.83149368603057	-3.47780068537866	-0.36926778692257
H	-3.18066055995785	-2.35685922529548	-0.15088884333479
C	-2.25285417473202	-2.17340420144678	-2.77567772413042
H	-3.34636461631156	-2.14368225883574	-2.72812387257731
H	-1.95989544453051	-3.21880000986248	-2.92092540244718
H	-1.92311510848140	-1.60553144543522	-3.65053904236850

TS2

Coordinates from ORCA-job
tk43b_NEB2aONIOM2_OptTS_true_afterGOAT
E -1100.689681575555

C	-1.23082926121743	-0.25237587982543	-0.13050765300399
N	-2.25204641937532	-0.99331486000822	-0.18762928511455
C	-3.60167032833744	-0.68114291813830	-0.16460313802819
C	-4.47316273366182	-1.73908806154609	0.20350083479466
C	-5.84749339476403	-1.51780411470715	0.23260951801369
H	-6.50589304047974	-2.33324365565174	0.52480166566803
C	-6.38459253488544	-0.27749025620803	-0.10517076121622
H	-7.45906945139349	-0.11785850184817	-0.08102173028802
C	-5.52946200615311	0.75175946298263	-0.48697287997722
H	-5.94105888166470	1.71856094440816	-0.77054018537794
C	-4.14482475454740	0.57838000056400	-0.52703079380518
C	-3.90615215392855	-3.08112715704202	0.56758113980240
H	-3.31812053363632	-3.50335250558679	-0.25638603034516
H	-3.22685366189552	-3.01054539095610	1.42575735885242
H	-4.70646983866975	-3.78407348367391	0.81751192466479
C	-3.26859203157695	1.70954310745722	-0.98297824220918
H	-2.56855547882729	1.38559976831887	-1.76171056062129
H	-3.87901262630993	2.52237890855725	-1.38789219038999
H	-2.66435067971713	2.11836754899566	-0.16507461068601
N	1.71181627528556	1.94770691939720	0.24787438973326

N	2.44440411611882	-1.39614003615865	-0.19313014699398
C	-0.14112073227044	0.34883127999242	-0.01982621410401
C	1.18236082022576	0.71301711244503	-0.04754064566816
C	2.24194323865894	-0.13480029087984	-0.50346425482413
C	3.12135328265894	1.84457285245398	0.39964381413966
H	3.19233373667728	0.72966294507766	-0.49858083774281
C	3.96285908328499	2.94080329435300	-0.19521112981245
H	3.60775065750331	3.24095242173545	-1.18605403597367
H	4.99614001259255	2.58940069070679	-0.29005960497512
H	3.99104353303379	3.83007541253611	0.45116368433781
C	3.61371939366156	1.31398598430988	1.72769816048426
H	2.92779715603959	0.56998295543981	2.14225633320380
H	3.70732092303636	2.13587203193683	2.45324114563795
H	4.60387121391486	0.85551946757238	1.62074829912505
C	0.97514104375969	3.22311678215025	0.15903106353524
H	1.73410360721781	4.00266660699161	0.28445492296041
C	-0.01849906931376	3.35132283954257	1.31444418810196
H	-0.49745410006636	4.33577770894515	1.28077192675047
H	0.49129803231781	3.24779747500407	2.27832408338054
H	-0.79747252948009	2.58586999874812	1.25029550191297
C	0.31669167014166	3.40694632952910	-1.20582521392164
H	1.05557839483195	3.32471590593730	-2.01019284406603
H	-0.14479229310801	4.39855728536580	-1.26254011745564
H	-0.46266660316736	2.65762824496750	-1.37323917915862
C	3.36880753944848	-2.20077859936019	-1.04301746269241
H	3.34695868969646	-3.21744588465914	-0.63819132643929
C	2.86891748046631	-2.24332834491160	-2.48269304501655
H	3.51053645442249	-2.90049015077978	-3.07923558556200
H	2.88853974837299	-1.24344224315340	-2.92947759824294
H	1.84308037875889	-2.62362130579511	-2.53080273966334
C	4.79697718629715	-1.67277505824751	-0.94391084652555
H	5.47189943548101	-2.32758000124079	-1.50523871606498
H	5.13690630406001	-1.64096630316737	0.09644808844731
H	4.87439524232990	-0.66579406108107	-1.36879331186958
C	1.81346118096047	-2.07326916570656	0.97484654953638
H	1.23465567937233	-1.29092453786703	1.47599496638234
C	0.85333191772669	-3.16900296062899	0.51682512566164
H	0.10096678947870	-2.77243235884791	-0.17152937713662
H	0.33392042590862	-3.58034207021019	1.38918596467070
H	1.38215754153112	-3.99441215718860	0.02702165915965
C	2.86294429193398	-2.60376389978464	1.94954628662900
H	3.54947929044928	-1.81268092849815	2.26633757224211
H	3.44784412733152	-3.42500301094895	1.52148406129114
H	2.35530330038072	-2.99139718302433	2.83959367709086

INT3

Coordinates from ORCA-job
tk43b_alkene_opt_freq_THF
E -1100.719552749550

C	1.15914465211395	-0.67904835182614	0.39877073465555
N	2.35301023180448	-1.03943928655170	0.67216889312854
C	3.51099398447047	-0.79465276330074	-0.03950970581318
C	4.66073419003472	-1.52157504600474	0.38155506089653
C	5.88243466627102	-1.32086234678824	-0.25328925179607
H	6.74734277599478	-1.89129192057467	0.08035444467927
C	6.01313689418604	-0.40949705212833	-1.30019120965485
H	6.97253083659019	-0.26013137024984	-1.78776735809947
C	4.89404243247981	0.31154222294129	-1.70626049007702
H	4.98448529725328	1.03243513085455	-2.51728271183415
C	3.64514935983925	0.14104280342452	-1.10498778212377
C	4.54339211406196	-2.50342758442064	1.51183688805355
H	3.80519788116651	-3.28425716801596	1.29193279660271
H	5.50701635067665	-2.98378734163241	1.70837750263172
H	4.20495578144917	-2.01446584411636	2.43374264305212
C	2.48490907170723	0.96414758291290	-1.59405665084151
H	1.71297690078985	0.34932546230211	-2.06918089445719
H	1.99369100578377	1.50314278868772	-0.77426826368465
H	2.82930215473696	1.69994260733019	-2.32813055749018
N	-0.81484318143460	1.78445249066213	0.45334018295597
N	-3.20387065632124	-0.92888562411833	-0.31807358316789
C	-0.08584542616432	-0.53405973622495	0.29945342870921
C	-1.15749478700295	0.37552680276535	0.21767254377789
C	-2.49043452953243	0.19757658129144	-0.04699607317475
C	-0.73146730769958	2.63155236095959	-0.53650297639311
H	-3.09351076168774	1.10272449014066	-0.04412421566236
C	-0.45235027885637	4.08239161041325	-0.30672807363009
H	0.49352588196131	4.23840329696863	0.22235531041398
H	-1.24565345642514	4.54166606343179	0.29557350325307
H	-0.40398029194373	4.60413987853722	-1.26335224277844
C	-0.91252807793965	2.20388445831236	-1.94887548416620
H	-0.90044459598384	1.12172364218408	-2.07235795688894
H	-0.13006122249657	2.67171627994828	-2.55783886070193
H	-1.86952715856120	2.58942182340501	-2.32723255658435
C	-0.50554910993779	2.21185246429449	1.86635055203005
H	-0.80614535197885	3.26180352441935	1.92545622288540
C	0.98903863595994	2.11133044641189	2.16777735618450
H	1.59755924786074	2.58011078007170	1.38839275632592
H	1.30032274821085	1.06852230287107	2.26633519594124
H	1.19204126444550	2.62732596001004	3.11229066423527
C	-1.33922406857771	1.42972826713655	2.86842830113910
H	-1.14404879594226	1.84024031938347	3.86470348876523
H	-1.07055000761455	0.36936636121944	2.87515237281660
H	-2.41032111657733	1.52182980595712	2.66442732229755
C	-4.67420055334631	-0.81626048836096	-0.40082659088510
H	-5.03956684728172	-1.83844172128175	-0.54411711539494
C	-5.29362955717611	-0.28267240063923	0.89336380098615
H	-5.03288112308131	0.76869987284273	1.06032530305616
H	-4.95551138055099	-0.86251188828818	1.75853950673683
H	-6.38605566532088	-0.34845952265094	0.84045254834149
C	-5.10923960734205	0.00643338531905	-1.61489037199207
H	-4.68074971858690	-0.40172777994824	-2.53637856775621
H	-4.79504909692239	1.05271048090443	-1.52282654807570
H	-6.20103970465795	-0.00501278993043	-1.70672205890910
C	-2.59935190574387	-2.26981561700176	-0.34193254531534
H	-1.51737687050231	-2.09781107340074	-0.28348442306675
C	-3.00450349924978	-3.09809289511572	0.87858105113139
H	-4.07975621114751	-3.31293207444608	0.89102268331032
H	-2.74273028509652	-2.57537642370755	1.80534528828054
H	-2.47436149080535	-4.05716803947895	0.86859178714459
C	-2.89152140407259	-2.99552918908779	-1.65509604833806
H	-2.56340383200106	-2.39723687723747	-2.51200170378236
H	-3.95627489470703	-3.22533188139588	-1.77904861534184
H	-2.34668652957748	-3.94592625038972	-1.67571864654088

1_{Xyl}

Coordinates from ORCA-job
tk43b_XyNCCC_opt_freq_THF
E -1100.742403622846

C	4.95509508172287	11.97859869006911	19.09444779256033
C	4.47372542931284	10.74016812154703	19.29702002670840
C	3.82115611187103	9.67175609212513	19.39970836059035
N	3.04328903355626	8.69659349799458	19.49998529170150
N	6.19407153299421	12.50182218886466	19.14311101837028
C	7.49027615577782	11.93379094404477	19.50807444686362
H	8.19175015869040	12.77837979006088	19.48376228630138
C	7.92835993180474	10.89962173208253	18.47020144523236
H	7.23261792664997	10.05381580440520	18.45021032225581
H	8.92384528929452	10.51784233828411	18.71984171165756
H	7.96731454648018	11.34207541798517	17.46949605161684
C	7.46162332045134	11.35429742834262	20.92165827388194
H	7.14775020610734	12.10975060655932	21.64937666186515
H	8.46045973980274	11.00101975478854	21.19800638357050
H	6.77011226807812	10.50695974180723	20.97993316529901
C	5.81502153195061	13.90118078349472	18.81744957130619
C	6.39324466789528	14.39537027162394	17.50619326542209
H	5.90577546210675	15.33279570210675	17.21772465353654
H	6.26036686518071	13.67237832840929	16.69801748951476
H	7.46576529466333	14.59564162141416	17.61721759225358
C	6.10134048891637	14.86696866677672	19.95606483508777
H	5.66158228890081	14.51397538865435	20.89532213990070
H	5.67918395688841	15.85128879844791	19.72400468708224
H	7.18064352218991	14.99494990422518	20.10266873070749
C	4.31909318491374	13.35413500269127	18.78439807796063
H	3.81182351383470	13.71144580053425	19.68763208471402
N	3.47293114639782	13.55475820873182	17.65386619812418
C	3.64712211610838	12.69894033252652	16.46726501633967
H	4.64156055209161	12.24464646593537	16.56766711640854
C	3.64316623920024	13.53492745881151	15.18389596819747
H	3.85795766977979	12.90200597527678	14.31502161186615
H	4.39366414983213	14.33047154722143	15.22691808413353
H	2.66420866720168	13.99967083583410	15.01641416063454
C	2.63936742158683	11.54692048120948	16.34717659091998
H	2.64411960136366	10.91452906263747	17.23932891755686
H	2.89965537274099	10.92054412255234	15.48575585909599
H	1.62080030731110	11.91834141644084	16.18593006042675
C	2.12650152527096	14.09041530880059	17.91662797143346
H	1.60389718478457	14.06837542557979	16.95237881004766
C	2.20312847060572	15.55402602661431	18.35797300484092
H	2.72131172095681	15.65282554315951	19.32000991107333
H	1.19773585072840	15.97294122240994	18.48203674547980

H	2.74173488274879	16.15519720910398	17.61722001172790
C	1.29466328702638	13.26972263017226	18.91393397993969
H	1.24641833311329	12.21481078009582	18.62929424282058
H	0.27022943047957	13.65802975393421	18.95848519410355
H	1.70882389482558	13.32962098484584	19.92749763749046
C	3.29524096545538	7.34987648181520	19.75209657063983
C	4.54679431284483	6.71927769422366	19.54960016802216
C	4.65593820658109	5.35159559364019	19.80768693007296
H	5.61618750840842	4.86576839029004	19.64757867630718
C	3.57062802262025	4.60660812213231	20.25716500431821
H	3.68073325493297	3.54355717884510	20.45325121972872
C	2.34132883326367	5.23224479572554	20.44495053446429
H	1.48560564554965	4.65685154060834	20.79097114429383
C	2.18257045840897	6.59296714863051	20.19273123542846
C	5.74157330653734	7.47772022315600	19.04824184022709
H	6.10847097372106	8.19509433027853	19.79092780501749
H	5.50518358328583	8.05052655800912	18.14411186833053
H	6.55731216278703	6.78760123888451	18.81398680627325
C	0.85071405764311	7.25678505531388	20.39290548434953
H	0.10615841467413	6.53069242554023	20.73178568477234
H	0.49033495081889	7.71551898324411	19.46457124143440
H	0.91087026965307	8.06233521042676	21.13399611769355

TS4

Coordinates from ORCA-job
tk43b_NEB3a_OptTS1_afterGOAT
E -1100.691600913809

C	-1.06536044173059	0.36426438719875	0.43865899462226
N	-2.22105263203107	0.60989291400994	0.04997377911608
C	-2.69405676460180	1.54304189631533	-0.87421421218337
C	-3.40082903210677	2.66769989239019	-0.40606512990266
C	-3.90438849233814	3.57503248765950	-1.33792733841642
H	-4.43549454415486	4.45545346409150	-0.98274887517228
C	-3.73034141605786	3.36996631257024	-2.70405685509539
H	-4.12600775193822	4.08685639865782	-3.41837973839160
C	-3.06319637181647	2.23309061207967	-3.15180787824222
H	-2.94527800534587	2.05788654726209	-4.21899029536431
C	-2.54701154342415	1.29708237673982	-2.25362522987405
C	-3.55854409908724	2.89056585942393	1.06893944051142
H	-4.02307607679778	2.02422674294157	1.55494986965641
H	-2.58517527971992	3.03590538091100	1.55496328843683
H	-4.17416111718678	3.77258283585002	1.26631545157969
C	-1.88165939448924	0.04050423891470	-2.73898652789809
H	-0.83818023474773	-0.02142495611223	-2.41123500288908
H	-2.38845877875101	-0.84648993512575	-2.33778561093604
H	-1.90679927213486	-0.01210266521116	-3.83112881404782
N	1.84314762514414	-1.45833412220751	0.38130735381776
N	1.56841688781743	1.77427716304040	-0.10908419114503
C	-0.23835488316310	-0.36520063856389	1.23719532230345
C	0.90968349446954	-0.50926629751060	0.43529405358836
C	0.78194563887667	0.72954868124428	-0.29459522621477
C	2.91849017597289	-1.36619986489446	-0.63254757158782
H	2.85123689665345	-0.35290357323030	-1.04218582603490
C	4.30182465948235	-1.54015714079067	-0.01031094771835
H	4.45713708212762	-0.84411491700502	0.81980553581765
H	5.06624031925527	-1.34386086030398	-0.76948777521814
H	4.45932142952166	-2.56012076695255	0.35676644976123
C	2.67672300572649	-2.34775487237741	-1.77937861101164
H	1.69586275814616	-2.17927995336270	-2.23640780296190
H	2.73277025598368	-3.38874459989666	-1.44253542660267
H	3.44280406451109	-2.20566102457349	-2.54917330034001
C	1.70642811913845	-2.72421002387747	1.14607687927226
H	2.51049157764966	-3.36864115666797	0.77618512877942
C	0.38322254878077	-3.43312309245674	0.86295313276188
H	-0.46526671781377	-2.87953390622142	1.27603887945388
H	0.40210423261077	-4.42553385742210	1.32597431069090
H	0.22625915420014	-3.55875279865552	-0.21352818063944
C	1.93929892921084	-2.49756845536918	2.63821373603808
H	1.93067775248720	-3.45957469562796	3.16236610442248
H	1.15473481926145	-1.86543610270882	3.06479475588258
H	2.90857199461241	-2.01951583045109	2.81545128904380
C	2.70286211349341	1.78948572725998	0.86211596201143
H	2.75650152129041	0.77155872277958	1.26042315326114
C	4.03239487475373	2.10971075533989	0.18188021197721
H	4.08729212090842	3.15409787378281	-0.14277757486836
H	4.20750454217122	1.46655091855815	-0.68617563121774
H	4.84706485643991	1.94767790119633	0.89577611452483
C	2.42376016134986	2.73422852667821	2.02928076355315
H	2.39482065912455	3.78061838274966	1.70698466958082
H	3.22730868151981	2.63590193445767	2.76756748760257
H	1.47671969280728	2.48768674266491	2.51953551658979
C	1.26427708501013	3.05818572676561	-0.80485316686954
H	2.13128718944708	3.70216373029398	-0.62601569245521
C	1.13345835492366	2.83943248310158	-2.30781647563834
H	2.02380649393464	2.34641120995378	-2.71333552955915
H	1.01243266586154	3.80483174101158	-2.81079194041599
H	0.25804887727048	2.22289672487399	-2.53712226651185
C	0.03044984982336	3.73767579815983	-0.22268024857448
H	0.11873078394968	3.88283268080700	0.85770000877514
H	-0.86261458916806	3.14150086532484	-0.42534742524005
H	-0.10621832771203	4.71867189801039	-0.69134752487374

INT5

Coordinates from ORCA-job
tk43b_CBY_opt_freq_THF_afterGOAT
E -1100.714356715984

C	-1.31469830245999	9.42129161862580	5.33526611671751
N	-1.34167997952416	9.31811116114049	6.61211013327914
C	-2.58229430317920	9.19150274146287	7.26843130179593
C	-2.99963762003151	10.25162000699568	8.10065614292121
C	-4.18751158244127	10.12451806712373	8.82044344538966
H	-4.51110898512870	10.94751242698030	9.45388107736738
C	-4.95505208396170	8.96618164119454	8.73724715845582
H	-5.87661528452034	8.87752800544886	9.30575927884570
C	-4.52640674837214	7.91732274137775	7.93107524819328
H	-5.11441321134363	7.00356435127658	7.87289006803821
C	-3.34468223912621	8.00636564821091	7.19115696270641
C	-2.17195023282210	11.50167342705036	8.19686914894824
H	-2.12241596862252	12.02711213354543	7.23632354006550
H	-2.59048852186312	12.18899853520343	8.93716082019314
H	-1.13812216044597	11.27171424055282	8.47913360126223
C	-2.89357521741012	6.84671016923794	6.34976820131700
H	-1.84399655902562	6.60056851272885	6.54184861661409
H	-3.49911162074553	5.95995069074357	6.55891158444839
H	-2.97420255626200	7.07357578302285	5.27999033050352
N	-1.26369099673479	10.88693854500500	2.33187641334226
N	0.89030060502900	8.92950041655423	4.07851331588680
C	-2.24848692693391	9.46938121363785	4.20782282707327
C	-1.26157521458859	10.13668432358492	3.43464084281655
C	-0.24831432958318	9.58419092045121	4.31245993515143
C	-0.05618409594646	11.65331599250354	1.94506761023864
H	0.74731995864306	11.27672814472769	2.58497065279100
C	0.32649334434078	11.41748990011229	0.48489282923689
H	-0.39371047468139	11.87656370749230	-0.20096962144711
H	0.39238251282893	10.35097569548247	0.24813357317239
H	1.30269182812661	11.87293657453904	0.28837645265003
C	-0.21499249986339	13.14392863685824	2.24786305820240
H	-1.00344089561399	13.60123284977677	1.64043760418970
H	0.72349529895177	13.65941490113882	2.01671276538594
H	-0.44464823584924	13.31007653858116	3.30486384332557
C	-2.54809150582759	11.22208765314351	1.66276889486137
H	-2.28130608682877	11.93230678781601	0.87316872146717
C	-3.53207934128882	11.91642425471866	2.60367946730088
H	-4.40301647495207	12.25321206362649	2.03100642251934
H	-3.07555647645489	12.79001275144647	3.08028251306681
H	-3.88038452292944	11.23462853672825	3.38604146395521
C	-3.15345103965710	9.98897607705852	0.99547697984780
H	-4.03712098390031	10.28026368696457	0.41720462118877
H	-3.45874311508681	9.24926588993193	1.74147046129554
H	-2.43407019393422	9.52283414573167	0.31336890891889
C	1.33741388012372	8.65501698265496	2.69020724027611
H	0.58399757003317	9.11322169076528	2.04494418826179
C	2.69950954722109	9.27544265927625	2.38322480735314
H	2.89647969215029	9.19401115895146	1.30925544150501
H	3.51056593560275	8.75616040239228	2.90404845512973
H	2.73615880986298	10.33404822634076	2.65809948499783
C	1.32322628399135	7.15431815174489	2.40059705157635
H	0.32916932589839	6.72928619487165	2.57307970039982
H	2.05088391838030	6.61473533816227	3.01517730043790
H	1.58753430024616	6.99238294024728	1.35016968699675
C	1.71223112132279	8.37911550865568	5.18941093044265
H	2.64182424265458	8.05934311341410	4.70834736797454
C	2.05238218038933	9.47581049820303	6.19389911345130
H	1.14875340002966	9.83520572509694	6.69664229907010
H	2.54679723928325	10.31900358821686	5.70014785343995
H	2.72981553231285	9.07659434420547	6.95549439686357
C	1.08757389739381	7.15703158515723	5.86116852791350
H	1.84478479574260	6.66544639205764	6.48171567939285
H	0.72707058933640	6.43336289724736	5.12475650421081
H	0.25517662737927	7.45150330198436	6.50395224280400

Cyclobutene 3

Coordinates from ORCA-job
tk43b_CB_DAC_opt_freq_THF
E -1798.489625482860

N	12.43602082205532	7.70301579098803	11.65505486466563
N	13.80746243209365	9.97746653986771	13.58088815757304
N	12.98588205969290	12.34416368865573	11.37921351115500
N	10.72738077696257	9.79976284062997	7.03450172097399
N	8.02788412999173	9.85583677205354	9.15033466653581
C	12.75134422532469	11.03399609322189	11.45126767879093
C	13.08075256754427	10.01353495919474	12.37330013082486
C	12.48806391217735	8.98115217539379	11.54849354040893
C	11.94451219397613	8.86026571597188	10.65229663842572
C	12.04399934866353	10.04990557390992	10.55474047154226
C	11.34537006862199	10.05475252025052	9.44888935292451
C	12.67105672676446	6.65886653501341	9.45801333731463
C	10.54558118458959	10.05256265667894	8.40938741213331
C	14.91767385266966	8.97998593790317	13.52889000080356
H	14.51805647215800	7.97289662621756	13.31927992071198
C	9.02783774528112	10.22365917185274	8.20014136263489
H	8.87687339576596	11.26980611959755	7.89749874500072
C	10.80815394387638	6.06828347637833	10.92896228107625
C	13.97586605966044	7.36928165650801	9.23540351979975
H	13.85995910601817	8.45739981630558	9.21617648322156
H	14.43100500365312	7.05705773243810	8.29089318854369
H	14.67982509724581	7.14279073691424	10.04744908639422
C	13.91159917050302	12.96349949079367	12.36295178542028
H	14.12533500240226	12.17517476999629	13.09118958014064
C	12.94454330268695	9.91259099563000	14.79660643021999
H	13.62427316505379	9.99570615665810	15.65210025077352
C	11.87903809040283	9.06323339500731	6.50057346895350
H	11.98317616869352	8.10330965791408	7.03821196369912
C	9.28312342543411	9.41320424555177	6.85781562362401
C	15.87048883476277	9.35752193103701	12.39558481097810
H	15.35290490545383	9.43580762884835	11.43426100746796
H	16.65001119161741	8.59468337303508	12.29453613055191
H	16.35457747640604	10.31950064771399	12.60677077425987
C	10.34957851786790	5.17515099131549	9.96025233444609
H	9.46002264714186	4.58362399755743	10.16789647613360
C	10.14380376981740	6.15831738843710	12.27200708388547
H	10.85675512654805	5.90993193836371	13.06919967863658
H	9.29949992540751	5.46600382338750	12.33753622401271
H	9.78106610356672	7.16743760197633	12.48954087150716
C	13.15230630214815	9.88161298065099	6.70722183282698
H	13.08451298995452	10.83130584791989	6.16243590936865
H	14.01654887591973	9.32559055416756	6.32753261172140
H	13.33111587498848	10.10024827901700	7.76223975261733
C	15.68954908767902	8.92889315380506	14.84437442058838
H	16.01737068310594	9.93204948634543	15.14486456396714
H	16.58202434687070	8.30926572944759	14.70648873506454
H	15.11236887669326	8.48876460655631	15.66273798885291
C	12.18962292623354	5.74239991568171	8.52089416443271
H	12.74739991791544	5.59146130312904	7.59872253238114
C	8.35906647197396	8.98945543536219	10.29168850038855
H	9.31235537826664	8.50887201161486	10.04249841310684
C	6.90686281421436	10.79455413973729	9.32982526142462
H	6.30031707132041	10.37764682031041	10.14291096988633
C	9.01485870260189	7.92913101654053	7.05070645120523
H	9.34471352681742	7.34030614618666	6.18827324601287
H	7.93858012677148	7.76571295972153	7.17416871372322
H	9.51959250060477	7.54308848935942	7.94279433268836
C	12.00974675536403	11.11554495398977	14.85161530909328
H	12.57180929515303	12.05357113874178	14.86862873079079
H	11.40431620022845	11.06695425178456	15.76330934678039
H	11.32390394254808	11.13144420487685	13.99578200546823
C	11.02391276554818	5.01767240761543	8.75197852432470
H	10.65747754664740	4.31677269081688	8.00664629421453
C	12.15213316206126	8.60887908673542	14.93196261315407
H	11.38113383416196	8.54030416613179	14.15700003491921
H	11.65721368552784	8.57328597777072	15.90977705746056
H	12.79554377853806	7.72704427914599	14.84782636458896
C	13.27044181431208	14.14061700783365	13.09789919055057
H	13.17731461616813	15.02945915045738	12.46451424153869
H	13.90272764674399	14.41441112432580	13.94953014250105
H	12.27833755426873	13.88240711988666	13.48048754390544
C	15.22538450684414	13.36261899312343	11.69262859198336
H	15.68520099421858	12.50689314017453	11.18743845688189
H	15.92539165525913	13.72850787080427	12.45219201134960
H	15.08153439703089	14.16476824625474	10.95949117626416
C	8.62509653354046	9.96155064119804	5.60766846975056
H	8.83615366566910	11.03043184615703	5.49123813622320
H	7.53932967751659	9.82630400758412	5.67148460043310
H	8.96516183886241	9.44114007801446	4.70621573128104
C	11.69659796096441	8.77888529650929	5.01268010380290
H	10.84461732877904	8.12625155982976	4.80817870612053
H	12.59400545551306	8.28012881480852	4.63135920759700
H	11.56292624139379	9.71301073255072	4.45421266696315
C	12.43281676063578	13.23560670467200	10.32875293277292
H	12.81090392836476	14.22798789033327	10.59134630567920
C	8.55897759115435	9.75378853920711	11.60976405762589
H	9.31756654983031	10.53569798678151	11.50353205069500
H	8.89105237322780	9.07001375579552	12.39860368600514
H	7.62667333934693	10.21648575230505	11.95423734973051
C	10.90796695885610	13.29224125848364	10.38968772099766
H	10.46930412096283	12.32849853200245	10.11708483058562
H	10.53878445451579	14.04577004219207	9.68497971413724
H	10.56644478640689	13.56299516036773	11.39446957758693
C	7.31686682360889	7.88087384674135	10.46710279300794
H	6.32313625591627	8.29237946324337	10.68170226589832
H	7.58778952630972	7.22824015401885	11.30454837001701
H	7.24429436517578	7.26791003492035	9.56259851642591
C	7.30541409127099	12.22146235514681	9.74295658016207
H	7.95503551463413	12.22482579340527	10.62184059689331
H	6.40605099381977	12.80102343309015	9.98311525536161
H	7.82040249586983	12.74968259490128	8.93208752279245
C	12.95261158916130	12.91314842213754	8.92966754144480
H	14.04001329072155	12.78562278958462	8.93040586668316
H	12.70467068406233	13.73905807022227	8.25336411707035
H	12.49157665499509	12.00554114477060	8.53727336197226
C	6.01668320369514	10.84402026798122	8.08574750639905
H	6.55001725661651	11.28730843688266	7.23700807570299
H	5.12905463899611	11.45920544458248	8.27534255094007
H	5.68705447634742	9.83935329438519	7.79934662904641

INT4

Coordinates from ORCA-job
tk43b_NEB4a_MEP15_opt_freq_THF_afterGOAT
E -1503.761075149029

C	1.21941867792908	1.93040796833323	-0.20705116556244
N	0.96084810063597	3.16149871958313	-0.24295435168917
C	-0.23177078576026	3.73074541325428	-0.71245331682854
C	-0.89193568193230	4.64714723897797	0.13452516242769
C	-2.05439111878129	5.27107376870251	-0.31728760792565
H	-2.57206891279808	5.96305069850763	0.34386804499422
C	-2.55916243854993	5.01442227697637	-1.58901158130241
H	-3.47281021688675	5.49998736813522	-1.92101510945047
C	-1.89078749789128	4.12425882069502	-2.42435605551225
H	-2.27605391971921	3.92396212775009	-3.42225561270836
C	-0.73043947643132	3.46797680783437	-2.00756723906243
C	-0.36540808810784	4.90654327896580	1.51629153581764
H	0.70223001015166	5.15282738056402	1.49785466066461
H	-0.90803936562567	5.72931441968710	1.99110663265176
H	-0.46720448857609	4.01782091477262	2.15339154201625
C	-0.02862852171820	2.50772511684279	-2.92479135662816
H	-0.44197144523715	2.57114813573417	-3.93563925530096
H	1.04698574409962	2.71496240681007	-2.97509664460817
H	-0.13430273651802	1.47049285892753	-2.58287445250928
N	2.83141150154538	-1.20992756831268	0.50304055516979
N	0.07564011923336	-2.66213674449176	-0.13428259751497
C	1.65281497784975	0.76527200672792	-0.06732365250402
C	1.68572480998364	-0.59952039125375	0.11660478206329
C	0.37790302788594	-1.26902196552613	-0.09990124851898
C	2.95965678715739	-2.66895789575215	0.72443848507725
H	2.01723663534302	-3.09907897909623	0.38675955219636
C	4.07923981949749	-3.27345267213328	-0.12507319031851
H	5.07828566558428	-2.96604982695480	0.20327425332649
H	3.96351500243847	-3.00701271674882	-1.18055873933086
H	4.03184637502642	-4.36538424873022	-0.04382927748486
C	3.13101183457849	-2.99274315952757	2.20974236351540
H	3.17367650423166	-4.07980258136368	2.34153382594844
H	2.28817975609804	-2.61062626188420	2.79596409400729
H	4.05745637830561	-2.57711146259353	2.62260094731429
C	4.05009953572268	-0.41341877403005	0.82225921965038
H	4.77578266244283	-1.16223683068447	1.15099762363824
C	3.85190158944667	0.53529438273388	2.00641175559197
H	3.47680091484025	-0.00989552680382	2.87972899547484
H	3.15685296326611	1.34629452603487	1.77783973808519
H	4.81986937208784	0.97461653768469	2.27264825584293
C	4.65494253860353	0.26906520052490	-0.40564961186417

H	5.61095927241547	0.72534531088395	-0.12515856558803
H	4.00449094227042	1.05320931626488	-0.80071176222379
H	4.84270941428053	-0.46008498035097	-1.20142326772435
C	-0.23223330666047	-3.25748774166778	-1.45513824079557
H	-0.62221436150072	-4.26046938954415	-1.24295038806248
C	-1.28961803507723	-2.51640838354415	-2.28122494053242
H	-2.20766761769827	-2.34240544402914	-1.71280836737646
H	-1.54559328766167	-3.11523316730153	-3.16306213083142
H	-0.91363119486422	-1.54942941259165	-2.63536193871984
C	1.05503708488101	-3.41049459661591	-2.26629073058833
H	1.75803293760286	-4.08722832625844	-1.77048963219761
H	1.54423019993158	-2.43689818071282	-2.39801434117167
H	0.83646776205820	-3.81233713366823	-3.26238553060587
C	-0.60075197564422	-3.21523885856706	1.06178571772566
H	-0.22109893290392	-2.60979999148540	1.89689679765688
C	-2.12841473678523	-3.09336862212708	1.04121776820749
H	-2.56168160176779	-3.71312734172098	0.24738963393962
H	-2.45324852610622	-2.05894149991535	0.88739850108862
H	-2.54461495288776	-3.43567867875896	1.99617861896921
C	-0.18890330027655	-4.66704691918759	1.31821585831184
H	-0.58698830788659	-4.99421400297506	2.28548399220551
H	0.89976277196271	-4.77938800042102	1.34260090688624
H	-0.58980303118381	-5.34666464088667	0.55765898125133
C	-0.66494660978172	-0.42534152740282	-0.20313152056258
N	-1.61839045009937	0.25528189497411	-0.49069566165795
C	-2.58537059735192	1.02202835903528	0.16075866941330
C	-3.65047381820716	1.49029821044444	-0.63084055126812
C	-4.59288549106135	2.32716468945150	-0.03576117206151
H	-5.41453379973895	2.70882071556308	-0.63624613363803
C	-4.47402420572840	2.69818505873445	1.29845725023601
H	-5.20075606127012	3.37679837786410	1.73585595267301
C	-3.41677809018245	2.22108377162612	2.06790083414987
H	-3.32635652230025	2.51587113171288	3.11032966762200
C	-2.45590055022519	1.37081824045732	1.52187837513914
C	-3.76956241288505	1.09799254751032	-2.07363274188852
H	-4.42405527832875	1.79152597238226	-2.60793195692912
H	-2.79256295790515	1.08114952191571	-2.56660442105039
H	-4.19518463294439	0.09096613089974	-2.17123132053215
C	-1.31878399403165	0.86153238035694	2.35335177044819
H	-1.36034594296442	-0.23011110847885	2.45742983521764
H	-0.34658462340341	1.10149576564139	1.90382868106640
H	-1.35208671860223	1.29829982595301	3.35457151906957

TS3

Coordinates from ORCA-job
tk43b_NEB5aONIOM_OptTS1
E -1503.756307444688

C	-0.49018700104864	0.91882236173759	1.74841208285621
N	-0.01176138857739	1.24372320276928	2.85533800993453
C	1.21929758244285	1.76184964725316	3.22049513367815
C	1.98982762511861	2.59193420758657	2.36912672703868
C	3.23966181969304	3.02508586317633	2.81309608228898
H	3.83697927660352	3.65460937112124	2.15662495567346
C	3.72852941788958	2.67208372376136	4.06723103036896
H	4.70589975308597	3.01924898230823	4.39098108215684
C	2.94755649752517	1.88361534841205	4.91001787419818
H	3.31859686351444	1.60965224002275	5.89529200062507
C	1.69495567689702	1.42520427951232	4.51017538622630
C	1.46898478308392	3.02575486425189	1.03106169234867
H	0.46927837678188	3.46963888109495	1.11797081534471
H	2.13659469359781	3.76885968964788	0.58541892577708
H	1.37913376609556	2.18725268901366	0.33092684905588
C	0.86587243418478	0.55501488155259	5.40908187093137
H	-0.11788473802986	0.99935068790713	5.60232016807200
H	0.67956468990938	-0.42460637788923	4.95141347198640
H	1.36838656110466	0.39813378018220	6.36791087384354
N	-3.03106063468148	-0.00985775422458	-0.49763213471875
N	-0.82477328940046	-1.35273668679606	-2.51320242628603
C	-0.96387166858355	0.44619237948108	0.68533990521173
C	-1.69959696097352	-0.10057623087394	-0.37571940169418
C	-0.72482043045883	-0.73839175043417	-1.23702639669845
C	-3.75364188193653	-0.61033713915247	-1.64495298662012
H	-2.98674785463705	-1.10161690655000	-2.24809115110939
C	-4.76167658362797	-1.66139807421669	-1.17960281741388
H	-5.61218837150527	-1.21681859415904	-0.65106202487821
H	-4.29232598329816	-2.39888111448525	-0.52129757937710
H	-5.15915512233824	-2.18720786872448	-2.05466641906332
C	-4.41312257268631	0.46588346623469	-2.50804688644370
H	-3.68877670886845	1.22903772675514	-2.80849827644003
H	-5.24580399941149	0.95939861329810	-1.99460618789229
H	-4.81486478832373	0.00010324603890	-3.41466732873916
C	-3.86771698279858	0.72593848012679	0.48978825139021
H	-4.88324623143790	0.63845510986950	0.09393179478511
C	-3.54896560020711	2.22006222212057	0.54560735334288
H	-3.52590081092759	2.65111304096023	-0.46074786041060
H	-2.59151025411974	2.42059225006695	1.03179712400970
H	-4.33400462326692	2.72822153700911	1.11622105496759
C	-3.87689209639429	0.06268971337623	1.86710687200744
H	-4.65272612190545	0.53229689770441	2.48160791633096
H	-2.92224815758307	0.17289215154374	2.38673038217935
H	-4.10758798273960	-1.00435958667750	1.78407354158604
C	-0.59968449808417	-2.82674014479460	-2.52069022503421
H	-0.89314137929095	-3.16947188419372	-3.51978758517368
C	0.85728814857922	-3.23606256862438	-2.28384748569096
H	1.16132335946051	-3.03449555678133	-1.25118502807578
H	1.54877951619214	-2.71206346105389	-2.95203748822917
H	0.97176770114912	-4.31146050385647	-2.46153178945341
C	-1.52090995246204	-3.51773857767745	-1.52290769786149
H	-2.57301229165154	-3.33457431375330	-1.75807903672434
H	-1.32714606355213	-3.18296336572757	-0.49703691798402
H	-1.35111372196520	-4.59917724738327	-1.56032004118911
C	-0.10848771621599	-0.59535817196674	-3.57815717340629
H	0.97396513218233	-0.55677046277668	-3.35179081893130
C	-0.28349407992976	-1.24825650755004	-4.94763741615131
H	-1.34631901100245	-1.40242284971765	-5.17213120504195
H	0.23641770868683	-2.20633317546331	-5.03603594554384
H	0.12985007527915	-0.58232036472177	-5.71231676268448
C	-0.62852799183210	0.83952674882824	-3.63018045937903
H	-0.54740311885083	1.34464187837346	-2.66253237419136
H	-1.67879510539409	0.85508507861320	-3.94453566382560
H	-0.04548898288642	1.41491780959954	-4.35702003940705
C	0.49077398667527	-0.44885176564963	-0.65034548174727
N	1.69434048902821	-0.26316131784621	-0.68284648789970
C	2.80950735809896	-0.26383434521809	0.13394214442138
C	3.90347982046606	0.52356646505163	-0.28420009711458
C	5.02431118825590	0.59411900986177	0.53923701924825
H	5.86873357630777	1.20570044806299	0.23094240851348
C	5.06653569868343	-0.09423587704365	1.74832166903826
H	5.94322600191468	-0.02241196429660	2.38598623155109
C	3.98587985461477	-0.87916740672986	2.13910153416162
H	4.02337184553481	-1.42246551698394	3.08031222402415
C	2.84358801677355	-0.99013838885771	1.34600202915641
C	3.84058633904264	1.27059111948746	-1.58306001450619
H	2.97524939299958	1.94352704492785	-1.61314699228122
H	4.74663468573869	1.86390855914185	-1.73259672241128
H	3.73488068444850	0.58434737046572	-2.43217509544257
C	1.70545832538665	-1.87188926501339	1.76608338910973
H	0.77242479855930	-1.31126918975519	1.88091150110072
H	1.51698410483347	-2.65604121080924	1.02248713930171
H	1.93210512646513	-2.35617957988127	2.71952540532370

Cyclobutene 2

Coordinates from ORCA-job
tk43b_CyclobuteneXyl_opt_freq_THF_CoordinateAdjusted
E -1503.795842782477

C	-1.70915445908031	11.38567324832007	6.79701478942078
N	-2.01850765312587	11.81160145127589	7.90490577167730
C	-1.54767593610350	12.88798785795162	8.67218875965834
C	-0.37649561705085	13.61512975357133	8.36656783794676
C	0.00828152266831	14.63460589057743	9.23901717843845
H	0.91321561131196	15.19659176346697	9.02052460266068
C	-0.74003340741070	14.93752958364058	10.37123338309866
H	-0.42079743756457	15.73723534733496	11.03411255149618
C	-1.89280335876993	14.21330273740766	10.65805776075402
H	-2.47626084338109	14.44638533525924	11.54515998187742
C	-2.30962530510541	13.17820051895005	9.82380161737925
C	0.45415783415510	13.31269564278933	7.15681762779591
H	-0.05712699929108	13.60269185126818	6.23082208904958
H	1.40326185422403	13.85406278999272	7.20319771667989
H	0.66938141611740	12.24284806101210	7.07901540961053
C	-3.54409325941500	12.38541832538843	10.14205250350552
H	-4.28895021142217	12.46431185758235	9.34194629099037
H	-3.31283577842940	11.31961389963125	10.25202614021429
H	-3.99745018225421	12.73977299663373	11.07171816370749
N	-2.85570269944428	11.43926485443293	3.59708342946098
N	-0.75207862502004	9.00117301942263	2.74725335910677
C	-1.52663482694690	10.72054902206663	5.68639764761643
C	-1.96400706269864	10.71139078173482	4.26344260301147
C	-1.06045285086365	9.66542819376725	3.93921829304113
C	-3.01924694671044	11.21322032291470	2.13728576203009

H	-2.35497601703051	10.37538536567376	1.90394533375957
C	-4.44985088478857	10.80753895014103	1.78825480099225
H	-5.16162996991346	11.62987208088772	1.92112055551541
H	-4.78050913918560	9.95869559096886	2.39472746917380
H	-4.48889363269253	10.50952020756495	0.73487092630540
C	-2.55393056305182	12.42855736437486	1.33762196185620
H	-1.51943010529958	12.68936595522262	1.58438492103939
H	-3.18650967211190	13.30586867970265	1.51492960687746
H	-2.60154609866780	12.19716845507028	0.26783516408420
C	-3.69435349763519	12.49546875082095	4.21859228106026
H	-4.29787825943921	12.88150652849107	3.39153771333798
C	-2.87365400023673	13.67036549555456	4.74805992849963
H	-2.15415464421393	14.01400867652264	3.99793020337681
H	-2.32728662325159	13.41831141671285	5.65990618026130
H	-3.54662736507927	14.50142796725469	4.98560930138314
C	-4.66252794849481	11.92273741953434	5.25182976998090
H	-5.33258308003713	12.71559949532579	5.60188774213419
H	-4.14262771332503	11.51579115831586	6.12360020713900
H	-5.27211215590200	11.12699014190633	4.81163170585731
C	-1.23082681663441	7.59951181135218	2.64257298724994
H	-0.87577517723734	7.23492909536444	1.66984803190929
C	-0.66937814302453	6.67215500192338	3.72453232272907
H	-1.08095913507075	6.92534245813628	4.70704066232915
H	0.42195294266863	6.73716511216918	3.78642637171084
H	-0.93387968254868	5.63159787979491	3.50173224674654
C	-2.75524850829961	7.55286170763344	2.62933875514436
H	-3.16191537215878	8.11784293325240	1.78439895023954
H	-3.16531664913855	7.97021585657606	3.55733479140359
H	-3.10135774421595	6.51643901186883	2.54772270344504
C	0.59207347421755	9.21722557432090	2.14490465616170
H	1.24155500791979	8.35469611755096	2.37999776899304
C	0.45888797009239	9.32308924769360	0.62296615561665
H	-0.09257948369566	10.23366698516664	0.35681401350269
H	-0.07004218941237	8.46738013268961	0.19161299322908
H	1.44901051676812	9.37090768122255	0.15446553493450
C	1.27207719937532	10.46944448317572	2.68431312026289
H	1.47541439114678	10.39937664790498	3.75610492370791
H	0.65979844318445	11.36109982529607	2.50299021379019
H	2.22779213974068	10.60789037295505	2.16705647369182
C	-0.54415406324836	9.62551105471853	5.29759877413557
N	0.39320146687939	9.00623307375805	5.91101650178409
C	0.59664663271306	9.24937550572210	7.28108682363047
C	1.78154475582688	9.90302879001961	7.67946496982002
C	1.98890756276705	10.16046337249548	9.03487139453611
H	2.89096801767468	10.68642610530923	9.34065919497075
C	1.06102128922851	9.75613396782045	9.99092403493068
H	1.23391102575880	9.96466050603168	11.04332195603371
C	-0.07878967762318	9.06484042608913	9.59243130750898
H	-0.79416048421528	8.72419057924957	10.33813181916530
C	-0.32718702135197	8.79311378390734	8.24507521125395
C	2.78400309354621	10.33755910250721	6.64919090320115
H	2.35949105121260	11.07425094164961	5.95587433831766
H	3.66080443676940	10.78546417792825	7.12609720514172
H	3.11501514164307	9.48999349071346	6.03696494554428
C	-1.54998301767545	8.01692951229331	7.84118730555876
H	-2.28758134308059	8.64490135138290	7.32723444383895
H	-1.29130158490051	7.20412306909818	7.15261480531432
H	-2.03463902663176	7.58352582080088	8.72081439964485

1_{Ad}

Coordinates from ORCA-job
tk44o1a
E -1180.538561536534

N	10.15868578877840	1.09771172532889	5.64056282172655
C	9.89924363580696	0.33404274895919	6.88227884369832
C	11.28208550294004	0.93438802365952	7.39853299825087
H	11.06309059573266	1.67895854578095	8.17245033840800
C	11.34380821774559	1.64656797475890	6.02862434839401
C	12.17471655569706	2.50659940451351	5.44613240955053
C	13.12315780401304	3.24454250524955	5.05158490806729
N	14.07537896837695	3.96078213046525	4.70994738994191
C	14.11899707619352	4.58037413063545	3.37176832199870
C	12.82669695748333	5.35788353195760	3.06824874808598
H	11.97314678694066	4.66654242217735	3.11187971460046
H	12.67499611510771	6.11861365663221	3.84610914067686
C	12.91769828554787	6.01121007954428	1.68097117880964
H	11.99015607780992	6.56457150130811	1.47794008169547
C	13.11152335289635	4.92066622829665	0.61644831105519
H	13.16089600207934	5.37502578659689	-0.38281562113280
H	12.25298301300790	4.23446035829564	0.62143870195247
C	14.40526286413810	4.14463174588071	0.90577517007410
H	14.54344621878474	3.36086618885713	0.14792549896384
C	14.31054706530539	3.49667756570042	2.29521005341688
H	15.22037387680099	2.92432762493499	2.52063233289432
H	13.46213894596849	2.79893289565532	2.33862809820639
C	14.11281620232109	6.97599236282542	1.64873926946741
H	13.97457079976209	7.76944318653656	2.39595806409737
H	14.17751527429797	7.46142165680960	0.66501026729249
C	15.40648873648884	6.20019793031703	1.94010263313233
H	16.26183637973249	6.88944263917134	1.92180437565416
C	15.59939812903648	5.11075361228683	0.87422638879150
H	15.68617929447894	5.56818847224291	-0.12113873242887
H	16.53168382765140	4.56121626863613	1.06351389409391
C	15.30927743510434	5.54684422023916	3.32666962938638
H	15.18081270829281	6.30904396738286	4.10657536010123
H	16.22756162927654	4.99254517359969	3.56113314075270
C	9.29169928576004	1.40584263838625	4.51045970888123
H	8.42071347652281	0.74213711996673	4.61450642299147
C	8.81806597246003	2.86188882005352	4.55287939154462
H	9.67764012995598	3.53746017678139	4.49474841392855
H	8.15421421899459	3.07040041004982	3.70755460148744
H	8.27578690930929	3.07256005746622	5.47953726696306
C	10.00129463851744	1.08153939204818	3.19339996512780
H	10.31434483830311	0.03334231169331	3.16745431302198
H	9.33357193365945	1.26959924088365	2.34648107032844
H	10.89085429818401	1.70976747590853	3.08145069164588
C	8.68577729628174	0.83307103309558	7.65536371850276
H	7.75391628316302	0.61976702489517	7.11743061298115
H	8.62859115986439	0.33008941821622	8.62727280372455
H	8.74498778497235	1.91325674073357	7.82684154352888
C	9.85099664447583	-1.16914742851997	6.68117242001727
H	10.66830418986281	-1.51643451886356	6.04538561452473
H	9.93173539011501	-1.67418575888740	7.64942660547644
H	8.90240502534346	-1.46704359297021	6.21845276409372
N	12.33453069147267	0.08042881117260	7.84442601821418
C	13.20008218047640	-0.56236401901694	6.84296827906591
H	12.64436552429095	-0.51554181985705	5.89680246668080
C	14.54353041196151	0.14132344240284	6.60267842759211
H	15.18045981216441	0.10374744349207	7.49386852652370
H	15.08496269680670	-0.36284478136104	5.79438740487033
H	14.39890770735257	1.18553255315371	6.31501576502111
C	13.42416221057645	-2.03987226607520	7.18341912215192
H	12.47224146965190	-2.56132069452191	7.31848203887646
H	13.98761725226156	-2.53498338037348	6.38486822576456
H	14.00426774633369	-2.15101692017962	8.10668018720900
C	12.91537696227705	0.37250217253102	9.16180353925727
H	13.77456292722482	-0.30238131546749	9.26493655825817
C	11.92695169459011	0.01791785269715	10.27688114709152
H	11.61724209558627	-1.02878528626217	10.19756776356532
H	12.37660188285264	0.18056776407058	11.26277799833104
H	11.02817990973185	0.64413162292934	10.21945985383942
C	13.43538038592384	1.80925974123557	9.33120100143836
H	12.61357541365997	2.53339925748307	9.36567801886318
H	13.98693802965052	1.90230837637180	10.27358347090567
H	14.09849249580834	2.09601461940158	8.51152817803325

Bis(methyleneketenimine) 4

Coordinates from ORCA-job
tk45o1f
E -1812.037048598066

C	0.69085653039625	0.38014810466457	6.73051635225684
C	-0.44438340769226	1.19852982027282	6.85271839950496
H	-0.36660032400250	2.12541514943968	7.41545369518868
C	-1.64817390938417	0.82703390596675	6.28163844856853
H	-2.52837088931277	1.45646103124392	6.37705158700926
N	1.93223277621667	0.70023273517344	7.28549428014345
C	2.20370026856305	1.81145358078255	7.78393200179747
C	2.62263757439869	2.87385152417982	8.31228455471544
C	2.88219435257402	4.10312132710369	8.76171380544528
C	2.08974375113469	5.42412795230913	8.86886060415037
H	1.31309319210085	5.30789735362708	9.63430724521137
C	3.39987783346841	6.00155946306097	9.57277353555258
N	3.96820234175737	4.64970620343220	9.36951896464128
N	1.53513760108187	6.05530530679355	7.71720285138589
C	0.12505343327770	6.45677343178697	7.78251537350758
H	-0.11743940978122	6.85782066001835	6.79009161756970
C	-0.06927244263835	7.59649366254796	8.78780822991407
H	0.15746750794181	7.26680523240087	9.80904490375732
H	-1.10682627674774	7.94809423484117	8.78110335039696
H	0.58945191039246	8.43679369271469	8.54707109034558

C	-0.85578852720820	5.30745155055155	8.06400431330522
H	-0.74196063665988	4.50312251741271	7.33366396120369
H	-1.88916921060289	5.66930806484035	8.01861670505405
H	-0.70362926074336	4.88231694416007	9.06267857644085
C	2.11800878351086	5.78391097440055	6.39458939261943
H	3.10827648155336	5.35089490523665	6.59048253445516
C	2.32140882736746	7.08114425453000	5.60300561062899
H	1.36201902073012	7.53979002800276	5.33785700172601
H	2.85141536593418	6.87619775380001	4.66642328051349
H	2.89652381973624	7.81078994352496	6.18006267439973
C	1.34376865520304	4.76185809596720	5.55072130421520
H	1.22725958814365	3.81528370169010	6.08405823464854
H	1.88470409606492	4.56204001867488	4.61918850938434
H	0.35161661653976	5.13823744687106	5.27655634922485
C	4.13687111945397	7.09370956016977	8.82106829994236
H	5.08213446194594	7.33697370454123	9.32090893983096
H	3.52361120766961	8.00035186210701	8.79098951059432
H	4.35665863342233	6.79536855014387	7.79365247285730
C	3.20959115293469	6.35873487316562	11.04027324055863
H	2.64631356936380	5.57986720180548	11.56546224111049
H	2.66029397176274	7.30292264856043	11.12709869775426
H	4.17313590422696	6.48906518269639	11.54785872202276
C	5.18633863493925	4.03757701491601	9.88593067509006
H	5.76260937407802	4.86029009072236	10.33401541701614
C	6.00177728125767	3.43138278235022	8.74124708524705
H	5.43585281069909	2.62914368880456	8.25745224044518
H	6.93777008525669	3.01286392301529	9.12464333709691
H	6.23968368055441	4.18956312614695	7.98888953816986
C	4.86961112749334	3.00193728165045	10.96846634686233
H	4.31221405285840	3.45472588430664	11.79410942889446
H	5.79608442130541	2.57656597776655	11.36736452279674
H	4.26809119505201	2.18974020362819	10.54832085352793
C	-1.76297598649491	-0.37989146980586	5.57579684353728
C	-0.62779079683275	-1.19831891456809	5.45371394421311
H	-0.70556342436551	-2.12517607106932	4.89094384401402
C	0.57600837867432	-0.82677797330418	6.02475051764784
H	1.45623633502198	-1.45614702813935	5.92925626224067
N	-3.00421549371423	-0.69984125348250	5.02036405512431
C	-3.27561147898597	-1.81117999396556	4.52212641346517
C	-3.69401311254975	-2.87381738417642	3.99390844194562
C	-3.95339432339325	-4.10321081473030	3.54483941948248
C	-3.16110819330448	-5.42448291331450	3.43946366693319
H	-2.38337814350484	-5.30884253510992	2.67501587753618
C	-4.47042658388128	-6.00197444943333	2.73407749914788
N	-5.03877082659604	-4.64987850858599	2.93596447248091
N	-2.60831189851151	-6.05512709776601	4.59223197361282
C	-1.19839137669546	-6.45741403819630	4.52897377418607
H	-0.95737196918429	-6.85795396666311	5.52195984371382
C	-1.00341523621031	-7.59790739165549	3.52467740284049
H	-1.22848668235439	-7.26874571435157	2.50290413171089
H	0.03389470146655	-7.95018875604258	3.53307916139367
H	-1.66301510013882	-8.43762444546910	3.76503400619523
C	-0.21650517720691	-5.30884290884148	4.24798403088444
H	-0.33035037885842	-4.50422723697733	4.97799170190519
H	0.81659364954371	-5.67139409366859	4.29431592355763
H	-0.36753382921160	-4.88390429182006	3.24905432820249
C	-3.19270983381064	-5.78249678577127	5.91389108939276
H	-4.18247060490748	-5.34903630301594	5.71645673453752
C	-3.39795612906445	-7.07906609731128	6.70611613434311
H	-2.43922012870215	-7.53812019723092	6.97290415689217
H	-3.92909777724840	-6.87312294783098	7.64184104265464
H	-3.97276036149994	-7.80876064791024	6.12882168872958
C	-2.41891693192220	-4.76038855507220	6.75810241811805
H	-2.30089789138969	-3.81432871199443	6.22419823389374
H	-2.96100494194327	-4.55941709538196	7.68871422302266
H	-1.42744660060055	-5.13732815857955	7.03393977564231
C	-5.20860265382222	-7.09365720807823	3.48529188658879
H	-6.15314301271656	-7.33718118267289	2.98422073984054
H	-4.59540484159561	-8.00028917265757	3.51680622606521
H	-5.42984501323362	-6.79470106214235	4.51221075854855
C	-4.27829471447044	-6.35975161400306	1.26696762492169
H	-3.71413234039995	-5.58122387405887	0.74221637239786
H	-3.72913959058647	-7.30412230120688	1.18119933976146
H	-5.24121050413469	-6.49003289788529	0.75816546532427
C	-6.25614251687432	-4.03774300653333	2.41772953215138
H	-6.83205879313743	-4.86059352997069	1.96942784397234
C	-7.07276249157349	-3.43070623505087	3.56111592540322
H	-6.50723414517255	-2.62828549405533	4.04507215612706
H	-8.00824617393819	-3.01224102641536	3.17641227336160
H	-7.31165810634088	-4.18839530725001	4.31364980349228
C	-5.93785932079093	-3.00279096500293	1.33500142422468
H	-5.37956045848087	-3.45617487173709	0.51029212401730
H	-6.86375048429229	-2.57743156051348	0.93473225373797
H	-5.33665540765083	-2.19049806805012	1.75541430816488

Cyclobutene 11

Coordinates from ORCA-job
opt_cyclobuteneCO2Me
E -1713.568542459554

C	0.01175495974726	-0.72529874854134	-1.16433712210046
C	0.01443706644621	-2.07244763754798	-1.68573792443294
C	1.38983999598571	-2.23090507395343	-1.67356823074678
C	1.52399615796520	-0.91864853422419	-0.95018993236084
C	-1.07428563580928	-2.88359137982129	-2.26624542416839
C	-0.99742086751951	0.20543406222961	-1.02936617848182
N	2.47348882437141	-0.55682094696888	-0.20365700094982
C	3.69232184674512	2.27753940688436	1.99442810030378
C	2.29445297385488	-3.26775333525449	-2.08341976796608
C	-1.35800087811605	1.61574563853699	-1.55402323148814
C	-2.69075010713526	1.44505021808351	-0.70354702035420
N	-2.24741686406731	0.03437982108267	-0.54603603586966
N	-0.43998995796232	2.73082918468192	-1.45764439442706
C	0.85508936915320	2.35525333951493	-2.11288609120792
C	-0.91371122929827	4.06957797378832	-1.93779971809327
C	-2.34010563734964	4.14693672753691	-2.47755865957260
C	-0.71985461591173	5.11815710326491	-0.83926966403181
C	-2.83405474700147	-1.15529544281498	0.06640762875709
C	0.73355880273517	1.97733040214936	-3.59684090742749
C	1.92643675979806	3.42714505444745	-1.92198713275607
C	-3.30670954152938	-0.88463858609158	1.49627632381011
C	-3.94945421892486	-1.78705991897684	-0.77292185664894
C	-4.02929278319424	1.53130238222799	-1.42238850832512
C	-2.67112245088430	2.22456941964347	0.60287032458890
O	-1.75957880243984	-2.49823429679240	-3.19090965968077
O	-1.21801170161168	-4.06976772750020	-1.65356140939725
C	-2.18043758430497	-4.95792537699695	-2.26201646925659
O	3.49363109492912	-3.30834852422295	-1.88065369659300
O	1.62637804218638	-4.24195115860106	-2.79225066612858
C	2.46279930857576	-5.31069484281713	-3.25694113782646
C	2.35769479392629	2.97632738025065	2.29363500092268
C	1.26637257120937	1.92157525585923	2.52408084220752
C	1.11231238850094	1.05905846643975	1.25897799507932
C	2.44258427275464	0.36085817821136	0.92652532711927
C	3.55265442285719	1.41022715653732	0.73505370605462
C	1.65303116486444	1.03415282153287	3.71415340496046
C	4.07785697325628	1.38815756218069	3.18550556641583
C	2.83316523241340	-0.52103666482532	2.14203857913452
C	2.98353986845061	0.33387440325059	3.40766143993011
H	4.47461043330936	3.03075425340173	1.82696414324218
H	-1.65305761023236	1.39644918456367	-2.60015453174224
H	1.20710677725725	1.46368044008976	-1.58483651200427
H	-0.27372137546913	4.35541005266914	-2.78264452270447
H	-2.46456348584248	5.10914559237115	-2.98467389750182
H	-2.56664125385065	3.36136509539654	-3.20400884284093
H	-3.07986993901799	4.11550681572617	-1.67451920871316
H	-1.36120669349599	4.89965718304303	0.02035008373786
H	-0.97464278321451	6.11723743283138	-1.21304975637594
H	0.31242082256193	5.13732442716169	-0.48391328172377
H	-1.99137534017990	-1.86087791462160	0.11463960633013
H	1.72883805314406	1.76746130122241	-4.00121667004911
H	0.13334826459713	1.07421354259357	-3.74311213900247
H	0.29845303182887	2.78937531915098	-4.19213166306376
H	2.89083145286543	3.02035893290340	-2.24179983754003
H	2.01285068566274	3.72188903195432	-0.87298630555167
H	1.74203313859520	4.32514421431060	-2.52087001090454
H	-3.58777082227987	-1.83305031013997	1.96468988906787
H	-2.51869555185123	-0.42219622436496	2.09764399680286
H	-4.18950832007022	-0.23750709255811	1.51004346312025
H	-4.17547590450975	-2.78412209733678	-0.37912372685503
H	-3.65387871445895	-1.88451348805278	-1.81987997527115
H	-4.86873209912704	-1.19608442654019	-0.72007919412034
H	-4.80281772286449	1.06771240581307	-0.80107543337539
H	-4.33848417226732	2.56177415923754	-1.59996122047550
H	-3.99660761596555	1.00028519400852	-2.37928917418912
H	-1.70648162904959	2.11440348644654	1.10590820255048
H	-2.82554492171131	3.28680539272762	0.39449483214389
H	-3.46985227881305	1.90054873816149	1.27508940375249
H	-2.17344038097105	-5.85719396498439	-1.64604280576406
H	-3.17455225393240	-4.50226419949835	-2.27408743056916
H	-1.87782127901822	-5.18861651707639	-3.28725209851028
H	1.80064840233440	-5.98872079976915	-3.79868209286131
H	3.24699967262293	-4.93198580069329	-3.92035097513523
H	2.93637518206757	-5.82870723206333	-2.41668124439748

H	2.07357533234768	3.62870530308195	1.45621742631020
H	2.45796079876989	3.61455738744328	3.18259325402962
H	0.31100580955427	2.42509841402364	2.73253051623412
H	0.77883855560026	1.69986635873729	0.43422504070812
H	0.33562286705637	0.29806770050413	1.41763520991227
H	4.49187505891410	0.88738262745326	0.51508354231526
H	3.31702911629521	2.03339565522282	-0.13384887792863
H	0.86796259541055	0.28707127465321	3.89678455041261
H	1.74500471088506	1.64196296730897	4.62514592122974
H	4.20101605551653	2.00035447913332	4.08975264563012
H	5.03969471824902	0.89543134007012	2.98977015726039
H	3.76858767254413	-1.04243139963518	1.90676429604019
H	2.05913008333620	-1.28745716453144	2.28426712379299
H	3.26302073920037	-0.31763065393448	4.24728339555519

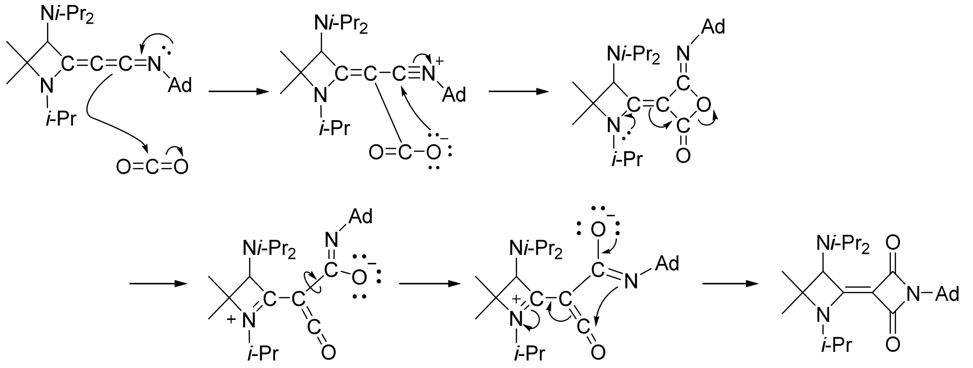

Scheme S1. Possible mechanism of the reaction of $\mathbf{1_{Ad}}$ with CO_2.[66]

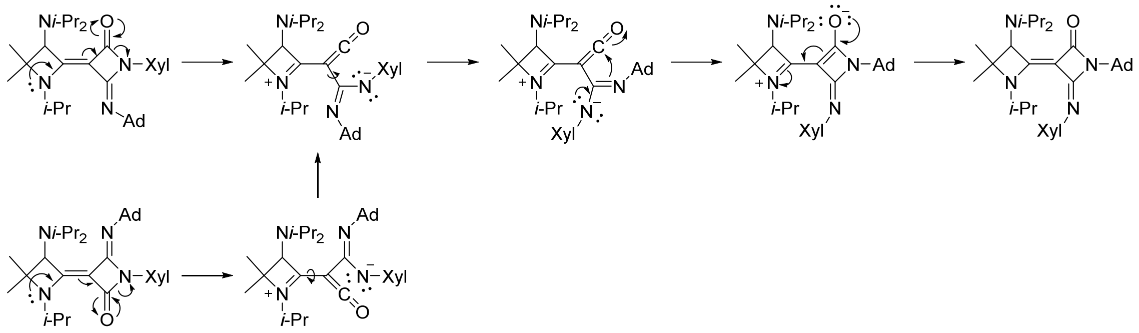

Scheme S2. Possible mechanism of the formation of **9**.

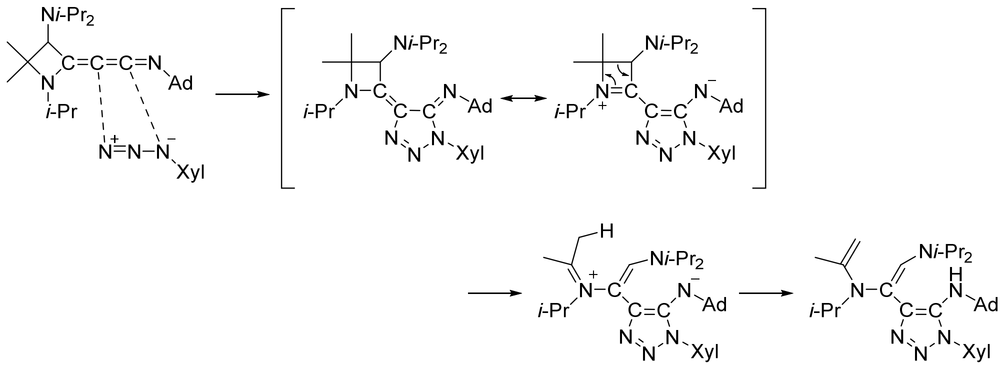

Scheme S3. Possible mechanism of the reaction of $\mathbf{1_{Ad}}$ with xylyl azide.[85-87]

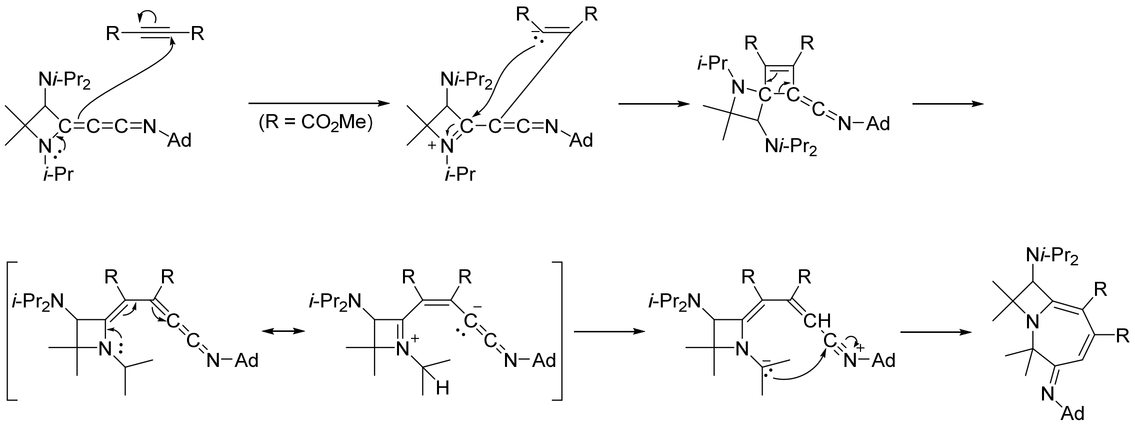

Scheme S4. Possible mechanism of the reaction of $\mathbf{1}_{\mathbf{Ad}}$ with dimethyl acetylenedicarboxylate (R = CO_2Me).[24]

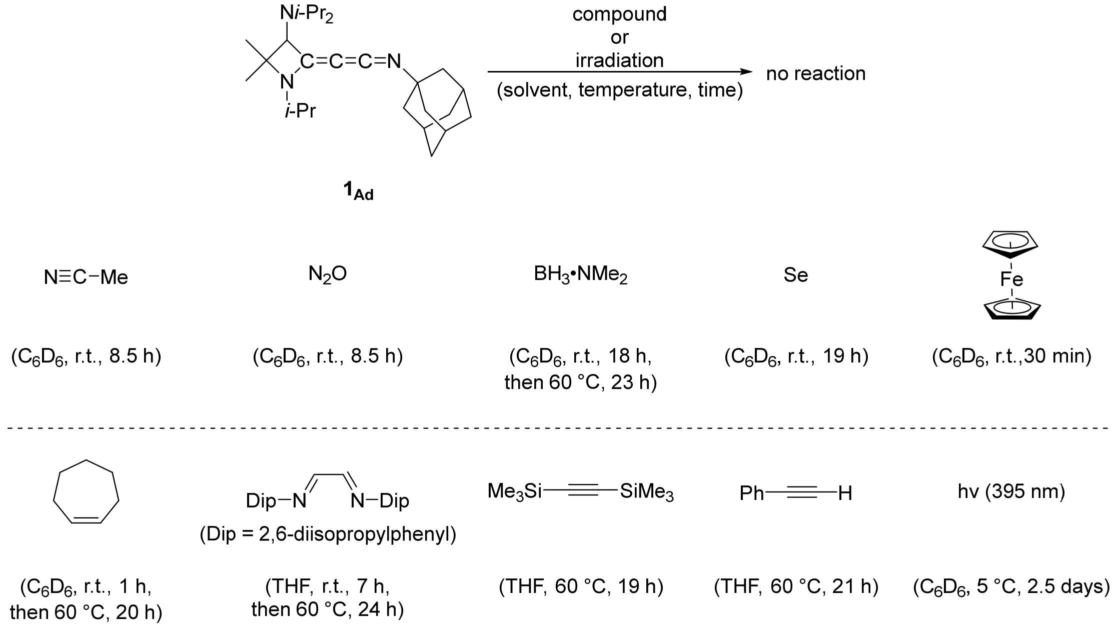

Scheme S5. Unreactive reagents toward $\mathbf{1}_{\mathbf{Ad}}$ and the reaction conditions.

9. References

68. G. R. Fulmer, A. J. M. Miller, N. H. Sherden, H. E. Gottlieb, A. Nudelman, B. M. Stoltz, J. E. Bercaw, K. I. Goldberg, “NMR Chemical Shifts of Trace Impurities: Common Laboratory Solvents, Organics, and Gases in Deuterated Solvents Relevant to the Organometallic Chemist.” *Organometallics* **29**, (2010): 2176.
69. J. M. Favale Jr., E. O. Danilov, J. E. Yarnell, F. N. Castellano, “Photophysical Processes in Rhenium(I) Diiminetricarbonyl Arylisocyanides Featuring Three Interacting Triplet Excited States.” *Inorganic Chemistry* **58**, (2019): 8750.
70. C. Wang, X. Kang, S. Dai, F. Cui, Y. Li, H. Mu, S. Mecking, Z. Jian, “Efficient Suppression of Chain Transfer and Branching via Cs-Type Shielding in a Neutral Nickel(II) Catalyst.” *Angewandte Chemie International Edition* **60**, (2021): 4018.
71. M. Tanabiki, K. Tsuchiya, K. Kumanomido, K. Matsubara, Y. Motoyama, H. Nagashima, “Nickel(II) Isocyanide Complexes as Ethylene Polymerization Catalysts.” *Organometallics* **23**, (2004): 3976.
72. J. Liu, T. Yang, Z.-P. Wang, P.-L. Wang, J. Feng, S.-Y. Ding, W. Wang, “Pyrimidazole-Based Covalent Organic Frameworks: Integrating Functionality and Ultrastability via Isocyanide Chemistry.” *Journal of the American Chemical Society* **142**, (2020): 20956.
73. M. J. Harper, E. J. Emmett, J. F. Bower, C. A. Russell, “Oxidative 1,2-Difunctionalization of Ethylene via Gold-Catalyzed Oxyarylation.” *Journal of the American Chemical Society* **139**, (2017): 12386.
74. G. M. Sheldrick, SADABS, Empirical absorption correction program; University of Göttingen, Göttingen (Germany), 1996.
75. G. M. Sheldrick, SHELXL-2018, 19, Program for the refinement of crystal structures; University of Göttingen, Göttingen (Germany), 2018, 2019.
76. K. Wakita, Yadokari-XG, Software for crystal structure analyses, 2001.
77. C. Kabuto, S. Akine, T. Nemoto, E. Kwon, “Release of software (Yadokari-XG 2009) for crystal structure analyses.” *Journal of the Crystallographic Society of Japan* **51**, (2009): 218.
78. M. J. Frisch, G. W. Trucks, H. B. Schlegel, G. E. Scuseria, M. A. Robb, J. R. Cheeseman, G. Scalmani, V. Barone, G. A. Petersson, H. Nakatsuji, X. Li, M. Caricato, A. V. Marenich, J. Bloino, B. G. Janesko, R. Gomperts, B. Mennucci, H. P. Hratchian, J. V. Ortiz, A. F. Izmaylov, J. L. Sonnenberg, D. Williams-Young, F. Ding, F. Lipparini, F. Egidi, J. Goings, B. Peng, A. Petrone, T. Henderson, D. Ranasinghe, V. G. Zakrzewski, J. Gao, N. Rega, G. Zheng, W. Liang, M. Hada, M. Ehara, K. Toyota, R. Fukuda, J. Hasegawa, M. Ishida, T. Nakajima, Y. Honda, O. Kitao, H. Nakai, T. Vreven, K. Throssell, J. A. Montgomery Jr., J. E. Peralta, F. Ogliaro, M. J. Bearpark, J. J. Heyd, E. N. Brothers, K. N. Kudin, V. N. Staroverov, T. A. Keith, R. Kobayashi, J. Normand, K. Raghavachari, A. P. Rendell, J. C. Burant, S. S. Iyengar, J. Tomasi, M. Cossi, J. M. Millam, M. Klene, C. Adamo, R. Cammi, J. W. Ochterski, R. L. Martin, K. Morokuma, O. Farkas, J. B. Foresman, D. J. Fox, Gaussian 16, Revision C.01, Gaussian, Inc., Wallingford, CT, 2016.
79. F. Neese, “The ORCA program system.” *WIREs Computational Molecular Science* **2**, (2012): 73.
80. F. Neese, “Software update: The ORCA program system—Version 5.0.” *Computational Molecular Science* **12**, (2022): e1606.
81. F. Neese, ORCA Program System, Version 6.1.0, Max Planck Institute for Chemical Energy Conversion: Mülheim an der Ruhr, Germany, 2025; can be found under https://orcaforum.kofo.mpg.de (accessed: 2025-11-26).
82. T. Lu, F. Chen, “Multiwfn: A Multifunctional Wavefunction Analyzer.” *Journal of Computational Chemistry* **33**, (2012): 580.
83. E. D. Glendening, J. K. Badenhoop, A. E. Reed, J. E. Carpenter, J. A. Bohmann, C. M. Morales, P. Karafiloglou, C. R. Landis, F. Weinhold, NBO 7.0, Theoretical Chemistry Institute, University of Wisconsin: Madison, 2018.
84. V. Asgeirsson, B. O. Birgisson, R. Bjornsson, U. Becker, F. Neese, C. Riplinger, H. Jonsson, “Nudged Elastic Band Method for Molecular Reactions Using EnergyWeighted Springs Combined with Eigenvector Following.” *Journal of Chemical Theory and Computation* **17**, (2021): 4929.
85. D. Caine, T. L. Smith Jr., “Extension of the Woodward-Hoffmann rules to heterocyclic systems: stereospecific thermal isomerization of 1-azacyclobutene 1-oxides.” *Journal of the American Chemical Society* **102**, (1980): 7570.
86. G. Stork, A. Brizzolara, H. Landesman, J. Szmuszkovics, R. Terrell, “The Enamine Alkylation and Acylation of Carbonyl Compounds.” *Journal of the American Chemical Society* **85**, (1963): 207.
87. J. L. Freeman, M. A. Brimble, D. P. Frukert, “Convenient access to 5-membered cyclic iminium ions: evidence for a stepwise [4 + 2] cycloaddition mechanism.” *Organic & Biomolecular Chemistry* **17**, (2019): 2705.
